# The Rfg1 and Bcr1 transcription factors regulate acidic pH–induced filamentous growth in *Candida albicans*

**DOI:** 10.1128/spectrum.01789-23

**Published:** 2023-11-07

**Authors:** Guobo Guan, Shuaihu Li, Jian Bing, Ling Liu, Li Tao

**Affiliations:** 1 Department of Infectious Diseases, Huashan Hospital, Shanghai Institute of Infectious Disease and Biosecurity and State Key Laboratory of Genetic Engineering, School of Life Sciences, Fudan University, Shanghai, China; 2 State Key Laboratory of Mycology, Institute of Microbiology, Chinese Academy of Sciences, Beijing, China; University of Wisconsin–Madison School of Medicine and Public Health, Madison, Wisconsin, USA

**Keywords:** *Candida albicans*, phenotypic transition, Rfg1, Bcr1

## Abstract

The human fungal pathogen *Candida albicans* colonizes and infects various host sites with diverse environmental pH. Adaptation to these diverse pH conditions plays a crucial role in its success as a commensal and pathogen. The conserved Rim101 pH sensing pathway is responsible for neutral–alkaline pH responses in *C. albicans*. In this study, we identified a novel Rfg1-Bcr1 regulatory pathway that governs acidic pH responses and regulates filamentation in *C. albicans*. A null mutant library was screened, and we have identified Rfg1 and Bcr1 as key regulators of filamentation under acidic pH conditions. Rfg1 directly binds to the promoter region of Bcr1 to regulate its transcriptional expression, which in turn suppresses the filamentation of *C. albicans*. *PHR1*, an alkaline pH response gene, is significantly activated by the absence of Rfg1, indicating that Rfg1 regulates acidic pH response through the Rim101-Phr1 pathway. Moreover, the cAMP signaling pathway, transcription factors Efg1 and Flo8, and the hyphal-specific G1 cyclin Hgc1 play critical roles in the regulation. Our findings provide new insights into the mechanisms underlying the acidic pH response of *C. albicans,* which reflects its elaborate regulatory control of environmental adaptation.

**IMPORTANCE:**

*Candida albicans* is a human commensal and frequent pathogen that encounters a wide range of pH stresses. The ability of *C. albicans* to adapt to changes in extracellular pH is crucial for its success in colonization and pathogenesis. The Rim101 pH sensing pathway is well known to govern neutral–alkaline pH responses in this pathogen. Here, we report a novel Rfg1-Bcr1 regulatory pathway that governs acidic pH responses and regulates filamentous growth in *C. albicans*. In addition, the Rim101-Phr1 pathway, cAMP signaling pathway, transcription factors Efg1 and Flo8, and hyphal-specific G1 cyclin Hgc1 cooperate with this regulation. Our findings provide new insights into the regulatory mechanism of acidic pH response in *C. albicans*.

## INTRODUCTION

Microorganisms are often exposed to diverse microenvironments. The ability to sense and respond to environmental stresses is necessary for their survival and morphogenesis. *Candida albicans*, which is the most common commensal and predominant fungal pathogen in humans, thrives in several human niches, such as the gastrointestinal tract (GI), oral cavity, and genital tract (1 – 4). In these host sites, pH is one of the major environmental factors that can vary widely, from acidic (pH 2–6, stomach, vagina, and oral cavity) to neutral–alkaline (pH 7–8, bloodstream, large intestine, and colon) (5 – 8). Thus, adaptation to pH fluctuations is critical for the physiology and pathogenicity of *C. albicans* (4).

In general, *C. albicans* is harmless to healthy individuals; however, it can invade various body niches and cause life-threatening disseminated candidiasis in patients with an immune-compromised state (9, 10). In response to environmental changes, this event is mainly attributed to its ability to switch between the budding yeast form and the filamentous growth form (11 – 14). Yeast cells are the primary commensal form and predominate in bloodstream infections, whereas filamentous cells are the pathogenic form and better at adhering to and invading host niches (15, 16). In *C. albicans*, acidic pH favors the unicellular budding of yeasts, whereas alkaline pH favors filamentous growth form (11, 17). Thus, acidic-to-neutral/alkaline pH promotes the transition from a budding yeast form to a filamentous growth form, a key event that is crucial for the virulence of *C. albicans*.

Given that the pH of human blood is 7.4 ± 0.1, the human body environment is primarily considered a neutral–alkaline pH condition (18). The signaling pathways and effectors responsible for adapting to a neutral–alkaline pH environment have received the most attention. The PacC/Rim101 signaling pathway is the most conserved and the best-investigated fungal pH response pathway (19). It has been found in ascomycetes (e.g., PacC in *Aspergillus nidulans* and *Saccharomyces cerevisiae*) and basidiomycetes (20). In *C. albicans*, the Rim101 pathway regulates both morphological transitions and virulence (18, 21). Neutral–alkaline pH is sensed by the plasma membrane complex composed of Rim9, Rim21, Rim23, and Rim8 (22, 23). When these proteins are triggered, the interaction of downstream effectors is triggered and results in the proteolytic removal of the inhibitory C-terminal domain of Rim101 (19, 24). This truncated Rim101 subsequently promotes a series of neutral–alkaline pH responses and causes filamentation (25, 26). Phr1 and Phr2 are functionally redundant cell wall glycosidases, and their expressions are regulated by environmental pH in opposite directions through Rim101. Phr1 is expressed exclusively under neutral–alkaline conditions (pH >5.5), whereas Phr2 is expressed preferentially under acidic conditions (pH <5.5) (19, 27). The *PHR1* deletion mutant exhibits apical growth defects of either yeast or hyphal growth forms under neutral–alkaline conditions (28), whereas the *PHR2* deletion mutant is unable to grow under acidic conditions (29).

Viewing the host environment as having a rigorously neutral–alkaline pH condition is unwise. Many bacterial pathogens can sense and respond to acidic pH conditions in hosts. For instance, an acid-tolerance response (ATR) system has been best identified in *Vibrio cholerae*, *Listeria monocytogenes*, and *Salmonella enterica* serovar Typhimurium, which can sense and induce a series of acid-shock responses that enable the bacteria to survive at acidic pH (30 – 32). *Shigella* spp. possess both the ATR system and a high basal capacity of acid resistance (33). Other bacteria pathogens such as *Helicobacter pylori* can survive in the stomach by expressing a urease that creates a hospitable pH condition through ammonia production (34). *C. albicans,* being the most common commensal and a predominant fungal pathogen, encounters diverse acidic pH conditions, including the stomach, vagina, and oral cavity (1 – 4). However, the mechanisms by which this pathogen adapts to acidic pH conditions and causes disease have not been sufficiently explored. Therefore, understanding the acidic pH response of *C. albicans* is important and helps in identifying potential antifungal targets and preventing infection. In this study, we identified a novel Rfg1-Bcr1-mediated acidic pH regulatory pathway in *C. albicans*. Rfg1 interacts with Bcr1 to regulate filamentation under acidic pH conditions. *PHR1*, an alkaline pH response gene, is significantly activated by the absence of Rfg1, indicating a key role of *PHR1* in Rfg1-modulated filamentation. The cAMP signaling pathway, transcription factors Efg1 and Flo8, and the hyphal-specific G1 cyclin Hgc1 are essential in this regulation. We identified a mechanism by which *C. albicans* could adapt to and survive in acidic environments, uncovered a Rfg1-mediated regulatory pathway that regulates this response, and shed new light on the environmental adaptation of *C. albicans*.

## RESULTS

### Identification of acidic pH sensing genes by screening a null mutant library

**Fig 1:**
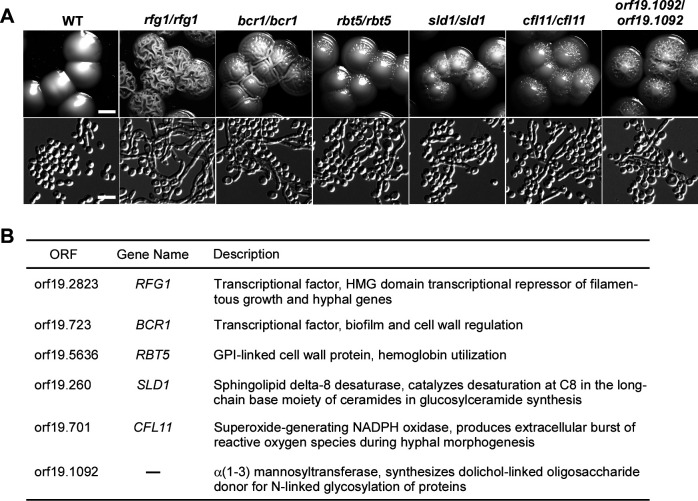
Library screening identified regulators governing filamentous growth in response to acidic pH. (A) Colony and cellular morphologies of the WT (SN250), *rfg1/rfg1*, *bcr1/bcr1*, *rbt5/rbt5*, *sld1/sld1*, *cfl11/cfl11,* and *orf19.1092-/-*mutants on YPD-K medium (pH 4). Colonies were grown at 30°C for 6 days. Scale bar for colonies, 2.5 mm; scale bar for cells, 10 µm. (B) Functional descriptions of the acidic pH sensing genes.

*C. albicans* colonizes and infects diverse acidic niches in the human body, including the stomach, vagina, and oral cavity (1 – 4). Adaptation to these acidic environments is critical for their survival and virulence. Thus, investigating regulators and signaling pathways that govern acidic pH response is necessary. Considering that acidic pH inhibits the filamentation of *C. albicans*, we screened a library containing approximately 505 null mutants to look for mutants that can enhance filamentation under acidic pH conditions (34, 35). Considering that the physiological temperature of mammalian skin, nails, external vaginal region, and baby diapers is approximately 30°C, we cultured the library mutants on the YPD-K (pH 4) medium at 30°C. As shown in Fig. 1A, six gene null mutants, including *rfg1/rfg1*, *bcr1/bcr1*, *rbt5/rbt5*, *sld1/sld1*, *cfl11/cfl11*, and *orf19.1092/orf19.1092*, formed obvious wrinkled colonies and displayed filamentation. Transcription factors Rfg1 and Bcr1 are involved in the regulation of filamentation and biofilm formation in *C. albicans* (Fig. 1B) (32 – 34, 36). More importantly, *rfg1/rfg1* mutants could undergo the most robust filamentation under acidic pH conditions, leading to the formation of wrinkled colonies by day 2, while other mutants required up to 6 days to show similar growth.

Figure S1A presents an example of a clinical isolate (HJ25) that could undergo filamentation at acidic pH. The HJ25 strain displayed robust filamentation on the YPD-K medium of pH 4. As expected, the expression level of *RFG1* in the HJ25 strain was significantly reduced under acidic pH (pH 4) compared to neutral pH (pH 7) conditions (Fig. S1B). To verify the role of Rfg1 in the filamentation of HJ25, we overexpressed *RFG1* in the HJ25 strain and observed suppression in filamentation under acidic pH conditions (Fig. S1C). These results suggest that Rfg1 is a potential filamentation regulator in response to acidic environmental pH.

### Rfg1 is a key filamentous inhibitor in acidic pH response

**Fig 2:**
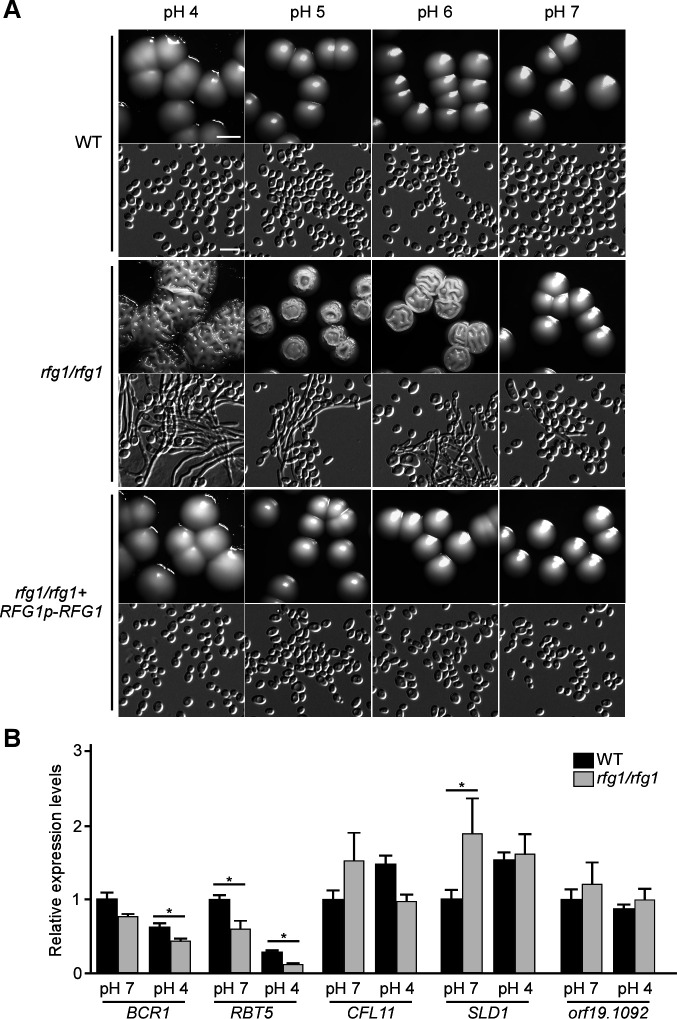
Deletion of *RFG1* results in filamentous growth under acidic pH conditions. (A) Cells of the WT (SN250), *rfg1/rfg1* mutant, and *rfg1/rfg1 + RFG1p-RFG1* reconstituted strain were grown on YPD-K medium (pH 4, pH 5, pH 6, and pH 7). The colony and cellular images were taken after 3 days of growth at 30°C. Scale bar for colonies, 2.5 mm; scale bar for cells, 10 µm. (B) Relative expression levels of *BCR1*, *RBT5*, *CFL11*, *SLD1*, and *orf19.1092* in the WT (SN250) and *rfg1/rfg1* strains on YPD-K medium (pH 4 or pH 7) at 30°C. Transcript levels of each gene in the WT under the pH 7 condition were set as “1.” Data are presented as the mean ± SEM. *P < 0.05 (Student’s *t*-test, two-tailed).

To extensively investigate the function of Rfg1 in regulating filamentation under acidic pH conditions, its filamentation ability was examined under diverse pH conditions (pH 4–7). As shown in Fig. 2A, the *rfg1/rfg1* mutant underwent robust filamentation at pH 4 and exhibited a gradual decrease in filament formation along with pH increase (pH 4–6). No noticeable filamentous phenotypes were observed at pH 7. As expected, the *RFG1*-reconstituted strain completely lost the ability to undergo filamentation at all the pH conditions tested (Fig. 2A). These results indicate that Rfg1 regulates the filamentation of *C. albicans* in response to the environmental pH, and it plays a specific role in suppressing filamentation at acidic pH.

Temperature, another host-related environmental factor, regulates filamentation in *C. albicans*. High temperatures stimulate yeast cells to undergo filamentation, whereas low temperatures inhibit this process (17). Accordingly, we tested the ability of the *rfg1/rfg1* mutant to undergo filamentation at low temperatures. As shown in Fig. S2, the *rfg1/rfg1* mutant exhibited obvious filamentation at 25°C under acidic pH conditions (pH 4–6). These results indicate that Rfg1-deletion-caused filamentation under acidic pH is independent of environmental temperature.

### Rfg1 interacted with Bcr1 to regulate filamentation in response to acidic pH

**Fig 3:**
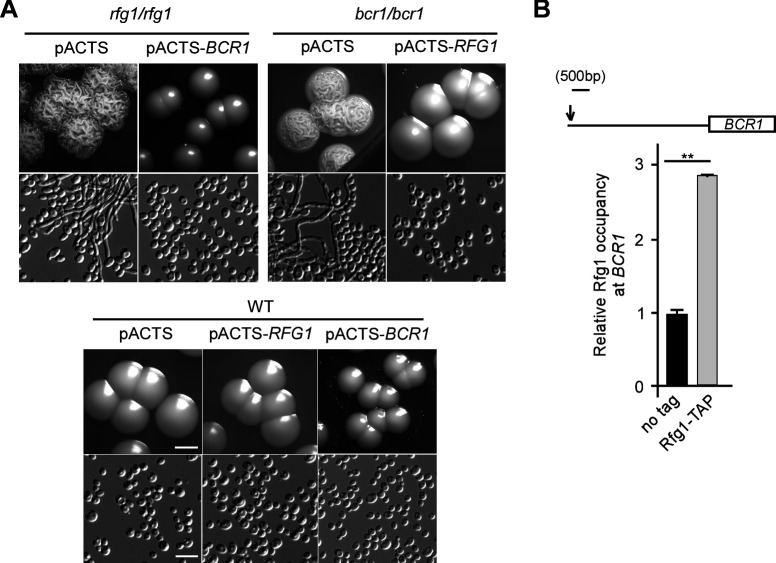
Rfg1 interacted with Bcr1 to regulate filamentous growth in response to acidic pH. (A) Morphologies of the *BCR1*-overexpressing strains (*rfg1/rfg1* + pACTS-*BCR1*), *RFG1*-overexpressing strains (*bcr1/bcr1* + pACTS-*RFG1*), and the corresponding controls (WT + pACTS, WT + pACTS-*BCR1*, and WT + pACTS-*RFG1*) on YPD-K medium (pH 4) at 30°C. Scale bar for colonies, 2.5 mm; scale bar for cells, 10 µm. (B) Rfg1 binds to the promoters of *BCR1*. The Rfg1-TAP and untagged Rfg1 strains were grown on YPD-K medium (pH 4) at 30°C for 2 days. Cells were collected and washed for ChIP assays. ChIP DNA was quantitated by qPCR with primers at the promotor region of *BCR1*. The *ADE2* coding region served as a negative control. The Rfg1-TAP occupancy at *BCR1* promoter is presented as a ratio of *BCR1* IP (bound/input) versus the *ADE2* IP (bound/input). The value in cells of untagged Rfg1 strain was set to 1. Data are presented as the mean ± SEM of three independent qPCR reactions. **P < 0.01 (Student’s *t*-test, two-tailed).

Besides Rfg1, five other regulators, such as Bcr1, Rbt5, Sld1, Cfl11, and orf19.1092, might also function in the regulation of filamentation in response to acidic pH conditions (Fig. 1). To investigate whether they were regulated by or interacted with Rfg1, we tested the relative expression levels of these genes in the *rfg1/rfg1* mutant using quantitative reverse-transcription polymerase chain reaction (qRT-PCR) assays. As shown in Fig. 2B, the expression levels of *BCR1* and *RBT5* were significantly reduced in the *rfg1/rfg1* mutant on the YPD-K medium (pH 4), suggesting a potential role of Rfg1 in regulating *BCR1* and *RBT5* under acidic pH conditions. Rbt5 is a glycosylphosphatidylinositol (GPI)-modified cell wall protein, which is regulated by Rfg1 (35). Bcr1 is a key transcription factor required for the regulation of cell-surface genes, biofilm formation, and opaque cell filamentation (37, 38). Given the important role of Bcr1 in the transcriptional control of filamentation, we hypothesized that there might be a genetic interaction between Rfg1 and Bcr1 when *C. albicans* cells were exposed to acidic pH. To test this, the *RFG1* gene was first overexpressed in the *bcr1/bcr1* mutant under the control of the *ACT1* promoter (39). As shown in Fig. 3A, the overexpressed *RFG1* in the *bcr1/bcr1* mutant inhibited filamentation under acidic pH conditions. Furthermore, the *BCR1* gene was overexpressed in the *rfg1/rfg1* mutant, and the suppression of filamentation at acidic pH was observed (Fig. 3A). These results indicate that the two transcription factors function in parallel during this process. Besides, chromatin immunoprecipitation (ChIP)-PCR assays demonstrated that Rfg1 directly binds to the *BCR1* promoter under acidic pH conditions, suggesting that Rfg1 interacts with Bcr1 to respond to the acidic pH condition and co-ordinate to regulate filamentation of *C. albicans* (Fig. 3B).

### Rfg1 inhibits the expression of pH-responsive genes, cell wall–related genes, and filament-regulating genes

**Fig 4:**
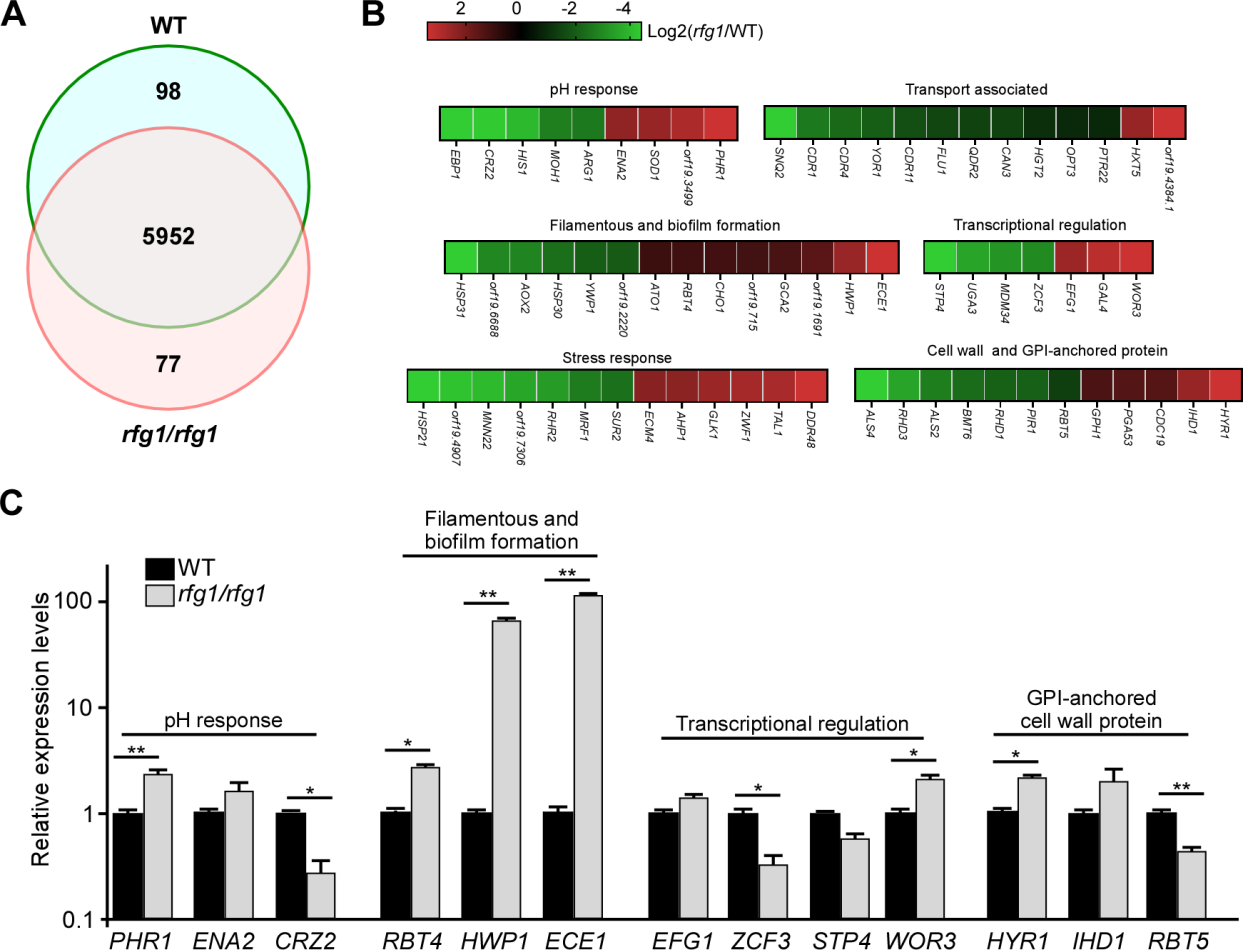
Global gene expression profile analysis in *rfg1/rfg1* mutant under acid pH condition. Cells were grown on YPD-K medium with pH 4 at 30°C for 2 days. Total RNA was extracted and used for RNA-Seq assays. A fold change value higher than or equal to 1.5, false discovery rates (FDRs) less than 0.05, and fragment per kilobase of transcript per million (FPKM) value higher than or equal to 20 at least in one sample were used to define differentially expressed genes. (A) Venn diagram depicting differentially expressed genes. (B) Functional categories of selected differentially expressed genes under acidic pH conditions. (C) Relative expression levels of Rfg1-related genes in the WT (SN250) and *rfg1/rfg1* strains on YPD-K medium (pH 4) at 30°C. Transcript levels of each gene in the WT were set as “1.” Data are presented as the mean ± SEM. *P < 0.05, **P < 0.01 (Student’s *t*-test, two-tailed).

The global gene expression profile analysis was performed to further investigate how Rfg1 responds to acidic pH and how it regulates filamentation in *C. albicans*. Total RNA was extracted from cells of the wild-type (WT) and *rfg1/rfg1* mutant grown on YPD-K (pH 4) at 30°C for 2 days. As shown in Fig. 4A, 77 genes were upregulated in *rfg1/rfg1* mutant, and 98 genes were upregulated in the WT strain (with a change >1.5 fold). Our key findings are summarized as follows: (i) A subset of pH response genes were differently expressed. Two alkaline pH-induced genes, namely, *PHR1* (encoding cell wall glycosidase) and *ENA2* (encoding sodium transporter), were upregulated in *the rfg1/rfg1* mutant under acidic pH conditions. *PHR1* and *ENA2* are regulated by Rim101 under alkaline pH (19, 40), suggesting that alkaline response signaling may be involved in Rfg1-mediated acidic pH response. (ii) As expected, several filamentation and biofilm-related genes were upregulated in the *rfg1/rfg1* mutant under acidic pH conditions, including *HWP1* (encoding hyphal cell wall protein), *ECE1* (encoding candidalysin), *GCA2* (encoding extracellular glucoamylase), and *RBT4* (encoding Pry family protein). (iii) The genes coding the cell wall and GPI-anchored proteins were differentially expressed in the *rfg1/rfg1* mutant. For example, *HYR1* and *IHD1* were upregulated in the *rfg1/rfg1* mutant, whereas *RBT5* and *PIR1* were upregulated in the WT strain. (iv) A subset of transcription factors that are involved in the regulation of morphogenesis was also differentially expressed. For example, *WOR3* (encoding white–opaque regulator) and *EFG1* (encoding filamentous growth regulator) are upregulated in the *rfg1/rfg1* mutant. Efg1 is a component of the cAMP pathway, which is essential for filamentation (41, 42). Taken together, the Rim101-Phr1 signaling pathway and cAMP-Efg1 pathway may play an important role in Rfg1-deletion-caused filamentation in response to acidic pH conditions.

### The Rim101-Phr1 pathway is involved in the regulation of Rfg1-deletion-caused filamentation

**Fig 5:**
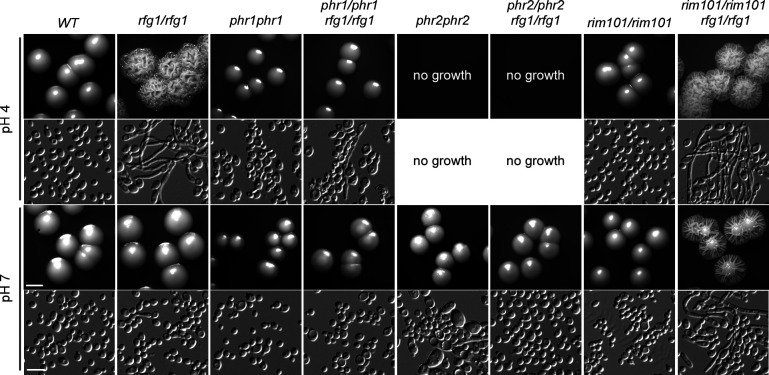
Role of the Rim101-Phr1-Phr2 pathway in Rfg1-regulated filamentation. Morphologies of WT (SN250), *rfg1/rfg1*, *phr1/phr1*, *phr1/phr1 rfg1/rfg1*, *phr2/phr2*, *phr2/phr2 rfg1/rfg1*, *rim101/rim101,* and *rim101/rim101 rfg1/rfg1* mutants. Cells were cultured on YPD-K media (pH 4, pH 7) at 30°C for 3 days. Scale bar for colonies, 2.5 mm; scale bar for cells, 10 µm.

The Rim101 signaling pathway governs pH response and is required for alkaline pH–induced filamentation in *C. albicans* (18, 19, 21). *PHR1* and *PHR2* are regulated by Rim101 under different pH conditions. *PHR1* is expressed at neutral–alkaline pH (pH >5.5), whereas *PHR2* is expressed at acidic pH (pH <5.5) (19, 27). Our RNA-sequencing (RNA-seq) analysis indicated that *PHR1* is upregulated in the *rfg1/rfg1* mutant under acidic pH conditions (Fig. 4B and C), suggesting that *PHR1* may play a role in Rfg1-mediated acidic pH response. To test this, we constructed *phr1/phr1 rfg1/rfg1*, *phr2/phr2 rfg1/rfg1,* and *rim101/rim101 rfg1/rfg1* double mutants and investigated their filamentation ability under pH 4–7 conditions. As shown in Fig. 5, *phr1/phr1 rfg1/rfg1* mutant could not undergo filamentation at either pH 4 or 7 conditions, indicating that *PHR1* is an important regulator involved in Rfg1-induced filamentation. Consistent with the *phr2/phr2* mutant, the *phr2/phr2 rfg1/rfg1* mutant could not grow under acidic pH conditions (17). Interestingly, the filamentation ability in the *rim101/rim101 rfg1/rfg1* mutant was dramatically increased, suggesting that Rim101 is a negative regulator governing filamentation when Rfg1 function is compromised.

### cAMP signaling pathway and downstream regulators, Efg1, Flo8, and Hgc1, are required for Rfg1-deletion-caused filamentation

As we mentioned above, Efg1, a downstream transcription factor of the cAMP signaling pathway, is upregulated in the *rfg1/rfg1* mutant under acidic pH conditions (Fig. 4B and C). To investigate the role of this pathway in Rfg1-mediated acidic pH response, we deleted the key component of this pathway, adenylyl cyclase Cyr1, in either WT or *rfg1/rfg1* background. As shown in Fig. S3A, the filamentation ability of the *cyr1/cyr1 rfg1/rfg1* mutant dramatically reduced, suggesting the key role of the cAMP signaling pathway in regulating Rfg1-deletion-caused filamentation. We further detected the role of downstream regulators of the cAMP signaling pathway. Efg1 and Flo8, two important regulators function downstream of the cAMP pathway, play a key role in the regulation of filamentation of *C. albicans* (41 – 44). As shown in Fig. S3A, the deletion of *EFG1* or *FLO8* could completely block the filamentation of the *rfg1/rfg1* mutant under acidic pH conditions. Hgc1, a hypha-specific G1 cyclin-related protein regulated by Efg1 and Flo8, is required for *C. albicans* hyphal morphogenesis (45). Therefore, we generated the double-deletion mutant *rfg1/rfg1 hgc1/hgc1*. Compared with the *rfg1/rfg1* mutant, the *rfg1/rfg1 hgc1/hgc1* mutant exhibited reduced filamentation ability under acidic pH conditions. Consistently, qRT-PCR assays indicated that the expression levels of *EFG1, FLO8,* and *HGC1* were significantly increased in the *rfg1/rfg1* mutant on the YPD-K (pH 4) medium (Fig. S3B). These results suggest that the cAMP signaling pathway and the downstream regulators Efg1, Flo8, and Hgc1 are essential for the Rfg1-deletion-caused filamentation under acidic pH conditions.

### Disrupting *RFG1* in *C. albicans* confers enhanced commensal fitness

**Fig 6:**
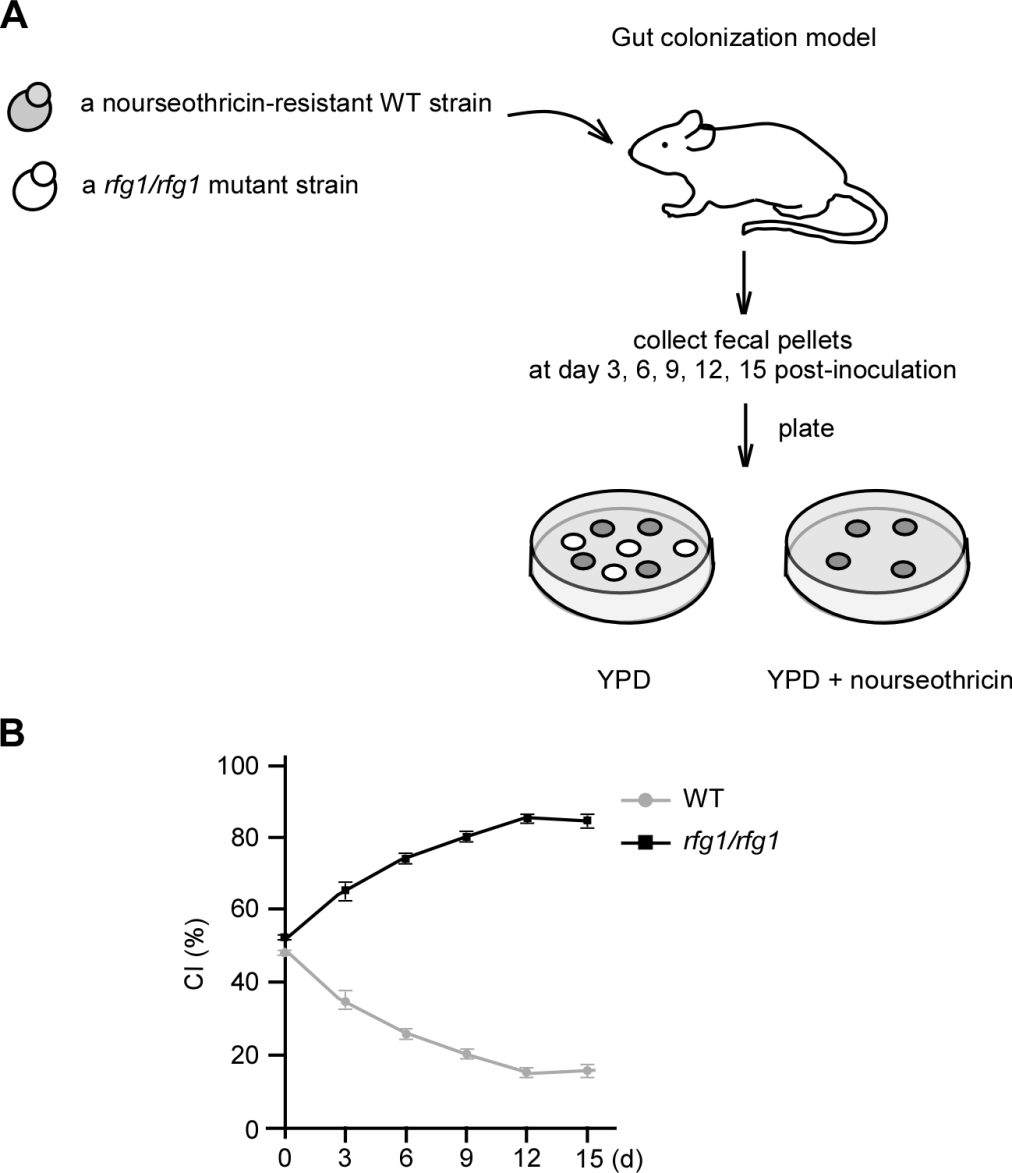
Deletion of *RFG1* results in enhanced commensal fitness. (A) Schematic of commensal competition experiments. Six- to eight-week-old BALB/c mice were orally inoculated with 1:1 mixtures of a nourseothricin-resistant WT strain (WT + pACTS) and an unmarked *rfg1/rfg1* mutant. The fecal pellets of each mouse were collected at days 3, 6, 9, 12, and 15 post-inoculation, and the relative abundance of strains was monitored by plating fecal homogenates on YPD plates supplemented with or without 200 µg/mL nourseothricin. The CI was defined as the proportion of the WT strain or *rfg1/rfg1* mutant to the total. (B) Competition between the WT strain and *rfg1/rfg1* mutant. *n* = 5. Data are presented as the mean ± SEM.

The filamentation ability of *C. albicans* is tightly related to its nutrient absorption, colonization, and infection in host niches. Given that disrupting *RFG1* allows filamentation of *C. albicans* under acidic pH conditions, we wonder whether it exerts influence on the gut commensal fitness of this pathogen. To test this, we performed a mouse commensal competition experiment in which mice were orally inoculated with 1:1 mixtures of a nourseothricin-resistant (NAT^r^) WT strain and *rfg1/rfg1* mutant (Fig. 6A). At various times post-inoculation, fresh fecal pellets were collected, resuspended, and plated on YPD plates with or without 200 µg/mL nourseothricin. The number of CFU per gram of sample (CFU/g) and the competitive index (CI, the proportion of the indicated strain to the total) were then determined. As expected, the *rfg1/rfg1* mutant exhibited enhanced commensal fitness in competition with WT (Fig. 6B). This result suggests that Rfg1-deletion-caused filamentation under acidic pH conditions is implicated in commensal growth of *C. albicans* in the host GI tract.

Overall, our data suggest a new mechanism involved in the regulation of filamentation of *C. albicans* in response to acidic pH conditions, in which Rfg1 plays a core role in responding to acidic pH conditions. Rfg1 directly binds to the *BCR1* promoter and regulates its transcriptional expression, which suppresses the filamentation of *C. albicans* under acidic pH conditions. During this process, the conserved Rim101-Phr1-mediated pathway and the cAMP signaling pathway play a positive role.

## DISCUSSION

**Fig 7:**
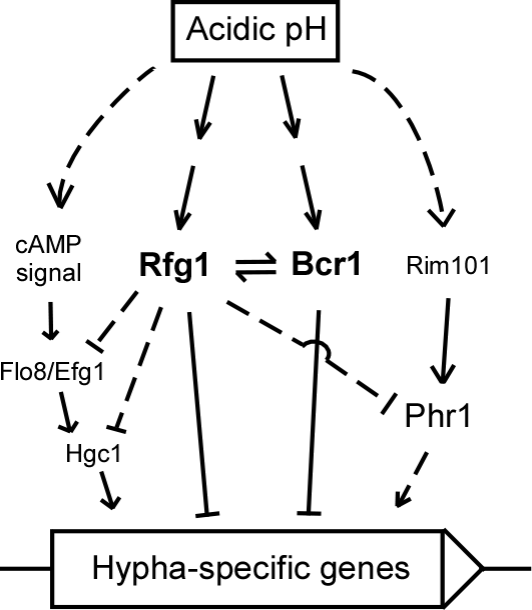
Model of Rfg1/Bcr1-regulated filamentation in response to acidic pH conditions in *C. albicans*. The Rim101-Phr1 pathway and cAMP signaling pathway play a positive role, while Bcr1 plays an inhibitory role in the Rfg1-suppressed filamentous growth under acidic pH conditions.

*C. albicans* can colonize and infect various host sites with diverse environmental pH ranging from acidic (pH 2–6, stomach, vagina, and oral cavity) to neutral–alkaline (pH 7–8, bloodstream, large intestine, and colon) (1 – 4). A neutral–alkaline pH can promote the filamentation and virulence of *C. albicans* through the conserved Rim101 pH sensing pathway (18, 21). In this study, we focused mostly on the acidic pH response of *C. albicans*. We identified a novel Rfg1-Bcr1 regulatory pathway that governs acidic pH response and filamentation in *C. albicans*. The Rim101-Phr1 pathway, cAMP signaling pathway, and a subset of downstream regulators, including Efg1, Flo8, and Hgc1, play critical roles in this regulation (Fig. 7).

The filamentation ability of *C. albicans* is tightly related to its virulence, which can enhance nutrient absorption, colonization, and infection in human niches. Many host-related environmental factors have been reported to promote filamentation, including physiological temperature, CO_2_, serum, and neutral–alkaline pH (14, 17). Acidic pH conditions, another environmental factor frequently encountered by *C. albicans* inside the human body, inhibit filamentation. Considering that filamentation is a key virulence feature of *C. albicans*, how can this pathogen survive and cause infection under acidic pH conditions? We propose that acidic pH conditions caused by lactic acid bacteria can promote *C. albicans* opaque cell filamentation through the co-regulation of cAMP signaling and Rfg1-mediated pathways (46). However, as we know, unlike the default white cell type, opaque cells were observed only in a minority (< 10%) of natural *C. albicans* isolates (47). Therefore, exploring the biological behavior of default white cells in response to acidic pH conditions is necessary. In this study, *C. albicans* white cells undergo robust filamentation under acidic pH conditions when the expression level of Rfg1 is reduced (Fig. 1 and 2). This event was also observed in some clinical isolates (Fig. S1), which indicates that Rfg1-deletion-caused filamentation under acidic pH conditions may be a general feature of natural *C. albicans* strains. Besides body site pH, physiological temperature is another environmental factor that is frequently encountered by *C. albicans* inside the human body. High temperatures stimulate yeast to filament transition, whereas low temperature inhibits this process (17). Restrained Rfg1 expression resulted in the filamentation of *C. albicans* under acidic pH conditions even at low temperatures (<30°C) (Fig. S2). Considering that the physiological temperatures of some human niches, including the skin, nails, and external region of the vagina, can be lower than 30°C, Rfg1-deletion-caused filamentation thereby may be considered clinically significant in specific body sites.

Transcription factor Rfg1, a homolog of *Saccharomyces cerevisiae* hypoxic regulator Rox1, functions as a condition-specific transcription regulator of the cell filamentation of *C. albicans* (35). Kadosh and Johnson (35) reported that the deletion of *RFG1* results in robust filamentation when *C. albicans* cells are cultured at 37°C or in a serum medium. Rfg1 repression activity is relieved by filament-inducing conditions, such as 37°C and serum. They also propose that Rfg1 regulates filamentation in a nutrient-dependent manner. Besides these environmental factors, pH is an important host stress frequently encountered by *C. albicans* in the mammalian body. However, no studies have investigated the regulatory role of Rfg1 in response to body sites with different pH. In this study, by using an exogenous pH buffering system (YPD + K_2_HPO_4_), we constructed diverse pH-stabilizing conditions, including pH 4, 5, 6, and 7. The results showed that *RFG1* deletion resulted in robust filamentation under acidic pH (pH 4–6) but not under neutral pH (pH 7) conditions (Fig. 2). Our findings indicate that Rfg1 is a repressing regulator in response to acidic pH conditions in the mammalian host. This regulatory mechanism is similar to our previous finding that the Rfg1 transcription factor plays a repressing role in lactic acid–induced opaque cell filamentation, suggesting a conserved role of Rfg1 in regulating filamentation of *C. albicans* in response to acidic pH conditions.

Besides Rfg1, Bcr1, Rbt5, Sld1, Cfl11, and orf19.1092 play important roles in filamentation under acidic pH conditions (Fig. 1). qRT-PCR assays indicated that the expression levels of *BCR1* and *RBT5* were significantly reduced in the *rfg1/rfg1* mutant on the YPD-K medium (pH 4), suggesting a potential role of Rfg1 in regulating *BCR1* and *RBT5* under acidic pH conditions. Previous studies have demonstrated that Bcr1, a transcription factor governing biofilm formation, is also required for opaque cell filamentation (37, 38). Rbt5 is a GPI-modified cell wall protein, which has previously been demonstrated to be regulated by Rfg1 (35). Our ChIP assay further indicated that Rfg1 directly binds to the promoter of *BCR1* and regulates its transcription under acidic pH conditions. Bcr1 binds to the promoters of *RFG1* and *RBT5* to regulate biofilm formation (48). These findings imply that Rfg1, together with Bcr1 and Rbt5, coregulate the filamentation of *C. albicans* in response to acidic pH conditions. The dysfunction of Sld1, Cfl11, and orf19.1092 also resulted in obvious filamentation under acidic pH conditions (Fig. 1). However, no interaction relationship was detected through qRT-PCR assays, which indicates that the inhibitory role of those three proteins in filamentation is independent of Rfg1 function.

The Rim101 pathway governs pH response and is required for alkaline pH–induced filamentation in *C. albicans*. The expression of *RIM101* is increased at alkaline pH, which is required for pH-regulated filamentation in *C. albicans* (18, 21). Phr1 and Phr2 are regulated by Rim101 under different pH conditions. Phr1 is expressed at neutral–alkaline pH (pH >5.5), whereas Phr2 is expressed at acidic pH (pH <5.5) (19, 27). In this study, the filamentation ability in the *rim101/rim101 rfg1/rfg1* mutant was dramatically increased, suggesting that Rim101 is a negative regulator of filamentation when Rfg1 function is compromised. However, neither RNA-seq analysis nor qRT-PCR assay detects the distinct transcriptional expression of Rim101 in the *rfg1/rfg1* mutant. Considering that at neutral–alkaline pH, Rim101 activation is aimed by removing a C-terminal 8 kDa peptide, we assume that the function of Rim101 may be regulated through a post-translational modification, but not through transcriptional regulation. Moreover, *PHR1* is upregulated in the *rfg1/rfg1* mutant under acidic pH conditions (Fig. 4B and C), and *PHR1* deletion in the *rfg1/rfg1* mutant caused filamentation defect under acidic pH conditions, suggesting that neutral–alkaline pH–specific regulator Phr1 plays an important role in Rfg1-mediated acidic pH response. Briefly, *RFG1* deletion may result in a neutral–alkaline pH environment, which promotes filamentation through the Rim101-Phr1 pathway.

The ability to adjust extracellular pH is associated with the virulence of pathogenic fungi (49). Several fungal species such as *Colletotrichum spp*. (50), *Alternaria alternata* (51)*,* and *Fusarium oxysporum* (52) could make the host environment alkaline by releasing volatile ammonia during their infection of plant roots and fruits. *C. albicans* can also regulate the pH of macrophage cells (53, 54). Once swallowed by macrophages, *C. albicans* uses the amino acids in the phagosome as a carbon source. Ammonia released by amino acid metabolism neutralizes the acidic environment in macrophages and promotes filamentation of *C. albicans*. We hypothesize that Rfg1-mediated acidic pH response may correlate with the release of ammonia, which thereby results in the rise of ambient pH and promotes filamentation.

In conclusion, our results reveal that Rfg1 is an essential acidic pH regulator in *C. albicans*. Rfg1 regulates filamentation under acidic pH conditions via the co-regulation of the Rim101-Phr1 pathway, cAMP signaling pathway, and regulators Bcr1, Efg1, Flo8, and Hgc1. These results provide new insights into the acidic pH response mechanism in *C. albicans*.

## MATERIALS AND METHODS

### Strains and media

The strains used in this study are listed in Table S1. YPD (20 g/L peptone, 10 g/L yeast extract, and 20 g/L glucose) was used for the routine growth of *C. albicans*. HCl was used to adjust the medium to the desired pH. 0.25% K_2_HPO_4_ (P9666, Sigma-Aldrich, St. Louis, MO, USA) was added to the YPD media for pH buffering. Peptone, yeast extract, and agar were purchased from BD Biosciences (211677, 212750, 214101; BD Biosciences, Sparks, MD, USA). Glucose was purchased from Sigma-Aldrich (G8270, Sigma-Aldrich, St. Louis, MO, USA).

### Plasmid and strain construction

To construct the pACTS*-RFG1* and pACTS*-BCR1* overexpression plasmids, the *RFG1* or *BCR1* open reading frames were amplified by PCR from SC5314 genomic DNA and subcloned into the *Eco*RV*/Hind*III site of the pACTS plasmid, respectively (39, 55). All the primers used in the study are listed in Table S2.

To construct the knockout plasmids pSFS2a-*RFG1* KO, pSFS2a-*FLO8* KO, pSFS2a-*EFG1* KO, and pSFS2a-*HGC1* KO, two flanking fragments of *RFG1*, *FLO8*, *EFG1*, or *HGC1* were inserted into the *Apa*I*/Xho*I and *Sac*II*/Sac*I sites of the plasmid pSFS2A (56).

To generate the reconstituted plasmid pSFS2a-*RFG1p-RFG1*, two fragments containing sequences of partial 3′UTR region and 5′UTR-ORF region of *RFG1* were sequentially inserted into the *Sac*II*/Sac*I and *Apa*I*/Xho*I sites of the plasmid pSFS2a (56).

To knock out the *RFG1* gene in the *phr1/phr1* mutant, the first copy of *RFG1* was deleted by transforming the *Apa*I/*Sac*I-linearized pSFS2a-*RFG1* KO plasmid. The result strain *phr1/phr1 RFG1/rfg1::SAT1-FLP* was then grown in YPmal medium (20 g/L peptone, 10 g/L yeast extract, and 20 g/L maltose) for FLP-mediated excision of the *SAT1*/flipper cassette, generating the strain *phr1/phr1 RFG/rfg1::FRT*. To delete the second copy of *RFG1*, the strain *phr1/phr1 RFG1/rfg1::FRT* was once again transformed with the *Apa*I*/Sac*I-linearized pSFS2a-*RFG1* KO plasmid. A similar strategy was used to knock out *RFG1* in the *phr2/phr2*, *rim101/rim101*, and *cyr1/cyr1* mutants, generating the *phr2/phr2 rfg1/rfg1*, *rim101/rim101 rfg1/rfg1*, and *cyr1/cyr1 rfg1/rfg1* double mutants.

Similar to above, two alleles of *EFG1* were subsequently disrupted with the *Apa*I*/Sac*I-linearized pSFS2a-EFG1 KO plasmid, generating the *rfg1/rfg1/efg1/efg1* double mutant.

To construct the *rfg1/rfg1/flo8/flo8* double mutant, a fragment of *FLO8* flanking *ARG4* selectable marker (57) was amplified from pRS-*ARG4ΔSpe*I plasmid with the primers FLO8-5DR/ FLO8-3DR and was transformed into the *rfg1/rfg1* mutant to delete the first copy of *FOL8*. A fragment of the *Apa*I*/Sac*I-linearized pSFS2a-*FLO8* KO plasmid was used to delete the second copy of *FLO8*, generating the *rfg1/rfg1/flo8/flo8* double mutant. A similar strategy was used to knock out *HGC1* in the *rfg1/rfg1* mutant, generating the *rfg1/rfg1 hgc1/hgc1* double mutant.

To construct a TAP-tagged Rfg1 expression strain, the *TAP-ARG4* cassette flanked by 60 bp 5′ and 3′ homologous sequences of *RFG1* was amplified from the strain CaLC2993 (58, 59) using primers *RFG1-TAP* 5DR and *RFG1-TAP* 3 DR. The cassette was then transformed into the *RFG1/rfg1* strain.

### RNA-seq and quantitative real-time PCR (qRT-PCR) assay

Cells of the WT and *rfg1/rfg1*strains were cultured on YPD-K medium at 30°C for 2 days and then plated onto solid YPD-K media (pH 4 and pH 7) and cultured at 30°C for 2 days. Cells were collected, and total RNA was extracted for RNA-seq analysis. Two biological repeats were performed for each condition. RNA-seq analysis was performed by BGI-Shenzhen according to the company’s protocol (http://www.genomics.cn/). Approximately 3 Gb (gigabases) of reads was obtained by sequencing each library. The library products were sequenced using the Illumina HiSeq 2000. Illumina OLB_1.9.4 software was used for base calling. The raw reads were filtered by removing the adapter and low-quality reads. Clean reads were mapped to the genome of *C. albicans* SC5314 using SOAP aligner/soap2 software (version 2.21) (60). Differentially expressed genes must satisfy two criteria: (i) fold change of ≥1.5 and (ii) FDRs of ≤0.05. The detailed RNA-seq data set has been deposited into the NCBI Gene Expression Omnibus (GEO) portal (GSE227654). The heat maps of selected differential genes were made using GraphPad Prism 9 software.

qRT-PCR assays were performed as previously described (61). One microgram of total RNA per sample was used to synthesize cDNA with Revert Aid H Minus reverse transcriptase (EP0451, Thermo Scientific, Waltham, USA). Quantification of transcripts was performed in the Bio-Rad CFX96 real-time PCR detection system (Bio-Rad, Hercules, USA) using SYBR green master mix (QPS-201, TOYOBO, Osaka, Japan). The signal of each sample was normalized to the expression level of the *ACT1* gene.

### Chromatin IP

Chromatin IP assays were performed according to previous publications with slight modifications (55, 59). Briefly, untagged and *RFG1-TAP* tagged stains were cultured on solid YPD-K media (pH 4) at 30°C for 2 days. Cells were collected and fixed in phosphate-buffered saline (PBS) containing 1% fresh formaldehyde and crosslinked for 20 min on a platform shaker at room temperature. The crosslinking reaction was quenched by adding 2.5 M glycine to a final concentration of 125 mM, and cells were incubated for 5 min at room temperature on a platform shaker. Cells were collected and washed with ice-cold PBS and homogenized in ice-cold lysis buffer using a bead beater. The lysate chromatin was sheared by sonication in a Diagenode Bioruptor (Diagenode, Denville, NJ, USA) for 12 min (high setting, 30 s on, and 1 min off) to obtain fragments of an average size of 250–1,000 bp. The shearing lysate was immunoprecipitated with 50 µL packed IgG Sepharose 6 Fast Flow matrix (GE Healthcare, Chicago, IL, USA). The Sepharose beads were washed, and then, the immunoprecipitated DNA was recovered, de-crosslinked, and purified. qRT-PCR assays were performed to determine Rfg1 targets.

### Commensal competition experiments in mouse

All animal experiments were performed according to the guidelines approved by the Animal Care and Use Committee of Fudan University. Four 6-week-old female BALB/c mice weighing 18–20 g were treated with antibiotics water (streptomycin, 2 mg/mL; penicillin, 0.97 mg/mL) for 3 days and then orally inoculated with 1:1 mixtures of a nourseothricin-resistant strain (WT + pACTS) and an unmarked *rfg1/rfg1* mutant at 5 × 10^8^ cells/mL as previously reported (62). Fresh fecal pellets collected at days 3, 6, 9, 12, and 15 post-inoculation were homogenized and plated on YPD plates containing streptomycin (100 µg/mL) and ampicillin (50 µg/mL) supplemented with or without 200 µg/mL nourseothricin. The CI was defined as the proportion of the WT strain or *rfg1/rfg1* mutant to the total.

## Data Availability

The RNA-seq data have been deposited in the NCBI Gene Expression Omnibus repository under accession number GSE227654.

## SUPPLEMENTAL MATERIAL

Dataset S1 spectrum.01789-23-s0001.pdf RNA-Seq data.

Supplemental figures and tables spectrum.01789-23-s0002.pdf Fig. S1 to S3; Table S1 and S2.

ASM does not own the copyrights to Supplemental Material that may be linked to, or accessed through, an article. The authors have granted ASM a non-exclusive, world-wide license to publish the Supplemental Material files. Please contact the corresponding author directly for reuse.

## References

[1] Limon JJ , Skalski JH , Underhill DM . 2017. Commensal fungi in health and disease. Cell Host Microbe 22:156–165. doi:10.1016/j.chom.2017.07.002 28799901 PMC5573128

[2] Kumamoto CA . 2011. Inflammation and gastrointestinal Candida colonization. Curr Opin Microbiol 14:386–391. doi:10.1016/j.mib.2011.07.015 21802979 PMC3163673

[3] Cauchie M , Desmet S , Lagrou K . 2017. Candida and its dual lifestyle as a commensal and a pathogen. Res Microbiol 168:802–810. doi:10.1016/j.resmic.2017.02.005 28263903

[4] Alves R , Barata-Antunes C , Casal M , Brown AJP , Van Dijck P , Paiva S , Filler SG . 2020. Adapting to survive: how Candida overcomes host-imposed constraints during human colonization. PLoS Pathog 16:e1008478. doi:10.1371/journal.ppat.1008478 32437438 PMC7241708

[5] Zwolińska-Wcisło M , Budak A , Trojanowska D , Bogdał J , Stachura J . 1998. Fungal colonization of the stomach and its clinical relevance. Mycoses 41:327–334. doi:10.1111/j.1439-0507.1998.tb00346.x 9861839

[6] Gunther LSA , Martins HPR , Gimenes F , Abreu A de , Consolaro MEL , Svidzinski TIE . 2014. Prevalence of Candida albicans and non-albicans isolates from vaginal secretions: comparative evaluation of colonization, vaginal candidiasis and recurrent vaginal candidiasis in diabetic and non-diabetic women. Sao Paulo Med J 132:116–120. doi:10.1590/1516-3180.2014.1322640 24714993 PMC10896579

[7] Cannon RD , Chaffin WL . 1999. Oral colonization by Candida albicans. Crit Rev Oral Biol Med 10:359–383. doi:10.1177/10454411990100030701 10759414

[8] De Bernardis F , Mühlschlegel FA , Cassone A , Fonzi WA . 1998. The pH of the host niche controls gene expression in and virulence of Candida albicans. Infect Immun 66:3317–3325. doi:10.1128/IAI.66.7.3317-3325.1998 9632601 PMC108348

[9] Brown GD , Denning DW , Gow NAR , Levitz SM , Netea MG , White TC . 2012. Hidden killers: human fungal infections. Sci Transl Med 4:165rv13. doi:10.1126/scitranslmed.3004404 23253612

[10] Berman J . 2012. Candida albicans. Curr Biol 22:R620–R622. doi:10.1016/j.cub.2012.05.043 22917504

[11] Huang G . 2012. Regulation of phenotypic transitions in the fungal pathogen Candida albicans. Virulence 3:251–261. doi:10.4161/viru.20010 22546903 PMC3442837

[12] Sudbery PE . 2011. Growth of Candida albicans hyphae. Nat Rev Microbiol 9:737–748. doi:10.1038/nrmicro2636 21844880

[13] Kadosh D . 2019. Regulatory mechanisms controlling morphology and pathogenesis in Candida albicans. Curr Opin Microbiol 52:27–34. doi:10.1016/j.mib.2019.04.005 31129557 PMC6874724

[14] Whiteway M , Bachewich C . 2007. Morphogenesis in Candida albicans. Annu Rev Microbiol 61:529–553. doi:10.1146/annurev.micro.61.080706.093341 17506678 PMC4452225

[15] Sudbery P , Gow N , Berman J . 2004. The distinct morphogenic states of Candida albicans. Trends Microbiol 12:317–324. doi:10.1016/j.tim.2004.05.008 15223059

[16] Koh AY , Köhler JR , Coggshall KT , Van Rooijen N , Pier GB , Filler SG . 2008. Mucosal damage and neutropenia are required for Candida albicans dissemination. PLoS Pathog 4:e35. doi:10.1371/journal.ppat.0040035 18282097 PMC2242836

[17] Biswas S , Van Dijck P , Datta A . 2007. Environmental sensing and signal transduction pathways regulating morphopathogenic determinants of Candida albicans. Microbiol Mol Biol Rev 71:348–376. doi:10.1128/MMBR.00009-06 17554048 PMC1899878

[18] Davis DA . 2009. How human pathogenic fungi sense and adapt to pH: the link to virulence. Curr Opin Microbiol 12:365–370. doi:10.1016/j.mib.2009.05.006 19632143

[19] Davis D , Wilson RB , Mitchell AP . 2000. RIM101-dependent and-independent pathways govern pH responses in Candida albicans. Mol Cell Biol 20:971–978. doi:10.1128/MCB.20.3.971-978.2000 10629054 PMC85214

[20] Cornet M , Gaillardin C . 2014. pH signaling in human fungal pathogens: a new target for antifungal strategies. Eukaryot Cell 13:342–352. doi:10.1128/EC.00313-13 24442891 PMC3957587

[21] Davis D , Edwards JE , Mitchell AP , Ibrahim AS . 2000. Candida albicans RIM101 pH response pathway is required for host–pathogen interactions. Infect Immun 68:5953–5959. doi:10.1128/IAI.68.10.5953-5959.2000 10992507 PMC101559

[22] Xu W , Smith FJ , Subaran R , Mitchell AP . 2004. Multivesicular body-ESCRT components function in pH response regulation in Saccharomyces cerevisiae and Candida albicans. Mol Biol Cell 15:5528–5537. doi:10.1091/mbc.e04-08-0666 15371534 PMC532031

[23] Wolf JM , Johnson DJ , Chmielewski D , Davis DA . 2010. The Candida albicans ESCRT pathway makes Rim101-dependent and -independent contributions to pathogenesis. Eukaryot Cell 9:1203–1215. doi:10.1128/EC.00056-10 20581294 PMC2918940

[24] Li M , Martin SJ , Bruno VM , Mitchell AP , Davis DA . 2004. Candida albicans Rim13p, a protease required for Rim101p processing at acidic and alkaline pHs. Eukaryot Cell 3:741–751. doi:10.1128/EC.3.3.741-751.2004 15189995 PMC420141

[25] Kullas AL , Martin SJ , Davis D . 2007. Adaptation to environmental pH: integrating the Rim101 and calcineurin signal transduction pathways. Mol Microbiol 66:858–871. doi:10.1111/j.1365-2958.2007.05929.x 17927701

[26] Du H , Huang G . 2016. Environmental pH adaption and morphological transitions in Candida albicans. Curr Genet 62:283–286. doi:10.1007/s00294-015-0540-8 26581628

[27] Fonzi WA . 1999. PHR1 and PHR2 of Candida albicans encode putative glycosidases required for proper cross-linking of beta-1,3-and beta-1,6-glucans. J Bacteriol 181:7070–7079. doi:10.1128/JB.181.22.7070-7079.1999 10559174 PMC94183

[28] Calderon J , Zavrel M , Ragni E , Fonzi WA , Rupp S , Popolo L . 2010. PHR1, a pH-regulated gene of Candida albicans encoding a glucan-remodelling enzyme, is required for adhesion and invasion. Microbiology (Reading) 156:2484–2494. doi:10.1099/mic.0.038000-0 20430812

[29] Mühlschlegel FA , Fonzi WA . 1997. PHR2 of Candida albicans encodes a functional homolog of the pH-Regulated gene PHR1 with an inverted pattern of pH-dependent expression. Mol Cell Biol 17:5960–5967. doi:10.1128/MCB.17.10.5960 9315654 PMC232444

[30] Cotter PD , Emerson N , Gahan CG , Hill C . 1999. Identification and disruption of lisRK, a genetic locus encoding a two-component signal transduction system involved in stress tolerance and virulence in Listeria monocytogenes. J Bacteriol 181:6840–6843. doi:10.1128/JB.181.21.6840-6843.1999 10542190 PMC94153

[31] Merrell DS , Hava DL , Camilli A . 2002. Identification of novel factors involved in colonization and acid tolerance of Vibrio cholerae. Mol Microbiol 43:1471–1491. doi:10.1046/j.1365-2958.2002.02857.x 11952899

[32] Wilmes-Riesenberg MR , Foster JW , Curtiss R . 1997. An altered rpoS allele contributes to the avirulence of Salmonella typhimurium LT2. Infect Immun 65:203–210. doi:10.1128/iai.65.1.203-210.1997 8975913 PMC174577

[33] Foster JW , Moreno M . 1999. Inducible acid tolerance mechanisms in enteric bacteria. Novartis Found Symp 221:55–69. doi:10.1002/9780470515631.ch5 10207913

[34] Noble SM , Johnson AD . 2005. Strains and strategies for large-scale gene deletion studies of the diploid human fungal pathogen Candida albicans. Eukaryot Cell 4:298–309. doi:10.1128/EC.4.2.298-309.2005 15701792 PMC549318

[35] Kadosh D , Johnson AD . 2001. Rfg1, a protein related to the Saccharomyces cerevisiae hypoxic regulator Rox1, controls filamentous growth and virulence in Candida albicans. Mol Cell Biol 21:2496–2505. doi:10.1128/MCB.21.7.2496-2505.2001 11259598 PMC86882

[36] Sidebotham RL , Baron JH . 1990. Hypothesis: Helicobacter pylori, urease, mucus, and gastric ulcer. Lancet 335:193–195. doi:10.1016/0140-6736(90)90279-e 1967668

[37] Nobile CJ , Mitchell AP . 2005. Regulation of cell-surface genes and biofilm formation by the C. albicans transcription factor Bcr1p. Curr Biol 15:1150–1155. doi:10.1016/j.cub.2005.05.047 15964282

[38] Guan GB , Xie J , Tao L , Nobile CJ , Sun Y , Cao C , Tong Y , Huang G . 2013. Bcr1 plays a central role in the regulation of opaque cell filamentation in Candida albicans. Mol Microbiol 89:732–750. doi:10.1111/mmi.12310 23808664 PMC3758918

[39] Huang GH , Wang HF , Chou S , Nie XY , Chen JY , Liu H . 2006. Bistable expression of WOR1, a master regulator of white-opaque switching in Candida albicans. Proc Natl Acad Sci U S A 103:12813–12818. doi:10.1073/pnas.0605270103 16905649 PMC1540355

[40] Bensen ES , Martin SJ , Li M , Berman J , Davis DA . 2004. Transcriptional profiling in Candida albicans reveals new adaptive responses to extracellular pH and functions for Rim101p. Mol Microbiol 54:1335–1351. doi:10.1111/j.1365-2958.2004.04350.x 15554973

[41] Stoldt VR , Sonneborn A , Leuker CE , Ernst JF . 1997. Efg1p, an essential regulator of morphogenesis of the human pathogen Candida albicans, is a member of a conserved class of bHLH proteins regulating morphogenetic processes in fungi. EMBO J 16:1982–1991. doi:10.1093/emboj/16.8.1982 9155024 PMC1169801

[42] Bockmühl DP , Ernst JF . 2001. A potential phosphorylation site for an A-type kinase in the Efg1 regulator protein contributes to hyphal morphogenesis of Candida albicans. Genetics 157:1523–1530. doi:10.1093/genetics/157.4.1523 11290709 PMC1461612

[43] Cao F , Lane S , Raniga PP , Lu Y , Zhou Z , Ramon K , Chen J , Liu H . 2006. The Flo8 transcription factor is essential for hyphal development and virulence in Candida albicans. Mol Biol Cell 17:295–307. doi:10.1091/mbc.e05-06-0502 16267276 PMC1345667

[44] Du H , Guan G , Xie J , Cottier F , Sun Y , Jia W , Mühlschlegel FA , Huang G . 2012. The transcription factor Flo8 mediates CO_2_ sensing in the human fungal pathogen Candida albicans. Mol Biol Cell 23:2692–2701. doi:10.1091/mbc.E12-02-0094 22621896 PMC3395658

[45] Zheng X , Wang Y , Wang Y . 2004. Hgc1, a novel hypha-specific G1 cyclin-related protein regulates Candida albicans hyphal morphogenesis. EMBO J 23:1845–1856. doi:10.1038/sj.emboj.7600195 15071502 PMC394249

[46] Liang W , Guan G , Dai Y , Cao C , Tao L , Du H , Nobile CJ , Zhong J , Huang G . 2016. Lactic acid bacteria differentially regulate filamentation in two heritable cell types of the human fungal pathogen Candida albicans. Mol Microbiol 102:506–519. doi:10.1111/mmi.13475 27479705 PMC5074855

[47] Lockhart SR , Pujol C , Daniels KJ , Miller MG , Johnson AD , Pfaller MA , Soll DR . 2002. In Candida albicans, white-opaque switchers are homozygous for mating type. Genetics 162:737–745. doi:10.1093/genetics/162.2.737 12399384 PMC1462282

[48] Nobile CJ , Fox EP , Nett JE , Sorrells TR , Mitrovich QM , Hernday AD , Tuch BB , Andes DR , Johnson AD . 2012. A recently evolved transcriptional network controls biofilm development in Candida albicans. Cell 148:126–138. doi:10.1016/j.cell.2011.10.048 22265407 PMC3266547

[49] Selvig K , Alspaugh JA . 2011. pH response pathways in fungi: adapting to host-derived and evironmental signals. Mycobiology 39:249–256. doi:10.5941/MYCO.2011.39.4.249 22783112 PMC3385132

[50] You B-J , Choquer M , Chung K-R . 2007. The Colletotrichum acutatum gene encoding a putative pH-responsive transcription regulator is a key virulence determinant during fungal pathogenesis on citrus. Mol Plant Microbe Interact 20:1149–1160. doi:10.1094/MPMI-20-9-1149 17849717

[51] Eshel D , Miyara I , Ailing T , Dinoor A , Prusky D . 2002. pH regulates endoglucanase expression and virulence of Alternaria alternata in persimmon fruit. Mol Plant Microbe Interact 15:774–779. doi:10.1094/MPMI.2002.15.8.774 12182334

[52] Caracuel Z , Roncero MIG , Espeso EA , González-Verdejo CI , García-Maceira FI , Di Pietro A . 2003. The pH signalling transcription factor PacC controls virulence in the plant pathogen Fusarium oxysporum. Mol Microbiol 48:765–779. doi:10.1046/j.1365-2958.2003.03465.x 12694620

[53] Vylkova S , Carman AJ , Danhof HA , Collette JR , Zhou H , Lorenz MC , Taylor JW . 2011. The fungal pathogen Candida albicans autoinduces hyphal morphogenesis by raising extracellular pH. mBio 2:e00055-11. doi:10.1128/mBio.00055-11 21586647 PMC3101780

[54] Vylkova S , Lorenz MC . 2017. Phagosomal neutralization by the fungal pathogen Candida albicans induces macrophage pyroptosis. Infect Immun 85:e00832-16. doi:10.1128/IAI.00832-16 27872238 PMC5278172

[55] Guan G , Tao L , Yue H , Liang W , Gong J , Bing J , Zheng Q , Veri AO , Fan S , Robbins N , Cowen LE , Huang G . 2019. Environment-induced same-sex mating in the yeast Candida albicans through the Hsf1-Hsp90 pathway. PLoS Biol 17:e2006966. doi:10.1371/journal.pbio.2006966 30865631 PMC6415874

[56] Reuss O , Vik A , Kolter R , Morschhäuser J . 2004. The SAT1 flipper, an optimized tool for gene disruption in Candida albicans. Gene 341:119–127. doi:10.1016/j.gene.2004.06.021 15474295

[57] Wilson RB , Davis D , Mitchell AP . 1999. Rapid hypothesis testing with Candida albicans through gene disruption with short homology regions. J Bacteriol 181:1868–1874. doi:10.1128/JB.181.6.1868-1874.1999 10074081 PMC93587

[58] Leach MD , Budge S , Walker L , Munro C , Cowen LE , Brown AJP . 2012. Hsp90 orchestrates transcriptional regulation by Hsf1 and cell wall remodelling by MAPK signalling during thermal adaptation in a pathogenic yeast. PLoS Pathog 8:e1003069. doi:10.1371/journal.ppat.1003069 23300438 PMC3531498

[59] Leach MD , Farrer RA , Tan K , Miao Z , Walker LA , Cuomo CA , Wheeler RT , Brown AJP , Wong KH , Cowen LE . 2016. Hsf1 and Hsp90 orchestrate temperature-dependent global transcriptional remodelling and chromatin architecture in Candida albicans. Nat Commun 7:11704. doi:10.1038/ncomms11704 27226156 PMC4894976

[60] Li R , Yu C , Li Y , Lam T-W , Yiu S-M , Kristiansen K , Wang J . 2009. SOAP2: an improved ultrafast tool for short read alignment. Bioinformatics 25:1966–1967. doi:10.1093/bioinformatics/btp336 19497933

[61] Tao L , Zhang Y , Fan S , Nobile CJ , Guan G , Huang G , Butler G . 2017. Integration of the tricarboxylic acid (TCA) cycle with cAMP signaling and Sfl2 pathways in the regulation of CO2 sensing and hyphal development in Candida albicans. PLoS Genet 13:e1006949. doi:10.1371/journal.pgen.1006949 28787458 PMC5567665

[62] Pierce JV , Kumamoto CA . 2012. Variation in Candida albicans EFG1 expression enables host-dependent changes in colonizing fungal populations. mBio 3:e00117–12. doi:10.1128/mBio.00117-12 22829676 PMC3413400

