# Supplementary material for: The Rfg1 and Bcr1 transcription factors regulate acidic pH–induced filamentous growth in Candida albicans
Source: Microbiol Spectr. 2023 Nov 7;11(6):e01789-23. doi: 10.1128/spectrum.01789-23 (PMC10715123; doi:10.1128/spectrum.01789-23)
Supplement: Supplemental figures and tables — Fig. S1 to S3; Table S1 and S2. [file spectrum.01789-23-s0002.pdf]

SUPPLEMENTAL METERIAL

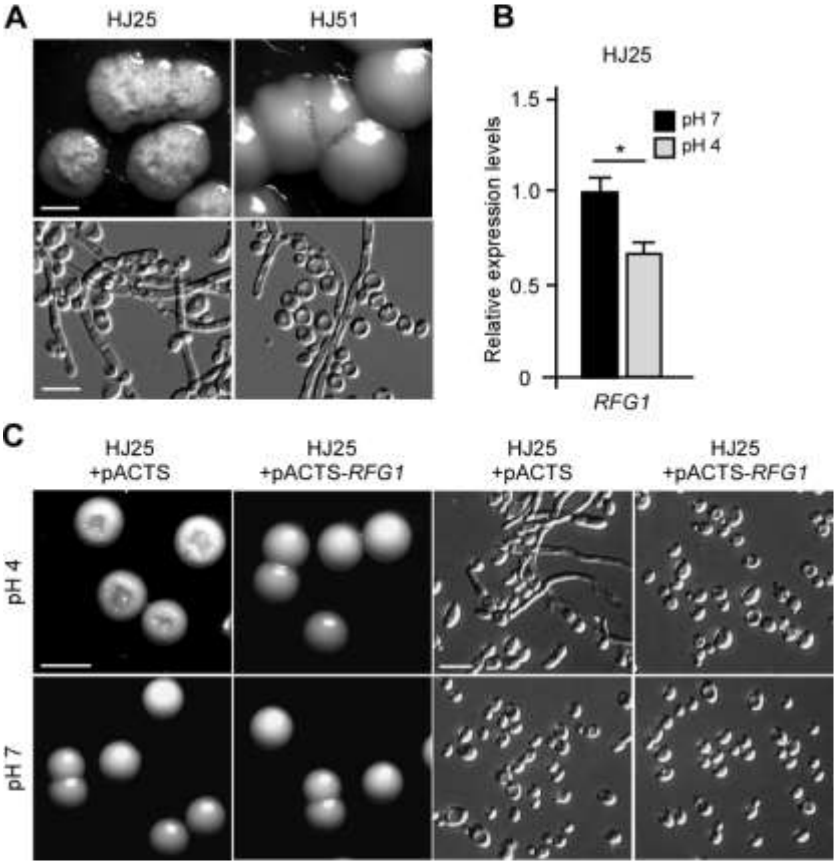

Fig. S1 Role of Rfg1 in acidic pH induced filamentation in clinical isolate HJ25

A. Morphologies of clinical isolates in response to acidic pH. Colony and cellular images of the clinical isolates (HJ25 and HJ51) under acidic pH conditions. Cells were grown on YPD-Kmedia (pH 4) at 30˚C for 5 days. Scale bar for colonies, 2.5 mm; scale bar for cells, 10 μm. B. Relative expression levels of *RFG1* in the HJ25 strain on YPD-K medium (pH 4 and 7) at 30˚C. Transcript level of *RFG1* under the pH 7 condition were set as ‘1’. Data are presented as the mean ± SEM. *P < 0.05 (Student’s *t*-test, two-tailed). C. Morphologies of the control (HJ25 + pACTS) and *RFG1*-overexpressing strains (HJ25 + pACTS-*RFG1*) on YPD-K medium (pH 4, pH 7) at 30˚C for two days.

Scale bar for colonies, 2.5 mm; scale bar for cells, 10 μm.

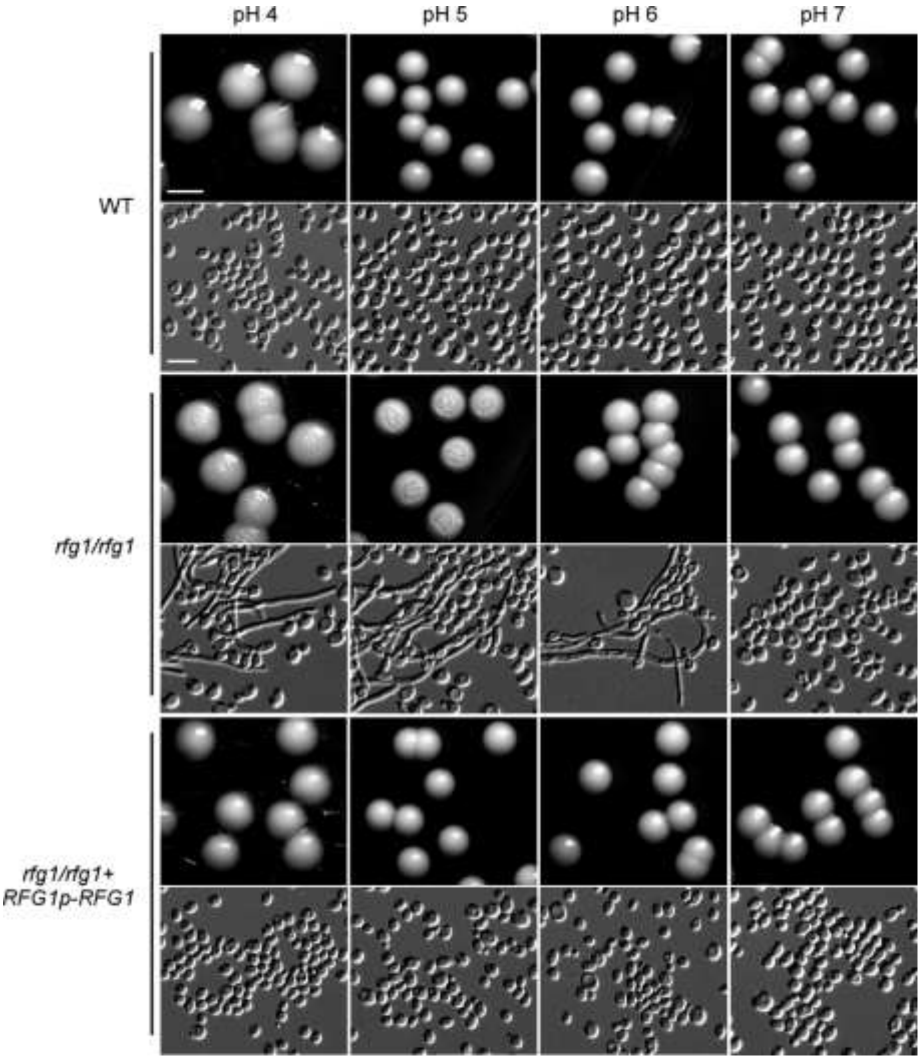

Fig. S2 *rfg1/rfg1* mutant could undergo filamentous growth at low temperature under acidic pH conditions

Cells of the WT (SN250), *rfg1/rfg1* mutant and *rfg1/rfg1+RFG1p-RFG1* reconstituted strain were grown on YPD-K medium (pH 4, pH 5, pH 6, pH 7). The colony and cellular images were taken after 3 days of growth at 25°C. Scale bar for colonies, 2.5 mm; scale bar for cells, 10 μm.

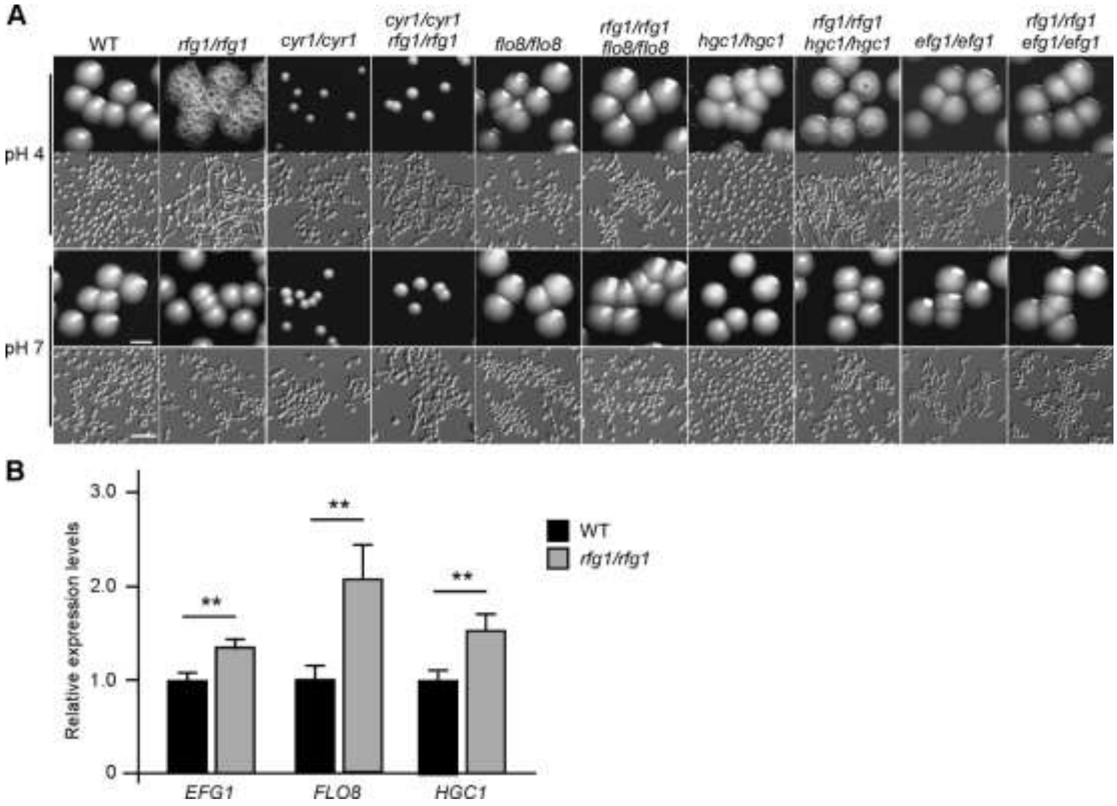

Fig. S3 Role of the cAMP signaling pathway, Flo8, Hgc1, and Efg1 in Rfg1 mediated pH response

A. Morphologies of the *cyr1/cyr1 rfg1/rfg1*, *rfg1/rfg1 flo8/flo8*, *rfg1/rfg1 hgc1/hgc1* and *rfg1/rfg1 efg1/rfg1* double mutants under acidic pH conditions. Cells were cultured on YPD-K media (pH 4, pH 7) at 30˚C for 3 days. Scale bar for colonies, 2.5 mm; scale bar for cells, 10 μm. B. Relative expression levels of *EFG1, FLO8, HGC1,* and in the WT (SN250) and *rfg1/rfg1* strains on YPD-K medium (pH 4) at 30˚C. Transcript levels of each gene in the WT were set as '1'. Data are presented as the mean ± SEM. **P < 0.01 (Student's *t*-test, two-tailed).

Table S1. Strains used in this study.

Strain name	Parent strain	Genotype	Reference
HJ25		Natural isolate, *MTL*a/α	1
HJ51		Natural isolate, *MTL*a/α	
HJ25+pACTS	HJ25	As HJ25, but pACTS	This study
HJ25+pACTS-*RFG1*	HJ25	As HJ25, but pACTS-*RFG1*	This study
SN152	CAI4	*MTL*a/α *ura3::imm434::URA3-IRO1/ura3::imm434 arg4::hisG/arg4::hisG his1::hisG/his1::hisG leu2::hisG /leu2::hisG*	2
SN250	CAI4	*MTL*a/α *ura3::imm434::URA3-IRO1/ura3::imm434 arg4::hisG/arg4::hisG his1::hisG/his1::hisG leu2::hisG::CdHIS1/leu2::hisG::CmLEU2*	2
rfg1/rfg1	SN152	*MTLa/α ura3::imm434::URA3-IRO1/ura3::imm434 arg4::hisG/arg4::hisG his1::hisG/his1::hisG leu2::hisG/leu2::hisG rfg1::CdHIS1/rfg1::CmLEU2*	3
rfg1/rfg1-RFG1p-RFG1	SN152	*MTLa/α ura3::imm434::URA3-IRO1/ura3::imm434 arg4::hisG/arg4::hisG his1::hisG/his1::hisG leu2::hisG/leu2::hisG rfg1::CdHIS1/rfg1::CmLEU2::RFG1p-RFG1-FRT-SAT1-FRT*	This study
bcr1/bcr1	SN152	*MTLa/α ura3::imm434::URA3-IRO1/ura3::imm434 arg4::hisG/arg4::hisG his1::hisG/his1::hisG leu2::hisG/leu2::hisG bcr1::CdHIS1/bcr1::CmLEU2*	4
rbt5/rbt5	SN152	*MTLa/α ura3::imm434::URA3-IRO1/ura3::imm434 arg4::hisG/arg4::hisG his1::hisG/his1::hisG leu2::hisG/leu2::hisG rbt5::CdHIS1/rbt5::CmLEU2*	5
sld1/sld1	SN152	*MTLa/α ura3::imm434::URA3-IRO1/ura3::imm434 arg4::hisG/arg4::hisG his1::hisG/his1::hisG leu2::hisG/leu2::hisG sld1::CdHIS1/sld1::CmLEU2*	
cfl11/cfl11	SN152	*MTLa/α ura3::imm434::URA3-IRO1/ura3::imm434 arg4::hisG/arg4::hisG his1::hisG/his1::hisG leu2::hisG/leu2::hisG cfl11::CdHIS1/cfl11::CmLEU2*	
orf19.1092/ orf19.1092	SN152	*MTLa/α ura3::imm434::URA3-IRO1/ura3::imm434 arg4::hisG/arg4::hisG his1::hisG/his1::hisG leu2::hisG/leu2::hisG*	

		orf19.1092::CdHIS1/orf19.1092::CmLEU2	
RFG1-TAP/rfg1	SN152	As SN152, but *rfg1:CdHIS1/RFG1-TAP-ARG4*	This study
WT+pACTS	SN152	As SN152, but pACTS	6
WT+pACTS-*BCR1*	SN152	As SN152, but pACTS-*BCR1*	This study
WT+pACTS-*RFG1*	SN152	As SN152, but pACTS-*RFG1*	This study
rfg1/rfg1+pACTS-*BCR1*	SN152	As SN152, but *rfg1::CdHIS1/rfg1::CmLEU2*::pACTS-*BCR1*	This study
bcr1/bcr1+pACTS-*RFG1*	SN152	As SN152, but *bcr1::CdHIS1/bcr1::CmLEU2*::pACTS-*RFG1*	This study
phr1/phr1	CAS8	*MTLa/α ura3::imm434/ura3::imm434 phr1::hisG/phr1::URA3*	7
phr1/phr1 rfg1/rfg1	CAS8	As CAS8, but *rfg1::FRT1/rfg1::FRT-SAT1-FRT*	This study
phr2/phr2	CFM-2	*MTLa/α ura3::imm434/ura3::imm434 phr2::hisG/phr2::hisG-URA3-hisG*	8
phr2/phr2 rfg1/rfg1	CFM-2	As CFM-2, but *rfg1::FRT1/rfg1::FRT-SAT1-FRT*	This study
rim101/rim101	CAR2	*MTLa/α ura3::imm434/ura3::imm434 prr2::hisG/prr2::URA3*	9
rim101/rim101 rfg1/rfg1	CFM-2	As CAR2, but *rfg1::FRT1/rfg1::FRT-SAT1-FRT*	This study
cyr1/cyr1	SC5314	*MTLa/a cyr1::FRT/cyr1::FRT*	10
cyr1/cyr1 rfg1/rfg1	SC5314	As GH1404, but *rfg1::FRT1/rfg1::FRT-SAT1-FRT*	This study
flo8/flo8	CCF3	*MTLa/a ura3::imm434/ura3::imm434 flo8::hisG/flo8::hisG-URA3-hisG*	11
rfg1/rfg1 flo8/flo8	CCF3	As *rfg1/rfg1*, *flo8::arg4/flo8::FRT-SAT1-FRT*	This study
hgc1/hgc1	SN250	As SN250, but *hgc1::arg4/hgc1::FRT-SAT1-FRT*	This study
rfg1/rfg1 hgc1/hgc1	SN250	As *rfg1/rfg1,* but *hgc1::arg4/hgc1::FRT-SAT1-FRT*	This study
efg1/efg1	SN152	*MTLa/α ura3::imm434::URA3-IRO1/ura3::imm434 arg4::hisG/arg4::hisG his1::hisG/his1::hisG leu2::hisG /leu2::hisG bcr1::CdHIS1/bcr1::CmLEU2*	4
rfg1/rfg1 efg1/efg1	SN152	As *rfg1/rfg1*, *efg1::FRT/efg1:: FRT-SAT1-RFT*	This study

Reference:

1. Hu J, Guan G, Dai Y, Tao L, Zhang J, Li H, Huang G (2016). Phenotypic diversity and correlation between white–opaque switching and the CAI microsatellite locus in

Candida albicans. Curr Genet. 62 (3): 585-593.

2. Noble SM, Johnson AD (2005). Strains and strategies for large-scale gene deletion studies of the diploid human fungal pathogen *Candida albicans*. Eukaryot Cell. 4: 298-309.
3. Liang W, Guan G, Dai Y, Cao C, Tao L, Du H, Nobile CJ, Zhong J, Huang G (2016). Lactic acid bacteria differentially regulate filamentation in two heritable cell types of the human fungal pathogen *Candida albicans*. Mol Microbiol. 102 (3): 506-519.
4. Homann OR, Dea J, Noble SM, and Johnson AD (2009). A phenotypic profile of the *Candida albicans* regulatory network. PLoS Genet. 5, e1000783.
5. Noble SM, French S, Kohn LA, Chen V, Johnson AD (2005). Systematic screens of a *Candida albicans* homozygous deletion library decouple morphogenetic switching and pathogenicity. Nat Genet. 42 (7): 590-598.
6. Guan G, Tao L, Yue H, Liang W, Gong J, Bing J, Zheng Q, Veri AO, Fan S, Robbins N, Cowen LE, Huang G (2019). Environment-induced same-sex mating in the yeast *Candida albicans* through the Hsf1-Hsp90 pathway. PLoS Biol. 17(3): 1-25.
7. Saporito-Irwin SM, Birse CE, Sypherd PS, Fonzi WA (1995). *PHR1*, a pH-regulated gene of *Candida albicans*, is required for morphogenesis. Mol Cell Biol. 15(2): 601-613.
8. Mühlschlegel FA, Fonzi WA (1997). PHR2 of *Candida albicans* encodes a functional homolog of the pH-regulated gene PHR1 with an inverted pattern of pH-dependent expression.Mol Cell Biol. 17 (10): 5960-5967.
9. Ramon AM, Porta A, Fonzi WA (1999). Effect of environmental pH on morphological development of *Candida albicans* is mediated via the PacC-related transcription factor encoded by PRR2. J Bacteriol.181 (24): 7524-7530.
10. Yi S, Sahni N, Daniels KJ, Lu KL, Srikantha T, Huang G, Garnaas AM, Soll DR (2011). Alternative mating type configurations (a/alpha versus a/a or alpha/alpha) of *Candida albicans* result in alternative biofilms regulated by different pathways. PLoS Biol 9, e1001117.
11. Cao F, Lane S, Raniga PP, Lu Y, Zhou Z, Ramon K, Chen J, and Liu H (2006). The Flo8 transcription factor is essential for hyphal development and virulence in *Candida albicans*. Mol Biol Cell 17, 295-307.

Table S2. Primers used in this study.

Name	Sequence (5' to 3')	Purpose
Rfg1 com-up fwd	TTATATGGGCCCTTCACACACCTGCACACCTAC	For construction of *RFG1* complemented plasmid
Rfg1 com-up rev	AATCATCTCGAGCCCTAACTTATACTTGGTCTGC	
Rfg1 com-down fwd	TAATATCCGCGGAGGGTTAATCCATATAGTTGC	
Rfg1 com-down rev	ATATACGAGCTCCATGGCGCATTGGTTTAGGAC	
RFG1 orf fwd	TAACTTGATATCTCTACTGCTATCTACTATTCAAC	For pACTS-*RFG1* expression plasmid
RFG1 orf rev	TAACTTAAGCTTCGAAAGGGACAAATATTCTCT	
RT-RFG1 fwd	TCCTAACCACACTAATGCTG	For q-RT-PCR
RT-RFG1 rev	TGGATTGATAAGTGTGGAGC	
RT-BCR1 fwd	AGCACCACAACAACAAGTAC	
RT-BCR1 rev	TGAGGTAATGGAGGTAATGG	
RT-RBT5 fwd	GAATCCACCACTGCCGAATC	
RT-RBT5 rev	AGATTCTTCAGCTTTGGTGG	
RT-CFL11 fwd	GTGCCATTGAGGTTTGAGTC	
RT-CFL11 rev	GGTACACAGAATCCAGCAAG	
RT-SLD1 fwd	GTGGACAAGGACCTAGACAG	
RT-SLD1 rev	CCTATTCCAACCACCTTCAA	
RT-orf19.1092 fwd	GATGTGGATGTTAGAAGAATGG	
RT-orf19.1092 rev	TAGCAAATCCATCTGATGTG	
RT-PHR1 fwd	AGGATCATGAAGAGTGTATG	
RT-PHR1 rev	CAGAAGCATCGGTATTTGAA	
RT-ENA2 fwd	GTTGTGTGATTACTGGTACG	
RT-ENA2 rev	ATCGCCAAGTTCTTTAGTTGC	
RT-CRZ2 fwd	CAACTGGCACCACAACAAG	
RT-CRZ2 rev	GATGATGTTGATGATGGAG	
RT-RBT4 fwd	GATGCTGATGGTGGTAATG	
RT-RBT4 rev	GAACGTCTCCATAGGTATG	
RT-HWP1 fwd	AAAATCAGATGTTCCAGCTAC	
RT-HWP1 rev	TGGTTTAGTTTCAGTACCAGC	
RT-ECE1 fwd	ACCATGCTCCAGAATTCAAC	
RT-ECE1 rev	TGGATTACTTGTGGAATGTTG	
RT-EFG1 fwd	CAACCTCAGCATTACAATG	
RT-EFG1 rev	AGGAGTTGGTTGTTGCATTG	
RT-ZCF3 fwd	TGTCAAGCCTGTACCAGAAAC	

RT-ZCF3 rev	GGTTCAGGTTGAGAGTGTAG	
RT-STP4 fwd	TAACCATGACTTCTCCACCAC	
RT-STP4 rev	CTATTGGAAGGTGTGGGTG	
RT-WOR3 fwd	ACTGTACTCCAACAACAACC	
RT-WOR3 rev	TTGCTCGTAACGGTTTTGTG	
RT-HYR1 fwd	TGGCTCTCAAACTGGTTCTG	
RT-HYR1 rev	TCCAGAACCTTGACCTGAAC	
RT-IHD1 fwd	CTAATGGTAACGATGGTGC	
RT-IHD1 rev	CCAGAACCAGCAGTATTAGTG	
BCR1 orffwd- StuI	CCAaggcctTCAGGGACATCACAAGTACTTC	For pACTS-*BCR1* expression plasmid
BCR1 orf rev-HindIII	TGAAAGCTTCACTGTCGTCACCCCAATACTG	
Rfg1 up-fwd	TTATATGGGCCCTTCACACACCTGCACACCTAC	For construction of pSFS2a-*RFG1* KO plasmid
Rfg1 up-rev	AATCATCTCGAGTTAATGGTGTGATGGTTTGC	
Rfg1 down fwd	TAATATCCGCGGAGGGTTAATCCATATAGTTGC	
Rfg1 down rev	ATATACGAGCTCCATGGCGCATTGGTTTAGGAC	
Rfg1 5 detect	CCTGTGTTATATTCATTCAAGG	*RFG1* deletion confirmation
Rfg1 3 detect	CCGTGAAACAATTATACTGAGAC	
Rfg1 check fwd	AGTTGGTGGTGGTAATGGTGG	
Rfg1 check rev	ACCACTGAGACTACTAGCAC	
RFG1 5'flank forward	TCATTTGGGATACTACTACTAG	Fusion PCR for *RFG1* KO
RFG1 5'flank bottom	CACGGCGCGCCTAGCAGCGGATGATGATGATAAACACAGAC	
RFG1 3'flank top	GTCAGCGGCCGCATCCCTGCAGAGAATATTTGTCCCTTTTCGTG	
RFG1 3'flank bottom	TATGTATGTATGTATGTATGTGCGTG	
LT31	CCGCTGCTAGGCGCGCCGTGGTTTTCCCAGTCACGACGTT	
LT32	GCAGGGATGCGGCCGCTGACTGTGGAATTGTGAGCGGATA	
FLO8-5DR	GGAAAGAAACTTATCAACTTAGCAGCAAACTGACATTAAAGGCAGCCAAATCTGAGTCTGgttttcccagtcacgacgtt	Deletion of the first copy of *FLO8* gene
FLO8-3DR	TACATTAGTTAGTATTTTATCATTGATTATTGGTGCAACTTGGTGGAATTTTGATCATTGtgtggaattgtgagcggata	
5-detect（FLO8）	CAACCAATCTACATCTCACTC	
3-detect（FLO8）	CATGAATGGTGTTGGAATG	
FLO8 up (in) fwd	TATGGGCCCCATTCCATCCATCCATCCATT	For construction of

	C	pSFS2a-*FLO8* KO plasmid
FLO8 up (in) rev	CACGGCGCGCCTAGCAGCGGCTCGAGTGCCAGCAATAATTGTAGT	
FLO8 down (in) fwd	GTCAGCGGCCGCATCCCTGCCCGCGGTTAATTTGAAGTTTCGGTTGGT	
FLO8 down (in) rev	TACGAGCTCCCCACCATCAGTCAATTTATC	
Flo8 check fwd	CATCAAGGCCAACCTCAAG	*FLO8* deletion confirmation
Flo8 check rev	AGTGCCGTTTGACATCTGACC	
Flo8 check2 fwd	CAGCCTTCAATGGAACAAC	
Flo8 check2 rev	TGTTGTTGTTGTTGGCTCTGG	
HGC1-5DR	TTCTTCCATACAAGAAGAGTCCAAACAAAATCTCAGAGAGAAATTCTGTCCTCCTCCCCTgttttcccagtcacgacgtt	Deletion of the first copy of *HGC1* gene
HGC1-3DR	AGTAACAATTCAGGACGAATAAAGGATACTTTCCAGTAGTGTATTATAGATTATCGCTTGtgtggaattgtgagcggata	
3-detect（HGC1）	GAGTTGACCATAAAGTAATTGC	
HGC1 up (in) fwd:	TATGGGCCCGATAGAAACTCGCTTACAAC	For construction of pSFS2a-*HGC1* KO plasmid
HGC1 up (in) rev	CACGGCGCGCCTAGCAGCGGCTCGAGGTTGGGTATTGGTTTGATGC	
HGC1 down (in) fwd	GTCAGCGGCCGCATCCCTGCCCGCGGGTAGAGAATGGAGAATGGAG	
HGC1 down (in) rev	TACGAGCTCTAGGGAAGATGTAAATGGG	
Hgc1 check fwd	GATTTAGGTTATGGATGTGGTG	*HGC1* deletion confirmation
Hgc1 check rev	ATGATACTGGAGTAGTAGAGCC	
Efg1 up (out) fwd	TTATATGGGCCCCATTTAGCTGCTATTTCAACC	For construction of pSFS2a-*EFG1* KO plasmid-1
Efg1 up (out) rev	AATCATCTCGAGAGTTAAGTGGGTTGGCTGGATG	
Efg1 down (out) fwd	TAATATCCGCGGGAGATGATAGTTTGTGGCGTG	
Efg1 down (out) rev	ATATACGAGCTCATCACCTGTAACTCGTGTCG	
Efg1 up (in) fwd	TTATATGGGCCCGAAGAGACAAGCAAACAAACG	For construction of pSFS2a-*EFG1* KO plasmid-2
Efg1 up (in) rev	AATCATCTCGAGTGGGTTATATTCTTGGTAGTC	
Efg1 down (in) fwd	TAATATCCGCGGGTTCAGTTCACCCTTCACC	
Efg1 down (in) rev	ATATACGAGCTCAAGACAATGACTTACACAC	
Efg1 5’detect	TATCCCAACTTTAATTCCTTCC	*EFG1* deletion confirmation
Efg1 3’detect	TGATGATACGGTTAGAAGTC	

Efg1 orf check fwd	ACCTTCCAATTCTACCAGTG	
Efg1 orf check rev	TCCAGACTCCTTTCAAATGC	
BCR1-UP FWD	TTATATGGGCCCAACCACAACAAATCAACCAACC	For construction of pSFS2a-*BCR1* KO plasmid
BCR1-UP REV	AATCATCTCGAGGTTGTTGTAGTTGTTGTTTTTG	
BCR1-DOWN FWD	TAATATCCGCGGACAGTATTGGGGTGACGACAGTG	
BCR1-DOWN REV	ATATACGAGCTCCAACAATCTCCCTTCCCAAATAC	
Bcr1 5'detect	GTCATTCACATACTTTCTTCC	*BCR1* deletion confirmation
d2(723) rev:	CAATGAAATCCAGACAGTCGAG	
Bcr1 3'detect	CGATTAGAGACACAAACGAAC	
d4(723) rev:	GATAGGATAGAACAGAACAGGC	
BCR1-5DR	AATTAACTAATTCAATCAATCAACCACAACAAATCAACCAACCAACCAACTACCATTTACgttttcccagtcacgacgtt	Deletion of the second copy of *BCR1* gene
BCR1-3DR	TGTAAATCAAGTAGAACACTCATACTCAGTTTATATAACAAACGAGTAAAGTAAGAACACtgtggaattgtgagcggata	
BCR1 orf check fwd	ATGAGCATTAATTTTAGGTGGTGG	*BCR1* deletion confirmation
BCR1 orf check rev	TTAATTGAACGATCTAAACTGAAAAAG	
RFG1-TAP 5DR	CATGCTGCTCAACATTATCAACAACAACAACAACAACATCATCAACAACAACCTCCACAAGGTCGACGGATCCCCGGGTT	For construction of Rfg1-TAP strain
RFG1-TAP 3DR	TTTTTTGGTTCAATTCATATGTATCTAAAAAAATTGTAAAAAATGATTGTATTGTAGAAATCGATGAATTCGAGCTCGTT	
RFG1-TAP check fwd	CTCCAGTATCATCAACTCATC	Confirm the Rfg1-TAP-tagged strain
RFG1-TAP check rev	GAACCCTAACTTATACTTGGTC	
TAP-R	TAAACTTTGGATGAAGGCG	
ARG4-F	ATGTTGGCTACTGATTTAGCTG	
bcr1 binding F	tcataatggtttggtccttgac	ChIP-PCRs
bcr1 binding R	agactaaggcgacatactaag	
ADE2 RT-fwd	GTTGTCAGATCATTAGAAGGGGAAG	
ADE2 RT-rev	AAGTATCTGGGATCCTGGCA	
