# Supplementary material for: The Rfg1 and Bcr1 transcription factors regulate acidic pH–induced filamentous growth in Candida albicans
Source: Microbiol Spectr. 2023 Nov 7;11(6):e01789-23. doi: 10.1128/spectrum.01789-23 (PMC10715123; doi:10.1128/spectrum.01789-23)
Supplement: Dataset S1 — RNA-Seq data. [file spectrum.01789-23-s0001.pdf]

Expression profiles of all genes

Gene_ID	GENE	FPKM of WT-1	FPKM of WT-2	FPKM of *rfg1* Δ/Δ-1	FPKM of *rfg1* Δ/Δ-2	Log2(*rfg1* Δ/Δ /WT)	p value	Description
orf19.1105.2	PGA56	23330.69	24531.33	24126.6	26999.46	0.079403917	0.998825	Regulator of sorbose utilization; putative GPI-anchor; hypha and cell wall regeneration induced; colony morphology-related gene regulation by Ssn6; induced by Tbf1, Hap43; Spider biofilm induced
orf19.3548.1	WH11	23327.03	23887.34	22553.68	23426.34	-0.048300185	0.998825	White-phase yeast transcript; expression in opaques increases virulence/switching; mutant switches as WT; Hap43, hypoxia, ketoconazol induced; required for RPMI biofilm; Bcr1-induced in RPMI a/a biofilm; rat catheter, Spider biofilm induced
orf19.7350	RCT1	12247.87	12486.08	13124.92	12712.04	0.04716553	0.998825	Fluconazole-induced protein; Ras1, Cyr1 repressed and Efg1 induced; regulated by Nrg1, Tup1, Tbf1, Ssn6; induced in oralpharyngeal candidasis; rat catheter biofilm repressed
orf19.3940.1	CUP1	8508.98	9111.69	5791.12	6301.75	-0.5266007	0.002138	Metallothionein; involved in copper resistance; copper induced; Spider biofilm induced; flow model biofilm repressed
orf19.382	TEF2	8683.48	8790.85	9535.66	9697.53	0.120323586	0.918336	Translation elongation factor 1-alpha; genes encoding ribosomal subunits, translation factors, and tRNA synthetases are downregulated upon phagocytosis by murine macrophage
orf19.930	PET9	7917.67	8101.34	6551.34	6628.31	-0.281126045	0.229994	Mitochondrial ADP/ATP carrier protein involved in ATP biosynthesis; possible lipid raft component; 3 predicted transmembrane helices; flucytosine induced; ketoconazole-induced; downregulated by Efg1p
orf19.5117	OLE1	7403.83	8028.62	8179.7	8258.98	0.073178031	0.998825	Fatty acid desaturase, essential protein involved in oleic acid synthesis; required for aerobic hyphal growth and chlamydospore formation; subject to hypoxic regulation; fluconazole-induced; caspofungin repressed; Hap43p-induced
orf19.2608	ADH5	7001.79	7724.15	8353.73	8994.23	0.211227242	0.606463	Putative alcohol dehydrogenase; regulated by white-opaque switch; fluconazole-induced; antigenic in murine infection; regulated by Nrg1, Tup1; Hap43, macrophage repressed, flow model biofilm induced; Spider biofilm induced

orf19.1435	TEF1	7725.53	7506.35	8709.91	8906.65	0.189707993	0.612468	Translation elongation factor 1-alpha; at cell surface; binds human plasminogen; macrophage/pseudohyphal-induced; induced in RHE model of oral candidiasis, in clinical oral candidiasis isolates; possibly essential; Spider biofilm repressed
orf19.670.2	orf19.670.2	5911.9	6002.02	3062.84	3116.17	-0.915151668	2.74E-11	Protein of unknown function; hypoxia, Hap43-repressed; ketoconazole induced; induced in oralpharyngeal candidasis; 16h flow model biofilm repressed, late-stage flow model biofilm induced; rat catheter and Spider biofilm induced
orf19.3942.1	RPL43A	5435.42	5848.53	5137.28	5596.4	-0.08021626	0.998825	Putative ribosomal protein, large subunit; repressed by human whole blood or PMNs; colony morphology-related gene regulation by Ssn6; Spider biofilm repressed
orf19.1470	RPS26A	5551.76	5720.82	5156.31	5925.66	-0.033199384	0.998825	Ribosomal protein; regulated by Nrg1, Tup1; repressed upon phagocytosis by murine macrophage; alternatively spliced intron in 5'-UTR; Spider biofilm repressed
orf19.2765	PGA62	4695.68	5645.47	5614.57	6138.06	0.159069835	0.872276	Adhesin-like cell wall protein; putative GPI-anchor; fluconazole-induced; induced in high iron; induced during cell wall regeneration; Cyr1 or Ras1 repressed; Tbf1 induced
orf19.4909.1	RPL42	5004.4	5572.03	5485.28	6983.27	0.211210818	0.732635	Putative 60S ribosomal subunit protein; colony morphology-related gene regulation by Ssn6; Spider biofilm repressed
orf19.6264.4	orf19.6264.4	5042.03	5562.7	5102.53	6536.24	0.11557204	0.998825	Ortholog of S. cerevisiae Rpl39; a component of the 60S ribosomal subunit; Hap43-induced; Spider biofilm repressed
orf19.2107.1	STF2	4865.98	5302.25	6039.99	6877.32	0.314175305	0.233294	Protein involved in ATP biosynthesis; repressed in hyphae; repressed by Efg1, Hap43; transcript upregulated in clinical isolates from HIV+ patients with oral candidiasis; rat catheter, flow model and Spider biofilm induced
orf19.827.1	RPL39	4859.13	5223.59	4501.99	5622.15	-0.003651037	0.998825	Ribosomal protein L39; transcript induced upon germ tube formation; colony morphology-related gene regulation by Ssn6; Hap43-induced

orf19.3087	UBI3	5096.47	5217.1	4687.94	5221.77	-0.064894629	0.998825	Fusion of ubiquitin with the S34 protein of the small ribosomal subunit; mRNA decreases upon heat shock, appears to be degraded; functional homolog of S. cerevisiae RPS31; Hap43-induced; Spider biofilm repressed
orf19.6814	TDH3	4212.17	5183.46	7763.38	9056.67	0.768059539	2.47E-05	NAD-linked glyceraldehyde-3-phosphate dehydrogenase; binds fibronectin, laminin; at cell surface; antigenic in infection; farnesol-repressed; stationary phase-enriched; GlcNAc-induced; flow model biofilm induced; Spider biofilm repressed
orf19.4623.3	NHP6A	5096.5	5108.86	5144.74	6414.53	0.160614176	0.87024	Putative non-histone chromatin component; RNA abundance regulated by tyrosol,cell density; Hap43-induced; amphotericin B, caspofungin repressed; 5'-UTR intron; rat catheter and Spider biofilm repressed
orf19.6253	RPS23A	5123.98	5078.27	4680.38	5108.61	-0.066806822	0.998825	Putative ribosomal protein; repressed upon phagocytosis by murine macrophage; Spider biofilm repressed
orf19.6663	RPS25B	4780.42	4845.71	4686.56	5012.01	-0.000638458	0.998825	Ribosomal protein; macrophage/pseudohyphal-induced after 16 h; repressed upon phagocytosis by murine macrophage; transcript positively regulated by Tbf1; 5'-UTR intron; Hap43-induced; Spider biofilm repressed
orf19.6771	UBI4	4450.34	4845.55	3255.96	3717.88	-0.401389332	0.062824	Ubiquitin precursor (polyubiquitin) contains 3 tandem repeats of the ubiquitin peptide that are processed to individual units; transcript induced by stress; mRNA in yeast and mycelia at similar abundance; Spider biofilm induced
orf19.5964.2	RPL35	4472.27	4789.1	4273.95	4830.49	-0.034284031	0.998825	Ribosomal protein; downregulation correlates with clinical development of fluconazole resistance; colony morphology-related gene regulation by Ssn6; Hap43-induced; Spider biofilm repressed
orf19.7018	RPS18	4517.97	4783.06	4266.33	5027.46	-0.011120252	0.998825	Predicted ribosomal protein; repressed upon phagocytosis by murine macrophage; repressed by nitric oxide; Hap43-induced; Spider biofilm repressed
orf19.2864.1	RPL28	4621.51	4737.48	4363.84	4935.58	-0.018748174	0.998825	Putative ribosomal protein; Plc1-regulated; downregulated upon phagocytosis by murine macrophage; Spider biofilm repressed

orf19.2767	PGA59	4606.95	4727.18	4195.62	4156.42	-0.165726388	0.769575	Adhesin-like cell wall protein; putative GPI-anchor; colony morphology-related gene regulation by Ssn6; Hap43-induced; Spider biofilm repressed
orf19.3997	ADH1	4169.16	4725.19	5260.7	5631.58	0.262808951	0.405369	Alcohol dehydrogenase; oxidizes ethanol to acetaldehyde; at yeast cell surface; immunogenic in humans/mice; complements S. cerevisiae adh1 adh2 adh3 mutant; fluconazole, farnesol-induced; flow model biofilm induced; Spider biofilm repressed
orf19.2310.1	RPL29	4159.35	4621.51	4336.47	4329.69	-0.032169544	0.998825	Ribosomal protein L29; induced upon germ tube formation; colony morphology-related gene regulation by Ssn6; intron in 5'-UTR; Spider biofilm repressed
orf19.5466	RPS24	4330.55	4614.29	3940.7	4103.91	-0.157774206	0.83812	Predicted ribosomal protein; hyphal downregulated; repressed upon phagocytosis by murine macrophage; transcriptionally activated by Tbf1; Spider biofilm repressed
orf19.6882.1	orf19.6882.1	4198.54	4464.57	3855.48	4829.56	-0.005576991	0.998825	Ribosomal 60S subunit protein; Spider biofilm repressed
orf19.5996.1	RPS19A	4210.44	4415.51	3606.02	3909	-0.19995174	0.645919	Putative ribosomal protein S19; protein level decreases in stationary phase cultures; Spider biofilm repressed
orf19.6873	RPS8A	4158.81	4357.68	3986.47	4221.81	-0.062471922	0.998825	Small 40S ribosomal subunit protein; induced by ciclopirox olamine; repressed upon phagocytosis by murine macrophage; 5'-UTR intron; Hap43-induced; Spider biofilm repressed
orf19.4375.1	RPS30	4588.22	4281.33	4305.09	4549.25	-0.011875719	0.998825	Putative 40S ribosomal protein S30; colony morphology-related gene regulation by Ssn6; rat catheter biofilm repressed
orf19.3325.3	RPS21B	3739.74	4150.48	3646.99	4219.08	-0.015360578	0.998825	Ribosomal protein S21; regulated by Nrg1, Tup1; colony morphology-related gene regulation by Ssn6; positively regulated by Tbf1, Hap43; Spider biofilm repressed

orf19.6085	RPL16A	3924.66	4097.6	3538.7	3833.57	-0.12687599	0.924887	Ribosomal protein; transposon mutation affects filamentous growth; repressed upon phagocytosis by murine macrophages; Hap43-induced; Spider biofilm repressed
orf19.4082	DDR48	3729.24	4059.42	7418.01	8639.67	0.970999333	5.23E-10	Immunogenic stress-associated protein; filamentation regulated; induced by benomyl/caspofungin/ketoconazole or in azole-resistant strain; Hog1, farnesol, alkaline repressed; stationary phase enriched; Spider, flow model biofilm induced
orf19.6375	RPS20	3710.71	4012.82	3578.19	4194.5	-0.001902547	0.998825	Putative ribosomal protein; repressed upon phagocytosis by murine macrophage; transcript positively regulated by Tbf1; Spider biofilm repressed
orf19.667.1	RPL37B	3741.94	3962.96	3226.96	3454.78	-0.206590671	0.612468	Ribosomal protein L37; Hap43-induced; Spider biofilm repressed
orf19.2111.2	RPL38	3572.77	3925.9	3535.21	4118.62	0.01679778	0.998825	60S ribosomal ribosomal protein subunit; genes encoding cytoplasmic ribosomal subunits, translation factors, tRNA synthetases are downregulated upon phagocytosis by murine macrophage
orf19.493	RPL15A	3508.6	3922.28	3176.32	3667.1	-0.122944778	0.952263	Putative ribosomal protein; repressed upon phagocytosis by murine macrophage; positively regulated by Tbf1; Spider biofilm repressed
orf19.4914.1	BLP1	4889.43	3873.41	4037.48	3669.38	-0.184575534	0.725007	Protein of unknown function, serum-induced
orf19.5904	RPL19A	3820.59	3859.14	3509.49	3616.1	-0.114714309	0.938642	Ribosomal protein L19; repressed upon phagocytosis by murine macrophages; Hap43-induced gene; Spider biofilm repressed
orf19.7048.1	RPS28B	3746.46	3725.63	3294.98	3780.95	-0.083462963	0.998825	Putative ribosomal protein S28B

orf19.4931.1	RPL14	3759.3	3719.07	3115.61	3316.95	-0.217119529	0.543746	Ribosomal protein L14; promoter bound directly by Tbf1p; Hap43p-induced gene
orf19.1354	UCF1	3392.72	3698.21	3944.42	4643.22	0.248539946	0.520851	Upregulated by cAMP in filamentous growth; induced in high iron, decreased upon yeast-hypha switch; downregulation correlates with clinical fluconazole resistance; Ras1-regulated; Hap43-repressed; flow model biofilm induced
orf19.2309.2	RPL2	3524.6	3692.85	3240.06	3463.44	-0.112923565	0.951606	Putative 60S ribosomal protein L2; Hap43-induced gene; repressed in infected rabbit kidney in SC5314, but not NGY152, strain background; Spider biofilm repressed
orf19.2994	RPL13	3440.63	3638.57	2996.62	3281.69	-0.175194923	0.789467	Putative ribosomal subunit; antigenic during murine infection; downregulated upon phagocytosis by murine macrophage; Spider biofilm repressed
orf19.4684.2	RPL40B	3492.71	3547.59	3440.58	4034.14	0.072052214	0.998825	Protein with similarity to the ribosomal protein portion of S. cerevisiae Rpl40Bp; colony morphology-related gene regulation by Ssn6; positively regulated by Tbf1; Spider biofilm repressed
orf19.5927	RPS15	3369.16	3509.44	3230.47	3356.61	-0.071337613	0.998825	Putative ribosomal protein; macrophage/pseudohyphal-induced after 16 h; repressed upon phagocytosis by murine macrophage; Spider biofilm repressed
orf19.2179.2	RPS10	3190.92	3492.09	3061.47	3610.42	-0.01301399	0.998825	Ribosomal protein S10; downregulated in the presence of human whole blood or PMNs; Spider biofilm repressed
orf19.1779	MP65	3194.86	3440.15	3182.5	3359.52	-0.031506044	0.998825	Cell surface mannoprotein; cell-wall glucan metabolism, adhesion; adhesin motif; O-glycosylation; induced by heat, germ tube formation, wall regeneration; mycelial antigen; diagnostic marker; fluconazole-repressed; Spider biofilm induced
orf19.5225.2	RPL27A	3102.3	3424.43	2864.31	3356.32	-0.075790249	0.998825	Ribosomal protein L27; Spider biofilm repressed

orf19.5982	RPL18	3067.12	3377.56	2577.79	2994.21	-0.207899807	0.692027	Predicted ribosomal protein; Plc1p-regulated, Tbf1-activated; repressed upon phagocytosis by murine macrophage; Hap43p-induced; Spider biofilm repressed
orf19.1635	RPL12	3369.28	3370.81	3156.15	3454.68	-0.036720165	0.998825	Ribosomal protein L12, 60S ribosomal subunit; downregulated by human whole blood or polymorphonuclear cells; genes encoding cytoplasmic ribosomal subunits are downregulated upon phagocytosis by macrophage; Tbf1p-activated; Hap43p-induced
orf19.2935	RPL10	3235.94	3338.24	2865.2	3002.24	-0.167633148	0.789669	Ribosomal protein L10; intron in 5'-UTR; downregulated upon phagocytosis by murine macrophage; transcription regulated by yeast-hypha switch; Spider biofilm repressed
orf19.2994.1	RPS16A	3089.74	3305.97	2920.89	3165.24	-0.079567779	0.998825	Putative 40S ribosomal subunit; macrophage/pseudohyphal-induced after 16 h; Spider biofilm repressed
orf19.3002	RPS1	3179.22	3269.8	2949.4	3186.14	-0.079351654	0.998825	Putative ribosomal protein 10 of the 40S subunit; elicits host antibody response during infection; transcript induced during active growth; Spider biofilm repressed
orf19.687.1	RPL25	3028.22	3267.12	2818.47	3291.59	-0.05088643	0.998825	Putative rRNA-binding ribosomal protein component of the 60S ribosomal subunit; Hap43-induced; colony morphology-related gene regulation by Ssn6
orf19.6220.4	orf19.6220.4	2876.11	3265.83	2562.92	2888.2	-0.173631838	0.840766	Ribosomal 60S subunit protein; Spider biofilm repressed
orf19.7514	PCK1	2976.06	3206.91	2688.74	2870.87	-0.157118451	0.854173	Phosphoenolpyruvate carboxykinase; glucose, C-source, yeast-hypha, Hap43 regulated; fluconazole, phagocytosis, H2O2, oral candidasis, Spider/rat catheter/flow model biofilm induced; repressed in biofilm by Bcr1, Tec1, Ndt80, Rob1, Brg1
orf19.3504	RPL23A	2923.77	3175.13	2660.26	2871.34	-0.145345643	0.880321	Ribosomal protein; downregulated upon phagocytosis by murine macrophage; Hap43-induced; sumoylation target; Spider biofilm repressed

orf19.4660	RPS6A	3032.22	3157.4	2604.77	2998.43	-0.145251215	0.89191	Ribosomal protein 6A; localizes to cell surface of yeast cells but not hyphae; repressed upon phagocytosis by murine macrophage; possibly essential; Hap43-induced; Spider biofilm repressed
orf19.2329.1	RPS17B	3109.91	3157.34	2927.44	3172.43	-0.047936264	0.998825	Ribosomal protein 17B; downregulated upon phagocytosis by murine macrophages; Hap43-induced; Spider biofilm repressed
orf19.4149.1	orf19.4149.1	2909.71	3155.6	2692.04	2890.82	-0.125328527	0.938404	Protein component of the small (40S) ribosomal subunit; Spider biofilm repressed
orf19.3572.3	orf19.3572.3	2833.86	3143.53	2679.05	3115.54	-0.053138752	0.998825	Ribosomal 60S subunit protein L31B; Spider biofilm repressed
orf19.3334	RPS21	2821.02	3042.71	2536.9	2749.62	-0.153061785	0.866583	Protein component of the small (40S) subunit; repressed upon phagocytosis by murine macrophage; positively regulated by Tbf1; Spider biofilm repressed
orf19.5928	RPP2B	2880.38	3013.2	2555.12	2845.26	-0.129845949	0.929146	Conserved acidic ribosomal protein; possibly involved in regulation of translation elongation; interacts with Rpp1A; 1 of 4 similar C. albicans proteins (Rpp1A, Rpp1B, Rpp2A, Rpp2B); macrophage/pseudohyphal-induced; Spider biofilm repressed
orf19.675	orf19.675	2468.37	2988.28	2321.93	2350.24	-0.224979466	0.616255	Cell wall protein; induced in core stress response and core caspofungin response; iron-regulated; amphotericin B, ketoconazole, and hypoxia induced; regulated by Cyr1, Ssn6; induced in oralpharyngeal candidasis; Spider biofilm repressed
orf19.4149	orf19.4149	2702.18	2986.44	2435.16	2622.79	-0.172189944	0.819869	Predicted protein of unknown function; overlaps orf19.4149.1
orf19.236	RPL9B	2883.61	2973.77	2429.3	2788.54	-0.167208523	0.841359	Ribosomal protein L9; repressed upon phagocytosis by murine macrophages; repressed by nitric oxide; protein levels decrease in stationary phase; Hap43-induced; Spider biofilm repressed

orf19.838.1	RPS9B	2722.64	2970.09	2308.89	2596.22	-0.213602688	0.639964	Predicted ribosomal protein; repressed upon phagocytosis by murine macrophage; transcript possibly regulated upon hyphal formation; Spider biofilm repressed
orf19.2023	HGT7	1630.98	2959.65	1248.84	2616.69	-0.106287383	0.998825	Putative MFS glucose transporter; glucose, fluconazole, Snf3 induced, expressed at high glucose; 20 member C. albicans glucose transporter family; 12 TM regions predicted; flow model biofilm induced; Spider biofilm repressed
orf19.3415.1	RPL32	2767.97	2957.79	2547.99	2883.52	-0.082525244	0.998825	Component of the large (60S) ribosomal subunit; Spider biofilm repressed
orf19.5806	ALD5	3665.89	2938.35	3318.8	2389.99	-0.208848642	0.732635	NAD-aldehyde dehydrogenase; decreased expression in fluconazole-resistant isolate, or in hyphae; biofilm induced; fluconazole-downregulated; protein abundance is affected by URA3 expression in the CAI-4 strain; stationary phase enriched
orf19.3690.2	orf19.3690.2	2717.66	2916.17	2485.28	2953.27	-0.057901011	0.998825	Ribosomal 60S subunit protein; Spider biofilm repressed
orf19.6265.1	RPS14B	2826.23	2898.3	2609.85	2837.22	-0.07867623	0.998825	Putative ribosomal protein; repressed upon phagocytosis by murine macrophage; transcript positively regulated by Tbf1; Spider biofilm repressed
orf19.5288	IFE2	2899.57	2897.38	1790.49	1652.55	-0.728924232	6.71E-07	Putative alcohol dehydrogenase; yeast-enriched transcript; Efg1-regulated; induced by prostaglandins, Hog1, fluconazole; rat catheter biofilm induced
orf19.5032	SIM1	2782.12	2885.83	3074.33	3352.11	0.160360765	0.842252	Adhesin-like protein; involved in cell wall maintenance, redundant with Sun41; possibly secreted; macrophage-repressed; repressed by Rim101, Cyr1, Ras1; Spider biofilm induced
orf19.2478.1	orf19.2478.1	2558.43	2877.26	2292.99	2628.57	-0.145594449	0.907282	60S ribosomal protein L7; snoRNA snR39b encoded within the 2nd intron

orf19.840	RPL21A	2554.2	2799.25	2285.72	2527.44	-0.156265712	0.868721	Putative ribosomal protein; repressed upon phagocytosis by murine macrophage; Spider biofilm repressed
orf19.804.1	orf19.804.1	2729.86	2753.1	2451.95	2549.26	-0.13757147	0.881124	Ortholog of S. cerevisiae : YPR010C-A, C. glabrata CBS138 : CAGL0J01699g, C. dubliniensis CD36 : Cd36_02990, C. parapsilosis CDC317 : CPAR2_211020 and Candida tenuis NRRL Y-1498 : CANTEDRAFT_116148
orf19.4490	RPL17B	2576.6	2722.59	2237.97	2409.41	-0.19102292	0.71162	Ribosomal protein L17; mutation confers hypersensitivity to 5-FU, tubercidin; repressed upon phagocytosis by macrophage; Hap43-induced; Spider biofilm repressed
orf19.3003.1	RPL6	2585.29	2688.99	2172.49	2377.18	-0.212687607	0.608044	Ortholog of S. cerevisiae ribosomal subunit, Rpl6B; transposon mutation affects filamentous growth; translation-related genes are downregulated upon phagocytosis by murine macrophage; Hap43-induced; Spider biofilm repressed
orf19.6906	ASC1	2570.41	2660.74	2292.32	2485.65	-0.134768487	0.906554	40S ribosomal subunit similar to G-beta subunits; glucose or N starvation induced filamentation; required for virulence in mice; snoRNA snR24 encoded in ASC1 intron; repressed in stationary phase; GlcNAc-induced; Spider biofilm repressed
orf19.3788.1	RPL30	2459.78	2628.51	2302.39	2565.79	-0.07156724	0.998825	Ribosomal 60S subunit protein; pre-rRNA processing; pre-mRNA alternatively spliced to productive/unproductive transcripts; temp-regulated splicing; colony morphology-related regulation by Ssn6, Tup1, Nrg1 regulated; Spider biofilm repressed
orf19.6286.2	RPS27	2213.23	2618.56	2295.82	2702.23	0.03319244	0.998825	Putative ribosomal protein; repressed upon phagocytosis by murine macrophage; Spider biofilm repressed
orf19.646	GLN1	2686.21	2613.11	1987.13	1928.25	-0.428082209	0.014168	Putative glutamate synthase; regulated by Tsa1, Tsa1B under H2O2 stress conditions; Spider biofilm induced
orf19.3268	TMA19	2661.02	2609.96	3039.75	3142.42	0.207949997	0.569936	Cell wall protein, ortholog of S. cerevisiae Tma19p (Ykl065cp)

orf19.3465	RPL10A	2585.27	2588.44	2319.26	2525.44	-0.100195627	0.998825	Predicted ribosomal protein; downregulated upon phagocytosis by murine macrophages; Hap43-induced; Spider biofilm repressed
orf19.5341	RPS4A	2358.71	2587.61	2247.4	2514.37	-0.06361678	0.998825	Predicted ribosomal protein; repressed upon phagocytosis by murine macrophage; positively regulated by Tbf1; Spider biofilm repressed
orf19.2232	RPL11	2440.41	2535.04	2244.76	2454.16	-0.088871474	0.998825	Ribosomal protein; repressed by phagocytosis; colony morphology-related gene regulation by Ssn6; Hap43-induced; Spider biofilm repressed
orf19.3426	ANB1	2732.83	2498.59	2945.87	2965.79	0.157960982	0.811201	Translation initiation factor eIF-5A; repressed in hyphae vs yeast cells; downregulated upon phagocytosis by murine macrophage; Hap43-induced; GlcNAc-induced protein; Spider biofilm repressed
orf19.4773	AOX2	1983.24	2459.62	221.26	284.98	-2.853126641	5.47E-60	Alternative oxidase; cyanide-resistant respiration; induced by antimycin A, oxidants; growth; Hap43, chlamydospore formation repressed; rat catheter, Spider biofilm induced; regulated in Spider biofilms by Bcr1, Tec1, Ndt80, Brg1
orf19.3789	RPL24A	2363.45	2421.49	2174.11	2340.43	-0.090408778	0.998825	Predicted ribosomal protein; downregulated upon phagocytosis by murine macrophage; intron in 5'-UTR; Hap43-induced; Spider biofilm repressed
orf19.395	ENO1	2021.6	2407.78	3889.34	4272.27	0.810972276	1.87E-06	Enolase; glycolysis and gluconeogenesis; major cell-surface antigen; binds host plasmin/plasminogen; immunoprotective; Hap43, phagocytosis induced; farnesol-repressed; flow model biofilm induced; Spider biofilm repressed; may be essential
orf19.1082.1	orf19.1082.1	2575.56	2385.31	2485.54	2575.77	0.017883044	0.998825	Ortholog(s) have cytochrome-c oxidase activity, role in mitochondrial respiratory chain complex IV assembly and cytosol, mitochondrial intermembrane space, mitochondrial respiratory chain complex IV, nucleus localization
orf19.2959.1	orf19.2959.1	2214.58	2378.4	1830.24	1972.48	-0.269147367	0.383626	Gene induced by hypoxia and ketoconazole; oral infection upregulated; mutants have reduced capacity to damage oral epithelial cells; Spider biofilm induced

orf19.6975	YST1	2230.61	2369.38	2052.25	2233.68	-0.107795488	0.98704	Ribosome-associated protein; antigenic in mice; complements S. cerevisiae yst1 yst2 mutant; similar to laminin receptor; predicted S/T phosphorylation, N-glycosylation, myristoylation, Hap43-, Gcn4-regulated; Spider biofilm repressed
orf19.4193.1	RPS13	2376.44	2365.62	2040.67	2285.18	-0.134868026	0.913857	Putative ribosomal protein of the small subunit
orf19.6541	RPL5	2208.89	2338.32	1932.98	2105.05	-0.173086018	0.811201	Ribosomal protein; repressed upon phagocytosis by murine macrophages; Hap43-induced; Spider biofilm repressed
orf19.2992	RPP1A	2281.81	2332.01	2076.78	2059.62	-0.162167601	0.81271	Conserved acidic ribosomal protein; likely role in regulation of translation elongation; interacts with Rpp2B; 1 of 4 similar C. albicans ribosomal proteins (Rpp1A, Rpp1Bp, Rpp2A, Rpp2B); Hap43-induced; Spider biofilm repressed
orf19.2849	AQY1	2051.43	2319.82	967.48	1224.32	-0.929531204	3.32E-07	Aquaporin water channel; osmotic shock resistance, WT freeze tolerance; virulent in mice; flucytosine repressed; flow model/RPMI/Spider/rat catheter biofilm induced; required for RPMI biofilm formation; Bcr1-induced in a/a RPMI biofilms
orf19.6367	SSB1	2221.96	2284.42	2254.11	2330.27	0.011593004	0.998825	HSP70 family heat shock protein; mRNA in yeast and germ tubes; at yeast cell surface, not hyphae; antigenic in human/mouse infection; macrophage, GCN-induced; possibly essential; sumoylation target; Hap43-induced; Spider biofilm repressed
orf19.6785	RPS12	2121.97	2263.47	1947.31	2089.6	-0.12449635	0.938642	Acidic ribosomal protein S12; regulated by Gcn4, activated by Tbf1; repressed by amino acid starvation (3-AT); protein abundance is affected by URA3 expression in CAI-4 strain background; sumoylation target; Spider biofilm repressed
orf19.7014	orf19.7014	2132.32	2220.77	2072.72	2163.01	-0.049205877	0.998825	Putative protein of unknown function; transcription is positively regulated by Tbf1p; overlaps orf19.7015
orf19.6322	ARD	2371.53	2195.89	1849.42	1665.05	-0.372443477	0.055853	D-arabitol dehydrogenase, NAD-dependent (ArDH); enzyme of D-arabitol and D-arabinose catabolism; D-arabitol is a marker for active infection in humans; rat catheter and Spider biofilm induced

orf19.4336	RPS5	1951.14	2150.28	1705.27	1931.67	-0.173804521	0.841359	Ribosomal protein S5; macrophage/pseudohyphal-induced after 16 h; downregulated upon phagocytosis by murine macrophage; Hap43-induced; Spider biofilm repressed
orf19.6312	RPS3	2018.03	2105.39	1666.34	1853.29	-0.226045217	0.576749	Ribosomal protein S3; Hog1, Hap43-induced; grepressed upon phagocytosis by murine macrophage; present in exponential and stationary phase cells; Spider biofilm repressed
orf19.1467	COX13	2085.57	2087.77	2272.1	2596.11	0.199675581	0.692027	Cytochrome c oxidase; flucytosine induced; repressed by nitric oxide
orf19.4632	RPL20B	1892.19	2081.9	1779	1979.99	-0.087103641	0.998825	Ribosomal protein L20; repressed upon phagocytosis by murine macrophage; alternatively spliced intron in 5' UTR; Hap43-induced; Spider biofilm repressed
orf19.1193	GNP1	2121.26	2048.18	1557.68	1509.05	-0.432976435	0.015023	Similar to asparagine and glutamine permease; fluconazole, caspofungin induced; regulated by Nrg1, Mig1, Tup1, Gcn2, Gcn4, and alkaline regulated by Rim101; repressed during chlamydospore formation; rat catheter, flow model biofilm induced
orf19.5114.1	orf19.5114.1	2122.21	2030.57	978.64	987.45	-1.033488346	5.20E-13	Ortholog of C. dubliniensis CD36 : Cd36_02630, C. parapsilosis CDC317 : CPAR2_206910, Candida tenuis NRRL Y-1498 : cten_CGOB_00051 and Pichia stipitis Pignal : psti_CGOB_00173
orf19.6415.1	orf19.6415.1	2046.98	2030.08	2028.05	2131.76	0.016705915	0.998825	Ortholog(s) have structural constituent of ribosome activity and cytosolic small ribosomal subunit, nucleus localization
orf19.3264.1	orf19.3264.1	2185.88	1979.4	1921.39	1878.3	-0.13667029	0.880321	Protein of unknown function; Spider biofilm repressed
orf19.263.1	orf19.263.1	1991.63	1977.56	1380.87	1530.7	-0.431660139	0.030706	Protein of unknown function; gene has intron; Spider biofilm induced

orf19.33	orf19.33	1907.03	1968.23	2534.22	2924.41	0.454909023	0.023155	Predicted ORF from Assembly 19; removed from Assembly 20; restored based on transcription data; similar to orf19.7550
orf19.7188	RPP1B	1839.75	1954.48	1745.36	1800.55	-0.104722866	0.991735	Conserved acidic ribosomal protein, likely involved in regulation of translation elongation; interacts with Rpp2A; 1 of 4 similar C. albicans ribosomal proteins (Rpp1A, Rpp1B, Rpp2A, Rpp2B); induced by Tbf1; Spider biofilm repressed
orf19.6403.1	RPP2A	1817.57	1941.96	1854.79	1949.16	0.003540701	0.998825	Acidic ribosomal protein; likely role in translation elongation regulation; interacts with Rpp1B; 1 of 4 similar ribosomal proteins (Rpp1A, Rpp1B, Rpp2A, Rpp2B); CUG start codon; upstream uORFs; Tbf1-induced; Spider biofilm repressed
orf19.5213.2	COX9	1864.1	1934.91	1947.33	2229.09	0.118184374	0.957711	Putative subunit VIIa of cytochrome c oxidase; flucytosine induced
orf19.6265	RPS22A	1674.5	1933	1597.85	1875.8	-0.062410655	0.998825	Predicted ribosomal protein; repressed upon phagocytosis by murine macrophage; Spider biofilm repressed
orf19.711	orf19.711	1487.11	1931.41	785.11	1000.89	-0.861524618	3.07E-05	Protein of unknown function; induced by nitric oxide; predicted ORF from Assembly 19; removed from Assembly 20; restored based on transcription data
orf19.1601	RPL3	1723.93	1895.27	1483.94	1544.74	-0.254745861	0.458126	Ribosomal protein, large subunit; induced by ciclopirox olamine treatment; genes encoding cytoplasmic ribosomal subunits are downregulated upon phagocytosis by murine macrophages; Hap43-induced gene; Spider biofilm repressed
orf19.7015	RPP0	1765.9	1894.67	1709.55	1814.07	-0.064098166	0.998825	Putative ribosomal protein; antigenic in mouse; repressed upon phagocytosis by murine macrophage; induced by Tbf1; overlaps orf19.7014; Spider biofilm repressed
orf19.5788	EFT2	1760.43	1891.81	2169.46	2268.64	0.253139116	0.432569	Elongation Factor 2 (eEF2); GTPase; essential; highly expressed; target of sordarin antifungals; antigenic in human/mouse; lacks site for regulatory phosphorylation by eEF2 kinase; GCN-regulated; higher protein amount in stationary phase

orf19.5960	NCE102	1443	1820.05	1174.56	1349.51	-0.354221808	0.254722	Non classical protein export protein; localized to plasma membrane; Hap43-induced gene; flow model biofilm induced; Spider biofilm induced
orf19.1409.1	orf19.1409.1	1698.45	1807.2	1529.15	1671.13	-0.135083226	0.921909	Ribosomal 60S subunit protein L22B; Spider biofilm repressed
orf19.7217	RPL4B	1673.94	1806.37	1600.08	1614.91	-0.121127876	0.946319	Ribosomal protein 4B; repressed upon phagocytosis by murine macrophage; Spider biofilm repressed
orf19.4450.1	orf19.4450.1	1722.35	1788.69	1315.87	1231.49	-0.452198875	0.014408	Protein conserved among the CTG-clade; 2 adjacent upstream SRE-1 elements; highly up-regulated in cecum-grown cells in a Cph2-dependent manner; Hap43-repressed; rat catheter, Spider and flow model biofilm induced
orf19.6834.1	TAR1	547.74	1783.98	1825.56	545.23	-0.007747625	0.998825	Ortholog of S. cerevisiae Tar1p; Transcript Antisense to Ribosomal RNA; encoded within the 25S rRNA gene on the opposite strand; induced by Tbf1
orf19.2192	GDH2	1705.88	1777.9	1477.36	1442.59	-0.254389482	0.405996	Putative NAD-specific glutamate dehydrogenase; fungal-specific; transcript regulated by Nrg1, Mig1, Tup1, and Gcn4; stationary phase enriched protein; flow model biofilm induced; Spider biofilm induced
orf19.6090	orf19.6090	1815.39	1770.19	1448.97	1496.4	-0.279760178	0.292199	Putative nucleolar protein with a predicted role in pre-rRNA processing and ribosome biogenesis; repressed by nitric oxide; required for flow model biofilm formation; Spider biofilm repressed
orf19.5741	ALS1	1727.93	1763.66	1896.02	2093.19	0.17059187	0.823294	Cell-surface adhesin; adhesion, virulence, immunoprotective roles; band at hyphal base; Rfg1, Ssk1, Spider biofilm induced; flow model biofilm repressed; CAI-4 strain background effects; promoter bound Bcr1, Tec1, Efg1, Ndt80, and Brg1
orf19.5653	ATP2	1628.25	1645.96	2041.8	2074.24	0.301315417	0.219074	F1 beta subunit of F1F0 ATPase complex; antigenic in human, mice; induced by ciclopirox olamine; caspofungin repressed; macrophage/pseudohyphal-induced; detected during exponential and stationary growth phases; Spider biofilm repressed

orf19.4152	CEF3	1507.33	1630.92	1456.81	1486.56	-0.099855437	0.998825	Translation elongation factor 3; antigenic in humans; predicted C-term nucleotide-binding active site; protein on surface of yeast, not hyphae; polystyrene adherence induced; higher protein amount in stationary phase; possibly essential
orf19.7504	orf19.7504	1838.17	1608.81	1449.31	1414.34	-0.263696716	0.35398	Ortholog of S. cerevisiae Rts3; a component of the protein phosphatase type 2A complex; Plc1-regulated; induced in core caspofungin response; Spider biofilm induced
orf19.3838	EFB1	1480.53	1600.26	1810.47	1882.84	0.233831358	0.541043	Translation elongation factor EF-1 beta; repressed by human whole blood or PMNs; macrophage/pseudohyphal-induced; antigenic in mouse; farnesol-downregulated; snoRNA snR18 is encoded within the EFB1 intron; Spider biofilm repressed
orf19.7417	TSA1	1503.9	1595.86	1533.34	1822.23	0.096636016	0.998825	TSA/alkyl hydroperoxide peroxidase C (AhPC) family protein; similar to thiol-dependent peroxidases of oxidative stress signaling; antigenic; hyphal surface, nucleus; yeast-form nucleus, cytoplasm; biofilm, phagocytosis, peroxide induced
orf19.7398.1	TSA1B	1503.9	1595.86	1533.34	1822.23	0.096636016	0.998825	Putative peroxidase; orf19.7398.1 is contig-truncated fragment of gene identical to TSA1; Tsa1p and Tsa1Bp role under oxidative/reductive stress, hyphal cell wall formation; in hyphal nucleus, cell wall; yeast-form nucleus, cytoplasm
orf19.3160	HSP12	1468.41	1587.03	2080.93	2078.88	0.407527055	0.043728	Heat-shock protein; induced by osmotic/oxidative/cadmium stress, fluphenazine treatment, low iron, CDR1 and CDR2 overexpression, or ssn6 or ssk1 null mutation; overexpression increases resistance to farnesol and azoles
orf19.903	GPM1	1336.3	1579.15	2315.28	2692.15	0.712865209	0.000136	Phosphoglycerate mutase; surface protein that binds host complement Factor H and FHL-1; antigenic; fluconazole, or amino acid starvation (3-AT) induced, farnesol-repressed; Hap43, flow model biofilm induced; Spider biofilm repressed
orf19.968	PGA14	1501.4	1547.8	1168.48	1302.82	-0.295298317	0.311527	Putative GPI-anchored protein; induced during cell wall regeneration; regulated by Ssn6p
orf19.1700	RPS7A	1394.52	1534.56	1114.49	1249.95	-0.30097248	0.328441	Ribosomal protein S7; genes encoding cytoplasmic ribosomal subunits, translation factors, and tRNA synthetases are downregulated upon phagocytosis by murine macrophage; Spider biofilm repressed

orf19.3335	orf19.3335	1566.98	1530.51	1633.12	1524.29	0.01309532	0.998825	Plasma membrane protein of unknown function; colony morphology-related gene regulation by Ssn6; repressed by nitric oxide
orf19.655	PHO84	1295.1	1503.03	1569.71	1750.39	0.217335614	0.685582	High-affinity phosphate transporter; transcript regulated by white-opaque switch; Hog1, ciclopirox olamine or alkaline induced; caspofungin, stress repressed; upregulated in RHE model; Spider and flow model biofilm induced, Hap43-induced
orf19.6472	CYP1	1463.67	1494.42	1936.06	2176.32	0.437151869	0.03181	Peptidyl-prolyl cis-trans isomerase; cyclosporin A sensitive activity; soluble in hyphae; biofilm induced, macrophage-induced protein; downregulated upon treatment of biofilm with farnesol; present in exponential and stationary phase cells
orf19.5818	SUR2	1145.93	1465.62	709.74	932.41	-0.616429702	0.0103	Putative ceramide hydroxylase; predicted enzyme of sphingolipid biosynthesis; regulated by Tsa1, Tsa1B under H2O2 stress conditions; Spider and flow model biofilm induced
orf19.2529.1	orf19.2529.1	976.75	1452.72	764.37	1227.13	-0.17638128	0.998825	Protein of unknown function; Spider biofilm repressed
orf19.3138	NOP1	1548.61	1448.34	1326.48	1174.51	-0.259970834	0.382965	Nucleolar protein; flucytosine induced; Hap43-induced; Spider biofilm repressed
orf19.3354	orf19.3354	1232.23	1438.87	1074.79	1243.74	-0.200995534	0.789669	Ortholog(s) have structural constituent of ribosome activity and 90S preribosome, cytosolic small ribosomal subunit, extracellular region, nucleolus localization
orf19.6002	RPL8B	1519.64	1433.58	1333.87	1357.18	-0.137916467	0.892679	Predicted ribosomal protein; regulated upon yeast-hypha switch; repressed upon phagocytosis by murine macrophage; Spider biofilm repressed
orf19.4216	orf19.4216	1322.91	1412.69	1901.77	2032.05	0.481657393	0.011405	Putative heat shock protein; decreased expression in hyphae; transcription is increased in populations of cells exposed to fluconazole over multiple generations; overexpression increases resistance to farnesol and azoles

orf19.2707.1	QCR9	1585.74	1410.15	1570.28	1744.03	0.129438849	0.932191	Putative ubiquinol cytochrome c reductase; shows colony morphology-related gene regulation by Ssn6p
orf19.822	HSP21	1186.15	1402.85	556.17	581.69	-1.112567849	6.13E-11	Small heat shock protein; role in stress response and virulence; fluconazole-downregulated; induced in cyr1 or ras1 mutant; stationary phase enriched protein; detected in some, not all, biofilm extracts; Spider biofilm induced
orf19.5213.1	COX8	1599.28	1400.71	1468.22	1675.24	0.056211242	0.998825	Putative cytochrome c oxidase; flucytosine induced; caspofungin repressed
orf19.409	orf19.409	1151.13	1392.13	628.92	780.23	-0.789920817	9.93E-05	Ortholog of S. cerevisiae Aim38/Rcf2, cytochrome c oxidase subunit; plasma membrane localized; Hap43-repressed; induced in oralpharyngeal candidasis; flow model biofilm induced; Spider biofilm repressed
orf19.4833	MLS1	1652.96	1353.61	1442.8	1070.02	-0.253815116	0.55304	Malate synthase; glyoxylate cycle enzyme; no mammalian homolog; regulated upon white-opaque switch; phagocytosis, strong oxidative stress induced; stationary phase enriched; flow model biofilm repressed; rat catheter, Spider biofilm induced
orf19.3182	GIS2	1277.06	1336.44	1224.25	1299.16	-0.059264001	0.998825	Translational activator for mRNAs with internal ribosome entry sites; induced in high iron; repressed by yeast-hypha switch; null exhibits sensitivity to sorbitol, 5-fluorocytosine, and cold temperatures; Spider biofilm repressed
orf19.3294	MBF1	1371.75	1328.85	1330.08	1419.18	0.013866906	0.998825	Putative transcriptional coactivator; caspofungin repressed
orf19.6854	ATP1	1332.78	1311.8	1698.04	1664.69	0.316602232	0.18018	ATP synthase alpha subunit; antigenic in human/mouse; at hyphal surface; ciclopirox, ketoconazole, flucytosine induced; Efg1, caspofungin repressed; may be essential; sumoylation target; stationary phase-enriched; Spider biofilm repressed
orf19.1042	POR1	1375.27	1296.53	1777.7	1722.66	0.358741779	0.082372	Mitochondrial outer membrane porin; in detergent-resistant membrane fraction (possible lipid raft component); antigenic in human, mouse; Hap43p-induced; flucytosine-, macrophage-, farnesol- induced; fluconazole, caspofungin repressed

orf19.7678	ATP16	1452.41	1292.36	1659.64	1627.01	0.236952824	0.473379	Subunit of the mitochondrial F1F0 ATP synthase; sumoylation target; protein newly produced during adaptation to the serum; Spider biofilm repressed
orf19.7566	orf19.7566	1523.91	1272.49	1473.95	1106.67	-0.1208425	0.976425	Predicted amino acid transport domain; transcript upregulated in clinical strains from HIV+ patients with oral candidiasis; alkaline upregulated by Rim101; rat catheter, Spider and flow model biofilm induced
orf19.5201.1	orf19.5201.1	1193.51	1270.48	1342.24	1547.98	0.204537751	0.72759	Has domain(s) with predicted enzyme inhibitor activity, role in negative regulation of nucleotide metabolic process and mitochondrion localization
orf19.5943.1	orf19.5943.1	1179.36	1234.78	1341.22	1441.32	0.181019158	0.789669	Protein of unknown function; transcript upregulated in an RHE model of oral candidiasis; rat catheter and Spider biofilm repressed
orf19.4565	BGL2	1109.8	1225.46	1046.79	1187.84	-0.070245481	0.998825	Cell wall 1,3-beta-glucosyltransferase; mutant has cell-wall and growth defects, but wild-type 1,3- or 1,6-beta-glucan content; antigenic; virulence role in mouse systemic infection; rat catheter biofilm induced
orf19.4980	HSP70	1170.19	1221.72	1281.24	1378.09	0.132131034	0.931333	Putative hsp70 chaperone; role in entry into host cells; heat-shock, amphotericin B, cadmium, ketoconazole-induced; surface localized in yeast and hyphae; antigenic in host; farnesol-downregulated in biofilm; Spider biofilm induced
orf19.1164	GAR1	1265.36	1211.93	1142.46	1137.07	-0.124820081	0.935403	Putative H/ACA snoRNP pseudouridylase complex protein; mutation confers hypersensitivity to tubercidin (7-deazaadenosine); macrophage/pseudohyphal-induced; Spider biofilm repressed
orf19.1069	RPN4	1168.51	1208.93	761.18	799.12	-0.582247725	0.001518	C2H2 transcription factor; putative regulator of proteasome genes; DNA recognition sequence (GAAGGCAAAA) enriched in regions upstream of proteasome genes; induced in core stress response; Hap43-induced; Spider biofilm induced
orf19.1059	HHF1	1280.41	1208.2	1216.93	1084.7	-0.118805856	0.946319	Putative histone H4; repressed in fkh2 mutant; regulated by Efg1; fluconazole induced; amphotericin B repressed; farnesol regulated; colony morphology-related gene regulation by Ssn6; Hap43-induced; rat catheter and Spider biofilm repressed

orf19.6127	LPD1	1222.76	1198.34	1047.22	1012.1	-0.23288642	0.525801	Putative dihydrolipoamide dehydrogenase; soluble in hyphae; antigenic in human oral infection and murine systemic infection; macrophage-induced protein; protein present in exponential and stationary phase yeast cultures; Hap43p-repressed
orf19.1280	SUI1	1173.07	1196.75	842.44	813.05	-0.500716782	0.007683	Putative translation initiation factor; flucytosine induced; genes encoding ribosomal subunits, translation factors, and tRNA synthetases are downregulated upon phagocytosis by murine macrophage
orf19.6247.1	orf19.6247.1	1284.41	1193.08	1037.53	1171.26	-0.163302368	0.8593	Ortholog(s) have protein channel activity and role in mitochondrial outer membrane translocase complex assembly, protein targeting to mitochondrion
orf19.3911	SAH1	1195.46	1185.78	1448.87	1619.21	0.334092572	0.19471	S-adenosyl-L-homocysteine hydrolase; sulfur amino acid metabolism; antigenic in human; alkaline-, fluconazole-induced expression; Gcn4-regulated; amino acid starvation (3-AT) repressed; flow model biofilm induced; Spider biofilm repressed
orf19.3651	PGK1	1011.27	1180.7	1782.05	2007.03	0.722048963	8.48E-05	Phosphoglycerate kinase; localizes to cell wall and cytoplasm; antigenic in murine/human infection; flow model biofilm, Hog1-, Hap43-, GCN-induced; repressed upon phagocytosis; repressed in Spider biofilms by Bcr1, Ndt80, Rob1, Brg1
orf19.1154	EGD1	1060.94	1153.5	1343.31	1609.94	0.375894268	0.160353	Putative GAL4 DNA-binding enhancer protein; soluble protein in hyphae; biofilm induced; macrophage/pseudohyphal-induced; equal level of protein in exponential and stationary growth phase yeast cultures; Spider biofilm repressed
orf19.3037	orf19.3037	1084.94	1153.36	1090.05	1124.31	-0.027079782	0.998825	Putative poly(A)-binding protein; regulated by Gcn4p; induced in response to amino acid starvation (3-AT treatment); protein present in exponential and stationary growth phase yeast cultures
orf19.6229	CAT1	1147.97	1150.11	1278.14	1348.24	0.170484357	0.823496	Catalase; resistance to oxidative stress, neutrophils, peroxide; role in virulence; regulated by iron, ciclopirox, fluconazole, carbon source, pH, Rim101, Ssn6, Hog1, Hap43, Sfu1, Sef1, farnesol, core stress response; Spider biofilm induced
orf19.4490.2	QCR8	1364.25	1146.68	1392.25	1325.4	0.097986575	0.998825	Putative ubiquinol cytochrome c reductase; macrophage and pseudohyphal-induced protein; colony morphology-related gene regulation by Ssn6; Hap43-repressed; Spider biofilm repressed

orf19.6311	orf19.6311	1232.23	1140.91	844.8	837	-0.478883632	0.010843	Protein of unknown function; Hap43-induced; rat catheter and Spider biofilm induced
orf19.6791	HHT1	1185.92	1131.64	1262.47	1182.31	0.060086352	0.998825	Putative histone H3; reduced mRNA abundance in fkh2 mutant; farnesol regulated; Spider biofilm induced; rat catheter biofilm repressed
orf19.1750	SLR1	1015.79	1129.61	949.51	1139.38	-0.04645781	0.998825	Protein similar to mammalian SR-like RNA splicing factor; involved in filamentous growth and virulence; gene has intron; Spider biofilm repressed
orf19.2048	orf19.2048	1109	1124.02	565.38	588.71	-0.902992252	3.36E-08	Proten of unknown function; transcript positively regulated by Sfu1; Hap43 repressed; Spider biofilm induced
orf19.1471	COX4	1139.39	1118.23	1183.52	1181.7	0.052707096	0.998825	Putative cytochrome c oxidase subunit IV; Mig1-regulated; macrophage/pseudohyphal-induced gene; macrophage-induced protein; repressed by nitric oxide; 5'-UTR intron; Hap43-repressed
orf19.6688	orf19.6688	1115.45	1113.51	132.77	112.19	-2.924805451	1.99E-69	Protein of unknown function; expression decreases by benomyl treatment or in an azole-resistant strain overexpressing MDR1; Spider biofilm induced
orf19.4716	GDH3	1291.29	1101.46	979.51	866.63	-0.361919834	0.120567	NADP-glutamate dehydrogenase; Nrg1, Plc1 regulated; hypha, hypoxia, Efg1-repressed; Rim101-induced at pH 8; GlcNAc, ciclopirox, ketoconazole induced; exp and stationary phase protein; Spider biofilm repressed; rat catheter biofilm induced
orf19.3668	HGT2	951.58	1097.34	651.25	593.43	-0.684956267	0.000265	Putative MFS glucose transporter; 20 member C. albicans glucose transporter family; 12 probable membrane-spanning segments; expressed in rich medium with 2% glucose; rat catheter and Spider biofilm induced
orf19.670	SMT3	1049.54	1094.42	992.92	1169.68	0.002198821	0.998825	SUMO, small ubiquitin-like protein; Smt3p-conjugated proteins localize to septation site and mother side of bud neck; C. albicans septins appear not to be Smt3p-modified, in contrast to S. cerevisiae septins

orf19.3010.1	ECM33	956.97	1081.17	1455.81	1638.81	0.549681215	0.006439	GPI-anchored cell wall protein; mutants show cell-wall defects and reduced adhesion, host cell damage, and endocytosis; mutant infection is immunoprotective in murine model; fluconazole-induced; caspofungin repressed
orf19.7238	NPL3	1108.23	1075.89	1096.47	1084.07	-0.014027141	0.998825	Putative RNA-binding protein; required for normal flow model biofilm growth; nuclear export is facilitated by Hmt1p; transcript upregulated in RHE model of oralcandidiasis; Spider biofilm repressed
orf19.5893	RIP1	1117.83	1070.04	1201.89	1187.54	0.109143342	0.974215	Putative ubiquinol cytochrome c-reductase; induced by high iron; Hap43, nitric oxide, alkaline repressed; Spider biofilm repressed
orf19.1854	HHF22	1126.88	1069.97	1035.37	968.56	-0.13733653	0.900811	Putative histone H4; regulated by Efg1; flucytosine, fluconazole-induced; amphotericin B, caspofungin repressed; colony morphology-related gene regulation by Ssn6; Hap43-induced; rat catheter and Spider biofilm repressed
orf19.6844	ICL1	1279.15	1068.06	751.17	514.54	-0.830902103	2.11E-05	Isocitrate lyase; glyoxylate cycle enzyme; required for virulence in mice; induced upon phagocytosis by macrophage; farnesol regulated; Pex5-dependent peroxisomal localization; stationary phase enriched; rat catheter, Spider biofilm induced
orf19.6852.1	orf19.6852.1	892.38	1061.92	931.47	967.73	-0.052537086	0.998825	Protein of unknown function; Spider biofilm induced
orf19.6402	CYS3	974.46	1059.2	798.79	794.19	-0.342831937	0.196416	Cystathionine gamma-lyase; induced by alkaline, amphotericin B, cadmium stress, oxidative stress via Cap1; possibly adherence-induced; Hog1 regulated; reduced levels in stationary phase yeast cells; Spider and flow model biofilm induced
orf19.5351	TIF11	1078.11	1059.08	952.23	1051.03	-0.097375937	0.998825	Translation initiation factor eIF1a; possibly transcriptionally regulated upon hyphal formation; genes encoding ribosomal subunits, translation factors, and tRNA synthetases are downregulated upon phagocytosis by murine macrophage
orf19.6000	CDR1	953.28	1052.4	285.52	236.18	-1.801551654	3.60E-27	Multidrug transporter of ABC superfamily; transports phospholipids in an in-to-out direction; induced by beta-estradiol, progesterone, corticosteroid, or cholesterol; Spider biofilm induced

orf19.227	COX7	1081.55	1051.48	1092.83	1141.6	0.052948565	0.998825	Putative cytochrome c oxidase; flucytosine induced; repressed by nitric oxide
orf19.6928	SAP9	826.82	1038.71	818.25	913.5	-0.113353572	0.998825	Secreted aspartyl protease; roles in adhesion, cell surface integrity; induced by antifungal drugs, stationary phase, or in white-phase cells; farnesol-downregulated in biofilm; autocatalytic processing; GPI-anchor; Spider biofilm induced
orf19.6081	PHR2	918	1033.15	906.58	949.2	-0.080363694	0.998825	Glycosidase; role in vaginal not systemic infection (low pH not neutral); low pH, high iron, fluconazole, Hap43-induced; Rim101-repressed at pH8; rat catheter biofilm induced; Bcr1-repressed in RPMI a/a biofilms
orf19.7043.1	ACB1	1073.05	1020.96	1480.34	1869.45	0.621856735	0.00169	Protein similar to a region of acyl-coenzyme-A-binding protein; amphotericin B repressed; regulated by Nrg1, Tup1; Hap43-induced
orf19.2531	CSP37	1019.08	1014.61	1155.35	1259.04	0.221867342	0.608044	Hyphal cell wall protein; role in progression of mouse systemic infection; predicted P-loop, divalent cation binding, N-glycosylation sites; expressed in yeast and hyphae; hyphal downregulated; stationary-phase enriched; GlcNAc-induced
orf19.18	IMH3	964.39	1004.43	862.49	924.49	-0.142432497	0.916335	Inosine monophosphate (IMP) dehydrogenase; enzyme of GMP biosynthesis; target of mycophenolic acid and mizoribine monophosphate; antigenic during infection; repressed in core stress response; snoRNA snR54 encoded within IMH3 intron
orf19.4688	DAG7	785.8	1001.83	557.29	608.62	-0.577603199	0.01199	Secretory protein; a-specific, alpha-factor induced; mutation confers hypersensitivity to toxic ergosterol analog; fluconazole-induced; induced during chlamydospore formation in C. albicans and C. dubliniensis
orf19.7569	SIK1	1063.53	989.87	875.22	912.11	-0.198736073	0.710493	Putative U3 snoRNP protein; Hap43p-induced gene; physically interacts with TAP-tagged Nop1p
orf19.6385	ACO1	920.73	974.73	1076.45	973.01	0.090531243	0.998825	Aconitase; induced in high iron; 2 upstream CCAAT motifs; amino acid starvation (3-AT), amphotericin B, phagocytosis, farnesol induced; Hap43, fluconazole-repressed; Gcn4-regulated; antigenic in infection; flow and Spider biofilm repressed

orf19.1770	CYC1	1054.71	973.55	913.29	1011.09	-0.080381282	0.998825	Cytochrome c; complements defects of S. cerevisiae cyc1 cyc7 double mutant; induced in high iron; alkaline repressed; repressed by nitric oxide; Hap43-dependent repression in low iron; regulated by Sef1, Sfu1
orf19.6146	CLG1	835.79	970.53	680.19	745.29	-0.329680565	0.29737	Putative cyclin-like protein; transcription is regulated upon yeast-hyphal switch
orf19.5007	ACT1	978.59	966.95	1331.14	1397.51	0.448995895	0.021894	Actin; gene has intron; transcript regulated by growth phase, starvation; at polarized growth site in budding and hyphal cells; required for wild-type Cdc42 localization; unprocessed N terminus; Hap43-induced; Spider biofilm repressed
orf19.2551	MET6	906.05	965.98	1406.88	1416.49	0.544740634	0.002804	Essential 5-methyltetrahydropteroyltriglutamate-homocysteine methyltransferase (cobalamin-independent methionine synthase); antigenic in murine/human systemic infection; heat shock, estrogen, GCN-induced; Spider biofilm repressed
orf19.3932	orf19.3932	1037.57	963.76	1178.55	1013.68	0.110758845	0.976613	Predicted RNA binding protein; stationary phase enriched; induced in core caspofungin response; induced by nitric oxide independent of Yhb1; repressed in ssr1 null; ketoconazole, hypoxia induced; Spider biofilm induced
orf19.7509.1	ATP17	938.99	957.95	1110.48	1088.02	0.187566099	0.748714	Mitochondrial ATPase complex subunit; downregulated by Efg1p; flucytosine induced; caspofungin repressed
orf19.986	GLY1	1061.03	956.38	953.33	929.83	-0.103807331	0.998825	L-threonine aldolase; complements glycine auxotrophy of S. cerevisiae shm1 shm2 gly1-1 triple mutant; macrophage/pseudohyphal-induced; the GLY1 locus has an RFLP and is triploid in strain SGY269; flow model biofilm induced
orf19.740	HAP41	687.7	954.83	459.5	632.39	-0.425936908	0.608044	Putative Hap4-like transcription factor; Hap43-repressed; not required for response to low iron; induced by Mnl1 under weak acid stress; Spider biofilm induced
orf19.4949	orf19.4949	1129.64	952.25	713.72	876.95	-0.363442465	0.213617	Ortholog of C. parapsilosis CDC317 : CPAR2_801735, C. dubliniensis CD36 : Cd36_84090, Candida orthopsilosis Co 90-125 : CORT_0B03960 and Spathaspora passalidarum NRRL Y-27907 : SPAPADRAFT_141253

orf19.6745	TPI1	880.64	949.08	1357.7	1448.03	0.566081165	0.002365	Triose-phosphate isomerase; antigenic in mouse/human; mutation affects filamentation; macrophage-repressed; protein in exponential and stationary growth phase yeast; possibly essential; flow model biofilm induced; Spider biofilm repressed
orf19.251	GLX3	1047.46	949.08	657.03	656.44	-0.577278454	0.001554	Glutathione-independent glyoxalase; binds human immunoglobulin E; alkaline, fluconazole, Hog1 repressed; hypoxia, oxidative stress via Cap1, Hap43 induced; stationary-phase enriched; rat catheter, Spider biofilm induced
orf19.7046	MET28	1003.66	924.77	1108.04	1307.37	0.295083124	0.365475	Predicted bZIP domain-containing transcription factor; protein induced during the mating process; possibly essential, disruptants not obtained by UAU1 method; Hap43-repressed; rat catheter biofilm induced
orf19.2020	HGT6	597.52	911.5	813.42	1082.27	0.208004521	0.998825	Putative high-affinity MFS glucose transporter; 20 family members; induced in core stress response; fluconazole, oralpharyngeal candidasis induced; flow model biofilm induced; Spider biofilm induced
orf19.842	ASR3	871.95	906.34	1121.34	1117.27	0.299581814	0.282737	Adenylyl cyclase and stress responsive protein; induced in cyr1 or ras1 mutant; Spider biofilm induced
orf19.6452	RBP1	819.46	901.19	998.04	1222.08	0.329717606	0.312123	Peptidyl-prolyl cis-trans isomerase; rapamycin-binding protein; homozygous null mutation confers rapamycin resistance; regulated by Gcn4p; macrophage-induced protein; repressed in response to 3-AT; functional homolog of S. cerevisiae Rbp1p
orf19.1896	SSC1	861.17	898.8	928.02	985.22	0.101017399	0.998825	Heat shock protein; at yeast-form cell surface, not hyphae; antigenic; Gcn4-regulated; induced by amino acid starvation (3-AT) or by adherence to polystyrene; macrophage-repressed; sumoylation target; possibly essential
orf19.5006.1	orf19.5006.1	915.69	889.91	756.09	786.52	-0.223557616	0.608898	Ortholog(s) have SNAP receptor activity and role in Golgi to plasma membrane transport, cytokinesis, endocytosis, exocytosis, vesicle fusion
orf19.550	PDX3	845.84	887.32	623.6	632.65	-0.445033576	0.042294	Pyridoxamine-phosphate oxidase; transcript regulated by yeast-hypha switch and by Nrg1, Mig1, Tup1; Hap43, caspofungin repressed; present in exponential and stationary phase yeast cultures

orf19.2066.1	ATP18	969.62	884.69	1075.35	1071.4	0.187969536	0.742982	F1F0 ATP synthase complex subunit; gene has intron
orf19.6529	CDC34	759.47	883.99	527.61	605.39	-0.50443268	0.033604	Putative ubiquitin-protein ligase; transcript regulated by Nrg1 and Tup1, and by Gcn2 and Gcn4; rat catheter biofilm induced
orf19.526	NHP2	886.09	883.72	705.66	744.09	-0.280132806	0.39542	Putative H/ACA snoRNP protein; macrophage/pseudohyphal-induced
orf19.5660.1	orf19.5660.1	1044.26	883.57	975.86	948.12	-0.012032927	0.998825	Ortholog(s) have proton-transporting ATPase activity, rotational mechanism, structural molecule activity and role in ATP synthesis coupled proton transport, cristae formation, protein complex oligomerization
orf19.3414	SUR7	807	882.73	794.57	798.84	-0.092125674	0.998825	Protein required for normal cell wall, plasma membrane, cytoskeletal organization, endocytosis; localizes to eisosome subdomains of plasma membrane; four transmembrane motifs; mutant shows ectopic, chitin-rich cell wall; fluconazole-induced
orf19.2568	IFU5	780.55	875.04	828.41	830.15	-0.012193409	0.998825	Predicted membrane protein; estradiol-induced; upregulation associated with CDR1 and CDR2 overexpression or fluphenazine; putative drug-responsive regulatory site; similar to S. cerevisiae Wwm1p; Hap43-repressed; Spider biofilm repressed
orf19.941	SEC14	862.84	874.01	591.67	600.13	-0.519606222	0.008955	Essential protein; functional homolog of S. cerevisiae Sec14p, a Golgi phosphatidylinositol/phosphatidylcholine transfer protein that regulates choline-phosphate cytidyltransferase and thereby affects secretion; biofilm-regulated
orf19.1152	orf19.1152	821.22	873.63	949.17	936.58	0.131224361	0.938642	Protein of unknown function; induced in core stress response; Gcn2 and Gcn4 regulated; flow model biofilm induced; Spider biofilm induced
orf19.6887	orf19.6887	863.72	859.74	876.05	965.93	0.079300281	0.998825	Predicted ORF from Assembly 19; repressed by nitric oxide; removed from Assembly 20; subsequently reinstated in Assembly 21 based on comparative genome analysis

orf19.5949	FAS2	807.52	854.06	928.26	903.1	0.117702322	0.964275	Alpha subunit of fatty-acid synthase; required for virulence in mouse systemic infection and rat oropharyngeal infection models; regulated by Efg1; fluconazole-induced; amphotericin B repressed; flow model and Spider biofilm repressed
orf19.4921.1	orf19.4921.1	732.85	853.75	215.05	264.11	-1.571424762	3.54E-17	Protein of unknown function; Spider biofilm repressed
orf19.4885	MIR1	875.65	848.32	896.78	856.13	0.010599958	0.998825	Putative mitochondrial phosphate transporter; caspofungin repressed; expression is increased in a fluconazole-resistant isolate; induced upon adherence to polystyrene
orf19.7611	TRX1	860.28	844.16	869.79	952.75	0.081252837	0.998825	Thioredoxin; involved in response to reactive oxygen species; biofilm, benomyl, flucytosine, peroxide, Hap43 induced; amphotericin B, caspofungin repressed; induced by human neutrophils; macrophage-repressed gene
orf19.6937	PTR22	923.93	838.03	598.61	539.51	-0.600891121	0.001128	Oligopeptide transporter involved in uptake of di-/tripeptides; regulated by Stp2 and Stp3; transcript induced upon phagocytosis by macrophage; repressed by Rim101 at pH 8; flow model biofilm induced
orf19.3324	TIF	774.32	833.36	882.15	904.26	0.129705353	0.946319	Translation initiation factor; upregulated in highly virulent strain compared to less virulent strain; antigenic in human; flucytosine induced; downregulated upon phagocytosis by macrophages; Spider biofilm repressed
orf19.1051	HTA2	893.88	831.45	901.56	718.35	-0.098827553	0.998825	Putative histone H2A; farnesol regulated; rat catheter biofilm repressed; Spider biofilm repressed; Hap43-induced
orf19.589	VPS21	859.36	825.06	647.73	647.41	-0.366162846	0.132203	Late endosomal Rab small monomeric GTPase involved in transport of endocytosed proteins to the vacuole; involved in filamentous growth and virulence; Spider biofilm induced
orf19.1224	FRP3	993.73	821.14	965.03	802.29	-0.047450528	0.998825	Putative ammonium transporter; upregulated in the presence of human neutrophils; fluconazole-downregulated; repressed by nitric oxide; Spider biofilm induced; rat catheter biofilm repressed

orf19.3646	CTR1	769.64	820.6	870.06	897.83	0.129846697	0.946319	Copper transporter; transcribed in low copper; induced Mac1, Tye7, macrophage interaction, alkaline pH via Rim101; 17-beta-estradiol repressed; complements S. cerevisiae ctr1 ctr3 copper transport mutant; flow model/Spider biofilm induced
orf19.5876	orf19.5876	850.97	814	789.26	788.36	-0.084585973	0.998825	Protein of unknown function; Cyr1-repressed; induced by alpha pheromone in SpiderM medium; rat catheter and Spider biofilm induced
orf19.5750	SHM2	912.98	812.08	1077.49	1056.69	0.279111553	0.34692	Cytoplasmic serine hydroxymethyltransferase; complements glycine auxotrophy of S. cerevisiae shm1 shm2 gly1-1 mutant; antigenic; farnesol-upregulated in biofilm; stationary-phase enriched protein; rat catheter and Spider biofilm repressed
orf19.5629	QCR7	782.59	810.51	794.04	924.88	0.091335268	0.998825	Putative ubiquinol-cytochrome-c reductase, subunit 7; Hap43p-repressed gene
orf19.873.1	COX6	899.43	810.19	841.79	854.85	-0.019177811	0.998825	Putative cytochrome c oxidase; flucytosine induced
orf19.7592	FAA4	972.63	803.36	867.14	746.87	-0.139659873	0.928284	Predicted acyl CoA synthase
orf19.1061	HHT21	909.22	800.79	868.38	691.64	-0.136672776	0.938642	Putative histone H3; amphotericin B repressed; regulated by Efg1, farnesol; Hap43-induced; rat catheter and Spider biofilm repressed
orf19.118	FAD2	866.53	799.79	734.65	706	-0.208313741	0.675986	Delta-12 fatty acid desaturase, involved in production of linoleic acid, which is a major component of membranes
orf19.5813	orf19.5813	676.02	797.63	417.17	461.4	-0.69695149	0.000854	Putative adhesin-like protein; upregulated during growth in the mouse cecum; flow model, rat catheter and Spider biofilm induced

orf19.6877	PNG2	660.69	797.5	681.43	705.02	-0.08179006	0.998825	Putative peptide:N-glycanase; gene has variable numbers of 12-bp repeats; induced by caspofungin, ciclopirox olamine, ketoconazole or hypoxia; gene of core caspofungin response; Hap43-induced; Spider biofilm induced
orf19.596.1	NOP10	766.35	795.1	749.19	727.42	-0.088571825	0.998825	Small nucleolar ribonucleoprotein; flucytosine induced
orf19.2762	AHP1	736.4	793.04	1212.8	1307.76	0.662534446	0.000253	Alkyl hydroperoxide reductase; immunogenic; fluconazole-induced; amphotericin B, caspofungin, alkaline repressed; core stress response induced; Ssk1/Nrg1/Tup1/Ssn6/Hog1 regulated; flow model biofilm induced; rat catheter biofilm repressed
orf19.1853	HHT2	904.38	779.59	780.84	688.83	-0.194727098	0.752654	Putative histone H3; farnesol regulated; Hap43-induced; rat catheter and Spider biofilm repressed
orf19.3527	CYT1	778.63	761.07	822.14	868.84	0.115932665	0.976613	Cytochrome c1; induced in high iron; alkaline repressed; possibly an essential gene, disruptants not obtained by UAU1 method; Hap43-repressed; Spider biofilm repressed
orf19.5419	ATP5	857.48	756.93	976.36	934.16	0.218138618	0.612468	Putative F0-ATP synthase FO subunit B; caspofungin repressed; protein level decreased in stationary phase yeast cultures; Spider biofilm repressed
orf19.3171	ACH1	722.85	753.05	1064.45	1013.29	0.449260678	0.028096	Acetyl-coA hydrolase; acetate utilization; nonessential; soluble protein in hyphae; antigenic in human; induced on polystyrene adherence; farnesol-, ketoconazole-induced; no human or murine homolog; stationary phase-enriched protein
orf19.6059	TTR1	767.62	752.75	654.19	784.76	-0.082144022	0.998825	Putative glutaredoxin; described as a glutathione reductase; induced by human neutrophils and benomyl treatment; alkaline repressed; regulated by Gcn2 and Gcn4; required for virulence in mouse model; Spider biofilm induced
orf19.5491.1	ATP14	789.69	750.06	859.85	909.25	0.177516427	0.823496	Putative mitochondrial F1F0 ATP synthase subunit; macrophage/pseudohyphal-induced

orf19.7107	orf19.7107	765.63	749.56	569.91	553.04	-0.416373988	0.06565	Ortholog(s) have role in ribosomal large subunit biogenesis and cytoplasm, nucleus, ribosome localization
orf19.7509.2	LSM6	698.23	747.15	728.77	896.5	0.147247518	0.938642	Putative Lsm protein; flucytosine induced
orf19.4826	IDH1	751.08	744.29	826.96	895.18	0.180329725	0.824505	Putative mitochondrial NAD-isocitrate dehydrogenase subunit 1; soluble protein in hyphae; protein level decrease in stationary phase cultures
orf19.2942	DIP5	911.76	741.81	1030.29	817.67	0.136850625	0.942804	Dicarboxylic amino acid permease; mutation confers hypersensitivity to toxic ergosterol analog; induced upon phagocytosis by macrophage; Gcn4-regulated; upregulated by Rim101 at pH 8; rat catheter and Spider biofilm induced
orf19.3712	orf19.3712	640.71	741.44	556.65	631.67	-0.211350077	0.788835	Protein of unknown function; induced by Mnl1 under weak acid stress; flow model biofilm induced; Spider biofilm induced
orf19.88	ILV5	716.62	737.54	970.75	1080.37	0.451546486	0.040181	Ketol-acid reductoisomerase; antigenic; regulated by Gcn4; GlcNAc, amino acid starvation (3-AT)-induced; macrophage-repressed protein; protein present in exponential and stationary phase; flow model and Spider biofilm repressed
orf19.3669	SHA3	583.91	735.46	388.4	494.44	-0.532045533	0.051679	Putative ser/thr kinase involved in glucose transport; Tn mutation affects filamentous growth; fluconazole-induced; ketoconazole-repressed; induced in by alpha pheromone in SpiderM; possibly essential; flow model biofilm induced
orf19.1631	ERG6	747.8	735.09	514.47	611.54	-0.375091789	0.193129	Delta(24)-sterol C-methyltransferase, converts zymosterol to fecosterol, ergosterol biosynthesis; mutation confers nystatin resistance; Hap43, GlcNAc-, fluconazole-induced; upregulated in azole-resistant strain; Spider biofilm repressed
orf19.6925	HTB1	791.74	729.99	761.33	566.5	-0.196864987	0.811201	Histone H2B; induced by adherence to polystyrene; amphotericin B, caspofungin repressed; flucytosine, fluconazole induced; Efg1 regulated; slow growth, increased white-to opaque switch in ectopic expression strains; Spider biofilm repressed

orf19.3061.1	orf19.3061.1	661.45	729.95	649.56	752.25	-0.000513618	0.998825	Ortholog of S. cerevisiae Rps22Ap and Rps22Bp; gene contains 5' UTR intron
orf19.93	orf19.93	743.67	729.12	563.37	548.43	-0.391793745	0.100993	Putative mitochondrial intermembrane space protein; colony morphology-related gene regulation by Ssn6; regulated by Sef1, Sfu1, and Hap43; Spider biofilm repressed; rat catheter biofilm induced
orf19.677	CHO1	705.47	726.95	1597.01	1599.91	1.079176877	6.51E-13	Putative phosphatidylserine synthase; ortholog of S. cerevisiae CHO1; transposon mutation affects filamentous growth; regulated by Nrg1, Tup1
orf19.3822	SCS7	669.75	723.49	529.29	560.8	-0.339851191	0.257292	Putative ceramide hydroxylase; regulated by Nrg1; induced in high iron; fluconazole-induced; Hap43-repressed; Spider biofilm induced
orf19.7049	CYB5	755.15	722.74	721.06	797.4	0.026656687	0.998825	Cytochrome b(5); ortholog of S. cerevisiae Cyb5; induced in high iron; fluconazole-induced; shows colony morphology-related gene regulation by Ssn6; mutants are viable
orf19.5642	orf19.5642	732.99	722.63	841.34	840.45	0.182828148	0.795378	Ortholog of S. cerevisiae Dif1; which regulates nuclear localization of Rnr2 and Rnr4; colony morphology-related gene regulation by Ssn6; induced in cyr1 mutant; Spider biofilm induced
orf19.171	DBP2	754.16	722.11	589.25	585.89	-0.318526245	0.266191	Putative DEAD-box family ATP-dependent RNA helicase; flucytosine induced; repressed in core stress response
orf19.913.2	orf19.913.2	759.96	722.07	810.07	857.42	0.148960851	0.893138	Ortholog of subunit 6 of the ubiquinol cytochrome-c reductase complex, a component of the mitochondrial inner membrane electron transport chain; Hap43-repressed gene
orf19.979	FAS1	662.28	716.94	732.3	727.44	0.062493124	0.998825	Beta subunit of fatty-acid synthase; multifunctional enzyme; Hap43, fluconazole-induced; amphotericin B, caspofungin repressed; macrophage/pseudohyphal-induced; flow model and Spider biofilm repressed

orf19.5885	orf19.5885	744.61	708.52	678.49	663.97	-0.119498853	0.960406	Putative U3 snoRNP protein; flucytosine induced
orf19.4813	GUA1	748.23	708.04	701.27	653.79	-0.109086434	0.998825	Putative GMP synthase, involved in the final step of guanine biosynthesis; soluble protein in hyphae; flucytosine induced; macrophage-downregulated protein abundance; protein level decreases in stationary phase cultures
orf19.4759	COX5	728	701.38	821.42	809.24	0.166715663	0.853836	Cytochrome oxidase subunit V; putative upstream CCAAT box regulatory element; macrophage/pseudohyphal-induced; repressed by nitric oxide; intron in 5'-UTR; Hap43p-dependent repression in low iron medium
orf19.5006	GCV3	762.99	698.85	822.84	778.4	0.111790915	0.98704	Glycine decarboxylase, subunit H; protein level decrease in stationary phase cultures
orf19.6114	orf19.6114	665.96	693.37	844.17	873.96	0.30402765	0.314254	Protein of unknown function; transcript detected on high-resolution tiling arrays
orf19.4311	YNK1	733.71	692.77	919.96	885.85	0.307837502	0.26523	Nucleoside diphosphate kinase (NDP kinase); homo-hexameric; soluble protein in hyphae; flucytosine induced; biofilm induced; macrophage-induced protein; stationary phase enriched protein; Spider biofilm repressed
orf19.2662	orf19.2662	753.37	685.82	1073.9	1157.94	0.58469634	0.001554	Predicted ORF of unknown function; overlaps TLO34/orf19.2661
orf19.2030	orf19.2030	672.01	683.39	630.47	646.71	-0.091320104	0.998825	Plasma membrane-associated protein; induced in cyr1 or ras1 mutant; induced by hypoxia, ketoconazole and during growth in the mouse cecum; induced in oralpharyngeal candidasis; Spider biofilm induced
orf19.6599.1	orf19.6599.1	815.26	681.23	781.14	699.83	-0.023888759	0.998825	Ortholog(s) have role in cytoplasmic translation and cytoplasm, nucleus localization

orf19.3097	PDA1	692.25	680.97	1111.45	964.22	0.545037572	0.003617	Putative pyruvate dehydrogenase alpha chain; fluconazole-induced; protein present in exponential and stationary growth phase yeast cultures; Hap43-induced; Spider biofilm repressed
orf19.5437	RHR2	519.48	680.33	264.14	354.75	-0.851920245	0.000345	Glycerol 3-phosphatase; roles in osmotic tolerance, glycerol accumulation in response to salt; Spider/flow model biofilm induced; regulated by macrophage, stress, yeast-hyphal switch, pheromone, Gcn4, Hog1, Nrg1, Tup1
orf19.1911	PGA52	686.76	674.01	644.89	628.54	-0.100891062	0.998825	GPI-anchored cell surface protein of unknown function; Hap43p-repressed gene; fluconazole-induced; possibly an essential gene, disruptants not obtained by UAU1 method
orf19.1389	orf19.1389	647.48	668.76	886.3	957.4	0.442120024	0.047791	Ortholog(s) have poly(A) RNA binding activity, role in mRNA metabolic process and cytoplasm localization
orf19.7124	RVS161	704.94	666.85	668.65	701.98	-0.010933692	0.998825	Protein required for endocytosis; contains a BAR domain, which is found in proteins involved in membrane curvature; null mutant exhibits defects in hyphal growth, virulence, cell wall integrity, and actin patch localization
orf19.1358	GCN4	594.55	659.19	645.45	650.36	0.029642749	0.998825	bZIP transcription factor; amino acid control response; required for Efg1-dependent pseudohyphal induction by amino acid starvation, not by serum; human whole blood and PMN induced; required for biofilm formation; Spider biofilm induced
orf19.6645	HMO1	699.2	658.09	628.33	579.3	-0.169879802	0.857977	HMG-box transcription factor; binds upstream of hexose and ergosterol metabolism and cell cycle genes; activates pseudohyphal growth when expressed in S. cerevisiae; repressed in hyphae; Spider biofilm repressed
orf19.4118	CNT	606.22	655.11	538.28	620.08	-0.123789362	0.993179	CNT family H(+)/nucleoside symporter; transports adenosine, uridine, inosine, guanosine, tubercidin; variant alleles for high/low-affinity isoforms; S or G at residue 328 affects specificity; Spider, flow model biofilm induced
orf19.7501	NAP1	653.18	651.37	525.27	532.85	-0.291965499	0.392322	Nucleosome assembly protein; mutants show constitutive filamentous growth; present in exponential and stationary growth phase yeast cultures

orf19.2439.1	orf19.2439.1	746.21	647.39	759.54	788.02	0.133611742	0.938642	Ortholog(s) have ubiquinol-cytochrome-c reductase activity, role in aerobic respiration, mitochondrial electron transport, ubiquinol to cytochrome c and mitochondrial respiratory chain complex III localization
orf19.1064	ACS2	603.92	647.33	771.43	808.84	0.301215584	0.357392	Acetyl-CoA synthetase; antigenic during human and murine infection; induced by Efg1; macrophage-induced protein; soluble protein in hyphae; gene contains intron; flow model and Spider biofilm repressed
orf19.4947	orf19.4947	680.42	643.37	633.08	622.44	-0.082160842	0.998825	Mitochondrial membrane protein of unknown function; Spider biofilm induced
orf19.778	PIL1	606.36	643.35	700.59	734.37	0.174200569	0.858075	Eisosome component; predicted role in endocytosis; echinocandin-binding protein; localizes to cell surface of hyphae, but not yeast-form cells; Hap43, YNB biofilm induced; rat catheter biofilm repressed
orf19.220	PIR1	543.42	640.51	258.35	280.95	-1.040925855	1.29E-07	1,3-beta-glucan-linked cell wall protein; N-mannosylated, O-glycosylated by Pmt1; cell wall defect in het mutant; Hog1/fluconazole/hypoxia induced; iron/Efg1/Plc1/temp regulated; flow model biofilm induced; hyphal, Spider biofilm repressed
orf19.7392	DED1	521.12	637.5	391.16	435.66	-0.453840641	0.106451	Predicted ATP-dependent RNA helicase; RNA strand annealing activity; Spider biofilm induced
orf19.2311	RPL82	544.66	633.58	506.19	543.9	-0.166457649	0.894293	Predicted ribosomal protein; genes encoding cytoplasmic ribosomal subunits, translation factors, and tRNA synthetases are downregulated upon phagocytosis by murine macrophage
orf19.6993	GAP2	820.38	630.68	601.14	339.21	-0.450833477	0.565541	General amino acid permease; ketoconazole, flucytosine repressed; Ssy1-dependent histidine induction; regulated by Nrg1, Tup1; colony morphology-related gene regulation by Ssn6; Spider and flow model biofilm induced
orf19.1652	POX1-3	613.52	626.14	652.28	700.1	0.105076902	0.998825	Predicted acyl-CoA oxidase; farnesol regulated; stationary phase enriched protein; Spider biofilm induced

orf19.4527	HGT1	457.34	626	734.2	869.05	0.494076011	0.082807	High-affinity MFS glucose transporter; induced by progesterone, chloramphenicol, benomyl; likely essential for growth; protein newly produced during adaptation to the serum; rat catheter and Spider biofilm induced
orf19.7520	POT1	674.71	623.34	853.75	878.44	0.379777405	0.10978	Putative peroxisomal 3-oxoacyl CoA thiolase; transcript regulated by Nrg1 and Mig1; farnesol regulated; Hap43-repressed
orf19.4618	FBA1	552.2	621.63	976.4	1018.97	0.697691184	0.000188	Fructose-bisphosphate aldolase; glycolytic enzyme; antigenic in murine/human infection; regulated by yeast-hypha switch; induced by Efg1, Gcn4, Hog1, fluconazole; phagocytosis-repressed; flow model biofilm induced; Spider biofilm repressed
orf19.5493	GSP1	673.48	621.31	674.35	677.29	0.047853465	0.998825	Small RAN G-protein; essential; no prenylation predicted; can rescue S. cerevisiae gsp1 viability; macrophage/pseudohyphal-induced; transcript not regulated by white-opaque, yeast-hypha switching; GlcNAc-induced; Spider biofilm repressed
orf19.5614	orf19.5614	648.04	621.05	1149.88	1059.36	0.739464771	7.85E-06	Putative ribonuclease H1; possibly an essential gene, disruptants not obtained by UAU1 method; flow model biofilm induced; Spider biofilm induced
orf19.7111.1	SOD3	663.82	621.02	485.06	482.34	-0.392116694	0.116527	Cytosolic manganese-containing superoxide dismutase; protects against oxidative stress; repressed by ciclopirox olamine, induced during stationary phase when SOD1 expression is low; Hap43-repressed; Spider and flow model biofilm induced
orf19.4056	BRG1	539.68	612.56	518.13	605.85	-0.044823544	0.998825	Transcription factor; recruits Hda1 to hypha-specific promoters; Tn mutation affects filamentation; Hap43-repressed; Spider and flow model biofilm induced; required for Spider biofilm formation; Bcr1-repressed in RPMI a/a biofilms
orf19.3014	BMH1	623.74	612.5	820.58	846.2	0.391454927	0.098488	Sole 14-3-3 protein in C. albicans; role in hyphal growth; possibly regulated by host interaction; localizes to yeast-form cell surface, not hyphae; alternatively spliced 5' UTR intron; Spider biofilm repressed
orf19.3579	ATP4	682.45	607.43	786.79	760.83	0.236977033	0.568801	Putative F0-ATP synthase subunit 4; macrophage/pseudohyphal-induced; present in exponential and stationary growth phases

orf19.6126	KGD2	648.01	601.57	558.42	519.52	-0.210529047	0.71162	Putative dihydrolipoamide S-succinyltransferase; induced in high iron; Hap43-repressed; Spider biofilm repressed
orf19.3223	ATP3	638.13	599.59	706.68	696.73	0.158766553	0.87796	F1-ATP synthase complex subunit; caspofungin repressed; flucytosine and macrophage/pseudohyphal-induced; present in exponential and stationary growth phases; Hap43p-induced gene
orf19.5417	DOT5	549.74	598.34	555.46	606.01	0.003308099	0.998825	Putative nuclear thiol peroxidase; alkaline downregulated; sumoylation target; Spider and flow model biofilm induced
orf19.4016	orf19.4016	608.14	597.02	646.04	661.67	0.099265086	0.998825	Putative ubiquinol-cytochrome-c reductase; amphotericin B induced; repressed by nitric oxide; protein level decreases in stationary phase cultures; Hap43-repressed; Spider biofilm repressed
orf19.7030	SSR1	565.52	596.83	520.37	543.95	-0.130650443	0.957711	Beta-glucan associated ser/thr rich cell-wall protein with a role in cell wall structure; GPI anchor; similar mRNA abundance in yeast-form and germ tubes; detected at germ tube plasma membrane; repressed in cells treated with Congo Red
orf19.5064.1	orf19.5064.1	740.04	596.47	779.89	787.64	0.207393691	0.732635	Ortholog of C. dubliniensis CD36 : Cd36_07450, C. parapsilosis CDC317 : CPAR2_208450, Candida tenuis NRRL Y-1498 : CANTEDRAFT_116488 and Debaryomyces hansenii CBS767 : DEHA2F26114g
orf19.5858	EGD2	534.16	591.28	701.74	730.69	0.310566898	0.350493	Nascent polypeptide associated complex protein alpha subunit; soluble protein in hyphae; macrophage/pseudohyphal-induced; protein level decrease in stationary phase cultures; GlcNAc-induced protein; Spider biofilm repressed
orf19.1199	NOP5	661.33	587.92	527.07	521.79	-0.243946857	0.578826	Ortholog of S. cerevisiae Nop58; involved in pre-rRNA process; Tn mutation affects filamentous growth; macrophage/pseudohyphal-induced; physically interacts with TAP-tagged Nop1; Spider biofilm repressed
orf19.787.1	orf19.787.1	592.88	587.62	396.88	446.02	-0.457412686	0.062824	Protein of unknown function; ORF added to Assembly 21 based on comparative genome analysis; protein detected by mass spec in stationary phase cultures

orf19.5636	RBT5	658.28	586.18	421.39	332.88	-0.677320652	0.000877	GPI-linked cell wall protein; hemoglobin utilization; Rfg1, Rim101, Tbf1, Fe regulated; Sfu1, Hog1, Tup1, serum, alkaline pH, antifungal drugs, geldamycin repressed; Hap43 induced; required for RPMI biofilms; Spider biofilm induced
orf19.2021	HGT8	420.61	584.39	259.2	386.53	-0.430675755	0.629783	High-affinity glucose transporter of the major facilitator superfamily; 20 members in the C. albicans glucose transporter family; 12 probable membrane-spanning segments; gene has intron; expressed in rich medium; flow model biofilm induced
orf19.7296	orf19.7296	562.57	582.38	198.61	140.24	-1.589411698	1.23E-16	Putative cation conductance protein; similar to stomatin mechanoreception protein; plasma-membrane localized; induced by Rgt1; rat catheter and Spider biofilm induced
orf19.5626	orf19.5626	545.26	576.42	532.56	562.8	-0.041832536	0.998825	Protein of unknown function; Plc1-regulated; induced by Mnl1 under weak acid stress; flow model biofilm induced
orf19.7626	EIF4E	602.65	575.5	653.53	722.47	0.198942068	0.787177	Translation initiation factor eIF4E; genes encoding ribosomal subunits, translation factors, tRNA synthetases downregulated by phagocytosis by macrophage; alternatively spliced intron in 5' UTR; protein levels decrease in stationary phase
orf19.6514	CUP9	531.4	575.41	487.14	452.07	-0.232292327	0.667542	Transcription factor; represses SOK1 expression in response to farnesol inhibition; yeast-hypha switch repressed; ketoconazole-induced; Plc1-regulated; colony morphology-related Ssn6 regulation; Spider, flow model biofilm induced
orf19.1891	APR1	604.53	573.43	701.71	696.92	0.220737185	0.648521	Vacuolar aspartic proteinase; transcript equivalent in yeast-form and mycelial cells but is elevated at lower growth temperatures; upregulated iby human neutrophils; protein enriched in stationary phase; Spider biofilm induced
orf19.5597.1	orf19.5597.1	666.61	572.01	699.69	738.85	0.192860991	0.793313	Has domain(s) with predicted proton-transporting ATP synthase activity, rotational mechanism, proton-transporting ATPase activity, rotational mechanism activity and role in ATP synthesis coupled proton transport
orf19.4274	PUT1	628.71	571.45	597.43	456.1	-0.187829185	0.851328	Putative proline oxidase; alkaline upregulated by Rim101; flow model biofilm induced; Spider biofilm induced

orf19.3223.1	orf19.3223.1	578.15	567.5	641.21	635.33	0.134092438	0.941977	Putative 12kDa subunit of mitochondrial NADH-ubiquinone oxidoreductase; gene has intron
orf19.2877	PDC11	500.29	564.61	695.37	794.77	0.433662109	0.098488	Pyruvate decarboxylase; antigenic; on hyphal not yeast cell surface; Hap43, Gcn4, Efg1, Efh1, Hsf1 regulated; fluconazole, farnesol induced; amino acid starvation repressed; flow model biofilm induced; Spider biofilm repressed
orf19.216.1	orf19.216.1	592.41	562.52	657.42	644.77	0.150073615	0.897436	Ortholog of C. parapsilosis CDC317 : CPAR2_806510, Candida tenuis NRRL Y-1498 : CANTEDRAFT_109089, Debaryomyces hansenii CBS767 : DEHA2B13508g and Pichia stipitis Pignal : PICST_59544
orf19.1591	ERG10	570.06	562.41	750.3	812.51	0.422947492	0.072406	Acetyl-CoA acetyltransferase; role in ergosterol biosynthesis; soluble in hyphae; changes in protein abundance associated with azole resistance; fluconazole or ketoconazole induced; macrophage-downregulated protein; GlcNAc-induced protein
orf19.5024	GND1	527.26	560.98	904.71	1032.19	0.760005083	4.62E-05	6-phosphogluconate dehydrogenase; soluble in hyphae; farnesol, macrophage-induced protein; antigenic in mice; dual localization to cytosol and peroxisomes depends on alternative splicing; rat catheter and Spider biofilm repressed
orf19.6924	HTA1	616.02	559.73	530.94	463.31	-0.23727882	0.613751	Histone H2A; repressed in fkh2 mutant; amphotericin B repressed; farnesol regulated; RNA abundance regulated by tyrosol and cell density; Hap43-induced gene; Spider biofilm repressed
orf19.97	CAN1	672.28	557.19	300.15	232.34	-1.110984169	3.78E-09	Basic amino acid permease; complements lysine transport mutation; 10 predicted transmembrane regions, 3 predicted N-glycosylation sites; phagocytosis by macrophages induces transcript; rat catheter, Spider and flow model biofilm induced
orf19.4445	orf19.4445	642.65	555.71	518.68	390.67	-0.378154885	0.213372	Protein of unknown function; Plc1p-regulated; expression induced early upon infection of reconstituted human epithelium (RHE), while expression of the C. dubliniensis ortholog is not; mutant is viable; Spider biofilm induced
orf19.7466	ACC1	580.81	554.49	647.31	598.72	0.113330052	0.998825	Putative acetyl-coenzyme-A carboxylases; regulated by Efg1; amphotericin B repressed; caspofungin repressed; 5'-UTR intron; gene used for strain identification by multilocus sequence typing; Hap43-induced; flow model biofilm repressed

orf19.4495	NDH51	590.58	553.31	587.73	559.32	-0.007855518	0.998825	Nicotinamide adenine dinucleotide dehydrogenase complex I subunit of the mitochondrial electron transport chain; required for wild-type filamentous growth; alkaline repressed; Hap43-repressed; Spider biofilm repressed
orf19.3854	orf19.3854	443.2	550.3	370.74	396.22	-0.351054185	0.346359	Ortholog of S. cerevisiae Sat4; amphotericin B induced; clade-associated gene expression; Spider biofilm induced
orf19.2125	orf19.2125	527.66	541.32	518.05	540.66	-0.023933367	0.998825	Protein of unknown function; GlcNAc-induced protein; Spider biofilm induced; rat catheter biofilm repressed
orf19.5431	orf19.5431	531.61	540.62	392.31	378.63	-0.452410212	0.061874	Protein of unknown function; Hap43-repressed; Spider biofilm induced
orf19.6923.1	orf19.6923.1	559.55	539.16	626.55	754.1	0.295464339	0.444628	Ortholog of C. parapsilosis CDC317 : CPAR2_502850, Candida tenuis NRRL Y-1498 : CANTEDRAFT_135869, Debaryomyces hansenii CBS767 : DEHA2F18260g and Pichia stipitis Pignal : psti_CGOB_00119
orf19.7269	orf19.7269	577.89	537.39	528.62	464.19	-0.167542864	0.872276	Putative polyamine acetyltransferase; acetylates polyamines (e.g. putrescine, spermidine, spermine) and aralkylamines (e.g. tryptamine, phenylethylamine); Spider biofilm repressed
orf19.5964	ARF2	542.56	537.02	596.92	619.72	0.149146244	0.917335	Putative ADP-ribosylation factor; mutation confers hypersensitivity to Brefeldin A; Spider biofilm repressed
orf19.4631	ERG251	471.71	535.83	272.75	295.42	-0.76283888	0.000345	C-4 sterol methyl oxidase; role in ergosterol biosynthesis; Hap43-induced; ketoconazole-induced; amphotericin B, caspofungin repressed; possibly essential gene, disruptants not obtained by UAU1 method; Spider biofilm repressed
orf19.4427	SKP1	506.2	533.34	616.63	738.73	0.342962402	0.29737	Putative subunit D of kinetochore protein complex CBF3; regulated by Gcn4p; repressed in response to amino acid starvation (3-aminotriazole treatment)

orf19.7053	GAC1	423.11	532.27	566.02	630.63	0.281904795	0.568429	Putative regulatory subunit of ser/thr phosphoprotein phosphatase 1; fluconazole-induced; caspofungin repressed; transcript induced by Mnl1 under weak acid stress; regulated by Nrg1, Tup1; Spider and flow model biofilm induced
orf19.4191	RLP24	550.62	531.14	413.3	434.46	-0.336413716	0.280569	Putative ribosomal protein; Hap43-induced; essential gene; heterozygous mutation confers hypersensitivity to 5-fluorocytosine (5-FC), 5-fluorouracil (5-FU), and tubercidin (7-deazaadenosine); Spider biofilm induced
orf19.3642	SUN41	518.91	530.78	508.61	481.34	-0.091602946	0.998825	Cell wall glycosidase; role in biofilm formation and cell separation; possibly secreted; hypoxia, hyphal induced; caspofungin repressed; Efg1, Cph1 regulated; O-glycosylated, possible Kex2 substrate; 5'-UTR intron; Spider biofilm induced
orf19.7219	FTR1	546.89	530.47	307.35	321.42	-0.725127066	0.000278	High-affinity iron permease; required for mouse virulence, low-iron growth; iron, amphotericin B, caspofungin, ciclopirox, Hog1p, Sef1p, Sfu1p, and Hap43p regulated; complements S. cerevisiae ftr1 iron transport; Hap43p-repressed
orf19.4393	CIT1	534.91	528.61	478.6	417.83	-0.241329864	0.616255	Citrate synthase; induced by phagocytosis; induced in high iron; Hog1-repressed; Efg1-regulated under yeast, not hyphal growth conditions; present in exponential and stationary phase; Spider biofilm repressed; rat catheter biofilm induced
orf19.1996	CHA1	647.48	526.97	821.75	630.23	0.269884985	0.543746	Similar to catabolic ser/thr dehydratases; repressed by Rim101; induced in low iron; regulated on white-opaque switch; filament induced; Tn mutation affects filamentation; flow model biofilm induced; Spider biofilm repressed
orf19.3278	GSY1	514.74	526.57	761.43	764.5	0.50069318	0.015792	UDP glucose/starch glucosyltransferase; transcript repressed by yeast-hyphal switch, Efg1-regulated; strong oxidative stress induced; colony morphology-related regulation by Ssn6; stationary phase enriched; flow model biofilm induced
orf19.6047	TUF1	514.6	525.07	599.58	571.92	0.147306802	0.921805	Translation elongation factor TU; macrophage/pseudohyphal-induced; repressed upon phagocytosis by murine macrophage; levels decrease in stationary phase cells; Spider biofilm repressed
orf19.449	orf19.449	511.9	522.99	524.27	472.03	-0.064998497	0.998825	Putative phosphatidyl synthase; stationary phase enriched protein; transcript repressed by yeast-hypha switch; Hap43-repressed; rat catheter, Spider and flow model biofilm induced

orf19.7676	XYL2	495.06	521.95	620.84	519.79	0.137813156	0.946319	D-xylulose reductase; immunogenic in mice; soluble protein in hyphae; induced by caspofungin, fluconazole, Hog1 and during cell wall regeneration; Mnl1-induced in weak acid stress; stationary phase enriched; flow model biofilm induced
orf19.111	CAN2	759.76	521.38	477.03	298.23	-0.649376975	0.015336	Basic amino acid permease; arginine metabolism; regulated by Nrg1/Tup1; caspofungin, flucytosine induced; colony morphology-related regulation by Ssn6; Hap43-repressed; rat catheter and Spider biofilm induced; promoter bound by Efg1
orf19.7256	orf19.7256	527.05	518.6	458.78	462.22	-0.181185208	0.853836	Ortholog of S. cerevisisae/S. pombe Lsm5; Lsm (Like Sm) protein involved in mRNA decay; Predicted ORF from Assembly 19; removed from Assembly 20; restored based on comparative genome analysis; flucytosine induced
orf19.3059	SUA71	466.61	518.29	341.58	399.64	-0.381780776	0.242853	Transcription factor TFIIB; required for transcription initiation and start site selection by RNA polymerase II; downregulated during planktonic growth, whereas related SUA72 is induced; induced by Tbf1; rat catheter, Spider biofilm induced
orf19.4526	HSP30	369.62	517.77	62.09	61.69	-2.404844238	1.70E-27	Putative heat shock protein; fluconazole repressed; amphotericin B induced; Spider biofilm induced; rat catheter biofilm induced
orf19.6659	GAP6	612.76	516.94	433.49	407.79	-0.403209244	0.124993	Broad-specificity amino acid permease; Plc1, Gcn4 regulated; rat catheter biofilm induced
orf19.5280	MUP1	544.52	515.6	525.77	532.31	-0.014337865	0.998825	Putative high affinity methionine permease; alkaline upregulated by Rim101; Spider biofilm induced
orf19.3618	YWP1	384.74	512.26	87.42	93.85	-2.001097495	7.54E-21	Secreted yeast wall protein; possible role in dispersal in host; mutation increases adhesion and biofilm formation; propeptide; growth phase, phosphate, Ssk1/Ssn6/Efg1/Efh1/Hap43 regulated; mRNA binds She3; flow and Spider biofilm repressed
orf19.7270	orf19.7270	502	512.09	564.09	603.91	0.177799928	0.8593	Protein of unknown function; Spider biofilm repressed

orf19.6531.1	TOM7	447.78	507.07	505.89	592.9	0.173604901	0.892679	Predicted component of the TOM (translocase of outer membrane) complex involved in protein translocation into mitochondria; flucytosine induced
orf19.2871	SDH12	518.48	502.62	584.72	552.21	0.132145361	0.946319	Succinate dehydrogenase; soluble protein in hyphae; macrophage-downregulated protein level; downregulated by Efg1p; repressed by nitric oxide; protein present in exponential and stationary growth phase yeast cultures; Hap43p-repressed gene
orf19.5832	HPT1	510.31	501.73	394.95	421.07	-0.2962571	0.434643	Putative hypoxanthine-guanine phosphoribosyltransferase; protein abundance affected by URA3 expression in the CAI4 strain background; protein level decreases in stationary phase; Spider biofilm induced
orf19.3732	ERG25	453.8	500.75	369.96	407.61	-0.28144638	0.545179	Putative C-4 methyl sterol oxidase; C4-demethylation of ergosterol biosynthesis intermediates, based on similarity to S. cerevisiae Erg25; fluconazole-induced; induced in azole-resistant strain; rat catheter and Spider biofilm induced
orf19.4077	MIT1	462.18	499.52	475.23	501.52	0.008444493	0.998825	Mannosylinositol phosphorylceramide (MIPC) synthase catalytic subunit; sphingolipid biosynthesis; fluconazole, caspofungin induced; macrophage-repressed; Spider biofilm induced
orf19.5854	SBP1	515.45	496.71	598.81	623.93	0.243203295	0.594776	Similar to RNA binding proteins; downregulated upon adherence to polystyrene; stationary-phase enriched protein
orf19.1738	UGP1	470.93	496.03	585.97	657.58	0.324048863	0.340411	UTP-glucose-1-phosphaturidyl transferase; localizes to yeast, not hyphal cell surface; Hog1-repressed; stationary phase enriched; induced in oralpharyngeal candidasis; rat catheter biofilm repressed; Bcr1-repressed in RPMI a/a biofilms
orf19.4195.1	FCA1	544.98	495.97	396.55	444.59	-0.289394429	0.476154	Cytosine deaminase; enzyme of pyrimidine salvage; functional homolog of S. cerevisiae Fcy1p; mutation is associated with resistance to flucytosine (5-FC) in a clinical isolate; hyphal downregulated; gene has intron
orf19.5231.2	ATP19	585.45	494.37	657.27	608.4	0.203200546	0.756052	Subunit k of the mitochondrial F1F0 ATP synthase; a large enzyme complex required for ATP synthesis; Spider biofilm repressed

orf19.2785	ATP7	522.73	493.93	601.05	646.34	0.263211974	0.532956	Putative subunit of the F1F0-ATPase complex; colony morphology-related gene regulation by Ssn6; farnesol, macrophage-downregulated protein abundance; protein present in exponential and stationary yeast growth phases; Hap43-induced
orf19.1030	orf19.1030	528.77	493.1	492.83	467.26	-0.094992833	0.998825	Putative peptidyl-prolyl cis-trans isomerase
orf19.5077	orf19.5077	515.7	491.67	494.82	519.13	-0.002142135	0.998825	Protein of unknown function; Hap43-repressed gene; repressed by nitric oxide
orf19.2570	MCI4	493.33	489.37	490.12	535.19	0.046958657	0.998825	Putative NADH-ubiquinone dehydrogenase; Hap43p-repressed gene
orf19.969	orf19.969	525.83	488.18	508.58	519.7	0.009610291	0.998825	Ortholog(s) have ribose phosphate diphosphokinase activity, role in 5-phosphoribose 1-diphosphate biosynthetic process, cytokinesis, fungal-type cell wall organization and cytosol, nucleus localization
orf19.3974	PUT2	540.22	486.09	683.76	534.37	0.214375302	0.749138	Putative delta-1-pyrroline-5-carboxylate dehydrogenase; alkaline upregulated; protein present in exponential and stationary growth phase yeast cultures; flow model biofilm induced; Spider biofilm induced
orf19.4456	GAP4	442.31	482.2	450.75	434.42	-0.070872624	0.998825	Putative amino acid permease; hyphal induced; regulated by Hap43, Gcn2 and Gcn4; colony morphology-related gene regulation by Ssnp; detected at plasma membrane of yeast and germ tube by mass spec; Spider biofilm induced
orf19.4827	ADE12	502.69	479.7	612.5	635.23	0.309408828	0.339615	Adenylosuccinate synthase; upregulated in biofilm; decreased expression in hyphae vs yeast-form cells; not induced during GCN response, in contrast to S. cerevisiae ADE12, which is induced by Gcn4p; stationary phase-enriched protein
orf19.5791	IDH2	473.64	478.71	483.85	500.99	0.032730678	0.998825	Putative mitochondrial NAD-isocitrate dehydrogenase subunit; induced by ciclopirox; induced in high iron; present in exponential and stationary growth phases; Spider biofilm repressed

orf19.4602	MDH1-1	483.3	476.52	736.19	615.57	0.443655584	0.059238	Predicted malate dehydrogenase precursor; macrophage-induced transcript; protein present in exponential and stationary growth phase yeast cultures; Spider biofilm repressed
orf19.3554	AAT1	513.97	475.39	693.77	691.3	0.442043763	0.050204	Aspartate aminotransferase; soluble protein in hyphae; macrophage-induced protein; alkaline upregulated; amphotericin B repressed; gene used for strain identification by multilocus sequence typing; farnesol-, Hap43p-induced; GlcNAc-induced
orf19.5113	ADH2	532.97	474.57	395.24	318.39	-0.470362229	0.061766	Alcohol dehydrogenase; soluble in hyphae; expression regulated by white-opaque switching; regulated by Ssn6; indued by Mnl1 in weak acid stress; protein enriched in stationary phase yeast cultures; Spider biofilm induced
orf19.287	orf19.287	518.59	474.04	500.02	490.86	-0.013076623	0.998825	Putative NADH-ubiquinone oxidoreductase subunit; Hap43p-repressed gene; repressed by nitric oxide; identified in detergent-resistant membrane fraction (possible lipid raft component)
orf19.3331	ABC1	464.94	473.72	383.54	337.53	-0.36188662	0.240742	Putative ubiquinol-cytochrome-c reductase; induced upon adherence to polystyrene; flow model biofilm induced; Spider biofilm induced
orf19.2571	SEC4	473.54	473.37	505.07	571	0.159916597	0.90951	Small GTPase of Rab family; role in post-Golgi secretion; possible C-terminal palmitoylation; downregulated on adherence to polystyrene; localizes to the Spitzenkorper during hyphal growth; functional homolog of S. cerevisiae Sec4p
orf19.4132	orf19.4132	455.35	472.01	521.28	494.18	0.107790043	0.998825	Protein of unknown function; UPF0057 protein family member; localizes to the plasma membrane; Spider biofilm induced
orf19.5079.1	orf19.5079.1	561.99	471.69	577.52	606.15	0.172997082	0.87024	Ortholog(s) have role in NADH oxidation, programmed cell death and mitochondrial respiratory chain complex I, membrane segment localization
orf19.7021	GPH1	445.82	467.6	820.03	841.99	0.789680657	1.04E-05	Putative glycogen phosphorylase; role in glycogen metabolism; regulated by Ssk1, Mig1, Tup1, Hap43; fluconazole-induced; localizes to cell surface of hyphae, not yeast; stationary phase enriched protein; Spider biofilm induced

orf19.3590	IPP1	451.34	463.2	763.45	822.83	0.726369482	0.000103	Putative inorganic pyrophosphatase; antigenic; soluble protein in hyphae; macrophage-induced protein; protein present in exponential and stationary phase yeast; possibly essential (UAU1 method); sumoylation target; Spider biofilm repressed
orf19.2538	PTC2	487.99	462.92	386.19	376.92	-0.303866712	0.404341	Protein phosphatase of the Type 2C-related family (serine/threonine-specific) with a potential role in DNA damage checkpoint control; localizes to both cytoplasm and mitochondria; mutant shows virulence defect
orf19.506	YDJ1	481.96	460.37	377.66	345.69	-0.361594831	0.232917	Putative type I HSP40 co-chaperone; heavy metal (cadmium) stress-induced
orf19.1727	PMC1	428.5	457.63	368.78	339.95	-0.309721442	0.418229	Vacuolar calcium P-type ATPase; transcript regulated by calcineurin and fluconazole; mutant shows increased resistance to fluconazole, lithium; increased sensitivity to calcium; Spider biofilm induced
orf19.2769	orf19.2769	401.75	456.43	610.64	638.46	0.484890457	0.043728	Putative protease B inhibitor; hyphal-induced expression; Cyr1p- and Ras1p-repressed
orf19.7571	UBC4	499.96	455.24	396.85	392.66	-0.26236445	0.561982	Ortholog(s) have ubiquitin binding activity
orf19.7255	RPC10	443.76	454.35	392.85	495.94	-0.021255692	0.998825	Putative RNA polymerase subunit ABC10-alpha of RNA polymerase complexes I, II, and III; flucytosine induced; Spider biofilm induced
orf19.2947	SNZ1	480.07	452.73	775.81	747.91	0.648783521	0.000451	Stationary phase protein; vitamin B synthesis; induced byyeast-hypha switch, 3-AT or in azole-resistant strain overexpressing MDR1; soluble in hyphae; regulated by Gcn4, macrophage; Spider biofilm induced; rat catheter biofilm repressed
orf19.5956	PIN3	413.59	451.25	399.03	418.67	-0.08594018	0.998825	Putative SH3-domain-containing protein; predicted role in actin cytoskeleton organization; Spider biofilm repressed

orf19.6548	ISU1	525.44	450.9	393.19	268.41	-0.519789683	0.056084	Protein with similarity to NifU; possible role in iron-sulfur cluster biogenesis; induced in low iron; rat catheter biofilm induced; rat catheter and Spider biofilm induced
orf19.2843	RHO1	490.64	447.7	507.1	488.76	0.068058064	0.998825	Small GTPase of Rho family; regulates beta-1,3-glucan synthesis activity and binds Gsc1p; essential; expected to be geranylgeranylated by geranylgeranyltransferase type I; plasma membrane-localized
orf19.1833	orf19.1833	516.21	444.55	421.13	388.92	-0.237446702	0.654087	Ortholog(s) have pseudouridine synthase activity
orf19.7481	MDH1	481.84	444.5	512.43	506.06	0.114594221	0.998825	Mitochondrial malate dehydrogenase; regulated by Mig1, Tup1, white-opaque switch, phagocytosis; induced in high iron; antigenic during murine and human infection; repressed in Spider biofilms by Bcr1, Tec1, Ndt80, Rob1, Brg1
orf19.3812	SSZ1	425.7	444.44	491.05	475.16	0.126375444	0.977056	Putative HSP70 chaperone; protein level decreases in stationary phase cultures; Spider biofilm repressed
orf19.4879.2	NTF2	455.85	442.82	558.39	581.64	0.306556537	0.372168	Putative nuclear envelope protein; regulated by Ssn6; rat catheter biofilm repressed
orf19.6794	FESUR1	484.81	442.34	512.02	486.98	0.089040754	0.998825	Putative ubiquinone reductase; transcriptionally induced by interaction with macrophage; alkaline downregulated; repressed by nitric oxide; clade-associated gene expression; Hap43p-repressed gene
orf19.3507	MCR1	479.59	439.3	534.78	528.06	0.184178922	0.842252	NADH-cytochrome-b5 reductase; soluble in hyphae; alkaline downregulated; farnesol, ketoconazole or flucytosine induced; protein present in exponential and stationary growth phase yeast; YNB biofilm induced; rat catheter biofilm repressed
orf19.1267	orf19.1267	498.95	439.13	369.08	298.15	-0.461723887	0.077996	Ortholog of S. cerevisiae : CAJ1, C. glabrata CBS138 : CAGL0L00957g, C. dubliniensis CD36 : Cd36_45380, C. parapsilosis CDC317 : CPAR2_500650 and Candida tenuis NRRL Y-1498 : CANTEDRAFT_132273

orf19.2340	CDC48	462.28	437.36	534.75	527.67	0.212956431	0.732772	Putative microsomal ATPase; plasma membrane-localized; regulated by Gcn2 and Gcn4; induced by amino acid starvation (3-AT); macrophage/pseudohyphal-repressed; protein levels decrease in stationary phase yeast; Spider biofilm repressed
orf19.7283	orf19.7283	393.64	433.88	121.97	126.26	-1.55305502	9.80E-15	Protein of unknown function; rat catheter and Spider biofilm induced
orf19.4657	orf19.4657	423.96	433.75	443.38	454.88	0.04890051	0.998825	Ortholog(s) have phosphoprotein phosphatase activity, role in negative regulation of phospholipid biosynthetic process, nuclear envelope organization and Nem1-Spo7 phosphatase complex, integral to membrane, mitochondrion localization
orf19.3888	PGI1	377.55	432.93	641.39	667.19	0.620215996	0.003507	Glucose-6-phosphate isomerase; enzyme of glycolysis; antigenic; Efg1-regulated; induced upon adherence to polystyrene; repressed by phagocytosis, human neutrophils; flow model biofilm induced; rat catheter and Spider biofilm repressed
orf19.4706	orf19.4706	380.53	431.92	329.27	324.01	-0.300919834	0.493752	Protein of unknown function; induced in cyr1 or ras1 mutant; induced by fluconazole, by alpha pheromone in SpiderM medium and during oralpharyngeal candidasis; Spider biofilm induced
orf19.717	HSP60	395.06	431.04	426.34	509.83	0.153573513	0.943903	Heat shock protein; soluble in hyphae; regulated by Nrg1 and by iron; induced in high iron; heavy metal (cadmium) stress-induced; sumoylation target; protein present in exponential and stationary phase cells; Hap43-repressed
orf19.6414.3	TPM2	444.41	430.52	682.55	716.85	0.618671427	0.00166	Putative tropomyosin isoform 2; regulated by Gcn4; repressed by amino acid starvation; macrophage-induced; protein levels decrease in stationary cells; Hap43-induced; rat catheter and Spider biofilm repressed
orf19.1650	TOM6	425.56	428.09	521.12	550.39	0.290412232	0.44985	Predicted component of the TOM (translocase of outer membrane) complex, involved in protein import into mitochondria
orf19.3664	HSP31	433.33	425.38	4.53	3.78	-4.407375541	5.93E-69	Putative 30 kda heat shock protein; repressed during the mating process; rat catheter biofilm induced

orf19.3149	LSP1	481.51	424.82	588.04	525.21	0.262026176	0.543746	Eisosome component with a predicted role in endocytosis; protein present in exponential and stationary growth phase yeast cultures; caspofungin repressed; biofilm induced; fungal-specific (no human/murine homolog); sumoylation target
orf19.1552	CPR3	472.01	423.72	492.89	543.68	0.185906619	0.854658	Putative peptidyl-prolyl cis-trans isomerase; macrophage-induced protein; protein levels decrease in stationary phase yeast cultures; predicted mitochondrial localization; overlaps orf19.1551
orf19.7329	orf19.7329	429.26	423.67	423.85	523.16	0.129165519	0.998825	Ortholog(s) have ubiquitin-protein ligase activity
orf19.6068	SVF1	441.36	423.24	435.29	550.36	0.164628452	0.924887	Putative survival factor; stationary phase enriched protein; fluconazole-induced; regulated by Gcn4p; repressed in response to amino acid starvation (3-AT treatment); fungal-specific (no human or murine homolog)
orf19.6062	orf19.6062	428.67	422.78	430.07	456.56	0.043000538	0.998825	Putative TIM23 translocase complex subunit; membrane-localized; Hap43-repressed
orf19.6117	orf19.6117	401.74	420.93	274.26	250.61	-0.60265472	0.009192	S. pombe ortholog SPAC5D6.04 is a predicted auxin family transmembrane transporter; ketoconazole and hypoxia induced
orf19.7161	SUI3	422.86	420.24	332.19	330.46	-0.330702777	0.346359	Putative translation initiation factor; genes encoding ribosomal subunits, translation factors, and tRNA synthetases are downregulated upon phagocytosis by murine macrophage
orf19.4413	CMD1	453.19	419.67	517.23	591.83	0.309072029	0.404341	Calmodulin; calmodulin inhibitors cause a defect in hyphal growth; transcript not regulated by yeast-hyphal transition; mutation confers hypersensitivity to cytochalasin D and high concentrations of tunicamycin; gene has intron
orf19.5544	SAC6	445.08	418.53	512.78	449.08	0.130327908	0.960406	Fimbrin; actin filament bundling protein; transcript regulated by Nrg1 and Mig1; protein level decreases in stationary phase

orf19.492	ADE17	411.07	418.01	563.61	578.62	0.416498717	0.10284	5-Aminoimidazole-4-carboxamide ribotide transformylase, enzyme of adenine biosynthesis; antigenic in human; soluble protein in hyphae; not induced during GCN response, in contrast to the S. cerevisiae ortholog
orf19.868	ADAEC	428.18	415.66	474.91	431.35	0.081631922	0.998825	Protein of unknown function; transcription is specific to white cell type
orf19.3052	YPT1	402.57	412.62	422.45	474.33	0.113250353	0.998825	Functional homolog of S. cerevisiae Ypt1p, which is an essential small Ras-type GTPase involved in protein secretion at ER-to-Golgi; dominant-negative mutation causes SAP secretion defect and accumulation of intracellular secretory vesicles
orf19.1945	AUR1	312.49	412.6	367.36	489.84	0.200410605	0.892679	Inositolphosphorylceramide (IPC) synthase; catalyzes the key step in sphingolipid biosynthesis; antifungal drug target; flow model biofilm induced; Spider biofilm induced
orf19.7196	orf19.7196	500.65	411.61	472.1	426.87	-0.030296507	0.998825	Putative vacuolar protease; upregulated in the presence of human neutrophils; Spider biofilm induced
orf19.953.1	COF1	356.15	411.47	601.13	682.62	0.665971458	0.00214	Putative cofilin; macrophage-induced protein; protein present in exponential and stationary-phase yeast cells, but higher amounts in stationary phase
orf19.6515	HSP90	386.59	409.35	394.94	424.42	0.026199079	0.998825	Essential chaperone, regulates several signal transduction pathways and temperature-induced morphogenesis; activated by heat shock, stress; localizes to surface of hyphae, not yeast cells; mediates echinocandin and biofilm azole resistance
orf19.7001	YCK2	399.17	409.16	391.98	414.87	-0.012704177	0.998825	Plasma membrane protein similar to S. cerevisiae casein kinase I, Yck2p; null mutant has defect in damaging oral epithelial cells and in hyphal branching; transcription is activated in weak acid stress or on contact with host cells
orf19.637	SDH2	422.29	409.15	392.61	392.42	-0.086570457	0.998825	Succinate dehydrogenase, Fe-S subunit; localizes to surface of yeast cells, but not hyphae; induced in high iron and during log phase aerobic growth; repressed by nitric oxide, Hap43

orf19.2929	GSC1	331.35	405.88	369.33	379.56	0.006640532	0.998825	Essential beta-1,3-glucan synthase subunit; gsc1 allele determines resistance/sensitivity to echinocandins; 16 predicted membrane-spanning regions; mRNA abundance declines after yeast-to-hypha transition; Spider biofilm induced
orf19.1682	orf19.1682	414.84	404.67	430.32	433.98	0.058343774	0.998825	Membrane protein; Hap43p-repressed gene; repressed by nitric oxide
orf19.6882	OSM1	377.16	403.86	403.42	409.23	0.038896894	0.998825	Putative flavoprotein subunit of fumarate reductase; soluble protein in hyphae; caspofungin repressed; stationary phase enriched protein; flow model biofilm induced; Spider biofilm repressed
orf19.7341.1	orf19.7341.1	415.05	403.63	342.25	317.5	-0.299283411	0.458126	Protein of unknown function; Spider biofilm induced
orf19.2954	orf19.2954	514.95	401.94	453.13	435.01	-0.050389303	0.998825	Protein of unknown function; Hap43-repressed gene; repressed by nitric oxide
orf19.6179	orf19.6179	456.03	401.65	451.5	389.21	-0.039835233	0.998825	Ortholog of Candida albicans WO-1 : CAWG_03090
orf19.2496	ATO2	405.36	401.4	370.96	413.35	-0.04612769	0.998825	Putative fungal-specific transmembrane protein; fluconazole repressed, Hap43-repressed; flow model biofilm induced; Spider biofilm induced
orf19.3575	CDC19	348.37	399.97	683.46	801.37	0.889674268	7.18E-06	Pyruvate kinase at yeast cell surface; Gcn4/Hog1/GlcNAc regulated; Hap43/polystyrene adherence induced; repressed by phagocytosis/farnesol; hyphal growth role; stationary phase enriched; flow model biofilm induced; Spider biofilm repressed
orf19.4936.1	orf19.4936.1	504	399.82	356.59	280.9	-0.466100457	0.09614	Putative adhesin-like protein; Spider biofilm induced

orf19.3684	orf19.3684	395.74	399.48	539.86	571.84	0.435291198	0.084877	Putative oxidoreductase; Spider biofilm induced
orf19.2821	orf19.2821	441.21	399.31	431.92	446.44	0.049339711	0.998825	Protein of unknown function; Hap43-repressed gene; repressed by nitric oxide
orf19.2314	orf19.2314	397.82	397.67	307.55	340.53	-0.277777453	0.568429	Protein with a role in nucleolar integrity and processing of pre-rRNA; mutation confers hypersensitivity to 5-fluorocytosine (5-FC), 5-fluorouracil (5-FU), and tubercidin (7-deazaadenosine); Hap43-induced; Spider biofilm induced
orf19.813	orf19.813	455.71	396.92	338.23	321.65	-0.348666437	0.288073	Protein of unknown function; mutants are viable; Hap43-induced gene; oxidative stress-induced via Cap1; rat catheter and Spider biofilm induced
orf19.1655.3	orf19.1655.3	440.74	396.52	325.54	319.89	-0.35359073	0.27463	Ortholog(s) have role in cation transport, regulation of membrane potential and plasma membrane localization
orf19.2344	ASR1	333.63	396.36	488.52	535.28	0.430321849	0.142022	Heat shock protein; transcript regulated by cAMP, osmotic stress, ciclopirox olamine, ketoconazole; repressed by Cyr1, Ras1; colony morphology-related regulated by Ssn6; stationary phase enriched; Hap43-induced; Spider biofilm induced
orf19.6190	SRB1	395.72	390.57	478.86	435.43	0.185801955	0.853836	Essential GDP-mannose pyrophosphorylase; makes GDP-mannose for protein glycosylation; functional in S. cerevisiae psa1; on yeast-form, not hyphal cell surface; alkaline induced; induced on adherence to polystyrene; Spider biofilm repressed
orf19.285	orf19.285	386.72	390.17	386.7	384.47	-0.022045733	0.998825	Ortholog of S. cerevisiae Igo2; role in mRNA stabilization of mRNA; Spider biofilm induced
orf19.7382	CAM1	419.43	389.19	477.81	455.75	0.181718469	0.858075	Putative translation elongation factor eEF1 gamma; protein level decreased in stationary phase cultures; Spider biofilm repressed

orf19.6489	FMP45	394.57	389.12	144.48	128.31	-1.374355878	4.10E-12	Predicted membrane protein induced during mating; mutation confers hypersensitivity to toxic ergosterol analog, to amphotericin B; alkaline repressed; repressed by alpha pheromone in SpiderM medium; rat catheter, Spider biofilm induced
orf19.1367.1	orf19.1367.1	409.28	388.94	439.71	453.5	0.138965228	0.951606	Ortholog of C. parapsilosis CDC317 : CPAR2_407330, Candida tenuis NRRL Y-1498 : CANTEDRAFT_114646, Debaryomyces hansenii CBS767 : DEHA2B05654g and Pichia stipitis Pignal : PICST_44644
orf19.4623	orf19.4623	328.45	388.91	342.79	355.54	-0.046761866	0.998825	Protein of unknown function; Spider biofilm repressed
orf19.6731.1	orf19.6731.1	386.92	387.55	547.82	585.5	0.494047427	0.033604	Ortholog of C. dubliniensis CD36 : Cd36_87460, C. parapsilosis CDC317 : CPAR2_808260, Debaryomyces hansenii CBS767 : DEHA2F07084g and Pichia stipitis Pignal : psti_CGOB_00185
orf19.7239	MDG1	364.92	387.09	330.88	333.51	-0.173800566	0.892679	Ortholog(s) have role in pheromone-dependent signal transduction involved in conjugation with cellular fusion and cytoplasm, membrane raft, nucleus, plasma membrane localization
orf19.4468	orf19.4468	443	386.86	444.49	511.56	0.179435863	0.878136	Putative succinate dehydrogenase; localized to the mitochondrial membrane; Hap43p-repressed gene
orf19.5440	RPT2	451.84	385.63	422.14	405.96	-0.025403879	0.998825	Putative ATPase of the 19S regulatory particle of the 26S proteasome; oxidative stress-induced via Cap1; Spider biofilm repressed
orf19.5294	PDB1	441.01	384.88	672.05	578.48	0.540851942	0.011405	Putative pyruvate dehydrogenase; fluconazole-induced; protein level decreases in stationary phase cultures; Spider biofilm repressed
orf19.5886	CUP5	405.91	384.62	507.04	514.08	0.329207021	0.306133	Putative proteolipid subunit of the vacuolar H(+)-ATPase V0 sector; required for vacuolar acidification, secretion, filamentous growth; caspofungin repressed

orf19.3366.1	orf19.3366.1	398.96	384.51	434.82	466.49	0.174288081	0.878538	Protein of unknown function; Hap43-repressed gene
orf19.1671	UTR2	411.53	384.2	384.36	359.69	-0.100796267	0.998825	Putative GPI anchored cell wall glycosidase; role in adhesion, hyphal growth on Spider (not serum) medium; chitin-binding, glycosyl hydrolase domains; induced during cell wall regeneration; mRNA in yeast-form cells; Spider biofilm induced
orf19.4560	BFR1	349.9	380.54	407.12	432.51	0.170812706	0.894293	Protein involved in the maintenance of normal ploidy; S. cerevisiae ortholog cofers Brefeldin A resistance; stationary phase enriched protein; Spider biofilm repressed
orf19.2724	orf19.2724	330.76	379.9	233.56	269.3	-0.457006723	0.147108	Protein of unknown function; flow model, rat catheter and Spider biofilm induced; Hap43-repressed
orf19.2701	orf19.2701	371.89	378.53	457.61	372.96	0.118286641	0.998825	Protein of unknown function; Ras1 and Cyr1 repressed; rat catheter and Spider biofilm induced
orf19.1872	orf19.1872	394.82	376.5	382.88	412.89	0.03083669	0.998825	Plasma membrane protein; repressed by nitric oxide
orf19.1179	orf19.1179	415.03	376.33	445.05	430.18	0.123538077	0.991735	Protein of unknown function; induced in high iron; possibly subject to Kex2 processing; Hap43-repressed
orf19.6178	FBP1	444.85	376.16	409.47	332.82	-0.144815995	0.951606	Fructose-1,6-bisphosphatase; key gluconeogenesis enzyme; regulated by Efg1, Ssn6; induced by phagocytosis; effects switch from glycolysis to gluconeogenesis in macrophage; rat flow model biofilm induced; overlaps orf19.6179
orf19.1089	PEX11	447.78	374.61	351.78	383.88	-0.154000506	0.938642	Putative peroxisomal membrane protein; role in fatty acid oxidation; expression is Tac1-regulated; Hms1p-dependent induction by geldamycin; Spider biofilm induced

orf19.7264	orf19.7264	436.89	374.34	401.59	364.06	-0.085907827	0.998825	Metalloprotease subunit of the 19S regulatory particle of the 26S proteasome lid; couples the deubiquitination and degradation of proteasome substrates; role in fission of mitochondria and peroxisome; Spider biofilm repressed
orf19.909	STP4	367.92	374.26	178.17	151.6	-1.058318837	3.45E-07	C2H2 transcription factor; induced in core caspofungin response; colony morphology-related gene regulation by Ssn6; induced by 17-beta-estradiol, ethynyl estradiol; rat catheter and Spider biofilm induced
orf19.3941	URA7	347.43	373.72	311.44	340.1	-0.144667948	0.957711	CTP synthase 1; flucytosine induced; protein present in exponential and stationary growth phase yeast cultures
orf19.2887	orf19.2887	407.88	373.21	496.04	445.71	0.237316416	0.654087	Ortholog in S. cerevisiae is localizedto the bud, mating projection tip, and associates with ribosomes; Spider biofilm induced
orf19.583	orf19.583	362.2	372.65	302.05	301.9	-0.269421163	0.596328	Indoleamine 2,3-dioxygenase (IDO); rate-limiting enzyme of tryptophan catabolism via kynurenine; enzyme inhibition by 1-methyl-D,L-tryptophan increases hyphal growth; expressed in yeast and hyphae; rat catheter and Spider biofilm induced
orf19.2286	orf19.2286	369.53	369.79	287.62	303.42	-0.30528274	0.476154	Putative deoxyhypusine hydroxylase; ketoconazole-induced; protein level decreases in stationary phase cultures; required for biofilm formation; Spider biofilm repressed
orf19.2775	IDI1	366.49	369.25	319.9	312.22	-0.210488382	0.811201	Ortholog(s) have isopentenyl-diphosphate delta-isomerase activity, role in farnesyl diphosphate biosynthetic process and cytosol, nucleus localization
orf19.2644	QCR2	367.45	367.31	429.9	415.54	0.175951039	0.87796	Ubiquinol-cytochrome-c reductase; antigenic; induced by interaction with macrophage; repressed by nitric oxide; in detergent-resistant membrane fraction (possible lipid raft component); levels decrease in stationary phase; Hap43p-repressed
orf19.7084	DFI1	327.17	366.64	341.68	368.75	0.019415638	0.998825	Cell-surface associated glycoprotein; promotes activation of Cek1 in a matrix-dependent manner; N-glycosylated; Spider biofilm induced

orf19.1751	SPT23	364.6	366.16	345.49	362.69	-0.052810289	0.998825	Protein involved in regulation of unsaturated fatty acid biosynthesis; controls the expression of the Ole1p delta-9-fatty acid desaturase
orf19.1415	FRE10	374.25	365.22	402.37	464.58	0.201104714	0.842252	Major cell-surface ferric reductase under low-iron conditions; 7 transmembrane regions and a secretion signal predicted; Tup1, Rim101, Ssn6, Hog1, caspofungin repressed; ciclopirox olamine induced; rat catheter biofilm induced
orf19.7108	orf19.7108	419.51	365.14	474.4	493.48	0.267949504	0.554747	D-ribulose-5-phosphate 3-epimerase; stationary phase enriched protein
orf19.3782.2	orf19.3782.2	410.65	365.07	462.04	461.81	0.222113014	0.727904	Ortholog(s) have role in cristae formation and integral to mitochondrial inner membrane, mitochondrial crista junction localization
orf19.542	HXK2	277.77	365.01	515.33	711.75	0.80836423	0.001056	Hexokinase II; antigenic in humans; repressed by human neutrophils; Efg1-regulated; fluconazole-induced; gene regulation by Ssn6; present in exponential and stationary growth phase; flow model biofilm induced; Spider biofilm repressed
orf19.3870	ADE13	380.08	364.12	485.05	496.61	0.358037776	0.240742	Adenylosuccinate lyase; enzyme of adenine biosynthesis; soluble protein in hyphae; not induced during GCN response, in contrast to the S. cerevisiae ortholog; repressed by nitric oxide
orf19.2241	PST1	303.55	364.1	533.1	536.21	0.60287284	0.00875	Putative 1,4-benzoquinone reductase; hyphal-induced; regulated by Cyr1, Ras1, Efg1, Nrg1, Rfg1, Tup1; Hap43-induced; Spider biofilm induced
orf19.7327	PHO88	393.57	362.8	365.32	388.29	-0.016185255	0.998825	Protein with a role in phosphate transport; biofilm-regulated expression; amphotericin B repressed
orf19.6287	AAT21	453.99	362.58	346.37	264.46	-0.391184904	0.250373	Putative aspartate aminotransferase; stationary phase enriched protein; Gcn4-regulated; Spider biofilm induced

orf19.1052	orf19.1052	360.19	362.48	370.25	284.3	-0.144455109	0.957711	Predicted histone H2B; Hap43-induced gene; Spider biofilm repressed
orf19.4215	FET34	349.52	360.98	313.59	372.06	-0.05589169	0.998825	Multicopper ferroxidase; induced by low iron, ciclopirox olamine, ketoconazole, hypoxia; alkaline induced by Rim101; repressed in fluconazole-resistant isolate; Sfu1, Hog1 repressed; complements S. cerevisiae fet3; Spider biofilm induced
orf19.4309	GRP2	383.07	360.91	325.64	304.42	-0.230523232	0.730295	Methylglyoxal reductase; regulation associated with azole resistance; induced in core stress response or by oxidative stress via Cap1, fluphenazine, benomyl, by Hap43 or with long term fluconazole treatment; Spider biofilm induced
orf19.2967	TIF34	360.03	359.9	345.87	322.54	-0.109241195	0.998825	Putative translation initiation factor eIF3, p39 subunit; mutation confers hypersensitivity to roridin A, verrucarin A; downregulated upon phagocytosis by murine macrophages; Spider biofilm repressed
orf19.3757	ATP20	403.03	359.36	411.33	405.85	0.082452464	0.998825	Putative mitochondrial ATP synthase; shows colony morphology-related gene regulation by Ssn6p; flucytosine induced; caspofungin repressed; macrophage/pseudohyphal-induced
orf19.7284	ASR2	338.83	358.57	274.79	290.79	-0.284773833	0.568429	Adenylyl cyclase and stress responsive protein; induced in cyr1 or ras1 mutant; stationary phase enriched protein; Spider biofilm induced
orf19.1277	orf19.1277	379.83	357.91	402.45	317.9	-0.046428275	0.998825	Protein of unknown function; Rgt1, Hap43-repressed; flow model biofilm induced; Spider biofilm induced
orf19.4577.3	TIM10	423.45	356.66	478.22	491.58	0.280637346	0.517563	Predicted protein of the mitochondrial intermembrane space with role in protein import into mitochondrial inner membrane
orf19.4223	GCD11	355.84	356.64	302.89	301.7	-0.227042908	0.754884	Gamma subunit of translation initiation factor eIF2; involved in identification of the start codon; likely essential for growth, based on an insertional mutagenesis strategy; Spider biofilm repressed

orf19.544.1	PRE6	394.8	355.62	353.53	378.88	-0.04065053	0.998825	Putative alpha-4 subunit of the proteasome; reported as macrophage-induced protein and macrophage/pseudohyphal-repressed gene; regulated by Gcn2p and Gcn4p; removed from/reinstated in Assembly 20 (see Locus History)
orf19.332.1	orf19.332.1	394.27	355.45	392.49	350.08	-0.023920359	0.998825	Ortholog of C. parapsilosis CDC317 : CPAR2_102640, C. dubliniensis CD36 : Cd36_83290, Candida tenuis NRRL Y-1498 : CANTEDRAFT_98613 and Lodderomyces elongisporus NRLL YB-4239 : LELG_02758
orf19.4622	orf19.4622	291.66	353.92	301.87	303.98	-0.096978554	0.998825	Ortholog(s) have role in telomere maintenance, transcription-coupled nucleotide-excision repair, ubiquitin-dependent protein catabolic process and nucleus localization
orf19.7664	orf19.7664	351.78	352.64	268.78	247.53	-0.417903836	0.172061	Ortholog(s) have nucleolus localization
orf19.819	orf19.819	377.08	352.46	394.99	377.71	0.066412541	0.998825	Ortholog(s) have role in lipoate biosynthetic process and mitochondrion localization
orf19.7236	TIF35	345.74	352.4	312.79	292.8	-0.198067727	0.853836	Putative translation initiation factor; repressed upon phagocytosis by murine macrophage; Spider biofilm repressed
orf19.6343	FEN1	309.88	351.77	206.53	262.02	-0.448470214	0.198279	Putative fatty acid elongase; predicted role in sphingolipid biosynthesis; possibly an essential gene, disruptants not obtained by UAU1 method; Spider and flow model biofilm induced
orf19.1288	FOX2	361.78	350.62	509.24	482.46	0.425519295	0.099285	3-hydroxyacyl-CoA epimerase; fatty acid beta-oxidation; induced by phagocytosis; regulated by Mig1, by white-opaque switch, by DNA methylation; transcriptional activation by oleate requires Ctf1; rat catheter and Spider biofilm induced
orf19.5288.1	orf19.5288.1	249.68	350.15	332.41	472.4	0.352188208	0.532956	Protein of unknown function; Spider biofilm repressed

orf19.5076	PFY1	350.43	348.73	440.41	510.59	0.395391418	0.201062	Profilin; functional homolog of S. cerevisiae Pfy1; hyphae, macrophage/pseudohyphal-induced; regulated by Nrg1, Tup1; gene lacks intron (unlike S. cerevisiae PFY1); complements growth of S. cerevisiae srv2 mutant; nonessential
orf19.3290	orf19.3290	374.47	346.35	328.04	367.04	-0.054751569	0.998825	Plasma membrane-localized protein; repressed by nitric oxide; Hap43p-repressed gene
orf19.3133	GUT2	333.73	344.61	253.86	253.74	-0.389878271	0.240742	Glycerol-3-phosphate dehydrogenase; Plc1p-regulated; rat catheter biofilm induced; Spider biofilm induced
orf19.6898.1	orf19.6898.1	349.39	344.03	323.09	409.44	0.063496143	0.998825	Ortholog(s) have mitochondrion localization
orf19.3733	IDP2	341.09	343.84	238.29	224.91	-0.522088124	0.046807	Isocitrate dehydrogenase; white-opaque switch regulated; morphology-regulation by Ssn6; protein in exponential and stationary phase yeast; Hap43-repressed; Spider biofilm repressed by Bcr1, Tec1, Ndt80, Rob1, Brg1; Spider biofilm induced
orf19.4555	ALS4	307.92	342.88	54.19	52.86	-2.212227314	7.61E-24	GPI-anchored adhesin; role in adhesion, germ tube induction; growth, temperature regulated; expressed during infection of human buccal epithelial cells; repressed by vaginal contact; biofilm induced; repressed during chlamydospore formation
orf19.1665	MNT1	375.15	342.71	326.51	316.28	-0.155678635	0.932191	Alpha-1,2-mannosyl transferase; predicted type II Golgi membrane protein; adds 2nd mannose during cell-wall mannoprotein biosynthesis; required for wild-type virulence and adherence to epithelial cells; Hap43-induced
orf19.1815	orf19.1815	399.35	341.73	316.66	297.91	-0.254555248	0.645572	Ortholog of S. cerevisae/S. pombe Tif6; constituent of 66S pre-ribosomal particles; Spider biofilm induced
orf19.2296	orf19.2296	284.04	341.68	308.52	347.97	0.049739218	0.998825	Predicted mucin-like protein; ketoconazole-induced; fluconazole-repressed; induced in cyr1 mutant; colony morphology-related gene regulation by Ssn6; flow model biofilm induced; Spider biofilm induced

orf19.3749	OPT3	371.1	341.6	229.75	218.23	-0.616337188	0.009196	Oligopeptide transporter; transcript induced by macrophage phagocytosis, BSA or peptides; fluconazole-induced; induced by Rim101 at pH 8; virulence-group-correlated expression; Hap43-repressed; Spider biofilm induced
orf19.7279.1	orf19.7279.1	301.41	341.16	207.56	175.66	-0.673710332	0.007248	Protein of unknown function; Spider biofilm induced
orf19.6447	ARF1	398.24	340.76	246.79	224.38	-0.597339833	0.012365	ADP-ribosylation factor; probable GTPase involved in intracellular transport; one of several C. albicans ADP-ribosylation factors; N-myristoylprotein; substrate of Nmt1; Spider biofilm induced
orf19.5079	CDR4	316.73	338.33	103.18	87.77	-1.56112212	2.08E-13	Putative ABC transporter superfamily; fluconazole, Sfu1, Hog1, core stress response induced; caspofungin repressed; fluconazole resistance not affected by mutation or correlated with expression; rat catheter and flow model biofilm induced
orf19.5178	ERG5	296.54	337.86	309.96	338.34	0.014127592	0.998825	Putative C-22 sterol desaturase; fungal C-22 sterol desaturases are cytochrome P450 enzymes of ergosterol biosynthesis, catalyze formation of the C-22(23) double bond in the sterol side chain; transposon mutation affects filamentous growth
orf19.6594	PLB3	315.21	337.72	157.37	165.57	-0.911889761	4.70E-05	GPI-anchored cell surface phospholipase B; possibly secreted; fungal-specific (no mammalian homolog); induced by Tbf1; fluconazole-induced; possible essential gene (UAU1 method); Spider and flow model biofilm induced
orf19.2183	KRE30	340.4	336.73	282.08	258.71	-0.305626934	0.485609	YEF3-subfamily ABC family protein; predicted not to be a transporter; repressed in core stress response; mutation confers hypersensitivity to amphotericin B
orf19.1618	GFA1	302.06	333.7	339.73	412.45	0.20670486	0.858075	Glucosamine-6-phosphate synthase, homotetrameric enzyme of chitin/hexosamine biosynthesis; inhibited by UDP-GlcNAc, FMDP, N-acyl peptide, kanosamine-6-P; functional homolog of S. cerevisiae Gfa1p; Cagrowth-phase regulated; catalytic Cys
orf19.2687.1	orf19.2687.1	369.57	328.72	283.37	311.95	-0.218286127	0.810399	Ortholog(s) have RNA polymerase I activity, RNA polymerase II activity, RNA polymerase III activity, RNA-directed RNA polymerase activity, zinc ion binding activity

orf19.4093	PES1	370.03	327.51	307.86	263.45	-0.274290568	0.594237	Pescadillo homolog required for yeast cell growth, lateral yeast growth on filamentous cells and virulence in mice; hyphal cells grow normally in mutant; mutation confers hypersensitivity to 5-fluorocytosine, 5-fluorouracil, tubercidin
orf19.3391	ADK1	358.52	327.08	500.52	486.32	0.472355883	0.05054	Putative adenylate kinase; repressed in hyphae; macrophage-induced protein; adenylate kinase release used as marker for cell lysis; possibly essential (UAU1 method); flow model biofilm induced; rat catheter and Spider biofilm repressed
orf19.4225.1	orf19.4225.1	325.56	325.44	301.49	305.56	-0.102696273	0.998825	Ortholog(s) have mitochondrion localization
orf19.1252	YME1	333.4	324.49	286.64	299.1	-0.159836547	0.933246	Ortholog(s) have ATP-dependent peptidase activity
orf19.2524	MGE1	317.17	324.14	332.8	362.45	0.095249482	0.998825	Putative mitochondrial matrix cochaperone; macrophage/pseudohyphal-repressed
orf19.3707	YHB1	309.9	324.03	327.34	317.34	0.007859504	0.998825	Nitric oxide dioxygenase; acts in nitric oxide scavenging/detoxification; role in virulence in mouse; transcript activated by NO, macrophage interaction; Hap43, hypha repressed; mRNA binds She3
orf19.1353	orf19.1353	404.98	323.94	335.49	418.16	0.037838317	0.998825	Protein of unknown function; repressed by yeast-hypha switch; Ras1-regulated; oral infection induced; mutants defective in damage to oral epithelium; flow model biofilm induced; Spider biofilm induced
orf19.2710	orf19.2710	341.22	323.79	333.45	365.43	0.054248141	0.998825	Ortholog(s) have enzyme activator activity, role in chromosome segregation and cytoplasm, nucleus localization
orf19.3541	ERF1	344.64	323.14	324.66	325.07	-0.046255307	0.998825	Putative translation release factor 1, which interacts with stop codons and promotes release of nascent peptides from ribosomes; Hap43p-induced gene

orf19.1625	orf19.1625	340.59	323.09	321.4	345.13	-0.005519661	0.998825	Putative ubiquinone oxidoreductase; repressed by nitric oxide; Hap43p-repressed
orf19.7585	INO1	313.48	321.46	374.51	353.31	0.168853663	0.904026	Inositol-1-phosphate synthase; antigenic in human; repressed by farnesol in biofilm or by caspofungin; upstream inositol/choline regulatory element; glycosylation predicted; rat catheter, flow model induced; Spider biofilm repressed
orf19.150	TIM17	326.31	321.11	288.67	284.3	-0.170233739	0.911702	Predicted component of the Translocase of the Inner Mitochondrial membrane (TIM23 complex), involved in protein import into mitochondria
orf19.3215	orf19.3215	307.12	319.33	328.83	322.52	0.040923846	0.998825	Putative plasma membrane protein; in S. cerevisiae it is localized to the cell bud and mating projection membrane; repressed by alpha pheromone in SpiderM medium
orf19.1658	orf19.1658	344.81	318.46	360.11	309.04	-0.000602471	0.998825	Ortholog(s) have cell cortex, cellular bud neck, cellular bud tip, mating projection tip localization
orf19.2175	orf19.2175	307.94	317.7	132.93	122.85	-1.149131655	1.08E-07	Putative mitochondrial cell death effector; induced by nitric oxide; Spider biofilm induced; rat catheter biofilm repressed
orf19.6073	HMX1	461.22	317.61	358.75	273.35	-0.276787543	0.665347	Heme oxygenase; utilization of hemin iron; transcript induced by heat, low iron, or hemin; repressed by Efg1; induced by low iron; upregulated by Rim101 at pH 8; Hap43-induced; Spider and flow model biofilm induced
orf19.5519	GCV1	364.56	316.82	335.49	307.72	-0.08766892	0.998825	Putative T subunit of glycine decarboxylase; transcript negatively regulated by Sfu1; Spider biofilm repressed
orf19.5081	FUN12	310.24	315.84	308.92	309.61	-0.026385869	0.998825	Functional homolog of S. cerevisiae Fun12 translation initiation factor eIF5B; genes encoding ribosomal subunits, translation factors, and tRNA synthetases are downregulated upon phagocytosis by murine macrophage

orf19.5537	WSC2	281.41	314.98	221.8	220.26	-0.397791557	0.266406	Putative cell wall integrity and stress response protein; mRNA binds She3; Spider biofilm induced
orf19.85	GPX2	290.25	314.85	220.68	201.89	-0.474835385	0.115209	Similar to glutathione peroxidase; induced in high iron; alkaline induced by Rim101; induced by alpha factor or interaction with macrophage; regulated by Efg1; caspofungin repressed; Spider biofilm induced
orf19.501	orf19.501	326.07	314.77	286.06	276.67	-0.180925054	0.885512	Ortholog(s) have RNA methyltransferase activity, role in maturation of LSU-rRNA from tricistronic rRNA transcript (SSU-rRNA, 5.8S rRNA, LSU-rRNA) and nucleolus, preribosome, large subunit precursor localization
orf19.3291	HMT1	323.7	313.42	288	261.34	-0.20638633	0.842252	Major type I protein arginine methyltransferase (PRMT); involved in asymmetric dimethylation of arginine residues; involved in nuclear export of Npl3p; Spider biofilm repressed
orf19.5576	orf19.5576	310.15	313.38	284.83	290.25	-0.115877357	0.998825	Putative pantothenate kinase; ortholog of S. cerevisiae Cab1; transposon mutation affects filamentous growth; repressed in core stress response
orf19.4048	DES1	306.72	312.68	345.8	374.4	0.185058099	0.878136	Putative delta-4 sphingolipid desaturase; planktonic growth-induced gene
orf19.1187	CPH2	284.01	312.39	208.16	198.09	-0.506802822	0.081502	Myc-bHLH transcription factor; promotes hyphal growth; directly regulates Tec1 to induce hypha-specific genes; probably homodimeric, phosphorylated; required for colonization of the mouse GI tract; rat catheter and Spider biofilm induced
orf19.1974	TFS1	319.38	311.93	428.19	425.79	0.388206337	0.196689	Putative carboxypeptidase y inhibitor; transcript regulated upon yeast-hypha switch; colony morphology-related gene regulation by Ssn6
orf19.4779	orf19.4779	294.15	310.95	42.38	26.48	-2.574850689	2.68E-28	Putative transporter; slightly similar to the Sit1p siderophore transporter; Gcn4p-regulated; fungal-specific; induced by Mnl1p under weak acid stress

orf19.691	GPD2	324.72	309.12	230.67	202.54	-0.503413769	0.067926	Surface protein similar to glycerol 3-P dehydrogenase; binds host Factor H, FHL-1, plasminogen; regulated by Ssn6, Nrg1, Efg1; induced by cell wall regeneration, macrophage/pseudohyphal growth, core stress response; Spider biofilm induced
orf19.1342	SHM1	351.79	307.04	380.06	344.28	0.112936696	0.998825	Mitochondrial serine hydroxymethyltransferase; complements the glycine auxotrophy of an S. cerevisiae shm1 null shm2 null gly1-1 triple mutant; protein present in exponential and stationary growth phase yeast cultures
orf19.7397.1	orf19.7397.1	357.55	306.29	260.97	234.27	-0.392411552	0.240742	Predicted ORF identical to NSA2
orf19.7424	NSA2	357.55	306.29	260.97	234.27	-0.392411552	0.240742	Putative protein constituent of 66S pre-ribosomal particles; Hap43-induced; repressed by prostaglandins
orf19.754	YBN5	307.89	304	330.76	314.13	0.056779892	0.998825	P-loop ATPase with similarity to human OLA1 and bacterial YchF; Spider biofilm repressed
orf19.4751.1	orf19.4751.1	320.32	302.9	395.25	466.01	0.414854484	0.181137	Has domain(s) with predicted NADH dehydrogenase (ubiquinone) activity and mitochondrion localization
orf19.7187	MAM33	309	302.18	279.74	282.23	-0.11993781	0.998825	Putative mitochondrial acidic matrix protein; regulated by Ssn6p; protein present in exponential and stationary growth phase yeast cultures
orf19.7178	PRE5	350.02	302.01	324.41	287.61	-0.093564668	0.998825	Alpha6 subunit of the 20S proteasome; regulated by Gcn4; induced in response to amino acid starvation (3-AT); Spider biofilm repressed
orf19.2665	MSN5	265.09	301.67	257.04	280.8	-0.079566562	0.998825	Predicted karyopherin involved in nuclear import and export of proteins; flow model biofilm induced; Spider biofilm induced

orf19.7613	HCR1	299.47	301.13	292.71	313.29	0.002455959	0.998825	Putative translation initiation factor; repressed upon phagocytosis by murine macrophage; Spider biofilm repressed
orf19.3122.2	orf19.3122.2	317.79	299.94	353.79	380.13	0.216083232	0.798341	Ortholog(s) have role in exocytosis, filamentous growth, mRNA export from nucleus, proteasome assembly, proteasome-mediated ubiquitin-dependent protein catabolic process, regulation of cell cycle
orf19.6220.3	MMD1	283.78	299.14	400.15	447.03	0.477660568	0.079162	Mitochondrial protein; possibly required for transamination of isoleucine; macrophage-downregulated protein abundance; rat catheter and Spider biofilm repressed
orf19.5285	PST3	265.94	299.03	410.21	452.42	0.538727996	0.035125	Putative flavodoxin; YNB biofilm induced; stationary phase enriched protein; rat catheter and Spider biofilm repressed
orf19.3076	orf19.3076	367.11	298.39	411.08	333	0.135360006	0.987362	Ortholog(s) have role in vesicle-mediated transport and COPI-coated vesicle, integral to Golgi membrane localization
orf19.1957	CYC3	306.36	298.11	250.59	237.34	-0.288849311	0.568429	Mitochondrial cytochrome c heme lyase; antigenic cell-wall protein; filament induced; induced on polystyrene adherence, interaction with macrophage; N-glycosylation, 2 heme-binding motifs; rat catheter, Spider biofilm induced
orf19.6828.1	orf19.6828.1	338.09	297.58	444.28	488.57	0.496368679	0.048371	Ortholog(s) have P-P-bond-hydrolysis-driven protein transmembrane transporter activity, structural molecule activity
orf19.2242	PRB1	323.16	297.43	232.57	223.01	-0.4093574	0.21299	Endoprotease B; regulated by heat, carbon source (GlcNAc-induced), nitrogen, macrophage response, human neutrophils; similar to (does not replace) S. cerevisiae vacuolar B protease Prb1p; flow model biofilm induced; Spider biofilm induced
orf19.2643	RPO26	298.03	297.09	219.4	256.53	-0.29581702	0.580563	Putative RNA polymerase subunit; heterozygous null mutant exhibits resistance to parnafungin in the C. albicans fitness test

orf19.7421	CYP5	334.57	296.9	343.74	345.46	0.103851045	0.998825	Putative peptidyl-prolyl cis-trans isomerase; macrophage-downregulated protein level; protein level decrease in stationary phase cultures; predicted endoplasmic reticulum (ER) localization
orf19.172	RPC19	318.66	296.84	216.57	291.47	-0.250936965	0.777732	Putative RNA polymerases I and III subunit AC19; Hap43-induced; rat catheter biofilm induced
orf19.2489	orf19.2489	289.82	296.28	295.04	300.12	0.008393807	0.998825	Putative karyopherin beta; repressed by nitric oxide
orf19.1915	MPP10	299.52	296.03	248.28	237.29	-0.279338267	0.600579	Putative SSU processome and 90S preribosome component; repressed in core stress response; repressed by prostaglandins
orf19.5021	PDX1	292.71	295.88	482.32	458.87	0.606361012	0.005707	Pyruvate dehydrogenase complex protein X; essential component of the mitochondrial pyruvate dehydrogenase complex; role in the respiratory pathway; protein present in exponential and stationary growth phase yeast; Spider biofilm repressed
orf19.3364	orf19.3364	344.7	295.16	63.87	59.12	-2.040036608	3.74E-21	Ortholog of C. parapsilosis CDC317 : CPAR2_403360, Debaryomyces hansenii CBS767 : DEHA2D00814g, Pichia stipitis Pignal : PICST_32156 and Candida guilliermondii ATCC 6260 : PGUG_04611
orf19.1375	LEU42	288.72	294.68	320.43	348.11	0.164674818	0.930538	Putative alpha-isopropylmalate synthase; fungal-specific; induced by human blood or polymorphonuclear cells; regulated by Gcn2 and Gcn4; stationary phase enriched protein; Spider biofilm induced
orf19.1363	orf19.1363	256.88	294.63	144.2	175.04	-0.698354971	0.009893	Putative protein of unknown function; Plc1-regulated; transcript induced by Mnl1 under weak acid stress; flow model, rat catheter, Spider biofilm induced
orf19.1339	CPY1	313.62	293.09	425.35	434.69	0.449259539	0.091064	Carboxypeptidase Y; transcript regulated at yeast-hypha transition or macrophage response; induced human neutrophils; regulated by Gcn2 and Gcn4; putative N-glycosylation

orf19.3423	TIF3	274.48	292.66	299.88	293.2	0.045503388	0.998825	Putative translation initiation factor; genes encoding ribosomal subunits, translation factors, and tRNA synthetases are downregulated upon phagocytosis by murine macrophage
orf19.2168.3	orf19.2168.3	314.6	292.43	280.18	282.65	-0.108146155	0.998825	Ortholog(s) have role in cellular protein localization, cytokinesis, site selection, endoplasmic reticulum inheritance, endoplasmic reticulum tubular network organization, nuclear pore complex assembly, vesicle-mediated transport
orf19.7477	YRB1	323.81	292.35	316.63	308.96	0.010951283	0.998825	Functional homolog of S. cerevisiae Yrb1p; regulates Gsp1 GTPase activity and thereby affects nucleocytoplasmic transport and cytoskeletal dynamics; transcript is not regulated by white-opaque switch or by dimorphic transition
orf19.158	orf19.158	337.49	291.68	197.6	212.35	-0.559649871	0.035362	Ortholog of S. cerevisiae Apd1; required for normal localization of actin patches and normal tolerance of sodium ions and hydrogen peroxide; Hap43-induced; Spider biofilm induced
orf19.4040	ILV3	292.42	290.42	324.15	347.93	0.177443542	0.893221	Dihydroxyacid dehydratase; repressed by nitric oxide; macrophage-induced protein; protein in exponential and stationary growth phase; Sef1, Sfu1p, Hap43-regulated; farnesol-repressed; flow model biofilm induced; Spider biofilm repressed
orf19.7459	orf19.7459	240.29	290.22	193.36	216.55	-0.33694785	0.517955	Putative mitochondrial protein with a predicted role in respiratory growth; fluconazole-induced; ketoconazole-repressed; mutants display a strong defect in flow model biofilm formation; Spider biofilm induced
orf19.5823	SGT2	342.68	290.16	316.53	305.13	-0.03217455	0.998825	Putative small tetratricopeptide repeat (TPR)-containing protein; protein abundance is affected by URA3 expression in the CAI-4 strain background; Mig1-regulated
orf19.1575	PRS1	306.14	289.76	273.44	268.22	-0.136922852	0.98704	Phosphoribosylpyrophosphate synthetase; enzyme of purine, pyrimidine, histidine, and tryptophan biosynthesis; essential; flucytosine induced; macrophage/pseudohyphal-induced
orf19.2661	TLO34	297.53	288.94	476.98	520.26	0.688189949	0.00117	Non-telomeric member of a family of telomere-proximal genes of unknown function; predicted N-terminus is extended, relative to that of other family members; overlaps orf19.2662

orf19.4371	TAL1	273.48	288.92	482.93	559.36	0.797593128	0.000147	Transaldolase; protein present in exponential and stationary growth phase yeast cultures; oxidative stress-induced via Cap1; induced by nitric oxide independent of Yhb1p; sumoylation target; rat catheter biofilm repressed
orf19.1760	RAS1	335.04	287.97	344.93	281.33	-0.005721184	0.998825	RAS signal transduction GTPase; regulates cAMP and MAP kinase pathways; role in hyphal induction, virulence, apoptosis, heat-shock sensitivity; nonessential; plasma membrane-localized; complements viability of S. cerevisiae ras1 ras2 mutant
orf19.5061	ADE5,7	315.04	287.35	339.17	350.23	0.167372942	0.916335	Phosphoribosylamine-glycine ligase and phosphoribosylformylglycinamidine cyclo-ligase; interacts with Vps34p; required for hyphal growth and virulence; flucytosine induced; not induced in GCN response, in contrast to S. cerevisiae ortholog
orf19.2755	orf19.2755	330.72	287.1	328.8	334.31	0.083102877	0.998825	Ortholog(s) have role in proteasomal ubiquitin-independent protein catabolic process, proteasome-mediated ubiquitin-dependent protein catabolic process and cytosol, nucleus, proteasome core complex, beta-subunit complex localization
orf19.1357	FCY21	280.6	286.64	244.4	250.28	-0.191958091	0.878538	High affinity, high capacity, hypoxanthine-adenine-guanine-cytosine/H+ symporter; similar to S. cerevisiae Fcy2; mutation confers resistance to 5-fluorocytosine (5-FC); flow model biofilm induced
orf19.1837	TBP1	290.46	286.35	262.79	263.72	-0.126590644	0.998825	Transcription initiation factor; binds TATA box sequence, binding does not require TFIIA; caspofungin repressed; functional homolog of S. cerevisiae and human TATA-binding proteins; Spider biofilm induced
orf19.2451	PGA45	283.79	285.57	277.8	313.03	0.037203943	0.998825	Putative GPI-anchored cell wall protein; repressed in core caspofungin response; Hog1-induced; regulated by Ssn6; Mob2-dependent hyphal regulation; flow model biofilm induced
orf19.4961	STP2	272.97	285.24	256.94	232.15	-0.184137081	0.893138	Amino-acid-regulated transcription factor; activates transcription of amino acid permease genes; activated by amino-acid-induced proteolytic processing (Ssy1, Csh3 dependent); required for alkalinization of medium; Spider biofilm induced
orf19.1614	MEP1	270.62	285.09	190.54	221.23	-0.392548381	0.306039	Ammonium permease; Mep1 more efficient permease than Mep2, Mep2 has additional regulatory role; 11 predicted transmembrane regions; low mRNA abundance; hyphal downregulated; flow model biofilm induced

orf19.6213	SUI2	298.81	284.66	245.52	232.65	-0.268505319	0.639737	Translation initiation factor eIF2, alpha chain; genes encoding ribosomal subunits, translation factors, and tRNA synthetases are downregulated upon phagocytosis by murine macrophage; stationary phase enriched protein
orf19.3810	orf19.3810	287.66	284.65	276.24	318.11	0.037557502	0.998825	Ortholog(s) have methylenetetrahydrofolate dehydrogenase (NAD+) activity, role in folic acid-containing compound biosynthetic process, one-carbon metabolic process, purine nucleobase biosynthetic process and cytosol, nucleus localization
orf19.4029	orf19.4029	321.91	284.45	244	227.58	-0.334467265	0.417295	Ortholog(s) have role in ribosomal large subunit assembly and cytosolic ribosome, nucleus localization
orf19.2480.1	AUT7	334.77	284.33	354.83	295.22	0.05222913	0.998825	Putative autophagosome protein; macrophage/pseudohyphal-repressed; alternatively spliced intron in 5' UTR; Spider biofilm induced
orf19.1873	orf19.1873	328.1	283.93	334.55	342.93	0.125303138	0.998825	Protein of unknown function; Hap43-repressed gene; repressed by nitric oxide
orf19.6474	orf19.6474	273.87	283.09	282.05	268.74	-0.02662512	0.998825	Protein with chitin synthesis regulation, resistance to Congo red domain; membrane-localized protein; Spider biofilm induced
orf19.7469	ARG1	227.74	282.25	133.26	171.83	-0.644746211	0.033604	Argininosuccinate synthase; arginine synthesis; Gcn4, Rim101 regulated; induced by amino acid starvation (3-AT), benomyl treatment; stationary phase enriched protein; repressed in alkalinizing medium; rat catheter, Spider biofilm induced
orf19.7081	SPL1	282.93	280.93	266.84	253.16	-0.117494833	0.998825	Protein similar to S. cerevisiae Spl1p, which is involved in tRNA splicing; member of pyridoxal-phosphate-dependent aminotransferase protein family; predicted to be essential
orf19.6531	NUC2	305.99	280.71	311.51	310.19	0.06555499	0.998825	Putative NADH-ubiquinone oxidoreductase; identified in detergent-resistant membrane fraction (possible lipid raft component); alkaline repressed; Hap43-repressed; Spider biofilm repressed

orf19.5640	PEX5	284.75	280.58	257.25	274.91	-0.090394662	0.998825	Pex5p family protein; required for PTS1-mediated peroxisomal protein import, fatty acid beta-oxidation; similar to S. cerevisiae Pas10p peroxisomal targeting receptor; macrophage/pseudohyphal-repressed; Hap43p-repressed
orf19.5550	MRT4	324.87	279.94	269.63	284.67	-0.120227321	0.998825	Putative mRNA turnover protein; Hap43-induced; mutation confers hypersensitivity to tubercidin (7-deazaadenosine); rat catheter biofilm induced
orf19.5248	MSO1	284.21	279.51	314.05	286.37	0.068138755	0.998825	Putative secretory protein involved in S. cerevisiae sporulation; repressed during pseudohyphal growth in the presence of lysed macrophages; Hap43-repressed; Spider biofilm induced
orf19.238	CCP1	286.96	279.35	332.67	290.64	0.114317984	0.998825	Cytochrome-c peroxidase N terminus; Rim101, alkaline pH repressed; induced in low iron or by macrophage interaction; oxygen-induced activity; regulated by Sef1, Sfu1, and Hap43; Spider biofilm induced; rat catheter biofilm repressed
orf19.6561	LAT1	277.39	278.98	493.88	467.72	0.711190591	0.000554	Putative dihydrolipoamide acetyltransferase component (E2) of pyruvate dehydrogenase complex; sumoylation target; Spider biofilm repressed
orf19.5627	orf19.5627	286.03	278.63	281.2	296.97	0.019429781	0.998825	S. cerevisiae ortholog Hek2/Khd1 is a putative RNA binding protein involved in the asymmetric localization of ASH1 mRNA; Hap43-induced gene
orf19.4941	TYE7	279.52	278.48	250.66	277.69	-0.078669012	0.998825	bHLH transcription factor; control of glycolysis; required for biofilm formation; hyphally regulated by Cph1, Cyr1; flucytosine, Hog1 induced; amphotericin B, caspofungin repressed; induced in flow model biofilm and planktonic cultures
orf19.734	GLK1	246.9	278.4	455.99	469.9	0.729436846	0.000764	Putative glucokinase; transcript regulated upon yeast-hyphal switch; Efg1 regulated; fluconazole-induced; induced in core stress response; colony morphology-related gene regulation by Ssn6; GlcNAc-induced protein
orf19.5793	PR26	331.64	278.06	276.66	287.55	-0.107486856	0.998825	Protein with similarity to proteasomal 26S regulatory subunit of S. cerevisiae, H. sapiens, Methanobacterium thermoautotrophicum (Archaebacterium)

orf19.131.2	orf19.131.2	254.06	277.85	268.49	296.82	0.066333716	0.998825	Ortholog(s) have endoplasmic reticulum localization
orf19.3696	TOM22	272.49	277.62	292.19	345.5	0.180508773	0.90951	Putative mitochondrial import receptor subunit; colony morphology-related gene regulation by Ssn6
orf19.2699	ABP1	276.75	277.41	397.74	356.29	0.390901317	0.216469	Ortholog of S. cerevisiae Abp1; actin-binding protein of the cortical actin cytoskeleton; caspofungin induced; protein only detected in stationary phase yeast-form cultures; Spider biofilm repressed
orf19.7322	orf19.7322	292.76	277.06	268.86	302.93	-0.004114038	0.998825	Protein of unknown function; S. cerevisae ortholog Ypl225w interacts with ribosomes; rat catheter biofilm induced
orf19.7384	NOG1	281.71	276.89	244.25	228.19	-0.230990532	0.789669	Putative GTPase; mutation confers hypersensitivity to 5-fluorocytosine (5-FC), 5-fluorouracil (5-FU), and tubercidin (7-deazaadenosine); repressed by prostaglandins; Hap43-induced
orf19.6749	KRS1	264.23	276.61	300.43	302.87	0.129006096	0.998825	Putative tRNA-Lys synthetase; repressed upon phagocytosis by murine macrophages; stationary phase enriched protein; Spider biofilm repressed
orf19.269	SES1	262.47	276.2	290.97	287.18	0.080707404	0.998825	Seryl-tRNA synthetase; charges the tRNA that recognizes the CUG codon, which typically specifies Leu, but specifies Ser in C. albicans; complements S. cerevisiae ses1 mutant viability; soluble protein in hyphae; macrophage-regulated
orf19.1114	orf19.1114	259.66	275.13	292.12	284.43	0.083175443	0.998825	Cytochrome c oxidase subunit; membrane-localized protein; rat catheter biofilm induced
orf19.7590	orf19.7590	298.99	274.79	296.14	292.25	0.021176796	0.998825	Putative NADH-ubiquinone oxidoreductase; identified in detergent-resistant membrane fraction (possible lipid raft component); predicted N-terminal acetylation; repressed by nitric oxide

orf19.2667	RPF1	280.25	273.99	223.52	234.85	-0.253012598	0.720423	Putative nucleolar protein with a predicted role in the assembly and export of the large ribosomal subunit; essential for growth; rat catheter and Spider biofilm induced
orf19.7312	ERG13	247.36	273.85	363.73	381.26	0.452810741	0.123707	3-hydroxy-3-methylglutaryl coenzyme A synthase; ergosterol biosynthesis; sumoylation target; Tn mutation affects filamentation; amphotericin B, caspofungin repressed; exponential, stationary growth phase expressed; Spider biofilm repressed
orf19.5446	orf19.5446	252.24	273.79	326.13	252.62	0.107046647	0.998825	Putative protein of unknown function; transcript is upregulated in clinical isolates from HIV+ patients with oral candidiasis; regulated by Ssn6
orf19.2031	VPS24	263.56	272.04	199.56	186.93	-0.430286972	0.207795	Protein similar to S. cerevisiae Vps24p, which is a member of the ESCRT III protein sorting complex; downregulated upon adherence to polystyrene
orf19.6524	TOM40	300.45	271.89	359.76	314.96	0.204672721	0.842252	Protein involved in mitochondrial protein import; Spider biofilm repressed
orf19.7605	PUP1	346.19	270.63	308.91	277.97	-0.073612631	0.998825	Putative beta 2 subunit of the 20S proteasome; macrophage/pseudohyphal-repressed; Spider biofilm repressed
orf19.6601.1	YKE2	239.69	269.89	385.88	448.42	0.625484534	0.010947	Possible heterohexameric Gim/prefoldin protein complex subunit; role in folding alpha-tubulin, beta-tubulin, and actin; transcript induced by yeast-to-hypha switch; regulated by Nrg1, Tup1; Spider and flow model biofilm induced
orf19.385	GCV2	289.51	269.85	227.07	185.57	-0.404005214	0.256424	Glycine decarboxylase P subunit; protein of glycine catabolism; repressed by Efg1; Hog1-induced; induced by Rim101 at acid pH; transcript induced in elevated CO2; stationary phase enriched protein
orf19.2953	TOM20	265.51	268.44	289.35	290.72	0.095995093	0.998825	Putative mitochondrial primary import receptor

orf19.1704	FOX3	290.55	267.98	388.38	376.69	0.401410788	0.188907	Putative peroxisomal 3-oxoacyl CoA thiolase; transcript regulated by white-opaque switch; Spider biofilm induced
orf19.4384	HXT5	189.07	267.93	357.37	431.34	0.670210808	0.015023	Putative sugar transporter; induced by ciclopirox olamine; Snf3-induced; alkaline repressed; colony morphology-related gene regulation by Ssn6; possibly essential gene
orf19.6165	KGD1	254.4	267.84	253.47	248.57	-0.062797168	0.998825	Putative 2-oxoglutarate dehydrogenase; regulated by Efg1 under yeast but not hyphal growth conditions; transcript induced in an RHE model of oral candidiasis; stationary phase enriched protein; Hap43-repressed; rat catheter biofilm induced
orf19.1862	orf19.1862	192.27	267.65	195.6	245.48	-0.064725183	0.998825	Possible stress protein; increased transcription associated with CDR1 and CDR2 overexpression or fluphenazine treatment; regulated by Sfu1, Nrg1, Tup1; stationary phase enriched protein; Spider biofilm induced
orf19.3713	orf19.3713	318.73	267.53	194.06	222.7	-0.444025451	0.189613	Protein of unknown function; flow model biofilm induced; Spider biofilm induced; induced by Mnl1 under weak acid stress; transcript detected in high-resolution tiling arrays
orf19.6710	UTP9	287.09	266.47	232.7	242.61	-0.203282446	0.861125	Small-subunit processome protein; Ssr1-induced; repressed by prostaglandins; physically interacts with TAP-tagged Nop1
orf19.3611	orf19.3611	272.71	265.14	266.18	285.08	0.021096828	0.998825	Protein of unknown function; Hap43-repressed gene; repressed by nitric oxide
orf19.3861	SIS1	271.98	265.13	243.21	225.99	-0.186933925	0.89191	Putative Type II HSP40 co-chaperone; macrophage/pseudohyphal-repressed; heavy metal (cadmium) stress-induced; heterozygous null mutant displays sensitivity to virgineone; rat catheter biofilm induced
orf19.4060	ARO4	273.63	263.92	267.51	278.65	0.01136385	0.998825	3-deoxy-D-arabinoheptulosonate-7-phosphate synthase; aromatic amino acid biosynthesis; GCN-regulated; feedback-inhibited by tyrosine if produced in S. cerevisiae Aro3p and Aro4p catalyze same reaction; protein decreases in stationary phase

orf19.5338	GAL4	218.41	263.88	439.51	540.78	0.900899583	5.35E-05	Zn(II)2Cys6 transcription factor; involved in control of glycolysis; ortholog of S. cerevisiae Gal4, but not involved in regulation of galactose utilization genes; caspofungin repressed; Spider biofilm repressed
orf19.6099	CCT8	268.64	263.85	265.63	252.71	-0.046266412	0.998825	Chaperonin-containing T-complex subunit; role in hyphal morphogenesis, particularly starvation-induced; essential; expression in S. cerevisiae inhibits Ras2-mediated pathways; CCT8 and TRP1 overlap; Spider biofilm repressed
orf19.4651	PGA53	205	263.61	410.32	534.24	0.874428401	0.000265	GPI-anchored cell surface protein of unknown function; greater mRNA abundance observed in a cyr1 homozygous null mutant than in wild type
orf19.3869	orf19.3869	292.01	263.57	224.77	179.86	-0.418718994	0.235084	Protein of unknown function; regulated by Tsa1, Tsa1B in minimal media at 37 degrees C; shows colony morphology-related gene regulation by Ssn6; Spider biofilm induced
orf19.6096	TRP1	267.77	263.17	294.88	308.79	0.157046598	0.944705	Phosphoribosylanthranilate isomerase; tryptophan biosynthesis; expected unifunctional, unlike trifunctional enzyme of some other fungi; complements E. coli trpC, S. cerevisiae trp1 mutant; CCT1 and TRP1 overlap; Spider biofilm repressed
orf19.6763	SLK19	256.81	262.49	334.25	300.97	0.24935767	0.701828	Alkaline-induced protein of plasma membrane; affects cell aggregation, cell wall; similar to S. cerevisiae Slk19p (a kinetochore protein with roles in mitosis, meiosis); required for wild-type virulence in mouse; macrophage-downregulated
orf19.2613	ECM4	278.43	262.01	448.71	435.95	0.639762395	0.003241	Cytoplasmic glutathione S-transferase; regulated by Nrg1, Tup1; induced in core stress response, in cyr1 or ras1 mutant (yeast or hyphal cells); Tn mutation affects filamentous growth; stationary phase enriched; Spider biofilm induced
orf19.7123	orf19.7123	225.26	261.45	242.95	224.15	-0.065618863	0.998825	Protein of unknown function; Spider biofilm repressed
orf19.2091	orf19.2091	274.01	261.38	258.95	242.68	-0.093960374	0.998825	Putative NADH dehydrogenase; repressed by nitric oxide, Hap43p-repressed

orf19.6648	SDA1	295.31	261.35	204.51	203.03	-0.407795292	0.240742	Predicted nuclear protein involved in actin cytoskeleton organization, passage through Start, 60S ribosome biogenesis; rat catheter biofilm induced; Hap43-induced
orf19.5917.3	orf19.5917.3	241.84	261.11	264.7	235.48	-0.021880712	0.998825	RNA binding protein required for export of poly(A)+ mRNA from the nucleus; Spider biofilm repressed
orf19.3034	RLI1	247.66	259.97	242.64	245.29	-0.063635834	0.998825	Member of RNase L inhibitor (RLI) subfamily of ABC family; predicted not to be a transporter; regulated by Sef1p, Sfu1p, and Hap43p
orf19.2435	MSI3	270.6	259.53	277.32	261.42	0.005449644	0.998825	Essential HSP70 family protein; required for fluconazole resistance and calcineurin-dependent transcription; interacts with Cgr1; transcript regulated by iron; rat catheter biofilm induced; farnesol repressed in biofilm; sumoylation target
orf19.6177	orf19.6177	258.65	259.18	314.65	290.21	0.18937801	0.878136	Protein of unknown function; Spider biofilm repressed
orf19.1075.1	orf19.1075.1	282.81	257.85	275.85	281.91	0.030620682	0.998825	Pseudogene; added to Assembly 21 based on comparative genome analysis
orf19.6014	RRS1	273.33	256.99	237.7	256.58	-0.097364647	0.998825	Putative ribosome biogenesis and nuclear export protein; Hap43p-induced gene; mutation confers hypersensitivity to 5-fluorocytosine (5-FC), 5-fluorouracil (5-FU), and tubercidin (7-deazaadenosine)
orf19.6554	orf19.6554	238.73	256.31	222.07	199.3	-0.220998646	0.846842	Regulator of calcineurin; regulated by calcineurin-Crz1 pathway; feedback regulator of calcineurin-dependent signaling; Hap43-repressed; induced by ketoconazole, hypoxia, during growth in the mouse cecum; Spider biofilm induced
orf19.7271	orf19.7271	269.2	254.43	267.84	255.62	-0.008609729	0.998825	Protein of unknown function; transcript detected on high-resolution tiling arrays

orf19.4150	orf19.4150	242.41	253.16	171.89	184	-0.429693132	0.23703	Putative glutaredoxin; induced by nitric oxide; Spider biofilm induced
orf19.6637	orf19.6637	291.84	252.93	255.81	201.67	-0.235409935	0.797	Predicted glycosyl hydrolase; hypoxia induced; flow model biofilm induced
orf19.2533.1	orf19.2533.1	272.71	252.41	347.52	323.62	0.311295915	0.468439	Protein with a predicted role in protein translocation from the endoplasmic reticulum
orf19.3297	orf19.3297	226.42	251.64	252.38	229.69	-0.003546593	0.998825	Ortholog(s) have structural constituent of ribosome activity and mitochondrial small ribosomal subunit localization
orf19.2709	ZUO1	264.55	251.64	255.12	240.18	-0.067635982	0.998825	Ortholog of S. cerevisiae Zuo1; a cytosolic ribosome-associated chaperone; likely to be essential for growth, based on an insertional mutagenesis strategy; Spider biofilm repressed
orf19.1107	orf19.1107	271.28	250.56	251.56	197.04	-0.206452601	0.87024	Protein of unknown function; Spider biofilm induced
orf19.7487	orf19.7487	246.02	249.99	274.49	290.94	0.157685328	0.946319	Has domain(s) with predicted RNA binding, RNA-directed DNA polymerase activity and role in RNA-dependent DNA replication
orf19.5118	SDS24	227.38	249.78	215.99	193.37	-0.210240131	0.87023	Protein similar to S. cerevisiae Sds24 involved in cell separation during budding; transcript regulated by Mig1 and Tup1; fluconazole-induced; flow model biofilm induced
orf19.1480	orf19.1480	259.67	249.66	278.02	278.98	0.103676226	0.998825	Putative succinate dehydrogenase; enzyme of citric acid cycle; repressed by nitric oxide; Efg1, Hap43 repressed

orf19.6063	UBP6	276.27	248.41	251.33	259.49	-0.044168179	0.998825	Putative ubiquitin-specific protease of the 26S proteasome; oxidative stress-induced via Cap1p
orf19.6840	orf19.6840	291.04	247.93	170.87	144.68	-0.689039842	0.007446	Protein of unknown function; transcript detected in high-resolution tiling arrays; transcription induced by alpha pheromone in SpiderM medium; Spider and early-stage flow model biofilm induced
orf19.203	STB3	177.99	247.86	164.36	209.59	-0.170869435	0.97752	Putative SIN3-binding protein 3 homolog; caspofungin induced; macrophage/pseudohyphal-repressed; rat catheter biofilm induced
orf19.5364	orf19.5364	222.07	247.3	178.21	193.43	-0.308088846	0.596073	Putative pre-mRNA splicing factor; intron in 5'-UTR; possibly an essential gene, disruptants not obtained by UAU1 method
orf19.603	IMP4	233.05	247.22	166.43	166.79	-0.475315133	0.155601	Putative SSU processome component; Hap43-induced; repressed by prostaglandins; Spider biofilm induced
orf19.374	orf19.374	258.62	247.2	276.42	250.99	0.04071199	0.998825	Ortholog(s) have role in protein targeting to vacuole involved in ubiquitin-dependent protein catabolic process via the multivesicular body sorting pathway
orf19.7422	orf19.7422	277.1	246.95	210.34	200.03	-0.325419432	0.486965	Ortholog(s) have RNA binding activity and nucleus localization
orf19.7247	RIM101	197.19	246.88	276.53	252.23	0.206455691	0.878136	Transcription factor; alkaline pH response; required for alkaline-induced hyphal growth; role in virulence in mice; activated by C-terminal proteolytic cleavage; mediates both positive and negative regulation; Spider biofilm induced
orf19.5137.1	HHO1	260.69	245.54	281.45	240.92	0.025099086	0.998825	Putative histone H1; farnesol regulated; Hap43-induced; contains 5' UTR intron; Spider biofilm repressed

orf19.6387	HSP104	245.82	245.46	246.25	247.35	-0.005906688	0.998825	Heat-shock protein; roles in biofilm and virulence; complements chaperone, prion activity in S. cerevisiae; guanidine-insensitive; heat shock/stress induced; repressed in farnesol-treated biofilm; sumoylation target; Spider biofilm induced
orf19.3123	RPT5	273.94	244.92	266.71	251.73	-0.010388814	0.998825	26S proteasome regulatory subunit; transcript regulated by Nrg1 and Mig1; regulated by Gcn2 and Gcn4; protein level decreases in stationary phase
orf19.1233	ADE4	286.08	243.69	237.42	222.03	-0.19506425	0.878538	Putative phosphoribosylpyrophosphate amidotransferase; flucytosine induced
orf19.4682	HGT17	290.81	243.53	216.61	180.12	-0.392251692	0.301253	Putative MFS family glucose transporter; 20 members in C. albicans; 12 probable membrane-spanning segments; induced at low (0.2%, compared to 2%) glucose in rich media; Spider biofilm induced
orf19.4025	PRE1	261.08	242.32	245.35	239.02	-0.060171323	0.998825	Putative beta 4 subunit of the 20S proteasome; Rim101-induced at acid pH; flucytosine induced; amphotericin B repressed
orf19.2803	HEM13	189.57	240.57	269.26	346.38	0.434149928	0.268336	Coproporphyrinogen III oxidase; antigenic; on yeast cell surface, not hyphae; iron-regulated expression; Hap43, macrophage-repressed; farnesol-induced; possibly essential; flow model biofilm induced; rat catheter, Spider biofilm repressed
orf19.2398	orf19.2398	188.61	240.34	194.89	213.18	-0.076157926	0.998825	Protein of unknown function; Hap43-repressed; transcript increased in azole-resistant strain overexpressing CDR1 and CDR2; possibly regulated by Tac1; induced by Mnl1 in weak acid stress; flow model biofilm induced; Spider biofilm induced
orf19.338	orf19.338	238.19	240.1	444.42	431.49	0.78114864	0.000163	Putative glycoside hydrolase; stationary phase enriched protein; Hog1p-downregulated; shows colony morphology-related gene regulation by Ssn6p
orf19.4536	CYS4	254.28	238.95	272.41	249.36	0.059826903	0.998825	Cystathionine beta-synthase; sulfur amino acid biosynthesis; antigenic in mouse; flow model biofilm induced; alkaline induced; macrophage/pseudohyphal-induced; present in exponential and stationary growth phase yeast cultures

orf19.6955	HBR3	248	238.32	199.25	212.96	-0.220856283	0.846842	Essential protein; regulated by hemoglobin; S. cerevisiae ortholog is essential; Hap43p-induced gene
orf19.4041	PEX4	238.32	238.23	176.12	201.98	-0.301542475	0.606662	Putative peroxisomal ubiquitin conjugating enzyme; regulated by Sef1, Sfu1, and Hap43; rat catheter biofilm induced; Spider biofilm induced
orf19.1963	GDS1	193.78	237.8	190.82	190.73	-0.168609332	0.953167	Putative mitochondrial protein; Hap43-repressed gene; Spider biofilm induced
orf19.922	ERG11	220.25	237.63	226.56	249.46	0.03814752	0.998825	Lanosterol 14-alpha-demethylase; cytochrome P450 family; role in ergosterol biosynthesis; target of azole antifungals; may contribute to drug resistance; azole or flow model biofilm induced; drug treated biofilm induced; hypoxia regulated
orf19.442	orf19.442	273.81	237.61	261.17	260.1	0.013397857	0.998825	Ortholog of Candida guilliermondii ATCC 6260 : PGUG_05525, Candida lusitaniae ATCC 42720 : CLUG_03378 and Candida albicans WO-1 : CAWG_00876
orf19.4102	RPN10	274.97	237.57	248.52	224.5	-0.112361568	0.998825	Putative 19S regulatory particle of the 26S proteasome; macrophage/pseudohyphal-repressed; regulated by Gcn2 and Gcn4; Spider biofilm repressed
orf19.7625	PGA1	222.87	237.31	250.75	231.65	0.049333302	0.998825	Putative GPI-anchored protein; induced during cell wall regeneration; required for normal adhesion to host cells and for adherence during Sabouraud biofilm formation; Spider biofilm induced
orf19.1494	RAD23	255.54	236.5	238.48	225.71	-0.088916198	0.998825	S. cerevisiae Rad23 ortholog; binds damaged DNA; Spider biofilm repressed
orf19.3358	LSC1	249.79	235.72	393.24	436.22	0.687658579	0.002174	Putative succinate-CoA ligase subunit; induced by high iron; fluconazole-induced; protein present in exponential and stationary growth phase yeast cultures; Spider biofilm repressed

orf19.4271	orf19.4271	216.82	235.59	218.92	263.11	0.068912221	0.998825	Predicted ORF from Assembly 19; removed from Assembly 20; subsequently reinstated in Assembly 21 based on comparative genome analysis
orf19.4230	orf19.4230	265.78	234.38	261.9	246.39	0.010320396	0.998825	20S proteasome subunit (beta7); protein present in exponential and stationary growth phase yeast cultures
orf19.3476	HRR25	234.08	234.13	200.08	198.4	-0.21832836	0.853836	Predicted protein serine/threonine kinase; Spider biofilm induced
orf19.5759	SNQ2	244.98	233.91	13.68	13.51	-3.045335908	3.28E-33	Protein similar to S. cerevisiae Snq2p transporter; member of PDR subfamily of ABC family; transposon mutation affects filamentation; benomyl-induced transcription; detected at yeast-form cell plasma membrane by mass spec
orf19.657	SAM2	200.11	233.57	312.37	356.8	0.541706347	0.059118	S-adenosylmethionine synthetase; localizes to surface of hyphae, not yeast cells; alkaline, Hog1-induced; farnesol-downregulated; F-12/CO2 early biofilm induced; Spider biofilm repressed
orf19.3612	PST2	227.84	233.1	290.92	299.86	0.309972535	0.523748	Putative NADH:quinone oxidoreductase; similar to 1,4-benzoquinone reductase; immunogenic in mice; induced by benomyl, oxidative stress via Cap1; fungal-specific; farnesol-repressed; Spider biofilm induced
orf19.3962	HAS1	257.83	231.49	193.32	197.69	-0.295000137	0.602635	Functional homolog of S. cerevisiae Has1p, which is a nucleolar protein of the DEAD-box ATP-dependent RNA helicase family that is involved in biogenesis of the ribosome, particularly the small (40S) subunit; caspofungin-downregulated
orf19.1225	PEX22	267.45	230.88	267.35	256.74	0.056204768	0.998825	Putative peroxin
orf19.4952	orf19.4952	215.02	230.85	196.17	188.45	-0.202974007	0.880321	Protein of unknown function; flow model biofilm induced; Spider biofilm induced

orf19.6250	orf19.6250	219.68	230.6	247.87	235.16	0.075242291	0.998825	Ortholog(s) have eukaryotic initiation factor 4G binding, mRNA binding activity
orf19.2622	YPT31	236.66	230.26	202.51	202.99	-0.189753135	0.894293	Protein required for resistance to toxic ergosterol analog
orf19.5649	orf19.5649	246.37	230.06	265.5	204.24	-0.03277357	0.998825	Ortholog of Candida lusitaniae ATCC 42720 : CLUG_00275 and Candida albicans WO-1 : CAWG_03759
orf19.918	CDR11	231.21	229.9	92.85	87.23	-1.172145875	7.85E-07	Putative transporter of PDR subfamily of ABC family; Gcn4-regulated; induced by Rim101 at pH 8; Spider biofilm induced
orf19.4758	orf19.4758	253.24	229.17	232.24	229.04	-0.067188638	0.998825	Putative reductase or dehydrogenase; Hap43-repressed gene; alkaline repressed
orf19.1549	orf19.1549	282.04	228.26	275.42	271.97	0.082329371	0.998825	Plasma membrane-associated protein identified in detergent-resistant membrane fraction (possible lipid raft component); repressed by nitric oxide; predicted transmembrane helix
orf19.6082	orf19.6082	229.9	228.03	255.69	237.1	0.082578395	0.998825	Ortholog(s) have unfolded protein binding activity, role in protein folding, protein localization to cell surface and integral to endoplasmic reticulum membrane, mitochondrial outer membrane localization
orf19.7498	LEU1	219.67	227.83	198.93	234.45	-0.052513067	0.998825	3-isopropylmalate dehydratase; antigenic in humans; repressed in hyphae; alkaline repressed; upregulated by human whole blood or PMNs; regulated by Sef1, Sfu1, and Hap43; rat catheter biofilm induced, Spider biofilm repressed
orf19.1220	RVS167	238.87	226.93	240.06	223.19	-0.019789429	0.998825	SH3-domain- and BAR domain-containing protein involved in endocytosis; null mutant exhibits defects in hyphal growth, virulence, cell wall integrity, and actin patch localization; cosediments with phosphorylated Myo5p

orf19.6132	orf19.6132	232.48	226.65	242.28	286.94	0.173070062	0.937109	Ortholog of C. dubliniensis CD36 : Cd36_33000, C. parapsilosis CDC317 : CPAR2_701740, Candida tenuis NRRL Y-1498 : CANTEDRAFT_93887 and Debaryomyces hansenii CBS767 : DEHA2E04554g
orf19.3140.1	orf19.3140.1	247.24	226.51	238.69	221.94	-0.046751998	0.998825	Ortholog(s) have role in cellular response to DNA damage stimulus, proteasome assembly, ubiquitin-dependent protein catabolic process and cytosol, nucleus localization
orf19.7085	orf19.7085	232.92	226.46	162.68	132.96	-0.565242161	0.059858	Protein of unknown function; induced in core stress response; induced by cadmium stress via Hog1; oxidative stress-induced via Cap1; induced by Mnl1 under weak acid stress; macrophage-repressed; rat catheter and Spider biofilm induced
orf19.377	PHR3	247.24	226.28	238.83	225.14	-0.035439738	0.998825	Putative beta-1,3-glucanosyltransferase with similarity to the A. fumigatus GEL family; fungal-specific (no human or murine homolog); possibly an essential gene, disruptants not obtained by UAU1 method
orf19.1388	orf19.1388	246.8	225.05	193.87	189.4	-0.2769486	0.66731	Putative 66S pre-ribosomal particle component; Hap43-induced; F-12/CO2 early biofilm induced
orf19.5647	SUB2	246.28	224.95	283.14	260.96	0.175267129	0.918336	Putative TREX complex component with a predicted role in nuclear mRNA export; transcription is regulated by Mig1; Hap43-induced gene; Spider biofilm repressed
orf19.3340	SOD2	225.01	224.93	182.94	176.62	-0.295216952	0.614758	Mitochondrial Mn-containing superoxide dismutase; protection against oxidative stress; homotetramer active; N-terminal 34 amino acids removed on mitochondrial import; H2O2-induced via Cap1p; Hap43p-, alkaline-downregulated, farnesol-induced
orf19.5641	CAR2	247.4	224.79	244.6	183.5	-0.137983534	0.998825	Ornithine aminotransferase; arginine metabolism; alkaline induced; mutant sensitivite to toxic ergosterol analog, to amphotericin B; exponential and stationary phase yeast; flow model biofilm induced; rat catheter, Spider biofilm repressed
orf19.3300	ZPR1	245.45	224.31	169.06	175.16	-0.402925105	0.293791	Protein with putative zinc finger; regulated by Gcn4p; repressed in response to amino acid starvation (3-aminotriazole treatment); upregulation correlates with clinical development of fluconazole resistance

orf19.6316.4	orf19.6316.4	229.03	224.22	163.26	148.28	-0.487465636	0.140986	Protein of unknown function; Hap43-induced; rat catheter and Spider biofilm induced
orf19.4937	CHS3	197.79	223.42	218.22	242.39	0.100846158	0.998825	Major chitin synthase of yeast and hyphae; synthesizes short-chitin fibrils; Chs4-activated; transcript induced at yeast-hyphal transition; Chs1 and Chs2, but not Chs3, are inhibited by the protoberberine HWY-289; Spider biofilm induced
orf19.6748	orf19.6748	236.67	223.21	194.85	207.14	-0.181307227	0.921909	Ortholog(s) have role in ascospore formation, cellular response to cadmium ion, detoxification of cadmium ion
orf19.3599	TIF4631	203.27	222.95	193.09	196.88	-0.124037511	0.998825	Putative translation initiation factor eIF4G; overexpression causes hyperfilamentation; hyphal- and macrophage-induced; genes encoding some translation factors are downregulated upon phagocytosis by murine macrophage
orf19.2774	LAB5	233.36	221.96	206.3	196.53	-0.166179481	0.946319	Ortholog(s) have role in protein lipoylation and mitochondrion localization
orf19.7258	DDI1	237.55	221.54	228.97	232.92	-0.004330179	0.998825	Putative DNA damage inducible v-SNARE binding protein; macrophage/pseudohyphal-repressed
orf19.36.1	orf19.36.1	172.24	221.06	232.21	351.86	0.473867544	0.252695	Ortholog of C. parapsilosis CDC317 : CPAR2_104640 and Candida orthopsilosis Co 90-125 : CORT_0B05695
orf19.7618	orf19.7618	260.7	220.6	200.25	194.9	-0.263153709	0.71836	Putative nucleolar protein with a predicted role in pre-18S rRNA processing; Plc1p-regulated; Spider biofilm induced
orf19.2965	orf19.2965	234.54	220.52	281.51	297.05	0.298971299	0.557651	Putative guanyl-nucleotide exchange factor; Spider biofilm repressed

orf19.909.1	orf19.909.1	278	220.2	317.54	294.37	0.257805765	0.692027	Ortholog of C. dubliniensis CD36 : Cd36_17965, C. parapsilosis CDC317 : CPAR2_211700, Candida tenuis NRRL Y-1498 : CANTEDRAFT_112898 and Debaryomyces hansenii CBS767 : DEHA2G05588g
orf19.441	RPT1	239.19	220.07	220.54	220.29	-0.061292435	0.998825	Putative 26S proteasome regulatory subunit 7; Hap43p-repressed gene; regulated by Gcn2p and Gcn4p; overlaps orf19.442
orf19.3369	MOH1	176.03	219.96	104.09	125.13	-0.674738204	0.031908	Ortholog of S. cerevisiae Moh1, essential for staionary phase growth; induced by alpha pheromone in SpiderM medium and by Mnl1 under weak acid stress; possibly essential (UAU1 method); flow model biofilm induced; Spider biofilm induced
orf19.4871	ERO1	188.3	219.85	127.46	133.03	-0.569353119	0.081502	Ortholog of S. cerevisiae Ero1; role in formation of disulfide bonds in the endoplasmic reticulum; fluconazole-induced; induced by Mnl1 under weak acid stress; Spider biofilm induced
orf19.2458	SIP5	232.02	219.75	222.85	173.28	-0.181503575	0.930538	Protein of unknown function; flow model, rat catheter and Spider biofilm induced
orf19.6160	orf19.6160	213.12	219.67	287.93	272.33	0.319031732	0.496552	Ortholog(s) have role in eisosome assembly and eisosome, membrane raft, mitochondrion, plasma membrane localization
orf19.1860	LSC2	227.86	219.23	329.42	337.63	0.50931028	0.059858	Putative succinate-CoA ligase beta subunit; regulated by Mig1, Tup1; induced in high iron; protein present in exponential and stationary growth phase yeast cells; Spider biofilm repressed
orf19.6109	TUP1	232.61	219.22	273.12	241.51	0.156074689	0.951606	Transcriptional corepressor; represses filamentous growth; regulates switching; role in germ tube induction, farnesol response; in repression pathways with Nrg1, Rfg1; farnesol upregulated in biofilm; rat catheter, Spider biofilm repressed
orf19.3542	LEM3	226.55	218.96	249.66	227.66	0.077046803	0.998825	Putative membrane protein; mutation increases resistance to miltefosine; early-stage flow model biofilm induced

orf19.3518	orf19.3518	214.64	218.53	310.76	317.42	0.466680795	0.115955	Ortholog(s) have dicarboxylic acid transmembrane transporter activity, role in mitochondrial transport and mitochondrial inner membrane localization
orf19.1336	PUP3	261.67	217.46	235.25	226.15	-0.057968929	0.998825	Putative beta 3 subunit of the 20S proteasome; macrophage/pseudohyphal-repressed
orf19.2726	orf19.2726	275.83	217.16	120.27	82.28	-1.101266825	1.05E-05	Putative plasma membrane protein; Plc1-regulated; Spider biofilm induced
orf19.2432	HAC1	207.17	216.62	199.14	208.55	-0.059999506	0.998825	bZIP transcription factor; role in unfolded protein response and control of morphology; transcript undergoes atypical splicing at C terminus under ER stress; induced during mating and by caspofungin; mRNA binds She3; Spider biofilm induced
orf19.327	HTA3	236.94	216	229.86	244.61	0.051935205	0.998825	Putative histone H2A; amphotericin B repressed; flucytosine induced; RNA abundance regulated by tyrosol and cell density; Spider biofilm repressed
orf19.2028	MXR1	230.56	215.86	185.83	174.28	-0.286266596	0.645919	Putative methionine sulfoxide reductase; Plc1-regulated; induced by human neutrophils, flucytosine; macrophage regulated (gene induced, protein decreased); possibly adherence-induced; Spider biofilm induced
orf19.6418	orf19.6418	206.7	215.66	176.59	175.02	-0.244234494	0.809395	Ortholog(s) have role in ribosomal large subunit biogenesis and cytoplasm, nucleus localization
orf19.2356	CRZ2	187.67	215.62	92.81	74.74	-1.074718925	3.29E-05	C2H2 transcription factor; crz1 not crz2 null mutation suppresses fluconazole resistance of homozygous cka2 null; Rim101-repressed at pH 8; required for yeast cell adherence to silicone substrate; Spider biofilm induced
orf19.7215.3	orf19.7215.3	209.87	215.54	258.93	246.09	0.206053141	0.866681	Ortholog(s) have chaperone binding, unfolded protein binding activity, role in protein refolding and mitochondrial matrix localization

orf19.2599	CRC1	219.1	215.02	217.4	218.4	-0.007887855	0.998825	Mitochondrial carnitine carrier protein
orf19.6807	orf19.6807	213.83	214.66	246.91	217.8	0.090115721	0.998825	Predicted ORF in retrotransposon Tca17 with similarity to parts of the Gag-Pol region of retrotransposons; clade-associated gene expression
orf19.2859	SRP40	201.86	214.57	157.84	151.82	-0.384962159	0.383894	Putative chaperone of small nucleolar ribonucleoprotein particles; macrophage/pseudohyphal-induced; rat catheter biofilm induced
orf19.4051	HTS1	213.84	214.25	218.94	190.47	-0.070927089	0.998825	Putative tRNA-His synthetase; downregulated upon phagocytosis by murine macrophage; stationary phase enriched protein; Spider biofilm repressed
orf19.5666	orf19.5666	211.43	213.6	271.3	272.96	0.306347949	0.546697	Ortholog(s) have transcription coactivator activity
orf19.225	orf19.225	207.43	213.07	194.96	221.79	-0.018765393	0.998825	Predicted 2-hydroxyacid dehydrogenase; Hap43-repressed gene
orf19.3931	SFC1	242.41	212.44	189.18	137.16	-0.427645824	0.280569	Putative succinate-fumarate transporter; involved in repression of growth on sorbose; alkaline induced; rat catheter biofilm induced; Spider biofilm induced
orf19.5906	ADE2	252.96	212.3	254.02	223.52	0.025105513	0.998825	Phosphoribosylaminoimadazole carboxylase; role in adenine biosynthesis; required for normal growth and virulence in immunosuppressed mouse infection; not induced in GCN response, in contrast to S. cerevisiae ADE2; stationary phase-enriched
orf19.5198	NOP4	236.84	212.19	171.22	178.08	-0.328247074	0.533718	Putative nucleolar protein; Hap43-induced; mutation confers hypersensitivity to 5-fluorocytosine (5-FC), 5-fluorouracil (5-FU), and tubercidin (7-deazaadenosine); represses in core stress response

orf19.2364	MIS11	214.41	211.61	299.8	316.32	0.465853919	0.122226	Predicted mitochondrial C1-tetrahydrofolate synthase precursor; putative protein of glycine catabolism; repressed by Efg1; fluconazole-induced; stationary phase enriched protein; rat catheter and Spider biofilm repressed
orf19.4674.1	CRD2	214.44	211.56	214.79	200.74	-0.042259198	0.998825	Metallothionein; for adaptation to growth in high copper; basal transcription is cadmium-repressed; Ssn6 regulated; complements copper sensitivity of an S. cerevisiae cup1 mutant; regulated by Sef1, Sfu1, and Hap43; Spider biofilm induced
orf19.7643	orf19.7643	230.54	210.86	256.64	235.54	0.129365548	0.998825	Ortholog(s) have 4-hydroxybenzoate octaprenyltransferase activity, antioxidant activity and role in cellular response to cadmium ion, detoxification of cadmium ion, ubiquinone biosynthetic process
orf19.3325	orf19.3325	185.17	210.3	200.5	202.17	0.012409904	0.998825	Putative glycogen synthesis initiator; regulated by Efg1 and Efh1; Hog1-repressed; colony morphology-related gene regulation by Ssn6; induced by prostaglandins; flow model biofilm induced
orf19.6886	orf19.6886	237.17	210.01	181.47	168.25	-0.324351251	0.541043	Ortholog(s) have rRNA binding activity, role in maturation of LSU-rRNA from tricistronic rRNA transcript (SSU-rRNA, 5.8S rRNA, LSU-rRNA), ribosomal large subunit export from nucleus and nucleolus localization
orf19.581	orf19.581	181.54	209.96	181.23	175.12	-0.134431615	0.998825	Putative RNA-binding protein; transcript is upregulated in an RHE model of oral candidiasis
orf19.6074	HBR1	199.96	209.86	173.67	189.38	-0.164104603	0.957711	Essential protein involved in regulation of MTL gene expression; hemoglobin-regulated inhibitor of white-opaque switching, may affect survival in host; activator of MTLalpha1 and MTLalpha2; transcript activated by hemoglobin
orf19.7265	orf19.7265	231.65	209.27	233.38	230.65	0.055474199	0.998825	Putative zinc-finger domain protein with a predicted role in pre-mRNA splicing; Hap43-repressed; Spider biofilm repressed
orf19.5228	RIB3	184.28	209.25	145.55	167.05	-0.293543865	0.692027	3,4-Dihydroxy-2-butanone 4-phosphate synthase; homodimeric enzyme of riboflavin biosynthesis; converts ribulose 5-phosphate to L-3,4-dihydroxy-2-butanone 4-phosphate; transcription regulated on yeast-hyphal switch, macrophage interaction

orf19.5447	HGT19	199.33	209.24	252.59	196.26	0.105748121	0.998825	Putative MFS glucose/myo-inositol transporter; 20 member family; 12 transmembrane segments, extended N terminus; expressed in rich medium; Hap43, phagocytosis, rat catheter, Spider and flow model biofilm induced
orf19.6623	orf19.6623	203.56	209.18	180.8	227.18	-0.023431924	0.998825	Ortholog(s) have RNA polymerase II core binding activity, role in regulation of transcription by chromatin organization, transcription elongation from RNA polymerase II promoter and transcription elongation factor complex localization
orf19.3867	RPL7	244.11	208.83	184.72	183.99	-0.269463445	0.711296	Ribosomal protein L7; repressed upon phagocytosis by murine macrophages; Hap43-induced; rat catheter and Spider biofilm induced
orf19.3606	orf19.3606	190.78	208.29	133.56	150.65	-0.42833048	0.29737	Ortholog of S. cereviae Sna4 vacuolar outer membrane protein that plays a role in sensitivity to NA+; induced by Mnl1 under weak acid stress
orf19.5850	NOC2	224.99	207.42	188.28	172.46	-0.242498829	0.800869	Putative nucleolar complex protein; Hap43-induced; transposon mutation affects filamentous growth; mutation confers hypersensitivity to 5-fluorouracil (5-FU), tubercidin (7-deazaadenosine); repressed in core stress response
orf19.978	BDF1	177.72	207.34	191.01	183.3	-0.050249482	0.998825	Bromodomain transcription factor; repressed upon adherence to polystyrene; reduced mRNA abundance detected in null mutant; macrophage/pseudohyphal-repressed; possibly essential, disruptants not obtained by UAU1 method
orf19.7600	FDH3	198.02	207.28	274.42	280.82	0.393000534	0.282737	Glutathione-dependent formaldehyde dehydrogenase; glycine catabolism; repressed by Efg1 in yeast, not hyphal growth conditions; induced by Mnl1 under weak acid stress; Spider biofilm repressed
orf19.482	RPT4	239.35	207.16	230.59	230.19	0.033339428	0.998825	26S proteasome regulatory subunit; regulated by Gcn2p and Gcn4p; protein level decreases in stationary phase cultures
orf19.5912	MAK21	206.5	206.49	177.42	184.32	-0.180683478	0.931333	Putative 66S pre-ribosomal particle subunit; mutation confers hypersensitivity to tubercidin (7-deazaadenosine)

orf19.1414	orf19.1414	188.49	206.39	167.77	149.36	-0.286078516	0.692076	Protein of unknown function; transcript is upregulated in an RHE model of oral candidiasis; Hap43-repressed
orf19.7310	orf19.7310	175.17	206.14	175.4	173.37	-0.126736368	0.998825	Protein with a role in directing meiotic recombination events to homologous chromatids; induced by ciclopirox olamine; positively regulated by Sfu1; Hog1, fluconazole-repressed; Hap43-induced; Spider biofilm induced
orf19.1350	orf19.1350	198.98	205.89	175.67	187.06	-0.148872998	0.998825	Protein with a thioredoxin domain; predicted role in cell redox homeostasis; rat catheter and Spider biofilm induced
orf19.7635	DRS1	217.24	205.79	176.9	168.68	-0.265426558	0.732635	Putative nucleolar DEAD-box protein; Hap43-induced; mutation confers hypersensitivity to 5-fluorouracil (5-FU), tubercidin (7-deazaadenosine); Tbf1-induced; repressed in core stress response
orf19.4831	MTS1	222.97	205.63	336.75	329.6	0.5625412	0.026248	Sphingolipid C9-methyltransferase; catalyzes methylation of the 9th carbon in the long chain base component of glucosylceramides; glucosylceramide biosynthesis is important for virulence; Spider biofilm repressed
orf19.6324	VID27	213.84	204.76	214.42	212.91	0.013568423	0.998825	Protein similar to S. cerevisiae Vid27p; transposon mutation affects filamentous growth; mutation confers hypersensitivity to toxic ergosterol analog; fungal-specific (no human or murine homolog)
orf19.4540	UBC8	195.68	204.75	129.99	150.85	-0.450943889	0.250467	Predicted ubiquitin-conjugating enzyme that negatively regulates gluconeogenesis by mediating the glucose-induced ubiquitination of fructose-1,6-bisphosphatase; induced by alpha pheromone in SpiderM medium
orf19.2573	FRS1	195.68	204.57	197.86	204	-0.008194494	0.998825	Phenylalanyl-tRNA synthetase; possible role in early cell wall biosynthesis; downregulated by phagocytosis by macrophages; possibly essential gene, disruptants not obtained by UAU1 method; protein present in exponential and stationary phase
orf19.2341	HNT1	211.46	204.08	268.33	243.35	0.255206262	0.727457	Protein with similarity to protein kinase C inhibitor-I; protein level decreases in stationary phase cultures

orf19.1376	orf19.1376	204	203.92	203.45	219.3	0.032808543	0.998825	Ortholog(s) have SNAP receptor activity, phosphatidic acid binding activity
orf19.551	orf19.551	183.6	203.91	92.39	102.95	-0.84786573	0.002129	Ortholog of C. dubliniensis CD36 : Cd36_29980, C. parapsilosis CDC317 : CPAR2_204170, Candida tenuis NRRL Y-1498 : CANTEDRAFT_110235 and Debaryomyces hansenii CBS767 : DEHA2F17072g
orf19.6318	orf19.6318	198.94	202.54	181.93	186.11	-0.122791249	0.998825	Ortholog(s) have endoplasmic reticulum, mitochondrion localization
orf19.1162	orf19.1162	178.61	202.46	193.51	223.09	0.102433112	0.998825	Protein of unknown function; transcript upregulated by benomyl treatment
orf19.5622	GLC3	210.08	202.44	449.83	454.28	1.017254046	1.93E-07	Putative 1,4-glucan branching enzyme; fluconazole-induced; colony morphology-related gene regulation by Ssn6; stationary phase enriched protein
orf19.5110	OPY2	195.43	202.4	140.44	135.1	-0.470116993	0.202185	Predicted transmembrane protein; role in cell wall biogenesis; required for Cek1 phosphorylation; Spider biofilm induced
orf19.2444	CHS7	214.23	202.2	218.51	187.89	-0.04018447	0.998825	Protein required for wild-type chitin synthase III activity; similar to (but not functional homolog of) S. cerevisiae Chs7p, which effects ER export of Chs3p; induced cyr1 mutant hyphae and ras1 yeast-form cells; Spider biofilm induced
orf19.3672	GAL10	134.35	202.17	68.36	93.8	-0.842341115	0.010366	UDP-glucose 4-epimerase; galactose utilization; mutant has cell wall defects and increased filamentation; GlcNAc-, fluconazole- and ketoconazole-induced; stationary phase enriched protein; rat catheter and flow model biofilm induced
orf19.3593	RPT6	225.78	202.09	220.99	224.75	0.043454178	0.998825	Putative ATPase of the 19S regulatory particle of the 26S proteasome; transcript regulated by Mig1; regulated by Gcn2 and Gcn4

orf19.2651	CAM1-1	192	201.76	239.94	234.34	0.223621316	0.844939	Putative translation elongation factor; downregulated upon phagocytosis by murine macrophages; Hap43-induced gene; Spider biofilm repressed
orf19.1093	FLO8	162.41	200.97	141.54	144.87	-0.302392715	0.69073	Transcription factor; required for hyphal formation and CO2 induced white-opaque switching; regulates hyphal gene expression; required for virulence in mouse systemic infection; binds Efg1p; binds Mss11p via LisH motif; has LUFS domain
orf19.767	ERG3	134.62	200.32	124.42	156.74	-0.220890937	0.919016	C-5 sterol desaturase; introduces C-5(6) double bond into episterol; some clinical isolates show increased azole resistance and defects in hyphal growth and virulence; Efg1p-repressed; fluconazole-induced
orf19.6327	HET1	208.52	200.3	282.37	292.63	0.428670488	0.19844	Putative sphingolipid transfer protein; involved in localization of glucosylceramide which is important for virulence; Spider biofilm repressed
orf19.5861	KRE9	219.33	200.14	202	189.82	-0.095348258	0.998825	Protein of beta-1,6-glucan biosynthesis; required for serum-induced hyphal growth; required for efficient utilization of galactose and for growth on glucose; similar to S. cerevisiae Kre9p and Knh1p; O-glycosylated by Pmt1p
orf19.2116	NAT2	218.89	199.82	183.12	183.33	-0.181222836	0.930538	Putative N-terminal acetyltransferase; Hap43p-repressed gene; mutation confers hypersensitivity to toxic ergosterol analog
orf19.7513	ALK2	191.41	199.52	46.25	45.04	-1.715827781	4.13E-12	N-Alkane inducible cytochrome P450
orf19.5975	TRY4	257.61	199.36	200.44	144.32	-0.365031265	0.470755	C2H2 transcription factor; fluconazole-repressed; induced in ssr1 mutant; required for yeast cell adherence to silicone substrate; Spider biofilm induced
orf19.2138	ILS1	185.29	198.78	183.28	192.05	-0.041318192	0.998825	Putative isoleucyl-tRNA synthetase, the target of drugs including the cyclic beta-amino acid icofungipen/PLD-118/BAY-10-8888 and mupirocin; protein present in exponential and stationary growth phase yeast cultures

orf19.549	orf19.549	223.85	198.74	224.18	230.54	0.084269422	0.998825	Ortholog(s) have structural constituent of ribosome activity and mitochondrial inner membrane, mitochondrial large ribosomal subunit localization
orf19.706	NMD3	229.55	198.64	171.7	168.05	-0.303196668	0.608044	Putative nonsense-mediated mRNA decay protein; repressed in core stress response; repressed by prostaglandins
orf19.7603	orf19.7603	201.17	198.6	201.88	211.74	0.033408084	0.998825	Protein with a predicted role in cytochrome c oxidase assembly; rat catheter biofilm induced
orf19.2483	RIM1	199.71	197.8	282.5	291.06	0.460412205	0.140994	Putative single-stranded DNA-binding protein; protein level decreases in stationary phase cultures; rat catheter biofilm repressed
orf19.198	ASN1	192.37	197.48	253.48	261.63	0.347795871	0.432569	Putative asparagine synthetase; soluble protein in hyphae; regulated by Rim101; decreased expression at pH 4 vs pH 8; protein detected during exponential and stationary phases of yeast-form growth
orf19.4530.1	orf19.4530.1	150.91	197.27	277.85	333.01	0.691252692	0.012136	Protein of unknown function; regulated by Nrg1, Tup1; Spider and flow model biofilm induced
orf19.5867	WSC1	156.48	196.75	128.37	155.11	-0.279817321	0.788835	Putative cell wall component; transcript upregulated in cyr1 mutant (yeast or hyphae); Spider and flow model biofilm induced
orf19.2598	VMA4	225.49	196.67	292.64	293.62	0.416623847	0.216469	H+ transporting ATPase E chain; transcript regulated by Mig1; caspofungin repressed; protein level decreases in stationary phase cultures; rat catheter biofilm repressed
orf19.5430	BUD21	210.07	196.59	165.63	173.39	-0.240111715	0.823496	Small-subunit processome component; repressed by prostaglandins

orf19.7221	SET3	182.55	195.65	132.99	145.88	-0.390456778	0.405236	NAD-dependent histone deacetylase; mutations affect filamentous growth; genetic evidence suggests Set3/Hos2 function as a complex to regulate white-opaque switching, morphogenesis, and virulence; flow model biofilm induced
orf19.1378	SUP35	194.27	195.39	208.22	203.43	0.058347791	0.998825	Translation factor eRF3; shows prion-like aggregation in some, not all, studies; partially complements S. cerevisiae sup35 mutant translation defect; species barrier with S. cerevisiae Sup35p prion; gene not regulated by yeast-hyphal switch
orf19.5547	orf19.5547	184.33	194.99	230.42	205.77	0.164283751	0.951606	Protein of unknown function; Hap43-repressed gene
orf19.86	orf19.86	208.88	194.66	146.66	155.45	-0.374174651	0.416682	Putative glutathione peroxidase; induced by peroxide, exposure to neutrophils and macrophage blood fractions; repressed during infection of macrophages; Spider biofilm induced; flow model biofilm repressed
orf19.2013	KAR2	202.69	194.55	266.79	262.28	0.354635568	0.39542	Similar to Hsp70 family chaperones; role in translocation of proteins into the ER; induced in high iron; protein present in exponential and stationary growth phase yeast cultures; flow model and Spider biofilm repressed
orf19.5299	ECM1	215.69	194.11	147.85	165.64	-0.342068314	0.525494	Putative pre-ribosomal factor; decreased mRNA abundance observed in cyr1 homozygous mutant hyphae; induced by heavy metal (cadmium) stress; Hog1p regulated
orf19.6062.3	orf19.6062.3	214.5	193.94	240.45	215.13	0.125023033	0.998825	Mitochondrial protein; component of the mitochondrial inner membrane organizing system ; role in maintenance of crista junctions and inner membrane architecture; Spider biofilm repressed
orf19.6285	GLC7	202.7	193.7	222.81	189.37	0.034361551	0.998825	Putative catalytic subunit of type 1 serine/threonine protein phosphatase; regulated by Shp1; induced in high iron; alternatively spliced intron in 5' UTR
orf19.7152	orf19.7152	199.09	193.37	394.9	379.69	0.875500411	2.75E-05	Protein similar to Aspergillus CYSK O-acetylserine sulfhydrylase, suggesting that C. albicans uses an O-acetyl-serine (OAS) pathway of sulfur assimilation; upregulated in biofilm; protein level decreases in stationary phase cultures

orf19.6838	orf19.6838	223.85	193.24	271.74	230.86	0.228719028	0.824505	Putative protein of unknown function, transcript upregulated in clinical isolates from HIV+ patients with oral candidiasis; Spider biofilm induced
orf19.3054	RPN3	225.26	192.82	209.73	209.5	-0.00582087	0.998825	Putative non-ATPase regulatory subunit of the 26S proteasome lid; amphotericin B repressed; oxidative stress-induced via Cap1p
orf19.5339	orf19.5339	181.22	192.32	153.74	170.67	-0.182211	0.938642	Ortholog of S. cerevisiae Spg5; required for proteasome assembly during quiescence; transcript detected on high-resolution tiling arrays; Spider biofilm induced
orf19.1149	MRF1	199.22	192.18	104.55	122.21	-0.677150127	0.021711	Putative mitochondrial respiratory protein; induced by farnesol, benomyl, nitric oxide, core stress response; oxidative stress-induced via Cap1; stationary-phase enriched protein; Spider biofilm induced
orf19.732	orf19.732	249.78	192.02	126.68	92.06	-0.868470486	0.001375	Possible dehydrogenase; flow model biofilm induced; rat catheter biofilm induced; Spider biofilm induced
orf19.5843	SRR1	204.17	191.8	183.1	178.77	-0.124031954	0.998825	Two-component system response regulator; involved in stress response; Plc1-regulated; upregulated in cyr1 null mutant; flow model biofilm induced; Spider biofilm induced
orf19.3038	TPS2	181.39	191.62	197.65	180.47	0.003566515	0.998825	Trehalose-6-phosphate (Tre6P) phosphatase; mutant heat sensitive, accumulates Tre6P, decreased mouse virulence; possible drug target; 2 conserved phosphohydrolase motifs; no mammalian homolog; Hap43-repressed; flow model biofilm induced
orf19.5633	orf19.5633	226.16	191.59	216.91	195.57	-0.025368842	0.998825	F-box domain-containing protein; flow model biofilm induced
orf19.6994	BAT22	203.98	191.38	231.96	201.64	0.107797276	0.998825	Putative branched chain amino acid aminotransferase; regulated by Gcn4p; induced by farnesol treatment, GlcNAc, amino acid starvation (3-aminotriazole treatment); present in exponential and stationary growth phase yeast cultures

orf19.5130	PDI1	200.79	191.34	290.41	292.03	0.498594858	0.084877	Putative protein disulfide-isomerase; antigenic in human infection; soluble protein in hyphae; induced by filamentous growth; protein present in exponential and stationary growth phase yeast cultures; flow model biofilm repressed
orf19.2472.1	orf19.2472.1	214.19	191.14	211.54	266.63	0.205589381	0.886087	Ortholog of C. dubliniensis CD36 : Cd36_61610, C. parapsilosis CDC317 : CPAR2_107530, Candida tenuis NRRL Y-1498 : CANTEDRAFT_115252 and Debaryomyces hansenii CBS767 : DEHA2D02200g
orf19.2308	orf19.2308	166.74	191.08	84.44	97.34	-0.830301929	0.004051	Putative 6-phosphofructo-2-kinase; catalyzes synthesis of fructose-2,6-bisphosphate; Hap43-repressed; flow model, rat catheter and Spider biofilm induced
orf19.6040	SNF7	200.13	191.04	166.7	179.54	-0.159562816	0.971608	ESCRT III complex protein; role in proteolytic activation of Rim101 and Rim8 processing/activation; separable roles in RIM101 pathway and in transport from MVB to vacuole; involved in echinocandin and azole sensitivity
orf19.3321	MRS7	204	190.76	184.11	165.06	-0.167857338	0.951606	Member of the LETM1-like protein family, mitochondrial membrane protein
orf19.5650	PRO3	203.92	190.26	207.51	169.28	-0.068473336	0.998825	Delta 1-pyrroline-5-carboxylate reductase; protein induced during the mating process; alkaline induced; stationary phase enriched protein; Spider biofilm repressed
orf19.5444	TIM44	198.68	190.25	191.27	194.68	-0.019902547	0.998825	Protein involved in transport across membranes; Spider biofilm repressed
orf19.5686	orf19.5686	190.46	190.07	177.8	198.81	-0.0194441	0.998825	Protein of unknown function; Spider biofilm induced
orf19.709	PUP2	221.54	189.68	236.06	224.8	0.134109374	0.998825	Alpha5 subunit of the 20S proteasome; macrophage/pseudohyphal-repressed; regulated by Gcn2p and Gcn4p; protein present in exponential and stationary growth phase yeast cultures

orf19.7561	DEF1	186.51	189.55	315.3	260.05	0.526214946	0.067926	RNA polymerase II regulator; role in filamentation, epithelial cell escape, dissemination in RHE model; induced by fluconazole, high cell density; Efg1/hyphal regulated; role in adhesion, hyphal growth on solid media; Spider biofilm induced
orf19.2193	PRS5	199.5	188.56	162.56	162.62	-0.229229651	0.853836	Putative 5-phospho-ribosyl-1(alpha)-pyrophosphate synthetase; Hap43-induced; repressed during core stress response
orf19.7534	MIS12	183.9	188.43	167.67	159.54	-0.169908613	0.951606	Mitochondrial C1-tetrahydrofolate synthase precursor
orf19.3089	orf19.3089	208.3	188.36	146.31	154.16	-0.356374459	0.476823	Predicted mitochondrial intermembrane space protein; predicted role in phospholipid metabolism; rat catheter and Spider biofilm induced
orf19.7222	PAM16	195.03	188.35	178.02	174.64	-0.113637464	0.998825	Putative maltase; regulated by Gcn4; repressed by amino acid starvation (3-AT); rat catheter biofilm induced
orf19.5334	orf19.5334	195.69	188.05	145.75	144.42	-0.361655763	0.465203	Ortholog od S. cerevisiae Tis11, a mRNA-binding protein; transcription regulated by yeast-hyphal switch; flow model biofilm induced; Spider biofilm repressed
orf19.6601	orf19.6601	156.7	187.47	210.69	229.28	0.295786315	0.664094	Protein of unknown function; rat catheter and flow model biofilm induced
orf19.6105	MVD	160.22	187.26	218.85	222.88	0.291296288	0.665347	Mevalonate diphosphate decarboxylase; functional homolog of S. cerevisiae Erg19; possible drug target; regulated by carbon source, yeast-hypha switch, growth phase, antifungals; gene has intron; rat catheter, Spider biofilm repressed
orf19.7484	ADE1	205.83	186.5	252.76	254.8	0.3192202	0.525494	Phosphoribosylaminoimadazole succinocarboxamide synthetase, enzyme of adenine biosynthesis; not induced in GCN response, unlike the S. cerevisiae ortholog; fungal-specific (no human or murine homolog); levels decrease in stationary phase

orf19.1800	orf19.1800	159.87	186.24	107.13	137.08	-0.431237069	0.366764	Protein of unknown function; Spider biofilm induced
orf19.1964	orf19.1964	167.8	186.2	169.33	188.89	0.00310895	0.998825	Protein of unknown function; repressed by fluphenazine treatment; induced by benomyl treatment and in an RHE model; regulated by Nrg1, Tup1
orf19.2233	PRE2	205.47	186.16	191.68	184.75	-0.056305742	0.998825	Putative proteasome beta-5 subunit; macrophage-induced protein
orf19.1124.2	orf19.1124.2	179.41	185.56	194.02	169.32	-0.020360913	0.998825	Ortholog(s) have diphthine synthase activity, role in peptidyl-diphthamide biosynthetic process from peptidyl-histidine and cytosol, nucleus localization
orf19.1344	orf19.1344	236.09	185.18	434.81	348.42	0.788874357	0.000526	Protein of unknown function; fluconazole-induced; Spider biofilm induced
orf19.5521	ISA1	180.16	185.03	167.96	162.73	-0.134386173	0.998825	Putative mitochondrial iron-sulfur protein; alkaline repressed; induced in high iron; regulated by Sef1, Sfu1, Hap43; Spider biofilm induced
orf19.7570	UGA3	156.71	184.95	75.78	93.05	-0.846976524	0.00428	Zn(II)2Cys6 transcription factor; required for utilization of gamma-aminobutyrate (GABA) as a nitrogen source; mutants display decreased CFU in mouse kidneys; Spider biofilm induced
orf19.7125	orf19.7125	187.49	184.94	242.29	234.02	0.303088965	0.580563	Protein of unknown function; Spider biofilm repressed
orf19.4886	orf19.4886	183.14	184.94	67.63	59.88	-1.270457061	6.39E-07	Putative adhesin-like protein; Hap43-repressed; rat catheter and Spider biofilm induced

orf19.5286	YCP4	162.77	184.58	196.37	207.18	0.171885706	0.951606	Putative flavodoxin; flow model, rat catheter and Spider biofilm repressed
orf19.1453	SPT5	185.78	184.37	196.15	180.46	0.008263981	0.998825	Protein similar to S. cerevisiae Spt5p transcription elongation factor; transposon mutation affects filamentous growth
orf19.882	HSP78	183.17	183.86	145.7	141.86	-0.313565147	0.612468	Heat-shock protein; regulated by macrophage response, Nrg1, Mig1, Gcn2, Gcn4, Mnl1p; heavy metal (cadmium) stress-induced; stationary phase enriched protein; rat catheter and Spider biofilm induced
orf19.4282	IFH1	179.59	183.61	162.16	173.07	-0.114305687	0.998825	Transcription factor; forms a heterodimer with Fhl11 that is tethered to promoters by Tbf1; positively regulates rRNA and ribosomal protein gene transcription; Spider biofilm induced
orf19.55	orf19.55	211.62	183.09	204.87	212.3	0.060381312	0.998825	Ortholog(s) have role in cellular zinc ion homeostasis and endoplasmic reticulum localization
orf19.2960	FRS2	186.05	182.74	192.53	193.31	0.046114171	0.998825	Putative tRNA-Phe synthetase; downregulated upon phagocytosis by murine macrophage; protein present in exponential and stationary growth phase yeast cultures; Spider biofilm repressed
orf19.24	RTA2	215.81	182.42	170.1	123.28	-0.391191245	0.410883	Flippase; sphingolipid long chain base release; mediates calcineurin-dependent resistance to azoles; stress-associate; Plc1, Ca2+, calcineurin-regulated; ketoconazole, caspofungin induced; Spider biofilm induced; flow biofilm repressed
orf19.5263	SER33	200.98	182.41	246.3	232.15	0.272637024	0.680517	Predicted enzyme of amino acid biosynthesis; Gcn4p-regulated; upregulated in biofilm; protein present in exponential and stationary growth phase yeast cultures; S. cerevisiae ortholog is Gcn4p regulated
orf19.3826	orf19.3826	181.8	182.41	162.36	165.18	-0.145021	0.998825	Predicted transmembrane protein with a role in cell wall polymer composition; Plc1-regulated; Spider biofilm induced

orf19.3015	ARX1	195.39	182.4	166.23	151.95	-0.224729725	0.8593	Putative ribosomal large subunit biogenesis protein; repressed in core stress response; repressed by prostaglandins
orf19.4739	MSS116	208.55	182.38	148.01	153.09	-0.335780441	0.543746	Putative DEAD-box protein; required for efficient splicing of mitochondrial Group I and II introns; Hap43-induced; rat catheter biofilm induced
orf19.6582	PRE10	210.8	182.21	188.66	195.44	-0.037699423	0.998825	Alpha7 (C8) subunit of the 20S proteasome; multiple phosphorylated residues; transcript induced upon filamentous growth; Spider biofilm repressed
orf19.4054	CTA24	171.22	182.21	254.87	262.49	0.47421281	0.144775	Putative transcription factor; positive regulator of gene expression; Efg1-repressed; member of a family of telomere-proximal genes; transcript upregulated in RHE model of oral candidiasis
orf19.339	NDE1	184.82	182.06	209.76	169.32	0.026709119	0.998825	Putative NADH dehydrogenase; may act alternatively to complex I in respiration; caspofungin repressed; rat catheter biofilm induced; Spider biofilm repressed
orf19.1214	orf19.1214	192.92	182.02	176.74	186.91	-0.046907274	0.998825	Ortholog(s) have metalloaminopeptidase activity, role in protein initiator methionine removal involved in protein maturation and cytosol, nucleus localization
orf19.2800	orf19.2800	78.78	181.74	105.77	178.21	0.040654296	0.998825	Ortholog of Candida albicans WO-1 : CAWG_00667
orf19.5732	NOG2	217.79	181.74	156.19	157.62	-0.311572928	0.606662	Putative nucleolar GTPase; repressed by prostaglandins; Hap43-induced, rat catheter and Spider biofilm induced
orf19.2694	TYS1	191.24	181.13	188.45	184.57	-0.007077793	0.998825	Putative tRNA-Tyr synthetase; downregulated upon phagocytosis by murine macrophages; stationary phase enriched protein; Spider biofilm repressed

orf19.2220	orf19.2220	199.59	181.08	40.73	27.58	-1.954541045	1.55E-14	Protein of unknown function; Hap43-repressed gene; by Rgt1; repressed in Spider biofilms by Bcr1, Tec1, Brg1, Rob1 and induced by Efg1, Ndt80; Spider biofilm induced
orf19.5407	SOF1	186.08	181.03	140.36	130.48	-0.393150878	0.389549	Putative protein with a predicted role in 40S ribosomal subunit biogenesis; rat catheter biofilm induced
orf19.2163	orf19.2163	181.18	180.94	218.87	210.77	0.206834435	0.878136	Ortholog(s) have cytosol localization
orf19.5063	COI1	193.59	180.73	199.78	191.63	0.045553658	0.998825	Secreted protein; ciclopirox olamine induced; regulated by Ssn6; induced by nitric oxide independent of Yhb1; Hap43-induced; Spider biofilm repressed
orf19.334	orf19.334	197.72	180.72	211.7	214.97	0.141941559	0.998825	Protein of unknown function; flow model biofilm induced; Spider biofilm induced; regulated by Sef1, Sfu1, and Hap43
orf19.5685	THS1	190.44	180.34	221.14	220.51	0.215813713	0.859429	Putative threonyl-tRNA synthetase; transcript regulated by Mig1 and Tup1; repressed upon phagocytosis by murine macrophages; stationary phase enriched protein; Spider biofilm repressed
orf19.1266	orf19.1266	176.85	180.09	175.82	156.51	-0.099917734	0.998825	Protein of unknown function; transcript detected on high-resolution tiling arrays
orf19.4347	orf19.4347	155.46	179.91	130.02	128.42	-0.333053585	0.600579	Putative serine/threonine protein kinase; Hog1p-induced
orf19.713	orf19.713	216.16	179.83	144.2	165.53	-0.311124235	0.621224	Ortholog(s) have role in apoptotic process and cytoplasm, nucleus localization

orf19.5078	OFR1	190.11	179.3	216.9	224.58	0.219603093	0.858075	Protein of unknown function; Hap43-repressed gene; rat catheter biofilm repressed
orf19.1553	ENT3	192.91	179.13	230.95	214.03	0.217007482	0.858769	Putative epsin; induced during the mating process; transcript is upregulated in an RHE model of oral candidiasis
orf19.424	TRP99	204.57	178.94	264.81	268.87	0.413571585	0.242657	Putative thioredoxin peroxidase/alkyl hydroperoxide reductase; induced in low iron; regulated by Gcn4; induced in response to amino acid starvation (3-AT treatment)
orf19.4870	DBP3	187.5	178.78	142.08	134.04	-0.362389287	0.476154	Putative ATP-dependent DEAD-box RNA helicase; Hap43-induced; repressed by prostaglandins; Spider biofilm induced
orf19.1448	APT1	190.71	178.66	197.02	207.16	0.101808434	0.998825	Adenine phosphoribosyltransferase; flucytosine induced; repressed by nitric oxide; protein level decreased in stationary phase yeast cultures
orf19.2014	BCY1	187.23	178.35	235.13	201.77	0.215959775	0.861217	Protein kinase A regulatory subunit; required for nuclear localization of Tpk1; physically interacts with Tpk1; essential; apoptosis-regulated; bcy1 heterozygous or bcy1 tpk2 mutant has filamentous growth defect
orf19.3553	RPF2	187.56	178.29	150.71	158.48	-0.221099473	0.87024	Putative pre-rRNA processing protein; Hap43p-induced gene; mutation confers hypersensitivity to 5-fluorocytosine (5-FC), 5-fluorouracil (5-FU), and tubercidin (7-deazaadenosine)
orf19.2934	orf19.2934	178.13	178.07	129.3	132.53	-0.389949562	0.410105	Similar to S. cerevisiae Bud20; predicted role in cellular bud site selection; rat catheter and Spider biofilm induced
orf19.518	orf19.518	169.98	177.94	169.61	180.37	-0.003683425	0.998825	Ortholog(s) have tRNA (cytosine-5-)-methyltransferase activity, role in tRNA methylation and nucleus localization

orf19.3799	orf19.3799	163.88	177.47	224.05	248.1	0.39949081	0.326634	Ortholog(s) have role in endoplasmic reticulum tubular network maintenance, nuclear pore complex assembly and Golgi apparatus, cortical endoplasmic reticulum, integral to endoplasmic reticulum membrane, mitochondrion localization
orf19.4492	orf19.4492	204.15	177.21	172.43	152.7	-0.209897027	0.881232	Ortholog(s) have role in nuclear division, rRNA processing and mitotic spindle pole body, nuclear periphery, nucleolus, preribosome, large subunit precursor localization
orf19.5635	PGA7	162.57	177.08	89.79	101.07	-0.706203922	0.021711	GPI-linked hyphal surface antigen; induced by ciclopirox olamine, ketoconazole, Rim101 at pH 8; Hap43, fluconazole; flow model biofilm induced; Spider biofilm induced; required for RPMI biofilm; Bcr1-induced in a/a biofilm
orf19.6434	PEX19	193.7	176.83	232.34	204.94	0.196563549	0.892679	Ortholog(s) have peroxisome membrane targeting sequence binding activity
orf19.3441	FRP6	177.48	176.69	124.23	109.55	-0.521819297	0.142557	Putative ammonia transport protein; regulated by Nrg1 and Tup1; regulated by Ssn6; induced by human neutrophils
orf19.6843	orf19.6843	157.36	176.55	172.47	187.73	0.081799261	0.998825	Ortholog(s) have role in CENP-A containing nucleosome assembly at centromere, chromatin silencing at centromere, mitotic sister chromatid segregation and nucleoplasm localization
orf19.5360	RPC11	182.41	176.29	126.32	131.09	-0.419981989	0.328035	Putative RNA polymerase III subunit C11; repressed in core caspofungin response; Spider biofilm induced
orf19.4396	orf19.4396	204.58	176.07	251.32	217.41	0.250888228	0.772112	Mitochondrial inner membrane protein; mammalian mitofilin domain; Spider biofilm repressed
orf19.6236	NOP6	202.92	175.62	139.5	148.58	-0.349739631	0.520013	Putative ortholog of S. cerevisiae Nop6; role in ribosomal small subunit biogenesis; Spider biofilm induced

orf19.2978	orf19.2978	192.53	174.77	203.24	189.25	0.069346847	0.998825	Protein of unknown function; Hap43-repressed gene
orf19.5058	SMI1	179.61	174.77	236.2	241.14	0.36556379	0.396023	Cell wall biosynthesis protein; Hap43, caspofungin-repressed; Cyr1-induced in hyphal cells; reduced biofilm cell wall glucan in mutant; possibly essential (UAU1 method); flow model biofilm induced
orf19.961.2	orf19.961.2	173.35	174.69	184.83	191.76	0.08998287	0.998825	Ortholog(s) have mitochondrion, nucleus localization
orf19.3053	orf19.3053	156.14	174.61	224.33	227.78	0.380153273	0.38818	Protein of unknown function; present in exponential and stationary phase yeast; identified in extracts from biofilm and planktonic cells; flow model biofilm induced gene; GlcNAc-induced protein
orf19.5450	ETR1	185.75	174.19	246.55	214.25	0.302244811	0.596579	Putative 2-enoyl thioester reductase; protein present in exponential and stationary growth phase yeast cultures; rat catheter biofilm repressed
orf19.6696	TIM9	239.22	173.77	225.05	271.84	0.228929656	0.858075	Predicted protein of the mitochondrial intermembrane space; rat catheter biofilm induced; Spider biofilm repressed
orf19.94	orf19.94	199.49	173.68	264.32	242.67	0.3820575	0.328035	Protein of unknown function; Spider biofilm induced
orf19.5968	RDI1	181.17	173.53	234.2	252.96	0.392398615	0.319876	Putative rho GDP dissociation inhibitor; transposon mutation affects filamentous growth; farnesol, filament-induced; regulated by Nrg1, Tup1; protein levels low in stationary phase yeast
orf19.1764	orf19.1764	172.41	173.02	127.34	131.07	-0.369245281	0.478105	Protein of unknown function; rat catheter and Spider biofilm induced

orf19.6365	PTP1	129.93	173.01	99.95	105.26	-0.477008031	0.291298	Phosphotyrosine-specific protein phosphatase; rat catheter biofilm induced
orf19.2003	HNM1	185.37	172.89	174.55	145.58	-0.148637101	0.998825	Putative choline/ethanolamine transporter; mutation confers hypersensitivity to toxic ergosterol analog; colony morphology-related gene regulation by Ssn6; clade-associated gene expression
orf19.1189	orf19.1189	143.98	172.74	82.8	89.67	-0.731307231	0.021239	Protein of unknown function; regulated by yeast-hypha switch; induced by Mnl1 in weak acid stress; 5' UTR intron; repressed by chlamydospore formation in C. albicans and C. dubliniensis; rat catheter, Spider and flow model biofilm induced
orf19.4513	orf19.4513	161.5	172.61	151.33	158.95	-0.103703282	0.998825	Protein of unknown function; mutants are viable; rat catheter and Spider biofilm induced
orf19.944	IFG3	154.03	172.18	168.72	167.2	0.024898871	0.998825	Putative D-amino acid oxidase; Spider biofilm induced
orf19.7335	PRE8	200.99	172.15	186.1	180.47	-0.033029231	0.998825	Putative alpha-2_sc subunit of proteasome; macrophage-induced protein; regulated by Gcn2p and Gcn4p; transcription is positively regulated by Tbf1p; stationary phase enriched protein
orf19.2601	HEM1	157.04	171.88	159.89	178.28	0.022712357	0.998825	Putative 5-aminolevulinate synthase; caspofungin repressed; induced by high iron, nitric oxide; regulated by Ssn6; Hap43-repressed; Spider biofilm induced
orf19.76	SPB1	187.62	171.86	139.43	132.99	-0.358520728	0.49115	Putative AdoMet-dependent methyltransferase; Hap43-induced; repressed by prostaglandins; possibly essential gene, disruptants not obtained by UAU1 method; Spider biofilm induced
orf19.4127	orf19.4127	171.54	171.76	162.95	148.15	-0.137974317	0.998825	Ortholog(s) have actin filament binding activity, role in actin cortical patch localization, actin filament bundle assembly, endocytosis and actin cortical patch localization

orf19.1153	GAD1	162.98	171.62	163.74	175.59	0.007716037	0.998825	Putative glutamate decarboxylase; alkaline, macrophage-downregulated gene; amphotericin B induced; induced by Mnl1 under weak acid stress; stationary phase enriched protein; rat catheter biofilm repressed
orf19.7166	orf19.7166	206.54	171.32	42	35.47	-1.836960776	3.65E-13	Predicted mitochondrial cardiolipin-specific phospholipase; upregulated in an azole-resistant strain that overexpresses MDR1; induced by Mnl1 under weak acid stress; rat catheter and Spider biofilm induced
orf19.745	VAC8	175.05	171.29	172.68	167.86	-0.029277781	0.998825	Protein involved in vacuolar inheritance; required for hyphal growth; contains armadillo repeats
orf19.5455	orf19.5455	205.69	171.25	272.55	235.25	0.369636019	0.382256	Ortholog(s) have mRNA binding, small GTPase regulator activity
orf19.7332	ELF1	183.54	171.2	141.5	130.66	-0.341523576	0.545514	Putative mRNA export protein; Walker A and B (ATP/GTP binding) motifs; required for wild-type morphology, growth; expressed in hyphal, pseudohyphal, and yeast form; Hap43-induced; Spider and flow model biofilm induced
orf19.933	orf19.933	184.11	170.62	165.63	181.99	-0.033559707	0.998825	Ortholog(s) have protein homodimerization activity, ubiquitin-protein ligase activity and role in UV-damage excision repair, free ubiquitin chain polymerization, postreplication repair, protein K63-linked ubiquitination
orf19.5005	OSM2	186.37	170.52	221.07	150.41	0.032662759	0.998825	Putative mitochondrial fumarate reductase; regulated by Ssn6p, Gcn2p, and Gcn4p; Hog1p-downregulated; stationary phase enriched protein; Hap43p-repressed gene
orf19.5112	TKL1	144.68	170	286.58	324.81	0.82762235	0.000732	Putative transketolase; localizes to surface of yeast cells, not hyphae; soluble protein in hyphae; transcript regulated by Nrg1, Mig1, and Tup1; antigenic in human or murine infection; possibly essential (by UAU1 method)
orf19.2693	GST2	171.02	169.51	129.3	150.15	-0.255956276	0.823496	Glutathione S transferase; induced by benomyl and in populations of cells exposed to fluconazole over multiple generations; regulated by Nrg1, Tup1; induced by nitric oxide; stationary phase enriched; Spider biofilm induced

orf19.2917	orf19.2917	212.28	169.5	153.97	132.66	-0.36121581	0.486965	Putative GTPase; heterozygous null mutant exhibits resistance to parnafungin in the C. albicans fitness test; Hap43p-induced gene
orf19.951	orf19.951	167.76	169.3	110.47	93.21	-0.629208178	0.051242	Protein of unknown function; transcript repressed upon yeast-hyphal switch; fluconazole-induced; Hap43-repressed; flow model biofilm induced
orf19.406	ERG1	188.55	169.25	163.12	171.51	-0.091716278	0.998825	Squalene epoxidase, epoxidation of squalene to 2,3(S)-oxidosqualene; ergosterol biosynthesis; allylamine antifungal drug target; NADH reducing cofactor but S. cerevisiae Erg1 uses NADPH; flow model biofilm induced; Spider biofilm repressed
orf19.2119	NDT80	169.02	169.2	128.48	106.48	-0.463548029	0.252792	Ortholog of Ndt80; meiosis-specific transcription factor; activator of CDR1 induction by antifungal drugs; required for wild-type drug resistance and for Spider biofilm formation; transcript induced by antifungal drug treatment
orf19.6563.1	orf19.6563.1	168.42	169.15	175.82	207.12	0.150896805	0.998825	Ortholog(s) have role in mitochondrial respiratory chain complex IV assembly, negative regulation of mitochondrial translation and integral to mitochondrial inner membrane localization
orf19.4882	orf19.4882	162.94	169.09	162.5	168.39	-0.014794081	0.998825	TFIIE small subunit; involved in RNA polymerase II transcription initiation; Spider biofilm induced
orf19.7448	LYS9	185.86	169.01	272.74	268.42	0.529352089	0.064426	Saccharopine dehydrogenase; lysine biosynthesis; soluble protein in hyphae; amphotericin B repressed; Gcn4-regulated; colony morphology-related gene regulation by Ssn6; protein present in exponential and stationary growth phase yeast
orf19.2842	GZF3	184.14	169	122.35	117.53	-0.485953337	0.198543	GATA-type transcription factor; oxidative stress-induced via Cap1; mutant has abnormal colony morphology and altered sensitivity to fluconazole, LiCl, and copper; Spider biofilm induced
orf19.223	orf19.223	194.88	168.89	155.07	145.89	-0.246832733	0.824321	Putative serine/threonine protein kinase; Hap43-repressed; induced by prostaglandins; possibly an essential gene, disruptants not obtained by UAU1 method; flow model biofilm induced; Spider biofilm induced

orf19.6406	orf19.6406	197.09	168.64	210.17	180.68	0.072535199	0.998825	Ortholog(s) have role in regulation of sporulation resulting in formation of a cellular spore and cytosol, mitochondrial ribosome, nucleus localization
orf19.4796	orf19.4796	160.5	168.52	174.36	201	0.149597874	0.998825	Putative eIF-4E-binding repressor of CAP-dependent translation; stationary phase enriched protein
orf19.1623	CAP1	176.71	168.46	157.17	132.08	-0.23218688	0.8593	AP-1 bZIP transcription factor; apoptotic, oxidative stress response/resistance, multidrug resistance; nuclear in oxidative stress; complements S. cerevisiae yap1 mutant; oralpharyngeal candidasis-, human neutrophil, Spider biofilm induced
orf19.3168	RPN8	193.39	168.44	172.41	164.75	-0.095794353	0.998825	Putative regulatory subunit of the 26S proteasome; mutation confers hypersensitivity to amphotericin B; regulated by Mig1, Gcn2 and Gcn4; Spider biofilm repressed
orf19.3220	orf19.3220	193.29	168.35	117.7	118.91	-0.527290204	0.132203	Putative rRNA processing protein; Spider biofilm induced
orf19.6584	PRT1	159.25	168.34	160.64	152.85	-0.062782571	0.998825	Putative translation initiation factor eIF3; mutation confers hypersensitivity to roridin A, verrucarin A; genes encoding ribosomal subunits, translation factors, tRNA synthetases are downregulated upon phagocytosis by murine macrophages
orf19.1866	VMA10	152.69	168.08	200.28	201.36	0.266695453	0.752654	Subunit G of the V1 peripheral membrane domain of the vacuolar H+-ATPase (V-ATPase); involved in vacuolar acidification; rat catheter biofilm repressed
orf19.1743	ACS1	178.7	168.05	278.35	219.71	0.444383831	0.213372	Acetyl-CoA synthetase; induced by human neutrophils; fluconazole-repressed; regulated by Nrg1/Mig1; colony morphology-related gene regulation by Ssn6; only in stationary phase cultures; rat catheter biofilm repressed, Spider biofilm induced
orf19.7062	RPA135	174.25	167.89	164.78	158.63	-0.078522805	0.998825	Putative RNA polymerase I subunit A135; repressed by prostaglandins

orf19.7308	TUB1	179.03	167.6	225.3	218.27	0.299384879	0.608044	Alpha-tubulin; gene has intron; complements cold-sensitivity of S. cerevisiae tub1 mutant; C. albicans has single alpha-tubulin gene, whereas S. cerevisiae has two (TUB1, TUB3); farnesol-upregulated in biofilm; sumoylation target
orf19.4009	CNB1	162.73	167.53	162.29	190.92	0.070810673	0.998825	Regulatory subunit of calcineurin B (Ca[2+]-calmodulin-regulated S/T protein phosphatase); required for wild-type resistance to fluconazole or to SDS; micafungin is fungicidal to null mutant
orf19.5031	SSK1	174.49	167.48	170.1	145.42	-0.111248993	0.998825	Response regulator of two-component system; role in oxidative stress response, cell wall biosynthesis, virulence, hyphal growth on solid media; expressed in hyphae and yeast; peroxisomal targeting sequence (PTS1); Spider biofilm induced
orf19.1564	orf19.1564	184.53	167.44	247.87	240.96	0.409010917	0.268118	Plasma membrane-localized protein of unknown function
orf19.7529	EPL1	148.32	167.21	137.49	138.73	-0.174505614	0.957711	Subunit of the NuA4 histone acetyltransferase complex
orf19.916	orf19.916	169.29	167.02	147.3	138.67	-0.211400662	0.892679	Putative protein involved in apoptosis; Spider biofilm induced
orf19.2393	orf19.2393	174.8	166.84	138	146.97	-0.235381411	0.858075	Putative transcription factor with zinc finger DNA-binding motif
orf19.6640	TPS1	165.71	166.56	244.86	204.64	0.366743535	0.416195	Trehalose-6-phosphate synthase; role in hyphal growth and virulence in mouse systemic infection; induced in presence of human neutrophils; macrophage/pseudohyphal-repressed after 16h; stationary phase enriched protein; Hap43-repressed
orf19.437	GRS1	166.02	166.52	191.03	180.1	0.123841966	0.998825	Putative tRNA-Gly synthetase; genes encoding ribosomal subunits, translation factors, tRNA synthetases are downregulated upon phagocytosis by murine macrophage; stationary phase enriched protein

orf19.1607	ALR1	157.97	166.43	117.91	117.79	-0.399172567	0.415549	Putative transporter of divalent cations; hyphal-induced expression; rat catheter biofilm induced
orf19.3366	CSH3	184.5	165.85	238.53	223.38	0.338909483	0.481084	Functional homolog of S. cerevisiae Shr3p, which is a chaperone specific for amino acid permeases; localized to ER; required for wild-type amino-acid responsive hyphal growth and for mouse systemic virulence; regulated by Gcn2p and Gcn4p
orf19.4534	orf19.4534	155.64	165.44	94.42	88.92	-0.689299123	0.027714	Putative UBX-domain (ubiquitin-regulatory domain) protein; macrophage-downregulated gene
orf19.1551	orf19.1551	147.62	165.43	183.64	194.39	0.223326496	0.87024	Predicted protein of unknown function; overlaps CPR3/orf19.1552
orf19.2703	orf19.2703	186.9	164.9	161.92	161.01	-0.11670791	0.998825	Specificity factor required for ubiquitination and sorting of specific cargo proteins at the multivesicular body; ortholog of S. cerevisiae Ear1; transcript regulated by iron; Ssr1-represssed; Spider biofilm induced
orf19.5682	orf19.5682	143.99	164.89	151.66	161.47	0.00384421	0.998825	Ortholog(s) have protein binding, bridging, protein transporter activity and role in chromosome condensation, protein import into nucleus, protein targeting to membrane, regulation of mitotic cell cycle
orf19.6991	PRE3	219.72	164.73	192.03	174.08	-0.071343792	0.998825	Putative beta-1 proteasome subunit; macrophage-induced protein; regulated by Gcn2p and Gcn4p; GlcNAc-induced protein
orf19.4956	RPN1	177.67	164.42	171.72	164.7	-0.028768631	0.998825	Putative 19S regulatory particle of the 26S proteasome; regulated by Gcn2p and Gcn4p
orf19.4023	MRP2	138.68	164.26	174.92	170.77	0.150539841	0.998825	Protein similar to S. cerevisiae Mrp2p, which is a component of the small subunit of the mitochondrial ribosome; transposon mutation affects filamentous growth

orf19.6435	orf19.6435	181.3	164.22	278.72	295.05	0.642200066	0.012136	Highly conserved subunit of mitochondrial pyruvate carrier; Hap43-repressed; Spider biofilm repressed
orf19.1361	TIM23	172.14	164.18	159.13	163.72	-0.059039992	0.998825	Protein involved in mitochondrial matrix protein import
orf19.6102	RCA1	154.85	163.92	153.32	142.58	-0.105147545	0.998825	Protein involved in regulation of carbonic anhydrases; controls CO2 sensing; bZIP domain-containing transcription factor of the ATF/CREB family; null mutant displays slow growth, abnormal colony morphology and invasive growth
orf19.5125	orf19.5125	172.28	163.89	151.1	140.13	-0.189848813	0.931333	Protein of unknown function; induced by ketoconazole; Spider, F-12/CO2 and flow model biofilm induced
orf19.680	TIM50	156	163.88	156.77	161.05	-0.019041766	0.998825	Predicted component of the Translocase of the Inner Mitochondrial membrane (TIM23 complex); involved in mitochondrial protein import; Spider biofilm repressed
orf19.489	DAP1	160.76	163.65	166.21	211.01	0.175634456	0.954573	Similar to mammalian membrane-associated progesterone receptors involved in DNA damage response; induced in core stress response; Hog1 regulated; clade-associated expression; Hap43-repressed
orf19.4591	CAT2	166.48	163.5	189.2	163.62	0.07549669	0.998825	Major carnitine acetyl transferase; intracellular acetyl-CoA transport; localized in peroxisomes and mitochondria; induced in macrophages; Hog1-repressed; stationary phase enriched; farnesol-upregulated in biofilm; Spider biofilm induced
orf19.3260	orf19.3260	172.97	163.4	157.9	146.49	-0.135712338	0.998825	Ortholog(s) have phosphopantothenoylcysteine decarboxylase activity, role in coenzyme A biosynthetic process, response to salt stress and phosphopantothenoylcysteine decarboxylase complex localization
orf19.5345	orf19.5345	178.11	163.27	204.16	179.57	0.138081293	0.998825	Putative ubiquitin-like polyubiquitin-binding protein; induced by nitric oxide independent of Yhb1; Spider biofilm repressed

orf19.5825.1	orf19.5825.1	178.24	163.21	183.45	218.18	0.195465201	0.918336	Ortholog(s) have role in ER to Golgi vesicle-mediated transport and ER to Golgi transport vesicle, endoplasmic reticulum membrane, integral to Golgi membrane localization
orf19.3205	MPRL36	154.4	163.17	143.66	161.19	-0.05773881	0.998825	Mitochondrial ribosomal protein of the large subunit; rat catheter biofilm induced
orf19.2525	LYS12	187.62	163.15	286.96	269.77	0.581275282	0.031297	Homoisocitrate dehydrogenase; catalyzes 4th step in the alpha-aminoadipate pathway of lysine biosynthesis; clade-associated gene expression; protein level decreases in stationary phase cultures; Spider biofilm repressed
orf19.2288	CCT5	169.95	162.67	165.56	175.6	0.023786062	0.998825	T-complex protein 1, epsilon subunit; protein present in exponential and stationary growth phase yeast cultures; Spider biofilm repressed
orf19.2045	orf19.2045	171.46	162.33	122.87	140.53	-0.293527206	0.718084	Ortholog(s) have diacylglycerol kinase activity, role in phosphatidic acid biosynthetic process and integral to endoplasmic reticulum membrane localization
orf19.1721	NCE103	305.7	162.22	181.32	108.29	-0.397726359	0.752654	Carbonic anhydrase; converts of CO2 to bicarbonate; essential for virulence in host niches with limited CO2, normal white-opaque switch; Mnl1-induced in weak acid stress; Hap43-induced gene; F-12/CO2, rat catheter, Spider biofilm induced
orf19.675.1	orf19.675.1	155.07	162.14	115.54	101.27	-0.473721258	0.249295	Putative adhesin-like protein
orf19.413.1	RPS27A	208.26	162.11	108.96	131.88	-0.526673877	0.157034	Ribosomal protein S27; rat catheter and Spider biofilm induced
orf19.5563	RNH1	164.88	162.09	189.21	218.23	0.265265797	0.767642	Ribonuclease H (RNAse H); hyphal-induced; flucytosine induced; similar to orf19.5564 (see Locus History); possibly essential (UAU1 method); rat catheter biofilm induced; flow model biofilm repressed

orf19.6589	SSF1	163.36	161.91	127.65	128.56	-0.29961002	0.692027	Protein involved in ribosome biogenesis; ortholog of S. cerevisiae Ssf1; Hap43-induced; rat catheter and Spider biofilm induced
orf19.661	KRR1	203.63	161.83	149.09	131.61	-0.336326158	0.568429	Putative nucleolar protein; repressed benomyl treatment or in an azole-resistant strain that overexpresses MDR1; F-12/CO2 early biofilm induced
orf19.3341	orf19.3341	170.79	161.81	170.93	172.13	0.02694558	0.998825	Putative tRNA-Arg synthetase; essential; genes encoding ribosomal subunits, translation factors, and tRNA synthetases are downregulated upon phagocytosis by murine macrophage; downregulated by growth in the mouse cecum
orf19.6257	GLT1	171.03	161.66	221.01	212.53	0.324553719	0.543746	Putative glutamate synthase; regulated by Sef1, Sfu1, and Hap43; rat catheter biofilm repressed
orf19.5461	orf19.5461	168.32	161.22	145.23	128.37	-0.242479987	0.849053	Ortholog of Candida albicans WO-1 : CAWG_02350
orf19.7282	PEX13	171.54	161.21	148.07	130.21	-0.235061965	0.858169	Protein required for peroxisomal protein import mediated by PTS1 and PTS2 targeting sequences; transcript induced in an RHE model of oral candidiasis; Hap43-repressed gene
orf19.3181.1	orf19.3181.1	138.11	160.61	106.82	134.87	-0.263056845	0.844939	Protein of unknown function; involved in secretion of proteins that lack classical secretory signal sequences; Spider biofilm induced
orf19.2974	YKT6	178.92	160.58	191.23	213.19	0.208761668	0.882407	Putative protein of the vacuolar SNARE complex; predicted role in vacuolar fusion; rat catheter biofilm repressed
orf19.2363	orf19.2363	187.51	160.36	158.56	188.69	-0.006112179	0.998825	Ortholog(s) have thiosulfate sulfurtransferase activity and mitochondrion localization

orf19.6828	orf19.6828	170.79	159.71	136.44	146.22	-0.207049854	0.902628	Ortholog(s) have role in rRNA processing and nucleolus, preribosome, large subunit precursor localization
orf19.1267.1	orf19.1267.1	133.8	159.71	174.25	202.41	0.291251133	0.723865	Ortholog(s) have enzyme activator activity, role in iron-sulfur cluster assembly and extrinsic to mitochondrial inner membrane, mitochondrial matrix localization
orf19.2920	orf19.2920	163.94	159.68	159.31	168.31	0.001672197	0.998825	Ortholog(s) have role in ribosome biogenesis and cytosol, ribosome localization
orf19.5365.1	orf19.5365.1	148.52	159.55	180.46	182.08	0.185540352	0.938404	Ortholog of C. parapsilosis CDC317 : CPAR2_407710, C. dubliniensis CD36 : Cd36_24540, Candida tenuis NRRL Y-1498 : CANTEDRAFT_116924 and Lodderomyces elongisporus NRLL YB-4239 : LELG_02895
orf19.1562	orf19.1562	131.6	159.51	64.01	76.49	-0.863187552	0.005668	Protein of unknown function; flow model biofilm induced; Spider biofilm induced; repressed by alpha pheromone in SpiderM medium
orf19.2795	LHP1	176.96	159.48	158.14	149.57	-0.118422816	0.998825	Ortholog(s) have tRNA binding activity, role in regulation of ascospore formation, tRNA 3'-trailer cleavage, endonucleolytic and nucleolus, nucleoplasm localization
orf19.5201	orf19.5201	159.36	158.98	169.8	175.79	0.094825269	0.998825	Ortholog(s) have structural constituent of ribosome activity and mitochondrial small ribosomal subunit localization
orf19.7076	GBP2	165.55	158.79	198.1	183.79	0.191779191	0.918389	Putative single-strand telomeric DNA-binding protein; protein level decreases in stationary phase cultures; Spider biofilm repressed
orf19.2422	ARC1	143.89	158.39	164.26	155.24	0.054815989	0.998825	Putative G4 nucleic acid binding protein; macrophage/pseudohyphal-repressed; protein enriched in stationary phase yeast-form cultures; Spider biofilm repressed

orf19.3478	NIP7	169.33	158.22	147.19	140.01	-0.17232027	0.957711	Putative nucleolar protein with role in ribosomal assembly; hyphal-induced; Hap43-induced; Spider biofilm induced
orf19.5602	BMT6	152.7	158.08	63.01	52.99	-1.162882572	2.19E-05	Beta-mannosyltransferase; beta-1,2-mannosylation of phospholipomannan; member of a 9-member family including Bmt1, Bmt2, Bmt3, and Bmt4 with roles in mannosylation of cell wall phosphopeptidomannan; flow model biofilm induced
orf19.4261	TIF5	165.8	158.04	146	134.41	-0.191877451	0.931333	Putative translation initiation factor; repressed upon phagocytosis by murine macrophage; Spider biofilm repressed
orf19.5000	CYB2	164.58	158.01	199.04	165.82	0.140089355	0.998825	Putative cytochrome b2 precursor; induced in high iron; alkaline repressed; colony morphology-related gene regulation by Ssn6; Hap43-repressed; pider biofilm induced
orf19.4045	EST1	155.18	157.95	146.92	127.55	-0.172264643	0.957711	Telomerase subunit; allosteric activator of catalytic activity, but not required for catalytic activity; has TPR domain
orf19.446.1	orf19.446.1	191.13	157.51	208.28	207.25	0.21023142	0.878136	Protein with a NADH-ubiquinone oxidoreductase B18 subunit domain; gene has intron
orf19.7349	CHS4	154.37	157.42	153.47	136.25	-0.103242133	0.998825	Activator of Chs3p chitin synthase; required for wild-type wall chitin content, but not for hyphal growth; mutant resistant to Calcofluor white; prenylation and 2 transmembrane segments predicted; functional homolog of S. cerevisiae Chs4p
orf19.6198.1	orf19.6198.1	205.52	157.37	165.55	227.19	0.095129432	0.998825	Ortholog of S. cerevisiae : YIL156W-B, C. glabrata CBS138 : CAGL0H06732g, C. dubliniensis CD36 : Cd36_06600, C. parapsilosis CDC317 : CPAR2_208850 and Candida tenuis NRRL Y-1498 : CANTEDRAFT_116509
orf19.2386	orf19.2386	186.17	157.35	130.1	126.96	-0.36462988	0.49417	Ortholog(s) have role in endonucleolytic cleavage in 5'-ETS of tricistronic rRNA transcript (SSU-rRNA, 5.8S rRNA and LSU-rRNA), more

orf19.3704.1	orf19.3704.1	159.84	156.75	145.81	172.92	-0.000495979	0.998825	Ortholog(s) have structural constituent of ribosome activity and mitochondrial small ribosomal subunit localization
orf19.3670	GAL1	96.55	156.74	67.27	106.67	-0.423856294	0.551067	Galactokinase; galactose, Mig1, Tup1, Hap43 regulated; fluconazole, ketoconazole-induced; stationary phase enriched protein; GlcNAc-induced protein; farnesol, hypoxia-repressed in biofilm; rat catheter and Spider biofilm induced
orf19.5684	orf19.5684	197.33	156.71	207.79	214.18	0.21411622	0.872276	Ortholog(s) have structural constituent of ribosome activity and mitochondrial large ribosomal subunit localization
orf19.3428	orf19.3428	157.16	156.35	121.38	94.25	-0.468049046	0.268834	Protein of unknown function; flow model biofilm induced
orf19.2841	PGM2	188.65	156.25	301.75	273.22	0.64319793	0.012136	Ortholog of S. cerevisiae Pgm2; induced in planktonic culture; Tye7p-regulated; flow model biofilm induced; rat catheter biofilm repressed
orf19.3161	orf19.3161	158.66	156.25	121.27	116.52	-0.354165093	0.543746	Ortholog(s) have ATPase activator activity, RNA binding activity
orf19.6445	ECI1	180.36	155.78	291.5	265.47	0.633181995	0.014285	Protein similar to S. cerevisiae Eci1p, which is involved in fatty acid oxidation; transposon mutation affects filamentous growth; expression is regulated upon white-opaque switching
orf19.5465	orf19.5465	125.04	155.7	114.04	118.63	-0.237389674	0.878538	Ortholog(s) have first spliceosomal transesterification activity, role in generation of catalytic spliceosome for first transesterification step and U2-type catalytic step 1 spliceosome, mitotic spindle pole body localization
orf19.5197	APE2	161.16	155.66	215.61	215.57	0.380107456	0.382965	Neutral arginine, alanine, leucine specific metallo-aminopeptidase; purified from cell wall/intracellular fractions; protein repressed during mating; Hog1, farnesol-induced; may be essential (UAU1 method); rat catheter biofilm repressed

orf19.4532	orf19.4532	161.66	155.6	154.36	141.6	-0.100270754	0.998825	Protein of unknown function; present in exponential and stationary growth phase yeast cultures
orf19.2407	DPS1-1	147.82	155.56	174.49	188.15	0.206555254	0.894293	Putative tRNA-Asp synthetase; genes encoding ribosomal subunits, translation factors, tRNA synthetases are downregulated upon phagocytosis by murine macrophage; protein enriched in stationary phase yeast cultures
orf19.6264.3	orf19.6264.3	159.26	155.51	151.47	142.18	-0.101048765	0.998825	Ortholog(s) have role in ER to Golgi vesicle-mediated transport and ER to Golgi transport vesicle, Golgi apparatus, endoplasmic reticulum localization
orf19.6423	orf19.6423	151.41	155.38	160.32	167.36	0.071675121	0.998825	Ortholog(s) have fructose-2,6-bisphosphate 2-phosphatase activity, role in glucose metabolic process and cytoplasm localization
orf19.5591	ADO1	175.85	155.23	214.23	204.96	0.287894238	0.664333	Adenosine kinase; heterozygous null mutant is resistant to cordycepin in C. albicans fitness test; ketoconazole-induced; protein level decrease in stationary phase cultures; sumoylation target; Spider biofilm repressed
orf19.5953	orf19.5953	139.22	155.22	133.13	137.38	-0.115897872	0.998825	C2H2 transcription factor; predicted regulator of ribosomal protein and biogenesis genes; possibly essential, disruptants not obtained by UAU1 method; rat catheter biofilm induced
orf19.5500	MAK16	198.55	155.04	131.81	127.77	-0.387349717	0.432569	Putative constituent of 66S pre-ribosomal particles; Hap43-induced; repressed by prostaglandins; Spider biofilm induced
orf19.847	YIM1	162.64	153.95	94.36	90.01	-0.667109087	0.035495	Protein similar to protease of mitochondrial inner membrane; increased transcription is observed upon benomyl treatment; macrophage-downregulated gene
orf19.350	PRE9	193.92	153.47	165.45	170.66	-0.046998925	0.998825	Alpha3 (C9) subunit of the 20S proteasome; transcript regulated by Mig1; flow model biofilm repressed

orf19.7292	ARP2	183.24	153.36	209.47	203.8	0.251322749	0.789669	Component of the Arp2/3 complex; required for virulence, hyphal growth, cell wall/cytoskeleton organization, not for endocytosis; mutation confers hypersensitivity to cytochalasin D; regulated by Gcn2 and Gcn4; Spider biofilm repressed
orf19.297	DTD2	147.07	153.3	153.88	133.58	-0.063422624	0.998825	Ortholog(s) have D-leucyl-tRNA(Leu) deacylase activity, D-tyrosyl-tRNA(Tyr) deacylase activity, role in D-leucine catabolic process, D-tyrosine catabolic process, tRNA metabolic process and cytosol, nucleus localization
orf19.6798	SSN6	135.72	153.12	139.87	129.18	-0.100975311	0.998825	Functional homolog of S. cerevisiae Cyc8/Ssn6; hyphal growth regulator; repressed during hyphal growth; Ssn6 and Tup1 regulate distinct sets of genes; overexpression or mutation causes avirulence in mouse IV infection; TPR motifs
orf19.399	orf19.399	146.45	153.05	132.78	126.16	-0.187987067	0.944705	Putative serine/threonine protein kinase; possibly an essential gene, disruptants not obtained by UAU1 method
orf19.4035	PGA4	135.8	152.96	164.28	153.77	0.104513008	0.998825	GPI-anchored cell surface protein; beta-1,3-glucanosyltransferase with similarity to the A. fumigatus GEL family; transcript induced in RHE model of oral candidiasis; fluconazol-induced
orf19.7160	orf19.7160	171.85	152.8	147.34	147.57	-0.129155798	0.998825	Ortholog(s) have unfolded protein binding activity
orf19.5459	orf19.5459	157.63	152.63	153.92	125.28	-0.146555503	0.998825	mRNA polyadenylation regulating protein; Hap43-repressed; transcript is upregulated in RHE model of oral candidiasis and in clinical isolates from HIV+ patients with oral candidiasis; Spider biofilm repressed
orf19.7261	GDI1	162.85	152.55	210.98	203.71	0.332957855	0.536918	Putative Rab GDP-dissociation inhibitor; GlcNAc-induced protein; Spider biofilm repressed
orf19.1661	DBP5	153.39	152.31	180.4	188.19	0.224206546	0.867579	Ortholog(s) have RNA helicase activity, RNA-dependent ATPase activity, inositol hexakisphosphate binding activity and role in mRNA export from nucleus, translational termination

orf19.7136	SPT6	152.29	152.1	162.75	151.02	0.028270615	0.998825	Putative transcription elongation factor; transposon mutation affects filamentous growth; transcript induced in an RHE model of oral candidiasis and in clinical isolates from oral candidiasis
orf19.5973	PHB2	161.67	151.86	185.46	166.54	0.131379921	0.998825	Prohibitin 2; plasma membrane localized
orf19.5378	SCL1	177.44	151.71	180.8	191.72	0.149207099	0.998825	Proteasome subunit YC7alpha; protein present in exponential and stationary growth phase yeast cultures; flow model and Spider biofilm repressed
orf19.4503	orf19.4503	155.47	151.45	201.93	211.71	0.369699892	0.424268	Similar to HMG-box variant of S. pombe; Spider biofilm repressed
orf19.2067	orf19.2067	143.19	151.45	120.34	130.44	-0.207771341	0.918842	Protein with a predicted role in mitochondrial iron metabolism; Hap43-repressed; expression upregulated during growth in the mouse cecum; Spider biofilm induced
orf19.357	orf19.357	162.47	150.93	181.09	149.18	0.053224463	0.998825	Ortholog(s) have role in inner mitochondrial membrane organization, mitochondrion inheritance, mitochondrion morphogenesis, negative regulation of proteolysis, protein folding, replicative cell aging
orf19.3004	orf19.3004	150.52	150.92	112.39	119	-0.336003671	0.606463	Ortholog(s) have fungal-type vacuole localization
orf19.4862.2	PET100	166.02	150.82	153.61	167.38	0.007152969	0.998825	Chaperone that facilitates the assembly of cytochrome c oxidase; plasma membrane protein
orf19.2439	orf19.2439	168.13	150.77	164.05	146.71	-0.04233439	0.998825	Fatty acid biosynthetic protein; shows colony morphology-related gene regulation by Ssn6p; protein newly produced during adaptation to the serum

orf19.5789	ADE8	164.86	150.73	186.19	204.99	0.258211147	0.789669	Putative phosphoribosylglycinamide formyl-transferase, enzyme of amino acid biosynthesis pathway; upregulated in biofilm; S. cerevisiae ortholog is Gcn4p regulated; protein enriched in stationary phase yeast-form cultures
orf19.791	RIM11	172.47	150.71	202.34	163.4	0.140540531	0.998825	Ortholog of S. cerevisiae Rim11; a protein kinase involved in meiosis and sporulation in S. cerevisiae; mutant is viable
orf19.7116	orf19.7116	167.74	150.26	158.6	140.76	-0.083017371	0.998825	Protein of unknown function; transcript induced in RHE model of oral candidiasis; Spider biofilm repressed
orf19.2151	NAG6	145.94	150.09	187.6	167.81	0.217013566	0.878538	Protein required for wild-type mouse virulence and wild-type cycloheximide resistance; putative GTP-binding motif; similar to S. cerevisiae Yor165Wp; in gene cluster that encodes enzymes of GlcNAc catabolism; no human or murine homolog
orf19.1559	HOM2	148.42	149.91	146.83	157.34	0.014462728	0.998825	Aspartate-semialdehyde dehydrogenase; forms a homodimer; conserved in bacteria, archaea, and fungi but not in mammals; ketoconazole-repressed; protein present in exponential and stationary growth phase yeast cultures; GlcNAc-induced protein
orf19.1949	VPS1	153.1	149.85	217.12	219.23	0.447877771	0.219359	Dynamin-family GTPase-related protein; induced upon adherence to polystyrene; regulated by Gcn2p and Gcn4p
orf19.6507	orf19.6507	162.79	149.84	194.18	187.01	0.236409371	0.842252	Putative curved DNA-binding protein orthologous to S. pombe Cdb4; stationary phase enriched protein; rat catheter and Spider biofilm repressed
orf19.4197	YHM2	183.48	149.78	194.45	142.69	0.002743488	0.998825	Predicted carrier protein; exports citrate from and imports oxoglutarate into the mitochondrion; alkaline induced; Spider biofilm repressed
orf19.3586	orf19.3586	140	149.65	145.31	137.66	-0.042377934	0.998825	Ortholog(s) have role in fungal-type cell wall organization, vesicle-mediated transport and integral to endoplasmic reticulum membrane localization

orf19.7229	IML2	130.2	149.64	119.96	125.13	-0.173454218	0.976613	Protein of unknown function; early-stage flow model biofilm induced; Hap43-repressed; Spider biofilm repressed
orf19.5026	orf19.5026	140.69	149.63	102.48	96	-0.475293394	0.266191	C2H2 transcription factor; Spider biofilm induced
orf19.4032	RPN5	166.89	149.62	143.59	149.74	-0.10193361	0.998825	Putative COP9 signalosome component; macrophage/pseudohyphal-repressed
orf19.7552	orf19.7552	146.89	149.58	119.4	112.96	-0.312355988	0.675986	Putative U3-containing small subunit processome complex protein; Hap43-induced gene; repressed in core stress response; Spider biofilm induced
orf19.1944	GPR1	135.53	149.51	195.68	203.92	0.40613003	0.34692	Plasma membrane G-protein-coupled receptor of the cAMP-PKA pathway; required for WT hyphal growth; reports differ on role in cAMP-mediated glucose signaling; Gpr1 C terminus binds Gpa2; regulates HWP1 and ECE1; flow model biofilm induced
orf19.5260	RPN2	171.56	149.29	175.34	155.74	0.027853729	0.998825	Putative 26S proteasome subunit; transcript regulated by Mig1; caspofungin repressed; regulated by Gcn2 and Gcn4; gene used for strain identification by multilocus sequence typing
orf19.2965.1	orf19.2965.1	143.35	149.12	148.77	207.95	0.236996888	0.871875	Protein of unknown function; similar to human SERF2; gene has an alternatively spliced intron
orf19.2275	orf19.2275	155.78	149.03	173.9	189.38	0.207831307	0.892679	Putative mitochondrial ribosomal protein; predicted role in aerobic respiration; Spider biofilm repressed
orf19.1785	orf19.1785	129.09	148.47	144.48	137.12	0.004200255	0.998825	Protein with a PI31 proteasome regulator domain; Hap43-repressed; flow model biofilm induced

orf19.7650	LTV1	161.44	148.35	118.44	127.64	-0.287170813	0.744983	Putative GSE complex component; repressed by prostaglandins
orf19.2191.1	orf19.2191.1	159.88	148.23	234.04	282.77	0.643680765	0.021264	Ortholog of C. dubliniensis CD36 : Cd36_11730, C. parapsilosis CDC317 : CPAR2_104195, Candida tenuis NRRL Y-1498 : CANTEDRAFT_109454 and Debaryomyces hansenii CBS767 : DEHA2C10076g
orf19.448	CUE5	148.95	148.16	210.3	190.11	0.361062432	0.459506	Predicted ubiquitin-binding protein; rat catheter biofilm repressed
orf19.2977	MIA40	171.49	148.05	146.71	145.88	-0.114067274	0.998825	Predicted component of the mitochondrial intermembrane space import machinery; Hap43p-repressed gene
orf19.1907	EMC9	144.72	147.59	144.5	139.21	-0.050015314	0.998825	Ortholog of S. cerevisiae Nnf2; possible role in chromosome segregation; mutants are viable, induced during the mating process
orf19.1250	orf19.1250	165.75	147.53	132.13	139.3	-0.189707058	0.938642	Ortholog(s) have role in maturation of SSU-rRNA from tricistronic rRNA transcript (SSU-rRNA, 5.8S rRNA, LSU-rRNA) and cytoplasm, nucleolus localization
orf19.4318	MIG1	129.88	147.17	148.45	168.8	0.154086784	0.998825	C2H2 transcription factor; repressor; regulates genes for carbon source utilization; Tup1-dependent and independent functions; hyphal, Hap43 and caspofungin repressed; Spider and flow model biofilm induced
orf19.1989	DCW1	159.13	146.98	169.53	165.06	0.102598443	0.998825	Protein with predicted GPI modification; dfg5 dcw1 double mutant is inviable; not required for wild-type hyphal growth; upregulated in cyr1 mutant (yeast or hyphal form); Hap43-induced
orf19.5307	JEN2	169.49	146.86	115.7	68.95	-0.641831247	0.075133	Dicarboxylic acid transporter; regulated by glucose repression; induced by Rgt1; disruptants not obtained by UAU1 method; rat catheter and Spider biofilm induced

orf19.5305	RHD3	140.36	146.58	26.3	29.73	-1.787138231	6.13E-11	GPI-anchored yeast-associated cell wall protein; induced in high iron; clade-associated gene expression; not essential for cell wall integrity; fluconazole-repressed; flow model and Spider biofilm repressed
orf19.2360	URA2	145.19	146.58	174.9	166.1	0.181009418	0.944705	Putative bifunctional carbamoylphosphate synthetase-aspartate transcarbamylase; flucytosine induced; macrophage/pseudohyphal-induced; 5'-UTR intron; flow model biofilm repressed
orf19.1402	CCT2	157.8	146.57	135.9	136.18	-0.15073902	0.998825	Chaperonin of the cytosolic TCP1 ring complex; protein present in exponential and stationary-phase yeast cells, but higher amounts in stationary phase; GlcNAc-induced protein
orf19.6032	SPE1	148.22	146.47	132	140.95	-0.100567143	0.998825	Ornithine decarboxylase; enzyme of polyamine biosynthesis; mutant is viable; functionally complements polyamine auxotrophy of S. cerevisiae spe1 mutant; downregulated upon adherence to polystyrene
orf19.6604	orf19.6604	158.87	146.31	183.84	215.96	0.330288357	0.568852	Ortholog of S. cerevisiae Pba1 that is involved in 20S proteasome assembly; upregulated in a cyr1 null mutant; contains a 5' UTR intron
orf19.1642	orf19.1642	182.74	146.01	126.46	125.46	-0.339118434	0.580563	Ortholog of S. cerevisiae Loc1, a nuclear protein involved in asymmetric localization of ASH1 mRNA in S. cerevisiae; Hap43-induced gene; Spider biofilm induced
orf19.7598	orf19.7598	159.01	145.93	127.66	129.15	-0.219557977	0.887125	Ortholog(s) have histone acetyltransferase activity, role in histone acetylation and Ada2/Gcn5/Ada3 transcription activator complex localization
orf19.6724	FUM12	183.14	145.8	249.26	196.48	0.367311604	0.44377	Putative fumarate hydratase; enzyme of citric acid cycle; fluconazole, Efg1 repressed; induced in high iron; protein present in exponential and stationary growth phase
orf19.3475	orf19.3475	129.51	145.74	67.35	54.54	-0.955537852	0.001518	Described as a Gag-related protein; hyphal induced; downregulation correlates with clinical development of fluconazole resistance; repressed by nitric oxide, 17-beta-estradiol, ethynyl estradiol

orf19.6358	orf19.6358	147.66	145.68	139.1	124.17	-0.148596565	0.998825	Ortholog(s) have protein homodimerization activity, ubiquitin-protein ligase activity and role in free ubiquitin chain polymerization, postreplication repair, protein K63-linked ubiquitination
orf19.4640	PWP1	159.66	145.61	126.3	124.23	-0.257409583	0.824505	Putative rRNA processing protein; Hap43-induced; repressed in core stress response
orf19.5420	orf19.5420	170.09	145.59	158.97	141.8	-0.070349393	0.998825	Ortholog(s) have role in fatty acid metabolic process
orf19.2737	orf19.2737	141.6	145.52	234.4	226.26	0.578109251	0.049927	Carbohydrate kinase domain-containing protein; Spider biofilm induced
orf19.6231	MRPL19	138.65	145.48	145.14	140.04	-0.00749672	0.998825	Putative ribosomal protein; induced upon adherence to polystyrene; Spider biofilm repressed
orf19.5661	PTC7	152.84	145.46	154.93	148.97	0.014082824	0.998825	Protein phosphatase, type 2C; has S/T phosphatase activity, Mn2+/Mg2+ dependent; predicted membrane-spanning segment and mitochondrion-targeting signal
orf19.3175	orf19.3175	150.91	144.96	161.4	161.61	0.098078061	0.998825	Putative ortholog of human electron transfer flavoprotein dehydrogenase (ETF-dH); alkaline repressed; repressed by nitric oxide; virulence-group-correlated expression; Spider biofilm induced
orf19.3439	orf19.3439	176.65	144.92	134.9	88.84	-0.446481561	0.34692	Protein of unknown function; Cyr1-repressed; rat catheter and Spider biofilm induced
orf19.2682	orf19.2682	135.21	144.79	113.32	123.79	-0.21191955	0.918336	Ortholog(s) have TBP-class protein binding, TBP-class protein binding RNA polymerase II transcription factor activity involved in preinitiation complex assembly activity

orf19.6254	ANT1	156.77	144.37	132.28	136.37	-0.151782098	0.998825	Peroxisomal adenine nucleotide transporter; role in beta-oxidation of medium-chain fatty acid and peroxisome proliferation; rat catheter biofilm induced
orf19.1742	HEM3	177.38	144.18	123.45	130	-0.298288507	0.711296	Hydroxymethylbilane synthase; converts 4-porphobilinogen to hydroxymethylbilane in heme biosynthesis; induced in high iron, CO2; alkaline repressed; regulated by Sef1, Sfu1, and Hap43; rat catheter and Spider biofilm induced
orf19.1387	orf19.1387	156.54	143.42	100.38	95.63	-0.523660045	0.177159	Ortholog(s) have 4 iron, 4 sulfur cluster binding activity, role in iron-sulfur cluster assembly, tRNA wobble uridine modification and cytoplasm localization
orf19.6271	orf19.6271	132.82	143.35	119.93	143.63	-0.062972101	0.998825	Ortholog(s) have role in mRNA splicing, via spliceosome and nucleus, transcriptionally active chromatin localization
orf19.446.2	orf19.446.2	129.47	143.19	163.92	179.4	0.274238414	0.779283	Protein with a NADH-ubiquinone oxidoreductase B18 subunit domain; gene has intron
orf19.3547	orf19.3547	165.55	143.04	122.9	111.79	-0.343924553	0.576749	Ortholog(s) have mRNA 3'-UTR binding, translation repressor activity, nucleic acid binding activity, role in negative regulation of translation, ribosomal large subunit biogenesis and large ribosomal subunit, nucleolus localization
orf19.699	orf19.699	131.3	142.95	86.42	107.57	-0.41679707	0.44528	Protein of unknown function; flow model, rat catheter, Spider biofilm induced
orf19.1168	ZCF3	122.52	142.89	80.8	64.02	-0.718637242	0.035341	Zn(II)2Cys6 domain transcription factor; required for filamentous growth, resistance to rapamycin and flucytosine; possibly an essential gene, disruptants not obtained by UAU1 method; Hap43-repressed; Spider and flow model biofilm induced
orf19.1560	POB3	143.28	142.88	153.79	144.78	0.043225546	0.998825	Protein involved in chromatin assembly and disassembly; ortholog of S. cerevisiae Pob3; transposon mutation affects filamentous growth; rat catheter biofilm repressed

orf19.4952.1	orf19.4952.1	158	142.79	181.75	212.29	0.327712348	0.578826	Ortholog(s) have FK506 binding, Hsp70 protein binding, chaperone binding, peptidyl-prolyl cis-trans isomerase activity, role in protein complex assembly and cytoplasm, nucleus localization
orf19.59	REI1	163.62	142.61	129.86	117.72	-0.271297254	0.789669	Putative cytoplasmic pre-60S factor; Hap43-induced; repressed by prostaglandins
orf19.798	TAF14	149.58	142.55	154.93	164.45	0.095776175	0.998825	Putative DNA-binding transcription factor; TFIIF subunit; macrophage-induced protein; possibly required for yeast cell adherence to silicone substrate; mutant has pleiotrophic effects
orf19.7675	orf19.7675	132.33	142.18	142.26	133.97	-0.00360041	0.998825	Mitochondrial ribosomal protein of the large subunit; Spider biofilm repressed
orf19.6237	RAC1	177.23	141.89	168.15	156.26	0.010640425	0.998825	G-protein of RAC subfamily; required for embedded filamentous growth, not for serum-induced hyphal growth; dynamic localization at plasma membrane and nucleus; similar to, but not interchangeable with, Cdc42p; lacks S. cerevisiae homolog
orf19.7097	orf19.7097	150.13	141.81	143.54	147.25	-0.013969552	0.998825	Putative cytoplasmic RNA-binding protein; heterozygous null mutant exhibits hypersensitivity to parnafungin and cordycepin in C. albicans fitness test; Spider biofilm repressed
orf19.2006.1	COX17	122.41	141.76	91.87	112.36	-0.314404678	0.729894	Putative copper metallochaperone; Hap43p-repressed gene; rat catheter biofilm induced; Spider biofilm induced
orf19.7050	NOP15	167.36	141.27	121.35	111.61	-0.349475788	0.568429	Nucleolar ribosome biogenesis factor; hyphal-induced expression; Hap43-induced; rat catheter biofilm induced
orf19.3292	orf19.3292	166.21	141.2	165.76	155.91	0.049859336	0.998825	Ortholog(s) have peptide-methionine (R)-S-oxide reductase activity, role in cellular response to oxidative stress and cytosol, mitochondrion, nucleus localization

orf19.6183	TIM8	115.69	140.83	124.02	165.54	0.134184367	0.998825	Predicted mitochondrial intermembrane space protein with role in protein import into mitochondrial inner membrane and cytosol
orf19.3831	orf19.3831	187.76	140.59	111.07	114.21	-0.466576279	0.268834	Ortholog(s) have telomerase inhibitor activity, role in box C/D snoRNA 3'-end processing, negative regulation of telomere maintenance via telomerase and nucleolus, nucleoplasm localization
orf19.7263	orf19.7263	147.02	140.48	141.53	125.91	-0.095534607	0.998825	Putative X-Pro aminopeptidase; Spider biofilm repressed
orf19.3462	SAR1	130.68	140.45	201.91	200.46	0.4794969	0.189885	Functional homolog of S. cerevisiae Sar1; which is required for ER-to-Golgi protein transport; binds GTP; similar to small GTPase superfamily proteins; gene has intron; Hap43-induced; rat catheter biofilm repressed
orf19.5279	orf19.5279	140.93	140.41	143.83	164.37	0.103638939	0.998825	Ortholog(s) have structural constituent of ribosome activity, role in aerobic respiration and mitochondrial large ribosomal subunit localization
orf19.4611	orf19.4611	149.08	140.32	154.86	167.33	0.124689649	0.998825	Ortholog(s) have ribose phosphate diphosphokinase activity, role in 5-phosphoribose 1-diphosphate biosynthetic process, cytokinesis, fungal-type cell wall organization and cytosol localization
orf19.1613	ILV2	157.09	140.27	168.09	182.4	0.195779897	0.922525	Putative acetolactate synthase; regulated by Gcn4p; induced by amino acid starvation (3-AT treatment); stationary phase enriched protein
orf19.215	orf19.215	126.19	140.26	107.54	84.64	-0.394955382	0.500172	Component of a complex containing the Tor2p kinase; possible a role in regulation of cell growth; Spider biofilm induced
orf19.5698	orf19.5698	151.74	139.94	137.74	135.1	-0.092550686	0.998825	Putative mitochondrial ribosomal protein of the large subunit; transcript is upregulated in clinical isolates from HIV+ patients with oral candidiasis; Spider biofilm repressed

orf19.753	MNN15	167.86	139.53	213.38	172.23	0.266751575	0.775225	Putative alpha-1,3-mannosyltransferase; predicted role in protein O-linked glycosylation; Spider biofilm induced
orf19.1279	CDS1	162.25	139.33	163.32	137.7	-0.010790692	0.998825	Protein similar to S. cerevisiae Cds1p; transposon mutation affects filamentous growth
orf19.6853	orf19.6853	140.25	139.3	166.12	171.46	0.223506415	0.875495	Protein of unknown function; Spider biofilm repressed
orf19.1203.1	orf19.1203.1	175.25	139.17	275.24	244.08	0.626622551	0.021718	Predicted dolichol-phosphate mannosyltransferase subunit; flow model and Spider biofilm repressed
orf19.6135.1	SMX4	136.89	138.98	147.19	142.14	0.045104044	0.998825	Component of heteroheptameric complexes (Lsm1p, Lsm8p) involved in RNA processing and decay; flucytosine induced
orf19.1097	ALS2	98.25	138.97	31.08	34.91	-1.361233731	8.36E-06	ALS family protein; role in adhesion, biofilm formation, germ tube induction; expressed at infection of human buccal epithelial cells; putative GPI-anchor; induced by ketoconazole, low iron and at cell wall regeneration; regulated by Sfu1p
orf19.200	orf19.200	154.83	138.8	187.96	196.16	0.324802319	0.577788	Putative nuclear RNA-binding protein; Spider biofilm repressed
orf19.3915	orf19.3915	131.37	138.77	163.46	175.51	0.269767859	0.788835	Putative metallodipeptidase; protein present in exponential and stationary growth phase yeast; Hog1-induced; Hap43-repressed; sumoylation target; Spider biofilm repressed
orf19.4960	orf19.4960	145.77	138.26	117.97	117.19	-0.24028677	0.863837	Ortholog(s) have spermine synthase activity, role in pantothenate biosynthetic process, spermine biosynthetic process and cytoplasm localization

orf19.2560	CDC60	134.26	138.12	143.19	137.51	0.028256238	0.998825	Cytosolic leucyl tRNA synthetase; conserved amino acid and ATP binding class I signature, tRNA binding, proofreading motifs; likely essential for growth; interacts with benzoxaborole antifungals; present in exponential and stationary phase
orf19.3914	orf19.3914	155.77	137.93	131.41	141.43	-0.103320531	0.998825	Has domain(s) with predicted translation initiation factor activity, role in translational initiation and cytoplasm localization
orf19.4940	orf19.4940	178.6	137.85	113.49	90.61	-0.539062978	0.160353	Putative histidine permease; fungal-specific (no human or murine homolog); Hap43p-induced gene
orf19.6340	orf19.6340	145.09	137.57	118.5	149.98	-0.068258865	0.998825	Ortholog(s) have RNA polymerase I activity, RNA polymerase II activity, RNA polymerase III activity, RNA-directed RNA polymerase activity
orf19.6883	orf19.6883	157.36	137.5	176.7	187.28	0.257602345	0.797	Putative oxysterol binding protein family; probable peripheral membrane protein of the Golgi complex; flow model and Spider biofilm repressed
orf19.1517	ARO3	134	137.48	128.26	115.11	-0.144637468	0.998825	3-deoxy-D-arabinoheptulosonate-7-phosphate synthase; aromatic amino acid synthesis; GCN-regulated; feedback-inhibited by phe if expressed in S. cerevisiae; decreased in stationary phase; flow model biofilm repressed
orf19.6793	orf19.6793	151.85	137.25	171.41	155.46	0.138637551	0.998825	Protein of unknown function; Sef1, Sfu1, and Hap43 regulated; rat catheter and Spider biofilm induced
orf19.1880	HEM15	146.57	137.15	126.03	123.56	-0.165549232	0.987611	Putative ferrochelatase involved in heme biosynthesis; transcript not regulated by iron levels and not affected by a yfh1 null mutation; Spider biofilm repressed
orf19.590	orf19.590	152.35	137.14	150.47	156.48	0.06171539	0.998825	Putative thiamine biosynthesis enzyme; decreased expression in an ssr1 null mutant; protein present in exponential and stationary growth phase yeast cultures

orf19.3126	CCT6	136.8	137.08	130.47	136.58	-0.041589773	0.998825	Putative cytosolic chaperonin Cct ring complex subunit; mutation confers hypersensitivity to cytochalasin D; GlcNAc-induced protein; Spider biofilm repressed
orf19.4004	CCT3	137.93	136.83	143.86	148.22	0.063342999	0.998825	Putative cytosolic chaperonin Cct ring complex subunit; mutation confers hypersensitivity to cytochalasin D
orf19.6355	orf19.6355	161.44	136.72	131.27	119.5	-0.224222951	0.881465	Ortholog(s) have role in ribosome biogenesis and cytosol, nucleolus localization
orf19.4099	ECM17	149.38	136.37	194.75	199.82	0.39872032	0.357668	Putative sulfite reductase beta subunit; role in cell wall biogenesis; regulated by Tsa1/Tsa1B in H2O2 stress; Gcn4-regulated; Tbf1-activated; Hap43-repressed; Spider, flow, F-12/CO2 model biofilm induced
orf19.7290	orf19.7290	161.6	136.3	160.78	159.15	0.079671073	0.998825	Ortholog(s) have RNA cap binding activity, role in deadenylation-dependent decapping of nuclear-transcribed mRNA and cytoplasmic mRNA processing body, cytosol, mRNA cap binding complex, nucleus localization
orf19.7154	UTP18	159.62	136.16	117.97	116.69	-0.289299034	0.748209	Putative U3 snoRNA-associated protein; Hap43-induced; repressed in core stress response; physically interacts with TAP-tagged Nop1
orf19.6720	orf19.6720	161.43	136.11	75.87	73.26	-0.824627691	0.006439	P-Loop domain-containing protein of unknown function; transposon mutation affects filamentous growth; Spider biofilm induced
orf19.3169	orf19.3169	153.72	135.73	117.51	113.73	-0.28071214	0.780572	Ortholog(s) have role in RNA polymerase II complex localization to nucleus, RNA polymerase III complex localization to nucleus, establishment of mitotic sister chromatid cohesion and cytosol, nucleus localization
orf19.976	BRE1	117.13	135.72	118.35	109.73	-0.137961101	0.998825	Putative transcription factor with C3HC4 zinc finger DNA-binding motif; similar to S. cerevisiae Bre1p; transposon mutation affects filamentous growth

orf19.3934	CAR1	160.61	135.61	145.17	92	-0.281987613	0.811201	Arginase; arginine catabolism; transcript regulated by Nrg1, Mig1, Tup1; colony morphology-related regulation by Ssn6; alkaline induced; protein decreased in stationary phase; sumoylation target; flow model biofilm induced
orf19.2511.2	orf19.2511.2	130.82	135.58	106.49	114.11	-0.242896783	0.87024	Ortholog of C. parapsilosis CDC317 : CPAR2_103040, Candida tenuis NRRL Y-1498 : CANTEDRAFT_116324, Debaryomyces hansenii CBS767 : DEHA2G01980g and Pichia stipitis Pignal : PICST_45936
orf19.6369	RIO2	145.92	135.37	118.23	112.34	-0.253330516	0.846842	Putative serine kinase with a predicted role in the processing of the 20S pre-rRNA into mature 18S rRNA; null mutants are hypersensitive to caspofungin
orf19.3663.1	orf19.3663.1	162.13	135.32	223.7	210.25	0.467403639	0.192542	Predicted plasma membrane protein; gene has intron
orf19.4679	AGP2	121.96	135.3	86.7	85.14	-0.485469439	0.280569	Amino acid permease; hyphal repressed; white-opaque switch regulated; induced in core caspofungin response, during cell wall regeneration, by flucytosine; regulated by Sef1, Sfu1, and Hap43; rat catheter and Spider biofilm induced
orf19.7445	orf19.7445	142.36	135.3	115.88	96.35	-0.334054057	0.627605	Ortholog of S.c. Vid24; a peripheral membrane protein located at Vid (vacuole import and degradation) vesicles; regulated by Sef1, Sfu1, and Hap43; Spider biofilm induced
orf19.5995	MCA1	131.46	135.16	133.74	116.39	-0.088122051	0.998825	Putative cysteine protease with similarity to S. cerevisiae Mca1p; has a role in apoptotic cell death upon H2O2 treatment; fungal-specific (no human or murine homolog); farnesol-induced
orf19.6008.4	orf19.6008.4	156.17	135.14	193.71	226.92	0.453087836	0.240742	Ortholog(s) have mitochondrion localization
orf19.2770.1	SOD1	126.29	135.14	295.01	318.52	1.07153093	1.70E-06	Cytosolic copper- and zinc-containing superoxide dismutase; role in protection from oxidative stress; required for full virulence; alkaline induced by Rim101; induced by human blood; rat catheter, flow model and Spider biofilm repressed

orf19.4658	orf19.4658	138.5	135.13	128.11	148.06	0.000485121	0.998825	RING finger and CHY zinc finger domain-containing protein; mutant are viable
orf19.1971	TRY3	142.78	135.08	79.21	81.67	-0.656275733	0.055379	RING-finger transcription factor; regulator of yeast form adherence; required for yeast cell adherence to silicone substrate; Spider biofilm induced
orf19.1416	COX11	146.65	135.03	142.1	119.43	-0.106182171	0.998825	Cytochrome oxidase assembly protein; transcript regulated by Nrg1; protein repressed during the mating process; Hap43-repressed gene; rat catheter biofilm induced
orf19.6881	YTH1	97.44	134.82	104.58	117.26	-0.064662075	0.998825	Putative mRNA cleavage and polyadenylation specificity factor; transcription is regulated upon yeast-hyphal switch; decreased expression in hyphae compared to yeast-form cells; fluconazole or flucytosine induced
orf19.3724	orf19.3724	142.34	134.78	116.66	103.49	-0.289934373	0.756052	Ortholog(s) have role in endonucleolytic cleavage in 5'-ETS of tricistronic rRNA transcript (SSU-rRNA, 5.8S rRNA and LSU-rRNA), more
orf19.2299	orf19.2299	137.36	134.62	116.18	134.24	-0.110585921	0.998825	Ortholog(s) have protein tag activity and role in cell budding, cellular response to oxidative stress, invasive growth in response to glucose limitation, protein urmylation, tRNA wobble position uridine thiolation
orf19.2720	orf19.2720	147.92	134.46	129.04	119.46	-0.166963511	0.98704	Cytosolic chaperonin Cct ring complex subunit; role in the assembly of actin and tubulins; Spider biofilm repressed
orf19.1826	MDM34	117.83	134.25	71.12	60.41	-0.766542229	0.021711	Putative zinc finger transcription factor; macrophage-downregulated; flow model biofilm induced; flow model and Spider biofilm induced
orf19.260	SLD1	118.35	133.95	145.66	158.77	0.220769755	0.892679	Sphingolipid delta-8 desaturase; catalyzes desaturation at C8 in the long-chain base moiety of ceramides in glucosylceramide synthesis, important for virulence; ketoconazole and hypoxia induced; Hap43-repressed; Spider biofilm induced

orf19.962	orf19.962	152.23	133.93	120.88	120.45	-0.217419828	0.894293	Protein with a fungal RNA polymerase I subunit RPA14 domain; proposed to play a role in the recruitment of pol I to the promoter; Hap43-induced gene
orf19.733	orf19.733	150.63	133.89	68.92	65.6	-0.885761175	0.002933	Ortholog(s) have endoplasmic reticulum localization
orf19.2618	MET2	122.27	133.79	156.26	150.87	0.209816013	0.90951	Homoserine acetyltransferase; Hap43p-, Gcn4p-regulated; macrophage/pseudohyphal-repressed; not highly biofilm induced, in contrast to many sulfur amino acid metabolic genes; no human or murine homolog; virulence-group-correlated expression
orf19.3967	PFK1	122.38	133.76	168.31	176.03	0.351767173	0.543746	Phosphofructokinase alpha subinit; activated by fructose 2,6-bisphosphate, AMP, ATP inhibited; activity reduced on hyphal induction; phagocytosis-repressed; fluconazole, flow model biofilm induced; rat catheter and Spider biofilm repressed
orf19.6189	orf19.6189	128.71	133.49	94.94	101.31	-0.356237112	0.587706	Chalcone related protein family; flow model biofilm induced
orf19.5369	orf19.5369	144.92	133.45	175.1	178.29	0.287173813	0.71162	Uroporphyrinogen decarboxylase; catalyzes the 5th step in the heme biosynthetic pathway; flow model and Spider biofilm repressed
orf19.6540	PFK2	114.33	132.89	149.97	152.93	0.23572818	0.870631	Phosphofructokinase beta subunit; fructose 2,6-bisphosphate, AMP activated; ATP inhibited; phagocytosis, hyphal repressed; fluconazole-induced; stationary-phase enriched; flow model biofilm induced; rat catheter/Spider biofilm repressed
orf19.4477	CSH1	138.3	132.87	247.5	231.63	0.706650524	0.007228	Aldo-keto reductase; role in fibronectin adhesion, cell surface hydrophobicity; regulated by temperature, growth phase, benomyl, macrophage interaction; azole resistance associated; Spider biofilm induced; rat catheter biofilm repressed
orf19.411	orf19.411	135.98	132.79	91.21	105.35	-0.385679753	0.51998	Protein similar to GTPase regulators; induced in low iron; transcript activated by Mnl1 under weak acid stress; Hap43-, Sfu1- and Sef1-regulated; flow model biofilm induced, Spider biofilm induced

orf19.2075	DFG5	123.41	132.72	140.38	147.59	0.132354826	0.998825	N-linked mannoprotein of cell wall and membrane; possible signal transducer; role in hyphal growth and HWP1 induction in alkaline pH; GPI modification predicted; dfg5 dcw1 double mutant is inviable; caspofungin-induced, fungal-specific
orf19.5445	GLO3	130.29	132.45	185.57	156.24	0.315129226	0.636186	Putative ARF GTPase activator; role in COPI coating of Golgi vesicle, ER to Golgi vesicle-mediated transport, retrograde Golgi to ER vesicle-mediated transport; Spider biofilm repressed
orf19.1646	orf19.1646	141.79	132.11	115.78	101.39	-0.292646378	0.752654	Ortholog(s) have rRNA primary transcript binding activity
orf19.7281	PDK2	147.75	132.06	152.67	113.09	-0.075026041	0.998825	Putative pyruvate dehydrogenase kinase; mutation confers hypersensitivity to amphotericin B
orf19.5462	orf19.5462	143.18	131.97	140.4	129.85	-0.03300679	0.998825	Protein of unknown function
orf19.7121	orf19.7121	141.4	131.97	155.78	152.58	0.142570637	0.998825	Protein of unknown function; Spider biofilm repressed
orf19.5758	SAL6	139.49	131.76	127.78	119.09	-0.12366438	0.998825	Putative protein phosphatase of the Type 1 family; serine/threonine-specific; similar to S. cerevisiae Ppq1; mutant has virulence defect; Spider biofilm induced
orf19.1936	SNF1	138.57	131.34	141.19	130.47	-0.006406895	0.998825	Essential protein; functional homolog of S. cerevisiae Snf1p regulator of sugar metabolism; constitutively expressed; Thr208 phosphorylation may have regulatory role; up-regulation associated with azole resistance; has polyhistidine tract
orf19.7433	orf19.7433	139.13	131.32	155.02	147.43	0.12719632	0.998825	Ortholog(s) have nucleocytoplasmic transporter activity, structural constituent of nuclear pore activity

orf19.6752	orf19.6752	126.64	131.21	131.18	143.3	0.063148419	0.998825	Ortholog(s) have structural constituent of ribosome activity and mitochondrial small ribosomal subunit localization
orf19.5961	orf19.5961	146.16	131.09	113	119.8	-0.217815908	0.901691	Ortholog(s) have role in proteasome regulatory particle assembly, proteolysis and cytosol, nucleus, proteasome regulatory particle localization
orf19.4780	orf19.4780	134.79	130.94	145.68	120.14	-0.012753538	0.998825	Predicted MFS family membrane transporter, member of the drug:proton antiporter (12 spanner) (DHA1) family; Spider biofilm induced
orf19.6660	orf19.6660	126.64	130.93	100.77	110.11	-0.250338391	0.861125	Protein of unknown function; mRNA binds to She3; Hap43-repressed; rat catheter and flow model biofilm induced
orf19.6608	orf19.6608	135.44	130.87	156.58	137.81	0.114921055	0.998825	Predicted DDE superfamily endonuclease domain; repression correlates with clinical development of fluconazole resistance; Spider biofilm induced
orf19.4044	MUM2	122.33	130.74	166.42	163.53	0.314581452	0.645919	Protein similar to S. cerevisiae Mum2, a protein essential for meiotic DNA replication and sporulation; induced by alpha pheromone in SpiderM medium; transcript regulated by Tup1
orf19.5688	orf19.5688	139.18	130.43	123.77	125.73	-0.098584675	0.998825	Predicted ORF from Assembly 19; removed from Assembly 20; subsequently reinstated in Assembly 21 based on comparative genome analysis
orf19.4670	CAS5	140.51	129.91	92.22	80.62	-0.544182317	0.170065	Transcription factor; cell wall damage response; required for adherence, response/resistance to caspofungin; repressed in core stress response; mutants have reduced CFU in mice, hyphal defect in C. elegans infection; Spider biofilm induced
orf19.2504	BMS1	137.02	129.75	114.32	107.11	-0.238885819	0.870631	Putative GTPase; Hap43-induced gene; mutation confers resistance to 5-fluorocytosine (5-FC); flucytosine induced; repressed by prostaglandins; Spider biofilm induced

orf19.3124	orf19.3124	132.65	129.74	111.81	114.61	-0.187538131	0.951606	Ortholog(s) have mRNA binding, metalloaminopeptidase activity, role in negative regulation of gene expression, protein initiator methionine removal involved in protein maturation and cytosolic ribosome, nucleolus localization
orf19.4932	orf19.4932	132.68	129.72	129.74	131.69	-0.014793753	0.998825	Ortholog(s) have role in mitochondrial translation and mitochondrion localization
orf19.7119	RAD3	131.73	129.35	125.45	133.16	-0.023563479	0.998825	Ortholog of S. cerevisiae Rad3; 5' to 3' DNA helicase, nucleotide excision repair and transcription, subunit of RNA polII initiation factor TFIIH and Nucleotide Excision Repair Factor 3 (NEF3)
orf19.4246	orf19.4246	135.68	128.91	260.63	238.03	0.786853142	0.001629	Protein with similarity to S. cerevisiae Ykr070w; Tn mutation affects filamentation; Hog1-repressed; colony morphology-related gene regulation by Ssn6p; induced during cell wall regeneration; possibly essential
orf19.7150	NRG1	154.44	128.66	190.01	194.16	0.372006793	0.444628	Transcription factor/repressor; regulates chlamydospore formation/hyphal gene induction/virulence and rescue/stress response genes; effects both Tup1 dependent and independent regulation; flow model biofilm induced; Spider biofilm repressed
orf19.2150	orf19.2150	150.17	128.59	162.88	162.23	0.179801616	0.946319	Putative ortholog of mammalian electron transfer flavoprotein complex subunit ETF-alpha; Spider biofilm repressed
orf19.5408	HRK1	125.27	128.59	135.08	133.61	0.058580006	0.998825	Putative serine/threonine kinase; predicted role in cellular ion homeostasis; Spider biofilm repressed
orf19.5833	orf19.5833	152.71	128.56	125.51	111.04	-0.220682229	0.893138	Ortholog(s) have polyubiquitin binding activity
orf19.6627	orf19.6627	153.9	128.48	117.79	96.09	-0.342021154	0.61093	Protein of unknown function; possibly transcriptionally regulated upon hyphal formation

orf19.6888	orf19.6888	107	128.47	92.83	97.41	-0.261375049	0.8593	Zn(II)2Cys6 domain transcription factor; regulated by Mig1 and Tup1; rat catheter and Spider biofilm induced
orf19.4836	URA1	134.92	128.15	163.17	156.67	0.231290893	0.868035	Dihydroorotate dehydrogenase; de novo pyrimidine biosynthesis; regulated by yeast-hypha switch, Nrg1/Mig1/Tup1; flow model biofilm induced; rat catheter and Spider biofilm repressed
orf19.3302	orf19.3302	97.98	128.02	97.02	114.15	-0.091660157	0.998825	Putative type-1 protein phosphatase targeting subunit; transcript repressed by yeast-hyphal switch; transcript induced by Mnl1p under weak acid stress; flow model biofilm induced
orf19.7485	orf19.7485	160.1	127.82	148.1	142.03	0.000377694	0.998825	Putative mitochondrial ribosomal protein of the large subunit; Hap43p-repressed gene; ortholog of S. cerevisiae MRPL9
orf19.4043	orf19.4043	102.88	127.7	153.99	152.37	0.325463556	0.651869	Protein with a predicted pleckstrin homology domain; induced by alpha pheromone in SpiderM medium
orf19.6754	orf19.6754	103.22	127.48	87.34	94.6	-0.284023124	0.828174	Protein of unknown function; Spider biofilm induced
orf19.6607	orf19.6607	154.49	127.44	177.87	158.38	0.2112429	0.893138	Ortholog(s) have role in mitochondrial respiratory chain complex I assembly and mitochondrial membrane localization
orf19.4204	orf19.4204	125.17	127.43	144.01	147.48	0.165040966	0.988116	Ortholog(s) have structural constituent of ribosome activity and mitochondrial small ribosomal subunit localization
orf19.5200	SIT4	137	127.36	118.28	126	-0.104121193	0.998825	Serine/threonine protein phosphatase catalytic subunit; has a role in cell wall maintenance, hyphal growth, and virulence in a mouse systemic infection model

orf19.5797	PLC2	160.46	127.28	94.24	81.57	-0.589931884	0.106451	Phosphatidylinositol (PtdIns)-specific phospholipase C (PI-PLC); predicted type 2 membrane protein; role in, and regulated by, filamentation, Nrg1 and Tup1; no mouse systemic virulence role; orf19.5797 and orf19.1586 are almost identical
orf19.563	RRP15	145.05	127.12	109.73	99.77	-0.32116014	0.672918	Putative nucleolar protein; constituent of pre-60S ribosomal particles; Hap43-induced; repressed by prostaglandins
orf19.5356	orf19.5356	140.42	127.05	117.07	118.62	-0.158476618	0.998825	Protein with a predicted role in cell wall integrity; repressed in core stress response
orf19.5835	orf19.5835	144.41	127.01	113.24	104.65	-0.272247001	0.81271	Ortholog(s) have role in rRNA processing, ribosomal small subunit assembly and CURI complex, UTP-C complex, cytosol localization
orf19.7363	KRE6	116.45	126.35	103.3	102.97	-0.203104844	0.938642	Essential beta-1,6-glucan synthase subunit; change in mRNA length, not abundance, at yeast-hypha transition; alkaline induced by Rim101, on cell wall regeneration; Spider biofilm induced; Bcr1-repressed in RPMI a/a biofilms
orf19.2170	PHM7	120.08	126.18	165.02	146.74	0.277884815	0.777732	Putative transporter; fungal-specific; Hog1-repressed; repressed by 17-beta-estradiol, ethynyl estradiol; Hap43-induced; Spider biofilm induced
orf19.6701	orf19.6701	122.54	126.06	138.74	135.83	0.109559996	0.998825	Protein with similarity to amino acid-tRNA ligase; stationary phase enriched protein; GlcNAc-induced protein
orf19.4031	orf19.4031	125.03	125.88	97.68	104.32	-0.268727483	0.838939	Ortholog(s) have cytoplasm localization
orf19.5126	orf19.5126	121.05	125.87	125.73	118.63	-0.021675255	0.998825	Putative adhesin-like protein

orf19.1566	UTP21	142.1	125.58	111.14	97.85	-0.309156627	0.71162	Putative U3 snoRNP protein; Hap43-induce; physically interacts with TAP-tagged Nop1; Spider biofilm induced
orf19.6634	VMA2	124.71	125.39	196.15	194.01	0.54129864	0.100993	Vacuolar H(+)-ATPase; protein present in exponential and stationary growth phase yeast cultures; plasma membrane localized; amphotericin B repressed, caspofungin repressed
orf19.7488	orf19.7488	129.66	125.39	94.17	110.98	-0.267352192	0.842252	Component of the SSU processome; predicted role in pre-18S rRNA processing; Spider biofilm induced
orf19.4537	orf19.4537	105.95	125.28	106.49	110.34	-0.091570912	0.998825	Ortholog(s) have RNA polymerase II core binding, RNA polymerase II regulatory region DNA binding, core RNA polymerase II recruiting transcription factor activity
orf19.2095	orf19.2095	123.05	124.86	115.61	121.52	-0.059691952	0.998825	Ortholog(s) have role in negative regulation of transcription from RNA polymerase II promoter in response to iron and cytosol, nucleus localization
orf19.5022	orf19.5022	102.36	124.76	102.84	104.02	-0.121336488	0.998825	Ortholog(s) have inorganic cation transmembrane transporter activity and role in cellular cobalt ion homeostasis, cellular manganese ion homeostasis, cobalt ion transport, manganese ion transport
orf19.1248	orf19.1248	137.19	124.71	140.96	134.59	0.053618907	0.998825	Putative RNA polymerase II subunit B44; heterozygous null mutant exhibits resistance to parnafungin in the C. albicans fitness test; Spider biofilm repressed
orf19.4268	UTP13	134.43	124.46	113.82	103.11	-0.218827047	0.907463	Putative U3 snoRNA-associated protein; Hap43-induced; repressed in core stress response; physically interacts with TAP-tagged Nop1
orf19.2639.1	orf19.2639.1	120.12	124.4	140.3	169.36	0.277497255	0.789669	Lsm (Like Sm) protein; predicted role in involved in mRNA decay; Spider biofilm repressed

orf19.7286	RPN7	146.6	124.27	137.81	131.07	-0.018012659	0.998825	Subunit of the proteasome regulatory particle; regulated by Gcn2p and Gcn4p; protein present in exponential and stationary growth phase yeast cultures
orf19.1940	orf19.1940	125.84	124.25	163.83	167.35	0.335003144	0.581559	Ortholog(s) have role in cellular response to cadmium ion, detoxification of cadmium ion, ubiquinone biosynthetic process and mitochondrial inner membrane localization
orf19.7438	UBA1	132.24	124.22	159.35	146.72	0.208132383	0.90951	Ubiquitin-activating enzyme; protein level decreases in stationary phase cultures
orf19.3243	SRP54	134.66	124.05	127.64	126.36	-0.033347806	0.998825	Putative signal recognition particle (SRP) subunit; induced in ssr1 null; Spider biofilm repressed
orf19.1677	orf19.1677	109.27	124.02	118.02	134.85	0.08775272	0.998825	Protein of unknown function; transcript detected on high-resolution tiling arrays
orf19.2115	orf19.2115	123.49	124.02	101.3	112.53	-0.180207406	0.974208	Putative molybdopterin-converting factor; fungal-specific (no human or murine homolog)
orf19.2250	SPE3	132.01	123.91	130.81	145.95	0.087443898	0.998825	Putative spermidine synthase; predicted role in pantothenate and spermidine biosynthesis; Spider biofilm repressed
orf19.2498	orf19.2498	110.74	123.69	88.24	86	-0.364097239	0.598332	Predicted ubiquitin-protein ligase; Hap43-repressed gene; oxidative stress-induced via Cap1; flow model biofilm induced
orf19.4488	orf19.4488	114.07	123.68	133.03	157.76	0.232746552	0.880321	Predicted ortholog of S. cerevisiae Swi3, subunit of the SWI/SNF chromatin remodeling complex; possibly an essential gene, disruptants not obtained by UAU1 method

orf19.7011	orf19.7011	127.23	123.57	113.06	109	-0.158266557	0.998825	Ortholog(s) have 90S preribosome, cytoplasm, mitotic spindle pole body, nucleolus localization
orf19.4089	SGT1	126.33	123.57	137.42	134.19	0.087421478	0.998825	Putative co-chaperone protein with a predicted role in kinetochore assembly; mutation confers hypersensitivity to radicicol; sumoylation target
orf19.5615	AYR2	159.13	123.07	204.77	193.48	0.420479258	0.312123	Putative NADPH-dependent 1-acyl dihydroxyacetone phosphate reductase; shows colony morphology-related gene regulation by Ssn6p
orf19.3333	orf19.3333	139.96	123.03	110.52	95.94	-0.297419562	0.749476	Ortholog(s) have poly(A) RNA binding activity, role in mRNA polyadenylation, poly(A)+ mRNA export from nucleus, regulation of mRNA stability and cytoplasm, nucleus localization
orf19.4815	YTM1	145.44	123.02	111.18	101.3	-0.291079407	0.768363	Protein similar to S. cerevisiae Ytm1p, which is involved in biogenesis of the large ribosomal subunit; transposon mutation affects filamentous growth; protein level decreases in stationary phase cultures; Hap43p-induced gene
orf19.723	BCR1	135.09	122.82	119.47	114.35	-0.132978157	0.998825	Transcription factor; regulates a/alpha biofilm formation, matrix, cell-surface-associated genes; confers adherence, impermeability, impenetrability, fluconazole resistance; Tup1/Tec1/Mnl1-regulated; mRNA binds She3; Spider biofilm induced
orf19.2005	REG1	107.75	122.55	95.07	88.68	-0.279778295	0.826113	Putative protein phosphatase regulatory subunit; Hap43-repressed gene; macrophage/pseudohyphal-induced; possibly regulated upon hyphal formation; flow model biofilm induced
orf19.3022	orf19.3022	113.1	122.37	133.41	128.5	0.119217162	0.998825	Ortholog(s) have structural constituent of ribosome activity and mitochondrial small ribosomal subunit localization
orf19.4084	KIS1	125.28	122.07	117.71	119.01	-0.059945221	0.998825	Snf1p complex scaffold protein; similar to S. cerevisiae Gal83p and Sip2p with regions of similarity to Sip1p (ASC and KIS domain); interacts with Snf4p; mutants are hypersensitive to caspofungin and hydrogen peroxide; Hap43p-repressed gene

orf19.6424	orf19.6424	135.48	122.03	124.07	105.13	-0.15011679	0.998825	Ortholog(s) have SUMO ligase activity and role in cellular response to DNA damage stimulus, hyphal growth, mitotic spindle elongation, protein sumoylation, regulation of mitotic cell cycle
orf19.7394	GDA1	117.9	121.98	76.16	86.91	-0.460328851	0.359369	Golgi membrane GDPase, required for wild-type O-mannosylation, not N-glycosylation; required for wild-type hyphal induction, cell wall, and cell surface charge; not required for HeLa cell adherence; functional homolog of S. cerevisiae Gda1p
orf19.400	GCF1	125.86	121.94	145.16	117.46	0.053498265	0.998825	HMG box mitochondrial protein; binds to mt DNA and the HWP1 promoter; mutant phenotype and functional complementation of an S. cerevisiae abf2 mutation suggest role in mt genome replication, maintenance; flow and Spider biofilm repressed
orf19.4290	TRR1	143.76	121.75	136.72	125.31	-0.027957267	0.998825	Thioredoxin reductase; regulated by Tsa1/Tsa1B, Hap43; induced by nitric oxide, peroxide; oxidative stress-induce via Cap1; induced by human neutrophils; stationary phase enriched protein
orf19.4449	CCS1	118.39	121.67	123.68	128.21	0.048203052	0.998825	Copper chaperone involved in activation and protection of superoxide dismutase Sod1p
orf19.2416.1	MLC1	118.45	121.62	150.64	219.67	0.514955014	0.212137	Microtubule-dependent localized protein; at Spitzenkorper and cytokinetic ring in hyphae; cell-cycle dependent localization to tip polarisome, bud neck in yeast and pseudohyphae; sumoylation target; rat catheter, Spider biofilm repressed
orf19.6902	DBP7	142.02	121.41	101.12	98.04	-0.342043269	0.616255	Putative ATP-dependent DEAD-box RNA helicase; Hap43-induced; rat catheter biofilm induced
orf19.6245	orf19.6245	118.04	121.38	109.01	114.94	-0.0876768	0.998825	Protein of unknown function; regulated by osmotic stress via Hog1 and oxidative stress (Hog1- and Cap1-independent); induced by alpha pheromone in SpiderM medium; Spider biofilm induced
orf19.3195	HIP1	139.44	121.06	132.49	131.4	0.005127526	0.998825	Similar to amino acid permeases; alkaline, flucytosine induced; regulated by Plc1, Gcn2 and Gcn4; fungal-specific; Spider biofilm induced

orf19.512	orf19.512	128.43	120.92	119.82	122.22	-0.044039979	0.998825	Ortholog of S. cerevisiae Kre33; essential; S. cerevisiae ortholog is essential and is required for biogenesis of the small ribosomal subunit
orf19.7057	GUS1	117.55	120.65	146.02	156.35	0.274773846	0.789669	Putative glutamine-tRNA ligase; stationary phase enriched protein; Spider biofilm repressed
orf19.4317	GRE3	117.5	120.49	153.61	162.43	0.340954102	0.581559	Putative D-xylose reductase; antigenic in murine systemic infection; soluble protein in hyphae; induced by farnesol, macrophage interaction and by Mnl1 under weak acid stress; stationary-phase enriched protein; Spider biofilm induced
orf19.2594	orf19.2594	136.56	120.32	111.01	107.6	-0.201399165	0.938404	Ortholog(s) have RNA polymerase I activity, role in transcription of nuclear large rRNA transcript from RNA polymerase I promoter and DNA-directed RNA polymerase I complex, cytosol localization
orf19.7654	CPR6	136.27	120.14	128.31	114.07	-0.078320532	0.998825	Putative peptidyl-prolyl cis-trans isomerase; macrophage/pseudohyphal-repressed; heavy metal (cadmium) stress-induced; heterozygous null mutant displays sensitivity to virgineone; rat catheter biofilm induced
orf19.3064	MRPL27	108.08	120	112.79	118.89	0.009478775	0.998825	Putative 60S ribosomal protein L27, mitochondrial precursor
orf19.1960	CLN3	130.28	119.82	140.85	123.88	0.059587304	0.998825	G1 cyclin; depletion abolishes budding and causes hyphal growth defects; farnesol regulated, functional in S. cerevisiae; possibly essential (UAU1 method); other biofilm induced; Spider biofilm induced
orf19.6202	RBT4	99.2	119.76	246.98	270.71	1.053514204	1.45E-05	Pry family protein; required for virulence in mouse systemic/rabbit corneal infections; not filamentation; mRNA binds She3, is localized to hyphal tips; Hap43-induced; in both yeast and hyphal culture supernatants; Spider biofilm induced
orf19.4304	GAP1	125.98	119.57	107.12	109.07	-0.165384249	0.998825	Amino acid permease; antigenic in human/mouse; 10-12 transmembrane regions; regulated by nitrogen source; alkaline, GlcNAc, phagocytosis induced; WT virulence in mice; Spider and flow model biofilm induced

orf19.5027	LCB2	115.12	119.53	161.6	153.08	0.346050739	0.568429	Putative serine palmitoyltransferase component; mutation confers hypersensitivity to aureobasidin A
orf19.213	orf19.213	159.93	119.34	127.47	124.68	-0.129604296	0.998825	Ortholog(s) have role in ubiquitin-dependent protein catabolic process and nucleus, proteasome regulatory particle, lid subcomplex, proteasome storage granule localization
orf19.1078	HBR2	148.72	119.26	137.52	117.02	-0.069727915	0.998825	Putative alanine glyoxylate aminotransferase; regulated by Gcn4p and hemoglobin; stationary phase enriched protein
orf19.6376	PTC5	114.39	119.14	120.56	114.34	-0.001598942	0.998825	Mitochondrial protein phosphatase of the Type 2C-related family (serine/threonine-specific), involved in drug response and cadmium tolerance
orf19.3337	orf19.3337	132.68	119.07	67.15	60.44	-0.80168147	0.012599	Protein of unknown function; merged with orf19.3338; rat catheter, flow and Spider model biofilm induced; promoter bound by Bcr1, Efg1, Ndt80, and Rob1; orf19.3338 Bcr1-repressed in RPMI a/a biofilms
orf19.7499.1	orf19.7499.1	88.57	118.83	86.01	76.67	-0.292493437	0.831295	Ortholog of C. parapsilosis CDC317 : CPAR2_800380, Candida tenuis NRRL Y-1498 : CANTEDRAFT_114079, Pichia stipitis Pignal : psti_CGOB_00127 and Candida orthopsilosis Co 90-125 : CORT_0A00460
orf19.390	CDC42	136.02	118.8	130.61	128.2	0.009811567	0.998825	Rho-type GTPase; required for budding and maintenance of hyphal growth; GGTase I geranylgeranylated; misexpression blocks hyphal growth, causes avirulence in mouse IV infection; shows actin-dependent localization to hyphal tip
orf19.5746	ALA1	120	118.69	128.7	119.24	0.033120054	0.998825	Alanyl-tRNA synthetase; translational regulation generates cytoplasmic and mitochondrial forms; Gcn4p-regulated; repressed by amino acid starvation (3-AT); translation-related genes downregulated upon phagocytosis by murine macrophages
orf19.3353	orf19.3353	115.87	118.63	115.28	88.94	-0.181005253	0.976613	Protein similar to a mitochondrial complex I intermediate-associated protein; fluconazole-repressed; Spider biofilm induced; rat catheter biofilm repressed

orf19.6458.1	orf19.6458.1	99.81	118.61	98.53	88.33	-0.198194214	0.951606	Ortholog(s) have U6 snRNA binding activity and role in cytoplasmic mRNA processing body assembly, mRNA splicing, via spliceosome
orf19.897	VPS20	100.21	118.5	121.17	112.86	0.064937245	0.998825	ESCRT III complex protein; role in multivesicular body (MVB) trafficking; required for processing of Rim8; Bcr1-repressed in RPMI a/a biofilms
orf19.392	orf19.392	140.34	118.43	176.32	162.6	0.327227611	0.600579	Ortholog(s) have role in mitochondrial respiratory chain complex III assembly and integral to mitochondrial membrane localization
orf19.1085	orf19.1085	140.95	118.42	155.88	158.27	0.228143269	0.877449	Ortholog(s) have ubiquitin-protein ligase activity
orf19.3481	orf19.3481	133.79	118.26	120.21	122.82	-0.052439514	0.998825	Putative mitochondrial ATP-dependent RNA helicase of the DEAD-box family, transcription is activated in the presence of elevated CO2
orf19.6065	orf19.6065	138.68	118.25	186.93	160.32	0.359445888	0.517955	RNA polymerase II holoenzyme/mediator subunit; regulated by Mig1, Tup1; amphotericin B, caspofungin repressed; protein present in exponential and stationary growth phase yeast; Hap43-repressed; Spider biofilm repressed
orf19.4967	COX19	136.23	118.15	87.41	91.36	-0.428499018	0.410883	Putative cytochrome c oxidase assembly protein; Plc1-regulated; rat catheter biofilm induced
orf19.3843	orf19.3843	107.53	118.09	137.34	132.1	0.197681396	0.938642	Ortholog(s) have role in filamentous growth, posttranslational protein targeting to membrane, translocation and Sec62/Sec63 complex localization
orf19.665	NEP1	126.65	118.08	125.36	113.74	-0.04062242	0.998825	Ortholog(s) have rRNA (pseudouridine) methyltransferase activity

orf19.2555	URA5	128.52	117.94	124.38	135.7	0.053827388	0.998825	Putative orotate phosphoribosyltransferase; protein abundance is affected by URA3 expression in the CAI-4 strain background; flucytosine induced; protein level decreased in stationary phase cultures
orf19.787	orf19.787	116.49	117.79	205.49	223.51	0.741454132	0.007141	Protein of unknown function
orf19.5645	MET15	120.5	117.77	243.02	241.21	0.873965931	0.00038	O-acetylhomoserine O-acetylserine sulfhydrylase; sulfur amino acid synthesis; immunogenic; Hog1, adherence-induced; brown color of mutant in Pb(2+) medium a visual selection; chlamydospore formation induced, F-12/CO2 biofilm induced
orf19.2717	SAS10	139.07	117.76	99.24	100.17	-0.313209482	0.712603	Putative U3-containing small subunit processome complex subunit; Hap43p-induced gene; mutation confers resistance to 5-fluorocytosine (5-FC); repressed upon high-level peroxide stress
orf19.5669	orf19.5669	109.08	117.7	143.33	133.94	0.228041219	0.885512	Protein with B-cell receptor-associated protein 31-like domain; membrane-localized protein
orf19.6706	GYP7	128.49	117.6	109.26	101.23	-0.199594856	0.938642	Protein similar to S. cerevisiae Gyp7p (GTPase-activating protein for Ypt1p); caspofungin-induced
orf19.6983	orf19.6983	128.24	117.48	135.71	117.95	0.03246063	0.998825	Protein of unknown function; Hap43-repressed gene; repressed by nitric oxide; Spider biofilm induced
orf19.4896	RPA34	133.91	117.46	107.94	99.45	-0.240124969	0.87796	Putative RNA polymerase I subunit; rat catheter biofilm induced
orf19.1805	PEX14	111.58	117.45	113.89	114.93	-0.010966816	0.998825	Ortholog(s) have peroxisome matrix targeting signal-1 binding, peroxisome matrix targeting signal-2 binding, protein binding, bridging activity

orf19.6776	GCD2	101.37	117.44	99.87	98.58	-0.11942512	0.998825	Putative translation initiation factor; genes encoding ribosomal subunits, translation factors, and tRNA synthetases are downregulated upon phagocytosis by murine macrophage
orf19.2369.1	ATX1	142.37	117.31	97.85	119.86	-0.20995179	0.931333	Putative cytosolic copper metallochaperone; flucytosine induced; Ssr1-repressed; rat catheter biofilm induced
orf19.4539	orf19.4539	109.64	117.31	108.65	97.42	-0.127657874	0.998825	Protein with a Rho GDP-dissociation inhibitor domain; Hap43-repressed gene; Spider biofilm induced
orf19.4358	orf19.4358	111.67	117.07	93.71	115.39	-0.115965224	0.998825	Putative protein of unknown function; Hap43p-repressed gene; S. cerevisiae ortholog YDL157C localizes to mitochondria
orf19.5773	orf19.5773	123.05	116.94	159.39	154.13	0.314853675	0.649978	Putative dipeptidyl-peptidase III; protein detected by mass spec in exponential and stationary phase cultures; Hog1p-induced; clade-associated gene expression
orf19.5083	DRG1	112.88	116.75	125.79	106.1	-0.002767101	0.998825	Member of the DRG family of GTP-binding proteins; involved in regulation of invasive filamentous growth
orf19.4220	orf19.4220	114.51	116.58	121.93	125.38	0.064857761	0.998825	Predicted pyridoxal 5'-phosphate synthase; regulated by Gcn4; repressed by amino acid starvation (3-AT treatment); rat catheter biofilm repressed
orf19.7115	SAC7	105.85	116.55	132.88	123.22	0.152712771	0.998825	Putative GTPase activating protein (GAP) for Rho1; repressed upon adherence to polystyrene; macrophage/pseudohyphal-repressed; transcript is upregulated in RHE model of oral candidiasis and in clinical oral candidiasis
orf19.84	CAN3	102.04	116.4	43.96	46.18	-0.984672524	0.002226	Predicted amino acid transmembrane transporter; transcript regulated by white-opaque switch; Hap43-repressed gene

orf19.3348	orf19.3348	110.92	116.39	127.84	129.6	0.14310325	0.998825	Ortholog(s) have structural constituent of ribosome activity and mitochondrial large ribosomal subunit localization
orf19.3656	COX15	115.51	116.24	105.64	110.1	-0.095161698	0.998825	Cytochrome oxidase assembly protein; transcript regulated by Nrg1 and Tup1; alkaline repressed; Hap43-repressed; early-stage flow model biofilm induced; Spider biofilm repressed
orf19.1426	orf19.1426	102.97	116.21	117.24	116.75	0.066754193	0.998825	Ortholog of S. cerevisiae Skg6; localizes to the cell cortex, cell bud neck, cell bud tip, incipient cellular bud site, and is membrane-bound; Spider biofilm induced
orf19.5438	orf19.5438	115.04	116.02	88.61	107	-0.201233076	0.946319	Putative ubiquitin-protein ligase; role in protein sumoylation, protein ubiquitination; Spider biofilm induced
orf19.898	HEM2	110.37	116.02	132.05	145.35	0.23509076	0.878136	Putative porphobilinogen synthase; induced in high iron; protein level decrease in stationary phase cultures; Spider biofilm repressed
orf19.3153	MSS4	115.97	115.93	86.77	80.66	-0.389455372	0.541043	Phosphatidylinositol-4-phosphate 5-kinase; activity induced by phosphatidic acid (Pld1 product); macrophage/pseudohyphal-repressed; mRNA binds to She3, localized to yeast cell buds and hyphal tips; Hap43-induced; Spider biofilm induced
orf19.5158	orf19.5158	114.43	115.89	127.63	112.19	0.03861294	0.998825	Protein with similarity to a human gene associated with colon cancer and to orf19.5158; regulated by Gcn4, Cyr1; induced by amino acid starvation; macrophage-induced protein, macrophage-repressed; Spider biofilm induced
orf19.6112	CTA2	112.2	115.87	176.19	164.89	0.481535212	0.218547	Putative transcription factor; Med2 mediator domain; activates transcription in 1-hybrid assay in S. cerevisiae; repressed by Efg1; member of a family of telomere-proximal genes; Tbf1-induced
orf19.451	SOK1	111.08	115.8	80.86	78.15	-0.425669736	0.448766	Protein kinase required for degradation of Nrg1p; mutants are sensitive to growth on hydrogen peroxide medium; induced by alpha pheromone in SpiderM medium; rat catheter and Spider biofilm induced

orf19.1967	orf19.1967	121.2	115.78	127.31	131.18	0.092956724	0.998825	Ortholog(s) have structural constituent of ribosome activity and mitochondrial large ribosomal subunit localization
orf19.7070	orf19.7070	127.05	115.73	106.71	111.24	-0.138732743	0.998825	Ortholog of Candida guilliermondii ATCC 6260 : PGUG_03499, Candida lusitaniae ATCC 42720 : CLUG_00410 and Candida albicans WO-1 : CAWG_05407
orf19.6246	orf19.6246	116.75	115.68	110.63	107.85	-0.084739277	0.998825	Ortholog(s) have peptide alpha-N-acetyltransferase activity, role in N-terminal peptidyl-methionine acetylation, cytoskeleton organization, mitochondrion inheritance and NatB complex, cytosol, nucleus localization
orf19.1910	orf19.1910	133.57	115.6	124.27	102.63	-0.126571169	0.998825	Predicted NADH-dependent flavin oxidoreductase; Hap43-repressed gene
orf19.7118	orf19.7118	124.65	115.42	179.02	157.18	0.402091113	0.39674	Ortholog(s) have adenylate kinase activity, nucleoside triphosphate adenylate kinase activity, role in nucleotide metabolic process and mitochondrial inner membrane localization
orf19.7610	PTP3	90.4	115.37	102.57	108.22	0.020653674	0.998825	Putative protein tyrosine phosphatase; hypha induced; alkaline induced; regulated by Efg1, Ras1, cAMP pathways; mutants are viable; Spider biofilm induced; rat catheter biofilm repressed; flow model biofilm repressed
orf19.7266	orf19.7266	153.78	115.3	152.07	149.4	0.130197006	0.998825	Ortholog of Candida albicans WO-1 : CAWG_00007
orf19.7502	orf19.7502	121.45	115.27	151.95	110.55	0.115881129	0.998825	Protein of unknown function; Hap43-induced gene; upregulated in a cyr1 null mutant; Spider biofilm induced
orf19.4585	TFG1	110.34	115.27	124.22	113.13	0.049915489	0.998825	Protein similar to S. cerevisiae Tfg1p, which is part of transcription factor TFIIF; transposon mutation affects filamentous growth; possibly an essential gene, disruptants not obtained by UAU1 method

orf19.7409	ERV25	129.82	115.22	157.91	178.56	0.382875954	0.463201	Component of COPII-coated vesicles; transcript induced upon filamentous growth; rat catheter biofilm repressed
orf19.2501	FLC1	108.35	115.15	108.14	101.49	-0.088902386	0.998825	Protein involved in heme uptake; putative FAD transporter, similar to S. cerevisiae Flc1; regulated by iron; macrophage-induced; mutant defective in filamentous growth; Spider biofilm induced
orf19.3932.1	orf19.3932.1	128.2	114.95	57.34	50.34	-0.941678397	0.002402	Has domain(s) with predicted nucleic acid binding, nucleotide binding activity
orf19.4845	TVP18	132.43	114.8	144.38	133.12	0.129693317	0.998825	Putative integral membrane protein; fluconazole-induced
orf19.6045	orf19.6045	122.56	114.78	136.22	114.45	0.047382038	0.998825	Putative phosphatidylserine decarboxylase; involved in phosphatidylethanolamine; Spider biofilm repressed
orf19.2017	orf19.2017	119.77	114.5	102.52	97.26	-0.202264783	0.938642	Ortholog(s) have RNA polymerase I activity and role in regulation of cell size, transcription elongation from RNA polymerase I promoter, transcription of nuclear large rRNA transcript from RNA polymerase I promoter
orf19.7156	FAA2-3	129.61	114.4	92.01	92.05	-0.342634145	0.636314	Predicted acyl CoA synthetase
orf19.7655	RPO21	105.64	114.35	108.94	109.77	-0.016688232	0.998825	RNA polymerase II; ortholog of S. cerevisiae Rpo21, transposon mutation affects filamentous growth; flow model biofilm repressed
orf19.7144	orf19.7144	137.9	114.22	127.26	108.07	-0.093844436	0.998825	Ortholog(s) have GTPase activity, ribosome binding activity, role in nonfunctional rRNA decay, nuclear-transcribed mRNA catabolic process, no-go decay, ribosome disassembly and cytosol localization

orf19.2727	GRX3	122.96	114.15	111.64	100.85	-0.138188972	0.998825	Putative glutaredoxin; flucytosine induced; regulated by Gcn4p; repressed in response to amino acid starvation (3-aminotriazole treatment)
orf19.2609	CET1	114.54	114.02	101.76	104.56	-0.129903745	0.998825	mRNA 5'-triphosphatase; large subunit of mRNA capping enzyme; positively regulates Ceg1 activity; functional homolog of S. cerevisiae Cet1; Cet1 and Cgt1 form a 2:1 complex; flow model biofilm induced
orf19.7069	orf19.7069	114.41	113.85	105.77	108.84	-0.079975125	0.998825	Putative AdoMet-dependent proline methyltransferase; Hap43-induced; required for normal flow model biofilm growth; Spider biofilm repressed
orf19.7140	orf19.7140	140.38	113.75	137.2	110.62	-0.039117442	0.998825	Putative catechol o-methyltransferase; stationary phase enriched protein; transcription upregulated in clinical isolates from HIV+ patients with oral candidiasis; Spider biofilm repressed
orf19.2257	orf19.2257	127.84	113.67	153.64	149.66	0.269438553	0.795758	Predicted ER protein involved in ER-nucleus signaling; Spider biofilm repressed
orf19.809	orf19.809	141.31	113.64	87.86	83.32	-0.478938636	0.294592	Ortholog(s) have role in maturation of LSU-rRNA from tricistronic rRNA transcript (SSU-rRNA, 5.8S rRNA, LSU-rRNA) and nucleolus, preribosome localization
orf19.233.1	HRT1	139.06	113.59	115.48	100.86	-0.198469355	0.943089	Ortholog of S. cerevisiae Hrt1; component of a nuclear ubiquitin-protein ligase complex involved in cell cycle control; induced by hydroxyurea; Spider biofilm induced
orf19.2270	SMF12	100.68	113.55	73.83	74.22	-0.440332544	0.432569	Ortholog of S. cerevisiae Smf1; manganese transporter; Gcn4-regulated; Hap43, alkaline induced; caspofungin repressed; mutants are viable
orf19.5683	orf19.5683	127.41	113.53	135.15	160.3	0.237777636	0.870631	Putative integral membrane protein of unknown function; clade-associated gene expression; Spider biofilm induced

orf19.7645	orf19.7645	114.16	113.47	132.52	113.61	0.089015325	0.998825	Biotin protein ligase; catalyzes covalent attachment of biotin to biotin-dependent enzymes including acetyl CoA carboxylase and pyruvate carboxylase
orf19.5749	SBA1	122.07	113.37	120.01	92.34	-0.13468829	0.998825	Similar to co-chaperones; induced in high iron; farnesol-, heavy metal (cadmium) stress-induced; protein level decreases in stationary phase cultures; Hap43-repressed
orf19.4998	ROB1	129.49	113.37	85.42	70.76	-0.522869244	0.231546	Zn(II)2Cys6 transcription factor; required for Spider model biofilm formation; mutant displays abnormal colony morphology and invasive growth; caspofungin repressed; flow model biofilm induced; rat catheter biofilm repressed
orf19.7388	PBS2	121.88	113.33	120.88	107.24	-0.04723085	0.998825	MAPK kinase (MAPKK); role in osmotic and oxidative stress responses, oxidative stress adaptation; required for stress regulation of Hog1p localization and activity; functional homolog of S. cerevisiae Pbs2p
orf19.4835	orf19.4835	126.02	113.29	94.29	79.49	-0.390666094	0.532956	Ortholog(s) have role in endonucleolytic cleavage in 5'-ETS of tricistronic rRNA transcript (SSU-rRNA, 5.8S rRNA and LSU-rRNA), more
orf19.3016	orf19.3016	108.15	113.24	135.29	120.35	0.159170477	0.998825	Ortholog(s) have role in meiotic chromosome segregation and cytosol, nucleus localization
orf19.3051	orf19.3051	120.56	113.21	171.46	167.09	0.44170373	0.291298	Protein of unknown function; S. pombe ortholog SPAC17A2.02c plays a role in resistance to cadmium; colony morphology-related gene regulation by Ssn6; Spider biofilm repressed
orf19.7131	orf19.7131	100.7	113.2	98.59	88.02	-0.17078081	0.998825	Butyrobetaine dioxygenase, the fourth enzyme of the carnitine biosynthesis pathway
orf19.2113	orf19.2113	113.27	113.1	135.35	126.85	0.166993744	0.994873	Putative integral peroxisomal membrane protein; Hap43p-repressed gene

orf19.4018	orf19.4018	119.69	113.08	102.75	128.12	-0.017076742	0.998825	Ortholog(s) have structural constituent of ribosome activity and mitochondrial small ribosomal subunit localization
orf19.815	DCK1	108.78	112.91	106.2	105.1	-0.071295948	0.998825	Putative guanine nucleotide exchange factor; required for embedded filamentous growth; activates Rac1; has a DOCKER domain; similar to adjacent DCK2 and to S. cerevisiae Ylr422wp; regulated by Nrg1; Spider biofilm induced
orf19.4190	PAM18	130.33	112.86	104.73	98.89	-0.217756818	0.918336	Predicted component of the presequence translocase-associated import motor (PAM complex) involved in protein import into mitochondrial matrix; rat catheter biofilm induced
orf19.3737	orf19.3737	105.16	112.79	136.1	122.9	0.193999999	0.946319	Protein with a Vps9 vacuolar protein sorting protein domain; Hap43-repressed; repressed by ciclopirox olamine; Spider biofilm induced
orf19.698	orf19.698	94.98	112.76	85.34	102.18	-0.132981308	0.998825	Integral ER membrane protein; predicted role in maintenance of ER zinc homeostasis; Spider biofilm induced
orf19.6317	ADE6	117.72	112.73	142.49	141.8	0.240812254	0.863837	5-Phosphoribosylformyl glycinamidine synthetase; adenine biosynthesis; not induced in GCN response, in contrast to S. cerevisiae Ade6; protein in stationary phase yeast-form cultures; flow model and rat catheter biofilm repressed
orf19.6345	RPG1A	114.54	112.39	114.5	103.53	-0.054132297	0.998825	Putative translation initiation factor; mutation confers hypersensitivity to roridin A and verrucarin A; repressed upon phagocytosis by murine macrophage; Spider biofilm repressed
orf19.2330	orf19.2330	108.22	112.22	98.86	85.58	-0.215760388	0.931333	Putative U3 snoRNA-associated protein; Hap43-induced; transposon mutation affects filamentous growth; repressed by prostaglandins
orf19.4248	orf19.4248	120.58	112.18	144.64	153.94	0.294667238	0.732635	Ortholog of S. cerevisiae YLR118C (alias Apt1); acyl-protein thioesterase responsible for depalmitoylation of Gpa1 in S. cerevisiae; regulated by Gcn4; repressed in response to amino acid starvation (3AT); rat catheter biofilm repressed

orf19.6041	RPO41	113.21	111.99	91.1	92.23	-0.25309924	0.87024	Putative mitochondrial RNA polymerase; repressed in core stress response; Spider biofilm induced
orf19.7523	MKC1	107.7	111.91	114.81	116.57	0.052606932	0.998825	MAP kinase; role in biofilm formation, contact-induced invasive filamentation, systemic virulence in mouse, cell wall structure/maintenance, caspofungin response; phosphorylated on surface contact, membrane perturbation, or cell wall stress
orf19.900	NSP1	120.43	111.9	140.28	132.27	0.182369011	0.956789	Essential component of the nuclear pore complex; sumoylation target; stationary phase enriched protein; rat catheter biofilm repressed
orf19.5170	ENA21	110.69	111.86	152.45	143.31	0.328308124	0.626697	Predicted P-type ATPase sodium pump; Gcn4p-regulated; flucytosine, amphotericin B, or ketoconazole-induced; osmotic stress-induced; overlaps orf19.5170.1, which is annotated as a blocked reading frame; Spider biofilm induced
orf19.895	HOG1	124.85	111.72	126	109.1	-0.021009622	0.998825	MAP kinase of osmotic-, heavy metal-, and core stress response; role in regulation of glycerol, D-arabitol in response to stress; phosphorylated in response to H2O2 (Ssk1-dependent) or NaCl; mutant induces protective mouse immune response
orf19.4443	YPD1	115.6	111.7	91.88	81.36	-0.335052705	0.677862	Phosphohistidine intermediate protein in a phosphorelay signal transduction pathway; residue His69 is the phosphoacceptor histidine; predicted to be soluble and cytosolic; functional homolog of S. cerevisiae Ypd1p
orf19.2998	TSR2	131.3	111.67	115.38	105.26	-0.128503524	0.998825	Protein with a predicted role in pre-rRNA processing; repressed by prostaglandins
orf19.5484	SER1	121.84	111.62	171.16	177.9	0.482292755	0.213372	Putative 3-phosphoserine aminotransferase; predicted role in serine and glycine biosynthesis; protein present in exponential and stationary yeast growth phases; Spider biofilm repressed
orf19.1986	ARO2	122.26	111.36	137.57	146.06	0.231490186	0.878136	Putative chorismate synthase; fungal-specific (no human or murine homolog); protein level decreased in stationary phase yeast cultures; GlcNAc-induced protein

orf19.6227	orf19.6227	109.61	111.28	86.77	95.44	-0.236727994	0.892679	Ortholog of C. dubliniensis CD36 : Cd36_06390, C. parapsilosis CDC317 : CPAR2_209040, Candida tenuis NRRL Y-1498 : CANTEDRAFT_114052 and Debaryomyces hansenii CBS767 : DEHA2D14300g
orf19.7109	orf19.7109	110.18	111.25	149.81	146.45	0.343653469	0.587511	Ortholog(s) have role in meiosis and cytosol, nucleus localization
orf19.1709	orf19.1709	120.16	110.71	227.17	231.74	0.847163182	0.000851	Sterol carrier domain protein; alkaline downregulated; colony morphology-related gene regulation by Ssn6; Spider biofilm induced
orf19.7619	orf19.7619	132.12	110.44	116.08	112.63	-0.07454187	0.998825	Protein with a mitochondrial distribution and morphology domain; possibly an essential gene, disruptants not obtained by UAU1 method; rat catheter and Spider biofilm induced
orf19.252	orf19.252	148.64	110.3	176.75	178.38	0.380360907	0.465203	Protein of unknown function; S. cerevisiae ortholog Fmp37 which localizes to mitochondria; Hap43-repressed; Spider biofilm repressed
orf19.999	GCA2	89.61	110.26	269.47	273.97	1.225172434	1.31E-07	Predicted extracellular glucoamylase; induced by ketoconazole; possibly essential, disruptants not obtained by UAU1 method; promotes biofilm matrix formation; Spider biofilm induced; Bcr1-induced in RPMI a/a biofilms
orf19.3198	OBPA	112.3	109.96	130.47	122.25	0.141311659	0.998825	Similar to oxysterol binding protein; non-sex gene located within the MTLa mating-type-like locus; Plc1p-regulated
orf19.2515	orf19.2515	115.93	109.92	139.22	126.42	0.181147421	0.957711	ZZ-type zinc finger protein; rat catheter and Spider biofilm induced
orf19.300	AIP2	121.79	109.89	155.2	149.03	0.320008645	0.645919	Putative actin interacting protein; regulated by Gcn4; induced in response to amino acid starvation (3-AT); repressed by elevated CO2; flow model biofilm repressed

orf19.2101	orf19.2101	116.37	109.88	129.88	135.28	0.182689888	0.957711	Ortholog(s) have protein anchor activity, role in protein insertion into ER membrane, retrograde vesicle-mediated transport, Golgi to ER and GET complex, mitochondrion localization
orf19.2090	orf19.2090	111.52	109.79	91.67	87.81	-0.256890072	0.863837	Ortholog of S. cerevisiae Ecm16, an essential DEAH-box ATP-dependent RNA helicase specific to the U3 snoRNP required for 18S rRNA synthesis; Hap43-induced; Spider biofilm induced
orf19.1584	orf19.1584	118.29	109.63	120.76	114.52	0.031163662	0.998825	Predicted MFS membrane transporter; monocarboxylate porter family member; colony morphology-related gene regulation by Ssn6; flow model biofilm induced; Spider biofilm induced
orf19.3564	RPC40	124.75	109.6	97.26	87.92	-0.292302134	0.789669	Putative RNA polymerase; protein level decreases in stationary phase cultures; Hap43p-induced gene
orf19.4307	orf19.4307	128.32	109.52	98.29	100.39	-0.226386596	0.90187	Ortholog(s) have role in late endosome to vacuole transport via multivesicular body sorting pathway, protein targeting to vacuole and late endosome localization
orf19.5184	orf19.5184	133.9	109.47	143.32	139.37	0.172530627	0.976613	Putative Ran guanyl-nucleotide exchange factor; probable signal transducer; Spider biofilm repressed
orf19.3401	CTA1	139.94	109.45	109.2	111.19	-0.15451317	0.998825	Protein similar to S. cerevisiae Mos10p, which affects S. cerevisiae filamentous growth; activates transcription in 1-hybrid assay in S. cerevisiae; protein levels increase under weak acid stress; nonessential
orf19.6587	GIR2	124.87	109.43	97.24	103.77	-0.187805931	0.957711	Ortholog of S. cerevisiae Gir2; transposon mutation affects filamentous growth
orf19.6417	TSR1	118.14	109.42	92.8	83.02	-0.309350422	0.748714	Component of 20S pre-rRNA processing unit; repressed by prostaglandins

orf19.5801	RNR21	107.03	109.25	118.32	100.38	-0.0017633	0.998825	Ribonucleoside-diphosphate reductase; regulated by tyrosol and cell density; ciclopirox olamine, fluconazole or flucytosine induced; regulated by Sef1, Sfu1, and Hap43
orf19.7519	orf19.7519	116.71	109.04	112.37	112.76	-0.014706605	0.998825	Ortholog of C. dubliniensis CD36 : Cd36_25180, C. parapsilosis CDC317 : CPAR2_800030, Candida tenuis NRRL Y-1498 : CANTEDRAFT_125790 and Debaryomyces hansenii CBS767 : DEHA2E18260g
orf19.6873.1	KTI11	102.71	108.84	51.22	70.65	-0.621888384	0.140986	Zn-ribbon protein; required for synthesis of diphthamide on translation factor eEF2; involved in modification of wobble nucleosides in tRNAs; rat catheter and Spider biofilm induced
orf19.2852	orf19.2852	116.54	108.84	115.23	148.03	0.174075517	0.988642	Ortholog(s) have structural constituent of ribosome activity, role in mitochondrial translational initiation and mitochondrial small ribosomal subunit localization
orf19.6753	orf19.6753	123.88	108.44	131.56	110.14	0.039660005	0.998825	Protein with a predicted RING-type zinc finger; possibly an essential gene, disruptants not obtained by UAU1 method
orf19.5814.1	orf19.5814.1	150.71	108.41	147.12	96.97	-0.080868028	0.998825	Ortholog of Debaryomyces hansenii CBS767 : DEHA2E07062g, Spathaspora passalidarum NRRL Y-27907 : SPAPADRAFT_60773 and Candida albicans WO-1 : CAWG_04278
orf19.7665	orf19.7665	129.49	108.21	125.53	117.72	0.026059809	0.998825	Ortholog(s) have role in mitochondrial respiratory chain complex IV assembly and mitochondrial inner membrane localization
orf19.3533	orf19.3533	97.05	107.99	94.6	107.69	-0.021563487	0.998825	Ortholog(s) have role in ascospore formation, mitochondrial respiratory chain complex assembly, positive regulation of transcription from RNA polymerase II promoter
orf19.1813	FLC2	106.2	107.98	101.54	89.25	-0.145705224	0.998825	Protein involved in heme uptake; putative FAD transporter, similar to S. cerevisiae Flc2p

orf19.638	FDH1	112.46	107.97	72.1	40.17	-0.75514795	0.043728	Formate dehydrogenase; oxidizes formate to CO2; Mig1 regulated; induced by macrophages; fluconazole-repressed; repressed by Efg1 in yeast, not hyphal conditions; stationary phase enriched; rat catheter and Spider biofilm induced
orf19.7387	ELP3	119.62	107.94	109.26	101.77	-0.102044136	0.998825	Predicted histone acetyltransferase; role in regulation of transcription, tRNA wobble uridine modification; Spider biofilm induced
orf19.1801	CBR1	108.35	107.67	145.01	136.83	0.311036352	0.692027	Putative cytochrome B5 reductase; plasma membrane-localized
orf19.7325	SCO1	110.13	107.58	119	112.28	0.058174703	0.998825	Putative copper transporter; Hap43p-repressed gene
orf19.4650	ILV6	110.83	107.47	153.17	158.06	0.422390837	0.36906	Putative regulatory subunit of acetolacetate synthase; alkaline induced; regulated by Gcn2 and Gcn4; protein present in exponential and stationary growth phase yeast; Spider biofilm repressed
orf19.2870	orf19.2870	89.98	107.39	46.7	63.15	-0.645213674	0.128656	Protein of unknown function; rat catheter and Spider biofilm induced
orf19.1690	TOS1	96.67	107.24	139.66	117.12	0.260865351	0.853836	Protein similar to alpha agglutinin anchor subunit; secreted; exogenously expressed protein is a substrate for Kex2 processing in vitro; fluconazole-induced; induced by alpha pheromone in SpiderM medium; Hap43-induced
orf19.346	ALT1	123.11	107.23	154.96	126.51	0.23509404	0.87796	Putative alanine transaminase; mutation confers hypersensitivity to 5-fluorocytosine (5-FC); rat catheter and flow model biofilm induced
orf19.316	SEC13	128.17	107.23	188.9	160.6	0.478138002	0.2248	Putative protein transport factor; antigenic during murine systemic infection; macrophage-downregulated protein; protein level decreases in stationary phase cultures; Spider biofilm repressed

orf19.7672	orf19.7672	106.05	107.15	93.49	87.59	-0.201617202	0.946319	Ortholog(s) have role in ER-associated ubiquitin-dependent protein catabolic process, endoplasmic reticulum unfolded protein response
orf19.6151	ARC15	122.03	107.15	136.5	118.76	0.123623537	0.998825	Putative ARP2/3 complex subunit; mutation confers hypersensitivity to cytochalasin D
orf19.5994	RHB1	127.51	106.8	160.35	146.31	0.31237362	0.673379	Putative small G protein from the Ras superfamily involved in cell wall integrity and control of filamentous growth under nitrogen starvation
orf19.5911	CMK1	105.9	106.76	108.46	116.63	0.055459143	0.998825	Putative calcium/calmodulin-dependent protein kinase II; expression regulated upon white-opaque switching; biochemically purified Ca2+/CaM-dependent kinase is soluble, cytosolic, monomeric, and serine-autophosphorylated; Hap43p-repressed
orf19.1917	orf19.1917	109.74	106.75	121.86	101.72	0.02545489	0.998825	Protein of unknown function; Spider biofilm repressed
orf19.254	orf19.254	106.02	106.71	83.08	85.4	-0.286067126	0.823496	Protein of unknown function; Hog1p-repressed; Spider biofilm induced
orf19.1710	ALI1	127.76	106.69	125.61	118.79	0.041280376	0.998825	Putative NADH-ubiquinone oxidoreductase; in detergent-resistant membrane fraction (possible lipid raft component); predicted N-terminal acetylation; nitric oxide-repressed; plasma membrane-localized; protein decreases in stationary phase
orf19.7583	ZCF39	112.88	106.42	88.31	87.19	-0.27213228	0.846842	Zn(II)2Cys6 transcription factor; mutants are viable; filament induced; required for yeast cell adherence to silicone substrate; Spider biofilm induced
orf19.4283	orf19.4283	114.1	106.39	118.83	110.38	0.03740359	0.998825	Ortholog(s) have role in translational initiation and cytosol, eukaryotic 43S preinitiation complex, eukaryotic translation initiation factor 3 complex, eIF3e, eukaryotic translation initiation factor 3 complex, eIF3m, nucleus localization

orf19.475	orf19.475	116.25	106.31	85.12	72.49	-0.412924198	0.487073	Putative rRNA processing protein; rat catheter biofilm induced
orf19.4166	ZCF21	98.65	106.26	134.28	103.03	0.156604641	0.998825	Predicted Zn(II)2Cys6 transcription factor; mutants display increased colonization of mouse kidneys; Spider biofilm induced
orf19.6176	SEC61	107.22	106.01	165.17	156.83	0.492365636	0.21299	ER protein-translocation complex subunit; essential; 10 predicted transmembrane regions; chimeric mutant partially functionally complements S. cerevisiae sec61 defects; Spider biofilm repressed
orf19.2559	CDC4	102.47	105.95	117.28	110.52	0.097443082	0.998825	F-box subunit of SCF(CDC4) ubiquitin ligase; functional homolog of S. cerevisiae Cdc4; mutation/depletion causes hyperfilamentation; Sol1 is a substrate of SCF-Cdc4; Spider biofilm induced
orf19.5282	orf19.5282	96.26	105.92	67.4	72.13	-0.434821635	0.468439	Protein of unknown function; Hap43-repressed gene; mRNA binds to She3; repressed in hyphae; Efg1 and Efh1 regulated; 5'-UTR intron; induced by Mnl1 under weak acid stress; rat catheter biofilm induced
orf19.6072.1	orf19.6072.1	91.27	105.83	61.89	80.06	-0.37557153	0.626748	Ortholog of C. parapsilosis CDC317 : CPAR2_105720, C. dubliniensis CD36 : Cd36_40990, Candida tenuis NRRL Y-1498 : CANTEDRAFT_101229 and Lodderomyces elongisporus NRLL YB-4239 : LELG_04694
orf19.6373	DCP2	108.01	105.68	110.56	100.05	-0.028139813	0.998825	Ortholog(s) have m7G(5')pppN diphosphatase activity, mRNA binding activity and role in deadenylation-dependent decapping of nuclear-transcribed mRNA, deadenylation-independent decapping of nuclear-transcribed mRNA, stress granule assembly
orf19.2283	DQD1	81.7	105.45	105.66	119.92	0.207479962	0.946319	Putative 3-dehydroquinate dehydratase; ketoconazole-repressed; protein abundance downregulated by macrophages; flow model biofilm induced
orf19.4228	orf19.4228	125.42	105.44	120.49	114.32	0.011864785	0.998825	Protein with a role in insertion of tail-anchored proteins into the ER membrane; Spider biofilm repressed

orf19.2278	orf19.2278	120.01	105.36	138.32	144.46	0.264920269	0.824505	Putative 20S proteasome assembly protein; filament induced; induced by alpha pheromone in SpiderM medium
orf19.3463	orf19.3463	123.72	105.23	91.15	85.9	-0.311086247	0.743771	Putative GTPase; role in 60S ribosomal subunit biogenesis; Spider biofilm induced
orf19.3526	ITR1	117.85	104.98	107.19	91	-0.151117112	0.998825	MFS inositol transporter; uptake of exogenous inositol; 12 transmembrane motifs; expressed in rich medium; fluconazole, caspofungin repressed; possibly essential (UAU1 method); flow model, rat catheter and Spider biofilm induced
orf19.2782	orf19.2782	121.2	104.91	104.66	82.88	-0.227148303	0.911702	Ortholog(s) have disulfide oxidoreductase activity, role in cellular response to oxidative stress, response to osmotic stress and mitochondrial matrix localization
orf19.2825	DRE2	101.56	104.87	92.48	85.45	-0.193990495	0.957711	Putative cytosolic Fe-S protein assembly protein; a-specific transcript; regulated by Sef1, Sfu1, and Hap43; rat catheter and Spider biofilm induced
orf19.2951	HOM6	97	104.8	146.93	152.93	0.465294711	0.282737	Putative homoserine dehydrogenase; Gcn4-regulated; induced by amino acid starvation (3-ATtreatment); macrophage-induced protein; protein level decreases in stationary phase cultures; flow model biofilm repressed
orf19.2414	orf19.2414	94.12	104.8	102.22	108	0.051961917	0.998825	Ortholog of S. cerevisiae Mpm1; a mitochondrial intermembrane space protein of unknown function; Hap43-repressed; Spider biofilm induced
orf19.7288	orf19.7288	117.25	104.78	175.4	150.53	0.457713392	0.271064	Protein with predicted oxidoreductase and dehydrogenase domains; Hap43-repressed; Spider biofilm induced
orf19.337	VTI1	108.26	104.69	122.93	116.13	0.126792073	0.998825	Ortholog(s) have SNAP receptor activity and role in Golgi to vacuole transport, intra-Golgi vesicle-mediated transport, vacuole fusion, non-autophagic, vesicle fusion

orf19.5771	PBP2	98.97	104.67	99.18	95.16	-0.07042021	0.998825	Putative RNA binding protein; transcript regulated by Nrg1, Mig1, and Tup1
orf19.6941	orf19.6941	119.92	104.57	161.56	145.47	0.368007634	0.522836	Putative diacylglycerol acyltransferase; catalyzes the terminal step of triacylglycerol formation; flow model biofilm induced; Spider biofilm induced
orf19.5342.2	orf19.5342.2	106.94	104.53	164.72	151.61	0.478120713	0.240375	Protein of unknown function; protein newly produced during adaptation to the serum
orf19.6129	MRPL8	116.73	104.52	110.23	118.59	0.028971054	0.998825	Mitochondrial 60S ribosomal protein subunit; Hap43p-repressed gene
orf19.7673	orf19.7673	136.79	104.43	120.88	113.34	-0.04275595	0.998825	Ortholog(s) have mRNA binding activity, role in mRNA splicing, via spliceosome and U1 snRNP, U2-type prespliceosome, U4/U6 x U5 tri-snRNP complex, U5 snRNP, commitment complex, cytosol localization
orf19.2518	orf19.2518	117.12	104.19	123.45	114.82	0.078959507	0.998825	Ortholog(s) have ATPase activator activity, soluble NSF attachment protein activity
orf19.4635	NIP1	113.83	104.17	105.05	89.04	-0.148384001	0.998825	Putative translation initiation factor; mutation confers hypersensitivity to roridin A and verrucarin A; genes encoding ribosomal subunits, translation factors, and tRNA synthetases are downregulated upon phagocytosis by murine macrophage
orf19.3330	CRL1	99.86	104.16	86.96	77.72	-0.257375333	0.870631	Predicted GTPase of RHO family; CAAX motif geranylgeranylated; expression in S. cerevisiae causes dominant-negative inhibition of pheromone response
orf19.7139	orf19.7139	125.21	104.14	122.88	111.33	0.016551503	0.998825	Ortholog(s) have role in mRNA splicing, via spliceosome

orf19.908	FEN12	119.48	104.13	90.98	89.43	-0.260218155	0.8593	Putative protein with a predicted role in the elongation of fatty acids; amphotericin B, caspofungin repressed
orf19.3535	orf19.3535	100.42	104.1	91.64	99.01	-0.086786839	0.998825	Ortholog(s) have cellular bud neck, fungal-type vacuole localization
orf19.6702	DED81	105.41	103.91	113.95	112.22	0.082403949	0.998825	Putative tRNA-Asn synthetase; genes encoding ribosomal subunits, translation factors, tRNA synthetases are downregulated upon phagocytosis by murine macrophage; protein enriched in stationary phase yeast cultures
orf19.2672	NCP1	82.98	103.86	119.25	132.83	0.337308512	0.675986	NADPH-cytochrome P450 reductase, acts with Erg11p in sterol 14 alpha-demethylation in ergosterol biosynthesis; subject to hypoxic regulation; ketoconazole-induced; caspofungin repressed
orf19.6787	orf19.6787	131.3	103.85	117.04	151.02	0.152151051	0.998825	Ortholog(s) have role in ER to Golgi vesicle-mediated transport, ascospore formation, axial cellular bud site selection and ER to Golgi transport vesicle, endoplasmic reticulum membrane localization
orf19.4883	orf19.4883	117.23	103.61	93.8	78.89	-0.296664827	0.789669	Protein of unknown function; S. cerevisiae ortholog Cos111 confers resistance to the antifungal drug ciclopirox olamine; constitutive expression independent of MTL or white-opaque status; Spider biofilm induced
orf19.4718	TRP5	117.26	103.54	112.57	95.03	-0.082657203	0.998825	Predicted tryptophan synthase; identified in detergent-resistant membrane fraction (possible lipid raft component); predicted N-terminal acetylation; Gcn4p-regulated; S. cerevisiae ortholog is Gcn4p regulated; upregulated in biofilm;
orf19.6747	orf19.6747	118.15	103.47	90.86	91.02	-0.236351811	0.892679	Ortholog(s) have acid phosphatase activity, role in dephosphorylation, intracellular sterol transport and cytoplasm, nucleus localization
orf19.4024	RIB5	93.05	103.47	135.05	149.69	0.436378402	0.377749	Putative riboflavin synthase; fungal-specific (no human or murine homolog); farnesol-downregulated; protein present in exponential and stationary growth phase yeast cultures

orf19.1477	orf19.1477	125.89	103.42	106.52	101.46	-0.122438178	0.998825	Protein of unknown function; possible ER protein; Hap43p-repressed; Spider biofilm induced
orf19.5751	ORM1	112.95	103.24	89.6	86.5	-0.242507429	0.883756	Putative endoplasmic reticulum membrane protein; Hap43p-repressed gene; mutation confers hypersensitivity to aureobasidin A
orf19.5443	BNA4	109.63	103.05	123.59	135.89	0.230385637	0.888717	Putative kynurenine 3-monooxygenase, involved in NAD biosynthesis; transposon mutation affects filamentous growth; Hap43p-repressed gene; oral infection upregulated; mutants have reduced capacity to damage oral epithelial cells
orf19.6625	orf19.6625	98.48	103.03	114.24	127	0.205201712	0.938642	Ortholog(s) have acetyltransferase activator activity, histone binding activity and role in double-strand break repair via nonhomologous end joining, nucleosome assembly, positive regulation of histone acetylation
orf19.6766	NOP13	134.9	102.91	100.47	82.53	-0.31648384	0.732796	Ortholog of S. cerevisiae Nop13; a nucleolar protein found in preribosomal complexes; Hap43-induced gene; rat catheter biofilm induced
orf19.5293	orf19.5293	103.24	102.84	160.97	167.83	0.55904609	0.113029	Ortholog of C. dubliniensis CD36 : Cd36_43710, C. parapsilosis CDC317 : CPAR2_402940, Candida tenuis NRRL Y-1498 : CANTEDRAFT_114940 and Debaryomyces hansenii CBS767 : DEHA2C09196g
orf19.5991	orf19.5991	113.91	102.79	116.83	96.03	-0.033450251	0.998825	Ortholog(s) have role in maturation of 5.8S rRNA from tricistronic rRNA transcript (SSU-rRNA, 5.8S rRNA, LSU-rRNA), maturation of LSU-rRNA from tricistronic rRNA transcript (SSU-rRNA, 5.8S rRNA, LSU-rRNA)
orf19.2640	FUR1	108.91	102.72	107.45	114.4	0.040429488	0.998825	Uracil phosphoribosyltransferase; predicted tetrameric enzyme of pyrimidine salvage; mutations associated with flucytosine resistance in clade I clinical isolates; flucytosine, macrophage-induced protein; levels decrease in stationary phase
orf19.401	TCP1	107.03	102.64	110.64	108.36	0.039546138	0.998825	Chaperonin-containing T-complex subunit, induced by alpha pheromone in SpiderM medium; stationary phase enriched protein

orf19.4176	orf19.4176	107.02	102.6	113.68	121.85	0.12982541	0.998825	Ortholog(s) have structural constituent of ribosome activity and mitochondrial small ribosomal subunit localization
orf19.7436	AAF1	108.09	102.51	128.22	88.95	0.018674905	0.998825	Possible regulatory protein; possible adhesin-like; Glu-rich domain; production in S. cerevisiae increases endothelial cell adherence and flocculence; flow model biofilm, alkaline or caspofungin induced
orf19.3930	YUH2	122.21	102.5	100.26	101.21	-0.141331017	0.998825	Putative ubiquitin C-terminal hydrolase; sumoylation target
orf19.2832	INN1	84.36	102.46	58.67	63.58	-0.469456015	0.415663	Protein with similarity to S. cerevisiae Inn1p, which is an essential protein of the contractile actomyosin ring required for ingression of the plasma membrane into the bud neck during cytokinesis; contains a C2 membrane targeting domain
orf19.4746	JIP5	114.93	102.22	96.75	107.05	-0.080354502	0.998825	Ortholog of S. cerevisiae Jip5; predicted role in biogenesis of the large ribosomal subunit; repressed in core stress response; Hap43-induced gene
orf19.5783	orf19.5783	119.96	102.21	99.8	79.62	-0.255628199	0.87024	Ortholog(s) have protein-arginine omega-N monomethyltransferase activity and role in peptidyl-arginine methylation
orf19.676	orf19.676	98.19	101.95	190.37	187.12	0.764866379	0.0069	Microtubule-binding protein of the cortical microtubule; delays exit from mitosis when the spindle is abnormally oriented; Spider biofilm repressed
orf19.5054	orf19.5054	100.86	101.87	131.33	123.95	0.261419384	0.846842	Putative quinolinate phosphoribosyl transferase, involved in NAD biosynthesis; Hap43p-repressed gene
orf19.3415	PTK2	105.01	101.87	76.59	66.59	-0.427334081	0.470755	Putative protein kinase of polyamine import; mutation confers hypersensitivity to high concentrations of tunicamycin; YPD flow model biofilm induced; rat catheter and Spider biofilm induced

orf19.1464	BMT5	106.08	101.81	75.02	69.76	-0.424863365	0.474358	Putative beta-mannosyltransferase involved in beta-1,2-mannosylation of phospholipomannan; 9-member family includes Bmt1, Bmt2, Bmt3, and Bmtp with roles in mannosylation of cell wall phosphopeptidomannan; Spider biofilm induced
orf19.3288	NMA111	104.39	101.78	90.22	87.8	-0.180649383	0.987362	Putative serine protease and general molecular chaperone; macrophage-induced gene; repressed in core stress response; merged with orf19.3288.1 in Assembly 21
orf19.7510	KIN2	98.5	101.74	126.71	119.51	0.234062202	0.885512	Protein with similarity to S. cerevisiae Kin2p, transcription is positively regulated by Tbf1
orf19.772	LYS21	99.38	101.73	112.56	111.06	0.117196187	0.998825	Homocitrate synthase; lysine biosynthesis; expression increased in a fluconazole-resistant isolate; colony morphology-related gene regulation by Ssn6; ketoconazole-repressed; regulated by Gcn2, Gcn4, Hap43
orf19.4600.1	orf19.4600.1	108.48	101.71	165.65	150.65	0.489764989	0.218547	Protein of unknown function; flow model biofilm repressed
orf19.4551	CTN1	134.96	101.61	39.39	27.28	-1.376024394	4.27E-06	Carnitine acetyl transferase; required for growth on nonfermentable carbon sources, not for hyphal growth or virulence in mice; induced in macrophage; macrophage/pseudohyphal-repressed after 16 hr; rat catheter, Spider biofilm induced
orf19.255	ZCF1	98.19	101.6	78.2	82.98	-0.259575615	0.870631	Zn(II)2Cys6 transcription factor; transcript regulated during hypha formation; 5'-UTR intron; mutants show decreased colonization of mouse kidneys; flow model biofilm induced; Spider biofilm induced
orf19.3689	orf19.3689	98.53	101.57	104.06	98.79	0.0003249	0.998853	Putative protein similar to 6-phosphofructo-2-kinase/fructose-2,6-bisphosphatase; expression downregulated in an ssr1 null mutant
orf19.1655	PXP2	101.39	101.36	198.1	175.97	0.742352125	0.009337	Putative acyl-CoA oxidase; enzyme of fatty acid beta-oxidation; induced during macrophage infection; opaque specific transcript; putative peroxisome targeting signal; Spider biofilm induced

orf19.1273	orf19.1273	100.83	101.3	91.42	97.47	-0.09330269	0.998825	Ortholog(s) have tubulin binding activity, role in tubulin complex assembly and prefoldin complex localization
orf19.967	orf19.967	112.18	101.16	108.16	105.37	-0.009366768	0.998825	Major mitochondrial nuclease; has RNAse and DNA endo- and exonucleolytic activities; roles in mitochondrial recombination, apoptosis and maintenance of polyploidy; Spider biofilm repressed
orf19.989	orf19.989	100.32	101.03	115.86	116.91	0.16487994	0.998825	Mitochondrial ribosomal protein of the small subunit; Spider biofilm repressed
orf19.1306	orf19.1306	107.4	101.03	109.72	119.89	0.110711064	0.998825	Ortholog(s) have cytosol, nucleus localization
orf19.688	orf19.688	103.68	100.97	96.29	107.2	-0.020098375	0.998825	Mitochondrial ribosomal protein of the small subunit; S. cerevisiae ortholog is essential for viability; Spider biofilm repressed
orf19.7489.3	orf19.7489.3	89.35	100.97	99.18	115.4	0.128263026	0.998825	Ortholog(s) have role in negative regulation of transcription from RNA polymerase II promoter in response to iron and cytosol, nucleus localization
orf19.2173	MAF1	86.39	100.97	67.48	72.69	-0.33619234	0.732772	Putative negative regulator of RNA polymerase III; decreased expression in hyphae vs yeast cells; caspofungin repressed; Spider biofilm repressed
orf19.2179	SIT1	105.98	100.93	122.41	118.88	0.171348822	0.992876	Transporter of ferrichrome siderophores, not ferrioxamine B; required for human epithelial cell invasion in vitro, not for mouse systemic infection; regulated by iron, Sfu1, Rfg1, Tup1, Hap43; rat catheter and Spider biofilm induced
orf19.3233	HSE1	102.66	100.88	95.19	93.16	-0.105864741	0.998825	ESCRT-0 complex subunit; SH3-domain-containing protein

orf19.288	MET13	113.36	100.76	172.9	167.8	0.560195068	0.102362	Putative methionine biosynthesis protein; ketoconazole-induced; amphotericin B repressed; Spider biofilm repressed
orf19.2655	BUB3	96.76	100.74	75.97	68.59	-0.36737336	0.621024	Protein similar to S. cerevisiae Bub3; a kinetochore checkpoint component; induced by hydroxyurea treatment; flow model biofilm induced; Spider biofilm induced
orf19.5639	HIS4	99.66	100.72	121.97	123.59	0.230263334	0.892679	Multifunctional enzyme that catalyzes three steps of histidine biosynthesis, with phosphoribosyl-AMP cyclohydrolase, phosphoribosyl-ATP diphosphatase, and histidinol dehydrogenase activities; required for wild-type adhesion to human calls
orf19.752	orf19.752	104.08	100.7	103.81	106.5	0.02441689	0.998825	Has domain(s) with predicted methyltransferase activity and role in metabolic process
orf19.1185	orf19.1185	112.74	100.68	104.33	92.81	-0.107701445	0.998825	Ortholog(s) have protein homodimerization activity, ubiquitin-protein ligase activity
orf19.5667	MNR2	112.8	100.66	106.88	98.07	-0.060723742	0.998825	Putative ion transporter; fungal-specific (no human or murine homolog)
orf19.1589.1	orf19.1589.1	108.05	100.62	159.81	179.16	0.579589403	0.092048	Has domain(s) with predicted catalytic activity, pyridoxal phosphate binding activity and role in biosynthetic process
orf19.2079	PHHB	90.61	100.57	88.6	76.83	-0.180116606	0.998825	Putative 4a-hydroxytetrahydrobiopterin dehydratase; transposon mutation affects filamentous growth; flow model biofilm induced; Spider biofilm induced
orf19.2047	orf19.2047	101.21	100.56	46.02	44.16	-0.897892127	0.007853	Putative protein of unknown function; Hap43p-repressed gene; mutation confers hypersensitivity to toxic ergosterol analog, and to amphotericin B

orf19.2057	orf19.2057	97.62	100.55	97.21	91.16	-0.077773445	0.998825	Ortholog(s) have ATP binding, ATPase activity, metallopeptidase activity, role in protein complex assembly, proteolysis, signal peptide processing and m-AAA complex, mitochondrial inner boundary membrane localization
orf19.4233	THR4	105.9	100.52	118.09	117.31	0.141188444	0.998825	Putative threonine synthase; protein present in exponential and stationary growth phase yeast cultures; Gcn4p-regulated; sumoylation target
orf19.393	APS3	110.12	100.47	120.88	110.14	0.1031142	0.998825	Component of the adaptor complex AP-3, which is involved in vacuolar protein sorting
orf19.980	orf19.980	102.96	100.41	97.42	96.54	-0.063077182	0.998825	Ortholog(s) have inositol heptakisphosphate kinase activity, inositol hexakisphosphate 1-kinase activity, inositol hexakisphosphate 3-kinase activity, inositol hexakisphosphate 4-kinase activity, inositol hexakisphosphate 6-kinase activity
orf19.2199	PHO86	118.77	100.4	98.68	95.46	-0.151221049	0.998825	Putative endoplasmic reticulum protein; possibly adherence-induced
orf19.5025	MET3	132.83	100.38	185.88	157.58	0.465560525	0.263233	ATP sulfurlyase; sulfate assimilation; repressed by Met, Cys, Sfu1, or in fluconazole-resistant isolate; Hog1, caspofungin, white phase-induced; induced on biofilm formation, even in presence of Met and Cys; Spider, F-12/CO2 biofilm induced
orf19.4430	orf19.4430	116.33	100.21	91.28	84.46	-0.255088537	0.87024	Ortholog(s) have ubiquitin binding activity, role in proteasome-mediated ubiquitin-dependent protein catabolic process and cytoplasm, nucleus localization
orf19.2111	orf19.2111	125.13	100.2	104.38	101.06	-0.117730066	0.998825	Ortholog(s) have core RNA polymerase II binding transcription factor activity and role in transcription elongation from RNA polymerase II promoter, transcriptional start site selection at RNA polymerase II promoter
orf19.6939	orf19.6939	97.57	100.16	72.66	73.28	-0.358541825	0.646416	Predicted ORF from Assembly 19; removed from Assembly 20; restored based on comparative genome analysis

orf19.5905	orf19.5905	123.25	100.06	75.38	76.7	-0.449819034	0.403142	Protein of unknown function; Hap43-induced; F-12/CO2 early biofilm induced
orf19.3074	TLO10	104.83	100.01	141.32	139.82	0.369981898	0.541043	Member of a family of telomere-proximal genes of unknown function
orf19.117	orf19.117	97.15	99.92	78.37	75.77	-0.293369207	0.823496	Ortholog(s) have SNAP receptor activity, role in exocytosis, vesicle fusion and SNARE complex, extrinsic to plasma membrane localization
orf19.2032	orf19.2032	101.39	99.91	85.93	70.72	-0.296150101	0.81271	Protein of unknown function; rat catheter biofilm induced
orf19.1633	UTP4	107.63	99.89	86.61	72.13	-0.321425882	0.740503	Putative U3 snoRNA-associated protein; Hap43-induced; physically interacts with TAP-tagged Nop1; Spider biofilm induced
orf19.145	RPB4	115.33	99.79	101.43	104.43	-0.064368274	0.998825	Protein similar to S. cerevisiae Rpb4p, which is a component of RNA polymerase II; transposon mutation affects filamentous growth
orf19.5143	TIM54	114.19	99.67	112.18	95.35	-0.049863881	0.998825	Predicted component of the mitochondrial TIM22 complex; involved in protein import into mitochondrial inner membrane; Spider biofilm repressed
orf19.1140	orf19.1140	124.89	99.63	142.35	126.95	0.205964187	0.931333	Protein of unknown function; Spider biofilm repressed
orf19.5634	FRP1	103.01	99.63	59.88	60.08	-0.602402021	0.150809	Ferric reductase; alkaline-induced by Rim101; iron-chelation-induced by CCAAT-binding factor; fluconazole-repressed; ciclopirox-, hypoxia-, Hap43-induced; colony morphology-related regulation by Ssn6; Spider and flow model biofilm induced

orf19.7214	orf19.7214	109.35	99.57	186.36	160.31	0.603889307	0.064175	Glucan 1,3-beta-glucosidase; regulated by Nrg1, Tup1 and possibly Tac1; induced by NO and during cell wall regeneration; stationary phase enriched; possibly essential (UAU1 method); F-12/CO2 early biofilm induced; flow biofilm repressed
orf19.2167	orf19.2167	128.83	99.38	108.25	94.48	-0.151021309	0.998825	Ortholog(s) have role in ribosomal large subunit biogenesis, ribosomal small subunit biogenesis and cytosolic large ribosomal subunit, nucleolus localization
orf19.4842	orf19.4842	93.28	99.18	104.29	140.95	0.276060818	0.846842	Protein of unknown function; Spider biofilm induced
orf19.6577	FLU1	81.24	99.01	32.49	34.09	-1.052587913	0.001922	Multidrug efflux pump of the plasma membrane; MDR family member of the MFS (major facilitator superfamily) of transporters; involved in histatin 5 efflux; fungal-specific (no human/murine homolog)
orf19.4898	orf19.4898	82.84	98.93	99.81	99.2	0.090654686	0.998825	Putative protein of unknown function; induced by prostaglandins
orf19.3919.1	orf19.3919.1	97.12	98.64	120.88	126.24	0.264658768	0.846842	Putative adhesin-like protein
orf19.1977	URA4	93.78	98.46	112.17	99.93	0.104065827	0.998825	Dihydroorotase; protein present in exponential and stationary growth phase yeast cultures
orf19.2185	NSA1	118.13	98.46	96.09	84.73	-0.215859063	0.931333	Putative 66S pre-ribosomal particles conmponent; Hap43-induced; repressed by prostaglandins
orf19.1609	orf19.1609	116.11	98.31	90.61	80.51	-0.263739495	0.8593	Ortholog(s) have role in endonucleolytic cleavage in ITS1 to separate SSU-rRNA from 5.8S rRNA and LSU-rRNA from tricistronic rRNA transcript (SSU-rRNA, 5.8S rRNA, LSU-rRNA) and mitotic spindle pole body, nucleolus localization

orf19.415	orf19.415	95.38	98.15	104.05	119.8	0.165763496	0.998825	Putative protein of unknown function; Hap43p-repressed gene
orf19.7092	orf19.7092	97.44	98.11	125.14	113.71	0.229781159	0.893221	Putative NAD dependent epimerase/dehydratase family protein; Spider biofilm repressed
orf19.6293	EMP24	84.01	98.08	110.64	125.93	0.297677593	0.789669	COPII-coated vesicle component
orf19.6175	orf19.6175	105.74	97.98	81.72	103.25	-0.119388945	0.998825	Putative 35S rRNA processing protein; Hap43-induced; repressed by prostaglandins; Spider biofilm induced
orf19.3319	orf19.3319	97.04	97.96	103.66	99.41	0.036121667	0.998825	Putative thioredoxin; Spider biofilm repressed
orf19.391	UPC2	90.43	97.91	130.53	128.49	0.368868774	0.565541	Zn2-Cys6 transcript factor; regulator of ergosterol biosynthetic genes and sterol uptake; binds ERG2 promoter; induced by ergosterol depletion, by azoles, anaerobicity; macrophage/pseudohyphal-repressed; flow model biofilm induced
orf19.3301	orf19.3301	89.98	97.9	72.71	63.41	-0.377654642	0.612468	Putative ubiquitin ligase complex component; induced by heavy metal (cadmium) stress; Hog1-induced; transcript induced by Mnl1p under weak acid stress; flow model biofilm induced; Spider biofilm induced
orf19.5196	ESS1	98.2	97.8	111.41	96.8	0.057834372	0.998825	Prolyl isomerase (parvulin class); essential; involved in yeast-hyphal switching, Cph1p pathway; has inflexible linker between WW and isomerase domains, unlike human homolog; functional homolog of S. cerevisiae Ess1p
orf19.4401	YVH1	100.74	97.78	69.57	72.84	-0.386422283	0.578826	Putative dual specificity phosphatase (phosphoserine/threonine and phosphotyrosine phosphatase); required for wild-type growth rate and for wild-type virulence in mouse model of systemic infection; Hap43p-induced gene

orf19.1687	orf19.1687	111.74	97.68	94.1	75.03	-0.262566622	0.863837	Ortholog of S. cerevisiae Prp43, an RNA helicase in the DEAH-box family that functions in both RNA polymerase I and polymerase II transcript metabolism; Hap43-induced gene
orf19.3944	GRR1	107.41	97.68	88.5	76.75	-0.255156376	0.873424	F-box protein component of the SCF ubiquitin-ligase complex required for cell cycle progression; involved in negative control of pseudohyphal growth; Spider biofilm induced
orf19.1678	orf19.1678	98.69	97.5	99.58	92.05	-0.035090044	0.998825	Protein of unknown function; transcript detected on high-resolution tiling arrays
orf19.2402	SSU72	105.7	97.49	80.96	79.8	-0.277044338	0.846842	Ortholog(s) have CTD phosphatase activity, protein tyrosine phosphatase activity
orf19.5464	orf19.5464	92.29	97.48	114.13	111.62	0.199372683	0.946319	Protein of unknown function
orf19.2379	NOT4	97.07	97.36	89.64	96.87	-0.05203135	0.998825	Putative E3 ubiquitin-protein ligase; required for maintenance, but not induction, of hyphal development; homozygous null mutant is avirulent in mouse systemic infection despite persistence in host; repressed in rat oral candidiasis
orf19.250	SLC1	122.28	97.31	114.02	86.33	-0.116583394	0.998825	Putative fatty acyltransferase; protein repressed during the mating process
orf19.5323	MDH1-3	119.37	97.18	164.88	136.88	0.394419586	0.463146	Predicted malate dehydrogenase; farnesol regulated; protein present in exponential and stationary growth phase yeast; Hap43p-repressed gene
orf19.2178.1	orf19.2178.1	74.18	97.14	76.07	82.28	-0.100721552	0.998825	Protein of unknown function; Spider biofilm induced

orf19.7544	TLO1	89.6	96.95	157.89	169.08	0.662654726	0.039836	Member of a family of telomere-proximal genes of unknown function; hypha-induced expression; rat catheter biofilm repressed
orf19.5326	orf19.5326	113.45	96.74	115.26	78.67	-0.104308527	0.998825	Putative transcription factor with zinc finger DNA-binding motif; possible ortholog of S. cerevisiae Mig2p
orf19.5515	orf19.5515	110.04	96.54	97.66	99.53	-0.060632567	0.998825	Ortholog(s) have ribosome binding activity, role in mitochondrial respiratory chain complex III assembly, positive regulation of mitochondrial translation and integral to mitochondrial membrane, mitochondrial ribosome localization
orf19.3349	orf19.3349	92.66	96.54	94.34	86.83	-0.067880808	0.998825	Putative RNA polymerase II subunit B150; heterozygous null mutant exhibits resistance to parnafungin in the C. albicans fitness test
orf19.6770	orf19.6770	87.25	96.49	39.37	39.94	-0.907244194	0.009513	protein with ENTH Epsin domain, N-terminal; Spider biofilm repressed
orf19.1232	VRG4	91.07	96.42	94.52	83.73	-0.062803616	0.998825	GDP-mannose transporter; essential; required for glycosylation, hyphal growth; functional homolog of S. cerevisiae Vrg4p, which imports GDP-mannose from cytoplasm to Golgi for protein and lipid mannosylation; no mammalian homolog
orf19.347	RSN1	98.67	96.39	97.29	85.34	-0.091571984	0.998825	Protein of unknown function; flow model biofilm induced; Spider biofilm induced; induced during the mating process; Hap43-repressed
orf19.3396	HCH1	94.56	96.24	87.87	99.82	-0.02712511	0.998825	Ortholog of S. cerevisiae Hch1, a regulator of heat shock protein Hsp90; regulated by Gcn4; induced in response to amino acid starvation (3-aminotriazole treatment); mutants are viable
orf19.2768	AMS1	107.54	96.22	184.38	152.5	0.598368016	0.074524	Putative alpha-mannosidase; transcript regulated by Nrg1; induced during cell wall regeneration; flow model biofilm induced; Spider biofilm induced

orf19.481	GCD1	105.96	96.1	93.43	92.74	-0.105350418	0.998825	Putative translation initiation factor; transcript regulated by Mig1; repressed upon phagocytosis by murine macrophage
orf19.2050	orf19.2050	101.84	96.08	95.29	87.24	-0.10863057	0.998825	Ortholog(s) have sterol esterase activity, role in cellular lipid metabolic process, sterol metabolic process and fungal-type vacuole, integral to membrane, lipid particle localization
orf19.3350	MRP20	105.73	96.02	103.73	108.61	0.054039529	0.998825	Component of mitochondrial ribosome; decreased expression in hyphae compared to yeast-form cells
orf19.1212	orf19.1212	89.72	95.95	149.24	146.13	0.541568512	0.158766	Ortholog(s) have FFAT motif binding, phosphatidylinositol binding activity
orf19.4262	orf19.4262	83.08	95.84	103.35	100.47	0.133380905	0.998825	Ortholog(s) have mRNA binding activity and role in cellular protein complex localization, establishment of mitochondrion localization, nuclear-transcribed mRNA catabolic process, deadenylation-dependent decay
orf19.6192	orf19.6192	84.94	95.83	98.58	95.07	0.06802251	0.998825	Protein of unknown function; Plc1-regulated; transcript detected in high-resolution tiling arrays
orf19.1065	SSA2	115.17	95.74	95.76	69.62	-0.286978976	0.833355	HSP70 family chaperone; cell wall fractions; antigenic; beta-defensin peptides impport; ATPase domain binds histatin 5; at hyphal surface, not yeast; farnesol-repressed in biofilm; flow model, Spider biofilm repressed; caspofungin repressed
orf19.5877	ATF1	119.69	95.72	85.57	72.21	-0.369759938	0.606662	Putative alcohol acetyltransferase; caspofungin repressed; expression depends on Tac1p
orf19.2438	orf19.2438	95.03	95.71	120.88	115.1	0.239389457	0.880772	Ortholog(s) have mitochondrion, ribosome localization

orf19.2256	orf19.2256	120.65	95.71	102.66	95.99	-0.109883976	0.998825	Ortholog(s) have cytosol, nucleus localization
orf19.2948	SNO1	104.14	95.71	118.85	111.68	0.160936688	0.998825	Protein with a predicted role in pyridoxine metabolism; stationary phase protein; regulated by Tup1, Efg1; Spider biofilm induced
orf19.6539	orf19.6539	100.05	95.66	111.94	100.23	0.081191991	0.998825	Putative trancription modulator; RuvB-like protein family member; heterozygous null mutant displays sensitivity to virgineone
orf19.5463	SEC6	94.2	95.59	123.94	104.22	0.201931555	0.946319	Predicted subunit of the exocyst complex, involved in exocytosis; localizes to a crescent on the surface of the hyphal tip; Hap43p-repressed gene, overlaps orf19.5464 and orf19.5465
orf19.5805	DLD1	102.8	95.57	89.02	62.2	-0.326102738	0.74873	Putative D-lactate dehydrogenase; white cell-specific trancript; colony morphology-related gene regulation by Ssn6; Hap43-repressed; rat catheter biofilm induced; Spider biofilm repressed
orf19.1756	GPD1	113.78	95.51	68.46	68.11	-0.503531717	0.294453	Glycerol-3-phosphate dehydrogenase; glycerol biosynthesis; regulated by Efg1; regulated by Tsa1, Tsa1B under H2O2 stress conditions; Sflow model and Spider biofilm induced
orf19.5393	orf19.5393	100	95.45	101.86	99.18	0.025547893	0.998825	Putative cysteine sulfinate decarboxylase; transcript positively regulated by Tbf1; Spider biofilm induced
orf19.6769	orf19.6769	101.26	95.38	68.67	68.81	-0.40835657	0.538847	Ortholog(s) have cytoplasm localization
orf19.6035	orf19.6035	106.33	95.27	96.7	99.95	-0.032038021	0.998825	Protein of unknown function; repressed by nitric oxide

orf19.23	RTA3	98.46	95.22	46.3	36.54	-0.923895181	0.0069	Similar to S. cerevisiae Rta1 (role in 7-aminocholesterol resistance) and Rsb1 (flippase); putative drug-responsive regulatory site; induced by fluphenazine, estradiol, ketoconazole, caspofungin; rat catheter biofilm induced
orf19.3065	DAO1	125.7	95.2	156.1	123.58	0.273246523	0.823496	Putative D-amino acid oxidase; transcription is regulated upon yeast-hyphal switch
orf19.2809	CTN3	90.32	95.17	125.06	116.57	0.305695951	0.748714	Peroxisomal carnitine acetyl transferase; no obvious metabolic, hyphal, virulence defects in Ura+ strain; induced by macrophage engulfment, hyphal growth, starvation, nonfermentable carbon sources; rat catheter, Spider biofilm induced
orf19.3912	GLN3	88.96	95.16	87.28	82.56	-0.102426334	0.998825	GATA transcription factor, involved in regulation of nitrogen starvation-induced filamentous growth; regulates transcription of Mep2 ammonium permease; regulated by Gcn2 and Gcn4; mRNA binds She3; Spider biofilm induced
orf19.1514	orf19.1514	111.62	95.01	116.28	100.07	0.04024499	0.998825	Ortholog(s) have enzyme binding, phosphatidylinositol-4-phosphate binding activity and role in endoplasmic reticulum unfolded protein response, protein localization to Golgi apparatus, retrograde vesicle-mediated transport, Golgi to ER
orf19.3040	EHT1	94.12	94.97	63.21	62.89	-0.465746988	0.404341	Putative acyl-coenzymeA:ethanol O-acyltransferase; regulated by Sef1, Sfu1, and Hap43; induced by alpha pheromone in SpiderM medium; Spider biofilm induced; promoter bound by Ndt80
orf19.4950	AKR1	96.95	94.87	83.88	71.56	-0.249481782	0.886087	Ankyrin-repeat protein; induced by fluphenazine
orf19.1578	orf19.1578	92.27	94.85	83.58	75.7	-0.190290804	0.976613	Ortholog of S. cereviaie Rrp5, an RNA binding protein involved in synthesis of 18S and 5.8S rRNAs; Hap43-induced gene
orf19.5828	orf19.5828	105.07	94.73	99.39	99.85	-0.014743322	0.998825	Ortholog(s) have pre-mRNA branch point binding activity, role in generation of catalytic spliceosome for first transesterification step, mRNA branch site recognition, mRNA cis splicing, via spliceosome and commitment complex localization

orf19.5161	orf19.5161	104.53	94.73	101.81	116.36	0.093903297	0.998825	Ortholog(s) have structural constituent of ribosome activity and mitochondrial large ribosomal subunit localization
orf19.5963	orf19.5963	102.02	94.63	87.62	83.82	-0.167137154	0.998825	Putative prenyltransferase; essential gene in S. cerevisiae; Spider biofilm induced
orf19.2549	SHP1	126.08	94.57	108.62	105.72	-0.039376682	0.998825	Regulator of the type 1 protein phosphatase Glc7p activity, involved in control of morphogenesis, progression through the cell cycle and response to DNA damage
orf19.5299.1	orf19.5299.1	62.38	94.43	37.33	35.53	-0.780228342	0.059118	Protein of unknown function
orf19.1045	orf19.1045	96.75	94.41	81.47	100.86	-0.062519573	0.998825	Putative U2 snRNP protein; Hap43p-induced gene; mutation confers hypersensitivity to 5-fluorocytosine (5-FC), 5-fluorouracil (5-FU), and tubercidin (7-deazaadenosine)
orf19.5227	orf19.5227	94.44	94.41	81.24	90.22	-0.117834753	0.998825	Chaperone component; involved in assembly of alpha subunits into the 20S proteasome; flow model biofilm induced
orf19.5181	NIK1	102.91	94.32	118.84	109.41	0.162796283	0.998825	Histidine kinase involved in a two-component signaling pathway that regulates cell wall biosynthesis; required for wild-type virulence in mouse systemic infection but not for wild-type growth or drug sensitivity/resistance; 9 HAMP domains
orf19.2511.1	MRPL33	106.94	94.08	91.01	95.94	-0.092084478	0.998825	Putative mitochondrial ribosomal protein of the large subunit; Ssr1-repressed; rat catheter biofilm induced
orf19.7428	APN1	93.91	94.04	112.66	112.95	0.206670123	0.938642	Ortholog of S. cerevisiae Apn1; an AP endonuclease; transcript induced by interaction with macrophages; fungal-specific (no human or murine homolog)

orf19.4887	ECM21	76.22	93.91	79.72	82.7	-0.059553246	0.998825	Predicted regulator of endcytosis of plasma membrane proteins; fluconazole induced, alkaline induced by Rim101; repressed by caspofungin and in azole-resistant strain overexpressing MDR1; flow model, rat catheter and Spider biofilm induced
orf19.6034	TUB2	95.47	93.91	116.19	109.78	0.200099649	0.946319	Beta-tubulin; functional homolog of ScTub2; overproduction makes S. cerevisiae inviable; has two introns; GlcNAc, hypha fluconazole-induced; slow growth, ectopic expression increases white-to opaque switch; rat catheter biofilm repressed
orf19.558	GUT1	129.61	93.9	124.68	87.2	-0.074087815	0.998825	Putative glycerol kinase; downregulated upon adherence to polystyrene; greater mRNA abundance observed in a cyr1 homozygous null mutant than in wild type
orf19.1691	orf19.1691	70.15	93.83	248.65	318.46	1.482828973	8.07E-10	Plasma-membrane-localized protein; filament induced; Hog1, ketoconazole, fluconazole and hypoxia-induced; regulated by Nrg1, Tup1, Upc2; induced by prostaglandins; flow model biofilm induced; rat catheter and Spider biofilm repressed
orf19.846	orf19.846	87.78	93.8	88.25	78.87	-0.110726004	0.998825	Predicted protein kinase similar to S. cerevisiae Nnk1; implicated in proteasome function in S. cerevisiae; induced by Mnl1 under weak acid stress
orf19.2545	DOT6	94.76	93.8	92.56	82.77	-0.09378009	0.998825	Protein with a predicted role in telomeric gene silencing and filamentation; repressed by high-level peroxide stress; Spider biofilm induced
orf19.5917	STP1	106.66	93.65	87.32	67.23	-0.315891408	0.769575	Transcription factor; regulates SAP2, OPT1 expression and thereby protein catabolism for nitrogen source; activated via amino-acid-induced proteolytic processing; macrophage/pseudohyphal-repressed; Spider biofilm repressed
orf19.2132	orf19.2132	92.27	93.6	105.36	105.04	0.131529206	0.998825	Ortholog of C. dubliniensis CD36 : Cd36_65220, C. parapsilosis CDC317 : CPAR2_213740, Candida tenuis NRRL Y-1498 : CANTEDRAFT_92162 and Debaryomyces hansenii CBS767 : DEHA2A01056g
orf19.1789.1	LYS1	118.26	93.56	110.29	95.74	-0.042704808	0.998825	Saccharopine dehydrogenase (biosynthetic); enzyme of alpha-aminoadipate lysine biosynthesis pathway; functionally complements S. cerevisiae lys1 mutation; fungal-specific (no human or murine homolog)

orf19.3412	ATG15	97.62	93.45	78.18	78.04	-0.241517289	0.894293	Putative lipase; fungal-specific (no human or murine homolog); Hap43p-repressed gene
orf19.3938	orf19.3938	105.27	93.4	100.65	112.39	0.078289579	0.998825	Putative mitochondrial ribosomal protein of the small subunit; mutation confers hypersensitivity to 5-fluorocytosine (5-FC), 5-fluorouracil (5-FU), and tubercidin (7-deazaadenosine)
orf19.568	SPE2	91.96	93.4	114.85	124.07	0.292542642	0.789669	Putative S-adenosylmethionine decarboxylase; Hap43-induced gene; possibly adherence-induced; Spider biofilm induced
orf19.3609	UTP15	103.26	93.37	78.63	77.27	-0.271400875	0.858075	Small subunit (SSU) processome component; mutation confers resistance to 5-fluorocytosine (5-FC); physically interacts with TAP-tagged Nop1p
orf19.3400	COQ3	90.13	93.36	118.01	106.48	0.225455972	0.914829	Protein with a predicted role in coenzyme Q biosynthesis; transcriptionally induced by interaction with macrophages; possibly an essential gene, disruptants not obtained by UAU1 method
orf19.1653	orf19.1653	96.31	93.32	73.51	97.31	-0.120282425	0.998825	Has domain(s) with predicted integral to membrane localization
orf19.2019	orf19.2019	92.7	93.27	109.81	104.97	0.159220666	0.998825	Ortholog(s) have structural constituent of ribosome activity and mitochondrial large ribosomal subunit localization
orf19.4135	PRC2	99.67	93.17	80.92	70.08	-0.291444829	0.824505	Putative carboxypeptidase; induced by human neutrophils; Spider biofilm induced
orf19.871	orf19.871	88.02	93.09	88.8	88.22	-0.036699466	0.998825	Ortholog(s) have protein transporter activity, role in Golgi to plasma membrane transport, intracellular protein transport and vesicle coat localization

orf19.1738.1	orf19.1738.1	87.29	93.03	100.73	140.23	0.329395721	0.723433	Protein involved in microtubule morphogenesis; required for protection from excess free beta-tubulin; possibly involved the beta-tubulin folding; Spider biofilm repressed
orf19.6116	GLK4	81.49	93.01	156.13	161.04	0.705252028	0.026072	Putative glucokinase; decreased expression in hyphae compared to yeast-form cells
orf19.1697	orf19.1697	102.42	92.99	89.77	79.13	-0.175829673	0.998825	Ortholog(s) have role in cytoplasmic translation and cytoplasm, polysomal ribosome localization
orf19.6665	orf19.6665	79.77	92.85	95.57	82.45	0.018688515	0.998825	Ortholog(s) have Ran GTPase binding, nucleocytoplasmic transporter activity
orf19.6084	orf19.6084	100.4	92.83	83.29	61.91	-0.336178099	0.72424	Protein of unknown function; flow model biofilm induced; Spider biofilm induced
orf19.5569	orf19.5569	91.4	92.81	85.83	90.58	-0.054444645	0.998825	Ortholog(s) have role in maintenance of rDNA, mitotic sister chromatid segregation and nuclear envelope localization
orf19.7195	RAD6	120.84	92.67	104.69	102.12	-0.046257373	0.998825	Functional ortholog of S. cerevisiae Rad6; negative regulator of hyphal growth; required for wild-type UV resistance; acts in pathway with Efg1; ; transcript is UV-induced, slightly repressed during hyphal growth; gene has two introns
orf19.5085	orf19.5085	87.37	92.66	85.45	86.36	-0.069624999	0.998825	Ortholog(s) have nuclear localization sequence binding, protein transporter activity and role in mRNA export from nucleus, protein import into nucleus, regulation of mitosis, regulation of protein desumoylation
orf19.7599	UTP5	102.49	92.66	77.84	82.06	-0.235777277	0.90951	Putative U3 snoRNA-associated protein; Hap43p-induced gene; mutation confers resistance to 5-fluorocytosine (5-FC), 5-fluorouracil (5-FU), and tubercidin (7-deazaadenosine); physically interacts with TAP-tagged Nop1p

orf19.7368	orf19.7368	77.24	92.63	104.53	102.05	0.214146715	0.938642	Ortholog(s) have mRNA binding, poly(U) RNA binding activity and role in nuclear-transcribed mRNA catabolic process, nonsense-mediated decay, regulation of mRNA stability, stress granule assembly
orf19.6319	orf19.6319	84.2	92.6	83.51	75.72	-0.12729175	0.998825	Ortholog(s) have mRNA binding, ubiquitin-specific protease activity and role in protein deubiquitination, regulation of ER to Golgi vesicle-mediated transport, regulation of response to osmotic stress, ribophagy
orf19.939	NAM7	89.04	92.55	93.6	97.86	0.051730054	0.998825	Putative role in nonsense-mediated mRNA decay; similar to S. cerevisiae Nam7p; gene induced by ciclopirox olamine treatment
orf19.4112	orf19.4112	92.76	92.52	96.83	101.5	0.063081361	0.998825	Ortholog(s) have 8-oxo-dGDP phosphatase activity, ATP binding, magnesium ion binding, thiamine binding, thiamine diphosphokinase activity, role in thiamine biosynthetic process and cytosol localization
orf19.3136	orf19.3136	96.53	92.43	117.77	93.11	0.116143558	0.998825	Ortholog(s) have transcription coactivator activity, role in DNA repair, cellular carbohydrate metabolic process and cytoplasm localization
orf19.6947	GTT11	88.16	92.37	112.1	101.3	0.18461278	0.976613	Glutathione S-transferase, localized to ER; induced in exponentially growing cells, under oxidative stress; induced by nitric oxide; Spider biofilm induced
orf19.4475	KTR4	113.06	92.35	122.94	96.09	0.066916223	0.998825	Mannosyltransferase; induced during cell wall regeneration; fungal-specific (no human or murine homolog); Bcr1-repressed in RPMI a/a biofilms
orf19.3540	MAK5	112.25	92.28	86.49	78.51	-0.253672307	0.878136	Putative nucleolar DEAD-box RNA helicase; oxidative stress-repressed via Cap1; repressed by prostaglandins
orf19.2831	RPC31	87.85	92.18	71.96	77.79	-0.216155691	0.943903	Putative RNA polymerase III subunit C31; repressed by nitric oxide; induced during infection of murine kidney, compared to growth in vitro; has murine homolog

orf19.7047	RTF1	97.92	92.12	86.81	97.83	-0.038805219	0.998825	Putative RNA polymerase II-associated Paf1 complex subunit; induced during the mating process
orf19.782	orf19.782	108.84	92.1	87.08	74.52	-0.258130278	0.872276	Ortholog(s) have serine hydrolase activity, triglyceride lipase activity, role in lipid homeostasis and lipid particle localization
orf19.6944	PHB1	113.36	92.06	159.99	140.2	0.449469579	0.316181	Putative prohibitin; identified in detergent-resistant membrane fraction (possible lipid raft component); predicted N-terminal acetylation; Hap43p-repressed gene
orf19.364	orf19.364	98.18	92.04	132.18	99.67	0.221466735	0.921805	Putative vacuolar H+-ATPase subunit; possibly an essential gene, disruptants not obtained by UAU1 method; Spider biofilm repressed
orf19.3957	orf19.3957	93.53	91.9	117.23	114.89	0.250671424	0.87024	Ortholog(s) have GTP cyclohydrolase I activity, role in folic acid-containing compound biosynthetic process and cytosol, nucleus localization
orf19.6734	TCC1	103.69	91.81	93.32	71.72	-0.209039017	0.946319	Putative transcription factor/corepressor; regulation of filamentation and virulence; interacts with Tup1; regulates hypha-specific gene expression; contains 4 tetratricopeptide repeat (TPR) motifs; flucytosine repressed; Tbp1-induced
orf19.4777	DAK2	97.02	91.8	87.66	90.1	-0.078639658	0.998825	Putative dihydroxyacetone kinase; repressed by yeast-hypha switch; fluconazole-induced; caspofungin repressed; protein enriched in stationary phase yeast cultures; flow model biofilm induced; rat catheter and Spider biofilm repressed
orf19.4258	orf19.4258	96.58	91.54	108.6	96.09	0.084000445	0.998825	Puttaive pre-mRNA polyadenylation factor; heterozygous null mutant exhibits hypersensitivity to parnafungin and cordycepin in the C. albicans fitness test
orf19.2287	RPA12	102.81	91.41	89.58	74.97	-0.19689486	0.957711	Putative DNA-directed RNA polymerase I; induced upon adherence to polystyrene

orf19.4828	orf19.4828	96.26	91.23	90.05	92.31	-0.040634113	0.998825	WD repeat domain protein; Hap43-repressed gene; flow model biofilm induced
orf19.6726	orf19.6726	99.68	91.15	97.53	96.73	0.015046661	0.998825	Ortholog(s) have DNA secondary structure binding activity
orf19.1889	orf19.1889	78.6	91	112.43	125.83	0.381098764	0.568429	Putative phosphoglycerate mutase family protein; stationary phase enriched protein; early-stage flow model biofilm induced; Spider biofilm repressed
orf19.6232	NPR1	105.43	90.94	104.63	92.45	-0.004032631	0.998825	Predicted serine/threonine protein kinase, involved in regulation of ammonium transport; induced in core stress response; Hap43p-repressed gene
orf19.2098	ARO8	88.63	90.87	136.99	111.5	0.364445509	0.586088	Aromatic transaminase of the Ehrlich fusel oil pathway of aromatic alcohol biosynthesis; Rim101 independent alkaline induction; protein abundance affected by URA3 expression in CAI-4 strain; Gcn4-regulated; stationary phase enriched
orf19.5095	orf19.5095	94.5	90.85	122.91	119.75	0.306695047	0.742982	Putative oxysterol-binding protein; caspofungin induced; possibly an essential gene, disruptants not obtained by UAU1 method
orf19.96	TOP1	125.62	90.75	85.51	59.78	-0.456337901	0.422853	DNA topoisomerase I; required for wild-type growth and for wild-type mouse virulence; sensitive to camptothecin; induced upon adherence to polystyrene; rat catheter biofilm induced
orf19.1626	orf19.1626	93.87	90.7	81.79	75.18	-0.198438747	0.957711	Deoxyhypusine synthase; catalyzes formation of deoxyhypusine, the first step in hypusine biosynthesis; Spider biofilm repressed
orf19.7551	ALO1	98.12	90.65	73.39	66.94	-0.350709087	0.680517	D-Arabinono-1,4-lactone oxidase involved in biosynthesis of dehydro-D-arabinono-1,4-lactone, which has a protective role against oxidative damage; plasma membrane-localized;required for full virulence in a mouse model of systemic infection

orf19.6139	FRE7	86.57	90.56	114.4	92	0.156124938	0.998825	Copper-regulated cupric reductase; repressed by ciclopirox olamine or 17-beta-estradiol; induced by alkaline conditions or interaction with macrophage; Spider biofilm induced
orf19.1886	RCL1	92.59	90.53	79.07	65.11	-0.289401832	0.842252	Putative U3-containing 90S preribosome processome complex subunit; Hap43-induced; essential; S. cerevisiae ortholog is essential; represses in core stress response;
orf19.1439	IPK1	101.12	90.52	82.02	76.46	-0.233440803	0.914829	Ortholog of S. cerevisiae/S. pombe Ipk1; an inositol pentakisphosphate 2-kinase, a nuclear protein required for synthesis of 1,2,3,4,5,6-hexakisphosphate; Spider biofilm induced
orf19.1545	orf19.1545	102.26	90.45	104.93	101.1	0.073396735	0.998825	Ortholog(s) have structural constituent of ribosome activity and mitochondrial small ribosomal subunit localization
orf19.7644	orf19.7644	101.33	90.37	109.48	104.48	0.118756086	0.998825	Ortholog(s) have ubiquitin-protein ligase activity, role in anaphase-promoting complex-dependent proteasomal ubiquitin-dependent protein catabolic process, protein ubiquitination and anaphase-promoting complex localization
orf19.7531	orf19.7531	87.29	90.31	97.31	103.36	0.131776785	0.998825	Protein of unknown function; stationary phase enriched protein; induced upon yeast-hypha transition; benomyl or caspofungin induced; Hap43-repressed; Spider biofilm induced
orf19.4399	NCS2	100.04	90.25	61.26	57.67	-0.532886851	0.265944	Putative cytosolic thiouridylase subunit; Spider biofilm induced
orf19.1355	orf19.1355	93.61	89.97	111.88	112.37	0.221710814	0.918842	Putative protein of unknown function; stationary phase enriched protein
orf19.6940	orf19.6940	89.78	89.75	77.9	74.28	-0.203012402	0.956789	Protein of unknown function

orf19.2614	RSR1	99.21	89.72	126.74	133.82	0.375728471	0.543746	RAS-related protein; GTP/GDP cycling required for wild-type polar bud site selection, hyphal growth guidance; role in systemic virulence in mouse; geranylgeranylation predicted; suppresses S. cerevisiae cdc24-4 mutant heat sensitivity
orf19.5379	ERG4	72.64	89.68	132.09	161.44	0.674234029	0.055379	Protein similar to sterol C-24 reductase; shows Mob2p-dependent hyphal regulation; fluconazole-induced; caspofungin repressed; rat catheter biofilm repressed
orf19.5436	UTP8	98.82	89.68	88.58	81.93	-0.125407475	0.998825	Essential nucleolar protein; involved in tRNA export from the nucleus and ribosomal small subunit biogenesis; physically interacts with TAP-tagged Nop1; Spider biofilm induced
orf19.5175	orf19.5175	89.34	89.42	92.64	87.35	0.001961935	0.998825	Ortholog(s) have ubiquitin-protein ligase activity, role in ER-associated ubiquitin-dependent protein catabolic process and Doa10p ubiquitin ligase complex, integral to endoplasmic reticulum membrane, nuclear inner membrane localization
orf19.5674	PGA10	61.01	89.34	92.66	142.46	0.483199863	0.403142	GPI anchored membrane protein; utilization of hemin and hemoglobin for Fe in host; Rim101 at ph8/hypoxia/ketoconazole/ciclopirox/hypha-induced; required for RPMI biofilm formation, Bcr1-induced in a/a biofilm; rat catheter biofilm repressed
orf19.58	RRP6	97.12	89.27	77.28	71.09	-0.270138766	0.861217	Putative nuclear exosome exonuclease component; Hap43p-induced gene; mutation confers hypersensitivity to 5-fluorocytosine (5-FC), 5-fluorouracil (5-FU), and tubercidin (7-deazaadenosine)
orf19.4241	orf19.4241	79.51	89.16	91.01	81.2	0.007311222	0.998825	Protein of unknown function; transcript detected on high-resolution tiling arrays
orf19.6783	orf19.6783	90.49	88.98	84.1	86.44	-0.068876635	0.998825	Putative geranylgeranyltransferase regulatory component
orf19.3367	orf19.3367	89.6	88.92	80.51	78.32	-0.145640538	0.998825	Ortholog(s) have structural constituent of ribosome activity and mitochondrial large ribosomal subunit localization

orf19.6314	RPB8	108.37	88.88	116.47	111.56	0.16439687	0.998825	Putative subunit of RNA polymerases I, II, and III; regulated by Gcn4p; repressed in response to amino acid starvation (3-aminotriazole treatment); heterozygous null mutant exhibits resistance to parnafungin
orf19.6730	orf19.6730	103.76	88.65	76.95	67.78	-0.335011167	0.720423	Ortholog(s) have nucleolus localization
orf19.7250	orf19.7250	99.37	88.64	75.99	80.4	-0.221439374	0.933046	Protein of unknown function; rat catheter and Spider biofilm induced
orf19.6173	STD1	95.39	88.63	96.81	99.94	0.070465343	0.998825	Putative transcription factor; involved in control of glucose-regulated gene expression; repressed by Rgt1; Spider biofilm induced
orf19.997	SNL1	89.61	88.61	111.88	125.96	0.325876354	0.701828	Ribosome-associated protein predicted to function in protein synthesis; 1 predicted transmembrane domain; rat catheter biofilm repressed
orf19.7175	HLJ1	87.3	88.6	87.45	89.48	-0.010124183	0.998825	Putative HSP40 co-chaperone; repressed during the mating process
orf19.522	PIM1	106.49	88.57	88.7	91.38	-0.100428411	0.998825	ATP-dependent Lon protease; role in degradation of misfolded proteins in mitochondria, biogenesis and maintenance of mitochondria; rat catheter biofilm induced
orf19.1783	YOR1	117.84	88.47	34.09	19.02	-1.388561995	1.04E-05	Protein similar to S. cerevisiae Yor1; ABC-type plasma membrane transporter involved in resistance to aureobasidin A; white cell type-specific transcript; Spider biofilm induced
orf19.5671	orf19.5671	87.04	88.45	80.84	74.53	-0.141739977	0.998825	Ortholog(s) have role in actin cortical patch assembly, septin cytoskeleton organization and cellular bud neck, cellular bud tip, mating projection base, mating projection tip localization

orf19.423	orf19.423	102.05	88.43	84.41	88.88	-0.115611986	0.998825	Ortholog(s) have enzyme activator activity, m7G(5')pppN diphosphatase activity, mRNA binding activity, role in deadenylation-dependent decapping of nuclear-transcribed mRNA and cytoplasmic mRNA processing body localization
orf19.4772	SSU81	77.04	88.29	63.17	65.87	-0.282885956	0.8593	Predicted adaptor protein involved in activation of MAP kinase-dependent signaling pathways; links response to oxidative stress to morphogenesis and cell wall biosynthesis; caspofungin repressed
orf19.372	orf19.372	68.91	88.26	70.56	66.54	-0.155218748	0.998825	Putative protein of unknown function; Hap43p-repressed gene
orf19.5812	orf19.5812	86.91	88.21	125.74	132.46	0.449266049	0.359369	Ortholog of S. cerevisiae Ett1, a nuclear protein that inhibits replication of Brome mosaic virus; early-stage flow model biofilm induced
orf19.5416	ESA1	88.5	88.13	76.86	71.24	-0.21359575	0.946319	Subunit of the NuA4 histone acetyltransferase complex; member of MYST family; Spider biofilm induced
orf19.7020	orf19.7020	92.52	88.02	59.48	52.19	-0.55142683	0.245269	Protein similar to S. cerevisiae Kex1p, which is a pheromone-processing peptidase; possible Kex2p substrate
orf19.4146	SMD3	98.24	88	101.16	110.01	0.13958773	0.998825	Putative core snRNP protein; induced upon adherence to polystyrene
orf19.2124	orf19.2124	94.85	87.97	105.64	99.46	0.122565565	0.998825	Predicted alcohol dehydrogenase; Spider biofilm induced
orf19.5605	orf19.5605	78.07	87.89	61.36	59.86	-0.359487595	0.692027	Ortholog(s) have protein binding, bridging, ubiquitin protein ligase binding activity and role in positive regulation of ubiquitin-dependent endocytosis, regulation of intracellular transport

orf19.4265	UAP1	88.16	87.87	73.04	77.7	-0.182767536	0.998825	UDP-N-acetylglucosamine pyrophosphorylase, catalyzes biosynthesis of UDP-N-acetylglucosamine from UTP and N-acetylglucosamine 1-phosphate; functional homolog of S. cerevisiae Qri1p; alkaline upregulated
orf19.3972	orf19.3972	93.96	87.82	89.49	100.21	0.035701627	0.998825	Ortholog(s) have role in ER to Golgi vesicle-mediated transport, Golgi to endosome transport and ER to Golgi transport vesicle, Golgi membrane, endoplasmic reticulum localization
orf19.124	CIC1	88.51	87.79	70.34	69.62	-0.274253373	0.861125	Putative proteasome-interacting protein; rat catheter biofilm induced
orf19.1680	TFP1	98.7	87.77	131.35	115.34	0.314517144	0.720423	Subunit of vacuolar H+-ATPase; stationary phase enriched protein; sumoylation target; Spider biofilm repressed
orf19.891	orf19.891	97.03	87.75	118.28	108.93	0.231068265	0.894293	Putative AMP deaminase; possibly an essential gene, disruptants not obtained by UAU1 method
orf19.2690	orf19.2690	97.54	87.66	87.79	78.48	-0.140847821	0.998825	Putative mitochondrial GTPase; required for mitochondrial morphology and genome maintenance; Spider biofilm induced
orf19.484	MRPL40	100.26	87.63	101.29	98.83	0.063422421	0.998825	Putative mitochondrial ribosomal protein; Spider biofilm repressed
orf19.2397.3	orf19.2397.3	79.88	87.63	72.64	77.59	-0.13033532	0.998825	Putative aminotransferase; Hap43-repressed; homozygous Tn insertion decreases colony wrinkling in filament-inducing conditions, does not block true hypha formation in liquid media; rat catheter and Spider biofilm induced
orf19.5522	orf19.5522	91.99	87.6	108.27	107.35	0.198286032	0.951606	Ortholog of C. dubliniensis CD36 : Cd36_62760, C. parapsilosis CDC317 : CPAR2_601700, Candida tenuis NRRL Y-1498 : CANTEDRAFT_115220 and Debaryomyces hansenii CBS767 : DEHA2A06666g

orf19.7077	orf19.7077	95.8	87.34	76.63	79	-0.190706436	0.976704	Putative ferric reductase; induced by Mac1 under copper starvation; Plc1-regulated; Rim101-repressed
orf19.6091	RIM8	71.31	87.25	56.28	50.72	-0.432391736	0.543746	Beta-arrestin-like protein; involved in pH response; required for pathogenesis, activation of Rim101 and alkaline pH-induced hyphal growth; colony morphology-related gene regulation by Ssn6p negative feedback regulation target
orf19.744	GDB1	88.74	87.07	117.77	125.6	0.375695736	0.561982	Putative glycogen debranching enzyme; expression is regulated upon white-opaque switch; regulated by Nrg1, Tup1; rat catheter biofilm repressed
orf19.5689	orf19.5689	86.26	87.03	118.66	116.39	0.34924793	0.626748	Epsilon-COP subunit of the coatomer; regulates retrograde Golgi-to-ER protein traffic; Spider biofilm repressed
orf19.7652	CKA1	93.15	86.94	90.47	73.09	-0.124832073	0.998825	Putative alpha subunit (catalytic subunit) of protein kinase CK2; Cka1p and Cka2p have a common target with respect to fluconazole resistance; synthetically lethal with CKA2; flucytosine induced
orf19.7215	orf19.7215	86.76	86.9	80.21	78.13	-0.120095292	0.998825	Nucleolar protein; component of the small subunit processome containing the U3 snoRNA; involved in pre-18S rRNA processing; flow model biofilm repressed
orf19.1331	HSM3	92.41	86.86	76.04	67.48	-0.264617726	0.872276	Ortholog(s) have role in mismatch repair, proteasome regulatory particle assembly and cytosol, nucleus, proteasome regulatory particle, base subcomplex localization
orf19.3955	MES1	96.78	86.72	100.46	103.11	0.106164705	0.998825	Cytoplasmic methionyl-tRNA synthetase; zinc-binding motif; ribosomal subunits, translation factors, tRNA synthetases are downregulated upon phagocytosis by murine macrophage; protein present in exponential and stationary phase yeast
orf19.3130	orf19.3130	103.62	86.71	72.2	68.48	-0.360595079	0.649978	Ortholog of S. cerevisiae Gpn3 a GTPase with a role in biogenesis of RNA pol II and polIII; possibly an essential gene, disruptants not obtained by UAU1 method

orf19.2829	orf19.2829	124.86	86.69	84.41	94.78	-0.19805896	0.957711	Ortholog(s) have role in protein transport and endoplasmic reticulum, nuclear envelope localization
orf19.2277	TPK2	93.63	86.6	126.88	125.42	0.38291352	0.536918	cAMP-dependent protein kinase catalytic subunit; isoform of Tpk1; needed for epithelial cell damage, engulfment and oral (not systemic) virulence in mice; control of morphogenesis and stress response; rat catheter, Spider biofilm repressed
orf19.1108	HAM1	82.18	86.59	86.07	102.16	0.1129366	0.998825	Putative deoxyribonucleoside triphosphate pyrophosphohydrolase; caspofungin repressed; regulated by Gcn2p and Gcn4p
orf19.1799	GAP5	115.71	86.53	94.39	91.66	-0.108082981	0.998825	Putative general amino acid permease; fungal-specific (no human or murine homolog)
orf19.3663	PHO91	75.73	86.48	70.48	79.83	-0.095715356	0.998825	Putative low-affinity phosphate transporter; fungal-specific (no human or murine homolog)
orf19.5663	RCH1	120.6	86.48	39.89	22.8	-1.235203747	0.000132	Plasma membrane protein; involved in regulation of cytosolic calcium homeostasis; null mutation confers sensitivity to calcium and resistance to azoles and terbinafine; rat catheter biofilm induced
orf19.6136	orf19.6136	95.78	86.4	101	90.21	0.04585752	0.998825	Ortholog of S. cerevisiae Mrpl4, a mitochondrial ribosomal protein of the large subunit; repressed by nitric oxide
orf19.6058	GLO1	95.75	86.39	125.44	144.36	0.455609888	0.341413	Putative monomeric glyoxalase I; oxidative stress-induced via Cap1; flow model and rat catheter biofilm repressed
orf19.4279	MNN1	82.03	86.22	56.23	48.77	-0.523782177	0.312123	Putative alpha-1,3-mannosyltransferase; of the mannosyltransferase complex; negatively regulated by Rim101; transcript elevated in chk1 and nik1 mutants, but not in sln1 mutant; Spider and flow model biofilm induced

orf19.2333	orf19.2333	96.71	86.19	86.61	78.22	-0.128564842	0.998825	Ortholog(s) have role in retrograde transport, endosome to Golgi and cytosol, late endosome, nucleus localization
orf19.7025	MCM1	86.92	86.16	110.76	108.06	0.265259315	0.858075	Transcription factor; regulator of hyphal growth; may act with Wor1p; canonical and non-canonical binding sites; MADS domain DNA-binding motif; similar to S. cerevisiae Mcm1p; greater expression in white than opaque cells; intron in 5'-UTR
orf19.3084	orf19.3084	90.47	86.14	67.94	72.4	-0.266402605	0.872276	Ortholog(s) have histone acetyltransferase activity, role in chromatin silencing at telomere and SAS acetyltransferase complex, nuclear chromatin localization
orf19.4594	CLC1	77.88	86.06	116.64	109.53	0.366387514	0.600579	Clathrin light chain; subunit of the major coat protein involved in intracellular protein transport and endocytosis; rat cathetr and Spider biofilm repressed
orf19.4217	orf19.4217	102.45	86.02	90.49	67.42	-0.215628377	0.943903	Ortholog of Candida tropicalis MYA-3404 : CTRG_03197, Candida lusitaniae ATCC 42720 : CLUG_02837 and Candida albicans WO-1 : CAWG_04597
orf19.7111	orf19.7111	98.32	86	116.23	119.13	0.280229582	0.823496	Putative mitochondrial outer membrane protein membrane fission effector; possibly an essential gene, disruptants not obtained by UAU1 method
orf19.5648	orf19.5648	86.94	85.95	93.03	110.06	0.176523273	0.998825	Ortholog of C. dubliniensis CD36 : Cd36_40300, C. parapsilosis CDC317 : CPAR2_402190, Candida tenuis NRRL Y-1498 : cten_CGOB_00197 and Debaryomyces hansenii CBS767 : DEHA2F06160g
orf19.6225.1	orf19.6225.1	70.47	85.94	107.52	108.88	0.366417342	0.616255	Ortholog(s) have role in mitochondrial respiratory chain complex IV assembly and mitochondrion localization
orf19.2319	orf19.2319	104.43	85.78	80.55	75.21	-0.233926674	0.916335	Putative nucleolar protein with a predicted role in pre-rRNA processing; Hap43-induced gene; repressed in core stress response

orf19.2201	orf19.2201	96	85.77	76.6	97.49	-0.057075718	0.998825	Ortholog(s) have ribosome binding activity, role in mitochondrial respiratory chain complex III assembly, positive regulation of mitochondrial translation and mitochondrial ribosome localization
orf19.2337	ALP1	108.22	85.74	102.13	82.32	-0.070852999	0.998825	Cystine transporter; present in pathogenic yeasts (no human or murine homolog); Spider biofilm induced
orf19.6438	orf19.6438	79.33	85.72	86.25	85.68	0.036207186	0.998825	Ortholog(s) have serine C-palmitoyltransferase activity, role in sphingolipid biosynthetic process and SPOTS complex localization
orf19.5322	orf19.5322	87.51	85.72	92.79	90.49	0.047917967	0.998825	Ortholog(s) have phosphatidylinositol-3-phosphate binding activity and role in CVT pathway, autophagic vacuole assembly, peroxisome degradation, protein localization to pre-autophagosomal structure, vesicle organization
orf19.173	orf19.173	66	85.61	64.21	66.18	-0.182437069	0.998825	C2H2 transcription factor; induced by Mnl1 under weak acid stress
orf19.2289	ARP3	85.41	85.52	115.84	113.27	0.331548176	0.690331	Protein with Myo5p-dependent localization to cortical actin patches at hyphal tip; mutation confers hypersensitivity to cytochalasin D; Spider biofilm repressed
orf19.1966	BUD23	105.59	85.51	88.57	74.58	-0.190005453	0.976613	Putative methyltransferase; Hap43-induced; repressed by prostaglandins
orf19.1058	orf19.1058	104.34	85.4	113.83	94.02	0.101722254	0.998825	Ortholog(s) have ubiquitin binding activity, role in ubiquitin-dependent protein catabolic process and proteasome regulatory particle, lid subcomplex, proteasome storage granule localization
orf19.5908	TEC1	69.29	85.32	107.42	93.67	0.289178191	0.839221	TEA/ATTS transcription factor; white cell pheromone response, hyphal gene regulation; required for Spider and RPMI biofilm formation; regulates BCR1; Cph2 regulated transcript; alkaline, rat catheter, Spider, flow model biofilm induced

orf19.4850	orf19.4850	86.14	85.31	81.92	90.86	0.004599726	0.998825	Ortholog(s) have cytosol, nucleus localization
orf19.543	FUM11	85.19	85.29	99.17	89.61	0.110924563	0.998825	Fumarate hydratase; induced in high iron; protein in exponential and stationary-phase yeast cells, but higher in stationary phase; rat catheter biofilm repressed
orf19.4128	orf19.4128	102.15	85.21	67.58	63.27	-0.408811887	0.545514	Ortholog of C. dubliniensis CD36 : Cd36_19390, C. parapsilosis CDC317 : CPAR2_209600, Candida tenuis NRRL Y-1498 : CANTEDRAFT_134010 and Debaryomyces hansenii CBS767 : DEHA2C14960g
orf19.4479	orf19.4479	108.72	85.19	80.9	69.87	-0.293557447	0.824505	Putative U3-containing 90S preribosome subunit; Hap43-induced; repressed in core stress response; Spider biofilm induced
orf19.4528	orf19.4528	94.11	85.17	110.57	110.51	0.243588753	0.880321	Ortholog(s) have role in vacuolar protein processing and endoplasmic reticulum localization
orf19.7436.1	ECM15	85.2	85.17	113.1	105.87	0.284718557	0.823496	Protein of unknown function; predicted role in cell wall organization; Hap43-repressed; caspofungin repressed; Spider biofilm induced; rat catheter biofilm repressed
orf19.6143	orf19.6143	102.05	84.97	100.99	76.1	-0.073997236	0.998825	Predicted long-chain-alcohol oxidase; Spider biofilm induced
orf19.1295	VAS1	86.14	84.91	99.77	88.05	0.097769024	0.998825	Putative tRNA-Val synthetase; genes encoding ribosomal subunits, translation factors, and tRNA synthetases are downregulated upon phagocytosis by murine macrophage
orf19.2102	CKB1	78.65	84.85	80.58	77.97	-0.046310032	0.998825	Regulatory subunit of protein kinase CK2 (casein kinase II), beta subunit; null mutants are hypersensitive to caspofungin and hydrogen peroxide medium

orf19.3103	orf19.3103	87.13	84.82	79.15	76.01	-0.128312642	0.998825	Ortholog(s) have RNA polymerase III activity, role in tRNA transcription from RNA polymerase III promoter and DNA-directed RNA polymerase III complex, cytoplasm localization
orf19.3778	orf19.3778	90.31	84.67	62.58	72.06	-0.297938478	0.833143	Protein with a predicted role in ribosome biogenesis; mutation confers hypersensitivity to 5-fluorocytosine (5-FC), 5-fluorouracil (5-FU); repressed in core stress response; repressed by prostaglandins; Hap43-induced
orf19.2712	HCA4	119.35	84.6	83.04	78.92	-0.269784346	0.861125	Putative role in regulation of cell wall biogenesis; Hap43p-induced gene; possibly an essential gene, disruptants not obtained by UAU1 method; flow model and rat catheter biofilm induced
orf19.5394.1	orf19.5394.1	69.18	84.58	65.57	77.51	-0.084122543	0.998825	Ortholog(s) have role in mitochondrial respiratory chain complex IV assembly and cytosol, integral to mitochondrial inner membrane, mitochondrial intermembrane space, nucleus localization
orf19.4587	HGH1	87.71	84.57	61.82	64.88	-0.353381805	0.692076	Putative HMG1/2-related protein; transcript regulated by Mig1
orf19.5492	orf19.5492	103.33	84.55	99.14	94.74	0.028317027	0.998825	Ortholog(s) have mRNA binding, pre-mRNA 5'-splice site binding activity, role in mRNA 5'-splice site recognition and U1 snRNP, U2-type prespliceosome, commitment complex localization
orf19.6313.2	MVB12	112.43	84.49	116.5	106.91	0.149051164	0.998825	ESCRT-I complex subunit with a role in multivesicular body (MVB) trafficking; mutant displays growth defect on alkaline medium; Hap43p-repressed gene
orf19.5106	DIP2	99.46	84.31	77.07	72.93	-0.234587562	0.918336	Putative small ribonucleoprotein complex; Tn mutation affects filamentous growth; physically interacts with TAP-tagged Nop1; heterozygous null mutant exhibits resistance to parnafungin; Hap43-induced gene; Spider biofilm induced
orf19.7267	SAM35	88.12	84.31	67.24	64.94	-0.305869672	0.816527	Predicted component of the sorting and assembly machinery (SAM complex) of the mitochondrial outer membrane, involved in protein import into mitochondria

orf19.5693	orf19.5693	82.65	84.31	127.42	112.37	0.408510384	0.480175	Subunit of the GPI (glycosylphosphatidylinositol):protein transamidase complex; removes the GPI-anchor signal and attaches GPI to proteins in the ER; Spider biofilm repressed
orf19.789	PYC2	72.56	84.27	90.3	104.45	0.2304217	0.924887	Putative pyruvate carboxylase; binds biotin cofactor; repressed by Ssk1 response regulator, by benomyl treatment, or in an azole-resistant strain overexpressing MDR1; stationary phase enriched protein; flow model biofilm repressed
orf19.2301	orf19.2301	86.22	84.26	73.35	68.92	-0.212784682	0.946319	Ortholog(s) have role in proteasome regulatory particle assembly and cytosol, nucleus localization
orf19.6811	ISA2	97.07	83.88	64.27	61.23	-0.422469451	0.525104	Protein required for maturation of mitochondrial [4Fe-4S] proteins; role in biotin biosynthetic process; rat catheter and Spider biofilm induced
orf19.2092	orf19.2092	93.75	83.7	98.73	90.69	0.065832357	0.998825	Putative peroxisomal cystathionine beta-lyase; Gcn4p-regulated
orf19.2863	orf19.2863	84.45	83.64	92.41	103.28	0.162738057	0.998825	Ortholog of C. dubliniensis CD36 : Cd36_28090, C. parapsilosis CDC317 : CPAR2_802560, Candida tenuis NRRL Y-1498 : CANTEDRAFT_104949 and Debaryomyces hansenii CBS767 : DEHA2A12848g
orf19.1861	orf19.1861	82.65	83.61	52.06	54.5	-0.495079745	0.377749	Protein of unknown function; flow model biofilm induced
orf19.1067	GPM2	85.08	83.6	68.66	87.47	-0.096845547	0.998825	Putative phosphoglycerate mutase; repressed in hyphae; macrophage/pseudohyphal-repressed; induced by high levels of peroxide stress, farnesol; flow model biofilm induced; rat catheter and Spider biofilm repressed
orf19.5525	orf19.5525	79.66	83.59	115.86	122.97	0.430140508	0.428049	Putative oxidoreductase; protein levels affected by URA3 expression in CAI-4 strain background; Efg1, Efh1 regulated; Rgt1-repressed; protein present in exponential and stationary growth phase yeast; rat catheter biofilm repressed

orf19.6831	PRP5	81.98	83.48	70.13	71.69	-0.177933591	0.998825	Putative pre-mRNA processing RNA-helicase; induced upon adherence to polystyrene; rat catheter and Spider biofilm induced
orf19.6273	orf19.6273	90.9	83.3	68.62	83.06	-0.158855166	0.998825	Ortholog of C. dubliniensis CD36 : Cd36_05960, C. parapsilosis CDC317 : CPAR2_301790, Candida tenuis NRRL Y-1498 : CANTEDRAFT_112420 and Debaryomyces hansenii CBS767 : DEHA2G15290g
orf19.585	orf19.585	92.68	83.3	99.77	90.39	0.078588899	0.998825	Ortholog(s) have structural constituent of ribosome activity and mitochondrial large ribosomal subunit localization
orf19.4525	orf19.4525	109.95	83.27	95.24	94.82	-0.024621474	0.998825	Ortholog(s) have endoplasmic reticulum, nuclear pore localization
orf19.3675	GAL7	55.43	83.2	24.98	42.85	-0.68415445	0.163231	Putative galactose-1-phoshphate uridyl transferase; downregulated by hypoxia, upregulated by ketoconazole; macrophage/pseudohyphal-repressed
orf19.3577	COQ5	81.97	83.17	120.88	121.84	0.444084893	0.38818	Putative methyltransferase of ubiquinone biosynthesis; regulated by Gcn4; repressed by amino acid starvation (3-AT), Hap43; induced upon adherence to polystyrene; Spider biofilm repressed
orf19.681	HAP43	80.83	83.14	84.79	87.56	0.050955516	0.998825	CCAAT-binding factor-dependent transcription factor; repressor; also called CAP2; required for low iron response; similar to bZIP transcription factor AP-1; repressed by Sfu1; ciclopirox olamine induced; rat catheter, Spider biofilm induced
orf19.3683	AGE3	93.04	83.13	103.47	101.61	0.168703161	0.998825	Putative ADP-ribosylation factor GTPase activating protein, functional ortholog of S. cerevisiae GCS1; mutation affects endocytosis, hyphal growth, chemical and drug resistance, and sensitivity to cell wall inhibitors
orf19.3192	STI1	92.68	83.11	91.91	86.75	0.010136789	0.998825	Protein that interacts with Cdc37 and Crk1 in two-hybrid; may be involved in Cdc37 chaperone activity; soluble protein in hyphae; protein in exponential and stationary phase yeast cultures; YNB biofilm induced; Spider biofilm induced

orf19.3060	orf19.3060	85.39	83.07	120.88	107.32	0.345786371	0.646416	OPutative dolichyl-diphosphooligosaccharide-protein glycotransferase; role in protein N-linked glycosylation; Spider biofilm repressed
orf19.5159	DUG3	80.98	82.77	64.61	69.48	-0.234255799	0.929262	Putative glutamine amidotransferase (GATase II); role in glutathione catabolism;
orf19.3402	FPG1	90.82	82.73	77.52	72.12	-0.177921308	0.998825	Formamidopyrimidine DNA glycosylase, involved in repair of gamma-irradiated DNA; Hap43p-repressed gene
orf19.6147	orf19.6147	92.84	82.71	103.44	79.77	0.033128155	0.998825	Putative histone chaperone; role in chromatin remodeling; rat catheter and Spider biofilm repressed
orf19.7064	GLN4	85.29	82.57	81.84	78.04	-0.065713876	0.998825	Putative tRNA-Gln synthetase; genes encoding ribosomal subunits, translation factors, tRNA synthetases are downregulated upon phagocytosis by murine macrophage; protein present in exponential and stationary growth phase yeast cultures
orf19.5768	SNF4	96.03	82.57	101.54	90.33	0.071407915	0.998825	Transcription factor; ortholog of S. cerevisiae Snf4; caspofungin repressed; transposon mutation affects filamentation
orf19.3276	PWP2	93.13	82.46	82.93	77.25	-0.11142928	0.998825	Putative 90S pre-ribosomal component; repressed in core stress response; repressed by prostaglandins; physically interacts with TAP-tagged Nop1; Hap43-induced
orf19.6556	orf19.6556	73.35	82.42	38.39	42	-0.700492006	0.100263	Protein of unknown function; rat catheter, flow model and Spider biofilm induced
orf19.3532	MRPL10	86.89	82.18	81.99	85.95	-0.015497956	0.998825	Putative mitochondrial large subunit ribosomal protein; colony morphology-related gene regulation by Ssn6

orf19.6553	orf19.6553	95.66	82.15	109.96	108.48	0.228710139	0.90951	Membrane-localized protein of unknown function; possibly secreted; fluconazole-induced
orf19.5658	MNN10	83.42	82.08	80.58	73.83	-0.081531374	0.998825	Ortholog(s) have alpha-1,6-mannosyltransferase activity, role in ascospore formation, barrier septum assembly and alpha-1,6-mannosyltransferase complex localization
orf19.1166	CTA3	83.05	82.08	99.99	97.59	0.200410045	0.951606	Protein similar to S. cerevisiae Ede1p, which is involved in endocytosis; activates transcription in 1-hybrid assay in S. cerevisiae
orf19.5232	CSI2	101.15	81.92	83.24	78.47	-0.154342328	0.998825	Putative 66S pre-ribosomal particle component; Hap43-induced; essential for growth; transposon mutation affects filamentous growth; Spider biofilm induced
orf19.802	UGA1	103.42	81.91	50.85	31.26	-0.867904892	0.016841	Putative GABA transaminase; transcription regulated by Mig1 and Tup1; stationary phase enriched protein; rat catheter and Spider biofilm induced
orf19.4066	orf19.4066	99.09	81.84	90.2	81.38	-0.074096715	0.998825	Putative glycerol-3-phosphate acyltransferase; Hog1-repressed
orf19.3630	RRP8	106.52	81.79	79.43	64.9	-0.315354471	0.788835	Ribosomal protein; Hap43-induced; F-12/CO2 early biofilm and rat catheter biofilm induced
orf19.3978	orf19.3978	106.19	81.76	71.4	59.87	-0.412871497	0.543746	Protein required for maturation of 18S rRNA; rat catheter biofilm repressed
orf19.367	CNH1	85.56	81.69	97.44	89.96	0.113012597	0.998825	Na+/H+ antiporter; required for wild-type growth, cell morphology, and virulence in a mouse model of systemic infection; not transcriptionally regulated by NaCl; fungal-specific (no human or murine homolog)

orf19.3628	RSP5	83.71	81.68	84.14	75.57	-0.052603181	0.998825	Putative NEDD4 family E3 ubiquitin ligase; induced during infection of murine kidney, compared to growth in vitro; has murine homolog; possibly an essential gene, disruptants not obtained by UAU1 method
orf19.498	orf19.498	75.28	81.58	82.33	74.64	-0.012025776	0.998825	Putative mitochondrial ribosomal component of the small subunit; possibly an essential gene, disruptants not obtained by UAU1 method; Spider biofilm repressed
orf19.240	PAM17	71.75	81.47	80.24	84.02	0.067180823	0.998825	Predicted component of the presequence translocase-associated import motor (PAM complex) involved in protein import into mitochondrial matrix
orf19.3559	orf19.3559	102	81.37	101.73	95.7	0.083471946	0.998825	S. cerevisiae ortholog Mrps35p is a structural constituent of ribosome and localizes to mitochondrial small ribosomal subunit; the snoRNA CD39 is encoded within the MRPS35 intron
orf19.1281	orf19.1281	76.18	81.35	54.97	66.74	-0.283154098	0.866583	Putative splicing factor required for the first step of pre-mRNA splicing; Spider biofilm induced
orf19.3483	orf19.3483	88.59	81.34	52.92	52.28	-0.535539378	0.284574	Putative phosphatidyl glycerol phospholipase C; Plc1-regulated; flow model biofilm induced; Spider biofilm induced
orf19.4395	orf19.4395	97.65	81.31	122.38	96.03	0.218558197	0.931333	Putative actin cytoskeleton component; protein present in exponential and stationary growth phase yeast cultures
orf19.1797	orf19.1797	106.37	81.25	56.5	47.04	-0.650218677	0.120567	D-arabinose 5-phosphate isomerase; has GutQ domain which is associated with phosphosugar binding; other biofilm induced; rat catheter and Spider biofilm induced; F-12/CO2 early biofilm induced
orf19.886	PAN1	74.33	81.25	105.4	92.49	0.261766475	0.870631	Essential protein involved in endocytosis and polarized growth; ortholog of S. cerevisiae Pan1, which is a part of a complex that regulates actin cytoskeleton; Spider biofilm repressed

orf19.3327	TRM2	89.33	81.22	79.24	79.09	-0.091948503	0.998825	Putative tRNA methyltransferase; repressed by prostaglandins; Spider biofilm induced
orf19.6344	RBK1	88.42	81.2	92.81	86.87	0.06260143	0.998825	Ortholog(s) have cytosol, nucleus localization
orf19.7359	CRZ1	66.75	81.11	79.24	67.04	-0.02889724	0.998825	Calcineurin-regulated C2H2 transcription factor; role in maintenance of membrane integrity, azole tolerance; not required for mouse virulence; repressed by low iron; regulates Ca++ influx during alkaline pH response; Spider biofilm induced
orf19.6652	DBP8	88.71	81.05	65.93	63.23	-0.316994917	0.789669	Protein similar to S. cerevisiae Dbp8p, an ATP-dependent helicase involved in rRNA processing; oxidative stress-repressed via Cap1p; flucytosine repressed; likely to be essential for growth, based on insertional mutagenesis
orf19.7306	orf19.7306	99.87	81.01	38.09	40.39	-0.90224791	0.010366	Aldo-keto reductase; increased transcript associated with MDR1 overexpression, benomyl or long-term fluconazole treatment; overexpression does not affect drug or oxidative stress sensitivity; stationary phase enriched; flow biofim repressed
orf19.4582	orf19.4582	93.68	80.95	81.29	72.54	-0.154378568	0.998825	Ortholog(s) have role in U1 snRNA 3'-end processing, U4 snRNA 3'-end processing and U5 snRNA 3'-end processing, more
orf19.6010.1	RPB11	97.61	80.95	89.32	96.76	0.037520557	0.998825	Putative RNA polymerase II subunit; flucytosine induced
orf19.1979	GIT3	89.63	80.81	66.32	58.1	-0.366910848	0.66298	Glycerophosphocholine permease; white cell specific transcript; fungal-specific; alkaline repressed; caspofungin, macrophage/pseudohyphal-repressed; flow model biofilm induced; Spider biofilm induced
orf19.330.1	RUB1	61.52	80.77	94.32	95.19	0.299698597	0.824505	Ubiquitin-related protein with similarity to mammalian NEDD8; reversible conjugation (neddylation) of the cullin subunit (Cdc53p) modulates the ubiquitin ligase activity of the SCF complexes

orf19.3206	CCT7	86.17	80.73	82.91	74.11	-0.080511753	0.998825	Cytosolic chaperonin Cct ring complex; protein is present in exponential and stationary growth phase yeast cultures; sumoylation target
orf19.5059	GCS1	92.85	80.72	62.5	64.22	-0.358958188	0.680517	Gamma-glutamylcysteine synthetase; glutathione synthesis, required for virulence;induced in low iron, H2O2, Cd, or presence of human neutrophils; possibly adherence-induced; Spider and F-12/CO2 biofilm induced
orf19.6461	orf19.6461	94.31	80.65	85.75	79.66	-0.069335084	0.998825	Ortholog(s) have unfolded protein binding activity, role in aerobic respiration, mitochondrial respiratory chain complex IV assembly, protein processing and mitochondrial inner membrane localization
orf19.4491	ERG20	88.76	80.53	89.59	88.66	0.050207027	0.998825	Putative farnesyl pyrophosphate synthetase involved in isoprenoid and sterol biosynthesis, based on similarity to S. cerevisiae Erg20p; likely to be essential for growth, based on an insertional mutagenesis strategy
orf19.7141	UFE1	71.08	80.5	78.67	73.31	-0.012346195	0.998825	Protein interacting with Sec20p, possibly involved in retrograde transport between the Golgi and the endoplasmic reticulum; functional homolog of S. cerevisiae Ufe1p, which is an ER t-SNARE that mediates the retrograde traffic
orf19.1011	MNN21	84.04	80.46	95.64	100.53	0.202465526	0.951606	Ortholog(s) have alpha-1,2-mannosyltransferase activity, role in cell wall mannoprotein biosynthetic process and Golgi apparatus localization
orf19.6416	orf19.6416	94.91	80.46	80.94	66.8	-0.198876249	0.965764	Protein involved in N-glycosylation; Spider biofilm induced; rat catheter biofilm repressed
orf19.134	orf19.134	77.08	80.45	87.52	103.42	0.21456183	0.943903	Protein of unknown function; overlaps retrotransposon Tca13; ciclopirox olamine induced
orf19.3106	MET16	78.71	80.44	105.08	103.52	0.305825525	0.786979	Putative 3'-phosphoadenylsulfate reductase; sulfur amino acid metabolism; reports differ on regulation during biofilm formation; fungal-specific; possibly an essential gene, disruptants not obtained by UAU1 method; Hap43p-repressed gene

orf19.1304	orf19.1304	77.56	80.43	65.39	65.56	-0.217412761	0.946319	Ortholog(s) have role in U4 snRNA 3'-end processing, exonucleolytic trimming to generate mature 3'-end of 5.8S rRNA from tricistronic rRNA transcript (SSU-rRNA, 5.8S rRNA and LSU-rRNA), more
orf19.6378	TRM9	88.04	80.37	69.72	70.16	-0.213289048	0.946319	Putative tRNA methyltransferase; repressed during the mating process
orf19.4669	AAT22	74.46	80.37	85.49	79.69	0.066218338	0.998825	Aspartate aminotransferase; nitrogen metabolism; similar but not orthologous to S. cerevisiae Aat2; clade-associated gene expression; protein levels decrease in stationary phase yeast; mutant is viable; flow model biofilm repressed
orf19.1485	orf19.1485	75.45	80.18	87.1	114.9	0.291986607	0.840766	Mitochondrial ribosomal protein of the large subunit; rat catheter biofilm induced
orf19.7506	orf19.7506	85.09	80.14	76.07	63.86	-0.194818622	0.98002	Ortholog(s) have ATPase activity, DNA binding, nucleosome binding activity, role in chromatin remodeling and Isw1 complex localization
orf19.7410	CYM1	88.27	80.12	69.83	70.57	-0.205231821	0.957711	Putative metalloprotease of the mitochondrial intermembrane space; rat catheter biofilm induced
orf19.992	LKH1	83.18	80.08	87.99	83.9	0.051191673	0.998825	Putative leukotriene A(4) hydrolase; repressed during the mating process; Hog1p-induced
orf19.7193	orf19.7193	77.92	80.07	78.43	75.76	-0.039363296	0.998825	Specificity factor required for ubiquitination; role in protein targeting to vacuole; involved in ubiquitin-dependent protein catabolism via the multivesicular body sorting pathway; Spider biofilm induced
orf19.2268	RCK2	86.53	80.02	93.08	87.44	0.076208993	0.998825	Predicted MAP kinase-activated protein kinase, similar to S. cerevisiae serine/threonine protein kinase Rck2p; induced by osmotic stress via Hog1p; macrophage/pseudohyphal-repressed; mutants are sensitive to rapamycin

orf19.3755	orf19.3755	95.4	80	88.49	89.6	0.011878251	0.998825	Ortholog(s) have structural constituent of ribosome activity and mitochondrial large ribosomal subunit localization
orf19.3873	ARC40	75.71	79.91	96.41	101.87	0.263343146	0.87024	Protein similar to S. cerevisiae Arc40; involved in actin filament organization in S. cerevisiae; transposon mutation affects filamentous growth; rat catheter and Spider biofilm repressed
orf19.407	GCD6	81.78	79.9	94.3	100.63	0.204233646	0.951606	Ortholog of S. cerevisiae Gcd6; catalytic epsilon subunit of the translation initiation factor eIF2B; genes encoding translation factors are repressed by phagocytosis by murine macrophages
orf19.5825	NCB2	70.7	79.89	71.39	67.41	-0.111301431	0.998825	Beta subunit of NC2, heterodimeric regulator of transcription; activates CDR1 transcription; possibly an essential gene, disruptants not obtained by UAU1 method; conditional mutants show decreased susceptibility to azoles
orf19.6717	orf19.6717	96.77	79.88	83.1	86.23	-0.060033043	0.998825	Putative serine hydrolase; Spider biofilm repressed
orf19.6030	orf19.6030	91.19	79.8	101.99	88.58	0.117487551	0.998825	Protein of unknown function; transcript detected on high-resolution tiling arrays
orf19.6470	AHP2	79.41	79.73	113.58	103.46	0.349232	0.649609	Putative thiol-specific peroxiredoxin; macrophage-downregulated gene
orf19.4028	orf19.4028	81.63	79.69	77.98	73.75	-0.082038944	0.998825	Putative cis-prenyltransferase involved in dolichol synthesis; participates in endoplasmic reticulum (ER) protein sorting; flow model biofilm induced
orf19.3700	TOM70	96.98	79.69	93.4	92.47	0.043159434	0.998825	Ortholog(s) have mitochondrion targeting sequence binding, protein channel activity and role in protein import into mitochondrial inner membrane, protein import into mitochondrial matrix

orf19.2154	HXK1	82.23	79.68	95.3	90.99	0.148280164	0.998825	N-acetylglucosamine (GlcNAc) kinase; involved in GlcNAc utilization; required for wild-type hyphal growth and mouse virulence; GlcNAc-induced transcript; induced by alpha pheromone in SpiderM medium
orf19.4892	TPK1	92.9	79.64	48.01	44.77	-0.679495478	0.099285	cAMP-dependent protein kinase catalytic subunit; Tpk2 isoform; control of morphogenesis and stress response; WT nuclear localization requires Bcy1; produced during stationary, not exponential growth; rat catheter and Spider biofilm induced
orf19.606	orf19.606	85.92	79.61	70.46	61.57	-0.265711967	0.878136	Protein of unknown function; Hap43-induced; Spider biofilm induced
orf19.1381	orf19.1381	71.97	79.57	92.86	80.28	0.133059565	0.998825	Ortholog of S. cerevisiae/S. pombe Lsb5; predicted role in actin cortical patch localization, actin filament organization, endocytosis; flow model biofilm induced; Spider biofilm repressed
orf19.4735	orf19.4735	81.52	79.52	99.48	91.59	0.178768756	0.998825	Ornithine cyclodeaminase family protein; Sef1, Sfu1, and Hap43-regulated; ortholog of S. cerevisiae YGL159W and S. pombe SPAP11E10.01; rat catheter biofilm induced
orf19.4199	SLY41	83.09	79.51	58.08	59.82	-0.364362215	0.680517	Protein involved in ER-to-Golgi transport; rat catheter and Spider biofilm induced
orf19.3329	orf19.3329	80.8	79.51	104	101.69	0.274917276	0.851328	Ortholog(s) have sphingosine-1-phosphate phosphatase activity, role in calcium-mediated signaling and endoplasmic reticulum localization
orf19.906	ROM2	76.3	79.2	82.76	81.97	0.06006332	0.998825	Putative GDP/GTP exchange factor; possibly an essential gene, disruptants not obtained by UAU1 method; Spider biofilm induced
orf19.3515	orf19.3515	80.3	79.13	77.06	71.74	-0.083440255	0.998825	Putative 3-hydroxyanthranilic acid dioxygenase, involved in NAD biosynthesis; Hap43p-repressed gene

orf19.5834	orf19.5834	77.66	79.1	82.33	82.11	0.039216293	0.998825	Ortholog(s) have protein transporter activity, role in mRNA export from nucleus and cytoplasm, nucleus localization
orf19.7210	orf19.7210	92.77	79.09	92.15	79.8	-0.00960814	0.998825	Protein of unknown function; Spider biofilm induced
orf19.5073	DPM1	72.03	78.91	117.43	145.46	0.629220326	0.09674	Putative dolichol phosphate mannose synthase; filament induced; Tup1-regulated; flow model and rat catheter biofilm repressed
orf19.7547	orf19.7547	94.79	78.87	94.19	95.86	0.093923939	0.998825	Ortholog(s) have phosphatidylinositol-3-phosphate binding, ubiquitin-protein ligase activity, role in protein ubiquitination and cytosol, fungal-type vacuole membrane, late endosome, nucleus localization
orf19.7507	orf19.7507	93.34	78.84	102.42	77.62	0.040760959	0.998825	Ortholog of C. dubliniensis CD36 : Cd36_25310, Pichia stipitis Pignal : PICST_30877, Candida tropicalis MYA-3404 : CTRG_01053 and Candida lusitaniae ATCC 42720 : CLUG_02644
orf19.2208	orf19.2208	78.07	78.82	90.27	78.25	0.064906729	0.998825	Ortholog(s) have role in response to salt stress and cytoplasm localization
orf19.6738	VAN1	82.67	78.82	96.07	93.22	0.167335189	0.998825	Member of Mnn9 family of mannosyltransferases; ortholog of S. cerevisiae Van1p; fungal-specific (no human or murine homolog)
orf19.431	ZCF2	72.42	78.82	49.6	45.7	-0.493949135	0.404341	Zn(II)2Cys6 transcription factor; regulates sulfite tolerance through expression of SSU1 and CDG1; Hap43-repressed; Spider biofilm induced
orf19.1722	orf19.1722	151.26	78.71	79	53.27	-0.602824058	0.200739	Ortholog of Candida albicans WO-1 : CAWG_02462

orf19.491	orf19.491	80.37	78.7	109.01	100.38	0.305371529	0.787177	Endoplasmic reticulum (ER) protein-translocation complex subunit
orf19.519	orf19.519	80.03	78.69	77.19	82.08	-0.009050517	0.998825	Ortholog(s) have RNA polymerase I upstream control element sequence-specific DNA binding, RNA polymerase I upstream control element sequence-specific DNA binding transcription factor activity
orf19.7297	orf19.7297	83.57	78.66	94.72	99.87	0.197644983	0.957711	Putative cystathionine gamma-synthase; decreased levels in stationary phase cultures; Hog1p-induced; Gcn4p-regulated
orf19.1183	orf19.1183	84.19	78.29	111.42	97.19	0.27849797	0.843898	Ortholog(s) have fungal-type vacuole localization
orf19.2488	FAL1	95.74	78.22	66.2	57.45	-0.393174793	0.598332	eIF4A subfamily of DEAD-box ATP-dependent RNA helicases; predicted nucleolar protein required for maturation of 18S rRNA; Spider biofilm induced
orf19.1287	orf19.1287	67.5	78.15	60.77	58.44	-0.225707093	0.946319	Protein of unknown function; flow model biofilm induced; Spider biofilm induced
orf19.4609	orf19.4609	86.98	78.07	101.34	116.91	0.315401928	0.752503	Putative dienelactone hydrolase; protein abundance is affected by URA3 expression in the CAI-4 strain background; protein present in exponential and stationary growth phase yeast cultures; rat catheter biofilm repressed
orf19.1839	RPA190	80.94	78.05	77.25	70.74	-0.09080219	0.998825	Putative RNA polymerase I subunit A190; Hap43p-induced gene; flucytosine induced
orf19.125	EBP1	73.12	78.03	25.66	25.42	-1.085695681	0.001922	NADPH oxidoreductase; interacts with phenolic substrates (17beta-estradiol); possible role in estrogen response; induced by oxidative, weak acid stress, NO, benomyl, GlcNAc; Cap1, Mnl1 induced; Hap43-repressed; rat catheter biofilm induced

orf19.410.3	RIB4	85.77	77.98	122.12	143.12	0.556107899	0.17099	Lumazine synthase (6,7-dimethyl-8-ribityllumazine synthase, DMRL synthase); catalyzes the penultimate step in the synthesis of riboflavin; Hap43-induced; rat catheter and Spider biofilm repressed
orf19.6713	WOR4	89.19	77.98	112.97	90.61	0.219908412	0.931333	Has domain(s) with predicted nucleic acid binding activity
orf19.6075	orf19.6075	93.91	77.94	85.18	72.42	-0.112807706	0.998825	Putative CCR4-Not complex transcription factor; ortholog of S. cerevisiae Cdc36; Hap43-repressed gene
orf19.3585	orf19.3585	64.68	77.89	57.9	56.86	-0.245933924	0.924887	Putative tRNA methyltransferase complex subunit; regulated by Gcn4; repressed in response to amino acid starvation (3-aminotriazole treatment); Spider biofilm induced
orf19.4447	YMC1	82.49	77.83	95.48	80.75	0.097328719	0.998825	Putative inner mitochondrial membrane transporter; flucytosine induced; Spider biofilm repressed
orf19.3900	orf19.3900	84.08	77.81	66.55	60.41	-0.279744307	0.861217	Ortholog(s) have role in mitochondrial fusion and integral to mitochondrial outer membrane localization
orf19.6987	DNM1	85.98	77.8	104.05	86.27	0.157783213	0.998825	Putative dynamin-related GTPase; transcript upregulated inbRHE model of oral candidiasis; transcript regulated by Nrg1, Mig1, and Tup1
orf19.5433	orf19.5433	77.18	77.72	75.83	89.47	0.062010523	0.998825	Ortholog(s) have protein binding involved in protein folding activity, role in mitochondrial respiratory chain complex III assembly and mitochondrial matrix localization
orf19.6187	orf19.6187	77.96	77.59	80.66	76.68	0.003856994	0.998825	possible pseudouridine monophosphate glycosidase; overlaps orf19.6185 and orf19.618; has intron; Spider biofilm repressed

orf19.1047	ERB1	85.7	77.57	68.44	63.15	-0.259453593	0.885512	Protein with a predicted role in ribosomal large subunit biogenesis; mutation confers hypersensitivity to 5-fluorocytosine (5-FC), 5-fluorouracil (5-FU), and tubercidin (7-deazaadenosine); hyphal, macrophage repressed
orf19.5920	orf19.5920	73.69	77.54	58.99	63.79	-0.240879505	0.925649	Ortholog of C. dubliniensis CD36 : Cd36_84570, C. parapsilosis CDC317 : CPAR2_404420, Candida tenuis NRRL Y-1498 : CANTEDRAFT_115339 and Debaryomyces hansenii CBS767 : DEHA2G13618g
orf19.6686	ENP2	82.09	77.47	65.43	53.62	-0.32874906	0.782481	Putative nucleolar protein; essential; heterozygous mutation confers resistance to 5-fluorocytosine (5-FC), 5-fluorouracil (5-FU), and tubercidin (7-deazaadenosine); Hap43-induced; Spider biofilm induced
orf19.1708	orf19.1708	90.61	77.47	69.16	69.12	-0.22787091	0.933046	Protein of unknown function; Spider biofilm induced
orf19.2735	SEN2	81.28	77.41	64.12	70.32	-0.190793363	0.994258	Putative tRNA splicing endonuclease subunit; mutation confers hypersensitivity to toxic ergosterol analog and to amphotericin B; 5'-UTR intron; Hap43-induced; Spider biofilm induced
orf19.6100	orf19.6100	83.16	77.36	70.38	66.95	-0.182441311	0.998825	Cardiolipin synthase; ortholog of S. cerevisiae Crd1; transcript is upregulated in clinical isolates from HIV+ patients with oral candidiasis; mutants are viable; Spider biofilm repressed
orf19.7274	orf19.7274	84.15	77.36	82.57	80.22	0.00468465	0.998825	Predicted ORF in retrotransposon Zorro2 with similarity to retroviral reverse transcriptase proteins
orf19.2108	SOD6	70.14	77.26	93.53	90.71	0.249278674	0.892679	Copper- and zinc-containing superoxide dismutase; gene family includes SOD1, SOD4, SOD5, and SOD6; gene may contain an intron; Hap43-repressed; flow model and rat catheter biofilm induced
orf19.1597	ABG1	85.32	77.2	104.51	94.43	0.227125139	0.921805	Vacuolar membrane protein; depletion causes abnormal vacuolar morphology, cell separation defect, sensitivity to cell wall stress, increased hyphal branching; essential, no mammalian homolog; Cyr1-regulated; rat catheter biofilm repressed

orf19.4754	ZWF1	88.58	77.19	175.47	148.34	0.786090092	0.008624	Glucose-6-phosphate dehydrogenase; antigenic in mice; activity induced by O2 or oxidizing agents H2O2, menadione, macrophage; caspofungin repressed; induced in core stress response; regulated by Gcn2, Gcn4; rat catheter biofilm repressed
orf19.6992	QDR2	108.79	77.17	39.23	33.82	-0.989187313	0.004002	Predicted MFS membrane transporter, member of the drug:proton antiporter (12 spanner) (DHA1) family; Spider biofilm induced
orf19.3959	SSD1	72.93	77.13	91.3	81.88	0.149472602	0.998825	Protein with role in resistance to host antimicrobial peptides; virulence role in murine infection; functional homolog of S. cerevisiae Ssd1p, which suppresses various mutant phenotypes; constitutively expressed and not cell-cycle regulated
orf19.5195	URA6	76.88	77.08	76.68	75.06	-0.024834072	0.998825	Ortholog(s) have adenylate kinase activity, uridylate kinase activity, role in 'de novo' pyrimidine nucleobase biosynthetic process and cytosol, mitochondrion, nucleus localization
orf19.3929	orf19.3929	90.18	77.08	71.84	67.28	-0.214209351	0.946319	Ortholog(s) have mitochondrion localization
orf19.3458	orf19.3458	108.01	76.97	96.35	77.11	-0.084387174	0.998825	Ortholog(s) have role in late endosome to vacuole transport via multivesicular body sorting pathway, protein targeting to vacuole and Golgi apparatus, Vps55/Vps68 complex, fungal-type vacuole membrane, mitochondrion localization
orf19.2322.3	ERP5	88.12	76.97	126.13	117.32	0.445095356	0.384933	Protein involved in ER to Golgi transport; rat catheter and Spider biofilm repressed
orf19.2143	orf19.2143	91.42	76.95	72.84	64.41	-0.237193066	0.921805	Ortholog(s) have role in endocytic recycling and cytosol, endosome, nucleus localization
orf19.5076.1	orf19.5076.1	69.26	76.93	68.11	59.45	-0.164632214	0.998825	Ortholog of C. parapsilosis CDC317 : CPAR2_804320, Candida tenuis NRRL Y-1498 : CANTEDRAFT_114112, Debaryomyces hansenii CBS767 : DEHA2G14938g and Candida orthopsilosis Co 90-125 : CORT_0A04360

orf19.5350	orf19.5350	91.88	76.86	93.8	88.56	0.082889487	0.998825	Ortholog(s) have cyclin-dependent protein serine/threonine kinase activity
orf19.1669	AFG3	94.69	76.65	97.6	78.36	0.016174072	0.998825	Similar to S. cerevisiae Afg3p, a subunit of the mitochondrial inner membrane m-AAA protease; likely to be essential for growth, based on an insertional mutagenesis strategy
orf19.1946	orf19.1946	86.9	76.57	159.28	142	0.713961766	0.023763	Similar to an aldose 1-epimerase-related protein; antigenic during murine systemic infection; protein present in exponential and stationary phase yeast cultures; Hap43-induced; Spider biofilm repressed
orf19.4864	orf19.4864	85.63	76.5	133.05	106.18	0.43478933	0.41868	Ortholog(s) have acylglycerol lipase activity, serine hydrolase activity, role in triglyceride metabolic process and lipid particle, mitochondrial outer membrane, plasma membrane localization
orf19.3141	orf19.3141	69.4	76.49	83.26	77.89	0.105304078	0.998825	Ortholog(s) have role in ER to Golgi vesicle-mediated transport and cytoplasmic mRNA processing body, endoplasmic reticulum membrane, extrinsic to membrane localization
orf19.5337	UBC15	68.73	76.46	110.56	136.29	0.603647736	0.129689	Putative E2 ubiquitin-conjugating enzyme
orf19.6403	orf19.6403	97.74	76.36	93.73	90.54	0.063133623	0.998825	Ortholog(s) have adenyl-nucleotide exchange factor activity, role in SRP-dependent cotranslational protein targeting to membrane, translocation and endoplasmic reticulum localization
orf19.1386	orf19.1386	73.68	76.27	73.48	60.84	-0.136331399	0.998825	Ortholog(s) have SNAP receptor activity and role in ER to Golgi vesicle-mediated transport, retrograde vesicle-mediated transport, Golgi to ER, vesicle fusion
orf19.5133	ZCF29	65.64	76.25	57.57	57.15	-0.238303108	0.932999	Zn(II)2Cys6 transcription factor; mutants sensitive to caffeine and menadione, resistant to fenpropimorph; Hap43-repressed; mutants have decreased CFU in mouse kidneys and defects in filamentous growth; Spider biofilm induced

orf19.1649	RNA1	83.91	76.21	93.13	88.14	0.131596682	0.998825	Putative GTPase-activating protein; protein level decreases in stationary phase cultures; Spider biofilm repressed
orf19.5383	PMA1	61.01	76.2	67.96	82.69	0.095851538	0.998825	Plasma membrane H(+)-ATPase; highly expressed, comprises 20-40% of total plasma membrane protein; levels increase at stationary phase transition; fluconazole induced; caspofungin repressed; upregulated in RHE model; Spider biofilm repressed
orf19.2172	ARA1	87.34	76.16	94.02	88.02	0.117139806	0.998825	D-Arabinose dehydrogenase; dehydro-D-arabinono-1,4-lactone synthesis; active on D-arabinose, L-fucose, L-xylose, L-galactose; inhibited by metal ions, thiol group-specific reagents; induced on polystyrene adherence; Spider biofilm induced
orf19.4000	GRF10	80.29	76.13	71.56	71.34	-0.106530434	0.998825	Putative homeodomain transcription factor; null mutant is an adenine auxotroph; Spider biofilm induced; promoter bound by Bcr1, Tec1, Efg1, Ndt80 and Brg1
orf19.3862	orf19.3862	89.85	76.06	77.03	69.56	-0.14551003	0.998825	Putative intracellular transport protein; heterozygous null mutant displays sensitivity to rapamycin; expression upregulated during growth in the mouse cecum
orf19.3616	ERG9	73.36	75.97	83.26	89.58	0.158959999	0.998825	Putative farnesyl-diphosphate farnesyl transferase (squalene synthase); sterol biosynthesis pathway; likely essential for growth; regulated by fluconazole, lovastatin; amphotericin B, caspofungin repressed; Spider biofilm repressed
orf19.3242	orf19.3242	80.63	75.92	80.93	65.33	-0.092535687	0.998825	Putative TFIID and SAGA complex subunit; possibly an essential gene, disruptants not obtained by UAU1 method
orf19.4474	orf19.4474	101.21	75.88	70.61	74.04	-0.229553136	0.931333	Ortholog(s) have cytoplasm, nucleus localization
orf19.4720	CTR2	80.27	75.82	59.64	66.41	-0.243037163	0.919016	Putative low-affinity copper transporter of the vacuolar membrane; induced by nitric oxide; clade-associated gene expression; rat catheter and flow model biofilm induced

orf19.2419	orf19.2419	85.03	75.8	92.08	80.65	0.071759501	0.998825	Ortholog of S. cerevisiae Dom34; a protein that facilitates ribosomal subunit dissociation when translation is stalled; peloto ortholog; Hap43-repressed gene
orf19.1412	orf19.1412	73.39	75.8	70.46	76.19	-0.031929946	0.998825	Ortholog of C. dubliniensis CD36 : Cd36_44020, C. parapsilosis CDC317 : CPAR2_302330, Candida tenuis NRRL Y-1498 : CANTEDRAFT_105448 and Debaryomyces hansenii CBS767 : DEHA2C10582g
orf19.2320	orf19.2320	87.38	75.79	67.21	60.28	-0.285738959	0.858075	Putative serine/threonine-protein kinase; possibly an essential gene, disruptants not obtained by UAU1 method
orf19.2638.1	orf19.2638.1	51.51	75.72	37.27	52.35	-0.371665154	0.743771	Ortholog of C. parapsilosis CDC317 : CPAR2_502070, Candida tenuis NRRL Y-1498 : CANTEDRAFT_115402, Lodderomyces elongisporus NRLL YB-4239 : LELG_04205 and Debaryomyces hansenii CBS767 : DEHA2E22660g
orf19.4363	SGD1	83.27	75.72	63.82	62.58	-0.257383076	0.892679	Predicted small ribosomal subunit biogenesis protein; repressed in core stress response; transcript increases in populations of cells exposed to fluconazole over multiple generations; Spider biofilm induced
orf19.607	orf19.607	83.65	75.61	70.51	60.76	-0.223638818	0.938642	Protein involved in transcription-coupled nucleotide excision repair of UV-induced DNA lesions; Spider biofilm induced
orf19.2276	orf19.2276	93.6	75.57	69.41	66.99	-0.25675989	0.888717	Ortholog(s) have DNA-directed RNA polymerase activity, RNA-directed RNA polymerase activity
orf19.7667	IAH1	75.69	75.43	87.48	82.5	0.123765425	0.998825	Protein similar to S. cerevisiae Iah1p, which is involved in acetate metabolism; mutation confers hypersensitivity to tunicamycin; transposon mutation affects filamentous growth
orf19.3895	CHT2	73.95	75.41	100.48	72.66	0.153608864	0.998825	GPI-linked chitinase; required for normal filamentous growth; repressed in core caspofungin response; fluconazole, Cyr1, Efg1, pH-regulated; mRNA binds She3 and is localized to yeast-form buds and hyphal tips; Spider biofilm repressed

orf19.6408	orf19.6408	85.39	75.15	63.99	51.26	-0.370968637	0.672744	Putative DnaJ-like heat shock/chaperone; Hap43-repressed; Spider and F-12/CO2 biofilm induced
orf19.3150	GRE2	84.55	75.09	98.98	88.8	0.175139649	0.998825	Putative reductase; Nrg1 and Tup1-regulated; benomyl- and hyphal-induced; macrophage/pseudohyphal-repressed; repressed by low iron; possibly involved in osmotic stress response; stationary phase enriched protein; Spider biofilm induced
orf19.934	orf19.934	97.21	75.09	92.55	89.35	0.057395395	0.998825	Predicted ORF from Assembly 19; removed from Assembly 20; subsequently reinstated in Assembly 21 and merged with orf19.2224, based on comparative genome analysis; flow model biofilm repressed
orf19.1139	orf19.1139	70.73	75.05	72.74	71.07	-0.02603769	0.998825	Protein of unknown funtion; S. cerevisiae ortholog Svl3 plays a role in endocytosis and is localized to the bud neck; Spider biofilm induced
orf19.3809	BAS1	72.27	75	63.02	61.07	-0.196490503	0.987997	Putative Myb-like transcription factor; ortholog S. cerevisiae Bas1, a regulator of purine biosynthetic genes; mutant exhibits adenine auxotrophy and abnormal colony morphology
orf19.4219	RIX7	82.73	74.98	69.92	68.18	-0.159679506	0.998825	Putative ATPase of the AAA family; role in ribosomal subunit export from the nucleus; mutation impairs hyphal growth and biofilm formation
orf19.5365	orf19.5365	69.14	74.83	74	76.93	0.042332379	0.998825	S. cerevisiae ortholog YMR259C interacts with Trm7 for 2'-O-methylation of C32 of substrate tRNAs; downregulated by fluphenazine treatment or in an azole-resistant strain that overexpresses CDR1 and CDR2
orf19.2557	SEC65	75.75	74.81	72.8	69.56	-0.070704359	0.998825	Component of the protein-targeting Signal Recognition Particle (SRP); similar to mammalian Srp19; functional homolog of S. cerevisiae Sec65; Tbf1-induced
orf19.366	orf19.366	76.45	74.75	86.1	78.62	0.089288688	0.998825	Ortholog(s) have double-stranded DNA binding activity, role in DNA damage checkpoint, double-strand break repair, reciprocal meiotic recombination and checkpoint clamp complex localization

orf19.5824	orf19.5824	97.46	74.75	69.1	59.17	-0.335609547	0.748714	Protein of unknown function; rat catheter and Spider biofilm induced
orf19.6555	orf19.6555	76.46	74.73	79.08	89.77	0.11851024	0.998825	Ortholog(s) have zinc ion binding activity, role in protein import into mitochondrial intermembrane space and cytosol, mitochondrial intermembrane space, nucleus localization
orf19.336	YAH1	77.09	74.38	63.88	61.56	-0.208558098	0.957711	Protein similar to oxidoreductases; induced in high iron; Hap43-repressed
orf19.1486	orf19.1486	70.05	74.35	68.29	53.74	-0.192450173	0.998825	Protein with a life-span regulatory factor domain; regulated by Sef1, Sfu1, and Hap43; flow model biofilm induced; Spider biofilm induced
orf19.4771	orf19.4771	67.4	74.35	40.26	34.25	-0.672050284	0.140986	Protein of unknown function; Spider biofilm induced
orf19.1084	CDC39	65.35	74.3	67.56	66.74	-0.043258973	0.998825	Protein similar to S. cerevisiae Cdc39p, which is part of the CCR4-NOT transcription regulatory complex; transposon mutation affects filamentous growth
orf19.3548	orf19.3548	78.04	74.3	108.05	100.59	0.357061673	0.636502	Ortholog(s) have mitochondrion localization
orf19.7617	orf19.7617	74	74.28	81.7	74.68	0.055267908	0.998825	Ortholog(s) have peptide alpha-N-acetyltransferase activity, role in N-terminal protein amino acid acetylation, histone acetylation and NatA complex, cytosol, cytosolic ribosome, nucleus localization
orf19.6538	VMA11	70.57	74.13	102.19	81.08	0.249527961	0.892679	Predicted ortholog of S. cerevisiae Tfp3/Vma11; c' subunit of the V0 subcomplex of the vacuolar ATPase; required for hemoglobin-iron utilization; Spider biofilm repressed

orf19.1654	orf19.1654	91.23	74.08	79.49	87.33	0.000608248	0.998825	Predicted membrane protein; induced by prostaglandins
orf19.5104	LTP1	81.83	74.08	78.15	76.18	-0.023430756	0.998825	Putative protein phosphatase of the PTP family (tyrosine-specific), similar to S. cerevisiae Ltp1p
orf19.7658	RFC4	75.75	73.98	62.96	63.89	-0.192105403	0.995548	Putative heteropentameric replication factor C subunit; flucytosine induced; periodic mRNA expression, peak at cell-cycle G1/S phase
orf19.6639	orf19.6639	82.29	73.97	68.71	59.9	-0.219890235	0.946319	Ortholog of S. cerevisiae Mdm36; mitochondrial distribution and morphology protein; Hap43-repressed gene
orf19.5328	GCN1	67.63	73.86	78.46	85.91	0.150872415	0.998825	Ortholog(s) have role in regulation of translational elongation and cytosolic ribosome, extracellular region, mitochondrion localization
orf19.7149	orf19.7149	76.95	73.84	94.63	85.16	0.188952126	0.986892	Putative GTPase inhibitor; predicted role in endocytosis, protein targeting to vacuole; rat catheter biofilm repressed
orf19.1588	orf19.1588	74.33	73.83	79.8	77.4	0.055857413	0.998825	Putative mitochondrial protein of unknown function; regulated by Sef1p-, Sfu1p-, and Hap43p
orf19.4543	UGA2	84.7	73.72	97.21	86.85	0.157381729	0.998825	Predicted succinate semialdehyde dehydrogenase; predicted role in glutamate catabolism; transcription regulated by Mig1, Tup1, Gcn4; mutants are viable
orf19.1462	SMP2	73.43	73.72	103.06	86.69	0.279723976	0.853836	Putative Mg2+-dependent phosphatidate phosphatase; transcript regulated by Nrg1

orf19.4727	orf19.4727	90.48	73.62	95.27	105.3	0.221622535	0.931333	Ortholog(s) have succinate dehydrogenase (ubiquinone) activity
orf19.692	orf19.692	68.96	73.53	71.55	77.21	0.035656903	0.998825	Protein of unknown function; Hap43-repressed gene; rat catheter and Spider biofilm induced
orf19.4147	GLR1	71.64	73.41	89.4	77.04	0.140161218	0.998825	Glutathione reductase; upregulated by human neutrophils; oxidative stress-induced regulation via Cap1p; overexpression correlates with multidrug resistance in a cap1 mutant, farnesol induced; stationary phase enriched protein
orf19.597	orf19.597	88.64	73.38	89.39	74.93	0.004431795	0.998825	Protein with an aspartate aminotransferase domain; Gcn4-regulated
orf19.1782	orf19.1782	76.09	73.32	100.16	91.81	0.279238736	0.853836	Membrane-localized protein of unknown function
orf19.4633	orf19.4633	82.3	73.31	91.95	81.8	0.121430309	0.998825	Ortholog(s) have carbonyl reductase (NADPH) activity, oxidoreductase activity, acting on the aldehyde or oxo group of donors, NAD or NADP as acceptor activity and cytosol, nucleus, ribosome localization
orf19.577	orf19.577	97.86	73.18	80.93	69.25	-0.154816724	0.998825	Predicted protein tyrosine phosphatase; rat catheter biofilm induced
orf19.2107	MUQ1	77.22	73.17	113.85	109.61	0.454898147	0.384357	Putative choline phosphate cytidylyltransferase/phosphoethanolamine cytidylyltransferase; repressed in hyphae compared vs yeast; Hap43-repressed; flow model biofilm induced; Spider biofilm repressed
orf19.5100	MLT1	72.99	73.15	73.02	73.29	-0.00979059	0.998825	Vacuolar membrane transporter; MRP subfamily of ABC family; may transport organic anions conjugated to glutathione, glucuronate, or sulfate; needed for virulence in mouse peritonitis; Spider biofilm induced; flow model biofilm repressed

orf19.1702	ARF3	75.38	73.14	84.02	106.08	0.274770227	0.861125	Similar to but not orthologous to S. cerevisae Arf3; transcript filament induced; Tup1 regulated; rat catheter biofilm repressed (see Locus History Note for Assembly 19 correction)
orf19.5230	MRPS9	81.86	73.07	76.06	75.28	-0.037901831	0.998825	Mitochondrial ribosomal protein S9; has N-terminal mitochondrial targeting signal and an S9 consensus motif; overexpression in S. cerevisiae causes respiratory defect that is reversible upon cessation of C. albicans gene expression
orf19.1954	PUS4	95.67	73.02	70.43	63.95	-0.265168466	0.878538	Putative pseudouridine synthase; transcript regulated by Nrg1, Mig1, and Tup1
orf19.760	orf19.760	68.72	73.02	59.9	68.5	-0.113217253	0.998825	Ortholog(s) have role in ribosome biogenesis and mitochondrion localization
orf19.6188	orf19.6188	77.36	72.82	90.66	73.26	0.088428999	0.998825	Putative adhesin-like protein
orf19.7663	CSM1	83.54	72.75	73.15	60.69	-0.185392098	0.998825	Ortholog(s) have role in homologous chromosome segregation, protein localization to nucleolar rDNA repeats, rDNA condensation and monopolin complex, nuclear envelope, nucleolus localization
orf19.6672	MDJ1	75.59	72.64	72.35	64.97	-0.103327455	0.998825	Putative member of the HSP40 (DnaJ) family of chaperones; rat catheter and Spider biofilm induced
orf19.2117	LEU5	87.93	72.63	77.64	64.97	-0.141743617	0.998825	Putative mitochondrial carrier protein; Hap43-repressed; rat catheter biofilm induced
orf19.5903	RAX1	68.36	72.61	76.25	69.6	0.026999591	0.998825	Protein with a predicted role in bud site selection; hypha-induced expression; Spider biofilm induced

orf19.494	orf19.494	85.05	72.59	60.44	58.29	-0.328146041	0.786316	Putative RNA-binding protein; role in assembly of box H/ACA snoRNPs and thus pre-rRNA processing; Spider biofilm induced
orf19.4340.1	orf19.4340.1	66.35	72.59	70.86	71.34	0.012563827	0.998825	Ortholog(s) have role in mRNA splicing, via spliceosome and U1 snRNP, U2 snRNP, U4/U6 x U5 tri-snRNP complex, U5 snRNP, cytosol localization
orf19.3503	orf19.3503	54.61	72.56	41.18	53.77	-0.313020359	0.853836	Ortholog of Candida albicans WO-1 : CAWG_05160
orf19.4375	orf19.4375	75.33	72.41	72.24	65.94	-0.080110281	0.998825	Ortholog(s) have S-adenosylmethionine-dependent methyltransferase activity, role in chromatin silencing at rDNA, nicotinamide metabolic process and cytosol localization
orf19.490	NAT5	85.73	72.4	69.65	86.93	-0.012171991	0.998825	Subunit of the N-terminal acetyltransferase NatA; Spider biofilm repressed
orf19.1438	orf19.1438	89.68	72.36	3.36	1.53	-2.380806285	3.58E-14	Protein with homology to NADH dehydrogenase; regulated by Sef1p-, Sfu1p-, and Hap43p
orf19.1991	orf19.1991	80.73	72.35	79.77	75.46	0.005416478	0.998825	Ortholog(s) have cytoplasm localization
orf19.5507	ENP1	84.5	72.33	66.17	72	-0.149822066	0.998825	Protein required for pre-rRNA processing and 40S ribosomal subunit synthesis; associated with U3 and U14 snoRNAs; transposon mutation affects filamentous growth; repressed by prostaglandins; Spider biofilm induced
orf19.1460	orf19.1460	83.96	72.3	75.31	70.55	-0.082598325	0.998825	Putative glutamine-dependent NAD synthetase, involved in NAD salvage pathway

orf19.863	orf19.863	74.14	72.3	77.73	77.03	0.057222525	0.998825	Ortholog(s) have structural constituent of ribosome activity and mitochondrial large ribosomal subunit localization
orf19.3230	BOI2	68.8	72.27	66.43	62.18	-0.116440494	0.998825	Putative SH3-domain-containing protein; mutation confers hypersensitivity to toxic ergosterol analog; Spider biofilm induced
orf19.467	WOR3	64.83	72.2	159.09	161.89	0.994568502	0.000385	Transcription factor; modulator of white-opaque switch; induced in opaque cells; promoter bound by Wor1; overexpression at 25 degr shifts cells to opaque state; deletion stabilizes opaque cells at higher temperatures; Spider biofilm induced
orf19.4793	orf19.4793	77.69	72.18	61.08	53.02	-0.308279519	0.831485	Putative ribosome-associated protein; ortholog of S. cerevisiae Tma16; Hap43-induced gene; Spider biofilm induced
orf19.6478	YCF1	76.98	72.16	58.51	57.21	-0.281732053	0.869089	Putative glutathione S-conjugate transporter; MRP/CFTR-subfamily, ABC type transporter; human neutrophil-induced; oxidative stress-induced via Cap1; possible association with multidrug resistance; possibly essential; Spider biofilm induced
orf19.3534	RHO3	74.61	72.15	110.55	115.05	0.488321892	0.306039	Putative Rho family GTPase; possible substrate of protein farnesyltransferase and geranylgeranyltransferase type I; greater transcription in hyphal form than yeast form; plasma membrane-localized
orf19.505	SRV2	74.79	72.04	103.79	101.02	0.371982048	0.606662	Adenylate cyclase-associated protein; regulates adenylate cyclase activity; required for wild-type germ tube formation and for virulence in mice; mutant defects in filamentous growth are rescued by cAMP or dbcAMP; Spider biofilm repressed
orf19.5959	NOP14	94.95	72.02	71.35	58.88	-0.284812712	0.8593	Putative nucleolar protein; Hap43-induced; mutation confers resistance to 5-fluorocytosine (5-FC), 5-fluorouracil (5-FU), and tubercidin (7-deazaadenosine); heterozygous mutant is resistant to parnafungin; Spider biofilm induced
orf19.3582	orf19.3582	83.55	71.92	70.21	72.75	-0.104381999	0.998825	Ortholog(s) have protein-lysine N-methyltransferase activity, role in peptidyl-lysine dimethylation, vesicle-mediated transport and cytosol, nucleus localization

orf19.1619	orf19.1619	67.66	71.85	71.16	67.34	-0.026836772	0.998825	Putative kinase subunit of RNA polymerase II carboxy-terminal domain kinase I; possibly an essential gene, disruptants not obtained by UAU1 method
orf19.704	SOL3	82.84	71.82	116.73	98.16	0.367157409	0.608898	Putative 6-phosphogluconolactonase; present in exponential and stationary growth phase yeast cultures; macrophage-downregulated protein; Spider biofilm repressed
orf19.2471	GIM5	81.21	71.71	66.61	76.02	-0.081906652	0.998825	Putative heterohexameric cochaperone prefoldin complex subunit; macrophage/pseudohyphal-repressed gene and macrophage-induced protein
orf19.7437	orf19.7437	91.52	71.63	104.54	92.45	0.201905257	0.953167	Putative protein of unknown function; Hap43p-repressed gene; ortholog of S. cerevisiae YJL218W
orf19.7378	orf19.7378	79.81	71.61	91.4	74.62	0.08840431	0.998825	Ortholog(s) have phosphopantothenoylcysteine decarboxylase activity, protein phosphatase inhibitor activity and role in regulation of mitotic cell cycle, response to salt stress
orf19.6252	orf19.6252	77.58	71.48	75.66	71.28	-0.024493999	0.998825	Ortholog(s) have RNA polymerase II transcription factor binding transcription factor activity, core RNA polymerase II binding transcription factor activity
orf19.3558	orf19.3558	73.8	71.38	87.07	94.5	0.248736478	0.892679	Ortholog(s) have Golgi apparatus, endoplasmic reticulum localization
orf19.2343.1	orf19.2343.1	71.39	71.36	91.68	93.17	0.289957869	0.841359	Putative vacuolar H+ ATPase subunit e of the V-ATPase V0 subcomplex; added to Assembly 21 based on comparative genome analysis
orf19.4943	PSA2	61.41	71.29	69.63	60.27	-0.029499736	0.998825	Mannose-1-phosphate guanyltransferase; Hap43, macrophage-repressed; stationary phase enriched protein; Spider biofilm induced; rat catheter biofilm repressed

orf19.7457	orf19.7457	71.31	71.28	61.72	52.3	-0.248236426	0.919016	Protein with Mob2p-dependent hyphal regulation
orf19.7182	orf19.7182	68.85	71.19	64.91	68.4	-0.065640912	0.998825	Ortholog(s) have RNA binding activity, role in tRNA modification and cytoplasm, nucleus localization
orf19.3029	EHD3	86.15	71.19	72.13	66.81	-0.143598169	0.998825	Predicted 3-hydroxyisobutyryl-CoA hydrolase; mitochondrially localized; Spider biofilm induced
orf19.5131	orf19.5131	74.79	71.17	49.61	44.65	-0.466632034	0.470755	Ortholog of S. cerevisiae Gid7, a GID complex protein; involved in proteasome-dependent catabolite inactivation of fructose-1,6-bisphosphatase; Hap43-repressed gene
orf19.344	orf19.344	50.81	71.06	8.14	5.81	-1.665379556	8.49E-07	Protein of unknown function; upregulated by fluphenazine treatment or in an azole-resistant strain that overexpresses CDR1 and CDR2; transcript possibly regulated by Tac1
orf19.7291	orf19.7291	86.23	71.05	69.21	56.49	-0.259586859	0.892679	Ortholog(s) have tRNA (adenine-N1-)-methyltransferase activity, role in tRNA methylation and cytosol, nucleus, tRNA (m1A) methyltransferase complex localization
orf19.3583	orf19.3583	80.53	70.95	68.03	71.88	-0.09873568	0.998825	Ortholog(s) have role in protein export from nucleus, regulation of chromatin silencing at telomere, ribosomal small subunit export from nucleus and cytosol, nuclear pore localization
orf19.1970	orf19.1970	83.97	70.9	85.91	97.79	0.188314085	0.987362	Putative vacuole biogenesis protein
orf19.7224	orf19.7224	77.55	70.88	78.34	74.1	0.013001339	0.998825	Ortholog(s) have ubiquitin-protein ligase activity and role in histone catabolic process, histone ubiquitination

orf19.5156	orf19.5156	66.05	70.84	88.58	78.31	0.208813269	0.955592	Protein similar to S. cerevisiae Phs1p, which is required for growth; has six putative membrane-spanning regions
orf19.528	SEC26	75.86	70.75	95.89	90.39	0.257958699	0.878136	Secretory vesicles coatomer complex protein
orf19.6284	orf19.6284	79.36	70.68	76.88	83.22	0.06469317	0.998825	Ortholog(s) have GTP binding, signal recognition particle binding activity, role in protein targeting to ER and integral to endoplasmic reticulum membrane localization
orf19.3007.2	orf19.3007.2	70.12	70.68	109.94	74.5	0.288258765	0.853836	Ortholog of C. dubliniensis CD36 : Cd36_02950, C. parapsilosis CDC317 : CPAR2_108520, Candida tenuis NRRL Y-1498 : CANTEDRAFT_100285 and Debaryomyces hansenii CBS767 : DEHA2G15774g
orf19.4659	orf19.4659	77	70.68	59.85	62.89	-0.210379485	0.957711	Ortholog(s) have RNA binding activity, role in mRNA splicing, via spliceosome and U2 snRNP, U2-type prespliceosome localization
orf19.5723	POX1	77.58	70.67	65.34	65.84	-0.150210448	0.998825	Predicted acyl-CoA oxidase; regulated upon white-opaque switch; upregulated upon phagocytosis; Spider biofilm induced
orf19.1246	orf19.1246	79.28	70.62	101.38	84.82	0.233996125	0.918389	Putative eisosome component role in proper eisosome assembly; upregulated in cyr1 null mutant
orf19.1449	orf19.1449	76.72	70.58	59.88	52.1	-0.311878279	0.824505	Protein of unknown function; induced in azole-resistant strain that overexpresses MDR1; protein present in exponential and stationary growth phase yeast cultures; Spider biofilm induced
orf19.6249	HAK1	27.15	70.46	8.72	20.92	-0.865022115	0.057575	Putative potassium transporter; similar to Schwanniomyces occidentalis Hak1p; amphotericin B induced; induced upon phagocytosis by macrophage; Hap43-repressed; rat catheter biofilm repressed

orf19.5838	SER2	79.62	70.4	80.03	96.24	0.17366685	0.998825	Ortholog(s) have phosphoserine phosphatase activity, role in L-serine biosynthetic process and cytoplasm, nucleus localization
orf19.1885	TAF4	67.66	70.39	70.57	66.24	-0.018812204	0.998825	Putative TFIID subunit; mutation confers hypersensitivity to toxic ergosterol analog
orf19.876	PGA33	77.36	70.38	76.9	67.09	-0.033049844	0.998825	Putative GPI-anchored protein of unknown function; Spider biofilm repressed
orf19.7393	orf19.7393	80.21	70.38	87.8	87.86	0.172846738	0.998825	Predicted NAD+/NADH kinase; possible role in cellular iron ion homeostasis; Spider biofilm induced
orf19.5989	orf19.5989	86.72	70.37	84.01	74.08	-0.001504025	0.998825	Putative cleavage factor I subunit; required for the cleavage and polyadenylation of pre-mRNA 3' ends; Spider biofilm repressed
orf19.3393	orf19.3393	85.9	70.37	61.62	55.8	-0.313138894	0.820281	Putative DEAD-box helicase; Hap43-induced; Spider biofilm induced
orf19.772.1	orf19.772.1	49.85	70.35	36.07	48.72	-0.350802263	0.793335	Ortholog of C. parapsilosis CDC317 : CPAR2_105440, Lodderomyces elongisporus NRLL YB-4239 : LELG_01504, Pichia stipitis Pignal : psti_CGOB_00118 and Candida orthopsilosis Co 90-125 : CORT_0B06660
orf19.3705	PTC6	103.4	70.34	111.57	85.32	0.138955514	0.998825	Mitochondrial protein phosphatase of the Type 2C-related family (serine/threonine-specific), functional homolog of S. cerevisiae Ptc6p; mutant shows virulence defect
orf19.1902	NOC4	87.44	70.25	63.95	52.95	-0.329309301	0.788414	Putative nucleolar protein; Hap43-induced; mutation confers resistance to 5-fluorocytosine (5-FC), 5-fluorouracil (5-FU), and tubercidin (7-deazaadenosine); Spider biofilm induced

orf19.4907	orf19.4907	61.52	70.17	24.72	18.41	-1.067763905	0.003617	Putative protein of unknown function; Hap43p-repressed gene; increased transcription is observed upon fluphenazine treatment; possibly transcriptionally regulated by Tac1p; induced by nitric oxide; fungal-specific (no human/murine homolog
orf19.3990	orf19.3990	74.95	70.14	74.75	65.18	-0.049274346	0.998825	Ortholog(s) have DNA-directed RNA polymerase activity, RNA polymerase III activity and role in tRNA transcription from RNA polymerase III promoter, transcription initiation from RNA polymerase III promoter
orf19.4022	SDH4	59.41	70.08	55.3	51.33	-0.21888565	0.957711	Succinate dehydrogenase, membrane subunit; induced in high iron
orf19.7341	orf19.7341	73.11	70	99.2	103.37	0.385697484	0.578826	Protein of unknown function; flow model biofilm induced; ketoconazole-repressed
orf19.272	FAA21	76.12	70	82.07	83.39	0.128805391	0.998825	Predicted acyl CoA synthetase; upregulated upon phagocytosis; transcript regulated by Nrg1 and Mig1
orf19.4603	ARL1	68.96	69.98	67.04	67.7	-0.041692167	0.998825	Putative GTPase; mutation confers dose-dependent sensitivity to Brefeldin A
orf19.4966	AGC1	91.24	69.92	58.76	48.49	-0.449491594	0.49417	Putative mitochondrial carrier protein; transcript is alkaline upregulated rat catheter biofilm induced
orf19.1972	orf19.1972	80.67	69.89	63.06	62.89	-0.206626922	0.961899	Ortholog(s) have S-adenosylmethionine-dependent methyltransferase activity, role in histone lysine methylation and cytoplasm, nuclear chromatin localization
orf19.2937	PMM1	75.19	69.88	98.31	83.22	0.239989881	0.90951	Phosphomannomutase; enzyme of O- and N-linked mannosylation; interconverts mannose-6-phosphate and mannose-l-phosphate; functional homolog of S. cerevisiae Sec53; antigenic in mice; Hap43-induced; flow model and Spider biofilm repressed

orf19.7307	orf19.7307	76.53	69.87	79.63	71.43	0.019941066	0.998825	Putative oxidoreductase; similar to S. cerevisiae Pga3p; possible Kex2p substrate
orf19.4704	ARO1	66.15	69.86	69.78	74.65	0.060804062	0.998825	Putative pentafunctional arom enzyme; fungal-specific (no human or murine homolog); Gcn2p-, Gcn4p-regulated
orf19.2267	RFA2	69.42	69.86	62.52	63.42	-0.116689098	0.998825	Putative DNA replication factor A; RNA abundance regulated by cell cycle, tyrosol and cell density
orf19.4849	orf19.4849	69.04	69.78	55.66	56.2	-0.241892704	0.930538	Protein required for localizing proteasomes to the nucleus; involved in ubiquitin-mediated protein degradation; possibly an essential gene, disruptants not obtained by UAU1 method; Spider biofilm induced
orf19.6731	orf19.6731	54.48	69.76	56.78	67.12	-0.010617428	0.998825	Protein of unknown function; repressed by alpha pheromone in SpiderM medium; Spider biofilm induced
orf19.5541	orf19.5541	66.92	69.67	53.56	57.16	-0.234713336	0.938642	Protein with similarity to S. pombe Nrd1p; transcription induced upon induction of hyphal growth; regulated by Cph1p, Efg1p, Cph2p; low-level expression; alkaline upregulated; fungal-specific (no human or murine homolog)
orf19.4839	orf19.4839	67.99	69.66	79.18	81.88	0.162017483	0.998825	GET complex subunit; expression downregulated in an ssr1 null mutant
orf19.6463	orf19.6463	73.92	69.65	58.39	54.57	-0.271668494	0.880321	Putative ortholog of S. cerevisiae Npa3p; possibly essential for growth (however, depletion mutant is viable)
orf19.4263	orf19.4263	61.08	69.6	67.03	80.17	0.116048264	0.998825	Has domain(s) with predicted nucleotide binding activity

orf19.3947	orf19.3947	73.2	69.58	61.04	61.01	-0.182092334	0.998825	Ortholog(s) have rDNA binding activity
orf19.5174	TAF19	71.55	69.57	67.85	58.07	-0.141382286	0.998825	Putative TFIID subunit; mutation confers hypersensitivity to amphotericin B; rat catheter biofilm induced
orf19.5368	orf19.5368	60.6	69.56	67.68	70.52	0.053709403	0.998825	Ortholog of Vms1; component of a Cdc48-complex involved in protein quality control in S. cerevisiae; Spider biofilm repressed
orf19.1325	ECM38	90.53	69.53	35.63	25.84	-0.976269803	0.0069	Putative gamma-glutamyltransferase; alkaline upregulated; Spider biofilm induced; possibly an essential gene, disruptants not obtained by UAU1 method
orf19.851	orf19.851	84.42	69.52	43.41	30.68	-0.766021126	0.059153	Protein of unknown function; repressed by Rim101; negatively modulates intracellular ATP levels during the development of azole resistance; induced by Ca(2+) in a calcineurin-dependent manner; Spider biofilm induced
orf19.3026	MAS1	80.93	69.48	79.52	67.17	-0.032269107	0.998825	Putative mitochondrial processing protease; Hap43-repressed; transcript regulated by Nrg1; oxidative stress-induced via Cap1
orf19.3442	orf19.3442	86.98	69.47	75.62	75.44	-0.043531519	0.998825	Putative oxidoreductase; Hap43-repressed gene
orf19.3815	orf19.3815	67.58	69.43	67.4	64.02	-0.059205902	0.998825	Has domain(s) with predicted ubiquitin thiolesterase activity and role in ubiquitin-dependent protein catabolic process
orf19.4578	CYT2	69.69	69.41	63.96	54.12	-0.188093187	0.998825	Cytochrome c1 heme lyase; transcript regulated by Nrg1; induced in high iron

orf19.2791	BBC1	69.34	69.38	91.91	82.38	0.2493	0.894293	Putative SH3-domain-containing protein
orf19.3740	PGA23	80.95	69.3	114.63	87.38	0.327398436	0.742982	Putative GPI-anchored protein of unknown function; Rim101-repressed; Cyr1-regulated; colony morphology-related gene regulation by Ssn6
orf19.4760	orf19.4760	81.23	69.23	54.22	60.16	-0.303632376	0.842252	Putative protein-histidine N-methyltransferase; Spider biofilm induced
orf19.3704	orf19.3704	75.88	69.23	62.57	60.71	-0.179782216	0.998825	Ortholog(s) have role in maturation of 5.8S rRNA from tricistronic rRNA transcript (SSU-rRNA, 5.8S rRNA, LSU-rRNA), maturation of LSU-rRNA from tricistronic rRNA transcript (SSU-rRNA, 5.8S rRNA, LSU-rRNA)
orf19.3249	LAG1	70.88	69.19	80.18	90.84	0.21583323	0.946319	Putative ceramide synthase component; Hap43p-repressed gene; amphotericin B repressed
orf19.1236	GVP36	71.94	69.18	94.17	98.99	0.347867664	0.688747	BAR domain protein; ocalizes to early and late Golgi vesicles; predicted role in adaptation to varying nutrient concentrations, fluid-phase endocytosis, actin cytoskeleton polarization and vacuole biogenesis; rat catheter biofilm repressed
orf19.1483	orf19.1483	75.25	69.16	68.5	57.89	-0.147781936	0.998825	Ortholog(s) have copper ion binding activity, role in mitochondrial respiratory chain complex IV assembly and extrinsic to mitochondrial inner membrane, mitochondrial intermembrane space localization
orf19.1335	orf19.1335	77.78	69.11	65.67	60.98	-0.169401448	0.998825	Ortholog of S. cerevisiae Mtr4, an ATP-dependent 3'-5' RNA helicase of the DEAD-box family; Hap43-induced gene; Spider biofilm induced
orf19.2746	EMP70	66.28	69.09	70.1	66.46	-0.002754238	0.998825	Protein with a role in endosome-to-vacuole sorting; rat catheter biofilm repressed

orf19.6440	orf19.6440	68.96	69.02	68.78	65.91	-0.034256201	0.998825	Ortholog(s) have ubiquitin-protein ligase activity, role in ubiquitin-dependent protein catabolic process and endoplasmic reticulum membrane, nuclear inner membrane localization
orf19.6346	orf19.6346	76.24	68.98	72.92	69.37	-0.032377337	0.998825	Putative nuclear export protein; Hap43p-induced gene; decreased transcription is observed in an azole-resistant strain that overexpresses MDR1
orf19.610	EFG1	65.08	68.95	136.63	121.64	0.752725541	0.022275	bHLH transcription factor; required for white-phase cell type, RPMI and Spider biofilm formation, hyphal growth, cell-wall gene regulation; roles in adhesion, virulence; Cph1 and Efg1 have role in host cytokine response; binds E-box
orf19.639.1	orf19.639.1	62.87	68.91	59.68	79.29	0.048072897	0.998825	Ortholog(s) have structural constituent of ribosome activity and mitochondrial large ribosomal subunit localization
orf19.6757	GCY1	71.91	68.9	70.65	72.53	0.014355998	0.998825	Aldo/keto reductase; mutation confers hypersensitivity to toxic ergosterol analog; farnesol-repressed; stationary phase enriched protein; flow model biofilm induced; Spider biofilm repressed
orf19.4732	SEC24	67.54	68.84	120.88	120.09	0.643234003	0.08253	Protein with a possible role in ER to Golgi transport; induced upon yeast-hyphal switch; sumoylation target; Spider biofilm repressed
orf19.2862	RIB1	62.43	68.82	72.86	66.98	0.060651287	0.998825	Putative GTP cyclohydrolase II; enzyme of riboflavin biosynthesis; fungal-specific (no human or murine homolog); regulated by Sef1p-, Sfu1p-, and Hap43p
orf19.7546	orf19.7546	80.53	68.8	55.59	63.76	-0.248771955	0.916335	Protein involved in rRNA processing; required for maturation of the 35S primary transcript of pre-rRNA and for cleavage leading to mature 18S rRNA; Spider biofilm induced
orf19.2176	IFM3	58.49	68.8	34.2	32.31	-0.660782645	0.172872	Protein with a 2-hydroxyacid dehydrogenase catalytic domain; Hap43-repressed; Plc1-regulated; overlaps orf19.2177

orf19.1852	orf19.1852	79.93	68.76	83.8	91.17	0.174454573	0.998825	Ortholog(s) have mitochondrion localization
orf19.2371	orf19.2371	63.77	68.7	60.24	72.45	-0.016584759	0.998825	Putative Gag protein of retrotransposon Tca2; separated by a stop codon from Pol protein orf19.2372; likely translated as single polyprotein that includes Gag, reverse transcriptase, protease, and integrase; rat catheter biofilm repressed
orf19.7500	PXA1	82.62	68.66	83.99	66.23	-0.024944119	0.998825	Putative peroxisomal, half-size adrenoleukodystrophy protein (ALD or ALDp) subfamily ABC family transporter
orf19.7581	orf19.7581	72.19	68.61	73.05	90.88	0.16084663	0.998825	Protein with a predicted role in assembly of U2 snRNP into the spliceosome; Spider biofilm induced
orf19.7080	LEU2	62.44	68.61	3.67	5.01	-1.953884251	2.09E-09	Isopropyl malate dehydrogenase; leucine biosynthesis; induced by human whole blood or PMNs; protein level decreases in stationary phase; GlcNAc-induced protein; flow model biofilm repressed
orf19.5160	orf19.5160	74.36	68.56	74.94	70.07	0.005707799	0.998825	Ortholog(s) have role in G1/S transition of mitotic cell cycle, TOR signaling cascade, dephosphorylation, regulation of mitotic sister chromatid segregation, tRNA wobble uridine modification
orf19.2103	orf19.2103	69.89	68.54	65.29	51.59	-0.197296514	0.992876	Ortholog of Candida albicans WO-1 : CAWG_03811
orf19.147	YAK1	62.42	68.47	56.42	54.35	-0.188381703	0.998825	Predicted serine-threonine protein kinase; involved in hyphal growth regulation and biofilm formation; flow model biofilm induced; induced in core caspofungin response
orf19.1667.1	orf19.1667.1	76.34	68.45	69.07	83.94	0.060871191	0.998825	Ortholog(s) have role in mitochondrial respiratory chain complex IV assembly and mitochondrial intermembrane space, nucleus localization

orf19.947	MRP17	81.92	68.43	64.36	63.21	-0.190127454	0.998825	Predicted mitochondrial ribosomal protein
orf19.559	FGR14	65.8	68.38	66.42	69.42	-0.001382457	0.998825	Protein encoded in retrotransposon Zorro3 with similarity to retroviral endonuclease-reverse transcriptase proteins; lacks an ortholog in S. cerevisiae; transposon mutation affects filamentous growth
orf19.2452	orf19.2452	67.8	68.38	74.05	76.71	0.106425577	0.998825	Protein of unknown function; induced in high iron; repressed in core caspofungin response; ketoconazole-repressed; colony morphology-related gene regulation by Ssn6; possibly subject to Kex2 processing
orf19.1847	ARO10	75.6	68.32	52.91	26.2	-0.612602429	0.237824	Aromatic decarboxylase; Ehrlich fusel oil pathway of aromatic alcohol biosynthesis; alkaline repressed; protein abundance affected by URA3 expression in CAI-4 strain; Spider biofilm induced
orf19.7036	orf19.7036	73.74	68.32	64.08	76.71	-0.014332367	0.998825	Ortholog(s) have phosphatase activator activity, role in actin filament organization, endocytosis, mitochondrion degradation, regulation of growth, response to heat, response to salt stress and cytosol, nucleus localization
orf19.517	HAP31	84.46	68.31	75.23	84.83	0.051529659	0.998825	Putative CCAAT-binding transcription factor; regulates CYC1; required for resistance to rapamycin, copper; induced in high iron; repressed in low iron; Sef1, Sfu1, and Hap43 regulated; rat catheter and Spider biofilm induced
orf19.5811	MET1	78.04	68.3	120.13	97.47	0.44393628	0.423394	Putative uroporphyrin-3 C-methyltransferase, methionine biosynthesis enzyme; upregulated by human whole blood or polymorphonuclear (PMN) cells; Hog1p-induced; fungal-specific (no human or murine homolog); Hap43p-repressed
orf19.412	SSH1	69.67	68.28	66.63	72.79	0.007416289	0.998825	Protein with a role in protein translocation across membranes
orf19.1352	TIM22	81.27	68.28	81.05	82.41	0.093269546	0.998825	Mitochondrial inner membrane protein; predicted role in protein import; Hap43-repressed gene; flow model biofilm induced; Spider biofilm repressed

orf19.7539.1	CPY1	72.91	68.25	76.42	86.74	0.154192202	0.998825	Putative adhesin-like protein; transcription detected in high-resolution tiling array experiments
orf19.3309	orf19.3309	78.15	68.23	80.19	87.77	0.150460483	0.998825	Ortholog of Candida tropicalis MYA-3404 : CTRG_04422 and Candida albicans WO-1 : CAWG_01254
orf19.1683	PPH21	79.18	68.22	91.61	83.78	0.194492771	0.976613	Putative protein phosphatase of the Type 2A-related family (serine/threonine-specific), similar to S. cerevisiae Pph21p; caspofungin repressed; possibly an essential gene, disruptants not obtained by UAU1 method
orf19.3357	orf19.3357	69.37	68.2	94.65	80.55	0.269840769	0.87024	Ortholog(s) have mitochondrion localization
orf19.5847	orf19.5847	73.77	68.09	60.07	54.56	-0.237577503	0.932191	Ortholog(s) have RNA polymerase III activity, role in tRNA transcription from RNA polymerase III promoter and DNA-directed RNA polymerase III complex, cytosol localization
orf19.6121	MNL1	65.62	68.03	61.79	52.3	-0.18473712	0.998825	Transcription factor; induces transcripts of stress response genes via SLE (STRE-like) elements; required for adaptation to weak acid stress; activates a subset of the genes that are repressed by Nrg1
orf19.7128	SYS1	79.9	67.91	82.77	68.1	0.012264718	0.998825	Putative Golgi integral membrane protein; transcript regulated by Mig1
orf19.6503	orf19.6503	84.07	67.83	81.72	87.64	0.119578058	0.998825	Ortholog(s) have structural constituent of ribosome activity, role in mitochondrial translation and mitochondrial large ribosomal subunit localization
orf19.3187	ZNC1	71.91	67.83	63.23	58.88	-0.159207504	0.998825	Zn(2)-Cys(6) transcription factor; regulated by Gcn2 and Gcn4; gene located in zinc cluster region of Chromosome 5, near the MTL locus; required for yeast cell adherence to silicone substrate; Spider biofilm induced

orf19.7458	orf19.7458	63.36	67.74	65.38	68.1	0.00645221	0.998825	Ortholog of C. dubliniensis CD36 : Cd36_86660, Candida tenuis NRRL Y-1498 : cten_CGOB_00028, Candida tropicalis MYA-3404 : CTRG_05695 and Candida albicans WO-1 : CAWG_02979
orf19.1544	orf19.1544	58.22	67.73	88.28	64.21	0.188110926	0.998825	Putative cis-golgi localized protein involved in ER to Golgi transport; Spider biofilm repressed
orf19.4377	KRE1	87.08	67.71	51.8	45.69	-0.49751804	0.39542	Cell wall glycoprotein; beta glucan synthesis; increases glucan content in S. cerevisiae kre1, complements killer toxin sensitivity; caspofungin induced; Spider/rat catheter/flow model biofilm induced; Bcr1-repressed in RPMI a/a biofilms
orf19.7277	orf19.7277	67.62	67.6	78.13	69.62	0.08246045	0.998825	Predicted ORF in retrotransposon Zorro2 with similarity to zinc finger-containing retroviral nucleocapsid proteins; un-merged from orf19.7275 in a revision of Assembly 21 based on comparative genome analysis and new sequence data
orf19.575	HYR3	58.3	67.58	74.3	72.61	0.156324851	0.998825	Putative GPI-anchored adhesin-like protein; similar to Hyr1p induced in high iron; clade-specific repeat variation
orf19.2708	orf19.2708	75.25	67.53	57.47	57.44	-0.253424407	0.90951	Ortholog(s) have guanyl-nucleotide exchange factor activity, role in mature ribosome assembly and cytosol, nucleolus, polysome, preribosome, large subunit precursor localization
orf19.4362	orf19.4362	74.14	67.52	65.67	58.38	-0.157678281	0.998825	Ortholog(s) have role in protein targeting to mitochondrion and mitochondrial outer membrane localization
orf19.6233	orf19.6233	71.11	67.42	69.46	57.85	-0.101141915	0.998825	Ortholog(s) have endoplasmic reticulum, nuclear envelope localization
orf19.6588	NBP2	62.91	67.4	57.77	59.08	-0.122800548	0.998825	Protein containing an SH3 domain; involved in vacuolar fusion in hyphae; mutants form multiple germ tubes; Spider biofilm induced

orf19.3951	TIP1	74.74	67.39	84.36	84.82	0.187224037	0.995201	Protein with a predicted role in biogenesis of ER-derived COPII transport vesicles; mutation confers hypersensitivity to toxic ergosterol analog
orf19.3973	orf19.3973	69.81	67.36	60.68	52.68	-0.208115832	0.971803	Ortholog of Hua1, a zinc finger domain protein with sequence similarity to Type I J-proteins; flow model biofilm induced
orf19.4681	RAT1	74.85	67.35	62.14	59.08	-0.1827796	0.998825	5'-->3' exoribonuclease; similar to S. cerevisiae nuclear exoribonuclease Rat1p; suppresses S. cerevisiae kem1 mutant slow growth, mating defect, and haploid invasive growth defect
orf19.738	MYO5	66.87	67.31	76.79	63.19	0.036051565	0.998825	Class I myosin; nonessential; role in cortical actin patch polarity and polar budding; required for hyphal growth, white-opaque switch; regulatory phosphorylation on S366; downregulated on adherence to polystyrene
orf19.633	CRG1	78.86	67.22	49.68	53.79	-0.369647675	0.692027	Methyltransferase involved in sphingolipid homeostasis, methylates a drug cantharidin; decreased expression in hyphae compared to yeast; expression regulated during planktonic growth; flow model biofilm induced; Hap43-repressed gene
orf19.2385	KTI12	75.26	67.14	58.04	58.01	-0.227047487	0.944705	Protein similar to S. cerevisiae Kti12p, which associates with Elongator complex; has a role in resistance to killer toxin; predicted Kex2p substrate; Hap43p-induced gene
orf19.2706	CRH11	58	67.02	66.18	76.95	0.135805929	0.998825	GPI-anchored cell wall transglycosylase, putative ortholog of S. cerevisiae Crh1p; predicted glycosyl hydrolase domain; similar to Csf4p and to antigenic A. fumigatus Aspf9; predicted Kex2p substrate; caspofungin-induced
orf19.1115	GUK1	67.37	67.01	46.6	52.65	-0.316660374	0.833355	Putative guanylate kinase; identified in extracts from biofilm and planktonic cells; protein level decrease in stationary phase cultures; Hap43p-induced gene
orf19.3480	orf19.3480	71.39	67	57.11	56.31	-0.2241184	0.946319	Ortholog(s) have structural constituent of ribosome activity and mitochondrial small ribosomal subunit localization

orf19.4088	GLO2	75.46	66.94	90.54	98.02	0.316377518	0.780641	Ortholog(s) have hydroxyacylglutathione hydrolase activity, role in cellular carbohydrate metabolic process, methylglyoxal catabolic process to D-lactate and cytosol, mitochondrion, nucleus localization
orf19.1868	RNR22	55.7	66.91	44.03	47.32	-0.319736201	0.842252	Putative ribonucleoside diphosphate reductase;colony morphology-related gene regulation by Ssn6; transcript regulated by tyrosol and cell density; Hap43-repressed; Spider biofilm induced
orf19.329	orf19.329	64.15	66.86	69.29	57.28	-0.054099579	0.998825	Putative pre-mRNA branch point binding protein; role in mRNA splicing via spliceosome; Spider biofilm repressed
orf19.1403	orf19.1403	70.65	66.84	67.87	70.64	-0.000580817	0.998825	Ortholog(s) have ATP:ADP antiporter activity, calcium ion binding activity, role in ADP transport, ATP transport, mitochondrial transport and mitochondrion localization
orf19.371	orf19.371	65.74	66.84	84.97	78.69	0.222551079	0.938642	Ortholog of C. dubliniensis CD36 : Cd36_40110, C. parapsilosis CDC317 : CPAR2_402300, Candida tenuis NRRL Y-1498 : CANTEDRAFT_94507 and Debaryomyces hansenii CBS767 : DEHA2F05588g
orf19.3952	SRP101	77.83	66.82	86.39	74.82	0.10759341	0.998825	Signal recognition particle (SRP) receptor alpha subunit; involved in SRP-dependent protein targeting; rat catheter biofilm repressed
orf19.2454	PHO87	71.56	66.79	55.09	42.8	-0.380077475	0.680517	Putative phosphate permease; transcript repressed by Rim101 at pH 8; regulated by white-opaque switch; caspofungin repressed; virulence-group-correlated expression; flow model biofilm induced
orf19.6923	orf19.6923	66.62	66.78	66.99	73.87	0.047185306	0.998825	Ortholog(s) have chromatin binding activity, role in RNA polymerase II transcriptional preinitiation complex assembly and transcription factor TFIID complex localization
orf19.2752	ADR1	79.51	66.78	78.82	60.28	-0.071979774	0.998825	C2H2 transcription factor; ortholog of S. cerevisiae Adr1 but mutant phenotype suggests a different set of target genes; transposon mutation affects filamentous growth; Spider biofilm induced

orf19.4255	ECM331	67.23	66.76	109.75	101.58	0.51325003	0.273277	GPI-anchored protein; mainly at plasma membrane, also at cell wall; Hap43, caspofungin-induced; Plc1-regulated; Hog1, Rim101-repressed; colony morphology-related regulated by Ssn6; induced by ketoconazole and hypoxia
orf19.1440.2	orf19.1440.2	66.79	66.76	48.1	50.55	-0.328132476	0.811201	Ortholog of C. dubliniensis CD36 : Cd36_62310
orf19.4933	FAD3	97.8	66.75	55.28	38.79	-0.599943019	0.218547	Omega-3 fatty acid desaturase; production of alpha-linolenic acid, a major component of membranes; caspofungin induced; Plc1-regulated; colony morphology-related gene regulation by Ssn6; Spider biofilm induced, flow model biofilm repressed
orf19.2204	orf19.2204	75.71	66.69	67.72	61.25	-0.122099188	0.998825	Predicted membrane protein of unknown function; Spider biofilm induced
orf19.6842	TUS1	54.83	66.65	61.14	55.61	-0.056745611	0.998825	Putative guanine nucleotide exchange factor; induced by Mnl1 under weak acid stress; Spider biofilm induced
orf19.5607	orf19.5607	68.43	66.6	49.43	49.81	-0.336872905	0.789669	Ortholog of C. dubliniensis CD36 : Cd36_63860, C. parapsilosis CDC317 : CPAR2_601140, Candida tenuis NRRL Y-1498 : CANTEDRAFT_92714 and Debaryomyces hansenii CBS767 : DEHA2A06072g
orf19.6578	orf19.6578	73.15	66.57	76.11	67.06	0.013311189	0.998825	Predicted membrane transporter; vesicular neurotransmitter (VNT) family, major facilitator superfamily (MFS); repressed in core caspofungin response; induced in oralpharyngeal candidasis; Spider biofilm induced
orf19.4593	RGA2	64.14	66.56	72.84	69.28	0.077479629	0.998825	Putative GTPase-activating protein (GAP) for Rho-type GTPase Cdc42; involved in cell signaling pathways controlling cell polarity; induced by low-level peroxide stress; flow model biofilm induced
orf19.7323	CBP1	73.66	66.54	69.92	54.97	-0.141704705	0.998825	Corticosteroid binding protein; transcription induced at late log-phase or upon adherence to polystyrene; not induced by corticosterone; contains a possible NAD/FAD binding region; regulated by Nrg1, Tup1; Spider biofilm induced

orf19.3827	orf19.3827	76.64	66.54	88.71	77.32	0.149669109	0.998825	Ortholog(s) have metalloendopeptidase activity, role in misfolded or incompletely synthesized protein catabolic process and mitochondrial inner membrane localization
orf19.3287	BUD22	81.7	66.53	62.46	54.61	-0.270933328	0.880321	Protein with a predicted role in 18S rRNA maturation and small ribosomal subunit biogenesis; repressed in core stress response; repressed by prostaglandins
orf19.2156	NAG1	66.74	66.46	57.89	60.15	-0.13769415	0.998825	Glucosamine-6-phosphate deaminase; required for normal hyphal growth and mouse virulence; converts glucosamine 6-P to fructose 6-P; reversible reaction in vitro; gene and protein is GlcNAc-induced; Spider biofilm induced
orf19.5654	STE24	72.19	66.44	88.99	70.85	0.15302275	0.998825	Prenyl-dependent protease
orf19.3392	DOG1	62.72	66.4	82.08	96.02	0.349427035	0.711296	Putative 2-deoxyglucose-6-phosphatase; haloacid dehalogenase hydrolase/phosphatase superfamily; similar to S. cerevisiae Dog1, Dog2, Hor1, Rhr2; regulated by Nrg1, Tup1; Spider biofilm repressed
orf19.2966	orf19.2966	68.9	66.36	128.4	135.86	0.771469551	0.016769	Predicted dienelactone hydrolase domain; clade-associated gene expression; farnesol-downregulated; rat catheter biofilm repressed
orf19.2650.1	orf19.2650.1	77.19	66.35	68.35	64	-0.096279869	0.998825	Mitochondrial ribosomal protein of the small subunit; Spider biofilm repressed
orf19.7216	YPT52	75.03	66.35	89.79	74.22	0.158229121	0.998825	Rab-family GTPase involved in vacuolar trafficking, colocolizes with Vps1p and Ypt53p in late endosome
orf19.2131	orf19.2131	77.24	66.28	72.92	66.02	-0.040242193	0.998825	Ortholog(s) have ubiquitin-protein ligase activity and role in protein ubiquitination, ubiquitin-dependent protein catabolic process via the multivesicular body sorting pathway

orf19.216	orf19.216	63.89	66.19	52.78	44.24	-0.318213383	0.833355	Protein with a metallo-dependent phosphatase domain; ketoconazole-induced; upregulation correlates with clinical development of fluconazole resistance
orf19.7197	orf19.7197	90.24	66.19	69.08	60.49	-0.215411723	0.951606	Putative intranuclear transport and DNA replication mediator; heterozygous null mutant exhibits resistance to parnafungin in the C. albicans fitness test; Spider biofilm induced
orf19.1121	orf19.1121	76.1	66.13	52.13	42.66	-0.434201027	0.55304	Has domain(s) with predicted hydrolase activity
orf19.3363	VTC4	70.33	66.05	73.86	63.18	-0.002699025	0.998825	Putative polyphosphate synthetase; decreased expression in hyphae compared to yeast-form cells; fungal-specific (no human or murine homolog); virulence-group-correlated expression
orf19.1923	RRN3	73.39	66.03	62.99	62.63	-0.115354092	0.998825	Protein with a predicted role in recruitment of RNA polymerase I to rDNA; caspofungin induced; flucytosine repressed; repressed in core stress response; repressed by prostaglandins
orf19.2093	RFA1	69.3	66.01	102.14	90.86	0.394282809	0.568429	Putative DNA replication factor A; RNA abundance regulated by cell cycle, tyrosol and cell density
orf19.6448	orf19.6448	67.13	65.99	81.07	70.08	0.12891921	0.998825	Protein of unknown function; Spider biofilm repressed
orf19.6031	VPS27	69.95	65.98	74.49	66.74	0.029617038	0.998825	Putative ESCRT-0 complex protein with a role in multivesicular body (MVB) trafficking
orf19.2820	orf19.2820	71.97	65.94	75.2	62.65	-0.010547388	0.998825	Ortholog(s) have nucleocytoplasmic transporter activity, structural constituent of nuclear pore activity

orf19.7624	orf19.7624	67.94	65.94	59.39	50.42	-0.231076337	0.943903	Ortholog(s) have role in rRNA processing and 90S preribosome, nucleolus localization
orf19.6544	LPI9	66.51	65.86	62.45	59.11	-0.111978919	0.998825	Protein phosphatase type 1 regulator; role in chromosome segregation, regulation of phosphoprotein phosphatase activity; rat catheter and Spider biofilm induced
orf19.4026	HIS1	64.19	65.86	22.53	24.21	-0.982805408	0.009188	ATP phosphoribosyl transferase; enzyme of histidine biosynthesis; acid upregulated/alkaline repressed by Rim101; regulated by Gcn2, Gcn4; strain CA9 is a his1 mutant; flow model biofilm induced; Spider biofilm repressed
orf19.2262	orf19.2262	65.64	65.78	63.03	62.68	-0.059938037	0.998825	Protein similar to quinone oxidoreductases; induced by benomyl treatment, nitric oxide; oxidative stress-induced via Cap1; stationary-phase enriched protein; Spider biofilm induced
orf19.4597	orf19.4597	72.94	65.77	73.82	76.37	0.076159492	0.998825	Putative F-actin-capping protein subunit beta; possibly an essential gene, disruptants not obtained by UAU1 method
orf19.432	orf19.432	75.65	65.73	57.06	54.33	-0.273603527	0.880321	Predicted SCF ubiquitin ligase complex protein; Spider biofilm induced; rat catheter biofilm induced
orf19.6816	orf19.6816	72.23	65.72	130.69	120.82	0.690345557	0.047229	Putative xylose and arabinose reductase; flow model biofilm induced; Spider biofilm repressed
orf19.6077	orf19.6077	68.61	65.72	66.04	49.45	-0.183243868	0.998825	Putative protein of unknown function; shows colony morphology-related gene regulation by Ssn6p
orf19.3555	BUD14	59.37	65.72	71.46	63.7	0.071205476	0.998825	Putative SH3-domain-containing protein; predicted role in bud-site selection; Spider biofilm induced

orf19.542.2	MIM1	63.17	65.71	58.39	57.85	-0.123587206	0.998825	Predicted mitochondrial protein involved in outer membrane protein import; rat catheter biofilm repressed
orf19.89	PEX7	78.83	65.67	78.95	80.22	0.095499148	0.998825	Ortholog(s) have peroxisome matrix targeting signal-2 binding activity, role in fatty acid metabolic process, protein import into peroxisome matrix, docking and cytosol, nucleus, peroxisome localization
orf19.837.1	INO4	65.18	65.67	80.25	67.52	0.122390177	0.998825	Transcription factor; activator that forms a heterodimer with Ino2; likely regulates genes involved in phosphatidylcholine and phosphatidylinositol biosynthesis, fatty acid beta-oxidation, and peroxisome biogenesis; gene has intron
orf19.2574	orf19.2574	72.92	65.59	66.18	63.65	-0.081937875	0.998825	Ortholog(s) have Golgi apparatus localization
orf19.4446	orf19.4446	73.28	65.58	62.56	66.04	-0.089356027	0.998825	Protein similar to ammomium permeases that is probably inactive, as a mep2 mep3 double homozygous null mutation abolishes cellular ammomium permease activity; fungal-specific (no human or murine homolog)
orf19.1191	orf19.1191	71.12	65.53	65.23	62.92	-0.083139843	0.998825	Ortholog(s) have ubiquitin-protein ligase activity and role in ER-associated ubiquitin-dependent protein catabolic process
orf19.3304	orf19.3304	76.28	65.49	65.57	61.58	-0.11404067	0.998825	Exosome non-catalytic core component; involved in 3'-5' RNA processing and degradation in the nucleus and cytoplasm; Spider biofilm induced
orf19.726	PPZ1	75.4	65.47	46.71	46.17	-0.439045088	0.543746	Protein phosphatase Z; type 1 family serine/threonine specific protein phosphatase involved in cation homeostasis and cell wall integrity
orf19.6979	orf19.6979	59.5	65.46	56.59	57.35	-0.099685077	0.998825	Ortholog(s) have role in cellular manganese ion homeostasis, mitochondrion organization and fungal-type vacuole membrane localization

orf19.429	orf19.429	72.27	65.39	62.89	58.16	-0.143728408	0.998825	Putative non-canonical poly(A) polymerase; repressed by nitric oxide; Spider biofilm induced
orf19.3746	OPT2	61.16	65.37	41.09	41.91	-0.437310136	0.568429	Oligopeptide transporter; induced upon phagocytosis by macrophage; macrophage/pseudohyphal-repressed after 16h; fluconazole-induced; virulence-group-correlated expression; Hap43-repressed
orf19.1186	orf19.1186	65.03	65.28	72.31	73.93	0.118034201	0.998825	Has domain(s) with predicted role in attachment of GPI anchor to protein and GPI-anchor transamidase complex localization
orf19.1254	SEC23	63.59	65.18	102.77	101.43	0.509598069	0.291165	Putative GTPase-activating protein; regulated upon yeast-hypha switch; Spider biofilm repressed
orf19.3722	orf19.3722	58.94	65.13	44.07	43.61	-0.36305536	0.742982	Predicted protein of rapamycin resistance; Spider biofilm induced
orf19.5098	NTG1	70.21	65.09	67.68	70.97	0.023707086	0.998825	Protein with strong similarity to both S. cerevisiae Ntg1 and Ntg2 DNA repair glycosylases; transcript induced by iron; Hap43-repressed gene; Spider biofilm induced
orf19.5168	orf19.5168	69.91	65.02	62.15	55.98	-0.154866313	0.998825	Ortholog(s) have role in maturation of SSU-rRNA and cytosol, nucleolus localization
orf19.1791	orf19.1791	74.74	65	59.18	58.52	-0.19276565	0.998825	Putative protein with a predicted role in 60S ribosomal subunit biogenesis; Hap43p-induced gene; ortholog of S. cerevisiae MAK11
orf19.1422	FZO1	64.11	65	60.16	56.7	-0.114813025	0.998825	Mitochondrial biogenesis protein; rat catheter and Spider biofilm induced

orf19.730	orf19.730	65.14	64.94	65.7	62.46	-0.027034593	0.998825	Ortholog(s) have Rho GTPase activator activity and role in establishment or maintenance of actin cytoskeleton polarity, small GTPase mediated signal transduction
orf19.4012	PCL5	73.01	64.92	55.08	47.21	-0.330804861	0.795378	Putative cyclin for Pho85 kinase; Gcn4-induced; suppresses toxicity of C. albicans Gcn4 overproduction in S. cerevisiae via increased Pho85-dependent phosphorylation and degradation of Gcn4; rat catheter and Spider biofilm induced
orf19.954	orf19.954	64.99	64.83	63.97	59.84	-0.06123857	0.998825	Putative DnaJ-like chaperone; Hap43-repressed gene
orf19.2857	orf19.2857	72.62	64.8	61.6	55.83	-0.178423659	0.998825	Ortholog(s) have ATP-dependent DNA helicase activity
orf19.1950	orf19.1950	63.94	64.76	83.78	73.74	0.212834583	0.951606	Ortholog(s) have phosphatase activity
orf19.4615	orf19.4615	66.72	64.72	72.25	66.31	0.03838517	0.998825	Ortholog(s) have histone deacetylase activity
orf19.73	orf19.73	63.31	64.71	52.45	58.26	-0.169158601	0.998825	Putative metalloprotease; associates with ribosomes and is involved in ribosome biogenesis; Spider biofilm induced
orf19.3006	GGA2	66.1	64.7	80.27	72.74	0.160920054	0.998825	Protein involved in Golgi trafficking; rat catheter and Spider biofilm repressed
orf19.3467	SEC27	64.26	64.63	84.01	74.4	0.212834583	0.951606	Ortholog(s) have ubiquitin binding activity

orf19.2089	orf19.2089	68.01	64.62	65.36	66	-0.025394995	0.998825	Ortholog of Nyv1, v-SNARE component of the vacuolar SNARE complex involved in vesicle fusion in S. cerevisiae; Hap43-repressed gene
orf19.6156	orf19.6156	73.98	64.51	67.5	46.68	-0.222856663	0.951606	Ortholog of S. cerevisiae : AIM11, C. glabrata CBS138 : CAGL0I04928g, C. dubliniensis CD36 : Cd36_80770, C. parapsilosis CDC317 : CPAR2_102260 and Candida tenuis NRRL Y-1498 : CANTEDRAFT_114262
orf19.3167	orf19.3167	75.28	64.48	79.91	64.84	0.036407558	0.998825	Heme A:farnesyltransferase; catalyzes the 1st step in conversion of protoheme to the heme A prosthetic group required for cytochrome c oxidase activity; Spider biofilm repressed
orf19.5617	orf19.5617	74.85	64.46	69.74	65.31	-0.040578177	0.998825	Ortholog(s) have methylated histone residue binding, transcription factor binding activity
orf19.4666	orf19.4666	69.33	64.46	58.5	47.84	-0.241707373	0.932191	Protein of unknown function; hyphal-induced expression, regulated by Cyr1, Ras1, Efg1; Spider biofilm induced
orf19.3293	orf19.3293	65.43	64.4	94.37	100.15	0.451084529	0.432569	Putative enoyl reductase involved in very long chain fatty acid elongation; possibly an essential gene, disruptants not obtained by UAU1 method
orf19.3921	orf19.3921	67.51	64.37	62.22	68.18	-0.024402219	0.998825	Ortholog(s) have cell division site, cell tip, cytosol, nucleus localization
orf19.35	orf19.35	78.94	64.35	61.88	49.64	-0.271316141	0.883681	Predicted kinase; rat catheter, flow model, Spider biofilm induced
orf19.5352	orf19.5352	80.46	64.31	90.4	66.84	0.085144142	0.998825	Protein with a predicted magnesium transporter domain; mutants are viable

orf19.6903	orf19.6903	71.72	64.21	57.5	59.5	-0.178917928	0.998825	Predicted RNA polymerase III subunit C37; Spider biofilm induced
orf19.671	PSP1	70.46	64.17	71	56.09	-0.069058488	0.998825	Protein repressed during the mating process
orf19.7657	POP3	69.81	64.16	43.61	44.86	-0.43497319	0.561982	Putative RNase MRP and nuclear RNase P component; decreased repressed by prostaglandins; Spider biofilm induced
orf19.5952	orf19.5952	63.01	63.98	49.4	64.94	-0.120101062	0.998825	Protein of unknown function; induced by nitric oxide independent of Yhb1; Sef1, Sfu1, and Hap43-induced; rat catheter and Spider biofilm induced
orf19.4546	HOL4	73.16	63.94	67.93	66.32	-0.032631423	0.998825	Putative ion transporter; alkaline induced by Rim101; Plc1-regulated; caspofungin repressed; rat catheter and Spider biofilm induced
orf19.2204.2	RHO2	75.41	63.68	116.07	122.53	0.618520357	0.106451	Ortholog(s) have GTPase activity
orf19.4384.1	orf19.4384.1	45.82	63.57	125.17	124.73	0.908940144	0.004996	Putative MFS transporter; predicted ORF in Assembly 20; removed from Assembly 21; restored based on conservation among several Candida species
orf19.4600	orf19.4600	110.38	63.55	96.05	80.37	0.007647795	0.998825	Protein of unknown function; possible mitochondrial protein; Spider biofilm induced
orf19.6846	PHO85	73.25	63.41	72.29	72.07	0.054988724	0.998825	Functional homolog of S. cerevisiae Pho85p, a cyclin-dependent kinase that regulates transcription of PHO genes involved in phosphate metabolism; necessary for geldanamycin-induced filamentation; gene has intron

orf19.454	SFL1	71.89	63.4	68.96	57.07	-0.08472955	0.998825	Transcription factor involved in negative regulation of morphogenesis, flocculation and virulence; induced in core caspofungin response; Spider biofilm induced
orf19.3282	BMT3	74.46	63.39	49.43	40.09	-0.459244786	0.509743	Beta-mannosyltransferase; adds 2nd beta-mannose to the acid-stable fraction of cell wall phosphopeptidomannan, elongation of beta-mannose chains on the phosphopeptidomannan acid-labile fraction; Hap43-induced; Spider biofilm induced
orf19.480	orf19.480	67.42	63.2	49.45	50.21	-0.295113726	0.861125	Protein of unknown function; rat catheter and Spider biofilm induced
orf19.6948	CCC1	71.27	63.2	76.3	59.2	-0.002848893	0.998825	Manganese transporter; required for normal filamentous growth; mRNA binds She3, localized to hyphal tips; repressed by NO, alkaline pH; colony morphology-related regulation by Ssn6; regulated by Sef1, Sfu1, Hap43; Spider biofilm induced
orf19.1012	orf19.1012	61.45	63.14	85.12	88.06	0.360201561	0.680517	Ortholog(s) have clathrin binding activity, role in Golgi to vacuole transport, endosomal transport and AP-1 adaptor complex, cytosol, endosome, nucleus localization
orf19.4625	TOA2	88.61	63.12	79.23	62.95	-0.082531005	0.998825	Putative TFIIA small subunit; protein abundance decreased in CAI4 strain compared to the SC5314 strain, abundance not affected by reintegration of URA3 in CAI4; flucytosine induced; possibly an essential gene (UAU1 method)
orf19.3185	orf19.3185	66.58	63.09	62.32	63.07	-0.051011881	0.998825	Ortholog(s) have peptide alpha-N-acetyltransferase activity, role in N-terminal protein amino acid acetylation and NatA complex, cytosolic ribosome, mitochondrion localization
orf19.6321	PGA48	48.73	63.08	41.75	51.69	-0.185974616	0.998825	Putative GPI-anchored adhesin-like protein; similar to S. cerevisiae Spi1p, which is induced at stationary phase; transcript induced in high iron; flow model biofilm induced; Spider biofilm repressed
orf19.6407	orf19.6407	74.68	63.06	66.5	59.03	-0.107679249	0.998825	Ortholog(s) have role in ribosomal large subunit biogenesis and cytoplasm, cytosolic large ribosomal subunit, preribosome, large subunit precursor localization

orf19.445	orf19.445	61.09	63.01	65.29	55.24	-0.046586664	0.998825	Protein of unknown function; repressed by prostaglandins
orf19.439	orf19.439	74.42	62.97	77.93	72.86	0.099149651	0.998825	Ortholog(s) have recombinase activity, single-stranded DNA binding activity
orf19.1444	orf19.1444	71.39	62.95	71.2	67.97	0.031443166	0.998825	Ortholog(s) have phosphatidylinositol-4,5-bisphosphate binding activity, role in actin cortical patch assembly, actin filament organization, endocytosis and actin cortical patch, mating projection tip localization
orf19.4726	orf19.4726	57.57	62.94	80.92	73.83	0.25955459	0.892679	Ortholog(s) have calcium ion binding, enzyme activator activity
orf19.418	KOG1	67.85	62.92	61.33	59.09	-0.103425424	0.998825	Putative TORC1 subunit; heterozygous null mutant displays sensitivity to rapamycin; likely to be essential for growth, based on an insertional mutagenesis strategy
orf19.500	orf19.500	75.41	62.91	62.08	56.45	-0.177474525	0.998825	Ortholog(s) have tRNA (adenine-N1-)-methyltransferase activity, role in tRNA methylation and nucleus, tRNA (m1A) methyltransferase complex localization
orf19.3653	FAT1	72.17	62.85	60.15	55.68	-0.171841683	0.998825	Predicted enzyme of sphingolipid biosynthesis; upregulated in biofilm
orf19.6328	orf19.6328	51.87	62.76	69.15	56.6	0.082896438	0.998825	Putative protein of the mitochondrial intermembrane space; predicted role in acetate utilization and gluconeogenesis; Spider biofilm repressed
orf19.6927	PEP8	68.54	62.74	57.74	60.95	-0.119389027	0.998825	Protein similar to S. cerevisiae Pep8p, which is involved in retrograde transport; transposon mutation affects filamentous growth

orf19.3021	orf19.3021	67.91	62.74	59.78	52.63	-0.167893305	0.998825	Putative protein of unknown function; Hap43-repressed gene; Spider biofilm induced
orf19.4730	orf19.4730	76.74	62.7	54.1	68.59	-0.143286796	0.998825	Ortholog of S. cerevisiae : YNL050C, C. glabrata CBS138 : CAGL0G01276g, C. dubliniensis CD36 : Cd36_08220, C. parapsilosis CDC317 : CPAR2_804440 and Candida tenuis NRRL Y-1498 : CANTEDRAFT_94168
orf19.2917.1	orf19.2917.1	58.51	62.67	31.26	46.87	-0.448814254	0.568429	Ortholog of C. parapsilosis CDC317 : CPAR2_401885, Pichia stipitis Pignal : psti_CGOB_00141, Candida orthopsilosis Co 90-125 : CORT_0E01925 and Spathaspora passalidarum NRRL Y-27907 : spas_CGOB_00016
orf19.685	YHM1	63.7	62.65	100.25	87.21	0.42316139	0.517955	Putative mitochondrial carrier protein; fungal-specific (no human or murine homolog); Hap43p-repressed gene
orf19.2055	NPL6	69.18	62.65	62.65	63.99	-0.050171222	0.998825	Putative nuclear protein localization factor; Hap43-induced; Spider biofilm repressed
orf19.7499	orf19.7499	58.27	62.6	67.72	60.1	0.048500609	0.998825	Putative nicotinic acid mononucleotide adenylyltransferase, involved in NAD salvage pathway; Spider biofilm repressed
orf19.1634	orf19.1634	55.79	62.57	69.71	56.93	0.056296905	0.998825	Has domain(s) with predicted fatty-acyl-CoA binding activity
orf19.3759	orf19.3759	64.92	62.53	51.82	50.13	-0.248833052	0.927528	Putative elongator complex subunit; for modification of wobble nucleosides in tRNA; Spider biofilm induced
orf19.596.2	DAD4	53.26	62.42	39.38	43.93	-0.337334963	0.818506	Subunit of the Dam1 (DASH) complex, which acts in chromosome segregation by coupling kinetochores to spindle microtubules

orf19.5049	orf19.5049	73.82	62.41	49.69	52.1	-0.311649555	0.842201	Putative U3-containing 90S preribosome processome complex subunit; Hap43-induced gene; rat catheter and Spider biofilm induced; F-12/CO2 early biofilm induced
orf19.3792	orf19.3792	67.57	62.38	78.24	69.23	0.125093774	0.998825	Ortholog(s) have role in cytoplasmic mRNA processing body assembly, deadenylation-dependent decapping of nuclear-transcribed mRNA, formation of translation preinitiation complex, negative regulation of translational initiation
orf19.2520	orf19.2520	59.82	62.34	81.37	66.47	0.190562332	0.998825	Ortholog(s) have structural constituent of ribosome activity and mitochondrial small ribosomal subunit localization
orf19.3031	SEC62	76.72	62.3	95.16	88.04	0.301854726	0.819869	Putative endoplasmic reticulum (ER) protein-translocation complex subunit
orf19.6680	FGR27	62.48	62.22	58.26	53.56	-0.118182673	0.998825	Zn(II)2Cys6 transcription factor; transposon mutation affects filamentous growth; required for yeast cell adherence to silicone substrate; Spider biofilm induced
orf19.5530	NAB3	66.85	62.19	66.81	56.45	-0.061743448	0.998825	Putative nuclear polyadenylated RNA-binding protein; flucytosine repressed
orf19.168	orf19.168	69.26	62.09	58.5	60.62	-0.102232538	0.998825	Ortholog(s) have role in U4 snRNA 3'-end processing, exonucleolytic trimming to generate mature 3'-end of 5.8S rRNA from tricistronic rRNA transcript (SSU-rRNA, 5.8S rRNA and LSU-rRNA), more
orf19.2484	orf19.2484	66.12	62.07	81.58	73.2	0.200917194	0.976175	Has domain(s) with predicted peptidase activity and role in proteolysis
orf19.562	FGR13	59.08	62	59.33	57.78	-0.045870945	0.998825	Protein encoded in retrotransposon Zorro3 with a potential zinc finger; lacks an ortholog in S. cerevisiae; transposon mutation affects filamentous growth

orf19.1336.2	orf19.1336.2	70.93	61.96	61.81	72.08	0.001812322	0.998825	Ortholog(s) have role in mitochondrial respiratory chain complex assembly and mitochondrial intermembrane space, nucleus localization
orf19.991	DJP1	74.99	61.8	63.36	58.83	-0.133422183	0.998825	Similar to bacterial DnaJ; reported to have a role in peroxisome biogenesis; induced in low iron and upon adherence to polystyrene
orf19.2686	orf19.2686	58.47	61.75	52.26	67.2	-0.016170488	0.998825	Ortholog(s) have carboxypeptidase activity, role in nitrogen compound metabolic process, proteolysis involved in cellular protein catabolic process and fungal-type vacuole lumen localization
orf19.6863	VPH1	74.89	61.7	76.85	70.48	0.069192392	0.998825	Vacuolar H(+)-ATPase; transcription regulated by Nrg1, Mig1, and Tup1
orf19.4328	CCC2	63.24	61.65	56.99	53.57	-0.135885523	0.998825	Copper-transporting P-type ATPase of Golgi; required for wild-type iron assimilation (indirect effect via Fet3p); induced by iron starvation, ciclopirox olamine; caspofungin repressed; not required for virulence in mouse systemic infection
orf19.2009	PEX12	69.53	61.64	58.05	60.41	-0.128161202	0.998825	Ortholog(s) have ubiquitin-protein ligase activity, role in protein import into peroxisome matrix and integral to peroxisomal membrane localization
orf19.4164	orf19.4164	66.58	61.62	74.71	71.56	0.134314914	0.998825	Ortholog(s) have mitochondrion, ribosome localization
orf19.4889	orf19.4889	63.99	61.53	52.21	47.86	-0.252426954	0.921805	Predicted MFS family membrane transporter, member of the drug:proton antiporter (12 spanner) (DHA1) family; Spider biofilm induced
orf19.6871	orf19.6871	59.16	61.53	59.09	48.43	-0.140936321	0.998825	Protein of unknown function; Hap43-induced; flow model biofilm repressed

orf19.966	DUS4	64.85	61.5	49.01	46.23	-0.303957072	0.858075	Ortholog(s) have tRNA dihydrouridine synthase activity, role in tRNA modification and mitochondrion localization
orf19.4143	FYV5	77.26	61.5	47.98	56.26	-0.303931372	0.853836	Protein with a predicted role maturation of 18S rRNA; rat catheter biofilm induced
orf19.7638	PRO1	64.13	61.46	57.5	53.96	-0.135539806	0.998825	Putative gamma-glutamyl kinase; transcript regulated by Nrg1; regulated by Gcn2 and Gcn4; Hap43-repressed gene; early-stage flow model biofilm induced gene
orf19.4954	orf19.4954	64.2	61.23	82	73.14	0.226376956	0.938642	Ortholog(s) have endoplasmic reticulum, integral to membrane localization
orf19.4728	orf19.4728	57.28	61.2	59.64	60.19	0.007679923	0.998825	Ortholog(s) have role in histone deacetylation, negative regulation of meiosis and Set3 complex localization
orf19.6018	LRO1	53.02	61.2	81.64	82.13	0.386099656	0.626697	Acyltransferase that catalyzes diacylglycerol esterification of phospholipids; role in lipid storage, triglyceride biosynthesis; flow model biofilm repressed
orf19.6026	ERG2	75.02	61.17	72.47	91.2	0.194716193	0.988598	C-8 sterol isomerase; enzyme of ergosterol biosynthesis; converts fecosterol to episterol; mutant is hypersensitive to multiple drugs; ketoconazole-induced; flow model and Spider biofilm repressed
orf19.3846	LYS4	70.33	61.12	79.48	88.32	0.262614215	0.881232	Homoaconitase; regulated by Gcn4, Gcn2; induced in response to amino acid starvation (3-AT); induced by human whole blood or PMNs; Hap43-repressed; flow model and Spider biofilm repressed
orf19.6781	ZFU2	63.76	61.11	64.37	62.9	0.007986041	0.998825	Zn(II)2Cys6 transcription factor; regulator of yeast form adherence; mutants display increased colonization of mouse kidneys; required for yeast cell adherence to silicone substrate; Spider biofilm induced

orf19.3841	orf19.3841	67.01	61.09	56.99	52.78	-0.170253534	0.998825	Putative protein serine/threonine kinase; predicted role in vesicle formation in autophagy and the cytoplasm-to-vacuole targeting (Cvt) pathway; Spider biofilm induced
orf19.2895	VMA8	73.38	61.05	94.67	81.88	0.302353162	0.823496	Putative vacuolar H+-ATPase subunit; regulated by Nrg1 and Mig1; transcript increases in populations of cells exposed to fluconazole over multiple generations; rat catheter and Spider biofilm repressed
orf19.6612	orf19.6612	69.16	61.05	82.72	69.41	0.161717631	0.998825	Putative mitochondrial protein; Hap43p-induced gene
orf19.4959	orf19.4959	52.95	61.01	62.01	59.33	0.051457898	0.998825	Protein of unknown function; Spider biofilm repressed
orf19.4875	orf19.4875	45.35	60.98	46.03	50.81	-0.100368624	0.998825	Ortholog(s) have role in mRNA splicing, via spliceosome and U2-type spliceosomal complex localization
orf19.2575	orf19.2575	65.72	60.97	59.07	48.25	-0.191574473	0.998825	Putative S-adenosylmethionine-dependent methyltransferase; Hap43p-induced gene
orf19.2985	orf19.2985	68.51	60.96	64.94	56.28	-0.086769988	0.998825	Protein of unknown function; Hap43-repressed gene
orf19.6668	orf19.6668	56.38	60.96	70.77	70.73	0.198760799	0.987997	Ortholog(s) have ubiquitin-protein ligase activator activity and role in ER-associated ubiquitin-dependent protein catabolic process, establishment of protein localization to endoplasmic reticulum membrane
orf19.3347	RPB7	74.82	60.95	66.78	66	-0.031623189	0.998825	Functional homolog of S. cerevisiae Rpb7; essential subunit of RNA Polymerase II; enhances hyperfilamentation of an S. cerevisiae rpb4 mutant; suppresses defects of S. cerevisiae rpb4 and ess1 mutants; Spider biofilm repressed

orf19.5147	LMO1	67.13	60.88	75.37	70.33	0.127386835	0.998825	Protein involved together with Dck1p and Rac1p in invasive filamentous growth and cell wall integrity; acts upstream of MAP kinase Cek1p; has similarity to human ELMO1
orf19.7374	CTA4	57.53	60.88	42.04	44.9	-0.329092853	0.824321	Zn(II)2Cys6 transcription factor; induced by nitric oxide; induced by Mnl1 under weak acid stress; repressed upon adherence to polystyrene; Hap43-repressed; Spider biofilm induced
orf19.2214	orf19.2214	63.89	60.8	73.14	72.23	0.153083709	0.998825	Ortholog(s) have structural constituent of ribosome activity and mitochondrial large ribosomal subunit localization
orf19.7404	orf19.7404	76.46	60.77	74.18	60.41	-0.033336137	0.998825	Catabolic L-serine (L-threonine) deaminase;, catalyzes degradation of L-serine and L-threonine; required to use serine or threonine as a sole nitrogen source; Spider biofilm repressed
orf19.1840	orf19.1840	56.12	60.68	93.07	93.86	0.511167379	0.312332	Predicted lipid-binding ER protein; involved in ER-plasma membrane tethering; Spider biofilm induced
orf19.2605	PRK1	62.3	60.67	67.65	63.52	0.066306417	0.998825	Putative protein serine/threonine kinase; mutants sensitive to growth on hydrogen peroxide medium
orf19.3003	orf19.3003	49.44	60.67	92.68	96.46	0.583703444	0.204164	Putative lipid-binding protein with a predicted role in calcium-dependent phospholipid-binding
orf19.3370	DOT4	68.64	60.66	58.6	47.73	-0.216200869	0.957711	Protein similar to ubiquitin C-terminal hydrolase; localizes to cell surface of hyphal cells, but not yeast-form cells; repressed upon high-level peroxide; Hap43p-induced; rat catheter biofilm induced
orf19.2610	orf19.2610	60.25	60.58	69.98	76.66	0.2029966	0.976613	Putative tRNA binding protein; intron-containing gene; Spider biofilm induced

orf19.5250	orf19.5250	88.69	60.58	86.46	82.81	0.127393691	0.998825	Has domain(s) with predicted cofactor binding activity
orf19.4775	CTA8	56.4	60.58	54.39	53.8	-0.094064708	0.998825	Essential transcription factor, mediates heat shock transcriptional induction; in the absence of heat stress, Cta8p levels are modulated by growth temperature to regulate basal expression of genes involved in protein folding
orf19.6325.1	orf19.6325.1	71.86	60.58	82.89	61.27	0.075463084	0.998825	Ortholog(s) have mitochondrion localization
orf19.535	RBR1	71.51	60.58	60.44	61.62	-0.100277864	0.998825	Glycosylphosphatidylinositol (GPI)-anchored cell wall protein; required for filamentous growth at acidic pH; expression repressed by Rim101 and activated by Nrg1; Hap43-induced
orf19.5045	PTP2	55.31	60.42	25.83	27.36	-0.74668948	0.098488	Predicted protein tyrosine phosphatase; involved in regulation of MAP kinase Hog1 activity; induced by Mnl1 under weak acid stress; rat catheter and Spider biofilm induced
orf19.825	GCD7	65.22	60.41	56.68	55.97	-0.116035234	0.998825	Putative translation initiator; downregulated in the presence of human whole blood or polymorphonuclear (PMN) cells
orf19.1377	IPK2	65.79	60.4	68.64	76.8	0.153508495	0.998825	Putative inositol polyphosphate multikinase; moderately induced at 42 deg
orf19.703	orf19.703	58.8	60.32	56.07	61.69	-0.017422728	0.998825	Ortholog(s) have 2',3'-cyclic-nucleotide 3'-phosphodiesterase activity, role in cyclic nucleotide metabolic process and clathrin-coated vesicle localization
orf19.4211	orf19.4211	58.78	60.28	66.62	53.2	-0.003176405	0.998825	Multicopper oxidase; for growth in low iron, prostaglandin E2 synthesis; ketoconazole/caspofungin/amphotericin B repressed; Sef1/Sfu1/Hap43 regulated; reports differ if functional homolog of ScFet3; rat catheter and Spider biofilm induced

orf19.1542	HEX3	61.83	60.27	61.15	56.52	-0.045305355	0.998825	Protein similar to S. cerevisiae Hex3p, which is involved in DNA damage response; transposon mutation affects filamentous growth
orf19.7203	MRP7	65.41	60.26	54.04	55.93	-0.143540196	0.998825	Mitochondrial ribosomal protein of the large subunit; rat catheter biofilm induced
orf19.7298	CHS2	57.5	60.22	65.53	63.25	0.093984775	0.998825	Chitin synthase; nonessential; required for wild-type chitin deposition in hyphae; transcript regulated during dimorphic transition; Chs1 and Chs2, but not Chs3, are inhibited by the protoberberine HWY-289; flow model biofilm repressed
orf19.1777	orf19.1777	61.49	60.14	67.76	66.93	0.108867746	0.998825	Ortholog(s) have ubiquitin-specific protease activity, role in endocytosis, protein deubiquitination and cell division site, cytosol, nucleus, peroxisome, transport vesicle localization
orf19.2867	orf19.2867	67.02	60.12	60.44	55.2	-0.115029327	0.998825	Ortholog(s) have phosphatidylinositol-3-phosphate binding, protein transporter activity and role in ascospore formation, intracellular protein transport, protein retention in Golgi apparatus, retrograde transport, endosome to Golgi
orf19.5720	orf19.5720	67.94	60.05	77.35	67.33	0.119132939	0.998825	Predicted membrane transporter, member of the monocarboxylate porter (MCP) family, major facilitator superfamily (MFS); ketoconazole or caspofungin repressed; Spider biofilm induced
orf19.1479	orf19.1479	45.86	60.03	48.1	54.83	-0.039672054	0.998825	Ortholog of the mitochondria localized S. cerevisiae Pib2 protein of unknown function; has a FYVE zinc finger domain; Spider biofilm induced
orf19.320	orf19.320	52.89	60.01	42.04	42.21	-0.309567462	0.858169	Predicted short chain dehydrogenase; Spider biofilm induced
orf19.3201	MTLA1	56.95	59.97	70.46	57.07	0.075162533	0.998825	Homeodomain protein of the MTLa mating type locus; a1 and alpha2 together repress white-opaque switching and mating (an opaque-specific process); a/alpha mating type may increase virulence by competitive advantage; Spider biofilm induced

orf19.3058	COQ6	56.98	59.93	80.54	86.3	0.381621292	0.631397	Ortholog(s) have role in ubiquinone biosynthetic process and mitochondrial inner membrane localization
orf19.30	SPF1	63.25	59.93	73.55	76.41	0.208528897	0.957711	P-type calcium-transporting ATPase, involved in control of calcium homeostasis, response to ER stress, hyphal growth, biofilm formation and virulence
orf19.5457	orf19.5457	67.1	59.8	77.82	72.46	0.173651885	0.998825	Ortholog(s) have Golgi apparatus, endoplasmic reticulum localization
orf19.4799	CEF1	66.87	59.77	51.96	47.1	-0.270387481	0.892679	Putative mRNA splicing factor; ortholog is essential in S. cerevisiae; Spider biofilm induced
orf19.4451	RIA1	73.21	59.76	60.61	57.13	-0.136417592	0.998825	Putative translation elongation factor; genes encoding ribosomal subunits, translation factors, and tRNA synthetases are downregulated upon phagocytosis by murine macrophage
orf19.5357	AKL1	59.93	59.74	63.95	56.06	-0.010827826	0.998825	Putative serine/threonine protein kinase; induced during the mating process
orf19.2459	orf19.2459	49.44	59.7	80.38	90.58	0.477938731	0.41868	Protein of unknown function; mRNA binds to She3; Hap43 repressed gene; Spider biofilm induced
orf19.7554	orf19.7554	78.15	59.7	24.44	18.16	-1.131649334	0.001554	Transporter; similar to the Sit1 siderophore transporter; induced by nitric oxide independent of Yhb1; repressed during chlamydospore formation in C. albicans and C. dubliniensis; rat catheter biofilm repressed
orf19.2695	UBR1	56.68	59.66	59.86	57.62	-0.001296898	0.998825	Protein similar to S. cerevisiae Ubr1p ubiquitin-protein ligase; transposon mutation affects filamentous growth; Spider biofilm induced

orf19.5064	MRPL3	63.99	59.65	59.36	59.18	-0.061656504	0.998825	Ribosomal protein of the large subunit, mitochondrial; repressed in core stress response; protein present in exponential and stationary growth phase yeast cultures
orf19.836.1	orf19.836.1	62.53	59.62	54.68	71.92	0.028081596	0.998825	Ortholog(s) have role in mRNA splicing, via spliceosome and U1 snRNP, U2 snRNP, U2-type prespliceosome, U4/U6 x U5 tri-snRNP complex, U5 snRNP, cytosol localization
orf19.1210	orf19.1210	62.06	59.61	68.31	62.95	0.066819654	0.998825	Ortholog(s) have L-arginine transmembrane transporter activity, L-aspartate transmembrane transporter activity and L-glutamate transmembrane transporter activity, more
orf19.6600	orf19.6600	72.72	59.6	58.15	59.76	-0.135214012	0.998825	Ortholog(s) have phosphatidic acid transporter activity and role in cardiolipin metabolic process, phospholipid transport, positive regulation of phosphatidylcholine biosynthetic process
orf19.2487	orf19.2487	62.86	59.57	52.41	44.12	-0.268183148	0.8944	Protein with a predicted phosphoribulokinase/uridine kinase domain; Spider biofilm induced
orf19.1440	orf19.1440	74.24	59.57	64.13	76.52	0.050324101	0.998825	Ortholog of C. dubliniensis CD36 : Cd36_43090, C. parapsilosis CDC317 : CPAR2_403340, Candida tenuis NRRL Y-1498 : CANTEDRAFT_94387 and Debaryomyces hansenii CBS767 : DEHA2G17622g
orf19.6568	SMC6	62.83	59.55	51.13	47.21	-0.246835401	0.931333	Putative structural maintenance of chromosomes (SMC) protein; Hap43-induced; cell-cycle regulated periodic mRNA expression; S. cerevisiae ortholog not cell-cycle regulated; Spider biofilm induced
orf19.2396	IFR2	52.92	59.53	40.23	37.6	-0.381267329	0.71836	Zinc-binding dehydrogenase; induced by benomyl, ciclopirox olamine, alpha pheromone, Hap43; regulated by oxidative stress via Cap1, osmotic stress via Hog1; protein present in exponential and stationary phase; rat catheter biofilm repressed
orf19.6534.2	CRN1	69.27	59.51	86.19	74.1	0.228425698	0.938404	Coronin; cortical actin cytoskeletal component; predicted role in regulation of actin patch assembly; rat catheter and Spider biofilm repressed

orf19.6509	orf19.6509	65.2	59.33	71.28	61.24	0.057483293	0.998825	Ortholog(s) have role in cellular response to zinc ion and cytosol, nucleus localization
orf19.5735.3	orf19.5735.3	66.87	59.19	50.02	53.47	-0.219200181	0.957711	Protein of unknown function; Spider biofilm induced
orf19.1802	OFD1	72.6	59.18	65.72	60.21	-0.058277815	0.998825	Putative prolyl hydroxylase family member; regulates Ume6p stability in response to oxygen; inhibited by hypoxia; repressed by prostaglandins; Spider biofilm induced
orf19.1468	CDC55	69.31	59.15	86.73	78.25	0.272523188	0.870631	Ortholog(s) have protein phosphatase type 2A regulator activity, protein serine/threonine phosphatase activity
orf19.5325	KIN3	67.87	59.13	74.06	63.16	0.072654346	0.998825	Protein similar to S. cerevisiae Kin3p; induced under Cdc5p depletion; shows Mob2p-dependent hyphal regulation; mutants are hypersensitive to caspofungin
orf19.2306	orf19.2306	56.97	59.13	69.12	69.09	0.17727894	0.998825	Ortholog(s) have acireductone dioxygenase (Ni2+-requiring) activity, role in L-methionine salvage from methylthioadenosine and cytosol, nucleus localization
orf19.3928	orf19.3928	61.59	59.1	64.21	44.04	-0.1308439	0.998825	Putative transcription factor with zinc finger DNA-binding motif
orf19.4339	VPS4	77.17	59.02	71.03	47	-0.160459216	0.998825	AAA-ATPase involved in transport from MVB to the vacuole and ESCRT-III complex disassembly; mutation decreases SAP secretion and virulence in murine intravenous infection; regulated by Gcn2p, Gcn4p; required for normal Rim8p processing
orf19.4802	FTH1	59.43	58.97	59.12	43	-0.16319612	0.998825	Protein similar to S. cerevisiae Fth1p, a high affinity iron transporter for intravacuolar stores of iron; repressed by Sfu1p, amphotericin B, caspofungin; induced by alkaline pH, ciclopirox olamine; regulated by Sef1p, Sfu1p, and Hap43p

orf19.5651	orf19.5651	61.37	58.96	60.27	54.64	-0.0550611	0.998825	Ortholog(s) have histone demethylase activity (H3-dimethyl-K4 specific), histone demethylase activity (H3-trimethyl-K4 specific) activity
orf19.1856	orf19.1856	60.05	58.92	57.5	57.47	-0.054602294	0.998825	Ortholog(s) have histone deacetylase activity
orf19.4634	orf19.4634	74.33	58.84	52.57	48.92	-0.287552104	0.870631	Protein required for thiolation of uridine at wobble position of Gln, Lys, and Glu tRNAs; has a role in urmylation; S. cerevisiae ortholog has a role in invasive and pseudohyphal growth
orf19.5782	orf19.5782	69.86	58.78	70.4	62.04	0.016050884	0.998825	Ortholog(s) have mitochondrion localization
orf19.6643	MCT1	68.68	58.74	50.18	41.37	-0.363069752	0.736076	Putative malonyl-CoA acyl carrier protein acyltransferase
orf19.4183	MUC1	58.39	58.71	64.92	60.2	0.060118973	0.998825	Protein similar to S. cerevisiae Muc1; a cell surface glycoprotein involved in filamentous growth; repressed by benomyl treatment; mutant is viable
orf19.497	EAF7	56.83	58.63	66.84	73.35	0.193610181	0.998825	Subunit of the NuA4 histone acetyltransferase complex
orf19.2342	SFT2	66.19	58.52	47.24	51.9	-0.250551631	0.927778	Putative membrane protein; transcript regulated by Mig1; Spider biofilm induced
orf19.6951	orf19.6951	63.43	58.48	58.03	44.84	-0.178922696	0.998825	Ortholog(s) have sphinganine-1-phosphate aldolase activity and role in calcium-mediated signaling, cellular response to starvation, sphingolipid metabolic process

orf19.1466	REX2	74.01	58.43	61.51	59.07	-0.100579643	0.998825	Putative 3'-5' RNA exonuclease with a predicted role in 3'-end processing of U4 and U5 snRNAs, 5S and 5.8S rRNAs; rat catheter biofilm induced
orf19.7067	orf19.7067	58.36	58.39	64.36	61.08	0.069780632	0.998825	Ortholog(s) have RNA polymerase II core binding, RNA polymerase II transcription factor binding transcription factor activity, triplex DNA binding activity
orf19.2328	orf19.2328	57.65	58.31	64.97	70.36	0.154233021	0.998825	Ortholog of C. dubliniensis CD36 : Cd36_10220, C. parapsilosis CDC317 : CPAR2_210530, Candida tenuis NRRL Y-1498 : CANTEDRAFT_105553 and Debaryomyces hansenii CBS767 : DEHA2G14432g
orf19.5020	orf19.5020	59.31	58.19	54.74	44.62	-0.173924643	0.998825	Protein of unknown function; Hap43-induced; Spider biofilm induced
orf19.7086	orf19.7086	54.16	58.18	61.51	53.99	0.025308947	0.998825	Ortholog(s) have protein transporter activity, role in transcription factor import into nucleus and cytosol, nuclear envelope localization
orf19.5985	orf19.5985	57.47	58.14	58.01	46.52	-0.106217685	0.998825	Ortholog(s) have tubulin binding activity, role in response to cold, tubulin complex assembly and nucleus, polysome, prefoldin complex localization
orf19.1533	orf19.1533	53.96	58	52.73	49.73	-0.09739848	0.998825	Possible vacuolar protein; Hap43-induced gene
orf19.3682	CWH8	64.03	57.95	68.58	90.61	0.289036277	0.8593	Putative dolichyl pyrophosphate (Dol-P-P) phosphatase; ketoconazole-induced; expression is increased in a fluconazole-resistant isolate; clade-associated gene expression; Hap43p-induced gene
orf19.3027	orf19.3027	61.26	57.94	65.87	57.78	0.033741913	0.998825	Putative DNA translocase; Spider biofilm repressed

orf19.3184	orf19.3184	60.9	57.91	64.29	58.04	0.016674865	0.998825	Ortholog(s) have Golgi apparatus, fungal-type vacuole membrane localization
orf19.7577	MSS51	63.17	57.86	71.84	63.83	0.116552978	0.998825	Putative mRNA maturation factor; fungal-specific (no human or murine homolog)
orf19.7044	RIM15	57.34	57.85	75.67	75.26	0.286453008	0.861125	Ortholog(s) have protein kinase activity and role in age-dependent response to oxidative stress involved in chronological cell aging, conidiophore development, hyphal growth, protein phosphorylation, regulation of meiosis
orf19.1341	PRR2	47.77	57.82	47.45	48.41	-0.121801447	0.998825	Putative serine/threonine protein kinase; mutation confers resistance to 5-fluorocytosine (5-FC)
orf19.3829	PHR1	48.3	57.76	189.87	183.25	1.462878326	9.37E-09	Cell surface glycosidase; may act on cell-wall beta-1,3-glucan prior to beta-1,6-glucan linkage; role in systemic, not vaginal virulence (neutral, not low pH); high pH or filamentation induced; Bcr1-repressed in RPMI a/a biofilm
orf19.806	VMA7	50.97	57.75	84.16	61.54	0.297056378	0.858075	Putative subunit of the V-ATPase complex, which is involved in control of vacuolar pH; highly similar to S. cerevisiae Vma7p; interacts with phosphatidylinositol 3-kinase Vps34p
orf19.556	orf19.556	62.5	57.74	72.72	75.04	0.211501884	0.957711	Protein of unknown function; regulated by Ssn6
orf19.896	CHK1	58.56	57.72	79.31	75.31	0.290399911	0.858075	Histidine kinase; 2-component signaling, cell wall synthesis; hyphal growth defect; avirulent in mouse, not rat vaginal infection; phagocytosis rate increased; Spider biofilm induced; required for RPMI biofilm; Bcr1-induced in a/a biofilm
orf19.2378	orf19.2378	77.18	57.72	60.44	69.53	-0.045055281	0.998825	Ortholog(s) have role in syncytium formation by plasma membrane fusion, vacuolar proton-transporting V-type ATPase complex assembly and integral to endoplasmic reticulum membrane localization

orf19.1150	orf19.1150	70.33	57.69	69.75	56.87	-0.017924695	0.998825	GATA-like transcription factor; oral infection induced; mutant has reduced capacity to damage oral epithelial cells; regulated by Gcn2 and Gcn4; Spider biofilm induced
orf19.7314	CDG1	52.25	57.48	45.45	40.67	-0.249873833	0.936054	Putative cysteine dioxygenases; role in conversion of cysteine to sulfite; transcript regulated upon white-opaque switch; rat catheter, Spider and flow model biofilm induced
orf19.1959	orf19.1959	61.18	57.47	67.41	63.31	0.094661382	0.998825	Ortholog(s) have ubiquitin-specific protease activity, role in protein deubiquitination and cytosol, ribosome localization
orf19.6092	KEL1	56.39	57.45	79.46	69.71	0.288734662	0.8593	Kelch repeat domain-containing protein; localizes to sites of polarized growth; mutant colonies exhibit slightly decreased filamentation ratio; not required for buccal epithelial cell adherence or virulence in mice
orf19.3013	CDC12	64.53	57.44	103.97	115.97	0.66075864	0.084475	Septin; essential for viability; forms ring at sites of cell division and also forms filaments in mature chlamydospore; filamentous growth induced; regulated by Nrg1, Tup1, tyrosol and cell density; rat catheter biofilm repressed
orf19.6565	orf19.6565	56.43	57.41	69.93	68.9	0.213024019	0.957711	Conserved mitochondrial inner membrane insertase; mediates insertion of mitochondrial- and nuclear-encoded proteins from the matrix into the inner membrane; Spider biofilm repressed
orf19.1482	HSK3	42.74	57.39	52.17	63.59	0.140960846	0.998825	Subunit of the Dam1 (DASH) complex, which acts in chromosome segregation by coupling kinetochores to spindle microtubules; expression regulated during planktonic growth
orf19.5101	CCR4	63.57	57.38	61.84	57.14	-0.026729363	0.998825	Component of the Ccr4-Pop2 mRNA deadenylase; transposon mutation affects filamentous growth
orf19.4676	orf19.4676	55.94	57.35	63.3	61.48	0.088749919	0.998825	Protein with homology to mitochondrial intermembrane space proteins; regulated by Sef1p-, Sfu1p-, and Hap43p

orf19.6234	orf19.6234	66.39	57.34	51.77	54.17	-0.163724617	0.998825	Putative U2 snRNP component; mutation confers hypersensitivity to 5-fluorocytosine (5-FC), 5-fluorouracil (5-FU), and tubercidin (7-deazaadenosine); Hap43-induced, Spider biofilm induced
orf19.3528	orf19.3528	55.98	57.34	53.52	61.79	0.019463024	0.998825	Protein of unknown function; Spider biofilm induced
orf19.7200	orf19.7200	61.34	57.23	67.85	69.67	0.160725404	0.998825	Ortholog of C. dubliniensis CD36 : Cd36_73480, Pichia stipitis Pignal : PICST_30727, Candida tropicalis MYA-3404 : CTRG_05165 and Candida albicans WO-1 : CAWG_05712
orf19.6283	orf19.6283	60.84	57.2	47.2	38.03	-0.344296215	0.79131	Ortholog(s) have mitochondrion localization
orf19.7328	orf19.7328	62.24	57.11	91.61	90.61	0.470908566	0.404341	Protein with a Staphylococcal nuclease domain; transcript regulated by Mig1 and Tup1; flow model and Spider biofilm repressed
orf19.291	orf19.291	72.11	57.09	67.4	66.14	0.026102331	0.998825	Ortholog(s) have role in cellular response to DNA damage stimulus, protein sumoylation, response to UV-B, response to UV-C and nuclear envelope localization
orf19.5551	MIF2	52.51	57.01	37.88	34.06	-0.428815713	0.608044	Centromere-associated protein; similar to CENP-C proteins; Cse4p and Mif2p colocalize at C. albicans centromeres
orf19.7177	KAP120	56.1	56.95	54.83	47.32	-0.117109619	0.998825	Putative karyopherin; mutation confers hypersensitivity to cytochalasin D
orf19.4538	orf19.4538	74.55	56.92	55.88	56.76	-0.171252851	0.998825	Ortholog(s) have mRNA binding, protein heterodimerization activity, role in mRNA cleavage, mRNA polyadenylation and U2 snRNP, mRNA cleavage factor complex localization

orf19.1091	NOP8	61.67	56.92	50.47	50.36	-0.18958372	0.998825	Ortholog of S. cereviiae Nop8; has a role in ribosomal large subunit biogenesis; rat catheter and Spider biofilm induced
orf19.6691	orf19.6691	68.28	56.89	59.25	50.18	-0.153644717	0.998825	Putative ember of the multi-drug and toxin extrusion (MATE) family of the multidrug/oligosaccharidyl-lipid/polysaccharide exporter superfamily; Spider biofilm induced
orf19.3170	orf19.3170	56.38	56.78	54.03	50.97	-0.086474556	0.998825	Ortholog(s) have role in RNA Polymerase I assembly, RNA Polymerase II core complex assembly, RNA Polymerase III assembly, formation of translation preinitiation complex and cytoplasm, cytosolic ribosome localization
orf19.3530	CKA2	56.98	56.77	49.61	35.52	-0.296462475	0.871816	Catalytic alpha-subunit of protein kinase CK2; interaction with calcineurin pathway affects fluconazole sensitivity; synthetically lethal with CKA1; attenuated virulence in a mouse oropharyngeal candidiasis but not in a systemic mouse model
orf19.6739	orf19.6739	56.49	56.77	59.14	56.32	0.008412896	0.998825	Ortholog(s) have phosphopentomutase activity, role in deoxyribose phosphate catabolic process and cytosol, nucleus localization
orf19.6812	PMT2	53.25	56.66	94.33	90.37	0.557800538	0.23914	Protein mannosyltransferase (PMT) with roles in hyphal growth and drug sensitivity; member of the PMT family which includes Pmt1p, Pmt2p, Pmt4p, Pmt5p, and Pmt6p; induced during cell wall regeneration; essential
orf19.4235	PDE1	70.53	56.64	33.35	26.93	-0.75262258	0.083254	Low affinity cyclic nucleotide phosphodiesterase; mediates cAMP signaling in response to glucose or intracellular acidification; macrophage-repressed; Spider biofilm induced
orf19.4229	orf19.4229	75	56.56	86.82	84.77	0.28764616	0.853836	Putative polyphosphate phosphatase; role in hydrolysis of diphosphorylated inositol polyphosphates and diadenosine polyphosphates; Spider biofilm induced
orf19.4373	orf19.4373	54.36	56.54	64.83	60.41	0.115096877	0.998825	Protein similar to S. cerevisiae Fmn1p, which is riboflavin kinase; predicted Kex2p substrate; Hap43p-repressed gene

orf19.4563	orf19.4563	52.02	56.54	38.78	42.29	-0.305819775	0.863837	Protein of unknown function; repressed by prostaglandins; Hap43-induced, Spider biofilm induced
orf19.5241	SNT1	63.6	56.54	65.51	56.26	-0.001049788	0.998825	Ortholog of S. cerevisiae Snt1; an NAD-independent histone deacetylase; heterozygous transposon mutation affects filamentous growth; null mutants are viable
orf19.792	orf19.792	60.89	56.5	45.07	41.34	-0.332595345	0.819869	Ortholog(s) have protein serine/threonine kinase activity, role in activation of bipolar cell growth, protein phosphorylation, regulation of cytokinesis, regulation of mitotic sister chromatid segregation and cytosol, nucleus localization
orf19.2018.2	orf19.2018.2	41.77	56.49	33.49	53.06	-0.131595553	0.998825	Ortholog of Candida tropicalis MYA-3404 : CTRG_04298
orf19.2259	orf19.2259	50.81	56.39	49.28	56.46	-0.028180878	0.998825	Ortholog(s) have structural constituent of ribosome activity and mitochondrial large ribosomal subunit localization
orf19.7637	YHB4	42.22	56.37	35.58	38.51	-0.268221438	0.921805	Protein related to flavohemoglobins; not required for wild-type nitric oxide resistance; has predicted globin, FAD-binding, and NAD(P)-binding domains but lacks some conserved residues of flavohemoglobins; Hap43p-repressed gene
orf19.5845	RNR3	64.74	56.37	48.5	54.86	-0.164581724	0.998825	Putative ribonucleotide reductase large subunit; transcript induced in low iron; rat catheter and Spider biofilm induced
orf19.298	orf19.298	50.5	56.31	56.18	61.18	0.083856554	0.998825	Protein of unknown function; flow model biofilm induced
orf19.1504	orf19.1504	73.12	56.26	72.14	66.65	0.073860221	0.998825	Putative patatin-like phospholipase; fungal-specific (no human or murine homolog)

orf19.6007	orf19.6007	75.31	56.15	68.39	52.11	-0.102009237	0.998825	Predicted fatty acid acyl transferase-related protein domain; repressed by prostaglandins
orf19.1961	orf19.1961	54.76	56.03	63.82	64.25	0.141988028	0.998825	Planktonic growth-induced gene
orf19.1109	orf19.1109	50.93	56	52.31	57.36	0.009897008	0.998825	Ortholog of C. dubliniensis CD36 : Cd36_53580, C. parapsilosis CDC317 : CPAR2_303030, Debaryomyces hansenii CBS767 : DEHA2G07766g and Pichia stipitis Pignal : psti_CGOB_00176
orf19.4076	MET10	57.69	55.99	101.85	85.12	0.538094241	0.265944	Sulfite reductase; role in sulfur amino acid metabolism; induced by human whole blood or PMNs; Hog1-induced; possibly adherence-induced; flow model, Spider model, F-12/CO2 biofilm induced
orf19.5987	orf19.5987	59.55	55.98	46.28	37.87	-0.337658646	0.811201	Ortholog(s) have tRNA (guanine-N2-)-methyltransferase activity, role in tRNA methylation and cytosol, nucleus, tRNA (m2G10) methyltransferase complex localization
orf19.3922	orf19.3922	59.5	55.97	50.58	50.56	-0.133428526	0.998825	Possible pyrimidine 5' nucleotidase; protein present in exponential and stationary growth phase yeast cultures; Hap43p-repressed gene
orf19.299	ECM14	58.36	55.96	80.85	79.23	0.361660505	0.692027	Ortholog(s) have endoplasmic reticulum, fungal-type vacuole localization
orf19.1338	orf19.1338	56.82	55.95	61.98	61.78	0.089516072	0.998825	Protein of unknown function; possible COPI-coated vesicle, Golgi apparatus, ribosome localization; rat catheter biofilm repressed
orf19.7006	orf19.7006	49.97	55.82	50.39	48.08	-0.090850886	0.998825	Ortholog(s) have role in reciprocal meiotic recombination

orf19.2834	RPD3	45.48	55.8	34.41	41.86	-0.284266756	0.893138	Histone deacetylase; regulates frequency of white-to-opaque and opaque-to-white switching; greater expression in white cells than opaque cells; has conserved deacetylation motif
orf19.5660	orf19.5660	65.6	55.79	72.28	62.48	0.09196942	0.998825	Ortholog(s) have ubiquitin-protein ligase activity, role in protein import into peroxisome matrix, protein ubiquitination and endoplasmic reticulum, peroxisomal membrane localization
orf19.5779	RNR1	58.66	55.76	52.7	45.29	-0.175635673	0.998825	Ribonucleotide reductase large subunit; induced in low iron; transposon mutation affects filamentous growth; farnesol upregulated in biofilm; regulated by cell cycle, tyrosol, cell density; regulated by Sef1, Sfu1, and Hap43
orf19.5211	IDP1	60.89	55.7	83.64	85.89	0.404058824	0.576749	Putative isocitrate dehydrogenase; transcriptionally induced by interaction with macrophage; alkaline induced; Spider biofilm repressed
orf19.2633	HGT14	56.74	55.68	49.31	45.75	-0.188116077	0.998825	Putative MFS glucose transporter; 20 member C. albicans glucose transporter family; 12 probable membrane-spanning segments; expressed in rich medium with 2% glucose; rat catheter and flow model biofilm induced
orf19.6899	orf19.6899	64.38	55.65	37.67	38.8	-0.455034249	0.543746	Putative oxidoreductase; mutation confers hypersensitivity to toxic ergosterol analog; rat catheter and Spider biofilm induced
orf19.3811	GYP1	58.96	55.65	67.76	77.45	0.242723667	0.928284	Putative Cis-golgi GTPase-activating protein; transcript regulated by Nrg1, Mig1, and Tup1
orf19.6167	AYR1	56.52	55.64	78.01	74.33	0.312572159	0.823496	Putative oxidoreductase; transcriptionally induced by interaction with macrophage; rat catheter biofilm repressed
orf19.4724	orf19.4724	47.99	55.63	45.14	36.74	-0.247819237	0.938642	Ortholog(s) have RNA binding activity, role in spliceosomal complex assembly and U2-type prespliceosome localization

orf19.1672	orf19.1672	57.15	55.62	73.38	72.44	0.260286828	0.894293	Alpha subunit of COPI vesicle coatomer complex; role in ER to Golgi vesicle-mediated transport, retrograde vesicle-mediated transport, Golgi to ER transport; flow model biofilm repressed
orf19.5215	TES15	64.68	55.62	46.12	35.86	-0.404456433	0.646416	Putative acyl-CoA thioesterase; Hap43-repressed; Spider biofilm induced
orf19.2157	DAC1	54.57	55.61	39.39	37.42	-0.38723181	0.70336	N-acetylglucosamine-6-phosphate (GlcNAcP) deacetylase; N-acetylglucosamine utilization; required for wild-type hyphal growth and virulence in mouse systemic infection; gene and protein are GlcNAc-induced; Spider biofilm induced
orf19.5411	orf19.5411	55.63	55.61	56.81	53.48	-0.028867812	0.998825	Ortholog(s) have NEDD8 ligase activity, role in protein neddylation, regulation of mitotic cell cycle and cytosol, nucleus localization
orf19.2983	CDC73	64.48	55.52	63.81	61.08	0.035278022	0.998825	Putative transcription elongation factor; cdc73 kap114 double transposon mutation affects filamentous growth; Spider biofilm repressed
orf19.5869	orf19.5869	54.59	55.45	50.93	43.15	-0.171432268	0.998825	Ortholog(s) have role in metal ion transport, protein targeting to vacuole, ubiquitin-dependent protein catabolic process and endoplasmic reticulum, fungal-type vacuole membrane localization
orf19.420	orf19.420	51.1	55.43	51.75	52.12	-0.029586216	0.998825	Protein of unknown function
orf19.5037	HRQ2	57.36	55.42	75.73	64.52	0.237004899	0.935972	Protein of unknown function; mutants are viable; rat catheter and Spider biofilm induced
orf19.7580	orf19.7580	44.31	55.37	55.24	73.4	0.255101951	0.926807	Ortholog of C. dubliniensis CD36 : Cd36_35095, C. parapsilosis CDC317 : CPAR2_200710, Candida tenuis NRRL Y-1498 : CANTEDRAFT_112338 and Debaryomyces hansenii CBS767 : DEHA2G06094g

orf19.2018	orf19.2018	53.58	55.31	47.74	49.47	-0.128742784	0.998825	Protein with a predicted DnaJ chaperone domain and a CSL-type zinc finger; Spider biofilm induced
orf19.880	orf19.880	50.71	55.3	50.7	46.8	-0.090955326	0.998825	Ortholog(s) have role in cargo loading into COPII-coated vesicle and COPII vesicle coat, cytosol, mating projection tip localization
orf19.6011	SIN3	62.98	55.3	69.68	63.21	0.119643838	0.998825	Protein similar to S. cerevisiae Sin3p (transcriptional corepressor involved in histone deacetylase recruitment); has paired amphipathic helix PAH1 domain; interacts with ScOpi1p, not CaOpi1p; transposon mutation affects filamentous growth
orf19.2529	orf19.2529	53.42	55.27	66.98	75.36	0.284072673	0.870631	Ortholog of S. cerevisiae Gpa15; involved in the synthesis of glycosylphosphatidylinositol (GPI) anchors; shuman Pig-H, which is also involved in GPI assembly; Spider biofilm induced
orf19.6718	orf19.6718	46.91	55.26	54.88	56.62	0.085893401	0.998825	Has domain(s) with predicted sequence-specific DNA binding activity
orf19.199	orf19.199	56.48	55.25	66.97	58.11	0.115265105	0.998825	Protein of unknown function; hyphal-induced expression; upregulated in a cyr1 null mutant
orf19.6214	ATC1	61.62	55.24	79.92	76.77	0.313386824	0.817443	Cell wall acid trehalase; catalyzes hydrolysis of the disaccharide trehalose; similar to S. cerevisiae vacuolar acid trehalase (Ath1p); Hap43p-repressed gene
orf19.2347	MNN2	51.81	55.2	45.28	46.8	-0.162519669	0.998825	Alpha-1,2-mannosyltransferase; similar to S. cerevisiae Mnn2; role in cell wall integrity, temperature sensitivity; iron utilization in low iron; Tn mutation affects filamentous growth; filament induced; increased chitin exposes beta-glucan
orf19.1864	orf19.1864	58.89	55.19	53.52	51.28	-0.094817373	0.998825	Ortholog(s) have role in ribosome-associated ubiquitin-dependent protein catabolic process and RQC complex, cytosol, cytosolic large ribosomal subunit localization

orf19.2474	PRC3	57.68	55.18	63.24	58.84	0.071842364	0.998825	Putative carboxypeptidase Y precursor; transcript regulated by Nrg1 and Mig1; regulated by Gcn2 and Gcn4
orf19.4167	orf19.4167	53.39	55.17	70.15	75.15	0.307223006	0.842252	Protein of unknown function; Spider biofilm induced
orf19.5001	CUP2	55.69	55.15	52.1	40.72	-0.191639312	0.998825	Putative copper-binding transcription factor; required for normal resistance to copper; activates transcription of metallothionein genes; Hap43-repressed; Spider biofilm induced
orf19.3298	CCH1	43.66	55.02	37.16	38.92	-0.267153359	0.921805	Voltage-gated Ca2+ channel of the high affinity calcium uptake system; roles in thigmotropism, establishment of galvanotropism; transcript regulated by Nrg1 and Mig1; flow model biofilm repressed
orf19.4021	orf19.4021	59.15	54.98	61.89	59.58	0.06310081	0.998825	Ortholog of C. dubliniensis CD36 : Cd36_54870, C. parapsilosis CDC317 : CPAR2_100250, Debaryomyces hansenii CBS767 : DEHA2D13992g and Pichia stipitis Pignal : PICST_40400
orf19.1228	HAP2	45.49	54.92	46.91	53.39	-0.009929976	0.998825	CCAAT-binding transcription factor; regulates low-iron induction of FRP1; in these conditions CBF comprises Hap43 and probably Hap2 and Hap3; possibly essential, disruptants not obtained by UAU1 method; Cap1-dependent induction in low iron
orf19.5861.1	orf19.5861.1	47.06	54.88	60.44	42.64	-0.004214244	0.998825	Protein of unknown function; Spider biofilm induced
orf19.446	orf19.446	55.3	54.86	57.69	45.62	-0.069441218	0.998825	Protein of unknown function; constitutive expression independent of MTL or white-opaque status; upregulated in a cyr1 mutant
orf19.1374	orf19.1374	77.19	54.83	61.79	67.83	-0.02085376	0.998825	Ortholog of C. dubliniensis CD36 : Cd36_23840, Candida tenuis NRRL Y-1498 : CANTEDRAFT_104074, Debaryomyces hansenii CBS767 : DEHA2B13310g and Pichia stipitis Pignal : PICST_58235

orf19.3679	orf19.3679	59.3	54.8	58.58	57.06	0.009465573	0.998825	Putative protein of unknown function; stationary phase enriched protein
orf19.578	orf19.578	69.39	54.76	58.71	55.01	-0.09660953	0.998825	Ortholog(s) have Rab GTPase activator activity and role in actin filament organization, endocytosis, exocytosis, regulation of GTPase activity, regulation of protein localization
orf19.2368	orf19.2368	53.23	54.72	35.39	48.72	-0.25471976	0.932999	Predicted component of U5 snRNP; Spider biofilm induced
orf19.1953	orf19.1953	70.73	54.67	72.22	55.02	-0.000890608	0.998825	Predicted protein of unknown function; merged with orf19.1952 in Assembly 21
orf19.2688	NAN1	59.36	54.65	45.99	37.43	-0.330809464	0.824505	Putative U3 snoRNP protein; Hap43p-induced gene; physically interacts with TAP-tagged Nop1p
orf19.7012	orf19.7012	68.46	54.6	61.56	61.9	0.001684611	0.998825	Ortholog(s) have structural constituent of ribosome activity and mitochondrial small ribosomal subunit localization
orf19.6195	orf19.6195	57.38	54.55	58.21	48.95	-0.058114138	0.998825	Ortholog(s) have 3'-5'-exoribonuclease activity
orf19.1448.1	orf19.1448.1	40.15	54.52	62.71	66.45	0.302804105	0.8593	Ortholog(s) have plus-end-directed microtubule motor activity
orf19.5680	orf19.5680	55.22	54.5	53.86	42.23	-0.150669456	0.998825	Protein with predicted hydrolase domains; similar to a universal stress protein family protein; possibly essential, disruptants not obtained by UAU1 method; Spider biofilm repressed

orf19.2516	orf19.2516	73.04	54.49	70.73	77.56	0.168721479	0.998825	Has domain(s) with predicted role in cell redox homeostasis
orf19.4753	PFK26	50.78	54.49	62.94	67.12	0.216363848	0.957711	Putative 6-phosphofructo-2-kinase; protein repressed during the mating process
orf19.4816	orf19.4816	56.45	54.49	35.88	28.69	-0.522091575	0.415549	Protein of unknown function; induced by nitric oxide
orf19.4798	orf19.4798	57.66	54.44	62.1	59.45	0.072772332	0.998825	Ortholog(s) have role in establishment of protein localization to plasma membrane and mitochondrion, plasma membrane localization
orf19.5010	DIM1	66.95	54.43	53.53	46.6	-0.196361904	0.998825	Putative 18S rRNA dimethylase; predicted role in rRNA modification and processing; Hap43-induced; likely to be essential for growth based on insertional mutagenesis strategy; F-12/CO2 early biofilm induced
orf19.532	RBR2	62.53	54.41	58.13	48.48	-0.112509033	0.998825	Cell wall protein; expression repressed by Rim101; transcript regulated upon white-opaque switching; repressed by alpha pheromone in SpiderM medium; macrophage-induced gene
orf19.6943	orf19.6943	74.82	54.4	70.92	56.71	-0.016253919	0.998825	Predicted ORF from Assembly 19; removed from Assembly 20; subsequently reinstated in Assembly 21 based on comparative genome analysis
orf19.2970	LYS2	52.68	54.32	69.29	67.56	0.254180824	0.914829	Heterodimeric alpha-aminoadipate reductase large subunit; lysine biosynthesis; predicted binding sites for AMP and alpha-aminoadipate; inhibited by lys or thialysine; regulated by Gcn2 and Gcn4; Spider biofilm induced, flow model repressed
orf19.3977	orf19.3977	76.51	54.32	67.36	54.84	-0.083019737	0.998825	Protein with a role in translation; flow model biofilm repressed

orf19.3966	CRH12	43.39	54.31	43.64	54.89	0.011242892	0.998825	CRH family cell wall protein; transcript regulated by Nrg1 and Tup1; alkaline induced by Rim101; repressed during cell wall regeneration; flow model biofilm induced
orf19.376	orf19.376	75.9	54.28	57	46.2	-0.250277365	0.929146	Protein of unknown function; Hap43-repressed; Spider biofilm induced
orf19.7427	orf19.7427	64.3	54.26	54.29	54.41	-0.100242801	0.998825	Ortholog(s) have Golgi apparatus, fungal-type vacuole membrane localization
orf19.6029	ROT1	57.44	54.25	51.46	50.46	-0.106441831	0.998825	Similar to S. cerevisiae Rot1p, which is involved in cell wall 1,6-beta-glucan biosynthesis; has predicted transmembrane region; possibly an essential gene, disruptants not obtained by UAU1 method
orf19.3250	orf19.3250	64.81	54.22	58.7	65.21	0.037577594	0.998825	Ortholog(s) have RNA binding activity, role in mRNA cis splicing, via spliceosome and U1 snRNP, U2-type prespliceosome localization
orf19.3008	COQ4	48.36	54.17	75.35	78.03	0.42444554	0.561982	Protein with a putative role in coenzyme Q biosynthesis; transcriptionally induced by interaction with macrophage; Hap43p-repressed gene
orf19.6060	GCN20	57.98	54.11	60.85	56.16	0.036645018	0.998825	YEF3-subfamily ABC family protein, predicted not to be a transporter
orf19.2245	YPT72	56.48	54.11	76.58	75.07	0.341410087	0.752654	Vacuolar Rab small monomeric GTPase involved in vacuolar biogenesis; involved in filamentous growth and virulence
orf19.7285	orf19.7285	56.49	54.08	53.27	49.15	-0.087682769	0.998825	Ortholog(s) have role in mRNA cleavage, mRNA polyadenylation, snoRNA 3'-end processing, termination of RNA polymerase II transcription, exosome-dependent, termination of RNA polymerase II transcription, poly(A)-coupled

orf19.3775	SSK2	55.37	54.08	64.79	61.6	0.152563034	0.998825	MAP kinase kinase kinase (MAPKKK); regulates Hog1 activation and signaling; repressed by ciclopirox olamine
orf19.3499	orf19.3499	44.67	53.98	124.75	172.71	1.223219901	2.88E-05	Secreted potein; Hap43-repressed; fluconazole-induced; regulated by Tsa1, Tsa1B under H2O2 stress conditions; induced by Mnl1p under weak acid stress; Spider biofilm induced
orf19.4133	orf19.4133	65.73	53.97	50.07	35.92	-0.347537904	0.789669	Protein wth a predicted role in transcription from RNA polymerase II promoters; Spider biofilm induced
orf19.7412	MUB1	49.1	53.95	51.23	46.02	-0.072109346	0.998825	Predicted protein required for ubiquitination; role in meiosis, regulation of cell budding in S. cereviae; Spider biofilm induced
orf19.3887	orf19.3887	57.08	53.89	62.2	54.09	0.036193665	0.998825	Ortholog(s) have cytoplasm, nucleus localization
orf19.2969	RAD16	69.39	53.88	54.69	41.66	-0.254264839	0.924887	Ortholog of S. cerevisiae Rad16; a protein that recognizes and binds damaged DNA; flucytosine induced; rat catheter and Spider biofilm induced
orf19.6180	orf19.6180	56.18	53.88	65.98	65.1	0.17715654	0.998825	Protein similar to Pichia anomala YDL054c and S. cerevisiae Ydl054cp; transmembrane regions predicted; Hap43p-repressed gene
orf19.3813	orf19.3813	56.02	53.77	55.62	53.37	-0.018920475	0.998825	Ortholog of C. dubliniensis CD36 : Cd36_44340, C. parapsilosis CDC317 : CPAR2_302240, Candida tenuis NRRL Y-1498 : CANTEDRAFT_105331 and Debaryomyces hansenii CBS767 : DEHA2E03454g
orf19.2186	SEH1	56.45	53.73	48.57	50.7	-0.106614348	0.998825	Ortholog(s) have Seh1-associated complex, cytosol, fungal-type vacuole membrane, nuclear membrane, nuclear pore outer ring localization

orf19.3240	ERG27	53.73	53.71	65.49	76.28	0.276749007	0.878538	3-Keto sterol reductase of ergosterol biosynthesis; acts in C-4 sterol demethylation with Erg25p and Erg26p; possible drug target, essential for viability; functional homolog of S. cerevisiae Erg27p
orf19.3116	EXM2	58.75	53.7	50.15	43.79	-0.198460468	0.998825	Putative U1 snRNP complex compotent; decreased transcription is observed upon fluphenazine treatment
orf19.469	HST7	46.24	53.66	49.77	43.37	-0.084928605	0.998825	MAP kinase kinase involved in mating and hyphal growth signal transduction pathways; phosphorylates Cek1p; wild-type virulence in mouse systemic infection; functional homolog of S. cerevisiae Ste7p; mutants are hypersensitive to caspofungin
orf19.5676	orf19.5676	58.35	53.62	57.27	42.35	-0.137878602	0.998825	Ortholog(s) have cytosol, nucleus localization
orf19.317	PNP1	54.66	53.62	73.3	70.41	0.282464156	0.870631	Purine nucleoside phosphorylase; metabolizes inosine and guanosine nucleosides; in the nicotinamide riboside salvage pathway; Spider biofilm repressed
orf19.2305	orf19.2305	63.28	53.61	68.46	65.22	0.129538038	0.998825	Ortholog(s) have tubulin binding activity, role in tubulin complex assembly and prefoldin complex localization
orf19.6973	orf19.6973	54.72	53.58	57.43	49.99	-0.029573177	0.998825	ATP-dependent LON protease family member; Hap43-repressed gene; regulated by Gcn2 and Gcn4; Spider biofilm induced
orf19.6211	orf19.6211	51.29	53.51	28.54	27.07	-0.604652347	0.270212	Protein of unknown function; protein newly produced during adaptation to the serum
orf19.511	orf19.511	48.27	53.5	51.8	50.73	0.009692112	0.998825	Ortholog(s) have ribosylnicotinamide kinase activity, role in NAD biosynthesis via nicotinamide riboside salvage pathway, nicotinamide riboside metabolic process and cytosol, nucleus localization

orf19.6920	orf19.6920	63.15	53.45	35.55	19.29	-0.708372864	0.142008	Protein of unknown function; induced during chlamydospore formation in both C. albicans and C. dubliniensis; Hap43-induced gene; Spider biofilm induced; F-12/CO2 early biofilm induced
orf19.3496	CHC1	54.02	53.45	83.21	76.39	0.420192894	0.557651	Clathrin heavy chain; subunit of the major coat protein; role in intracellular protein transport and endocytosis; flow model and rat catheter biofilm repressed
orf19.417	MTM1	49.49	53.41	45.06	47.18	-0.113371948	0.998825	Ortholog(s) have metallochaperone activity, role in manganese ion transport and mitochondrion localization
orf19.3627	orf19.3627	50.5	53.41	55.67	52.46	0.028672028	0.998825	Ortholog of C. dubliniensis CD36 : Cd36_22640, C. parapsilosis CDC317 : CPAR2_406910, Candida tenuis NRRL Y-1498 : CANTEDRAFT_104937 and Debaryomyces hansenii CBS767 : DEHA2D02706g
orf19.1543	OPI1	56.72	53.4	53.42	45.95	-0.118252249	0.998825	Leucine zipper transcription factor; repressor of INO1 involved in inositol biosynthesis; functional homolog of S. cerevisiae Opi1; has putative Opi1-Sin3 interaction domain; interacts with ScSin3, but not CaSin3
orf19.3844	MRP8	49.36	53.4	66.05	53.56	0.156976737	0.998825	Mitochondrial ribosomal protein; ortholog of S. cerevisiae Mrp8; transcript induced in hyphal form; mutant is viable; flow model and rat catheter biofilm repressed
orf19.5543	orf19.5543	56.7	53.34	57.59	47.73	-0.048725589	0.998825	Ortholog(s) have cell division site, cytosol localization
orf19.7662	orf19.7662	68.08	53.29	54.47	51.65	-0.145360565	0.998825	Ortholog(s) have RNA polymerase II core binding activity, role in mRNA 3'-end processing, negative regulation of transposition, RNA-mediated and nuclear chromatin, site of double-strand break localization
orf19.3649	orf19.3649	55.27	53.26	52.03	46.91	-0.099180395	0.998825	Ortholog(s) have adenyl-nucleotide exchange factor activity, role in cytoplasm-associated proteasomal ubiquitin-dependent protein catabolic process, cytoplasmic translation and cytosolic ribosome, nucleus localization

orf19.3129	orf19.3129	57.62	53.26	58.28	45.14	-0.087896993	0.998825	Putative chromatin remodelling complex protein; heterozygous null mutant displays sensitivity to virgineone; Spider biofilm repressed
orf19.729.1	RGD3	54.24	53.22	52.32	50.5	-0.048457961	0.998825	Putative Rho GTPase activating protein; fungal-specific (no human or murine homolog)
orf19.6621	MHP1	51.7	53.2	56.81	49.72	0.00879317	0.998825	Protein similar to S. cerevisiae Mhp1p, which is involved in microtubule stabilization; transposon mutation affects filamentous growth; possibly transcriptionally regulated upon hyphal formation; possibly an essential gene (by UAU1 method)
orf19.5553	orf19.5553	52.84	53.19	54.17	47.52	-0.049892058	0.998825	Ortholog(s) have methionine-R-sulfoxide reductase activity, role in cellular response to oxidative stress and cytoplasm, nucleus localization
orf19.6676	orf19.6676	63.62	53.18	37.87	63.3	-0.14909056	0.998825	Has domain(s) with predicted diphthine synthase activity and role in peptidyl-diphthamide biosynthetic process from peptidyl-histidine
orf19.4117	orf19.4117	60.34	53.17	60.57	54.83	0.018618384	0.998825	Ortholog of C. dubliniensis CD36 : Cd36_20450, C. parapsilosis CDC317 : CPAR2_104160, Candida tenuis NRRL Y-1498 : CANTEDRAFT_95516 and Debaryomyces hansenii CBS767 : DEHA2F05192g
orf19.1701	RKI1	55.84	53.17	59.91	51.7	0.018254593	0.998825	Ortholog(s) have ribose-5-phosphate isomerase activity, role in pentose-phosphate shunt, pyridoxine biosynthetic process and cytoplasm, nucleus localization
orf19.3328	orf19.3328	54.5	53.16	72.91	64.46	0.243832809	0.930538	Putative transcription factor; S. cerevisiae ortholog Hot1 is required for the transient induction of glycerol biosynthetic genes GPD1 and GPP2 in response to high osmolarity; filament induced
orf19.2621	orf19.2621	36.15	53.14	34.99	37.09	-0.210025588	0.998825	Ortholog(s) have role in mRNA splicing, via spliceosome and U1 snRNP, U2 snRNP, U2-type prespliceosome, U4/U6 x U5 tri-snRNP complex, U5 snRNP, cytosol localization

orf19.3127	CZF1	38.56	53.13	64.64	77.53	0.44543757	0.546245	Transcription factor; regulates white-opaque switch; hyphal growth regulator; expression in S. cerevisiae causes dominant-negative inhibition of pheromone response; required for yeast cell adherence to silicone; Spider biofilm induced
orf19.3845	FGR3	50.7	53.13	41.92	42.02	-0.222936913	0.964275	Protein lacking an ortholog in S. cerevisiae; transposon mutation affects filamentous growth
orf19.544	RPR2	57.34	53.12	52.07	57.16	-0.016945307	0.998825	Ortholog(s) have ribonuclease P activity, role in intronic box C/D snoRNA processing and cytosol, nucleolar ribonuclease P complex localization
orf19.6230	orf19.6230	57.73	53.09	50.74	45.31	-0.15850786	0.998825	Ortholog(s) have enzyme regulator activity, pyrophosphatase activity
orf19.4698	PTC8	57.05	53.04	22.41	30.94	-0.688402022	0.163742	Predicted type 2C protein phosphatase, ser/thr-specific; required for hyphal growth; transcript induced by stress; flow model biofilm induced; Spider biofilm induced
orf19.4843	orf19.4843	53.95	53.01	23.17	22.32	-0.80606174	0.064252	Putative iron/copper reductas; involved in iron homeostasis; rat catheter and Spider biofilm induced
orf19.2876	CBF1	48.48	53.01	36.77	28.95	-0.419457848	0.645919	Transcription factor; binds ribosomal protein gene promoters and rDNA locus with Tbf1; regulates sulfur starvation-response, respiratory, glycolytic genes; does not bind to centromeres as does S. cerevisiae Cbf1; Spider biofilm repressed
orf19.419	orf19.419	55.22	52.98	53.49	53.15	-0.028151816	0.998825	Protein of unknown function; flow model biofilm induced; Spider biofilm induced
orf19.3086	SEC10	54.1	52.91	72.7	61.66	0.23661994	0.938642	Ortholog(s) have role in Golgi to plasma membrane transport, cytokinetic cell separation, exocytosis and cell cortex of cell tip, cell division site, cytosol, exocyst, mating projection tip, mitotic spindle pole body, nucleus localization

orf19.7185	orf19.7185	52.16	52.91	56.49	49.58	-0.000728044	0.998825	Ortholog of C. dubliniensis CD36 : Cd36_73630, C. parapsilosis CDC317 : CPAR2_805330, Candida tenuis NRRL Y-1498 : CANTEDRAFT_119330 and Debaryomyces hansenii CBS767 : DEHA2G16544g
orf19.4120	LAS1	54.93	52.9	40.46	45.59	-0.236328271	0.946319	Putative bud formation and morphogenesis protein; mutation confers hypersensitivity to 5-fluorocytosine (5-FC), 5-fluorouracil (5-FU); macrophage-induced; Spider biofilm induced
orf19.3045	orf19.3045	61.38	52.8	50.86	45.14	-0.185835904	0.998825	Predicted aldehyde dehydrogenase domain; virulence-group-correlated expression
orf19.5535	orf19.5535	56	52.78	66.18	65	0.186923187	0.998825	Predicted membrane transporter, member of the anion:cation symporter (ACS) family, major facilitator superfamily (MFS)
orf19.7207	DOA4	52.27	52.78	47.37	44.85	-0.143713461	0.998825	Ortholog of S. cerevisiae Doa4;, a ubiquitin hydrolase involved in recycling ubiquitin from proteasome-bound ubiquitinated intermediates; oxidative stress-induced via Cap1; mutants are viable
orf19.2721	orf19.2721	53.34	52.78	37.82	48.47	-0.215313246	0.983377	Ortholog(s) have role in ascospore formation, regulation of mitotic cell cycle, regulation of translation and cytosol, nucleus localization
orf19.414	RSM22	60.52	52.7	46.26	43	-0.260927576	0.918389	Predicted mitochondrial small ribosomal subunit; rat catheter and Spider biofilm induced
orf19.1628	LAP41	51.02	52.68	83.49	83.33	0.498738679	0.372168	Putative aminopeptidase yscI precursor; mutant is viable; protein present in exponential and stationary growth phase yeast cultures; Spider biofilm repressed
orf19.6140	FRE30	49.8	52.65	64.83	58.87	0.183829743	0.998825	Protein with similarity to ferric reductases; downregulated in response to amphotericin B, estradiol, or ciclopirox olamine, and upregulated by interaction with macrophage; un-merged from orf19.6139 in a revision of Assembly 21

orf19.2237.1	orf19.2237.1	60.6	52.61	49.31	49.92	-0.15327087	0.998825	Ortholog of Slx9 required for pre-rRNA processing; associated with 90S pre-ribosome and 43S small ribosomal subunit precursor, interacts with U3 snoRNA in S. cerevisiae; gene has intron; Spider biofilm induced
orf19.7386	orf19.7386	56.49	52.57	65.62	65.8	0.195329499	0.998825	Ortholog(s) have ribosome binding activity
orf19.4899	GCA1	35.16	52.55	95.62	97.65	0.829357341	0.023763	Extracellular/plasma membrane-associated glucoamylase; expressed in rat oral infection; regulated by carbohydrates, pH, galactose; promotes biofilm matrix formation; flow model biofilm induced; Bcr1 repressed in RPMI a/a biofilms
orf19.6259	orf19.6259	62.44	52.55	49.09	48.89	-0.172780904	0.998825	Exosome non-catalytic core component; involved in 3'-5' RNA processing and degradation in the nucleus and cytoplasm; Spider biofilm induced
orf19.6420	PGA13	30.91	52.55	88.15	169.69	0.716036416	0.181137	GPI-anchored cell wall protein involved in cell wall synthesis; required for normal cell surface properties; induced in oralpharyngeal candidasis; Spider biofilm induced; Bcr1-repressed in RPMI a/a biofilms
orf19.5340	orf19.5340	52.7	52.46	52.56	46.91	-0.06011176	0.998825	Putative Rab GTPase activator; role in ER to Golgi vesicle-mediated transport; Spider biofilm induced
orf19.243	OXR1	55.34	52.42	67.13	68.55	0.247454938	0.924887	Ortholog(s) have role in cellular response to oxidative stress and cytosol, mitochondrion, nucleus localization
orf19.1528	orf19.1528	74.33	52.38	55.99	54.01	-0.148099423	0.998825	Putative mitochondrial RNA polymerase specificity factor; possibly an essential gene, disruptants not obtained by UAU1 method
orf19.5229	orf19.5229	59.56	52.34	53.32	48.85	-0.105265158	0.998825	Ortholog(s) have 3'-5'-exoribonuclease activity, endoribonuclease activity, tRNA binding activity

orf19.3470	orf19.3470	62.63	52.3	54.71	46.42	-0.143151812	0.998825	Putative flavodoxin; similar to S. cerevisiae Tyw1, an iron-sulfur protein required for synthesis of wybutosine modified tRNA; predicted Kex2p substrate; Spider biofilm induced
orf19.1536	orf19.1536	55.02	52.28	57.72	45.53	-0.04012906	0.998825	Putative vacuolar transporter; Hap43-induced; required for normal filamentous growth; mRNA binds She3 and is localized to hyphal tips
orf19.6052	CNS1	70.2	52.27	52.94	48.93	-0.192873201	0.998825	Putative co-chaperone; Hap43p-induced gene; mutation confers hypersensitivity to radicicol
orf19.7095	orf19.7095	53.73	52.27	51.67	55.15	0.001551598	0.998825	Ortholog(s) have endoplasmic reticulum localization
orf19.4382	orf19.4382	62.2	52.25	67.47	44.75	-0.029561463	0.998825	Ortholog(s) have role in retrograde vesicle-mediated transport, Golgi to ER and COPI vesicle coat, cytosol, nucleus localization
orf19.4188	NMD5	56.2	52.21	44.64	46.96	-0.170987604	0.998825	Karyopherin; carrier protein involved in nuclear import of proteins; repressed in core stress response; Hap43-induced; Spider biofilm induced
orf19.5224	PKH2	60.02	52.2	62.51	63.52	0.126056762	0.998825	Putative serine/threonine protein kinase; predicted role in sphingolipid-mediated signaling pathway that controls endocytosis; mRNA binds She3 and is localized to hyphal tips
orf19.7586	CHT3	44.36	52.19	25.33	22.27	-0.655676858	0.218547	Major chitinase; secreted; functional homolog of S. cerevisiae Cts1p; 4 N-glycosylation motifs; possible O-mannosylation; putative signal peptide; hyphal-repressed; farnesol upregulated in biofilm; regulated by Efg1p, Cyr1p, Ras1p
orf19.3859	orf19.3859	61.36	52.19	64.7	61.17	0.107568362	0.998825	Putative microsomal beta-keto-reductase; transcript upregulated by treatment with ciclopirox olamine; induced by alpha pheromone in SpiderM medium; regulated by Sef1, Sfu1, and Hap43

orf19.458	orf19.458	63.96	52.18	66.3	66.41	0.131259449	0.998825	Ortholog(s) have protein transmembrane transporter activity and role in chaperone-mediated protein complex assembly, mitochondrial respiratory chain complex III assembly, protein insertion into mitochondrial membrane from inner side
orf19.5486.1	SMD2	38.4	52.18	54.45	52.63	0.160473874	0.998825	Putative Core Sm protein; Hap43p-induced gene; flucytosine induced
orf19.4342	SUT1	44.79	52.15	44.15	59.18	0.052542448	0.998825	Zn2Cys6 transcription factor involved in sterol uptake; flow model biofilm induced; Spider biofilm repressed
orf19.2178	MRS4	59.25	52.09	67.37	55.79	0.098688183	0.998825	Mitochondrial carrier family member, involved in iron homeostasis; putative membrane transporter localized to the mitochondrial membrane; transcription under control of Atf2p; regulated by Sef1p, Sfu1p, and Hap43p
orf19.5030	DOS2	49.46	52.05	59.4	57.28	0.133643604	0.998825	Protein of unknown function; induced during planktonic growth
orf19.1888	orf19.1888	50.15	52.03	45.85	47.72	-0.091631456	0.998825	Putative nicotinamide riboside hydrolase; cleaves N-glycosidic bonds in nucleosides; pyrimidine salvage and nicotinamide riboside salvage pathways; Spider biofilm induced
orf19.3644	orf19.3644	58.36	51.97	51.77	45.56	-0.127948999	0.998825	Protein of unknown function; Cyr1-repressed; rat catheter and Spider biofilm induced
orf19.600	TRK1	46.86	51.94	42.86	41.74	-0.161573707	0.998825	Potassium transporter; mediates K+ and Cl- influx; role in sensitivity to cationic antimicrobial peptides, not by direct uptake, possibly related to membrane permeability; contains 5' UTR intron; Spider biofilm induced
orf19.6867	orf19.6867	48.79	51.85	64.54	94.2	0.47168323	0.470755	Protein with a predicted cytochrome b5-like heme/steroid binding domain; repressed by alpha pheromone in SpiderM medium

orf19.3131	OYE32	48.37	51.8	57.57	48.79	0.057637903	0.998825	NAD(P)H oxidoreductase family protein; induced by nitric oxide, amphotericin B, oxidative stress via Cap1; upregulation associated with MDR1 overexpression or benomyl treatment; macrophage-downregulated protein; Spider biofilm induced
orf19.6263	orf19.6263	49.92	51.77	49.26	46.57	-0.071428562	0.998825	Predicted MFS membrane transporter; member of the monocarboxylate porter (MCP) family; Spider biofilm induced
orf19.5348	TPS3	47.76	51.76	51.15	56.35	0.060269507	0.998825	Predicted trehalose-phosphate synthase regulatory subunit; regulated by Efg1; regulated by Tsa1, Tsa1B under H2O2 stress conditions; flow model biofilm induced (reports differ)
orf19.4157	SPS20	56.47	51.68	89.03	94.62	0.579884115	0.206743	Peroxisomal 2,4-dienoyl-CoA reductase; stationary phase enriched protein; Spider biofilm induced
orf19.5066	orf19.5066	59.89	51.55	52.38	53.07	-0.074465615	0.998825	Putative pre-60S pre-ribosomal particle subunit; essential gene; S. cerevisiae ortholog RRP17 is essential; Hap43p-induced; rat catheter biofilm induced
orf19.3659	orf19.3659	40.4	51.53	54.56	50.55	0.129804444	0.998825	Putative CTD phosphatase; role in dephosphorylation of RNA polymerase II C-terminal domain, transcription from RNA polymerase II promoter; flow model biofilm induced
orf19.2177	orf19.2177	42.82	51.51	27.5	26.41	-0.545838585	0.399561	Sef1p-, Sfu1p-, and Hap43p-regulated gene; overlaps IFM3/orf19.2176
orf19.5849	CWT1	52.16	51.5	50.1	39.21	-0.157699263	0.998825	Zn2Cys6 transcription factor involved in negative regulation of nitrosative stress response; mutant has cell wall defects; transcription increased at stationary phase; has predicted PAS domain; similar to S. cerevisiae Rds2p
orf19.2324	UBA4	52.51	51.5	46.57	49.66	-0.079013232	0.998825	Putative ubiquitin activating protein; Hap43-repressed; induced by prostaglandins; clade-associated gene expression

orf19.4580	orf19.4580	53.67	51.46	43.69	45.49	-0.175106191	0.998825	Protein of unknown function; Hap43-repressed gene
orf19.5342.1	MTR2	67.37	51.31	57.59	57.57	-0.024186217	0.998825	Nuclear export protein; member of NTF2 family; monomeric in solution; interacts with Mex67p via the NTF2-like domain
orf19.1299	RPN6	61.6	51.29	53.03	47.02	-0.134373006	0.998825	Putative 26S proteasome subunit; Hap43p-induced gene; regulated by Gcn2p and Gcn4p
orf19.7242	NCR1	49.65	51.29	68.55	63.22	0.272255685	0.888717	Putative vacuolar membrane protein; predicted role in sphingolipid metabolism; transcript regulated by Nrg1 and Mig1; induced by prostaglandins
orf19.4192.1	orf19.4192.1	50.01	51.26	60.44	79.84	0.335665311	0.795378	Ortholog of C. parapsilosis CDC317 : CPAR2_602880, C. dubliniensis CD36 : Cd36_60590, Candida tenuis NRRL Y-1498 : CANTEDRAFT_115034 and Debaryomyces hansenii CBS767 : DEHA2F15334g
orf19.5255	PXA2	58.29	51.25	62	44.48	-0.039814592	0.998825	Putative peroxisomal, half-size adrenoleukodystrophy protein (ALD or ALDp) subfamily ABC transporter; Gcn4p-regulated
orf19.133	orf19.133	34.72	51.11	47.22	62.3	0.221870237	0.976613	Protein of unknown function; induced by nitric oxide independent of Yhb1p
orf19.2106	orf19.2106	51.94	51.11	51	45.58	-0.071008416	0.998825	Putative protein of unknown function; Hap43p-repressed gene
orf19.6820	orf19.6820	53.15	51.08	56.84	38.95	-0.0928371	0.998825	Has domain(s) with predicted protein heterodimerization activity, role in DNA-dependent transcription, initiation and transcription factor TFIID complex localization

orf19.2016	ERG28	46.76	51.04	84.71	97.99	0.66968453	0.105087	Ortholog(s) have protein binding, bridging activity, role in ergosterol biosynthetic process and endoplasmic reticulum membrane localization
orf19.2604	orf19.2604	55.87	51	49.77	44.9	-0.130556358	0.998825	S. pombe ortholog SPAC2C4.06c is a predicted tRNA (cytosine-5-)-methyltransferase; Spider biofilm induced
orf19.2180	orf19.2180	51.01	50.99	56.75	49.17	0.028901965	0.998825	Ortholog(s) have calcium ion binding, zinc ion binding activity, role in axial cellular bud site selection, bipolar cellular bud site selection, regulation of COPII vesicle coating and cellular bud neck, cytosol, nucleus localization
orf19.6649	BRF1	50.65	50.97	54.53	45.14	-0.031230002	0.998825	Component of the general transcription factor for RNA polymerase III (TFIIIB); possibly an essential gene, disruptants not obtained by UAU1 method
orf19.6494	WHI3	42.41	50.96	39.77	39.14	-0.169151098	0.998825	Putative RNA binding protein; induced during infection of murine kidney, compared to growth in vitro; has murine homolog
orf19.5370	orf19.5370	59.43	50.95	90.06	83.4	0.488904154	0.382965	Ortholog(s) have fungal-type vacuole membrane localization
orf19.2123	orf19.2123	62.56	50.81	64.34	72.1	0.182773888	0.998825	Predicted ORF from Assembly 19; removed from Assembly 20; restored based on comparative genome analysis
orf19.828	orf19.828	55.59	50.8	62.34	57.08	0.102568901	0.998825	Putative ribosomal protein, large subunit, mitochondrial precursor; repressed by prostaglandins; Spider biofilm repressed
orf19.92	orf19.92	52.8	50.79	72.12	66.89	0.299847573	0.854173	Protein with a predicted thioredoxin-like domain; Hap43-repressed; induced by prostaglandins

orf19.4830	orf19.4830	47.13	50.79	61.05	72	0.310966166	0.846842	Ortholog of C. dubliniensis CD36 : Cd36_09110, C. parapsilosis CDC317 : CPAR2_803660, Candida tenuis NRRL Y-1498 : cten_CGOB_00140 and Debaryomyces hansenii CBS767 : DEHA2B12056g
orf19.1372	orf19.1372	55.75	50.76	92.02	97.11	0.621221666	0.144775	Protein of unknown function; transcript regulated by Mig1 and Tup1
orf19.4436	GPX3	48.1	50.74	57.04	49.84	0.068552736	0.998825	Putative glutathione peroxidase involved in Cap1p-dependent oxidative stress response, required for Cap1p oxidation in response to H2O2; planktonic growth-induced
orf19.2623	ECM22	41.83	50.73	40.79	34.65	-0.206548772	0.998825	Zn(II)2Cys6 transcription factor; rat catheter and Spider biofilm induced
orf19.132	orf19.132	51.75	50.69	50.68	46.5	-0.060436159	0.998825	Ortholog(s) have role in histone deacetylation, negative regulation of chromatin silencing at telomere, negative regulation of meiosis, positive regulation of stress-activated MAPK cascade
orf19.4121	orf19.4121	58.33	50.67	27.71	20.73	-0.7537006	0.098955	Predicted thioesterase/thiol ester dehydrase-isomerase; Spider biofilm induced
orf19.3482	orf19.3482	52.83	50.53	60.44	54.93	0.095151586	0.998825	Ortholog(s) have NAD+ diphosphatase activity, role in NADH metabolic process and cytosol, nucleus, peroxisome localization
orf19.6272	orf19.6272	61.83	50.52	51.39	47.2	-0.154528972	0.998825	Putative ubiquitin C-terminal hydrolase; regulated by Gcn2p and Gcn4p
orf19.4987	NUP49	53.13	50.48	54.09	51.44	0.010747527	0.998825	Nuclear pore protein

orf19.1589	orf19.1589	52.78	50.48	47.95	44.24	-0.123523374	0.998825	Putative transcription factor component of the core factor (CF) rDNA transcription factor complex; Spider biofilm induced
orf19.6066	orf19.6066	61.97	50.43	57.02	44.48	-0.115494428	0.998825	Hexadecenal dehydrogenase; involved in the conversion of sphingosine 1-phosphate breakdown product hexadecenal to hexadecenoic acid; Spider biofilm induced
orf19.3820	orf19.3820	48.43	50.37	18.94	41.74	-0.424037026	0.669881	Protein with DNA binding domain, an endonuclease domain; adjacent to CEN4, the centromere region of Chr 4; clade-associated gene expression
orf19.4225	LEU3	51.2	50.37	43.36	42.4	-0.181147617	0.998825	Zn(II)2Cys6 transcription factor; predicted regulator branched-c ofhain amino acid biosynthesis genes; alkaline induced; induced by Mnl1 under weak acid stress; required for yeast cell adherence to silicone substrate; Spider biofilm induced
orf19.6193	TAF145	53.52	50.31	51.3	47.12	-0.069755111	0.998825	Protein similar to S. cerevisiae Taf145p, a component of RNA polymerase II transcription factor TFIID; flucytosine repressed; likely to be essential for growth, based on an insertional mutagenesis strategy
orf19.899	orf19.899	49.42	50.3	73.37	69.33	0.37344664	0.692027	Ortholog of C. dubliniensis CD36 : Cd36_18040, C. parapsilosis CDC317 : CPAR2_211790, Candida tenuis NRRL Y-1498 : CANTEDRAFT_109928 and Debaryomyces hansenii CBS767 : DEHA2E21010g
orf19.1662	orf19.1662	53.65	50.25	52.97	58.99	0.069600875	0.998825	Ortholog(s) have structural constituent of ribosome activity and mitochondrial small ribosomal subunit localization
orf19.5167	IFM1	54.19	50.24	49.97	48.5	-0.058841514	0.998825	Putative mitochondrial translation initiation factor; transcript regulated by Nrg1, Mig1, and Tup1
orf19.1308	orf19.1308	57.84	50.16	62.24	56.91	0.093434664	0.998825	Predicted membrane transporter, member of the drug:proton antiporter (14 spanner) (DHA2) family, major facilitator superfamily (MFS)

orf19.6586	orf19.6586	60	50.15	12.61	8.6	-1.32810869	0.000209	Protein of unknown function; transcript induced by benomyl or in azole-resistant strain overexpressing MDR1; Ssn6 colony morphology-related regulation; induced by NO; Hap43-repressed; rat catheter and flow model biofilm induced
orf19.4499	RIM2	50.92	50.12	45.33	49.82	-0.069621693	0.998825	Putative mitochondrial carrier protein; induced by alpha pheromone in SpiderM medium; Spider biofilm induced
orf19.2298	WBP1	54.63	50.06	85.69	74.59	0.453961688	0.476823	Putative oligosaccharyltransferase subunit; Spider biofilm repressed
orf19.5941	orf19.5941	45.36	50.06	72.56	63.66	0.372845768	0.701828	Ortholog(s) have role in ER to Golgi vesicle-mediated transport and ER to Golgi transport vesicle, integral to Golgi membrane, integral to endoplasmic reticulum membrane localization
orf19.649	orf19.649	46.64	50.03	47.14	49.95	-0.008998938	0.998825	Ortholog of C. dubliniensis CD36 : Cd36_30530, C. parapsilosis CDC317 : CPAR2_203340, Candida tenuis NRRL Y-1498 : CANTEDRAFT_117008 and Debaryomyces hansenii CBS767 : DEHA2G24244g
orf19.2987	orf19.2987	49.81	50	59.82	55.55	0.144275866	0.998825	Ortholog(s) have role in cellular ion homeostasis, establishment or maintenance of cell polarity, mitochondrion inheritance, regulation of cardiolipin metabolic process and mitochondrial inner membrane, nucleus localization
orf19.2039	MSF1	56.98	50	62.24	59.16	0.121065637	0.998825	Putative phenylalanine-tRNA ligase; protein level decreases in stationary phase cultures; Hap43p-repressed gene
orf19.3310	orf19.3310	61.36	49.93	56.14	55.1	-0.007177462	0.998825	Protein of unknown function; Hap43-repressed; rat catheter and Spider biofilm induced
orf19.54	RHD1	45.99	49.93	16.73	10.24	-1.049759526	0.007975	Putative beta-mannosyltransferase required for the addition of beta-mannose to the acid-labile fraction of cell wall phosphopeptidomannan; 9-gene family memebr; regulated on yeast-hypha and white-opaque switches; Spider biofilm repressed

orf19.4792	orf19.4792	52.61	49.93	45.72	44.03	-0.144953888	0.998825	Protein with a regulator of G-protein signaling domain; Plc1-regulated; Spider biofilm induced; rat catheter biofilm repressed
orf19.5207	orf19.5207	58.12	49.92	45.44	39.01	-0.261206819	0.921805	Predicted diphthamide biosynthesis protein; Spider biofilm induced
orf19.6705	orf19.6705	48.41	49.92	47.11	42.88	-0.095466475	0.998825	Putative guanyl nucleotide exchange factor with Sec7 domain; required for normal filamentous growth; regulated by yeast-hyphal switch; filament induced; regulated by Nrg1, Tup1, Mob2, Hap43; mRNA binds She3; Spider biofilm induced
orf19.4046	orf19.4046	58.89	49.91	50.22	45.94	-0.138120287	0.998825	Putative transcription factor containing a Zn(2)-Cys(6) binuclear cluster
orf19.3516	orf19.3516	87.04	49.89	64.59	47.08	-0.21365629	0.976613	Protein of unknown function; Hap43-repressed; Spider biofilm repressed
orf19.3425	orf19.3425	62	49.86	69.02	59.08	0.133080297	0.998825	RING/FYVE/PHD zinc finger protein; Spider biofilm induced
orf19.1857	orf19.1857	52.96	49.82	62.42	51.66	0.095023515	0.998825	Putative L-azetidine-2-carboxylic acid acetyltransferase; mutants are viable
orf19.5552	orf19.5552	50.64	49.7	44.66	43.14	-0.14741263	0.998825	Putative transcriptional regulator of ribonucleotide reductase genes; Spider biofilm induced
orf19.4517	orf19.4517	54.68	49.65	37.41	39.97	-0.313898272	0.858075	Protein of unknown function; Hap43-induced gene

orf19.1394	orf19.1394	60.99	49.64	58.88	56.51	0.038858897	0.998825	Putative protein of unknown function; mutant is viable; protein level decreases in stationary phase cultures; Spider biofilm induced
orf19.4931	orf19.4931	52.42	49.64	52.51	45.74	-0.040473597	0.998825	Putative tRNA-Cys synthetase; induced by alpha pheromone in SpiderM medium; ribosomal subunits, translation factors, tRNA synthetases are downregulated upon phagocytosis by murine macrophage
orf19.1716	URA3	58.5	49.59	26.14	27.52	-0.67074132	0.182093	Orotidine-5'-phosphate decarboxylase; pyrimidine biosynthesis; gene used as genetic marker; decreased expression when integrated at ectopic chromosomal locations can cause defects in hyphal growth and virulence; Spider biofilm repressed
orf19.7272	orf19.7272	47.75	49.56	68.99	65.29	0.31952666	0.824505	Protein of unknown function; transcript detected on high-resolution tiling arrays
orf19.2546	TRP2	45.54	49.55	47.66	56.51	0.082569066	0.998825	Putative anthranilate synthase with a predicted role in tryptophan biosynthesis; regulated by Gcn2p and Gcn4p
orf19.3183	orf19.3183	68.85	49.49	64.13	62.68	0.072333135	0.998825	Protein with a role in insertion of tail-anchored proteins into the ER membrane; required for efficient mating, in shmoo formation and nuclear migration in the pre-zygote of S. cerevisiae; Hap43-repressed
orf19.2989	GOR1	51.81	49.42	43.09	42.34	-0.180742069	0.998825	Ortholog(s) have glyoxylate reductase activity, role in glyoxylate catabolic process and cytosol, extracellular region, mitochondrion, nucleus localization
orf19.5225	orf19.5225	53.16	49.42	51.96	60.94	0.099131839	0.998825	Ortholog of Candida albicans WO-1 : CAWG_00201
orf19.7478	orf19.7478	59.87	49.41	68.43	63.19	0.18746268	0.998825	Ortholog(s) have antioxidant activity, di-trans,poly-cis-decaprenylcistransferase activity, protein heterodimerization activity, trans-hexaprenyltranstransferase activity

orf19.4752	MSN4	47.46	49.39	22.9	25.15	-0.638341875	0.240375	Zinc finger transcription factor; similar to S. cerevisiae Msn4, but not a significant stress response regulator in C. albicans; partly complements STRE-activation defect of S. cerevisiae msn2 msn4 double mutant; flow model biofilm induced
orf19.6056	orf19.6056	51.12	49.38	44.4	43.52	-0.136552722	0.998825	Ortholog(s) have phosphatase activity, role in dephosphorylation and cytosol, nucleus localization
orf19.3569	orf19.3569	50.19	49.34	50.9	40.5	-0.084416077	0.998825	Protein of unknown function; S. cerevisiae YLR407W mutants have abnormal budding; constitutive expression independent of MTL or white-opaque status; Spider biofilm induced
orf19.5281	orf19.5281	47.07	49.31	90.38	85.94	0.648280942	0.127549	Predicted essential RNA-binding G protein; ortholog an effector of mating response pathway in S. cerevisiae; mainly associated with nuclear envelope and ER; flow model and Spider biofilm repressed
orf19.1441	orf19.1441	62.2	49.3	50.28	43.79	-0.177343011	0.998825	Ortholog of C. dubliniensis CD36 : Cd36_16230, C. parapsilosis CDC317 : CPAR2_213950, Candida tenuis NRRL Y-1498 : CANTEDRAFT_112720 and Debaryomyces hansenii CBS767 : DEHA2A01848g
orf19.3443	OYE2	48.32	49.26	42.99	40.67	-0.152079172	0.998825	Putative NAPDH dehydrogenase; induced by nitric oxide; Spider biofilm induced
orf19.7019	YML6	51.3	49.23	46.18	43.89	-0.114845271	0.998825	Putative mitochondrial ribosomal protein; induced upon adherence to polystyrene
orf19.3556	orf19.3556	48.44	49.2	45.8	44.29	-0.084472205	0.998825	Transportin or cytosolic karyopherin beta; Spider biofilm induced
orf19.4172	orf19.4172	45.35	49.05	35.29	28.06	-0.388157256	0.732318	Has domain(s) with predicted hydrolase activity and role in metabolic process

orf19.7612	CTM1	46.69	49	56.05	53.34	0.120028683	0.998825	Putative cytochrome c lysine methyltransferase; regulated by Gcn2 and Gcn4; transcript induced by Mnl1 under weak acid stress; early-stage flow model biofilm induced
orf19.7446	OPI3	50.41	48.97	77.95	89.77	0.563116512	0.251616	Phosphatidylethanolamine N-methyltransferase; phosphatidylcholine biosynthesis; downregulation correlates with clinical development of fluconazole resistance; amphotericin B, caspofungin repressed; Hap43-induced; Spider biofilm repressed
orf19.6500	ECM42	47.86	48.97	48.44	57.16	0.072974216	0.998825	Ornithine acetyltransferase; Gcn2, Gcn4-regulated; clade-specific gene expression; possibly essential gene, disruptants not obtained by UAU1 method; Spider biofilm induced
orf19.4476	orf19.4476	57.97	48.95	89.39	95.2	0.589893475	0.195854	Protein with a NADP-dependent oxidoreductase domain; transcript induced by ketoconazole; rat catheter and Spider biofilm induced
orf19.6217	PGA63	52.1	48.94	77.45	76.84	0.445304211	0.512672	Component COPII vesicle coat; required for vesicle formation in ER to Golgi transport; predicted GPI-anchor; flow model and Spider biofilm repressed
orf19.5727	orf19.5727	49.2	48.91	45.19	58.47	0.043832147	0.998825	Ortholog of C. dubliniensis CD36 : Cd36_64130, C. parapsilosis CDC317 : CPAR2_601230, Candida tenuis NRRL Y-1498 : CANTEDRAFT_115908 and Debaryomyces hansenii CBS767 : DEHA2E12540g
orf19.4620	TIM12	34.63	48.83	49.34	51.78	0.168884407	0.998825	Predicted component of the TIM22 complex, involved in protein import into mitochondrial inner membrane; flow model biofilm induced
orf19.6638	PTC4	63.87	48.79	49.04	51.18	-0.131456834	0.998825	Type PP2C serine/threonine phosphatase; localized to mitochondria; mutation causes sensitivity to sodium, potassium and azole drugs; decreased expression in hyphae compared to yeast-form cells
orf19.5710	orf19.5710	48.16	48.78	47.09	42.86	-0.084891951	0.998825	Nucleoporin component of central core of the nuclear pore complex; mRNA binds She3

orf19.6938	MEU1	68.97	48.75	69.88	61.86	0.112560571	0.998825	Putative methylthioadenosine phosphorylase; protein level decreases in stationary phase cultures
orf19.4579	ERV29	52.03	48.75	91.98	76.68	0.548090839	0.269313	Putative SURF4 family member; plasma membrane-localized; flow model biofilm repressed
orf19.4906	orf19.4906	52.92	48.66	52.93	49.88	0.0011947	0.998825	Putative adhesin-like protein; positively regulated by Tbf1; Spider biofilm induced
orf19.763	orf19.763	50.93	48.63	51.64	48.21	-0.009402338	0.998825	Small subunit of the heterodimeric cap binding complex; component of the spliceosomal commitment complex; Spider biofilm repressed
orf19.1759	PHO23	53.91	48.62	45.81	40.53	-0.17850485	0.998825	Ortholog(s) have methylated histone residue binding activity
orf19.6658	orf19.6658	57.07	48.51	60.33	55.53	0.085973141	0.998825	Stationary phase enriched protein; predicted ORF from Assembly 19; removed from Assembly 20; subsequently reinstated in Assembly 21 based on comparative genome analysis
orf19.5425	orf19.5425	55.44	48.48	51.87	40.27	-0.12367028	0.998825	Ortholog(s) have 3'-tRNA processing endoribonuclease activity, role in tRNA 3'-trailer cleavage, endonucleolytic and mitochondrion, nucleus localization
orf19.3752	RAD51	49.35	48.46	53.88	43.15	-0.010975305	0.998825	Protein involved in homologous recombination and DNA repair; flucytosine induced; slow growth and increased white-to-opaque switching frequency in null mutant
orf19.3803	MNN22	43.51	48.42	13.49	14.11	-1.011997918	0.011564	Alpha-1,2-mannosyltransferase; required for normal cell wall mannan; regulated by Tsa1, Tsa1B at 37 deg; repressed in core stress response; NO, Hog1 induced; confers sensitivity to cell wall perturbing agents; Spider biofilm repressed

orf19.5291	orf19.5291	55.89	48.42	45.38	51.81	-0.076570363	0.998825	Ortholog(s) have role in phospholipid biosynthetic process and endoplasmic reticulum localization
orf19.7089	PMR1	50.23	48.41	64.31	55.81	0.20051735	0.998825	Putative secretory pathway P-type Ca2+/Mn2+-ATPase; required for protein glycosylation and cell wall maintenance; required for hyphal tip oscillation in semisolid substrate; putative ortholog of S. cerevisiae PMR1
orf19.6083	orf19.6083	53.03	48.35	54.63	55.76	0.089737834	0.998825	Has domain(s) with predicted nucleic acid binding, nucleotide binding activity
orf19.6113	orf19.6113	46.11	48.32	47.85	53	0.064041309	0.998825	Protein of unknown function; transcript detected on high-resolution tiling arrays
orf19.6727	RIT1	55.34	48.21	47.67	48.64	-0.068420522	0.998825	Putative initiator tRNA methionine ribosyltransferase; fungal-specific (no human or murine homolog)
orf19.4407	orf19.4407	62.83	48.21	48.9	41.43	-0.219910861	0.969267	Protein of unknown function; Hap43-repressed gene
orf19.7244	orf19.7244	55.64	48.19	88.19	84.02	0.541327953	0.27463	Putative fumarylacetoacetate hydrolase; induced by nitric oxide independent of Yhb1; regulated by Sef1, Sfu1, Hap43; flow model biofilm induced
orf19.2830	RRP9	54.18	48.19	42.13	43	-0.189565774	0.998825	Ribosomal protein; mutation confers resistance to 5-fluorocytosine (5-FC), 5-fluorouracil (5-FU), and tubercidin (7-deazaadenosine); physically interacts with TAP-tagged Nop1; Hap43-induced; Spider biofilm induced
orf19.6064	orf19.6064	46.86	48.18	44.64	42.08	-0.097046393	0.998825	Ortholog(s) have phosphatidylinositol binding, ubiquitin binding activity

orf19.5719	orf19.5719	45.2	48.17	47.56	33.67	-0.134854122	0.998825	Predicted ORF from Assembly 19; removed from Assembly 20; subsequently reinstated in Assembly 21 and merged with orf19.1020 based on comparative genome analysis
orf19.2972	PDE2	58.22	48.14	36.48	31.28	-0.45326825	0.568429	High affinity cyclic nucleotide phosphodiesterase; moderates signaling by cAMP; required for virulence, switching, cell wall, hyphal, not pseudohyphal growth; expressed shortly after hyphal induction; rat catheter and Spider biofilm induced
orf19.6464	orf19.6464	51.4	48.08	46.18	46.05	-0.082092095	0.998825	Protein of unknown function; induced upon adherence to polystyrene; oxidative stress-induced via Cap1
orf19.6404	GSH2	43.85	48.05	51.26	44.73	0.031499794	0.998825	Putative glutathione synthase; induced in high iron; heavy metal (cadmium) stress-induced
orf19.3758	orf19.3758	55.77	48.05	43.38	46.61	-0.152018191	0.998825	Ortholog(s) have fungal-type vacuole membrane localization
orf19.6632	ACO2	57.26	48	79.65	76.13	0.419918074	0.565326	Putative aconitate hydratase 2; induced in high iron; regulated by Gcn4; repressed by amino acid starvation (3-AT); protein levels decrease in stationary phase yeast; flow model and Spider biofilm repressed
orf19.715	orf19.715	43.11	47.98	140.35	106.22	1.089626591	0.000375	Protein of unknown function; Hap43-induced; rat catheter and Spider biofilm induced
orf19.7367	UBP1	50.28	47.97	49.54	46.47	-0.030202324	0.998825	Ortholog(s) have ubiquitin-specific protease activity, role in protein deubiquitination and endoplasmic reticulum localization
orf19.4811	orf19.4811	65.76	47.97	56.82	57.36	-0.005612856	0.998825	Putative tricarboxylate carrier family protein; localized to the mitochondrial membrane

orf19.1282	CKS1	45.28	47.93	61.77	55.1	0.225141541	0.957291	Ortholog(s) have histone binding, protein complex binding, protein homodimerization activity, protein kinase activator activity, ubiquitin binding activity
orf19.5406	orf19.5406	42.98	47.9	50.32	47.06	0.053155009	0.998825	Predicted plasma membrane associated protein phosphatase; required for normal filamentous growth; mRNA binds She3 and is localized to hyphal tips
orf19.5182	POL3	54.35	47.84	52.91	50	0.001627591	0.998825	Large subunit of DNA polymerase III; partially complements defects of an S. cerevisiae cdc2 mutant; differing reports about periodic (G1/S) or non-periodic mRNA expression through cell cycle; Hap43p-repressed
orf19.2964	orf19.2964	51.25	47.83	58.28	51.78	0.098992074	0.998825	Ortholog(s) have DNA translocase activity
orf19.1048	IFD6	60.68	47.82	36.03	35.41	-0.427140074	0.612468	Aldo-keto reductase; similar to aryl alcohol dehydrogenases; protein increase correlates with MDR1 overexpression (not CDR1 or CDR2) in fluconazole-resistant clinical isolates; farnesol regulated; possibly essential; Spider biofilm induced
orf19.2990	XOG1	31.08	47.81	22.93	29.14	-0.370345351	0.795912	Exo-1,3-beta-glucanase; 5 glycosyl hydrolase family member; affects sensitivity to chitin and glucan synthesis inhibitors; not required for yeast-to-hypha transition or for virulence in mice; Hap43-induced; Spider biofilm induced
orf19.761	TCO89	45.3	47.8	43.53	37.35	-0.146019575	0.998825	Putative homolog of S. cerevisiae Tco89p, which is a component of the TOR C1 complex that is involved in the regulation of growth and cell wall integrity; heterozygous null mutant displays sensitivity to rapamycin
orf19.1111	orf19.1111	36.2	47.7	43.76	35.81	-0.05959356	0.998825	Ortholog(s) have chromatin binding, protein complex scaffold activity and role in RNA polymerase II transcriptional preinitiation complex assembly, histone acetylation
orf19.1753	PUS7	54.04	47.7	43.31	39.57	-0.21370932	0.98704	Pseudouridine synthase; catalyzes pseudouridylation in U2 snRNA, 5S rRNA, cytoplasmic tRNAs and in pre-tRNA(Tyr); F-12/CO2 early biofilm induced

orf19.6362	SPA2	50.69	47.67	49.31	46.38	-0.050643089	0.998825	Protein involved in cell polarity, Spitzenkorper formation; required for mouse virulence; localizes to hyphal tip; cell-cycle regulated localization in yeast-form cells; functional domains conserved with S. cerevisiae ; Hap43p-induced gene
orf19.6220	orf19.6220	49.53	47.67	41.7	45.56	-0.114859881	0.998825	Ortholog(s) have role in positive regulation of transcription elongation from RNA polymerase II promoter and CCR4-NOT core complex localization
orf19.1569	UTP22	51.02	47.67	42.27	40.32	-0.195375794	0.998825	Putative U3 snoRNP protein; Ssr1-induced; repressed by prostaglandins; heterozygous null mutant is resistant to parnafungin
orf19.4844	orf19.4844	57.82	47.64	39.53	41.31	-0.274614537	0.901815	Ortholog(s) have RNA binding, polynucleotide adenylyltransferase activity, protein binding, bridging activity
orf19.1493	RAD7	56.82	47.63	52.8	47.4	-0.058327352	0.998825	Protein similar to S. cerevisiae Rad7p, which is a subunit of the Nucleotide Excision Repair Factor 4; induced under hydroxyurea treatment
orf19.3104	YDC1	56.35	47.61	50.9	39.85	-0.144054825	0.998825	Alkaline dihydroceramidase; involved in sphingolipid metabolism; Mob2-dependent hyphal regulation; transcript is regulated by Nrg1 and Mig1; Hap43-repressed
orf19.5848	orf19.5848	40.04	47.57	46.77	53.28	0.117320599	0.998825	RING-type zinc finger protein; upregulated during oral infection; mutants have reduced ability to damage oral epithelial cells; Spider and flow model biofilm induced
orf19.850	orf19.850	57.1	47.56	45.95	34.95	-0.266713323	0.918389	Ortholog(s) have protein-N-terminal asparagine amidohydrolase activity, protein-N-terminal glutamine amidohydrolase activity and role in cellular protein modification process, protein catabolic process
orf19.5480	ILV1	53.11	47.55	64.12	59.33	0.197465788	0.998825	Putative threonine dehydratase; regulated by Gcn4 and Gcn2; induced by amino acid starvation (3-AT); protein present in yeast exponential and stationary phase cultures; early-stage flow model biofilm induced

orf19.3091	orf19.3091	48.12	47.51	46.3	46.06	-0.051539963	0.998825	Protein of unknown function; repressed during core stress response
orf19.3969	SFL2	42.58	47.48	76.17	69.35	0.490974648	0.41868	Transcription factor involved in regulation of morphogenesis; required for filamentous growth, for virulence in RHE model but not in mice; induced upon RHE infection but C. dubliniensis ortholog is not; Spider biofilm induced
orf19.1848.1	orf19.1848.1	41.41	47.45	61.45	71.48	0.408913117	0.621224	Ortholog of S. cerevisiae : KSH1, C. dubliniensis CD36 : Cd36_32390, C. parapsilosis CDC317 : CPAR2_203260, Candida tenuis NRRL Y-1498 : CANTEDRAFT_116778 and Debaryomyces hansenii CBS767 : DEHA2A10604g
orf19.541	orf19.541	46.87	47.36	50.92	43.39	-0.010561611	0.998825	Has domain(s) with predicted oxidoreductase activity, acting on paired donors, with incorporation or reduction of molecular oxygen, 2-oxoglutarate as one donor, and incorporation of one atom each of oxygen into both donors activity
orf19.6905	orf19.6905	40.83	47.32	39.04	40.32	-0.11379942	0.998825	Ortholog of S. cerevisiae : YNL011C, C. glabrata CBS138 : CAGL0H03619g, C. dubliniensis CD36 : Cd36_71090, C. parapsilosis CDC317 : CPAR2_300420 and Candida tenuis NRRL Y-1498 : CANTEDRAFT_111980
orf19.1804	orf19.1804	55.42	47.29	39.43	46.47	-0.186754294	0.998825	Ortholog(s) have role in cellular response to cadmium ion, detoxification of cadmium ion and mitochondrion localization
orf19.6602	orf19.6602	48.18	47.29	46.96	48.67	0.002249359	0.998825	Ortholog(s) have role in aerobic respiration, ubiquinone biosynthetic process and mitochondrial inner membrane localization
orf19.1735	orf19.1735	56.66	47.28	48.15	49.6	-0.067026537	0.998825	Ortholog(s) have U2-type spliceosomal complex localization
orf19.4785	PTC1	56.94	47.27	54.96	53.6	0.041339343	0.998825	Putative protein phosphatase of the Type 2C-related family (serine/threonine-specific), similar to S. cerevisiae Ptc1p; mutant shows virulence defect

orf19.169	CHO2	49.68	47.25	56.92	56.69	0.158309238	0.998825	Putative phosphatidyl-ethanolamine N-methyltransferase; fungal-specific (no human or murine homolog); amphotericin B repressed; Hap43p-induced gene
orf19.2711	orf19.2711	56.36	47.25	44.21	36.74	-0.2468822	0.938642	Similar to S. cerevisiae Elp2, an Elongator complex subunit required for modification of wobble nucleosides in tRNA; repressed in core stress response
orf19.394	orf19.394	53.73	47.25	64.06	56.78	0.179812213	0.998825	Putative kynureninase; predicted role in NAD biosynthesis; Hap43-repressed gene; flow model biofilm induced
orf19.3435	orf19.3435	65.06	47.22	77.77	43.97	0.075562742	0.998825	Ortholog of Candida albicans WO-1 : CAWG_05215
orf19.5802	orf19.5802	61.69	47.2	50.52	45.98	-0.126015444	0.998825	Ortholog(s) have role in maturation of SSU-rRNA and cytoplasm, nucleus localization
orf19.2049	orf19.2049	40.18	47.04	14.39	10.4	-1.023911762	0.011156	Plasma membrane-associated protein; heterozygous null mutant displays sensitivity to virgineone; Spider biofilm induced
orf19.705	GCN5	52.44	47.03	48.16	38.48	-0.149873882	0.998825	Putative histone acetyltransferase; mutation confers hypersensitivity to 5-fluorouracil (5-FU); possibly an essential gene, disruptants not obtained by UAU1 method
orf19.3517	orf19.3517	53.82	47.02	61.57	57.25	0.162465154	0.998825	Ortholog(s) have Dsc E3 ubiquitin ligase complex, endoplasmic reticulum, fungal-type vacuole lumen localization
orf19.5890	orf19.5890	52.45	46.98	57.64	52.17	0.099411619	0.998825	Ortholog of C. dubliniensis CD36 : Cd36_84350, C. parapsilosis CDC317 : CPAR2_806070, Candida tenuis NRRL Y-1498 : CANTEDRAFT_133308 and Debaryomyces hansenii CBS767 : DEHA2F11682g

orf19.5418	orf19.5418	43.96	46.93	56.94	48.09	0.133128656	0.998825	Ortholog(s) have role in ER to Golgi vesicle-mediated transport and endoplasmic reticulum localization
orf19.1757	orf19.1757	52.13	46.92	45.54	40.55	-0.137916861	0.998825	Putative C2H2 transcription factor; expression upregulated in clinical isolates from HIV+ patients with oral candidiasis; Spider biofilm induced
orf19.5136	orf19.5136	47.75	46.9	34.54	38.42	-0.262429688	0.930538	Putative pyridoxamine 5'-phosphate oxidase; planktonic growth and early-stage flow model biofilm induced
orf19.6931	orf19.6931	53.37	46.89	45.9	47.27	-0.080625098	0.998825	Putative cleavage factor I subunit; heterozygous null mutant exhibits hypersensitivity to parnafungin and cordycepin in the C. albicans fitness test
orf19.7034	orf19.7034	43.15	46.73	54.13	47.48	0.10445411	0.998825	Putative eIF4E-associated protein;, accelerates mRNA degradation by promoting decapping; Spider biofilm repressed
orf19.6171	NUP159	40.88	46.72	60.29	60.48	0.306799618	0.858075	FG-nucleoporin component of central core of the nuclear pore complex (NPC); contributes to nucleocytoplasmic transport; rat catheter biofilm repressed
orf19.1007	KCS1	55.98	46.72	35.8	27.64	-0.464663842	0.55304	Putative bZIP transcription factor; Spider biofilm induced
orf19.2631	orf19.2631	60.41	46.67	47.89	37.04	-0.238354934	0.946319	Subunit of Elongator complex; required for modification of wobble nucleosides in tRNA in S. cerevisiae; Hap43-induced gene
orf19.2909	ERG26	49.16	46.64	63.83	66.65	0.317711453	0.833355	C-3 sterol dehydrogenase, catalyzes the 2nd of 3 steps required to remove 2 C-4 methyl groups from an intermediate in ergosterol biosynthesis; amphotericin B, caspofungin repressed; Spider biofilm repressed

orf19.2730	orf19.2730	44.97	46.63	43.52	42.25	-0.076185942	0.998825	Has domain(s) with predicted zinc ion binding activity
orf19.1433	orf19.1433	40.82	46.61	63.26	55.24	0.287348306	0.878538	Protein of unknown function; Hap43-repressed; colony morphology-related gene regulation by Ssn6; Spider biofilm induced
orf19.2930	orf19.2930	42.53	46.6	46.02	37.27	-0.090512098	0.998825	Predicted translation initiation factor; role in translational initiation; Spider biofilm repressed
orf19.6773	ECM29	46.17	46.58	47.23	48.12	0.012396895	0.998825	Putative scaffold protein; assists in association of the proteasome core particle with the regulatory particle; ortholog of S. cerevisiae Ecm29; transposon mutation affects filamentous growth; flow model biofilm repressed
orf19.1253	PHO4	44.14	46.56	40.76	33.84	-0.197190984	0.998825	bHLH transcription factor of the myc-family; required for growth in medium lacking phosphate and for resistance to copper and Phloxine B; induced by Mnl1 under weak acid stress
orf19.3728	orf19.3728	48.92	46.56	63.26	59.65	0.248937826	0.930538	Ortholog(s) have protein phosphatase 1 binding, protein phosphatase type 1 regulator activity, role in chromosome segregation, regulation of phosphoprotein phosphatase activity and cytoplasm localization
orf19.5620	orf19.5620	49.62	46.55	96.13	115.14	0.849081713	0.013442	Stationary phase enriched protein; Gcn4-regulated; induced by amino acid starvation (3-AT), benomyl or in azole-resistant strain that overexpresses MDR1; flow model biofilm induced; rat catheter biofilm repressed; overlaps orf19.5621
orf19.7670	orf19.7670	49.04	46.5	53.88	46.57	0.041567493	0.998825	Putative Ca2+/H+ antiporter; oral infection upregulated gene; mutants have reduced capacity to damage oral epithelial cells
orf19.4928	SEC2	52.41	46.5	54.8	56.65	0.11968524	0.998825	Guanyl-nucleotide exchange factor for the small G-protein Sec4; delivery of post-Golgi secretory vesicles to sites of polarized growth; phosphorylation by Cdc28 needed for normal hyphal growth; Hap43-repressed; flow model biofilm induced

orf19.3122	ARR3	47.28	46.47	34.02	38.43	-0.253351501	0.938642	Ortholog of S. cerevisiae Arr3; arsenite transporter of the plasma membrane required for resistance to arsenic compounds; benomyl-induced; Gcn4-regulated; Hap43-repressed; mutant is viable; Spider biofilm induced
orf19.5754	orf19.5754	49.38	46.46	45.25	45.93	-0.050953401	0.998825	Putative membrane protein with a predicted role in zinc ion homeostasis; Hap43-induced; fluconazole-induced; rat catheter and Spider biofilm induced
orf19.3681	orf19.3681	44.96	46.42	48.27	51.39	0.076002196	0.998825	Ortholog(s) have Ran guanyl-nucleotide exchange factor activity, protein transporter activity
orf19.4287	orf19.4287	41.26	46.38	52.24	63.82	0.282361009	0.888717	Putative oxidoreductase; Hap43-repressed gene; clade-associated gene expression
orf19.7234	orf19.7234	44.73	46.37	46.92	45.69	0.012363369	0.998825	Putative RSC chromatin remodeling complex component; possibly an essential gene, disruptants not obtained by UAU1 method
orf19.242.2	orf19.242.2	57.04	46.33	72.29	66.33	0.302759896	0.853836	Ortholog(s) have endoplasmic reticulum localization
orf19.1031	HMG1	43.71	46.32	59.19	60.81	0.286602575	0.878136	HMG-CoA reductase; enzyme of sterol pathway; inhibited by lovastatin; gene not transcriptionally regulated in response to lovastatin and fluconazole
orf19.4722	RTG1	46.08	46.3	55.15	50.99	0.134128541	0.998825	Ortholog(s) have sequence-specific DNA binding RNA polymerase II transcription factor activity
orf19.7403	orf19.7403	42.83	46.25	49.49	39.67	-0.011412853	0.998825	Ortholog of S. cerevisiae : YML020W, C. glabrata CBS138 : CAGL0J10538g, C. dubliniensis CD36 : Cd36_86190, C. parapsilosis CDC317 : CPAR2_404740 and Candida tenuis NRRL Y-1498 : CANTEDRAFT_107670

orf19.3756	CHR1	64.36	46.23	38.92	34.96	-0.395746126	0.692027	Predicted DEAD-box ATP-dependent RNA helicase; functional homolog of S. cerevisiae Rok1; Hap43-induced; Spider biofilm induced
orf19.4993	SEC18	47.86	46.23	56.05	47.25	0.08235839	0.998825	Functional homolog of S. cerevisiae Sec18p, which acts in protein transport; conserved ATP binding site; not glycosylated
orf19.2722	CGR1	57.1	46.22	59.34	44.07	-0.009605861	0.998825	Negative regulator of yeast-form growth; HSP70 family member; induced by growth cessation at yeast-hyphal transition or in planktonic growth; physically interacts with Msi3p; similar to rat anti-aging gene, SMP30, stationary phase enriched
orf19.6860	orf19.6860	43.72	46.2	41.95	55.72	0.077727153	0.998825	Ortholog(s) have CDP-diacylglycerol-inositol 3-phosphatidyltransferase activity, role in phosphatidylinositol biosynthetic process and Golgi apparatus, integral to membrane, mitochondrial outer membrane localization
orf19.4184	orf19.4184	49.73	46.13	61.08	45.08	0.089291306	0.998825	Protein with a predicted role in clathrin cage assembly; Hap43-repressed; Spider biofilm repressed
orf19.5278	orf19.5278	53.09	46.12	60.8	54.93	0.155593479	0.998825	Protein of unknown function; Spider biofilm induced
orf19.1345	LIP8	52.76	46.1	76.24	74.46	0.436600324	0.541043	Secreted lipase, member of a differentially expressed lipase gene family with possible roles in nutrition and/or in creating an acidic microenvironment; LIP5 and LIP8 are expressed at all stages of both mucosal and systemic infection
orf19.6837	FMA1	34.31	46.06	51.7	66.65	0.383355026	0.720423	Putative oxidoreductase; induced by ciclopirox olamine; upregulation correlates with clinical development of fluconazole resistance; Spider biofilm repressed
orf19.2527	orf19.2527	56.68	46.05	44.5	36.8	-0.234415617	0.951606	Putative protein of unknown function; Hap43-induced; required for normal biofilm growth; F-12/CO2 early biofilm induced

orf19.5107	NOT5	47.75	45.97	50.04	43.06	-0.020595662	0.998825	Protein with similarity to S. cerevisiae Not5p, a member of the transcription regulatory CCR4-NOT complex; required for hyphal growth; antigenic during human oral infection; greater expression in yeast-form cells than hyphae
orf19.4529	orf19.4529	48.25	45.92	54.66	54.25	0.142642363	0.998825	Ortholog of Srp21, signal recognition particle subunit, functions in protein targeting to the endoplasmic reticulum membrane; predicted adhesin-like protein; mutants are viable
orf19.405	VCX1	45.04	45.92	99.09	81.74	0.732043798	0.059858	Putative H+/Ca2+ antiporter; Spider biofilm repressed
orf19.5565	HPD1	57.07	45.92	41.06	30.64	-0.356583886	0.789669	3-hydroxypropionate dehydrogenase; involved in degradation of toxic propionyl-CoA; rat catheter and Spider biofilm induced
orf19.7179	orf19.7179	48.91	45.89	53.69	53.76	0.12279928	0.998825	Ortholog(s) have role in intracellular sequestering of iron ion, iron-sulfur cluster assembly, protein maturation and mitochondrial matrix localization
orf19.1830	orf19.1830	82.64	45.89	72.65	61.02	0.035517158	0.998825	Protein of unknown function; Hap43-induced; rat catheter and Spider biofilm induced
orf19.4930	SPC3	51.23	45.85	80.14	64.41	0.40778269	0.606786	Essential protein; similar to S. cerevisiae Spc3p, a component of the signal peptidase complex required for signal peptidase activity; predicted integral ER membrane protein; complements spc3 and suppresses sec61 S. cerevisiae mutants
orf19.7576	orf19.7576	56.53	45.82	49.77	56.86	0.033700658	0.998825	Ortholog of S. pombe atl1 alkyltransferase-like protein; mutants are viable; Spider biofilm induced
orf19.2884	CDC68	55.92	45.8	61.99	54.54	0.134389417	0.998825	Functional homolog of S. cerevisiae Cdc68, a transcription elongation factor; essential; possible drug target

orf19.2437	ARC35	58.79	45.75	71.99	51.93	0.162970128	0.998825	Putative ARP2/3 complex subunit; shows colony morphology-related gene regulation by Ssn6p; mutation confers hypersensitivity to cytochalasin D
orf19.4686	orf19.4686	43.78	45.65	51.24	44.66	0.053828886	0.998825	Ortholog of S. cerevisiae : ESL1, C. dubliniensis CD36 : Cd36_41030, C. parapsilosis CDC317 : CPAR2_401340, Candida tenuis NRRL Y-1498 : CANTEDRAFT_104645 and Debaryomyces hansenii CBS767 : DEHA2E18766g
orf19.1901	MCM3	51.44	45.56	80.86	66.26	0.431242534	0.554747	Putative DNA replication protein; periodic mRNA expression, peak at cell-cycle M/G1 phase; Spider biofilm induced
orf19.4888	orf19.4888	52.86	45.55	56.12	48.83	0.051321994	0.998825	Has domain(s) with predicted nucleic acid binding, nucleotide binding activity
orf19.5171	PMT1	42.41	45.54	79.77	75.06	0.588808581	0.229994	Protein mannosyltransferase; required for virulence in mice and for adhesion to epithelial cells; role in hyphal growth and drug sensitivity; Als1, Sec20, Kre9, Pir1 are substrates; 1 of 5 PMT family members
orf19.211	orf19.211	32.27	45.52	37.55	34.32	-0.08641635	0.998825	Predicted homeodomain-like transcription factor; also has a metal-binding Ada DNA repair protein domain; Spider biofilm induced
orf19.1086	orf19.1086	46.91	45.51	44.61	46.37	-0.030052154	0.998825	Ortholog(s) have role in protein deubiquitination, regulation of ER to Golgi vesicle-mediated transport, regulation of retrograde vesicle-mediated transport, Golgi to ER, ribophagy and cytoplasm localization
orf19.5296	orf19.5296	53.93	45.51	60.44	57.52	0.164370977	0.998825	Ortholog(s) have role in mitochondrial respiratory chain complex IV assembly and integral to mitochondrial inner membrane localization
orf19.2602	OPT1	46.02	45.46	33.69	40.58	-0.192363013	0.998825	Oligopeptide transporter; transports 3-to-5-residue peptides; alleles are distinct, one has intron; suppresses S. cerevisiae ptr2-2 mutant defects; induced by BSA or peptides; Stp3p, Hog1p regulated; flow model biofilm induced

orf19.7235	orf19.7235	45.95	45.45	47.47	40.25	-0.054395558	0.998825	Putative protein of unknown function; mutation confers hypersensitivity to amphotericin B
orf19.2823	RFG1	51.24	45.36	0.1	0	-2.004586891	NA	HMG domain transcriptional repressor of filamentous growth and hyphal genes; in Tup1-dependent and -independent pathways; binds DNA; transcript not regulated by oxygen or serum; not responsible for hypoxic repression; Spider biofilm induced
orf19.7366	orf19.7366	48.02	45.35	44.18	43.63	-0.06292881	0.998825	Ortholog(s) have S-adenosylmethionine-dependent methyltransferase activity and nucleolus localization
orf19.6094	VPS53	50.82	45.26	62.46	54.26	0.186175162	0.998825	Ortholog(s) have role in Golgi to vacuole transport, retrograde transport, endosome to Golgi and GARP complex localization
orf19.6527	orf19.6527	37.78	45.24	34.58	36.17	-0.156435646	0.998825	Pheromone-regulated protein (Prm10) of S. cerevisiae; colony morphology-related gene regulation by Ssn6; induced by Mnl1 under weak acid stress; possibly essential gene, disruptants not obtained by UAU1 method; Spider biofilm induced
orf19.1247	orf19.1247	46.94	45.22	51.5	45.09	0.032977055	0.998825	Ortholog of C. dubliniensis CD36 : Cd36_45230, C. parapsilosis CDC317 : CPAR2_500450, Candida tenuis NRRL Y-1498 : CANTEDRAFT_103249 and Debaryomyces hansenii CBS767 : DEHA2C07524g
orf19.4496	orf19.4496	52.56	45.21	41.01	37.6	-0.219592552	0.976613	Ortholog(s) have role in cellular calcium ion homeostasis and Golgi medial cisterna, cis-Golgi network, endoplasmic reticulum, fungal-type vacuole membrane localization
orf19.6443	orf19.6443	52.08	45.2	60.91	49.79	0.128731761	0.998825	Protein with an enoyl-CoA hydratase related domain; Spider biofilm induced
orf19.4055	orf19.4055	41.38	45.19	73.91	89.15	0.66620765	0.128665	Protein similar to S. cerevisiae Ybr075wp; transposon mutation affects filamentous growth; clade-associated gene expression

orf19.1032	SKO1	45.1	45.19	44.45	45.39	-0.019600759	0.998825	bZIP transcription factor; involved in cell wall damage response; represses the yeast-to-hypha transition; mutants are caspofungin sensitive; induced by osmotic stress via Hog1; induced by Mnl1 under weak acid stress
orf19.7409.1	orf19.7409.1	58.55	45.18	63.51	74.74	0.301570454	0.858075	Ortholog(s) have structural constituent of ribosome activity and mitochondrial large ribosomal subunit localization
orf19.5389	FKH2	36.76	45.17	41.22	40.3	-0.022469802	0.998825	Forkhead transcription factor; morphogenesis regulator; required for wild-type hyphal transcription, cell separation, and for virulence in cell culture; mutant lacks true hyphae, is constitutively pseudohyphal; upregulated in RHE model
orf19.2012	NOT3	50.95	45.13	47.23	40.27	-0.106780715	0.998825	Transcriptional regulator; not required for buccal epithelial cell adherence or virulence in mouse systemic infection; null mutant colonies exhibit slightly decreased filamentation ratio; required for yeast adherence to silicone substrate
orf19.4013	orf19.4013	48.14	45.11	59.3	63.69	0.281903975	0.880321	Putative protein of unknown function; Hap43p-repressed gene; S. cerevisiae ortholog YHR045W localizes to the endoplasmic reticulum
orf19.2418	orf19.2418	45.11	45.1	46	41.62	-0.031826056	0.998825	Ortholog of C. dubliniensis CD36 : Cd36_28190, C. parapsilosis CDC317 : CPAR2_802530, Candida tenuis NRRL Y-1498 : CANTEDRAFT_109615 and Debaryomyces hansenii CBS767 : DEHA2A12650g
orf19.2249	orf19.2249	35.24	45.1	36.48	50.03	0.06029169	0.998825	Putative metalloprotease of the mitochondrial inner membrane; expression downregulated in an ssr1 null mutant
orf19.3041	orf19.3041	46.54	45.1	37.89	39.86	-0.168262454	0.998825	Ortholog(s) have ubiquitin protein ligase binding activity
orf19.6926	CSC25	47.67	45.09	45.12	42.5	-0.063386729	0.998825	Guanyl-nucleotide exchange factor; activator of Ras/adenylyl cyclase pathway; functional homolog of S. cerevisiae Cdc25p; commonly called Cdc25; transposon mutation affects filamentous growth

orf19.1769	orf19.1769	50.82	45.08	42.12	43.52	-0.11611083	0.998825	Ortholog(s) have G-protein beta/gamma-subunit complex binding, actin binding activity, role in positive regulation of transcription from RNA polymerase II promoter by pheromones, protein folding and cytoplasm localization
orf19.1942	SGE1	51.35	45.04	42.39	37.85	-0.187479087	0.998825	Putative multidrug resistance factor; induced in low iron; regulated by Sef1, Sfu1, and Hap43
orf19.4641	NMT1	51.51	45.02	54.67	52.45	0.092370176	0.998825	Myristoyl-CoA:protein N-myristoyltransferase; attaches the fatty acid myristate to a small number of proteins at an N-terminal Gly; essential; antifungal drug target; functional homolog of S. cerevisiae Nmt1p
orf19.4751	orf19.4751	53.77	45.01	51.07	52.54	0.043625655	0.998825	Ortholog(s) have structural constituent of ribosome activity and mitochondrial small ribosomal subunit localization
orf19.188	orf19.188	50.81	45	43.32	38.2	-0.175747838	0.998825	Ortholog of Candida albicans WO-1 : CAWG_04234
orf19.4553	orf19.4553	45.34	44.99	41.74	34.92	-0.161055935	0.998825	Ortholog of C. dubliniensis CD36 : Cd36_64990, C. parapsilosis CDC317 : CPAR2_600170, Candida tenuis NRRL Y-1498 : CANTEDRAFT_121753 and Debaryomyces hansenii CBS767 : DEHA2E19712g
orf19.7383	MNN9	41.76	44.97	54.85	50.43	0.178486507	0.998825	Protein of N-linked outer-chain mannan biosynthesis; mutant has defective cell wall; required for wild-type hyphal growth; mutant is hygromycin B sensitive and vanadate resistant; has N-terminal membrane-spanning segment (positions 18-34)
orf19.4818	orf19.4818	48.97	44.96	63.02	60.76	0.27950226	0.881712	Protein of unknown function; Spider biofilm induced
orf19.3477	orf19.3477	51.22	44.96	46.66	41.91	-0.084088593	0.998825	Putative pseudouridine synthase; predicted role in snRNA pseudouridine synthesis, tRNA pseudouridine synthesis; Spider biofilm induced

orf19.4278	orf19.4278	48.63	44.93	42.89	44.25	-0.084385428	0.998825	Ortholog(s) have methylated histone residue binding activity and role in negative regulation of transcription from RNA polymerase II promoter
orf19.2182	BLM3	44.54	44.92	42.82	43.46	-0.053457488	0.998825	Putative proteasome activator; binds core proteasome and stimulates proteasome-mediated protein degradation by inducing gate opening; ortholog of S. cerevisiae Blm10; transcript regulated by Nrg1 and Mig1
orf19.6891	RFC1	48.99	44.9	64.92	54.55	0.242522137	0.938642	Putative DNA replication factor C subunit; ortholog of S. cerviaie Rfc1; likely essential, based on an insertional mutagenesis strategy; rat catheter biofilm repressed
orf19.7184	orf19.7184	55.09	44.9	51.56	41.4	-0.081844294	0.998825	Ortholog of C. dubliniensis CD36 : Cd36_73640, C. parapsilosis CDC317 : CPAR2_702340, Candida tenuis NRRL Y-1498 : CANTEDRAFT_112965 and Debaryomyces hansenii CBS767 : DEHA2G16566g
orf19.7483	CRM1	51.35	44.87	53.65	46.38	0.032087934	0.998825	Functional homolog of S. cerevisiae Crm1, which acts in protein nuclear export; predicted to be resistant to antifungal drug leptomycin B; partially suppresses signal transduction defects of S. cerevisiae ste20 mutant
orf19.6209	orf19.6209	49.29	44.85	30.87	20.74	-0.549645109	0.39542	Predicted membrane transporter; monocarboxylate porter (MCP) family, major facilitator superfamily (MFS); possibly essential, disruptants not obtained by UAU1 method; rat catheter and Spider biofilm induced
orf19.1675	orf19.1675	54.68	44.85	61.06	60.56	0.200389347	0.998825	Ortholog(s) have role in positive regulation of catalytic activity
orf19.4372	orf19.4372	38.56	44.81	39.86	40.7	-0.044741445	0.998825	Predicted transmembrane transporter; rat catheter and Spider biofilm induced
orf19.874	orf19.874	42.1	44.8	44.86	33.33	-0.116583665	0.998825	Predicted mitochondrial i-AAA protease supercomplex; degrades misfolded mitochondrial proteins; Hap43-repressed gene

orf19.757.1	orf19.757.1	31.62	44.78	44.67	43.34	0.11442522	0.998825	Protein of unknown function
orf19.3018	SPP1	57.07	44.78	44.22	45.57	-0.131422438	0.998825	Putative histone methyltransferase; induced by nitric oxide independent of Yhb1p; cell-cycle regulated periodic mRNA expression
orf19.6585	orf19.6585	54.05	44.75	39.75	40.82	-0.204636908	0.998825	Ortholog(s) have mitochondrion localization
orf19.974	ROT2	41.5	44.73	66.9	64.55	0.433095868	0.568429	Alpha-glucosidase II, catalytic subunit, required for N-linked protein glycosylation and normal cell wall synthesis; alkaline downregulated
orf19.4297	CKB2	53.26	44.72	51.7	46.49	-0.008417758	0.998825	Regulatory subunit of protein kinase CK2 (casein kinase II), beta' subunit; null mutants are hypersensitive to caspofungin
orf19.7420	orf19.7420	62.77	44.71	50.61	41.65	-0.157850346	0.998825	Ortholog(s) have RNA polymerase II transcription coactivator activity involved in preinitiation complex assembly activity
orf19.4079	SLP2	51.7	44.71	45.81	47.22	-0.049412212	0.998825	Protein similar to stomatin mechanoreception protein
orf19.2973	orf19.2973	49.17	44.71	42.98	42.68	-0.0959439	0.998825	Ortholog(s) have RNA binding, flap-structured DNA binding activity and role in nuclear-transcribed mRNA poly(A) tail shortening, positive regulation of endodeoxyribonuclease activity
orf19.459	ADP1	50.27	44.71	60.97	61.23	0.25181825	0.927778	Putative PDR-subfamily ABC transporter; similar to WHITE subfamily proteins; gene used for strain identification by multilocus sequence typing

orf19.4809	ERG12	49.06	44.71	48.06	52.35	0.055206281	0.998825	Ortholog(s) have mevalonate kinase activity and role in ergosterol biosynthetic process, farnesyl diphosphate biosynthetic process, mevalonate pathway, isopentenyl diphosphate biosynthetic process, mevalonate pathway
orf19.5564	orf19.5564	43.6	44.68	61.26	57.68	0.288523058	0.878136	Ortholog(s) have nucleolus localization
orf19.2105	orf19.2105	45.52	44.62	47.17	33	-0.136095296	0.998825	Putative pre-mRNA-splicing factor; possibly an essential gene, disruptants not obtained by UAU1 method
orf19.4201	NHX1	47.96	44.51	62.29	56.8	0.245408393	0.936054	Protein similar to S. cerevisiae Nhx1p, which is an Na+/H+ exchanger required for intracellular sequestration of Na+
orf19.1969	CCW14	48.25	44.5	50.68	44.14	0.011656827	0.998825	Putative mannoprotein of cell wall with role in response to stress; increased mRNA abundance observed in cyr1 homozygous mutant (hyphal or yeast-form cells) and in ras1 homozygous mutant (yeast-form cells)
orf19.1643	orf19.1643	53.51	44.48	55.08	46.84	0.032266945	0.998825	Ortholog(s) have serine-type endopeptidase activity, role in regulation of mitochondrion organization, signal peptide processing and mitochondrial inner membrane localization
orf19.755	MRPL37	40.59	44.46	33.27	37.13	-0.19098036	0.998825	Putative mitochondrial large subunit ribosomal protein; shows colony morphology-related gene regulation by Ssn6p
orf19.6869	orf19.6869	52.71	44.45	45.6	33.6	-0.197612828	0.998825	Putative lipid raft associated protein; Spider biofilm induced
orf19.3154	orf19.3154	54.45	44.44	51.37	46.62	-0.007556472	0.998825	Ortholog(s) have mitochondrial membrane localization

orf19.2146	HAT2	41.82	44.41	43.5	39.73	-0.044303044	0.998825	Putative Hat1-Hat2 histone acetyltransferase complex subunit; role in DNA damage repair and morphogenesis; mutations cause constitutive pseudohyphal growth, caspofungin sensitivity; rat catheter and Spider biofilm repressed
orf19.1548	orf19.1548	50.41	44.39	36.47	45.05	-0.150083652	0.998825	Ortholog(s) have role in mRNA splicing, via spliceosome and U4/U6 x U5 tri-snRNP complex localization
orf19.2346	orf19.2346	54.57	44.37	49.69	42.37	-0.081632899	0.998825	Putative protein of unknown function, transcription is positively regulated by Tbf1p
orf19.2263	orf19.2263	47.62	44.35	58.28	61.49	0.257550983	0.921805	Has domain(s) with predicted adenyl nucleotide binding activity
orf19.48	RPM2	39.73	44.27	29.08	27.1	-0.378416286	0.769055	Mitochondrial RNase P subunit; roles in nuclear transcription, cytoplasmic and mitochondrial RNA processing, mitochondrial translation; virulence-group-correlated expression; likely essential (UAU1 method); rat catheter biofilm induced
orf19.6874	orf19.6874	41.38	44.21	31.54	25.14	-0.375142117	0.777732	Putative helix-loop-helix (HLH) transcription factor with a role in filamentous growth
orf19.3226	orf19.3226	52.23	44.18	68.45	55.07	0.24737488	0.931333	Ortholog(s) have role in intracellular sterol transport and extracellular region, fungal-type vacuole lumen localization
orf19.2070	orf19.2070	44.5	44.16	48.61	36.44	-0.056120331	0.998825	Ortholog(s) have role in G1/S transition of mitotic cell cycle, chromatin remodeling, nucleosome disassembly, transcription elongation from RNA polymerase II promoter and RSC complex localization
orf19.6071	orf19.6071	45.49	44.16	39.77	37.53	-0.136649437	0.998825	Ortholog(s) have peptide alpha-N-acetyltransferase activity, role in N-terminal peptidyl-methionine acetylation, cytoskeleton organization, mitochondrion inheritance and NatB complex localization

orf19.5612	BMT4	44.48	44.15	20.12	16.07	-0.771935429	0.10182	Beta-mannosyltransferase; for elongation of beta-mannose chains on the acid-labile fraction of cell wall phosphopeptidomannan; 9-gene family member; regulated by Tsa1, Tsa1B; flow model biofilm induced; rat catheter biofilm repressed
orf19.1201	orf19.1201	42.86	44.09	46.11	34.13	-0.09228625	0.998825	Ortholog(s) have serine-tRNA ligase activity, role in invasive growth in response to glucose limitation, mitochondrial seryl-tRNA aminoacylation, pseudohyphal growth, spore germination and mitochondrion localization
orf19.7391	OCH1	57.85	44.06	64.64	57.18	0.178938789	0.998825	Alpha-1,6-mannosyltransferase; initiates N-glycan outer chain branch addition; similar to S. cerevisiae Och1p; required for wild-type virulence in mouse intravenous infection; fungal-specific (no human or murine homolog)
orf19.5312	MET4	37.11	44.06	49.13	48.62	0.176604785	0.998825	Putative transcription coactivator; predicted role in sulfur amino acid metabolism; required for yeast cell adherence to silicone substrate; Spider biofilm induced
orf19.2406	orf19.2406	50.39	44.01	60.94	50.06	0.160171675	0.998825	Ortholog(s) have role in microautophagy, positive regulation of TOR signaling cascade, positive regulation of transcription from RNA polymerase II promoter
orf19.4914	orf19.4914	48.6	44.01	58.73	44.45	0.092049382	0.998825	Ortholog(s) have role in cellular response to DNA damage stimulus
orf19.2264	OLE2	50.95	43.99	43.54	43.4	-0.094753113	0.998825	Protein with similarity to fatty acid desaturase (stearoyl-CoA desaturase); homozygous null mutant shows decreased production of prostaglandin E2
orf19.3077	VID21	43.27	43.98	47.99	40.45	0.000491204	0.998825	Subunit of the NuA4 histone acetyltransferase complex; soluble protein in hyphae; Spider biofilm repressed
orf19.1404	orf19.1404	57.01	43.97	43.21	40.8	-0.189351729	0.998825	Predicted tRNA dihydrouridine synthase; Spider biofilm induced

orf19.4897	SFH5	55.89	43.92	35.99	29.91	-0.405280481	0.682685	Putative phosphatidylinositol transporter; rat catheter and Spider biofilm repressed
orf19.3945	orf19.3945	50.57	43.9	39.38	32.09	-0.288951059	0.892193	Predicted COP9 signalosome complex subunit 12; flow model biofilm induced
orf19.4953	orf19.4953	47.72	43.9	51.17	42.52	0.011855074	0.998825	Putative ATPase; predicted role in ER-associated protein catabolism; induced during chlamydospore formation in both C. albicans and C. dubliniensis; rat catheter biofilm repressed
orf19.2387	orf19.2387	51.02	43.86	46.74	45.76	-0.028821586	0.998825	Putative tRNA-Pro synthetase; genes encoding ribosomal subunits, translation factors, and tRNA synthetases are downregulated upon phagocytosis by murine macrophage
orf19.921	HMS1	39.2	43.86	34.79	38.27	-0.130051494	0.998825	hLh domain Myc-type transcript factor; required for morphogenesis induced by elevated temperature or Hsp90 compromise; acts downstream of Pcl1; Spider biofilm induced
orf19.149	orf19.149	52.01	43.85	24.81	26.15	-0.586996745	0.314131	Protein of unknown function; transcript detected on high-resolution tiling arrays
orf19.1198	orf19.1198	39.93	43.83	44.8	40.86	0.012340028	0.998825	Predicted mitochondrial intermembrane space protein of unknown function; possibly an essential gene, disruptants not obtained by UAU1 method
orf19.4851	TFA1	55.06	43.81	49.87	42.59	-0.070722729	0.998825	Protein with polyglutamate motifs and abundant Ser/Thr residues; described as a subunit of TFIIE, which is a basal transcription initiation factor of RNA Polymerase II; possibly an essential gene, disruptants not obtained by UAU1 method
orf19.443	orf19.443	48.74	43.79	42.04	52.2	0.005066501	0.998825	Ortholog(s) have DNA-directed RNA polymerase activity, RNA polymerase III activity and role in tRNA transcription from RNA polymerase III promoter, transcription initiation from RNA polymerase III promoter

orf19.7567	orf19.7567	37.98	43.77	36	40.47	-0.080842478	0.998825	Protein of unknown function; induced by alpha pheromone in SpiderM medium
orf19.4335	TNA1	40.44	43.7	46.51	55.45	0.184180699	0.998825	Putative nicotinic acid transporter; detected at germ tube plasma membrane by mass spectrometry; transcript induced upon phagocytosis by macrophage; rat catheter biofilm induced
orf19.5486	orf19.5486	51.01	43.65	40.1	34.53	-0.237876196	0.951606	Putative endoribonuclease; heterozygous null mutant exhibits hypersensitivity to parnafungin and cordycepin in the C. albicans fitness test
orf19.4232	PTH1	42.34	43.65	42.89	42.08	-0.02078075	0.998825	Putative gluconate transport protein; antigenic during human oral infection; possibly an essential gene, disruptants not obtained by UAU1 method
orf19.4455	orf19.4455	50.74	43.64	37.08	38.4	-0.236425618	0.951606	Protein of unknown function; Spider biofilm induced
orf19.1095	GLE2	45.61	43.64	46.3	40.11	-0.054311002	0.998825	Putative nuclear pore complex; possibly an essential gene, disruptants not obtained by UAU1 method; rat catheter biofilm repressed
orf19.5531	CDC37	49.99	43.64	51.2	48.25	0.044121356	0.998825	Chaperone for Crk1p; interacts with Crk1p kinase domain and with Sti1p; putative phosphorylation site at Ser14; functional homolog of S. cerevisiae Cdc37p; likely to be essential for growth; regulated by Gcn2p and Gcn4p
orf19.4180	SEC72	56.21	43.63	61.69	52.58	0.136178798	0.998825	ER protein-translocation complex component; regulated by Gcn4p; repressed in response to amino acid starvation (3-aminotriazole treatment); sumoylation target
orf19.702	orf19.702	47.03	43.63	38.78	37.94	-0.17024172	0.998825	Ortholog(s) have DNA helicase activity, role in regulation of translational termination and polysome localization

orf19.3798	orf19.3798	53.27	43.6	46.77	42.32	-0.093758971	0.998825	Ortholog(s) have tRNA (guanine-N7-)-methyltransferase activity, role in tRNA methylation and nucleolus, tRNA methyltransferase complex localization
orf19.6674	BTS1	58.71	43.59	42.93	44.04	-0.17379217	0.998825	Putative geranylgeranyl diphosphate synthase; repressed by benomyl treatment; Spider biofilm induced
orf19.3573	PEX6	41.25	43.51	56.85	60.3	0.315575611	0.851328	Ortholog(s) have ATPase activity, protein heterodimerization activity, role in fatty acid metabolic process, protein import into peroxisome matrix, receptor recycling, replicative cell aging and cytosol, nucleus, peroxisome localization
orf19.7275	FGR24	46.72	43.5	50.05	49.5	0.087500147	0.998825	Protein encoded in retrotransposon Zorro2 with similarity to retroviral endonuclease-reverse transcriptase proteins; lacks an ortholog in S. cerevisiae; transposon mutation affects filamentous growth
orf19.3342	orf19.3342	50.89	43.46	49.25	41.44	-0.051915828	0.998825	Ortholog(s) have role in protein deubiquitination and cytoplasm, nucleus localization
orf19.4757	NAR1	48.85	43.46	31.53	28.89	-0.393273584	0.720423	Putative cytosolic iron-sulfur (FeS) protein assembly machinery protein; induced by nitric oxide; oxidative stress-induced via Cap1p
orf19.5343	ASH1	41.51	43.43	52.99	55.31	0.232297853	0.951606	GATA-like transcription factor; localizes to daughter cell, hyphal tip cell nuclei; mRNA localization mediated by She3; required for WT virulence and filamentous growth on solid media; Ras1 repressed in yeast, Cyr1 repressed yeast, hyphae
orf19.1865	MCS7	40	43.4	68.26	71.73	0.533347556	0.34341	S. cerevisiae ortholog Mcs7 has a role in reciprocal meiotic recombination; Spider biofilm repressed
orf19.716	orf19.716	57.08	43.38	117.37	97.82	0.839199355	0.014168	Protein of unknown function; Hap43-induced; regulated by Nrg1, Tup1; repressed by alpha pheromone in SpiderM medium; Spider biofilm induced; Bcr1-repressed in RPMI a/a biofilms

orf19.1637	orf19.1637	55.38	43.33	42.23	42.81	-0.146764495	0.998825	Ortholog(s) have cytosol, nucleus localization
orf19.7357	orf19.7357	44.06	43.32	43.04	37.11	-0.090961397	0.998825	Ortholog(s) have role in coenzyme A biosynthetic process and cytosol, nucleus localization
orf19.2181	orf19.2181	55.46	43.32	46.15	42.1	-0.114136688	0.998825	Ortholog of Spathaspora passalidarum NRRL Y-27907 : spas_CGOB_00067 and Candida albicans WO-1 : CAWG_05895
orf19.4123	orf19.4123	42.39	43.32	44.43	38.92	-0.033101975	0.998825	Putative THO complex subunit; possibly an essential gene, disruptants not obtained by UAU1 method; protein newly produced during adaptation to the serum
orf19.1159	orf19.1159	52.52	43.32	82.78	81.28	0.568945693	0.24345	Protein similar to A. nidulans CysA serine O-trans-acetylase; suggests that C. albicans uses an O-acetyl-serine (OAS) pathway of sulfur assimilation; F-12/CO2 early biofilm induced; Spider biofilm induced
orf19.20	RTS1	42.76	43.32	69.08	53.5	0.345007622	0.789669	Putative serine/threonine-protein phosphatase B-type regulatory subunit; transcription is regulated upon yeast-hyphal switch
orf19.3355	ISN1	49.8	43.31	54.52	56.24	0.173643072	0.998825	Putative inosine 5'-monophosphate 5'-nucleotidase; fungal-specific (no human or murine homolog)
orf19.90	orf19.90	55.06	43.29	73.62	69.68	0.400933201	0.616255	Ortholog(s) have cytoplasm localization
orf19.6399	ATS1	52.21	43.18	46.64	52.67	0.045981005	0.998825	Protein required for modification of wobble nucleosides in tRNA; induced upon adherence to polystyrene; regulated by Sef1, Sfu1, and Hap43

orf19.3505	orf19.3505	44.74	43.18	49.92	42.45	0.033946245	0.998825	Ortholog(s) have role in TOR signaling cascade, actin filament bundle assembly, eisosome assembly, endosomal transport, establishment or maintenance of actin cytoskeleton polarity, regulation of cell growth
orf19.4841	SHY1	45.28	43.17	45.33	40.1	-0.043298818	0.998825	Cytochrome c oxidase biosynthesis protein; transcript regulated by Nrg1 and Mig1
orf19.4193	orf19.4193	32	43.13	46.42	51.71	0.246873487	0.946319	Ortholog(s) have Arp2/3 complex binding activity, role in actin cortical patch assembly, actin filament debranching, negative regulation of Arp2/3 complex-mediated actin nucleation and actin cortical patch, cytosol, nucleus localization
orf19.5424	orf19.5424	39.21	43.12	44.44	33.05	-0.071628769	0.998825	Ortholog of Candida albicans WO-1 : CAWG_02376
orf19.3449.2	orf19.3449.2	51.54	43.09	39.88	34.88	-0.237146601	0.951606	Putative mitochondrial phosphatidylglycerophosphatase (PGP phosphatase); essential for cardiolipin biosynthesis; rat catheter biofilm induced
orf19.2110	orf19.2110	40.85	42.98	45.56	53.44	0.154602988	0.998825	Ortholog(s) have arginyltransferase activity, role in protein arginylation and cytosol, nucleus localization
orf19.2073	orf19.2073	48.56	42.98	38.85	30.76	-0.270075886	0.921805	Protein with a multidrug and toxin extrusion protein domain; induced by Mnl1 under weak acid stress
orf19.1272	orf19.1272	56.37	42.9	50.79	49.86	0.013976916	0.998825	Protein of unknown function; may play a role in regulation of cell size; rat catheter biofilm repressed
orf19.2892	orf19.2892	38.37	42.9	46.98	51.21	0.177991467	0.998825	Ortholog of C. dubliniensis CD36 : Cd36_45910, C. parapsilosis CDC317 : CPAR2_501110, Candida tenuis NRRL Y-1498 : CANTEDRAFT_119393 and Debaryomyces hansenii CBS767 : DEHA2E08976g

orf19.7589	orf19.7589	59.04	42.9	55.25	56.26	0.081431337	0.998825	Protein of unknown function; Hap43-repressed gene; transcript induced by elevated CO2
orf19.71	orf19.71	45.41	42.89	55.27	46.51	0.138169207	0.998825	Ortholog(s) have role in ER to Golgi vesicle-mediated transport, protein secretion and COPII vesicle coat, Golgi membrane, cell division site, cell tip, cis-Golgi network, cytosol, nucleus localization
orf19.6054	orf19.6054	53.44	42.86	38.17	36.96	-0.246506201	0.944705	Protein with a Bul1 domain; binds the ubiquitin ligase Rsp5 and is involved in intracellular trafficking of a general amino acid permease Gap1; repressed in an azole-resistant strain overexpressing MDR1; Spider biofilm induced
orf19.6675	orf19.6675	62.95	42.84	46.41	46.39	-0.146729444	0.998825	Protein of unknown function; from Assembly 19; removed from Assembly 20; restored based on comparative genome analysis; F-12/CO2 early biofilm induced
orf19.1956	orf19.1956	51.21	42.78	55.06	48.03	0.084088321	0.998825	Ortholog(s) have role in reciprocal meiotic recombination and mitochondrion localization
orf19.6751	orf19.6751	51.79	42.75	43.94	41.56	-0.105712008	0.998825	Ortholog(s) have tRNA methyltransferase activity, role in cytoplasmic translation, tRNA methylation and cytoplasm localization
orf19.5105	MED15	44.53	42.7	50.85	47.42	0.098354158	0.998825	RNA polymerase II mediator complex subunit; possibly an essential gene, disruptants not obtained by UAU1 method
orf19.4711	orf19.4711	38.73	42.7	45.56	44.82	0.091780273	0.998825	Ortholog of C. dubliniensis CD36 : Cd36_40890, C. parapsilosis CDC317 : CPAR2_401100, Candida tenuis NRRL Y-1498 : CANTEDRAFT_119698 and Debaryomyces hansenii CBS767 : DEHA2G22154g
orf19.6654	orf19.6654	53.63	42.67	45.55	33.12	-0.208684523	0.998825	Predicted membrane transporter, member of the L-amino acid transporter-3 (LAT3) family, major facilitator superfamily (MFS)

orf19.3132	orf19.3132	41.19	42.65	41.18	40.47	-0.044382607	0.998825	Predicted endoplasmic reticulum zinc transporter; induced by nitric oxide
orf19.946	MET14	36.55	42.63	93.06	90.77	0.879470653	0.012599	Putative adenylylsulfate kinase; predicted role in sulfur metabolism; possibly adherence-induced; protein present in exponential and stationary growth phase yeast; F-12/CO2 biofilm induced
orf19.6552	orf19.6552	35.41	42.63	47.75	38.28	0.080234913	0.998825	Flavin-linked sulfhydryl oxidase; predicted localization to endoplasmic reticulum lumen; involved in disulfide bond formation within the ER; Spider biofilm induced
orf19.3177	orf19.3177	45.61	42.55	54.37	47.03	0.117432416	0.998825	Ortholog(s) have 2,5-diamino-6-ribitylamino-4(3H)-pyrimidinone 5'-phosphate deaminase activity, pseudouridine synthase activity, role in riboflavin biosynthetic process, tRNA pseudouridine synthesis and cytosol localization
orf19.6716	ABD1	47.12	42.52	52.48	44.49	0.054838208	0.998825	SAM-dependent RNA methyltransferase; methylates mRNA 5' cap; binds phosphorylated RNA Pol II C-term domain peptide; does not bind mRNA TPase and mRNA GTase (Cet1,Cgt1); functional homolog of S. cerevisiae Abd1; rat catheter biofilm induced
orf19.6517	RAD14	45.33	42.48	40.66	32.17	-0.177031428	0.998825	Putative DNA repair protein; transcription is regulated upon yeast-hyphal switch; flucytosine repressed
orf19.4236	RET2	42.92	42.47	78.83	65.68	0.547953397	0.305349	Delta subunit of the coatomer complex (COPI); coats Golgi-derived transport vesicles; involved in retrograde transport between Golgi and ER; interacts with Crk1 in the two-hybrid system; Spider biofilm repressed
orf19.5016	orf19.5016	44.07	42.41	32.42	37.89	-0.200562032	0.998825	Ortholog(s) have role in cellular response to UV and cytosol, nucleus localization
orf19.1765	orf19.1765	57.76	42.4	67.46	67.52	0.309518771	0.846842	Secreted protein; fluconazole-induced

orf19.3123.2	orf19.3123.2	60.16	42.32	55.11	54.19	0.062548252	0.998825	Ortholog(s) have cytidine deaminase activity, role in cytidine catabolic process, deoxycytidine catabolic process, pyrimidine-containing compound salvage and cytosol, nucleus localization
orf19.308	orf19.308	52.24	42.28	24.9	16.79	-0.716916325	0.149488	Ortholog(s) have role in nucleobase-containing compound transport and plasma membrane localization
orf19.3237	orf19.3237	49.16	42.23	46.08	45.27	-0.007389512	0.998825	Ortholog(s) have cytosol, mitochondrion, nucleus localization
orf19.758	orf19.758	37.88	42.19	31.84	28.59	-0.257797508	0.938642	Ortholog(s) have signal sequence binding activity, role in vacuolar transport and late endosome localization
orf19.5901	PKC1	47.1	42.17	60.16	56.9	0.272013507	0.894293	Protein kinase C; functional homolog of S. cerevisiae Pkc1p; mutant has abnormal yeast-form cell morphology and increased cell lysis; activated by phosphatidylserine; target of antifungal, cercosporamide; R400P mutant is activated
orf19.6596	orf19.6596	56.24	42.12	52.37	40.34	-0.071066506	0.998825	Putative esterase; possibly transcriptionally regulated by Tac1; induced by Mnl1 under weak acid stress; protein present in exponential and stationary growth phase yeast cultures; Spider biofilm repressed
orf19.5226	WRS1	48.86	42.1	44.78	45.64	-0.00695553	0.998825	Putative tRNA-Trp synthetase; genes encoding ribosomal subunits, translation factors, tRNA synthetases are downregulated upon phagocytosis by murine macrophages
orf19.3399	orf19.3399	43.78	42.08	47.44	38.83	-0.009972304	0.998825	Ortholog(s) have histone binding activity, role in histone exchange and Swr1 complex, cytoplasm localization
orf19.7486	MRPL6	46.41	42.02	51.18	46.53	0.098821567	0.998825	Putative mitochondrial ribosomal protein

orf19.6086	LEU4	48.12	42.02	50.33	47.01	0.066854512	0.998825	Putative 2-isopropylmalate synthase; regulated by Nrg1, Mig1, Tup1, Gcn4; induced by human whole blood or PMNs; macrophage/pseudohyphal-repressed after 16h; Spider biofilm repressed
orf19.1150.1	orf19.1150.1	66.47	42.01	49.72	48.72	-0.09190381	0.998825	Ortholog of C. dubliniensis CD36 : Cd36_10990, C. parapsilosis CDC317 : CPAR2_804585, Candida tenuis NRRL Y-1498 : cten_CGOB_00256 and Debaryomyces hansenii CBS767 : DEHA2F10406g
orf19.2400	orf19.2400	46.11	41.99	43.72	39.69	-0.054103112	0.998825	Ortholog(s) have role in mRNA splicing, via spliceosome, maturation of SSU-rRNA, positive regulation of ATPase activity, positive regulation of helicase activity
orf19.1055	CDC3	47.81	41.98	69.86	65.77	0.423655096	0.579712	Septin; essential for viability; functional homolog of S. cerevisiae Cdc3p; down-regulation associated with azole resistance; macrophage/pseudohyphal-repressed; virulence-group-correlated expression; gene has intron
orf19.4168	orf19.4168	46.23	41.94	39.52	44.92	-0.040450769	0.998825	Ortholog(s) have ribonuclease MRP activity, ribonuclease P activity, role in intronic box C/D snoRNA processing, mRNA cleavage and nucleolar ribonuclease P complex, ribonuclease MRP complex localization
orf19.6003	orf19.6003	50.74	41.91	48.12	38.79	-0.074402654	0.998825	Protein of unknown function; role in intracellular signal transduction; Spider biofilm induced
orf19.866	RAD32	61.37	41.9	58.13	38.32	-0.078523652	0.998825	Protein similar to S. cerevisiae protein with role in nucleotide excision repair; down-regulation associated with azole resistance; Hap43p-repressed gene
orf19.3770	ARG8	39.58	41.89	37.3	38.1	-0.093401216	0.998825	Putative acetylornithine aminotransferase; Gcn2, Gcn4 regulated; rat catheter biofilm induced; Spider biofilm induced
orf19.4357	orf19.4357	52.68	41.84	46.97	51.18	0.025637126	0.998825	Putative protein similar to S. cerevisiae Mgr3p, a subunit of the i-AAA protease supercomplex that degrades misfolded mitochondrial proteins

orf19.1481	HAP42	33.78	41.83	43.26	56.83	0.256061867	0.938404	Predicted transcription factor; possibly an essential gene, disruptants not obtained by UAU1 method
orf19.3710	YHB5	69.95	41.74	71.47	48.76	0.06310955	0.998825	Flavohemoglobin-related protein; not required for normal NO resistance; predicted globin/FAD-binding/NAD(P)-binding domains but lacks some conserved residues of flavohemoglobins; filament induced; rat catheter and Spider biofilm induced
orf19.5432	TPT1	60.6	41.73	47.49	56.71	0.00690529	0.998825	tRNA 2'-phosphotransferase; enzyme of tRNA splicing; functionally complements viability of S. cerevisiae tpt1 null mutant
orf19.1505	orf19.1505	42.28	41.72	31.85	33.46	-0.247223266	0.946319	Ortholog(s) have cytoplasm localization
orf19.905	AVT7	43.93	41.66	55.09	52.24	0.211346906	0.987362	Ortholog of S. cerevisiae Avt7 transporter; repressed upon adherence to polystyrene; constitutive expression independent of MTL or white-opaque status; Spider biofilm induced
orf19.4755	KEX2	49.4	41.65	56.19	50.61	0.156067908	0.998825	Subtilisin-like protease (proprotein convertase); processes aspartyl proteinase Sap2; required for hyphal growth and wild-type virulence in mice; functional homolog of S. cerevisiae Kex2, which processes alpha-factor; Tup1-repressed
orf19.3546	PEX2	43.71	41.62	52.75	55.83	0.231426412	0.951606	Ortholog(s) have ubiquitin-protein ligase activity, role in fatty acid metabolic process, protein import into peroxisome matrix and peroxisomal membrane localization
orf19.2246	orf19.2246	53.68	41.54	59.95	57.7	0.206143494	0.989657	Ortholog(s) have cytosol, nucleus localization
orf19.881	DFG16	48.81	41.54	36.26	30.74	-0.298535219	0.880321	Rim101 pathway protein that acts in Rim101 processing; regulates filamentation in response to alkaline pH; required for host tissue invasion during infection; flow model biofilm induced

orf19.6573	BEM2	39.78	41.52	35.28	36.63	-0.13098913	0.998825	Putative Rho1p GTPase activating protein (GAP); serum-induced transcript; Spider biofilm induced; flow model biofilm repressed
orf19.3088	orf19.3088	48.34	41.47	32.02	33.8	-0.288030178	0.893138	bZIP transcription factor; possibly transcriptionally regulated upon hyphal formation; Hap43; F-12/CO2 early biofilm induced; Spider biofilm induced
orf19.3139	orf19.3139	36.6	41.45	38	42.34	0.015448388	0.998825	Putative NADP-dependent oxidoreductase; Hap43-repressed; induced by benomyl treatment; oxidative stress-induced via Cap1; rat catheter biofilm repressed
orf19.593	FGR32	47.4	41.42	49.18	45.03	0.057262194	0.998825	Protein similar to S. cerevisiae Swa2p; induced upon adherence to polystyrene; transposon mutation affects filamentous growth; Hap43p-repressed gene
orf19.7666	orf19.7666	49.71	41.41	49.7	50.14	0.087910219	0.998825	Predicted membrane transporter, member of the anion:cation symporter (ACS) family, major facilitator superfamily (MFS)
orf19.820	SDS22	41.74	41.39	47.62	45.1	0.102785105	0.998825	Putative protein serine-threonine phosphatase; macrophage/pseudohyphal-repressed
orf19.6712	orf19.6712	39.62	41.38	46.33	46.31	0.115576162	0.998825	Ortholog(s) have COPI-coated vesicle, Golgi apparatus localization
orf19.4293	orf19.4293	48.22	41.36	34.12	36.05	-0.23468419	0.957711	Ortholog(s) have role in iron-sulfur cluster assembly, ribosomal large subunit export from nucleus, tRNA wobble uridine modification and cytosol, nucleus localization
orf19.1796	orf19.1796	58.71	41.36	55.3	53.05	0.072954762	0.998825	Putative glyoxylate reductase; acts on glyoxylate and hydroxypyruvate substrates; Spider biofilm repressed

orf19.4624	HRT2	43.84	41.35	67.29	73.42	0.512197621	0.387957	Protein described as having a role in Ty3 transposition; repressed in hyphae; stationary phase enriched protein; rat catheter and Spider biofilm repressed
orf19.5129	orf19.5129	43.29	41.32	52.92	59.57	0.292135252	0.878136	Ortholog of C. dubliniensis CD36 : Cd36_72910, C. parapsilosis CDC317 : CPAR2_704100, Candida tenuis NRRL Y-1498 : CANTEDRAFT_113193 and Debaryomyces hansenii CBS767 : DEHA2E23100g
orf19.6801	RPD31	51.82	41.31	46.24	39.28	-0.096095557	0.998825	Putative histone deacetylase; involved in regulation of white-opaque switch; Spider biofilm repressed
orf19.3373	orf19.3373	31.59	41.28	10.17	10.76	-0.921677677	NA	Putative protein of unknown function; transcript upregulated in clinical isolates from HIV+ patients with oral candidiasis; Spider biofilm induced
orf19.7227	orf19.7227	24.67	41.28	26.21	22.99	-0.258727182	0.946319	Protein phosphatase inhibitor; Hap43-repressed; homozygous Tn insertion decreases colony wrinkling but does not block hyphal growth in liquid media; mutation confers hypersensitivity to toxic ergosterol analog; Spider biofilm induced
orf19.4829	DOA1	53.09	41.27	55.04	48.81	0.094866249	0.998825	WD repeat protein; required for ubiquitin-mediated protein degradation; role in control of cellular ubiquitin levels; may promote efficient NHEJ in postdiauxic/stationary phase; Spider biofilm repressed
orf19.1190	STV1	50.85	41.27	62.38	54.06	0.229329035	0.951606	Predicted subunit a of vacuolar proton-translocating ATPase V0 domain, Golgi isoform
orf19.4114	FAA2-1	49.1	41.19	59.5	49.8	0.185410485	0.998825	Predicted long chain fatty acid CoA ligase; upregulated upon phagocytosis; induced by nitric oxide independent of Yhb1
orf19.2534	PIN4	37.72	41.19	41.88	42.36	0.049587944	0.998825	Protein with similarity to S. cerevisiae Pin4p; transposon mutation affects filamentous growth

orf19.6904	GCN3	44.44	41.19	45.13	43.49	0.025557852	0.998825	Putative translation initiator; downregulated in the presence of human whole blood or polymorphonuclear (PMN) cells
orf19.1699	BZZ1	44.18	41.18	62.82	50.79	0.286830211	0.880321	Protein similar to S. cerevisiae Bzz1p, which is an SH3 domain protein involved in the regulation of actin polymerization
orf19.4699	orf19.4699	43.52	41.17	41.34	35.13	-0.116345108	0.998825	Putative phospholipase of patatin family; similar to S. cerevisiae Tgl3p; predicted Kex2p substrate
orf19.4234	orf19.4234	57.92	41.15	32.36	31.01	-0.432087461	0.62036	Protein of unknown function; Hap43-induced gene; Ctr86 ortholog in S. cerveisiae is essential; Spider biofilm induced
orf19.6847	orf19.6847	41.04	41.14	41.16	33.42	-0.10749049	0.998825	Putative tRNA U44 2'-O-methyltransferase; virulence-group-correlated expression; induced during oral infection; mutants have reduced ability to damage oral epithelial cells; early-stage flow model biofilm induced
orf19.4125	PZF1	45.04	41.09	40.39	42.93	-0.041448463	0.998825	C2H2 transcription factor; Hap43-induced; rat catheter and Spider biofilm induced
orf19.1180	orf19.1180	50.4	41.09	66.87	55.62	0.301658249	0.859429	Putative 2-aminoadipate transaminase; rat catheter and Spider biofilm repressed
orf19.6442	PRP8	38.7	40.98	41.79	42.45	0.037875735	0.998825	Protein similar to S. cerevisiae Prp8, a component of the U4/U6-U5 snRNP complex; repressed by alpha pheromone in SpiderM medium
orf19.4984	orf19.4984	42.79	40.98	35.64	38.13	-0.128085443	0.998825	Has domain(s) with predicted chitin binding, chitinase activity and role in carbohydrate metabolic process, cell wall macromolecule catabolic process, chitin catabolic process

orf19.2069	SMF3	44.7	40.95	45.78	44.44	0.036532851	0.998825	Putative vacuolar iron transporter; alkaline upregulated; caspofungin repressed; induced by Mnl1 under weak acid stress; Hap43-repressed
orf19.3360	orf19.3360	32.79	40.82	29.3	30.57	-0.196855689	0.998825	Protein of unknown function; flow model biofilm induced; Spider biofilm induced
orf19.7260	orf19.7260	40.32	40.79	36.31	33.86	-0.146057427	0.998825	Protein with a predicted NADP-dependent oxidoreductase or aldo-keto reductase domain; Hap43-repressed; Spider biofilm repressed
orf19.278	orf19.278	47.8	40.73	54.71	47.65	0.138322477	0.998825	Protein of unknown function; required for adhesion to abiotic substrate; Spider biofilm induced
orf19.6044	MOB2	54.78	40.72	51.43	50.81	0.055439594	0.998825	Mob1/phocein domain protein of RAM signaling network; cell wall integrity; role in cell separation, cortical actin polarization; required for hyphal growth; phosphorylated by Cdc28 on hyphal induction; activates Cbk1; mRNA binds She3
orf19.1914	FAV3	36.69	40.71	45.88	42.95	0.117156775	0.998825	Putative alpha-1,6-mannanase; induced by mating factor in MTLa/MTLa opaque cells
orf19.3577.1	orf19.3577.1	52.3	40.66	51.33	57.1	0.146210363	0.998825	Ortholog(s) have mitochondrial intermembrane space localization
orf19.6580	orf19.6580	43.72	40.66	35.6	25.65	-0.301172592	0.881124	Protein of unknown function; Spider biofilm induced
orf19.514	SNP3	47.06	40.62	51.91	43.35	0.066689504	0.998825	Putative U6 snRNA-associated protein; transcript regulated by Mig1

orf19.6225	PCL7	37.73	40.6	38.44	39.49	-0.033244828	0.998825	Putative cyclin-like protein; possible Pho85 cyclin; hyphal repressed; induced by Mnl1 under weak acid stress
orf19.3605	PEX17	54.01	40.56	42.57	46.75	-0.058505353	0.998825	Putative peroxin
orf19.504	orf19.504	44.68	40.54	50.48	44.85	0.089802108	0.998825	Ortholog(s) have ATP-dependent 3'-5' DNA helicase activity, role in nucleosome mobilization and Ino80 complex, cytosol localization
orf19.1546	orf19.1546	39.9	40.53	49.25	49.87	0.189472024	0.998825	Membrane-localized protein of unknown function
orf19.6033	CMP1	43.27	40.52	52.35	45.56	0.143447049	0.998825	Catalytic subunit of calcineurin (Ca[2+]-calmodulin-regulated S/T protein phosphatase); required for wild-type virulence, resistance to high pH, Na(+), Li(+), Mn(2+), and fluconazole tolerance; micafungin is fungicidal to null mutant
orf19.3797	orf19.3797	50.54	40.47	50.65	44.76	0.045798646	0.998825	Ortholog(s) have structural constituent of ribosome activity and mitochondrial large ribosomal subunit localization
orf19.5962	HGT4	44.32	40.44	44.53	40.33	0.002722728	0.998825	Glucose and galactose sensor; fermentation, filamentation, virulence roles; 20-member glucose transporter family; extended C terminus; high-glucose repressed; macrophage induced; flow model biofilm repressed; Spider biofilm induced
orf19.7201	SLA2	51.38	40.42	58.43	46.8	0.13554667	0.998825	Actin binding protein with roles in growth control and morphogenesis; required for alkaline pH-induced hyphal formation; localized to actin patches; rat catheter biofilm repressed
orf19.6360	orf19.6360	41.82	40.39	27.24	37.15	-0.231876128	0.969808	Protein involved in pre-mRNA splicing; Spider biofilm induced

orf19.1832	FCY23	48.92	40.32	36.82	33.3	-0.235614156	0.957711	Putative transporter; Gcn4p-regulated; more similar to S. cerevisiae Tpn1p, which is a vitamin B6 transporter, than to purine-cytosine permeases
orf19.3047	orf19.3047	45.73	40.3	45.66	39.73	-0.008556222	0.998825	Protein kinase-related protein, required for normal sensitivity to caspofungin
orf19.6057	orf19.6057	56.94	40.25	48.68	51.49	0.025914997	0.998825	Ortholog(s) have 3-methyl-2-oxobutanoate hydroxymethyltransferase activity, role in pantothenate biosynthetic process, respiratory chain complex IV assembly and mitochondrion localization
orf19.2723	HIT1	57.95	40.24	51.62	30.42	-0.17939106	0.998825	Ortholog of S. cerevisiae Hit1; protein of unknown function required for high temperature growth; flow model biofilm induced; Spider biofilm induced
orf19.182	orf19.182	40.58	40.17	40.99	37.16	-0.045218156	0.998825	Ortholog(s) have rRNA (guanine-N1-)-methyltransferase activity, role in rRNA modification and endoplasmic reticulum, mitochondrion localization
orf19.6707	orf19.6707	45.97	40.17	38.48	29.96	-0.229724782	0.968573	Predicted vacuolar protein with a calcineurin-like phosphoesterase domain; repressed by alpha pheromone in SpiderM medium
orf19.3190	HAL9	44.22	40.08	45.25	43.05	0.03733017	0.998825	Putative Zn(II)2Cys6 transcription factor; gene in zinc cluster region of Chr. 5; induced by Mnl1 in weak acid; similar to S. cerevisiae Hal9, a putative transcription factor involved in salt tolerance
orf19.7042	orf19.7042	51.37	40.04	4.39	2.3	-1.645695446	NA	Protein of unknown function; induced by benomyl or in an azole-resistant strain overexpressing MDR1; induced by nitric oxide; Spider biofilm induced
orf19.1490	MSB2	30.08	40.04	37.09	39.02	0.064890656	0.998825	Mucin family adhesin-like protein; cell wall damage sensor; required for Cek1 phosphorylation by cell wall stress; Rim101-repressed; activation releases extracellular domain into medium; Spider biofilm induced

orf19.5776	TOM1	37.38	40.03	51.29	52.77	0.287481721	0.886087	Putative E3 ubiquitin ligase; transcript regulated by Nrg1 and Mig1
orf19.4866	CPP1	40.64	40.01	43.32	42.06	0.038041621	0.998825	VH1 family MAPK phosphatase; regulates Cst20-Hst7-Cek1-Cph1 filamentation pathway; represses yeast-hyphal switch; required for virulence in mice; yeast-enriched; induced by alpha pheromone in SpiderM medium; Spider biofilm induced
orf19.1161	PLD1	45.12	40.01	59.3	56.04	0.299048018	0.869089	Phospholipase D1; required for phosphatidic acid and for most diacylglycerol production; required for wild-type mouse virulence, but not rat oral virulence; mutant defect in hyphal growth on solid substrates; similar to S. cerevisiae Spo14p
orf19.7492	SWC4	45.55	40	44.26	34.85	-0.090849688	0.998825	Subunit of the NuA4 histone acetyltransferase complex
orf19.844	STE11	41.35	39.99	39.9	31.54	-0.120849163	0.998825	Protein similar to S. cerevisiae Ste11p; mutants are sensitive to growth on H2O2 medium
orf19.4418.1	orf19.4418.1	65.76	39.96	60.44	44.99	-0.015791759	0.998825	Ortholog of C. parapsilosis CDC317 : CPAR2_501415, Candida tenuis NRRL Y-1498 : CANTEDRAFT_105081, Debaryomyces hansenii CBS767 : DEHA2D12298g and Pichia stipitis Pignal : psti_CGOB_00211
orf19.7622	SPT3	42.82	39.94	39.47	34.3	-0.130646055	0.998825	Functional homolog of S. cerevisiae Spt3p; required for virulence in mouse systemic infection; homozygous null mutant is hyperfilamentous
orf19.807	CHS5	47.07	39.91	78.21	72.36	0.566228416	0.266191	Putative chitin biosynthesis protein; fungal-specific; repressed upon yeast-to-hypha switch; rat catheter biofilm repressed
orf19.2794	orf19.2794	41.01	39.85	35.67	49.28	0.040643403	0.998825	Putative non-specific single-domain racemase; regulated by Gcn4p; repressed in response to amino acid starvation (3-AT treatment); alkaline upregulated; macrophage-induced protein

orf19.3345	SIZ1	37.96	39.85	42.24	43.87	0.085232392	0.998825	Possible SUMO/Smt3 ligase; Rim101-repressed
orf19.1261	CGT1	50.5	39.83	48.71	36.41	-0.075364382	0.998825	mRNA 5' guanylyltransferase; small subunit of mRNA capping enzyme; binds an RNA Pol II C-terminal domain peptide; Cet1p and Cgt1p form a 2:1 complex; functional homolog of S. cerevisiae Ceg1p
orf19.2248	ARE2	41.25	39.81	54.27	60.71	0.345497464	0.795378	Acyl CoA:sterol acyltransferase; uses cholesterol and oleoyl-CoA substrates; protoberberine derivative drug inhibits enzyme activity; ketoconazole-induced; Hap43-repressed; flow model biofilm induced; Spider biofilm induced
orf19.943	FET33	44.98	39.79	44.46	41.46	-0.008109732	0.998825	Putative multicopper ferro-O2-oxidoreductase; repressed in core caspofungin response; fails to complement S. cerevisiae fet3 iron-related phenotypes; rat catheter and Spider biofilm induced
orf19.2915	orf19.2915	42.62	39.77	42.77	35.15	-0.069060524	0.998825	Ortholog(s) have ATPase activity, GTPase activity, adenosine-diphosphatase activity, cytidine diphosphatase activity, guanosine-diphosphatase activity, nucleoside-triphosphate diphosphatase activity, uridine-diphosphatase activity
orf19.2260	orf19.2260	44.37	39.7	36.85	36.49	-0.141116779	0.998825	Putative transcription factor with zinc finger DNA-binding motif
orf19.3234.1	SFT1	37.61	39.69	40.29	51.38	0.141411901	0.998825	Putative Golgi v-SNARE; Plc1-regulated; Spider biofilm induced
orf19.100	orf19.100	46.12	39.67	32.45	34.57	-0.240580758	0.951606	Alpha/beta hydrolase and lipase domain protein; Hap43-repressed; Spider and flow model biofilm induced
orf19.3994	orf19.3994	45.49	39.65	74.1	60.57	0.469306697	0.486965	Dolichyl-diphosphooligosaccharide-protein glycotransferase; predicted role in protein N-linked glycosylation, protein O-linked mannosylation; Spider biofilm repressed

orf19.3858	orf19.3858	41.39	39.65	49.63	38.06	0.069150061	0.998825	Protein of unknown function; rat catheter and Spider biofilm induced
orf19.6048	orf19.6048	43.93	39.64	46.85	44.8	0.081322557	0.998825	Protein of unknown function; Spider biofilm induced
orf19.3949	orf19.3949	43.53	39.61	34.79	27.35	-0.289680614	0.894293	Ortholog(s) have chromatin binding, histone binding activity
orf19.5770	OPT8	43.8	39.59	35.48	30.25	-0.247791062	0.946319	Oligopeptide transporter; similar to Opt1 and to S. cerevisiae Ygl114wp, but not other OPTs; induced by nitric oxide, amphotericin B; expression of OPT6, 7, 8 does not complement mutants lacking Opt1, Opt2, and Opt3; Spider biofilm induced
orf19.1730	orf19.1730	40.6	39.58	54.19	49.33	0.229485286	0.957711	HMG-box protein; Spider biofilm repressed
orf19.6260	orf19.6260	45.82	39.57	45.7	42.3	0.014401117	0.998825	Ubiquitin-specific protease; cleaves ubiquitin from ubiquitinated proteins; Spider biofilm induced
orf19.2465	POL32	49.93	39.56	45.02	40.07	-0.063418686	0.998825	Putative subunit of DNA polymerase delta, involved in chromosomal DNA replication; cell-cycle regulated periodic mRNA expression
orf19.2112	orf19.2112	38.37	39.53	46.9	46.18	0.161898823	0.998825	snRNP U5 splicing factor component; involved in positioning the 3' splice site during the 2nd catalytic step of splicing; Spider biofilm induced
orf19.5681	orf19.5681	43.44	39.52	35.02	38.37	-0.128517912	0.998825	Ortholog of C. dubliniensis CD36 : Cd36_50190, C. parapsilosis CDC317 : CPAR2_302680, Candida tenuis NRRL Y-1498 : CANTEDRAFT_109949 and Debaryomyces hansenii CBS767 : DEHA2G00814g

orf19.697	orf19.697	43.96	39.51	37.9	39.67	-0.078139458	0.998825	Ortholog(s) have mitochondrial outer membrane localization
orf19.797	BAT21	32.68	39.49	42.4	37.48	0.075475227	0.998825	Putative branched chain amino acid aminotransferase; regulated by Gcn4, Gcn2; induced in response to amino acid starvation (3-aminotriazole treatment); early-stage flow model biofilm formation
orf19.6070	ENA2	38.19	39.48	102.65	98.19	1.026333534	0.001629	Putative sodium transporter; induced by ciclopirox olamine; alkaline induced by Rim101; repressed by high-level peroxide stress; induced in oral candidiasis clinical isolates; possibly essential gene; rat catheter and Spider biofilm induced
orf19.2731	orf19.2731	34.61	39.45	44.23	46.75	0.189578106	0.998825	Ortholog of Debaryomyces hansenii CBS767 : DEHA2E16016g, Pichia stipitis Pignal : PICST_38487, Spathaspora passalidarum NRRL Y-27907 : SPAPADRAFT_58603 and Candida tropicalis MYA-3404 : CTRG_00248
orf19.6528	orf19.6528	48.29	39.44	62.64	51.91	0.274431529	0.894293	Ortholog of C. dubliniensis CD36 : Cd36_71740, C. parapsilosis CDC317 : CPAR2_703440, Candida tenuis NRRL Y-1498 : CANTEDRAFT_102923 and Debaryomyces hansenii CBS767 : DEHA2C03190g
orf19.3128	orf19.3128	37.48	39.39	49.29	45.33	0.196542773	0.998825	Ortholog(s) have syntaxin binding activity and role in ER to Golgi vesicle-mediated transport, positive regulation of SNARE complex assembly, retrograde vesicle-mediated transport, Golgi to ER, vesicle fusion with Golgi apparatus
orf19.1744	HEM4	43.35	39.38	34.4	36.01	-0.155503604	0.998825	Putative uroporphyrinogen III synthase; induced in high iron or elevated CO2; alkaline, Hap43-repressed
orf19.4616	POL30	41.3	39.33	50.69	46.28	0.180440967	0.998825	Similar to proliferating cell nuclear antigen (PCNA); RNA abundance regulated by tyrosol, cell density; induced by flucytosine, interaction with macrophages; stationary phase enriched protein; rat catheter and Spider biofilm repressed
orf19.4544	orf19.4544	40.93	39.32	33.93	32.32	-0.186864996	0.998825	Ortholog of C. dubliniensis CD36 : Cd36_01640, C. parapsilosis CDC317 : CPAR2_106830, Candida tenuis NRRL Y-1498 : cten_CGOB_00039 and Debaryomyces hansenii CBS767 : DEHA2B06578g

orf19.7558	YTA6	41.27	39.31	40.49	36.59	-0.045028218	0.998825	Protein similar to S. cerevisiae Yta6p ATPase but ortholog of S. cervisiae Sap1; transposon mutation affects filamentous growth; induced by Mnl1 under weak acid stress
orf19.4101	orf19.4101	41.29	39.28	24.57	27.22	-0.39091513	0.743771	Ortholog of C. parapsilosis CDC317 : CPAR2_104840, Candida tenuis NRRL Y-1498 : CANTEDRAFT_116070, Debaryomyces hansenii CBS767 : DEHA2F04994g and Pichia stipitis Pignal : psti_CGOB_00085
orf19.2084	CDH1	42.92	39.28	39.5	36.6	-0.080352618	0.998825	Protein involved in regulation of mitosis; similar to S. cerevisiae Cdh1, which is an APC/C component; transcriptionally induced by Mnl1 under weak acid stress
orf19.4736	orf19.4736	41.97	39.26	52.6	42.29	0.145948308	0.998825	Ortholog(s) have alkaline phosphatase activity and role in cellular response to cadmium ion, detoxification of cadmium ion, nicotinamide nucleotide metabolic process, protein dephosphorylation
orf19.4175	TOK1	45.41	39.25	35.02	34.92	-0.178927365	0.998825	Outwardly rectifying, noisily gated potassium channel; modulates sensitivity to human salivary histatin (Hst5); very similar to S. cerevisiae Tok1p; Bcr1-repressed in RPMI a/a biofilms
orf19.7299.1	orf19.7299.1	30.3	39.24	28.16	24.71	-0.238390516	0.964275	Ortholog of Candida albicans WO-1 : CAWG_02179
orf19.5003	orf19.5003	50.62	39.23	40.11	42.71	-0.08386288	0.998825	Ortholog(s) have GTPase regulator activity and cytoplasm, nuclear envelope localization
orf19.4506	LYS22	41.81	39.2	60.79	63.29	0.426686192	0.594776	Putative homocitrate synthase; repressed by nitric oxide and by hypoxia; protein level decreases in stationary phase cultures; induced by ketoconazole, Spider biofilm induced; flow model biofilm repressed
orf19.397	orf19.397	60.16	39.2	59.55	65.74	0.244871759	0.938404	Ortholog(s) have structural constituent of ribosome activity and cytosol, mitochondrial large ribosomal subunit, nucleus localization

orf19.5942	orf19.5942	45.29	39.1	44.27	37.07	-0.043745607	0.998825	Zinc finger protein orthologous to S. cerevisiae Itt1; repressed by adherence to polystyrene; Spider biofilm induced
orf19.2646	ZCF13	40.12	39.02	41.68	38.57	0.014956317	0.998825	Predicted Zn(II)2Cys6 transcription factor; similar to but not the true ortholog of S. cerevisiae Hap1; mutants display decreased colonization of mouse kidneys
orf19.1474	SLA1	40.42	38.99	54.08	49.84	0.263694717	0.921805	Protein required for assembly of the cortical actin cytoskeleton; contains three SH3 domains; transcription is regulated by Nrg1 and Mig1; flow model biofilm repressed
orf19.6667	orf19.6667	40.53	38.97	33.49	36.55	-0.131561207	0.998825	Predicted histone deacetylase activity; Spider biofilm induced
orf19.2875	orf19.2875	38.48	38.93	36.54	40.2	-0.007949164	0.998825	Ortholog of S. pombe SPAC1071.09c DNAJ domain protein; Hap43-induced gene
orf19.4379	PRP13	41.82	38.89	60.07	52.33	0.316386418	0.853836	Putative integral inner mitochondrial membrane protein with similarity to exonucleases
orf19.3050	AGE1	37.69	38.89	46.91	46.06	0.174485499	0.998825	Putative GTPase activator; induced in low iron; rat catheter biofilm repressed
orf19.7565	GNP3	44.96	38.82	50.4	38.71	0.046746466	0.998825	Putative high-affinity glutamine permease; fungal-specific (no human or murine homolog)
orf19.2222	orf19.2222	51.19	38.79	45.12	40.33	-0.062817463	0.998825	Putative casein kinase; plasma membrane-localized

orf19.6756	orf19.6756	48.74	38.76	51.99	36.96	0.00147252	0.998825	Ortholog(s) have Rab GTPase binding activity and Golgi apparatus, endoplasmic reticulum localization
orf19.3311	IFD3	42.67	38.71	39.16	55.58	0.138040219	0.998825	Putative aldo/keto reductase; Mig1-regulated
orf19.7037	YAE1	50.56	38.71	40.77	41.11	-0.096305427	0.998825	Ortholog(s) have role in ribosomal large subunit biogenesis, translational initiation and cytoplasm, nucleus localization
orf19.6650	orf19.6650	46.8	38.7	37.61	32.55	-0.189360953	0.998825	Ortholog of C. dubliniensis CD36 : Cd36_30990, C. parapsilosis CDC317 : CPAR2_204660, Candida tenuis NRRL Y-1498 : CANTEDRAFT_104939 and Debaryomyces hansenii CBS767 : DEHA2G23760g
orf19.7468	orf19.7468	39.17	38.7	29.41	28.02	-0.291264534	0.894293	Putative transcriptional activator; expression upregulated during growth in the mouse cecum; Spider biofilm induced
orf19.261	orf19.261	39.21	38.67	41.73	42.13	0.062564117	0.998825	Ortholog(s) have dolichol kinase activity, role in protein glycosylation and endoplasmic reticulum membrane localization
orf19.3212	MID1	41.1	38.65	39.78	30.92	-0.121696108	0.998825	Putative stretch-activated Ca2+ channel of the high affinity calcium uptake system; role in thigmotropism; decreased transcription is observed upon fluphenazine treatment or in an azole-resistant strain with CDR1 and CDR2 overexpression
orf19.499	orf19.499	47.25	38.61	43.64	41.16	-0.018783074	0.998825	Ortholog(s) have S-adenosylmethionine-dependent methyltransferase activity, role in translational readthrough and mitochondrion localization
orf19.6496	TRS33	37.08	38.58	42.7	34.26	0.001213914	0.998825	Putative TRAPP complex subunit; constitutive expression independent of MTL or white-opaque status

orf19.386	SAM4	47.27	38.57	32.64	27.79	-0.325469642	0.858075	Putative S-adenosylmethionine-homocysteine methyltransferase; Hap43-repressed; alkaline induced; Spider biofilm repressed
orf19.5857	orf19.5857	32.21	38.57	46.1	47.35	0.24753822	0.946319	Ortholog of C. dubliniensis CD36 : Cd36_84070, C. parapsilosis CDC317 : CPAR2_102720, Candida tenuis NRRL Y-1498 : CANTEDRAFT_98617 and Debaryomyces hansenii CBS767 : DEHA2D07392g
orf19.4092	orf19.4092	39.75	38.55	34.54	42.76	-0.018279949	0.998825	Trimeric GatFAB AmidoTransferase(AdT) complex subunit; involved in the formation of Q-tRNAQ; repressed by fluphenazine treatment or in an azole-resistant strain that overexpresses CDR1; Spider biofilm induced
orf19.163	PAN6	36.36	38.53	38.24	37.83	0.003255798	0.998825	Ortholog(s) have pantoate-beta-alanine ligase activity, role in pantothenate biosynthetic process and cytosol, nucleus localization
orf19.3110	orf19.3110	42.27	38.52	29.55	28.56	-0.297195475	0.888717	Possible mannosyltransferase; Spider biofilm induced
orf19.5700	TLO11	53.24	38.5	82.76	82.06	0.61760691	0.182093	Member of a family of telomere-proximal genes of unknown function; may be spliced in vivo
orf19.1723	orf19.1723	58.88	38.47	70.18	52.68	0.234811877	0.946319	Ortholog(s) have role in response to purine-containing compound and cytoplasm, nucleus localization
orf19.3960	HYS2	46.6	38.47	40.34	36.92	-0.101665997	0.998825	Putative DNA polymerase III (delta) subunit with a predicted role in DNA replication and DNA repair; cell-cycle regulated periodic mRNA expression
orf19.2315	RTG3	44.05	38.44	39.3	29.08	-0.18349688	0.998825	Putative transcription factor with bZIP DNA-binding motif; mutants are sensitive to cations and antifungal drugs, and defective in filamentous growth

orf19.201	orf19.201	38.85	38.44	40.68	48.79	0.143564324	0.998825	Protein of unknown function; unmerged from orf19.202 in a revision of Assembly 21
orf19.6930	SOL1	48.05	38.43	38.61	43.38	-0.051189398	0.998825	Cell cycle regulator; partial functional homolog of S. cerevisiae Sic1p; protein degradation regulated by SCF(CDC4) pathway; CDK phosphorylation predicted
orf19.6460	PEX1	37.54	38.42	43.54	42.4	0.109622479	0.998825	Ortholog(s) have ATPase activity, protein heterodimerization activity, role in fatty acid metabolic process, protein import into peroxisome matrix, receptor recycling and cytosol, peroxisomal membrane localization
orf19.4292	orf19.4292	38.65	38.41	31.93	30.77	-0.191821593	0.998825	Ortholog(s) have SNAP receptor activity, role in Golgi to vacuole transport, vacuole inheritance and Golgi apparatus, endosome localization
orf19.3344	VPS17	51.18	38.4	51.75	37.22	-0.008813119	0.998825	Ortholog(s) have phosphatidylinositol-3-phosphate binding, protein transporter activity
orf19.239	orf19.239	42.38	38.38	44.38	40.26	0.03854541	0.998825	Putative ATP-dependent helicase; induced by nitric oxide
orf19.51	orf19.51	40.34	38.35	39.56	36.82	-0.02086339	0.998825	Ortholog(s) have role in mitochondrion organization, translational initiation and eukaryotic translation initiation factor 3 complex localization
orf19.6196	orf19.6196	30.47	38.33	42.68	34.81	0.113594246	0.998825	Component of the SWI/SNF and RSC chromatin remodeling complexes; suggested role in chromosome maintenance; Spider biofilm induced
orf19.7176	NPT1	39.91	38.27	49.97	47.44	0.205113361	0.998825	Putative nicotinate phosphoribosyltransferase, involved in NAD salvage pathway; fungal-specific (no human or murine homolog)

orf19.1249	orf19.1249	49.75	38.2	39.92	36.29	-0.147033401	0.998825	Ortholog(s) have 1-(5-phosphoribosyl)-5-[(5-phosphoribosylamino)methylideneamino]imidazole-4-carboxamide isomerase activity, role in histidine biosynthetic process and cytosol, nucleus localization
orf19.2564	orf19.2564	48.96	38.19	33.07	35.24	-0.23687154	0.957711	Putative nucleolar protein; implicated in ribosome biogenesis; rat catheter biofilm repressed
orf19.1606	orf19.1606	32.36	38.18	19.73	22.68	-0.41458803	0.710493	Protein of unknown function; Plc1-regulated
orf19.4186	PCT1	40.49	38.17	57.05	61.76	0.418464276	0.614782	Putative choline-phosphate cytidylyl transferase, antigenic during human oral infection
orf19.3322	DUT1	29.48	38.07	40.84	58.78	0.36710773	0.788835	dUTP pyrophosphatase; cell-cycle regulated if expressed in S. cerevisiae; upstream MluI and SCB elements; 17-beta-estradiol, ethynyl estradiol, macrophage induced; decreased in stationary phase yeast; rat catheter, Spider biofilm repressed
orf19.6855	orf19.6855	46.34	38.06	54.45	44.91	0.154101986	0.998825	Ortholog of C. dubliniensis CD36 : Cd36_04370, C. parapsilosis CDC317 : CPAR2_105410, Candida tenuis NRRL Y-1498 : CANTEDRAFT_114891 and Debaryomyces hansenii CBS767 : DEHA2D13420g
orf19.3874	orf19.3874	36.96	38.05	34.76	36.15	-0.059147041	0.998825	Predicted cation transmembrane transporter; Spider biofilm induced
orf19.4545	SWI4	39.86	38.04	37.89	35.24	-0.070822278	0.998825	Putative component of the SBF transcription complex involved in G1/S cell-cycle progression; periodic mRNA expression, peak at cell-cycle G1/S phase; predicted, conserved MBF binding sites upstream of G1/S-regulated genes
orf19.3836	orf19.3836	43.11	38.02	43.5	31.34	-0.094934233	0.998825	Ortholog(s) have cytoplasm localization

orf19.99	HAL21	43.95	37.97	68.9	63.25	0.482172146	0.463201	Putative phosphoadenosine-5'-phosphate or 3'-phosphoadenosine 5'-phosphosulfate phosphatase; possible role in sulfur recycling; ortholog of S. cerevisiae Met22; predicted Kex2 substrate; F-12/CO2 biofilm induced
orf19.3023	NGG1	37.68	37.96	46.6	39.32	0.108054396	0.998825	Ortholog(s) have histone acetyltransferase activity and role in cellular response to cadmium ion, detoxification of cadmium ion, histone acetylation, regulation of transcription from RNA polymerase II promoter
orf19.5752	orf19.5752	44.14	37.93	40.16	36.19	-0.080249746	0.998825	Ortholog(s) have ubiquitin-protein ligase activity, role in SCF-dependent proteasomal ubiquitin-dependent protein catabolic process and SCF ubiquitin ligase complex localization
orf19.1876	orf19.1876	39.44	37.91	32.89	29.59	-0.192336016	0.998825	Ortholog(s) have mRNA binding activity, role in mRNA splice site selection, positive regulation of mRNA splicing, via spliceosome and U1 snRNP, U2-type prespliceosome, commitment complex localization
orf19.5534	orf19.5534	43.44	37.88	41.18	39.92	-0.007512233	0.998825	Protein with a predicted role in mitotic spindle elongation, vesicle-mediated transport; flow model biofilm induced
orf19.6852	orf19.6852	40.4	37.86	45.79	54	0.23699169	0.951606	Ortholog of Rmd6 involved in S. cerevisiae sporulation; flow model biofilm induced
orf19.3244	orf19.3244	52.08	37.86	46.27	42	-0.028292167	0.998825	Ortholog(s) have role in mitochondrial respiratory chain complex assembly and mitochondrion localization
orf19.6393	orf19.6393	40.7	37.84	43.87	40.87	0.062063965	0.998825	Putative Arf3p GTPase activating protein; Hap43p-repressed gene; possibly an essential gene, disruptants not obtained by UAU1 method
orf19.434	PRD1	40.08	37.79	67.79	64.69	0.538869939	0.341413	Putative proteinase; transcript regulated by Nrg1, Mig1, and Tup1; Hogp-induced; stationary phase enriched protein; Hap43-repressed; rat catheter biofilm repressed

orf19.1516	orf19.1516	44.27	37.76	48.47	44.52	0.115678335	0.998825	Ortholog(s) have ubiquitin-specific protease activity
orf19.307	orf19.307	38.46	37.69	36.21	25.6	-0.194859542	0.998825	Ortholog(s) have transcription coactivator activity, role in histone acetylation, transcription from RNA polymerase II promoter and Ada2/Gcn5/Ada3 transcription activator complex, SAGA complex, SLIK (SAGA-like) complex localization
orf19.2715	RPC53	48.23	37.64	35.77	32.19	-0.227740828	0.974208	Ortholog(s) have RNA polymerase III activity, role in tRNA transcription from RNA polymerase III promoter and DNA-directed RNA polymerase III complex localization
orf19.2611	MCM6	37.17	37.64	62.94	58.95	0.480902739	0.481084	Putative MCM DNA replication initiation complex component; mRNA expression peak at cell-cycle M/G1 phase; regulated by tyrosol and cell density; repressed by alpha pheromone in SpiderM medium; Hap43-induced gene
orf19.2231	orf19.2231	45.94	37.62	27.05	25.82	-0.421694677	0.669402	Protein of unknown function; Spider biofilm induced
orf19.6208	MEF2	34.43	37.58	35.23	32.47	-0.075593651	0.998825	Putative mitochondrial translation elongation factor; caspofungin induced
orf19.4862	orf19.4862	51.2	37.58	70.13	51.69	0.311656753	0.856554	Putative integral membrane protein; S. cerevisiae ortholog Tvp23 localizes to late Golgi vesicles; rat catheter biofilm induced
orf19.1457	orf19.1457	38.12	37.58	36.84	32.1	-0.098995425	0.998825	Putative RNA polymerase transcription factor TFIIH core component; possibly an essential gene, disruptants not obtained by UAU1 method
orf19.7354	LAC1	38.46	37.57	43.44	45.02	0.132893242	0.998825	Ceramide synthase; required for biosynthesis of ceramides with C18:0 fatty acids, which serve as precursors for glucosylsphingolipids; caspofungin induced

orf19.5363	orf19.5363	32.34	37.57	32.83	31.95	-0.076671373	0.998825	Component of UDP-GlcNAc transferase; required for the 2nd step of dolichyl-linked oligosaccharide synthesis; Spider biofilm induced
orf19.3159	UTP20	36.94	37.54	33.79	33.07	-0.112470191	0.998825	Putative snoRNA-binding protein; S. cerevisiae Utp20 ortholog; likely essential for growth; repressed in core stress response; mutation confers resistance to 5-fluorocytosine (5-FC) and parnafungin
orf19.7511	orf19.7511	35.61	37.52	41.31	40.31	0.076275373	0.998825	Ortholog(s) have structural constituent of nuclear pore activity and role in cell division, nuclear pore organization, regulation of mitotic cell cycle
orf19.2883	CSO99	40.84	37.41	47.18	44.31	0.141542058	0.998825	Protein of unknown function; Hap43-repressed gene; protein not conserved in S. cerevisiae
orf19.4721	orf19.4721	45.52	37.41	38.16	38.7	-0.077522595	0.998825	Ortholog(s) have RNA binding activity, role in cytoplasmic translation, mRNA processing and mitochondrion localization
orf19.2215	GLE1	46.85	37.41	41.65	36.69	-0.066013669	0.998825	Putative nucleoporin; moderately induced at 42 degrees C
orf19.2639	orf19.2639	39.28	37.39	38.43	39.53	0.017109876	0.998825	Ortholog(s) have structural constituent of ribosome activity and mitochondrial large ribosomal subunit localization
orf19.6323	HPA2	35.67	37.38	38.15	41.56	0.078350629	0.998825	Ortholog(s) have D-amino-acid N-acetyltransferase activity, role in D-amino acid metabolic process and cytosol, nucleus localization
orf19.1715	IRO1	39.26	37.38	24.59	19.52	-0.459750673	0.599768	Putative transcription factor; role in iron utilization, pathogenesis; both IRO1 and adjacent URA3 are mutated in strain CAI4; suppresses S. cerevisiae aft1 mutant low-iron growth defect; hyphal-induced; reports differ about iron regulation

orf19.4212	FET99	39.22	37.36	15.63	16.88	-0.686464056	0.203072	Multicopper oxidase family protein; similar to S. cerevisiae Fet3; does not complement S. cerevisiae fet3 mutant growth under low-iron; iron-repressed; regulated by Tup1, Rim101; flow model biofilm induced; Spider biofilm repressed
orf19.1364	orf19.1364	49.17	37.36	52.3	42.14	0.079432136	0.998825	Ortholog of S. pombe Stm1 G-protein coupled receptor; PQ-loop domains; constitutive expression independent of MTL or white-opaque status; Hap43-repressed
orf19.2265	orf19.2265	45.52	37.3	41.83	38.12	-0.042536379	0.998825	Ortholog(s) have role in positive regulation of transcription from RNA polymerase II promoter in response to amino acid starvation and SWI/SNF complex, cytosol localization
orf19.5194.1	orf19.5194.1	36.31	37.3	49.45	49.43	0.277527842	0.904026	Putative protein of unknown function; clade-associated gene expression
orf19.586	ERV46	45.11	37.28	62.39	49.32	0.293867078	0.878136	Putative ER-derived vesicle protein; COPII-coated vesicle complex subunit; transcript induced by filamentous growth; Spider biofilm repressed
orf19.4159	orf19.4159	37.56	37.2	34.56	39.82	-0.006846808	0.998825	Has domain(s) with predicted role in transmembrane transport
orf19.5767	orf19.5767	31.41	37.19	20.22	30.2	-0.264226035	0.938642	Ortholog(s) have RNA binding activity and role in mRNA splicing, via spliceosome
orf19.3438	orf19.3438	45.17	37.19	57.85	57.49	0.334595501	0.821978	Ortholog(s) have chaperone binding activity, role in ER-associated ubiquitin-dependent protein catabolic process, protein folding in endoplasmic reticulum, response to unfolded protein and endoplasmic reticulum lumen localization
orf19.6779	PRO2	45.97	37.18	51.41	42.91	0.113934842	0.998825	Putative gamma-glutamyl phosphate reductase with a predicted role in proline biosynthesis; regulated by Gcn2p and Gcn4p

orf19.2956	MGM101	50.73	37.17	53.11	45.99	0.109995613	0.998825	Putative mitochondrial genome maintenance protein; fungal-specific (no human or murine homolog); mutation confers hypersensitivity to tubercidin (7-deazaadenosine)
orf19.6133	PIF1	36.77	37.16	36.6	34.96	-0.03424067	0.998825	Putative DNA helicase; decreased transcription is observed upon fluphenazine treatment
orf19.5678	orf19.5678	44.21	37.14	40.67	37.62	-0.031321495	0.998825	Has domain(s) with predicted role in peptidyl-diphthamide biosynthetic process from peptidyl-histidine and cytoplasm localization
orf19.270	orf19.270	33.23	37.13	21.45	24.03	-0.38022832	0.788414	Ortholog of C. parapsilosis CDC317 : CPAR2_102150, C. dubliniensis CD36 : Cd36_82780, Pichia stipitis Pignal : psti_CGOB_00155 and Candida orthopsilosis Co 90-125 : CORT_0B03450
orf19.5204	orf19.5204	29.28	37.1	26.82	23.98	-0.229799991	0.986722	Ortholog(s) have role in cellular response to methylmercury and cytoplasm, nucleus localization
orf19.1630	orf19.1630	40.37	37.04	44.38	38.31	0.050905512	0.998825	Ortholog(s) have cytoplasm localization
orf19.5734	POP2	38.05	37.03	38.45	31.65	-0.073375974	0.998825	Component of the Ccr4-Pop2 mRNA deadenylase; heterozygous null mutant exhibits resistance to parnafungin and cordycepin in the C. albicans fitness test
orf19.2149	orf19.2149	48.42	37.03	53.23	42.84	0.111675442	0.998825	Putative sulfate permease; S. cerevisiae ortholog YPR003C localizes to the endoplasmic reticulum; regulated by Sef1p-, Sfu1p-, and Hap43p
orf19.5164	ECM39	41.35	37	38.6	42.8	0.041168242	0.998825	Putative mannosyltransferase similar to S. cerevisiae Ecm39p, which has a role in Calcofluor white resistance; predicted Kex2p substrate; has HKEXRF motif

orf19.3774	PPG1	29.03	36.95	32.68	27.59	-0.080587081	0.998825	Putative protein phosphatase of the Type 2A-related family (serine/threonine-specific), similar to S. cerevisiae Ppg1p; possibly an essential gene, disruptants not obtained by UAU1 method
orf19.5757	orf19.5757	46.1	36.94	42.41	36.25	-0.066989401	0.998825	Ortholog(s) have cytoplasm, nucleus localization
orf19.5321	orf19.5321	44.61	36.89	46.79	41.78	0.071997902	0.998825	Ortholog(s) have methylenetetrahydrofolate reductase (NAD(P)H) activity and role in methionine biosynthetic process, one-carbon metabolic process
orf19.3035	orf19.3035	38.81	36.89	40.44	36.62	0.003729074	0.998825	Ortholog(s) have ATP-dependent DNA helicase activity, chromatin DNA binding, methylated histone residue binding, rDNA binding activity
orf19.3695	orf19.3695	46.5	36.87	45.75	45.27	0.071900445	0.998825	Ortholog(s) have diacylglycerol cholinephosphotransferase activity, ethanolaminephosphotransferase activity, role in phosphatidylcholine biosynthetic process, phosphatidylethanolamine biosynthetic process and Golgi apparatus localization
orf19.3135	orf19.3135	31.97	36.85	41.64	42.24	0.174752941	0.998825	Ortholog(s) have protein binding, bridging activity and role in ER-associated ubiquitin-dependent protein catabolic process, lipid particle organization
orf19.8278	orf19.8278	39.21	36.82	19.55	26.06	-0.436468266	0.647046	Predicted ORF from original SGTC Assembly 19 annotation, removed from the reduced ORF set by the SGTC; subsequently reinstated in Assembly 21 based on comparative genome analysis
orf19.2447	orf19.2447	41.49	36.79	31.94	30.78	-0.203280669	0.998825	Putative Type II phosphatidylinositol 4-kinase; Ssr1-repressed; flow model biofilm repressed
orf19.3071	MIH1	25.26	36.77	12.32	16.01	-0.591306245	NA	Putative protein phosphatase of the PTP family (tyrosine-specific); ortholog of S. cerevisiae Mih1; mRNA binds She3

orf19.2302	orf19.2302	38.8	36.75	42.61	38.67	0.063500098	0.998825	Ortholog(s) have enzyme regulator activity, role in cellular calcium ion homeostasis and integral to endoplasmic reticulum membrane localization
orf19.5747	orf19.5747	41.44	36.7	41.59	36.86	0.00349758	0.998825	Ortholog(s) have structural constituent of ribosome activity and mitochondrial small ribosomal subunit localization
orf19.4450	ZCF23	40.29	36.68	37.46	41.47	0.005503771	0.998825	Predicted Zn(II)2Cys6 transcription factor; ortholog of S. cerevisiae Gsm1; flow model biofilm induced
orf19.185	orf19.185	42.27	36.65	42.49	39.93	0.027998971	0.998825	Ortholog(s) have structural constituent of ribosome activity, role in mitochondrial translational initiation and mitochondrial small ribosomal subunit localization
orf19.1298	NUP84	40.81	36.65	35.12	35.03	-0.10819896	0.998825	Ortholog(s) have structural constituent of nuclear pore activity
orf19.548	CDC10	36.43	36.59	57.29	50.81	0.376389441	0.734743	Septin, required for wild-type cell, hyphal, or chlamydospore morphology; role in virulence and kidney tissue invasion in mouse infection; forms ring at sites of cell division and filaments in mature chlamydospore; Spider biofilm repressed
orf19.2372	orf19.2372	32.78	36.58	33.5	37.24	-0.008235361	0.998825	Pol protein of retrotransposon Tca2; separated by a stop codon from Gag protein orf19.2371; likely translated as single polyprotein with Gag, reverse transcriptase, protease, and integrase; rat catheter biofilm repressed
orf19.6459	DPP3	42.32	36.55	67.81	54.27	0.429376315	0.594776	Protein similar to S. cerevisiae pyrophosphate phosphatase Dpp1; required for farnesol biosynthesis; repressed by 17-beta-estradiol, ethynyl estradiol; Spider biofilm induced
orf19.4323	orf19.4323	32.63	36.5	20.92	22.85	-0.398329351	0.742982	Ortholog(s) have phosphatidylinositol-3-phosphate binding activity and endosome localization

orf19.5815	SCT2	38.32	36.5	53.86	53.23	0.343124196	0.811201	Putative glycerol-3-phosphate acyltransferase; fungal-specific (no human or murine homolog)
orf19.1666	orf19.1666	37.41	36.46	42.42	41.82	0.1251562	0.998825	Ortholog of Dig2, a MAP kinase-responsive inhibitor of Ste12; regulates mating-specific genes and invasive growth in S. cerevisiae; induced by alpha pheromone in SpiderM medium
orf19.4606	ERG8	41.74	36.41	48.09	47.93	0.196037391	0.998825	Putative phosphomevalonate kinase; enzyme of the mevalonate pathway of isoprenoid biosynthesis; transcript regulated by Nrg1; possible drug target; not conserved in H. sapiens
orf19.5830	LHS1	38.76	36.37	62.93	61.13	0.500191526	0.432617	Protein similar to S. cerevisiae Hsp70p; predicted Kex2p substrate; possibly essential, disruptants not obtained by UAU1 method; flow model biofilm repressed
orf19.5735	CDC50	45.02	36.35	45.21	38.44	0.015544242	0.998825	Putative endosomal protein; induced by Mnl1p under weak acid stress
orf19.4465	orf19.4465	39.44	36.34	31.3	30.56	-0.180224818	0.998825	Protein of unknown function; Spider biofilm induced
orf19.7347	orf19.7347	36.31	36.3	25.27	27.86	-0.275212604	0.927778	Ortholog(s) have ubiquitin-protein ligase activity, role in ER-associated ubiquitin-dependent protein catabolic process and endoplasmic reticulum membrane localization
orf19.839	orf19.839	28.92	36.29	29.34	29.91	-0.09387941	0.998825	Putative endoribonuclease; induced by nitric oxide independent of Yhb1p; mutation confers hypersensitivity to aureobasidin A
orf19.7527	orf19.7527	38.73	36.26	39.14	29.66	-0.086490736	0.998825	Ortholog of C. dubliniensis CD36 : Cd36_25130, C. parapsilosis CDC317 : CPAR2_800100, Candida tenuis NRRL Y-1498 : CANTEDRAFT_107256 and Debaryomyces hansenii CBS767 : DEHA2E18370g

orf19.5206	orf19.5206	40.16	36.25	38.59	32.87	-0.059128618	0.998825	Putative chaperone protein; role in the assembly of box H/ACA snoRNPs and thus for pre-rRNA processing; Spider biofilm induced
orf19.5148	CYR1	39.38	36.22	46.69	46.48	0.200505476	0.998825	Class III adenylyl cyclase; mutant lacks cAMP; mutant hyphal growth defect rescued by exogenous cAMP; required for mouse mucosal or systemic infection; role in macrophage sensitivity, apoptosis; downstream of Ras1p and CO2 signaling
orf19.3303	orf19.3303	33.34	36.19	30.04	33.68	-0.075136198	0.998825	Ortholog(s) have tRNA methyltransferase activity, role in tRNA methylation, wybutosine biosynthetic process and cytosol, mitochondrion, nucleus localization
orf19.5884	orf19.5884	41.86	36.16	36.57	32.57	-0.108593315	0.998825	Putative nucleolar preribosomal-associated protein; decreased transcription is observed upon benomyl treatment or in an azole-resistant strain that overexpresses MDR1; Spider biofilm induced
orf19.592	orf19.592	47.66	36.13	38.25	36.5	-0.113235624	0.998825	Ortholog of S. cerevisiae YNL092W and S. pombe SPBC1778.07; a putative N2227-like methyltransferase; Hap43-repressed gene
orf19.7360	orf19.7360	48.37	36.13	36.6	38.24	-0.112678931	0.998825	Ortholog(s) have enzyme activator activity, role in histone deubiquitination, regulation of transcription from RNA polymerase II promoter and DUBm complex, SAGA complex localization
orf19.5984	orf19.5984	43.66	36.12	39.04	34.14	-0.09367533	0.998825	Putative protein of unknown function; homozygous transposon insertion causes decreased colony wrinkling under filamentous growth-inducing conditions, but does not block true hyphal formation in liquid media
orf19.5102	PLB5	35.48	36.12	43.03	33.5	0.052046379	0.998825	Putative GPI-linked phospholipase B, fungal-specific (no mammalian homolog); null mutation eliminates cell-associated phospholipase A2 activity and attenuates virulence; fluconazole-repressed; flow model biofilm repressed
orf19.204	orf19.204	37.05	36.1	37.58	35.68	0.004137347	0.998825	Ortholog of C. dubliniensis CD36 : Cd36_23180, Candida tropicalis MYA-3404 : CTRG_01968 and Candida albicans WO-1 : CAWG_05983

orf19.6382	orf19.6382	37.66	36.09	37.6	35.21	-0.021186286	0.998825	Putative MRP/CFTR-subfamily ABC transporter; member of multidrug resistance-associated protein (MRP) subfamily of ABC family; similar to S. cerevisiae Bpt1p
orf19.993	orf19.993	32.35	36.05	40.42	51.51	0.277210484	0.914829	Protein of unknown function; rat catheter biofilm repressed
orf19.4406	NIF3	32.67	36.03	45.83	45.14	0.254046685	0.938642	Protein of unknown function; ortholog of S. cerevisiae Nif3; rat catheter biofilm repressed
orf19.485	orf19.485	35.72	36.03	41.99	44.51	0.172310169	0.998825	Ortholog(s) have structural constituent of cytoskeleton activity
orf19.3235	orf19.3235	44.61	36.03	43.36	42.44	0.039956519	0.998825	Putative F-actin capping protein subunit alpha; possibly an essential gene, disruptants not obtained by UAU1 method
orf19.7114	CSA1	34.84	36.03	42.91	41.45	0.149848566	0.998825	Surface antigen on elongating hyphae and buds; strain variation in repeat number; ciclopirox, filament induced, alkaline induced by Rim101; Efg1-, Cph1, Hap43-regulated; required for WT RPMI biofilm formation; Bcr1-induced in a/a biofilms
orf19.6833.2	orf19.6833.2	42.58	36.02	50.64	45.17	0.182746169	0.998825	Ortholog of Candida albicans WO-1 : CAWG_00951
orf19.1526	SNF2	37.25	36.02	38.79	34.48	-0.009255738	0.998825	Protein involved in transcriptional regulation; ortholog of S. cerevisiae Snf2p, which is the catalytic subunit of the SWI/SNF chromatin remodeling complex; interacts with Swi1p; SWI/SNF complex is essential for hyphal growth and virulence
orf19.4825	orf19.4825	34.33	36.01	42.26	35.91	0.090417986	0.998825	Mitochondrial matrix protein; required for assembly/stability of the F1 sector of mitochondrial F1F0 ATP synthase at high temperature; rat catheter biofilm repressed

orf19.332	orf19.332	38.91	36	35.39	32.66	-0.098348525	0.998825	Member of a complex that contains Prp19; stabilizes U6 snRNA in catalytic forms of the spliceosome containing U2, U5, and U6 snRNAs; Spider biofilm induced
orf19.26	orf19.26	41.51	35.97	35.73	34.61	-0.094862584	0.998825	Putative peptide N-glycanase; induced by nitric oxide independent of Yhb1p
orf19.674	orf19.674	41.84	35.97	42.58	36.21	0.003537984	0.998825	Ortholog(s) have U6 snRNA binding, pre-mRNA binding activity and role in mRNA cis splicing, via spliceosome, spliceosomal snRNP assembly
orf19.4617	orf19.4617	38.47	35.91	27.14	33.64	-0.178859049	0.998825	Predicted peptide alpha-N-acetyltransferase; flow model biofilm induced
orf19.6455	orf19.6455	38.2	35.89	39.24	33.5	-0.034668398	0.998825	Protein of unknown function, transcript is upregulated in clinical isolates from HIV+ patients with oral candidiasis
orf19.105	HAL22	40.16	35.88	47.31	45.17	0.177258808	0.998825	Putative phosphoadenosine-5'-phosphate or 3'-phosphoadenosine 5'-phosphosulfate phosphatase; possible role in sulfur recycling; Hap43-repressed; F-12/CO2 biofilm induced
orf19.7572	SPT7	36.8	35.85	32.08	29.23	-0.170524931	0.998825	Putative SAGA transcriptional regulatory complex subunit; mutation confers hypersensitivity to toxic ergosterol analog, and to amphotericin B
orf19.155	URE2	43.31	35.84	44.47	39.37	0.039456432	0.998825	Functional homolog of S. cerevisiae Ure2p, which is a regulator of nitrogen utilization, and which also has an infectious prion form called [URE3]; forms [URE3] prion when expressed in S. cerevisiae
orf19.6642	orf19.6642	45.71	35.84	46.43	41.81	0.061520545	0.998825	Ortholog(s) have role in ER to Golgi vesicle-mediated transport, cellular response to nitrogen starvation, negative regulation of G0 to G1 transition

orf19.759	SEC21	38.98	35.82	56	53.16	0.362676131	0.767642	Ortholog(s) have role in ER to Golgi vesicle-mediated transport, retrograde vesicle-mediated transport, Golgi to ER and COPI vesicle coat, cytosol, endosome localization
orf19.1478	STT3	38.57	35.77	71.79	64.33	0.600135949	0.237436	Putative oligosaccharyltransferase complex component; flow model and rat catheter biofilm repressed
orf19.4036	APM1	40.87	35.75	53.5	37.77	0.150016573	0.998825	Ortholog of S. cerevisiae/S. pombe Apm1; a clathrin-associated protein complex (AP-1) subunit; phosphorylated protein; Tn mutation affects filamentous growth; Spider biofilm repressed
orf19.5166	DBF4	36.61	35.74	34.8	34.17	-0.060079208	0.998825	Putative Cdc7p-Dbf4p kinase complex regulatory subunit; Hap43p-induced gene; macrophage/pseudohyphal-repressed; cell-cycle regulated periodic mRNA expression; S. cerevisiae ortholog is not cell-cycle regulated
orf19.2612	orf19.2612	46.15	35.74	40.46	38.17	-0.054098843	0.998825	C2H2 zinc finger transcription factor; expression reduced in ssr1 null mutant; flow model biofilm induced
orf19.3536	orf19.3536	44.21	35.73	41.95	42.06	0.040389322	0.998825	Ortholog(s) have acetylglucosaminyltransferase activity, role in protein N-linked glycosylation and Golgi medial cisterna localization
orf19.4705	orf19.4705	35.31	35.65	34.52	34.62	-0.021488664	0.998825	Ortholog(s) have tRNA adenylyltransferase activity, role in tRNA 3'-terminal CCA addition and cytosol, mitochondrial matrix localization
orf19.5677	DUR4	41.75	35.63	36.96	34.23	-0.095361541	0.998825	Putative urea permease; fungal-specific (no human or murine homolog); possibly an essential gene, disruptants not obtained by UAU1 method
orf19.3064.1	orf19.3064.1	34.76	35.63	40	23.1	-0.118010317	0.998825	Ortholog of C. parapsilosis CDC317 : CPAR2_108915, Candida tenuis NRRL Y-1498 : CANTEDRAFT_102387, Debaryomyces hansenii CBS767 : DEHA2B06776g and Pichia stipitis Pignal : psti_CGOB_00149

orf19.5353	orf19.5353	38.34	35.61	35.14	34.16	-0.072508512	0.998825	Ortholog of C. parapsilosis CDC317 : CPAR2_407790, C. dubliniensis CD36 : Cd36_24630, Candida orthopsilosis Co 90-125 : CORT_0C07130 and Spathaspora passalidarum NRRL Y-27907 : SPAPADRAFT_149801
orf19.5730	orf19.5730	35.49	35.6	36.6	35.5	0.003885302	0.998825	Putative phenylacrylic acid decarboxylase; clade-associated gene expression
orf19.3984	orf19.3984	33.47	35.57	42.84	44.13	0.210012303	0.998825	Protein of unknown function; induced in core caspofungin response; induced in ssr1 mutant; induced by nitric oxide independent of Yhb1; Spider biofilm induced
orf19.6419.1	orf19.6419.1	41.01	35.49	36.63	35.39	-0.057636823	0.998825	Ortholog of C. parapsilosis CDC317 : CPAR2_205710, Candida tenuis NRRL Y-1498 : CANTEDRAFT_117073, Lodderomyces elongisporus NRLL YB-4239 : LELG_02945 and Debaryomyces hansenii CBS767 : DEHA2A11462g
orf19.4448	SOG2	38.01	35.45	34.25	30.58	-0.11390566	0.998825	Leucine-rich-repeat domain protein of RAM cell wall integrity signaling network; role in cell separation, azole sensitivity; required for hyphal growth; lacks orthologs in higher eukaryotes
orf19.5358	orf19.5358	44.58	35.43	38.3	36.5	-0.066961562	0.998825	Phosphorylated protein of unknown function
orf19.3228	orf19.3228	38.97	35.42	31.9	31.21	-0.153540577	0.998825	Putative endosomal transmembrane protein; Hap43p-induced; mutation confers hypersensitivity to amphotericin B
orf19.7078	orf19.7078	39.69	35.41	27.24	35.73	-0.161232634	0.998825	Ortholog(s) have role in meiosis and cytoplasm localization
orf19.6740	orf19.6740	45.69	35.37	40.29	39.02	-0.029714523	0.998825	Ortholog(s) have ubiquitin-protein ligase activity and role in generation of catalytic spliceosome for first transesterification step

orf19.3839	SAP10	42.78	35.36	20.65	18.04	-0.590663731	0.340941	Secreted aspartyl protease; roles in adhesion, virulence (RHE model), cell surface integrity; distinct specificity from Sap9; at cell membrane and wall; GPI-anchored; induced in low iron; Tbf1-activated; Spider biofilm induced
orf19.864	orf19.864	35.25	35.35	38.99	34.72	0.042122202	0.998825	Ortholog(s) have role in nuclear-transcribed mRNA catabolic process, 3'-5' exonucleolytic nonsense-mediated decay and cytosol, polysome localization
orf19.3176	RIM21	31.12	35.32	28.59	26.36	-0.167731482	0.998825	Plasma membrane pH-sensor involved in the Rim101 pH response pathway; required for processing and activation of Rim101 and for alkaline pH-induced hyphal growth; Spider biofilm induced
orf19.2037	orf19.2037	39.54	35.23	30.86	27.42	-0.238481765	0.957711	Ortholog of C. dubliniensis CD36 : Cd36_15810, C. parapsilosis CDC317 : CPAR2_212950, Candida tenuis NRRL Y-1498 : CANTEDRAFT_116162 and Debaryomyces hansenii CBS767 : DEHA2D18392g
orf19.6351	orf19.6351	42.93	35.21	39.63	32.06	-0.082843668	0.998825	Ortholog of C. dubliniensis CD36 : Cd36_11880, Candida tenuis NRRL Y-1498 : cten_CGOB_00231, Debaryomyces hansenii CBS767 : DEHA2D04928g and Pichia stipitis Pignal : PICST_29234
orf19.1401	EAP1	33.98	35.2	36.15	34.63	0.017444209	0.998825	GPI-anchored cell wall adhesin; Efg1-regulated; mutant suppresses cell adhesion, filamentation, invasive growth defects of S. cerevisiae flo8 or flo11 mutant; biofilm induced; required for biofilm formation; Bcr1-repressed in a/a biofilms
orf19.1027	PDR16	41.47	35.2	8.44	4.39	-1.226830902	NA	Phosphatidylinositol transfer protein; induction correlates with CDR1, CDR2 overexpression/azole resistance; fluphenazine, 17-beta-estradiol, ethynyl estradiol, NO induced; farnesol-downregulated in biofilm; rat catheter biofilm induced
orf19.1890	orf19.1890	39.13	35.18	39.53	37.36	0.02929501	0.998825	Ortholog(s) have role in medium-chain fatty acid biosynthetic process
orf19.6685	ISY1	32.56	35.16	22.99	26.3	-0.286757943	0.914958	Putative pre-mRNA-splicing factor; mutation confers resistance to 5-fluorocytosine (5-FC); rat catheter biofilm induced

orf19.3678	orf19.3678	35.06	35.16	30.44	29.99	-0.145480879	0.998825	Ortholog of C. dubliniensis CD36 : Cd36_02060, C. parapsilosis CDC317 : CPAR2_106200, Candida tenuis NRRL Y-1498 : CANTEDRAFT_96322 and Debaryomyces hansenii CBS767 : DEHA2C02310g
orf19.3622	ANP1	30.08	35.16	45.8	36.51	0.214248838	0.998825	Putative mannosyltransferase of Golgi; member of Mnn9p family; similar to S. cerevisiae Anp1p; fungal-specific (no human or murine homolog)
orf19.3734	GEF2	33.8	35.15	24.87	24.93	-0.285332443	0.916335	Member of the voltage chloride channel family; Hap43p-repressed gene
orf19.5413	orf19.5413	38.96	35.1	32.94	26.85	-0.197250993	0.998825	Ortholog(s) have role in mRNA splicing, via spliceosome and Prp19 complex, spliceosomal complex localization
orf19.4788	ARG5,6	34.17	35.09	47.44	43.91	0.254540894	0.938642	Arginine biosynthetic enzyme; processed in S. cerevisiae into 2 polypeptides with acetylglutamate kinase (Arg6) activity and acetylglutamate-phosphate reductase (Arg5) activity; Gcn4 regulated; alkaline repressed; Spider biofilm induced
orf19.1083	orf19.1083	33.11	35.06	41.97	41.95	0.189381301	0.998825	Putative protein of unknown function; macrophage-induced gene
orf19.2331	ADA2	28.76	35.04	34.26	31.46	0.003230444	0.998825	Zinc finger and homeodomain transcriptional coactivator; role in cell wall integrity and in sensitivity to caspofungin; required for the normal transcriptional response to caspofungin; required for yeast cell adherence to silicone substrate
orf19.6396	orf19.6396	35.96	35.03	50.07	52.76	0.355204337	0.789669	Putative patatin-like phospholipase; similar to S. cerevisiae Nte1p, which is predicted to be a membrane protein; antigenic during human oral infection; Hap43p-repressed gene
orf19.7106	VPS70	33.91	35.02	39.17	39.39	0.10541697	0.998825	Ortholog(s) have role in protein targeting to vacuole and endoplasmic reticulum localization

orf19.5799	orf19.5799	51.26	35	49.16	43.4	0.059746643	0.998825	Ortholog(s) have cytoplasm localization
orf19.2736	HFL2	48.93	35	38.05	43.4	-0.038548513	0.998825	HAP5-like; ortholog of S. cerevisiae Bur6, a heterodimeric NC2 transcription regulator complex subunit; flucytosine induced
orf19.4242	CST20	37.2	34.94	45.39	39.46	0.13680692	0.998825	Protein kinase of Ste20p/p65PAK family, required for wild-type mating efficiency and virulence in a mouse model; Cst20p-Hst7p-Cek1p-Cph1p MAPK pathway regulates some hyphal growth; involved in Cdc42p growth regulation
orf19.2200	orf19.2200	36.93	34.92	40.96	37.52	0.078589964	0.998825	Protein of unknown function; rat catheter biofilm repressed
orf19.296	orf19.296	27.2	34.91	36.63	13.87	-0.169549878	0.998825	Ortholog of C. dubliniensis CD36 : Cd36_82980, C. parapsilosis CDC317 : CPAR2_102360, Candida tenuis NRRL Y-1498 : cten_CGOB_00165 and Debaryomyces hansenii CBS767 : DEHA2G00990g
orf19.6792	RRD1	36.68	34.91	37.9	33.06	-0.021846033	0.998825	Putative peptidyl-prolyl cis/trans-isomerase; caspofungin induced
orf19.4181	SPC2	56	34.89	55.47	66.14	0.284837071	0.885512	Signal peptidase complex component; role in ER protein translocation; transcript is induced upon filamentous growth
orf19.3256	SLN1	38.62	34.87	33.46	30.34	-0.15406025	0.998825	Histidine kinase involved in a two-component signaling pathway that regulates cell wall biosynthesis; mutants are sensitive to growth on H2O2 medium; rat catheter and Spider biofilm induced
orf19.3030	orf19.3030	36.15	34.85	31.04	33.82	-0.086936405	0.998825	Ortholog(s) have phosphatidylinositol binding activity

orf19.6422	SSY5	37.14	34.85	30.37	29.32	-0.185850451	0.998825	Ortholog(s) have serine-type endopeptidase activity, role in protein processing, response to amino acid and extrinsic to plasma membrane localization
orf19.878	NBN1	35.26	34.83	29.69	28.84	-0.160324941	0.998825	Subunit of the NuA4 histone acetyltransferase complex; Plc1p-regulated
orf19.3193	FCR3	29.52	34.82	30.22	30.67	-0.064848822	0.998825	bZIP transcription factor; ortholog of S. cerevisiae Yap3; partially complements fluconazole sensitivity of S. cerevisiae pdr1 pdr3; Hap43-induced; required for yeast cell adherence to silicone substrate; flow model biofilm repressed
orf19.3613	orf19.3613	45.85	34.81	41.62	37.36	-0.030640414	0.998825	Ortholog(s) have RNA polymerase II core binding, RNA polymerase II core promoter sequence-specific DNA binding transcription factor activity, TFIIF-class binding transcription factor activity, chromatin binding activity
orf19.5068	IRE1	35.92	34.81	34.78	29.18	-0.103138589	0.998825	Putative protein kinase; role in cell wall regulation; mutant is hypersensitive to caspofungin; Spider biofilm induced
orf19.814	SSY1	37.52	34.79	29.7	21.9	-0.305331186	0.883756	Amino acid sensor; required for wild-type hyphal growth on solid serum or Lees media, not under all conditions; 12 predicted membrane spanning regions; Hap43-repressed; Spider biofilm induced
orf19.7300	orf19.7300	31.8	34.78	27.12	20.9	-0.292431192	0.906554	Protein of unknown function; Spider biofilm induced
orf19.4205.1	orf19.4205.1	44.44	34.73	29.82	45.11	-0.050105025	0.998825	Ortholog(s) have role in mRNA splicing, via spliceosome and U1 snRNP, U2 snRNP, U2-type prespliceosome, U4/U6 x U5 tri-snRNP complex, U5 snRNP, cytosol localization
orf19.1995	MNN24	43.15	34.72	36.71	34.36	-0.094706601	0.998825	Alpha-1,2-mannosyltransferase; required for normal cell wall mannan content

orf19.325	orf19.325	44.4	34.7	37.18	34.01	-0.106482562	0.998825	Putative mRNA cleavage and polyadenlylation factor; heterozygous null mutant exhibits hypersensitivity to parnafungin and cordycepin in the C. albicans fitness test
orf19.4595	orf19.4595	37.56	34.7	36.04	33.61	-0.046659039	0.998825	Ortholog of C. dubliniensis CD36 : Cd36_41860, C. parapsilosis CDC317 : CPAR2_400440, Candida tenuis NRRL Y-1498 : CANTEDRAFT_103033 and Debaryomyces hansenii CBS767 : DEHA2B03234g
orf19.4812	orf19.4812	26.39	34.69	49.72	47.26	0.422517492	0.648522	Phosphopantetheine:protein transferase (PPTase); Spider biofilm induced
orf19.6020	orf19.6020	38.02	34.62	37.73	36.4	0.004678526	0.998825	Ortholog(s) have Atg8 ligase activity and role in C-terminal protein lipidation, CVT pathway, autophagic vacuole assembly, cellular response to nitrogen starvation, mitochondrion degradation, piecemeal microautophagy of nucleus
orf19.7379	FAA2	37.27	34.57	31.48	31.79	-0.127966079	0.998825	Putative acyl CoA synthetase; expression regulated upon white-opaque switch; rat catheter biofilm induced; Spider biofilm induced
orf19.796	HYM1	37.55	34.56	35.6	27.05	-0.142545191	0.998825	Protein of RAM cell wall integrity signaling network; involved in regulation of Ace2 activity and cellular morphogenesis; role in cell separation, azole sensitivity; required for hyphal growth
orf19.4874	MNN23	39.21	34.51	34.43	40.51	0.006963047	0.998825	Alpha-1,2-mannosyltransferase; mutants hypersensitive to toxic ergosterol analog, amphotericin B, calcofluor white, growth at 42 degrees and have decreased cell wall mannan; flow model biofilm induced
orf19.2485	orf19.2485	37.6	34.51	40.96	34.88	0.028647073	0.998825	Ortholog(s) have structural constituent of nuclear pore activity and role in chromosome segregation, mitotic spindle assembly checkpoint, nuclear pore complex assembly, protein targeting to nuclear inner membrane
orf19.5675	orf19.5675	35.44	34.48	27.75	27.3	-0.206653255	0.998825	Protein involved in proteolytic control of sumoylated substrates; interacts with SUMO; member of the SWI/SNF family of DNA-dependent ATPases; Spider biofilm induced

orf19.5180	PRX1	33.7	34.47	43.77	52.34	0.322375355	0.858075	Thioredoxin peroxidase; transcriptionally induced by interaction with macrophage; fluconazole induced; Fkh2p-downregulated; caspofungin repressed; protein present in exponential and stationary growth phase yeast cultures
orf19.3806	orf19.3806	41.44	34.46	38.1	34.36	-0.04535968	0.998825	Ortholog(s) have role in negative regulation of gluconeogenesis, proteasome-mediated ubiquitin-dependent protein catabolic process, traversing start control point of mitotic cell cycle and GID complex, cytoplasm, nucleus localization
orf19.7110	orf19.7110	40.58	34.44	41.19	40.09	0.066083608	0.998825	Ortholog(s) have tRNA-specific adenosine-37 deaminase activity, role in tRNA modification and cytosol, nucleus localization
orf19.2668	RHD2	35.66	34.41	38.65	35.34	0.042692187	0.998825	Predicted ORF in retrotransposon Tca4 with similarity to the Gag region encoding nucleocapsid-like protein; overlaps blocked reading frame orf19.2668.1; yeast-enriched transcript; rat catheter biofilm induced
orf19.4639	orf19.4639	37.84	34.41	45.68	41.12	0.17185005	0.998825	Protein present in exponential and stationary growth phase yeast cultures
orf19.2691	orf19.2691	29.37	34.39	43.55	42.47	0.278718823	0.914829	Planktonic growth-induced gene
orf19.3173	orf19.3173	39.18	34.38	37.39	26.69	-0.128127162	0.998825	Ortholog(s) have Golgi apparatus, endoplasmic reticulum localization
orf19.25	orf19.25	34.77	34.37	30.22	28.27	-0.161872576	0.998825	Ortholog(s) have tRNA (guanine) methyltransferase activity, role in tRNA methylation and cytoplasm, nucleolus localization
orf19.4442	ALG9	36.32	34.32	40.18	38.07	0.092711926	0.998825	Putative mannosyltransferase; similar to S. cerevisiae Alg9p; has HKEXRF motif

orf19.1673	PPT1	34.41	34.29	28.96	30.81	-0.118311414	0.998825	Putative serine/threonine phosphatase; induced in high iron
orf19.1927	SNM1	45.05	34.25	29.74	27.83	-0.284474061	0.90951	Protein similar to RNase MRP RNA binding protein; ciclopirox olamine induced; regulated by Sef1, Sfu1, and Hap43; Spider biofilm induced
orf19.6885	SPO7	37.47	34.2	45.06	33.15	0.076963375	0.998825	Putative regulatory subunit of Nem1-Spo7 phosphatase holoenzyme that regulates nuclear growth by controling phospholipid biosynthesis; induced by alpha pheromone in SpiderM medium
orf19.3351	orf19.3351	45.02	34.18	33.89	35.81	-0.117266981	0.998825	Protein of unknown function; Hap43-induced; Spider biofilm induced
orf19.6462	orf19.6462	39.03	34.17	46.9	44.46	0.204144334	0.998825	Ortholog(s) have role in protein folding in endoplasmic reticulum and ER membrane protein complex, cytosol localization
orf19.1160	orf19.1160	37.43	34.15	41.18	35.83	0.066948343	0.998825	Ortholog(s) have COPII adaptor activity and role in ER to Golgi vesicle-mediated transport, fungal-type cell wall organization, protein glycosylation, protein retention in Golgi apparatus
orf19.107	orf19.107	43.53	34.13	30.59	28.29	-0.258454141	0.938642	DEAH-box ATP-dependent RNA helicase, required for 18S rRNA synthesis; rat catheter biofilm induced
orf19.1913	orf19.1913	38.76	34.13	43.26	36.8	0.077758944	0.998825	Protein of unknown function; flow model biofilm induced
orf19.6425	SKI2	36.24	34.12	44.13	45.33	0.221032383	0.983377	Ortholog(s) have role in cellular response to cadmium ion, detoxification of cadmium ion, nuclear-transcribed mRNA catabolic process, 3'-5' exonucleolytic nonsense-mediated decay, nuclear-transcribed mRNA catabolic process, non-stop decay

orf19.2850	orf19.2850	30.86	34.12	23.61	21.03	-0.315618441	0.878136	Protein of unknown function; induced by nitric oxide independent of Yhb1p
orf19.3980	orf19.3980	36.87	34.11	40.11	38.74	0.0922603	0.998825	Ortholog(s) have role in cytoplasmic translation, regulation of translation and cytosol, polysomal ribosome localization
orf19.5019	orf19.5019	33.89	34.06	31.24	29.01	-0.119586529	0.998825	Ortholog of C. dubliniensis CD36 : Cd36_12800, C. parapsilosis CDC317 : CPAR2_203460, Candida tenuis NRRL Y-1498 : CANTEDRAFT_94106 and Debaryomyces hansenii CBS767 : DEHA2C13948g
orf19.229	orf19.229	33.14	34.02	40.04	33.63	0.081101233	0.998825	Ortholog(s) have ubiquitin-protein ligase activity, role in protein ubiquitination involved in ubiquitin-dependent protein catabolic process and chromosome, centromeric region, mitotic spindle pole body, nucleus localization
orf19.7601	orf19.7601	37.06	34.02	26.73	30.44	-0.200555047	0.998825	Putative mitochondrial 2' O-ribose methyltransferase; predicted role in methylation of U(2791) in 21S rRNA; Spider biofilm induced
orf19.1985	orf19.1985	41.28	34	47.49	31.75	0.038444054	0.998825	Has aminoglycoside phosphotransferase and protein kinase domains; rat catheter and flow model biofilm induced
orf19.4014	orf19.4014	40.01	33.96	41.7	39.63	0.089988957	0.998825	Planktonic growth-induced gene
orf19.1033	STR2	42.34	33.92	34.88	36.41	-0.069079551	0.998825	Ortholog(s) have cystathionine gamma-synthase activity, role in transsulfuration and cytosol, nucleus localization
orf19.1711	END3	42.77	33.92	42.97	31.62	-0.033172569	0.998825	Regulated by Gcn4p; induced in response to amino acid starvation (3-aminotriazole treatment)

orf19.6186	orf19.6186	35.16	33.91	33.3	36.46	-0.00708521	0.998825	Predicted protein of unknown function; overlaps orf19.6185
orf19.2297	ARL3	31.66	33.87	37.5	37.23	0.095860395	0.998825	Putative Ras superfamily GTPase; induced by nitric oxide independent of Yhb1p
orf19.4807	orf19.4807	33.88	33.87	39.97	45.35	0.202165995	0.998825	Ortholog(s) have inorganic diphosphatase activity, role in aerobic respiration and mitochondrion localization
orf19.6498	orf19.6498	42.66	33.86	32.5	37.6	-0.079792551	0.998825	Ortholog(s) have role in RNA polymerase II complex localization to nucleus, RNA polymerase III complex localization to nucleus and DNA-directed RNA polymerase II, holoenzyme, cytoplasm localization
orf19.4857	orf19.4857	43.96	33.86	39.63	31.17	-0.095111338	0.998825	Protein with a dual-specificity phosphatase domain; Hap43-induced gene
orf19.804	orf19.804	36.17	33.85	38.37	31.26	-0.022513463	0.998825	Putative mitochondrial carrier family transporter; rat catheter biofilm induced
orf19.3221	CPA2	21.74	33.85	24.23	22.15	-0.167940956	NA	Putative arginine-specific carbamoylphosphate synthetase; protein enriched in stationary phase yeast cultures; rat catheter biofilm induced; Spider biofilm induced
orf19.7316	orf19.7316	36.78	33.84	33.14	30.62	-0.10169867	0.998825	Putative phytanoyl-CoA dioxygenase family protein; mutation confers hypersensitivity to 5-fluorocytosine (5-FC), 5-fluorouracil (5-FU), and tubercidin (7-deazaadenosine); induced by nitric oxide
orf19.7680	CTA26	30.94	33.84	59.42	60.45	0.585988539	0.280569	Putative transcription factor/activator; Med2 mediator complex ddomain; transcript is upregulated in an RHE model of oral candidiasis; member of a family of telomere-proximal genes; Efg1, Hap43-repressed

orf19.3539	orf19.3539	42.44	33.83	32.41	27.06	-0.235137232	0.964275	Predicted nucleolar S-adenosylmethionine-dependent rRNA methyltransferase; Spider biofilm induced
orf19.4388	orf19.4388	41.72	33.81	37.67	26.87	-0.150878375	0.998825	Protein with a histone fold domain; similar to TAFII47 proteins from S. cerevisiae, Danio rerio, Drosophila melanogaster, human, and mouse
orf19.835	orf19.835	35.89	33.77	37.35	30.56	-0.035525539	0.998825	Ortholog(s) have role in nucleosome mobilization and Ino80 complex, cytosol localization
orf19.4185	orf19.4185	37.23	33.75	29.79	34.95	-0.085661143	0.998825	Ortholog(s) have phosphoprotein phosphatase activity, thiosulfate sulfurtransferase activity, role in DNA-dependent DNA replication, protein dephosphorylation, regulation of DNA-dependent DNA replication and cytosol, nucleus localization
orf19.156	FGR51	37.04	33.7	48.26	34.3	0.123749343	0.998825	Protein lacking an ortholog in S. cerevisiae; transposon mutation affects filamentous growth; Hap43p-repressed gene
orf19.2616	UGT51C1	38.93	33.68	34.69	29.64	-0.113723904	0.998825	UDP-glucose:sterol glucosyltransferase; enzyme of sterol glucoside (membrane-bound lipid) biosynthesis; has UDP-sugar binding domain; activity is UDP-glucose-specific in vitro; enzyme does not use UDP-mannose; Mig1-regulated
orf19.1674	CDC53	39.5	33.68	41.6	37.58	0.078290509	0.998825	Cullin, a scaffold subunit of the SCF ubiquitin-ligase complexes; depletion leads to increased filamentous growth and premature cell death
orf19.827	orf19.827	41.28	33.66	48.76	38.52	0.143999134	0.998825	Ortholog of C. dubliniensis CD36 : Cd36_18620, C. parapsilosis CDC317 : CPAR2_212480, Candida tenuis NRRL Y-1498 : CANTEDRAFT_115371 and Debaryomyces hansenii CBS767 : DEHA2D06798g
orf19.5439	orf19.5439	30.34	33.65	27.82	28.2	-0.124289509	0.998825	Ortholog of C. parapsilosis CDC317 : CPAR2_504010, C. dubliniensis CD36 : Cd36_80270, Lodderomyces elongisporus NRLL YB-4239 : LELG_02294 and Pichia stipitis Pignal : PICST_29581

orf19.6760	MDS3	36.71	33.65	41.72	36.63	0.091267746	0.998825	TOR signaling pathway component; required for growth and hyphal formation at alkaline pH, for full virulence in a mouse model of systemic infection and for SD and Spider medium biofilm formation; role in chlamydospore formation
orf19.6511	LIG1	40.08	33.65	34.01	31.79	-0.111758034	0.998825	tRNA ligase; functional homolog of S. cerevisiae Trl1
orf19.4869	SFU1	34.25	33.64	32.49	35.1	-0.020668069	0.998825	GATA-type transcription factor; regulator of iron-responsive genes; represses iron utilization genes if iron is present; Hap43-repressed; promotes gastrointestinal commensalism in mice; Spider biofilm induced
orf19.2747	RGT1	26.61	33.62	27.55	32.82	-0.008468811	0.998825	Zn(II)2Cys6 transcription factor; transcriptional repressor involved in the regulation of glucose transporter genes; ortholog of S. cerevisiae Rgt1; mutants display decreased colonization of mouse kidneys
orf19.1636	STE50	33.61	33.6	33.92	26.77	-0.106275762	0.998825	Protein with sterile alpha motif (SAM) and Ras-associated domain (RAD); similar to S. cerevisiae Rad50p, which is involved in signal transduction via interaction with and regulation of MAPKKK
orf19.6796	YSA1	32.76	33.56	51.73	43.81	0.33347408	0.842252	Predicted Nudix hydrolase family member with ADP-ribose pyrophosphatase activity; role in metabolism of O-acetyl-ADP-ribose to AMP and acetylated ribose 5'-phosphate; rat catheter biofilm repressed
orf19.4322	DAP2	39.28	33.55	38.51	31.04	-0.047461793	0.998825	Putative dipeptidyl aminopeptidase; transcriptionally regulated during macrophage response
orf19.6866	orf19.6866	29.4	33.54	31.66	35.06	0.045958608	0.998825	Putative U1-70K component of the U1 snRNP, involved in splicing; ortholog of S. cerevisiae SNP1; downregulated upon adherence to polystyrene
orf19.4134	orf19.4134	34.76	33.52	36.05	26.06	-0.105482406	0.998825	Ortholog(s) have palmitoyltransferase activity, role in protein palmitoylation and plasma membrane localization

orf19.841	orf19.841	32.65	33.52	42.7	31.66	0.091921775	0.998825	Ortholog(s) have role in Golgi vesicle transport and Golgi membrane localization
orf19.536	orf19.536	37.04	33.48	47.23	35.46	0.137807705	0.998825	Ortholog(s) have chromatin binding, protein complex scaffold, ubiquitin binding activity and role in histone acetylation, transcription from RNA polymerase II promoter
orf19.3083	orf19.3083	31.94	33.48	31.54	31.68	-0.022055252	0.998825	Putative lipid phosphatase of the endoplasmic reticulum; role in DNA repair, actin cytoskeleton organization, cellular manganese ion homeostasis; Spider biofilm repressed
orf19.5814	orf19.5814	36.36	33.46	38.57	29.92	-0.00958535	0.998825	Protein of unknown function; Spider biofilm induced
orf19.6736	orf19.6736	39.71	33.43	31.39	30.3	-0.168049912	0.998825	Protein required for mitochondrial ribosome small subunit biogenesis; role in maturation of SSU-rRNA; Spider biofilm induced
orf19.645.1	VMA13	40.49	33.33	52.39	48.93	0.3095954	0.863837	Predicted proton-transporting ATPase; predicted role in ATP hydrolysis coupled proton transport; rat catheter biofilm repressed
orf19.6291	orf19.6291	35.71	33.3	35.9	31.82	-0.021594802	0.998825	Ortholog(s) have DNA binding, DNA-dependent ATPase activity, chromatin binding activity
orf19.4542	orf19.4542	38.19	33.29	36.04	32.41	-0.047231153	0.998825	Ortholog(s) have cyclin-dependent protein serine/threonine kinase inhibitor activity and role in phosphorylation of RNA polymerase II C-terminal domain, regulation of mitotic cell cycle, transcription from RNA polymerase II promoter
orf19.7602	AHA1	37.08	33.27	32.84	34.44	-0.046549553	0.998825	Putative Hsp90p co-chaperone; Hap43-repressed; heavy metal (cadmium) stress-induced; oxidative stress-induced via Cap1; rat catheter biofilm induced; flow model biofilm repressed

orf19.5947	SEC7	30	33.24	35.51	34.86	0.098402998	0.998825	Putative guanine nucleotide exchange factor (GEF); mutation confers hypersensitivity to Brefeldin A; caspofungin repressed
orf19.1026	orf19.1026	41.79	33.23	35.81	32.95	-0.083416388	0.998825	Ortholog(s) have role in exonucleolytic trimming to generate mature 3'-end of 5.8S rRNA from tricistronic rRNA transcript (SSU-rRNA, 5.8S rRNA and LSU-rRNA), more
orf19.4340	orf19.4340	45.31	33.21	45.38	42.72	0.109984213	0.998825	Ortholog of C. dubliniensis CD36 : Cd36_52890, C. parapsilosis CDC317 : CPAR2_501970, Candida tenuis NRRL Y-1498 : CANTEDRAFT_113546 and Debaryomyces hansenii CBS767 : DEHA2E09900g
orf19.686	orf19.686	38.92	33.2	37.89	39.85	0.068602898	0.998825	Protein of unknown function; regulated by Nrg1
orf19.2633.1	orf19.2633.1	29.73	33.15	18.25	10.26	-0.612777983	NA	Ortholog of C. dubliniensis CD36 : Cd36_00150 and Lodderomyces elongisporus NRLL YB-4239 : LELG_01269
orf19.6338	orf19.6338	32.63	33.14	40.81	47.5	0.262993814	0.931333	Ortholog of Candida albicans WO-1 : CAWG_05352
orf19.2528	orf19.2528	39.28	33.13	42.89	33.96	0.053656659	0.998825	Putative RNA polymerase III transcription factor (TFIIIB) subunit; flucytosine repressed
orf19.3312	orf19.3312	34.21	33.11	54.52	39.71	0.319957872	0.8593	Protein of unknown function; flow model and Spider biofilm repressed
orf19.3125	orf19.3125	26.25	33.11	31.08	29.59	0.018513969	0.998825	Ortholog(s) have cytosol, nucleus localization

orf19.4086	orf19.4086	36.66	33.1	36.85	30.06	-0.048551674	0.998825	Ortholog(s) have cytoplasm localization
orf19.843	orf19.843	35.5	33.04	38.27	38.59	0.107632986	0.998825	Putative DNA repair exonuclease; fungal-specific (no human or murine homolog)
orf19.1036	MNS1	33.31	33	55.04	51.41	0.450778979	0.569936	Alpha-1,2-mannosidase; processes Man9GlcNAc2 to Man8GlcNAc2 isomer B; member of ER localized glycosyl hydrolase family 47; ER form is converted by Kex2 to cytosolic form; flow model biofilm repressed
orf19.1137	orf19.1137	30.16	32.99	25.82	30.06	-0.108662024	0.998825	Ortholog(s) have thymidylate kinase activity, uridylate kinase activity, role in dTDP biosynthetic process, dTTP biosynthetic process, dUDP biosynthetic process and cytosol, nucleus localization
orf19.720	GST3	28.52	32.96	19.26	23.99	-0.303865799	0.892679	Glutathione S-transferase; expression regulated upon white-opaque switch; induced by human neutrophils; peroxide-induced; induced by alpha pheromone in SpiderM medium; Spider biofilm induced
orf19.4090.1	orf19.4090.1	27.63	32.96	40.88	37.31	0.212576578	0.998825	Ortholog(s) have role in vacuolar proton-transporting V-type ATPase complex assembly and endoplasmic reticulum membrane localization
orf19.7516	orf19.7516	31.89	32.96	34.28	29.76	-0.023124482	0.998825	Putative glycoside hydrolase; Hap43-induced gene
orf19.2772	HOS3	34.96	32.96	21.27	21.08	-0.407918012	0.723865	Histone deacetylase; similar to S. cerevisiae Hos3p; greater expression and longer mRNA in white cells, compared to opaque cells; has conserved deacetylation motif
orf19.2094	PDR6	32.35	32.94	26.29	25.79	-0.199232835	0.998825	Putative pleiotropic drug resistance regulatory protein 6; transcript regulated by Nrg1; induced by alpha pheromone in SpiderM medium

orf19.6990	orf19.6990	41.36	32.93	45.57	39.55	0.135479875	0.998825	Putative peptidyl-prolyl cis-trans isomerase; predicted endoplasmic reticulum (ER) localization
orf19.4979	KNS1	30.35	32.93	34.05	33.85	0.059316149	0.998825	Protein kinase involved in negative regulation of PolIII transcription; effector kinase of the TOR signaling pathway, phosphorylates Rpc53p to regulate ribosome and tRNA biosynthesis; Spider and flow model biofilm induced
orf19.3840	orf19.3840	32.75	32.87	38.89	31.18	0.043060207	0.998825	Putative serine/threonine kinase similar to S. cerevisiae Sak1; possibly an essential gene, disruptants not obtained by UAU1 method; Spider biofilm induced
orf19.304	orf19.304	35.58	32.86	26.06	26.29	-0.252371747	0.947586	Putative transporter similar to MDR proteins; fungal-specific; Spider biofilm induced
orf19.4677	orf19.4677	43.9	32.85	33.58	30.88	-0.160995251	0.998825	Putative protein of unknown function, transcript is upregulated in clinical isolates from HIV+ patients with oral candidiasis; Spider biofilm induced
orf19.1203	SRO77	35.73	32.82	42.34	34.83	0.092574606	0.998825	Protein with a predicted role in docking and fusion of post-Golgi vesicles with the plasma membrane; filament induced; fungal-specific (no human or murine homolog)
orf19.7153	orf19.7153	34.13	32.8	31.71	31.57	-0.048736348	0.998825	Putative exportin, member of the Exportin-T family; flow model biofilm repressed
orf19.808	orf19.808	40.16	32.77	35.31	27.73	-0.14100486	0.998825	Ortholog(s) have role in histone deacetylation, negative regulation of chromatin silencing at rDNA and negative regulation of chromatin silencing at silent mating-type cassette, more
orf19.4090	orf19.4090	30.67	32.76	30.59	31.68	-0.022155342	0.998825	Predicted membrane transporter, member of the fucose:proton symporter (FHS) family, major facilitator superfamily (MFS)

orf19.2362	orf19.2362	38.83	32.76	30	25.95	-0.229760007	0.977056	Putative 90S preribosome component; Hap43p-induced gene; possibly an essential gene, disruptants not obtained by UAU1 method
orf19.2384	MTG1	37.35	32.75	31.15	31.3	-0.115774901	0.998825	Putative mitochondrial GTPase; likely essential for respiratory competence and in large ribosomal subunit assembly; mitochondrial translation; Spider biofilm induced
orf19.207	PGA55	26.1	32.75	30.5	35.21	0.088658125	0.998825	GPI-anchored adhesin-like protein; filament induced; regulated by Nrg1, Tup1; regulated upon hyphal formation; mRNA binds to She3 and is localized to yeast-form buds and hyphal tips; induced during chlamydospore formation
orf19.2582	orf19.2582	38.88	32.73	41.35	32.81	0.016762533	0.998825	Ortholog(s) have role in cytoplasmic translation, ribosome-associated ubiquitin-dependent protein catabolic process and RQC complex, cytosol, cytosolic large ribosomal subunit localization
orf19.4662	RLM1	31.65	32.71	30.97	24.83	-0.138991927	0.998825	Putative transcription factor; required for wild-type resistance to cell wall perturbation, caspofungin treatment; regulates caspofungin induction of PGA13
orf19.2836	orf19.2836	21.93	32.68	47.71	43.72	0.453958965	0.596073	Protein with similarity to carbonic anhydrases
orf19.7231	FTR2	30.22	32.67	23.21	18.94	-0.340373757	0.857977	High-affinity iron permease; probably interacts with ferrous oxidase; regulated by iron level, ciclopirox olamine, amphotericin B, caspofungin; complements S. cerevisiae ftr1 iron transport defect; Hap43-repressed; Spider biofilm induced
orf19.7228	orf19.7228	43.31	32.66	33.53	27.84	-0.191268714	0.998825	Possible Golgi membrane protein; transcript positively regulated by Tbf1; mRNA binds She3
orf19.6953	IRS4	34.89	32.66	42.7	36.03	0.129906341	0.998825	Protein with roles in cell wall integrity, systemic (but not oral) murine infection, adherence, hyphal growth, and agar-embedded filamentous growth; antigenic in human infection; similar to S. cerevisiae Irs4p

orf19.1406	orf19.1406	33.15	32.64	32.07	37.97	0.046537303	0.998825	Ortholog(s) have DNA-directed DNA polymerase activity, role in error-free translesion synthesis, error-prone translesion synthesis and mitochondrion, nuclear chromatin, zeta DNA polymerase complex localization
orf19.4368	orf19.4368	34.77	32.59	38.01	33.25	0.030566899	0.998825	Has domain(s) with predicted hydrolase activity and role in cellular process
orf19.2863.1	ERV1	53.16	32.59	56.9	52.28	0.23033663	0.957711	Predicted component of the mitochondrial intermembrane space (IMS), involved in protein import into mitochondrial intermembrane space
orf19.7213	orf19.7213	38.15	32.58	39.3	33.19	0.00453121	0.998825	Putative ATP-dependent RNA helicase; fungal-specific (no human or murine homolog)
orf19.1663	MNT2	42.02	32.58	36.4	31.28	-0.11041965	0.998825	Alpha-1,2-mannosyl transferase; adds 3rd mannose in cell-wall mannoprotein biosynthesis; partially redundant with Mnt1; role in adherence and virulence; expressed in yeast and hyphae; fungal-specific; Hap43-induced; Spider biofilm induced
orf19.6135	orf19.6135	37.66	32.57	33.19	33	-0.073088826	0.998825	Ortholog of C. dubliniensis CD36 : Cd36_32970, C. parapsilosis CDC317 : CPAR2_701710, Candida tenuis NRRL Y-1498 : CANTEDRAFT_106599 and Debaryomyces hansenii CBS767 : DEHA2E04488g
orf19.6644	orf19.6644	29.55	32.54	30.47	19.47	-0.220323612	0.998825	Protein of unknown function; 2 predicted transmembrane domains; transcript detected on high-resolution tiling arrays; flow model biofilm induced; Spider biofilm repressed
orf19.1563	ECM3	33.87	32.5	33.55	29.13	-0.06396311	0.998825	Ortholog(s) have endoplasmic reticulum localization
orf19.920	RMT2	43.66	32.49	34.07	34.19	-0.107073083	0.998825	Minor protein arginine methyltransferases (PRMT) involved in methylation of arginine residues

orf19.4450.2	orf19.4450.2	39.52	32.48	21.02	27.14	-0.349698211	0.841359	Ortholog of C. dubliniensis CD36 : Cd36_06690, Debaryomyces hansenii CBS767 : DEHA2F26620g, Pichia stipitis Pignal : PICST_62738 and Spathaspora passalidarum NRRL Y-27907 : spas_CGOB_00038
orf19.4683	MLP1	28.99	32.47	28.97	26.47	-0.097877795	0.998825	Ortholog(s) have ribonucleoprotein complex binding activity
orf19.7518	ZCF38	37.15	32.46	37.58	37.45	0.069944044	0.998825	Putative Zn(II)2Cys6 transcription factor
orf19.955	orf19.955	36.28	32.41	51.2	43.29	0.300291293	0.878136	Protein of unknown function; Spider biofilm repressed
orf19.6295	MAS2	42.46	32.36	37.97	36.65	0.006631417	0.998825	Putative processing peptidase, catalytic (alpha) subunit; protein level decreases in stationary phase cultures
orf19.3706	orf19.3706	37.81	32.36	46.36	45.13	0.243550305	0.946319	Has domain(s) with predicted oxidoreductase activity, zinc ion binding activity and role in oxidation-reduction process
orf19.5093	orf19.5093	47.09	32.33	39.52	35.78	-0.041670328	0.998825	Ortholog(s) have DNA translocase activity
orf19.3446	orf19.3446	28.89	32.33	29.75	31.93	0.005998294	0.998825	Ortholog(s) have Ran GTPase binding activity, role in protein import into nucleus and nucleus localization
orf19.6508	orf19.6508	40.64	32.31	50.72	47.38	0.278653269	0.902628	Ortholog(s) have protein transporter activity, role in Golgi to plasma membrane transport, intracellular protein transport and vesicle coat localization

orf19.3991	orf19.3991	35.76	32.3	32.23	35.56	-0.005919284	0.998825	Ortholog(s) have role in cellular lipid metabolic process and cytosol localization
orf19.6078	POL93	32.09	32.27	41.01	25.69	0.028513265	0.998825	Predicted ORF in retrotransposon Tca8 with similarity to the Pol region of retrotransposons encoding reverse transcriptase, protease and integrase; downregulated in response to ciclopirox olamine; F-12/CO2 early biofilm induced
orf19.1314	orf19.1314	38.37	32.27	34.71	30.6	-0.060763892	0.998825	Protein of unknown function; planktonic growth-induced gene
orf19.6341	orf19.6341	30.4	32.25	29.7	33.4	0.006386422	0.998825	Ortholog(s) have 5-amino-6-(5-phosphoribosylamino)uracil reductase activity, role in riboflavin biosynthetic process and cytosol, nucleus localization
orf19.2088	DPB4	27.63	32.23	40.61	34.07	0.189531452	0.998825	Putative DNA polymerase epsilon subunit D; null mutant is viable but slow-growing and displays abnormal invasive growth on SD and YPD media; Spider biofilm repressed
orf19.2680	orf19.2680	34.04	32.21	38.33	32.81	0.057540022	0.998825	Ortholog(s) have phospholipid-translocating ATPase activity, role in intracellular protein transport, phospholipid translocation and trans-Golgi network transport vesicle localization
orf19.1285	orf19.1285	28.19	32.2	21.88	20.55	-0.279108601	0.929903	Plasma membrane-localized protein of unknown function; Hap43p-repressed gene
orf19.6039	orf19.6039	44.06	32.15	43.01	41.69	0.100922844	0.998825	Ortholog(s) have SNAP receptor activity and role in ER to Golgi vesicle-mediated transport, Golgi organization, intra-Golgi vesicle-mediated transport, vesicle fusion with Golgi apparatus
orf19.52	orf19.52	37.74	32.13	45.36	38.33	0.151768235	0.998825	Ortholog(s) have role in cellular iron ion homeostasis and mitochondrion localization

orf19.5234	RBD1	39.88	32.13	49.32	44.89	0.249442618	0.943089	Rhomboid-like protein; similar to putative intramembrane serine proteases; role in filamentous growth; Gcn4-regulated; caspofungin repressed; Spider biofilm induced
orf19.5069	orf19.5069	24.82	32.12	14.59	12.5	-0.528778502	NA	Ortholog of S. cerevisiae Sae3; meiosis specific protein involved in DMC1-dependent meiotic recombination in S. cerevisiae; Spider biofilm induced
orf19.1754	CMK2	36.8	32.11	41.89	37.2	0.117970158	0.998825	Putative calmodulin-dependent protein kinase; involved in cell wall integrity and oxidative stress response
orf19.2467	PRN1	33.21	32.11	26.42	26.04	-0.198791715	0.998825	Protein with similarity to pirins; induced by benomyl and in response to alpha pheromone in SpiderM medium; transcript induced by Mnl1 in weak acid stress; rat catheter and Spider biofilm induced
orf19.3893	SCW11	39.67	32.06	42.12	42.59	0.151144253	0.998825	Cell wall protein; repressed in ace2 mutant; repressed in core caspofungin response; induced in high iron; possibly an essential gene, disruptants not obtained by UAU1 method; rat catheter and Spider biofilm repressed
orf19.4531	orf19.4531	34.31	32.04	29.67	28.08	-0.121256238	0.998825	Putative PDR-subfamily ABC transporter; Spider biofilm induced
orf19.1570	ERG7	31.86	32.02	44.59	43.22	0.289961945	0.892679	2,3-epoxysqualene-lanosterol cyclase (lanosterol synthase), conversion of 2,3-oxidosqualene to lanosterol in sterol biosynthesis; fluconazole-induced; possibly essential, disruptants not obtained by UAU1 method; rat catheter biofilm induced
orf19.6076	orf19.6076	30.3	31.99	36.75	27.55	0.030683259	0.998825	Ortholog(s) have protein transporter activity and role in ascospore formation, cellular response to cadmium ion, detoxification of cadmium ion, intracellular protein transport, retrograde transport, endosome to Golgi
orf19.22	orf19.22	32.25	31.98	15.82	14.78	-0.568674445	NA	Protein with homology to peroxisomal membrane proteins; Sef1p-, Sfu1p-, and Hap43p-regulated gene

orf19.3080	orf19.3080	30.86	31.96	35.5	33.79	0.073009138	0.998825	Ortholog(s) have role in attachment of spindle microtubules to kinetochore, cellular response to nitrogen starvation, cohesin localization to chromatin and double-strand break repair, more
orf19.773	orf19.773	43.8	31.94	26.28	24.62	-0.35382824	0.824505	Protein similar to S. cerevisiae Rsa3 predicted nucleolar protein involved in maturation of pre-60S ribosomal particles; rat catheter and Spider biofilm induced
orf19.2128	orf19.2128	32.27	31.94	32.97	30.13	-0.022688117	0.998825	Ortholog(s) have mitochondrion localization
orf19.4963	orf19.4963	37.11	31.93	32.02	34.81	-0.032513824	0.998825	Ortholog(s) have role in ER-associated ubiquitin-dependent protein catabolic process and cytoplasm, nucleolus localization
orf19.2742	orf19.2742	27.8	31.92	56.3	42.97	0.458215864	0.570984	Ortholog of C. parapsilosis CDC317 : CPAR2_400095, C. dubliniensis CD36 : Cd36_42560, Candida tenuis NRRL Y-1498 : CANTEDRAFT_113280 and Lodderomyces elongisporus NRLL YB-4239 : LELG_04166
orf19.457	orf19.457	38.43	31.91	38.24	35.93	0.044250284	0.998825	Ortholog(s) have ubiquitin-specific protease activity, role in protein deubiquitination, ubiquitin-dependent protein catabolic process via the multivesicular body sorting pathway and cytosol localization
orf19.2988	orf19.2988	31.93	31.91	40.83	41.25	0.221419547	0.987362	Predicted aminotransferase based on S. pombe ortholog SPBC660.12c; flow model biofilm induced
orf19.1565	orf19.1565	33.82	31.9	33.83	28.7	-0.050131591	0.998825	Protein of unknown function
orf19.6298	SPB4	36.46	31.85	30.22	24.16	-0.208699076	0.998825	Putative ATP-dependent RNA helicase; flucytosine repressed; Spider biofilm induced

orf19.2066	orf19.2066	33.21	31.84	41.07	41.43	0.207906449	0.998825	Ortholog(s) have cytoplasm localization
orf19.4059	orf19.4059	34.32	31.82	31.46	32.46	-0.048526166	0.998825	Ortholog(s) have role in actin cortical patch localization, arginine transport, cellular protein localization, cellular response to heat, endocytosis, late endosome to vacuole transport and lysine transport, more
orf19.6818	orf19.6818	32.37	31.78	28.6	29.12	-0.093671354	0.998825	Ortholog(s) have cytosol, mitochondrion localization
orf19.6598	WAL1	36.34	31.76	33.75	30.2	-0.062351525	0.998825	Protein required for hyphal growth and for wild-type cell morphology, polarized budding, endocytosis, vacuole morphology; similar to Wiskott-Aldrich syndrome protein; localizes to cortical actin patches and hyphal tips
orf19.4689	PGA57	34.33	31.63	23.52	15.77	-0.416168551	0.711296	Putative GPI-anchored protein; Hap43p-induced gene
orf19.1200	orf19.1200	37.31	31.61	33.81	30.77	-0.061310085	0.998825	Protein of unknown function; Spider biofilm induced
orf19.465	IFF9	29.76	31.61	33.18	38.18	0.113946362	0.998825	Predicted GPI-linked cell-wall protein; similar to Hyr1; induced in low iron; possibly an essential gene, disruptants not obtained by UAU1 method
orf19.6492	orf19.6492	36.18	31.59	27.1	22.98	-0.271688516	0.931333	Predicted protein serine/threonine kinase and/or protein tyrosine kinase; Spider biofilm induced
orf19.4605	TYR1	43.89	31.56	37	36.7	-0.029932495	0.998825	Putative prepephenate dehydrogenase; enzyme of tyrosine biosynthesis; fungal-specific (no human or murine homolog)

orf19.4642	orf19.4642	25.46	31.55	32.82	42.45	0.233549678	0.974208	Protein of unknown function; Hap43-induced gene
orf19.6989	orf19.6989	42.93	31.55	34.63	28.95	-0.150491426	0.998825	Putative U2 snRNP-associated protein; Spider biofilm induced
orf19.5269.1	orf19.5269.1	26.56	31.53	59.61	69.51	0.764090325	0.07733	Ortholog(s) have endoplasmic reticulum localization
orf19.2198	FLC3	35.32	31.53	27.7	28.71	-0.148414655	0.998825	Protein involved in heme uptake; putative FAD transporter, similar to S. cerevisiae Flc3p
orf19.6374	orf19.6374	32.04	31.53	29.14	25.49	-0.155069536	0.998825	Ortholog(s) have unfolded protein binding activity, role in mitochondrial proton-transporting ATP synthase complex assembly and integral to mitochondrial membrane, mitochondrial inner membrane localization
orf19.3265.1	orf19.3265.1	29.09	31.5	33.85	41.08	0.193125934	0.998825	Ortholog of C. parapsilosis CDC317 : CPAR2_800920, Candida tenuis NRRL Y-1498 : CANTEDRAFT_109948, Debaryomyces hansenii CBS767 : DEHA2E17182g and Pichia stipitis Pignal : psti_CGOB_00115
orf19.488	MEX67	40.4	31.49	42.99	35.89	0.091981737	0.998825	Nuclear export protein; has NTF2-like domain; interacts with Mtr2p via the NTF2-like domain
orf19.4062	TRY2	37.54	31.47	30.39	32.05	-0.1008922	0.998825	Transcription factor; regulator of yeast form adherence; required for yeast cell adherence to silicone substrate; Spider biofilm induced
orf19.7326	orf19.7326	31.69	31.46	31.28	26.27	-0.095579774	0.998825	Ortholog(s) have protein-lysine N-methyltransferase activity, role in peptidyl-lysine dimethylation and cytoplasm, nucleus localization

orf19.5139	orf19.5139	36.49	31.45	41.75	36.87	0.144694573	0.998825	Ortholog of C. dubliniensis CD36 : Cd36_72800, Candida tropicalis MYA-3404 : CTRG_05211 and Candida albicans WO-1 : CAWG_05649
orf19.1362	SMM1	37.91	31.44	28.07	20.61	-0.299763679	0.892679	Putative dihydrouridine synthase; Hap43-induced gene; rat catheter biofilm induced; Spider biofilm induced
orf19.4437	orf19.4437	33.01	31.43	42.6	35.43	0.171905947	0.998825	Ortholog(s) have ATPase activity, DNA binding, nucleosome binding, rDNA binding activity
orf19.4109	PMT4	32.17	31.43	60.19	62.36	0.643469475	0.19844	Protein mannosyltransferase; required for WT cell wall composition and virulence in mice; roles in hyphal growth and drug sensitivity; one of 5 family members; Axl2 a substrate; Spider biofilm repressed; Bcr1-repressed in RPMI a/a biofilm
orf19.5967	FGR44	33.41	31.43	39.69	36.79	0.161312376	0.998825	Protein lacking an ortholog in S. cerevisiae; transposon mutation affects filamentous growth
orf19.1975	orf19.1975	51.31	31.4	45.44	41.01	0.040970371	0.998825	Ortholog(s) have role in mRNA splicing, via spliceosome, regulation of DNA methylation and U4/U6 x U5 tri-snRNP complex, U5 snRNP localization
orf19.4380.1	orf19.4380.1	31.46	31.39	41.46	45.2	0.296155419	0.888061	Ortholog(s) have role in mitochondrial genome maintenance and integral to mitochondrial inner membrane localization
orf19.945	VPS2	43.02	31.37	51.26	44.5	0.244471045	0.946319	Ortholog(s) have role in intralumenal vesicle formation, protein retention in Golgi apparatus, protein targeting to vacuole involved in ubiquitin-dependent protein catabolic process via the multivesicular body sorting pathway
orf19.3158	orf19.3158	36.7	31.33	29.44	25.52	-0.192153391	0.998825	Ortholog(s) have cytoplasm localization

orf19.932	orf19.932	29.43	31.3	24	23.38	-0.210335855	0.998825	Putative aminophospholipid translocase (flippase); merged with orf19.2226 in Assembly 21; possibly an essential gene, disruptants not obtained by UAU1 method
orf19.3615	orf19.3615	37.34	31.3	46.02	36.72	0.17747039	0.998825	Protein of unknown function; induced in core caspofungin response; expression upregulated in an ssr1 null mutant; induced by nitric oxide independent of Yhb1p
orf19.771	LPG20	37.22	31.28	35.51	32.45	-0.007436927	0.998825	Aldo-keto reductase family protein; similar to aryl alcohol dehydrogenases; osmotic stress-induced, correlates with overexpression of MDR1 in fluconazole-resistant isolate; stationary phase enriched protein
orf19.477	orf19.477	37.57	31.26	36.5	30.2	-0.034499999	0.998825	Ortholog(s) have role in mitochondrial translation and mitochondrion localization
orf19.4626	orf19.4626	33	31.24	38.29	26.88	0.00343309	0.998825	Ortholog(s) have role in TOR signaling cascade, positive regulation of transcription from RNA polymerase I promoter and cytosol, extrinsic to membrane, nucleus localization
orf19.685.1	orf19.685.1	25.71	31.21	24.72	21.05	-0.184010323	0.998825	Ortholog of C. parapsilosis CDC317 : CPAR2_602135, Candida tenuis NRRL Y-1498 : cten_CGOB_00042, Debaryomyces hansenii CBS767 : DEHA2F11858g and Pichia stipitis Pignal : PICST_60705
orf19.7257	MLH3	36.46	31.21	29.11	23.1	-0.226008884	0.988598	Ortholog(s) have role in meiotic mismatch repair, reciprocal meiotic recombination and MutLbeta complex localization
orf19.1993	orf19.1993	44.47	31.19	32.93	34.86	-0.09361284	0.998825	Ortholog(s) have structural molecule activity, role in proteasome assembly, ubiquitin-dependent protein catabolic process and nucleus, proteasome regulatory particle, lid subcomplex, proteasome storage granule localization
orf19.1598	ERG24	36.19	31.19	61.82	65.8	0.640250139	0.196416	C-14 sterol reductase, has a role in ergosterol biosynthesis; mutation confers increased sensitivity to dyclonine; rat catheter and Spider biofilm repressed

orf19.5943	orf19.5943	43.17	31.18	47.66	36.85	0.122945264	0.998825	Ortholog(s) have role in peroxisome organization and peroxisomal membrane localization
orf19.4173	orf19.4173	42.83	31.16	27.36	23.83	-0.329110428	0.8593	Ortholog(s) have role in peptidyl-diphthamide biosynthetic process from peptidyl-histidine and cytosol localization
orf19.3881	orf19.3881	40.58	31.16	34.91	34.89	-0.031236925	0.998825	Ortholog of C. dubliniensis CD36 : Cd36_31790, C. parapsilosis CDC317 : CPAR2_204900, Candida tenuis NRRL Y-1498 : CANTEDRAFT_136864 and Debaryomyces hansenii CBS767 : DEHA2F13200g
orf19.2135	TSM1	33.34	31.15	32.95	28.58	-0.036440357	0.998825	Putative transcription initiation factor TFIID subunit; transcript is upregulated in clinical isolates from HIV+ patients with oral candidiasis; Nrg1-regulated
orf19.1368	orf19.1368	23.25	31.15	22.07	18.42	-0.243089678	NA	Protein of unknown function; flow model biofilm induced; Spider biofilm induced
orf19.2190	VRP1	25.06	31.08	40.35	31.73	0.208739344	0.998825	Verprolin-related protein involved in actin cytoskeleton organization and polarized morphogenesis; interacts with Wal1p and Myo5p; downregulated upon adherence to polystyrene
orf19.3742	orf19.3742	27.93	31.08	28.38	26.53	-0.068425332	0.998825	Protein of unknown function; Spider biofilm induced; upregulated in a cyr1 null mutant
orf19.829	SCH9	35.77	31.07	26.25	21.56	-0.29052637	0.90951	Protein kinase; involved in growth control, cell size, resistance to rapamycin, cations, chlamydospore formation, filamentous growth under some conditions, and virulence; prevents hyphal growth in hypoxia at high CO2; Spider biofilm induced
orf19.273	orf19.273	38.72	31.04	43.65	32.63	0.079271936	0.998825	Ortholog(s) have unfolded protein binding activity, role in sterol biosynthetic process and endoplasmic reticulum localization

orf19.2099	HIR1	37.4	31.03	34.13	31.62	-0.033798401	0.998825	Component of the HIR complex, a nucleosome assembly factor involved in chromatin formation; involved in regulation of white-opaque switching frequency
orf19.7522	orf19.7522	32.14	31.01	41.62	45.11	0.293699913	0.89191	Protein with a pyridoxal phosphate-dependent transferase domain; Hap43-repressed; mutation confers hypersensitivity to amphotericin B; Spider biofilm repressed
orf19.4504	orf19.4504	31.58	31.01	57.93	40.36	0.40406152	0.692027	Has domain(s) with predicted oxidoreductase activity, transferase activity, transferring acyl groups other than amino-acyl groups, zinc ion binding activity and role in oxidation-reduction process
orf19.6656	orf19.6656	26.97	31	38.04	32.57	0.168628714	0.998825	Spermidine transporter; induced in strains from HIV patients with oral candidiasis; alkaline repressed; amphotericin B induced; colony morphology regulated by Ssn6; reduced oral epithelial cell damage by mutant; Spider biofilm induced
orf19.3603	MFG1	30.83	30.98	28.48	26.77	-0.11087028	0.998825	Regulator of filamentous growth; required for biofilm formation, virulence; interacts with Flo8 and Mss11
orf19.5567	POP4	33.24	30.97	25.71	21.41	-0.267120805	0.938642	Ortholog of S. cerevisiae Pop4; a subunit of both RNase MRP and nuclear RNase P; filament induced; regulated by Nrg1, Tup1; likely essential, based on UAU1 strategy; rat catheter and Spider biofilm induced
orf19.6810	orf19.6810	35.48	30.96	38.44	37.85	0.122643222	0.998825	Ortholog(s) have protein serine/threonine phosphatase activity
orf19.3055	MED17	29.21	30.95	25.2	26.72	-0.128061504	0.998825	Putative RNA polymerase II mediator complex subunit; possibly an essential gene, disruptants not obtained by UAU1 method
orf19.3773	CDL1	33.5	30.93	31.13	27.74	-0.079290557	0.998825	Putative RNAse III, ortholog of S. cerevisiae RNT1; merged with orf19.3772 in Assembly 21

orf19.7381	AHR1	33.91	30.93	78.88	58.24	0.733396008	0.093194	Zn(II)2Cys6 transcription factor; involved in regulation of adhesion genes; involved in white-opaque switch; forms complex with Mcm1; mutant is sensetive to 5-fluorocytosine and lithium chloride; Spider biofilm induced
orf19.3197	PAP1	33.31	30.85	43.66	37.42	0.208347371	0.998825	Poly(A) polymerase, likely involved in mRNA polyadenylation; PAP is inhibited by parnafungin antifungals; non-sex gene located within the MTLa mating-type-like locus
orf19.7563	BET2	32.69	30.84	38.46	39.91	0.174537617	0.998825	Putative Type II geranylgeranyltransferase beta subunit; transcript regulated by Mig1
orf19.7451	FUN31	31.5	30.84	26.9	23.94	-0.173815193	0.998825	Putative PAS kinase involved in cell wall damage response; similar to S. cerevisiae Psk1p, a putative serine/threonine protein kinase; induced by Mnl1 under weak acid stress; rat catheter and Spider biofilm induced
orf19.904	orf19.904	25.11	30.84	28.04	31.57	0.050065238	0.998825	Ortholog(s) have cytosol, nucleus localization
orf19.2886	CEK1	36.57	30.81	42.06	33.66	0.093939379	0.998825	ERK-family protein kinase; required for wild-type yeast-hypha switch, mating efficiency, virulence in mice; Cst20-Hst7-Cek1-Cph1 MAPK pathway regulates mating, and invasive hyphal growth under some conditions; Spider biofilm induced
orf19.191	KIC1	32.37	30.77	36.26	25.4	-0.038608876	0.998825	Member of the GCK-III subfamily of eukaryotic Ste20p kinases; in RAM cell wall integrity signaling network; role in cell separation, azole sensitivity; required for hyphal growth; constitutive expression is MTL, white-opaque independent
orf19.5449	orf19.5449	36.24	30.75	35.53	45.53	0.182172539	0.998825	Predicted integral membrane protein; Spider biofilm induced
orf19.3825	RCE1	37.71	30.74	41.86	37.36	0.118366169	0.998825	Putative Type II CAAX prenyl protease; induced during the mating process

orf19.6933	orf19.6933	27.77	30.73	29.44	25.07	-0.084534719	0.998825	Ortholog(s) have peptidyl-prolyl cis-trans isomerase activity, protein phosphatase type 2A regulator activity
orf19.5764	SKI8	37.21	30.7	44.56	36.44	0.154004379	0.998825	Ortholog(s) have role in nuclear-transcribed mRNA catabolic process, 3'-5' exonucleolytic nonsense-mediated decay, nuclear-transcribed mRNA catabolic process, non-stop decay and protein complex assembly, more
orf19.862	orf19.862	36.63	30.69	41.37	26.04	-0.023356466	0.998825	Protein of unknown function; Spider biofilm induced
orf19.3108	orf19.3108	41.02	30.66	43.85	34.99	0.078688003	0.998825	Putative DNA repair methyltransferase; induced by nitric oxide independent of Yhb1; Spider biofilm induced
orf19.746	orf19.746	29.1	30.65	28.54	29.49	-0.037513432	0.998825	Has domain(s) with predicted role in protein transport
orf19.5684.1	orf19.5684.1	26.47	30.64	27.09	25.69	-0.070114473	0.998825	Protein of unknown function
orf19.5609	orf19.5609	31.17	30.64	33.33	27.96	-0.023437267	0.998825	Ortholog of C. dubliniensis CD36 : Cd36_63840, C. parapsilosis CDC317 : CPAR2_601510, Debaryomyces hansenii CBS767 : DEHA2A06270g and Pichia stipitis Pignal : PICST_31363
orf19.6849	ELC1	43.58	30.63	25.13	40.73	-0.113086353	0.998825	Putative elongin C; transcript regulated by Nrg1 and Mig1; regulated by Gcn2 and Gcn4
orf19.2018.1	orf19.2018.1	22.16	30.62	33.14	43.52	0.315210983	0.87796	PProtein of unknown function; gene has intron; similar to human BLOC1S2

orf19.5643	ECM7	34.34	30.59	32.44	31.23	-0.035106968	0.998825	Protein involved in control of calcium homeostasis and oxidative stress response; mutants show defects in hyphal growth
orf19.1976	TRX2	32.28	30.57	28.81	31.62	-0.034991626	0.998825	Similar to thioredoxins; not expected to be enzymatically active because it lacks a canonical thioredoxin active site (Trp-Cys-Gly-Pro-Cys); TRX1 encodes all of the cytoplasmic/nuclear thioredoxin function in C. albicans; Hap43p-induced
orf19.2029	RFC5	28.14	30.55	35.3	32.87	0.115990062	0.998825	Putative heteropentameric replication factor C subunit; periodic mRNA expression, peak at cell-cycle G1/S phase
orf19.5864	URK1	36.43	30.52	25.13	24.32	-0.2734062	0.931333	Protein similar to uridine kinase; repressed by ciclopirox olamine; Spider biofilm induced
orf19.4855	BUD31	40.11	30.51	28.7	33.03	-0.125478932	0.998825	Bud31 ortholog; not subject to mating-type regulation, in contrast to S. cerevisiae Bud31 which has a role in specifying the bud site; Spider biofilm induced
orf19.1300	orf19.1300	40.45	30.5	42.01	33.9	0.055223858	0.998825	Putative mitochondrial membrane protein; homozygous transposon insertion causes decreased colony wrinkling under filamentous growth-inducing conditions, but does not block true hyphal formation in liquid media
orf19.719	orf19.719	31.18	30.39	50.43	49.14	0.4516776	0.579712	Ortholog(s) have ubiquitin-protein ligase activity
orf19.5672	MEP2	34.63	30.35	31.44	24.09	-0.152439521	0.998825	Ammonium permease and regulator of nitrogen starvation-induced filamentation; 11 predicted transmembrane regions; in low nitrogen cytoplasmic C-terminus activates Ras/cAMP and MAPK signal transduction pathways to induce filamentation
orf19.1816	ALS3	31.03	30.33	53.54	45.04	0.449146314	0.586088	Cell wall adhesin; epithelial adhesion, endothelial invasion; alleles vary in adhesiveness; immunoprotective in mice; binds SspB adhesin of S. gordonii in mixed biofilm; induced in/required for Spider biofilm; flow model biofilm repressed

orf19.2236	FHL1	30.06	30.32	27.86	23.03	-0.145522667	0.998825	Fork-head transcription factor; forms a heterodimer with Ifh1 that is tethered to promoters by Tbf1; positively regulates rRNA and ribosomal protein gene transcription
orf19.7065	PSF3	27.02	30.29	20.75	18.2	-0.307702496	NA	Putative GINS complex subunit; cell-cycle regulated periodic mRNA expression; Spider biofilm induced
orf19.202	CDC47	30.85	30.29	58.16	42.99	0.465882421	0.55586	Phosphorylated protein described as having role in control of cell division; RNA abundance regulated by tyrosol and cell density; merged with orf19.201 in Assembly 20; unmerged from orf19.201 in a revision of Assembly 21
orf19.970	orf19.970	35.25	30.29	30.22	26.2	-0.136865232	0.998825	Protein with a predicted role in microtubule-related processes; Spider biofilm induced
orf19.2434	NPL4	34.07	30.24	33.05	31.39	-0.007316834	0.998825	Putative ubiquitin-binding protein; regulated by Gcn2p and Gcn4p
orf19.985	orf19.985	34.41	30.22	37.98	32.18	0.072289702	0.998825	Ortholog(s) have endoplasmic reticulum, ribosome localization
orf19.2043	orf19.2043	29.15	30.18	26.77	24.67	-0.114684759	0.998825	Ortholog of C. dubliniensis CD36 : Cd36_15750, C. parapsilosis CDC317 : CPAR2_213000, Debaryomyces hansenii CBS767 : DEHA2D18524g and Pichia stipitis Pignal : PICST_42355
orf19.7159	orf19.7159	30.11	30.16	28.07	24.08	-0.129490493	0.998825	Putative protein of unknown function; Hap43p-repressed gene; ortholog of S. cerevisiae YMR185W
orf19.5958	CDR2	26.44	30.13	16.53	14.45	-0.450352445	NA	Multidrug transporter, ATP-binding cassette (ABC) superfamily; transports phospholipids, in-to-out direction; overexpressed in azole-resistant isolates; repressed in young biofilms

orf19.6444	orf19.6444	30.3	30.12	30.39	25.83	-0.067869387	0.998825	Ortholog(s) have role in cellular response to amino acid starvation, meiosis, negative regulation of TOR signaling cascade, regulation of autophagic vacuole assembly
orf19.3736	KAR4	30.13	30.12	33.26	38.64	0.156145759	0.998825	Transcription factor; required for gene regulation in response to pheromones; ortholog of S. cerevisiae Kar4; role in karyogamy; opaque-specific, a-specific; induced by alpha factor
orf19.7198	orf19.7198	35.79	30.1	37.59	35.68	0.097682073	0.998825	CCR4-NOT complex component; involved in controlling mRNA initiation, elongation and degradation; rat catheter biofilm induced
orf19.4182	orf19.4182	31.63	30.1	33.26	28.68	-0.008367266	0.998825	Ortholog(s) have role in response to drug and mitochondrion localization
orf19.6889	MKK2	30.93	30.08	39.18	36.94	0.191340652	0.998825	Ortholog of S. cerevisiae Mkk2; MAP kinase kinase involved in signal transduction; macrophage-downregulated; mutants are viable and hypersensitive to caspofungin
orf19.747	NBP35	31.3	30.06	23.56	20.95	-0.258675699	0.946319	Similar to nucleotide-binding proteins; increased transcription is observed upon benomyl treatment; transcription is induced upon filamentous growth
orf19.4174	orf19.4174	35.51	30.02	36.1	30.75	0.005888848	0.998825	Ortholog(s) have nicotinamide riboside transmembrane transporter activity, role in nicotinamide riboside transport, transmembrane transport and fungal-type vacuole membrane localization
orf19.3371	JAB1	32.83	29.99	32.14	26.87	-0.065501259	0.998825	Component of the COP9 signalosome (CSN) complex, provides catalytic activity for deneddylation that cleaves the ubiquitin-like protein tag Rub1p off the cullin (Cdc53p) molecule
orf19.4824	orf19.4824	41.48	29.97	37.25	32.94	-0.019064903	0.998825	Planktonic growth-induced gene

orf19.3823	ZDS1	28.52	29.96	40.69	38.12	0.256072496	0.943089	Nonessential protein; similar to S. cerevisiae Zds1p
orf19.7450	orf19.7450	24.1	29.95	31.94	33.98	0.163235527	0.998825	Ortholog(s) have role in cytokinesis, septin ring assembly and cellular bud neck septin ring localization
orf19.2290	TOR1	29.16	29.89	29.03	28.07	-0.037799161	0.998825	Protein similar to TOR family phosphatidylinositol kinases; mutation confers resistance to rapamycin; involved in regulation of ribosome biogenesis, starvation response, and adhesion
orf19.5728	orf19.5728	36.13	29.88	33.09	29.44	-0.062704213	0.998825	Putative cytochrome P450; Spider biofilm induced
orf19.1010	MNT3	26.75	29.88	21.9	22.72	-0.199339272	NA	Mannosyltransferase; transcription elevated in cyr1, chk1, nik1, and sln1 homozygous null mutants; fungal-specific (no human or murine homolog)
orf19.2607	orf19.2607	31.94	29.88	26.95	32.65	-0.033949044	0.998825	Protein of unknown function; Spider biofilm induced
orf19.3920	orf19.3920	33.67	29.87	30.85	27.9	-0.078664798	0.998825	Ortholog(s) have protein disulfide isomerase activity, protein disulfide oxidoreductase activity, role in protein folding and fungal-type vacuole localization
orf19.837	GNA1	43.91	29.85	34.16	35.9	-0.054630545	0.998825	Glucosamine-6-phosphate acetyltransferase; enzyme of UDP-GlcNAc biosynthesis; required for viability in absence of GlcNAc supplementation; required for persistent infection and wild-type virulence in mouse systemic infection
orf19.137	orf19.137	36.48	29.84	28.72	32.16	-0.074839319	0.998825	Putative transferase involved in phospholipid biosynthesis; induced by alpha pheromone in SpiderM medium

orf19.3795	AGP3	38.03	29.81	34.79	33.69	0.007135676	0.998825	Putative serine transporter; possible role in assimilation of sulfur; F-12/CO2 early biofilm induced
orf19.388	CAF16	33.54	29.8	34.62	24.24	-0.080155362	0.998825	ABC family protein, predicted not to be a transporter; Hap43, caspofungin repressed; rat catheter and Spider biofilm repressed
orf19.5902	RAS2	26.48	29.8	14.67	20.33	-0.363445958	NA	Protein similar to S. cerevisiae Ras2; has opposite effects to Ras1; Tn mutation affects filamentous growth; rat catheter and Spider biofilm induced
orf19.4324	orf19.4324	31.18	29.76	27.25	30.03	-0.064876191	0.998825	Has domain(s) with predicted NADH dehydrogenase (ubiquinone) activity, electron carrier activity and membrane localization
orf19.640	orf19.640	35.93	29.75	22.73	25.39	-0.27439259	0.931333	Integral membrane protein of the ER; role in the synthesis of beta-1,6-glucan in the cell wall; S. cerevisiae ortholog required for cell viability; Spider biofilm induced
orf19.4645	BEM1	32.01	29.75	33.48	25.97	-0.052170131	0.998825	Protein required for wild-type budding, hyphal growth, and virulence in a mouse systemic infection; suppresses pseudohyphal and filamentous growth defects of various S. cerevisiae mutants and heat sensitivity of S. cerevisiae cdc24-4 mutant
orf19.6457	orf19.6457	38.52	29.75	34.59	30.98	-0.046613112	0.998825	Ortholog of S. cerevisiae : YBL086C, C. glabrata CBS138 : CAGL0C01815g, C. dubliniensis CD36 : Cd36_72270, C. parapsilosis CDC317 : CPAR2_702850 and Candida tenuis NRRL Y-1498 : CANTEDRAFT_107537
orf19.3434	TRY5	34.08	29.73	36.89	20.45	-0.109172139	0.998825	Zn(II)2Cys6 transcription factor; regulator of yeast form adherence; required for yeast cell adherence to silicone substrate
orf19.2230	orf19.2230	38.72	29.73	40.85	42.51	0.178966008	0.998825	Putative pre-mRNA-splicing factor; decreased transcription is observed upon benomyl treatment

orf19.331	orf19.331	40.98	29.71	25.04	30.49	-0.225663858	0.987997	Ortholog(s) have S-adenosylmethionine-dependent methyltransferase activity, protein methyltransferase activity and role in peptidyl-glutamine methylation, regulation of translation
orf19.3102	CTA6	28.8	29.69	30.22	25.12	-0.068988998	0.998825	Protein that activates transcription in 1-hybrid assay in S. cerevisiae
orf19.5657	SWI1	32.91	29.67	35.85	31.26	0.046498599	0.998825	Protein involved in transcription regulation; ortholog of S. cerevisiae Swi1p, which is a subunit of the SWI/SNF chromatin remodeling complex; interacts with Snf2p; SWI/SNF complex is essential for hyphal growth and virulence
orf19.2754	TIM13	33.39	29.67	36.39	45.61	0.236371699	0.957711	Predicted mitochondrial intermembrane space protein with a role in protein import into mitochondria
orf19.2473	orf19.2473	37.64	29.66	30.95	28.02	-0.131692027	0.998825	Putative RSC chromatin remodeling complex component; decreased transcription is observed upon fluphenazine treatment or in an azole-resistant strain that overexpresses CDR1 and CDR2
orf19.6203	PUT3	29.77	29.57	27.44	28.11	-0.081555277	0.998825	Zn(II)2Cys6 transcription factor; has similarity to S. cerevisiae Put3, a transcription factor involved in the regulation of proline utilization genes
orf19.4144	orf19.4144	36.24	29.57	33.08	27.55	-0.077127808	0.998825	Predicted protein kinase; clade-associated gene expression
orf19.4423	orf19.4423	34.12	29.55	36.37	29.52	0.01903005	0.998825	Putative glucosyltransferase; localized to the mitochondrial membrane
orf19.1761	orf19.1761	31.06	29.53	50.62	40.4	0.362679369	0.789669	Predicted olichyl-diphosphooligosaccharide-protein glycotransferase; role in protein N-linked glycosylation; Spider biofilm repressed

orf19.7345	orf19.7345	33.4	29.45	28.07	23.94	-0.156922208	0.998825	Ortholog(s) have ATP binding activity and cytosol, nucleus localization
orf19.7060	orf19.7060	28.27	29.42	19.7	17.56	-0.326201454	NA	Ortholog of C. dubliniensis CD36 : Cd36_70070, C. parapsilosis CDC317 : CPAR2_301080, Debaryomyces hansenii CBS767 : DEHA2F22946g and Pichia stipitis Pignal : PICST_46820
orf19.3445	HOC1	34.09	29.34	38.56	39.33	0.188909885	0.998825	Protein with similarity to mannosyltransferases; similar to S. cerevisiae Hoc1p and C. albicans Och1p
orf19.4970	orf19.4970	34.81	29.32	24.54	21.1	-0.282261619	0.921805	Protein of unknown function; Spider biofilm induced
orf19.4207	orf19.4207	33.23	29.31	32.49	27.28	-0.051080803	0.998825	Predicted ORF from Assembly 19; removed from Assembly 20; restored based on comparative genome analysis
orf19.4533	orf19.4533	33.74	29.31	28.38	25.56	-0.13902513	0.998825	Ortholog(s) have ribosome localization
orf19.2813	orf19.2813	25.41	29.31	23.4	29.23	-0.037705687	0.998825	Ortholog of Candida albicans WO-1 : CAWG_02711
orf19.1973	HAP5	32.01	29.3	31.03	22.05	-0.127984144	0.998825	Component of CCAAT-binding transcription factor; roles in filamentous growth, low-iron induction of FRP1; regulates genes involved in respiratory growth; functional homolog of S. cerevisiae Hap5p; Cap1p-dependent expression in low iron
orf19.387	GCR3	31.44	29.29	28.87	31.55	0.007998571	0.998825	Functional homolog of S. cerevisiae Gcr3, which acts in regulation of glycolytic genes; no intron predicted, in contrast to intron in S. cerevisiae GCR3 gene

orf19.7088	orf19.7088	35.28	29.23	30.95	32.73	-0.005632877	0.998825	Ortholog(s) have single-stranded telomeric DNA binding activity, role in regulation of translational fidelity, telomere maintenance, threonylcarbamoyladenosine metabolic process and cytosol, nucleus localization
orf19.3787	orf19.3787	35.42	29.2	33.32	28.11	-0.049639851	0.998825	Ortholog(s) have chromatin DNA binding activity and role in positive regulation of transcription from RNA polymerase II promoter, telomere maintenance, threonylcarbamoyladenosine metabolic process
orf19.1035	WAR1	30.24	29.19	21.11	18.78	-0.318304574	NA	Zn(II)2Cys6 transcription factor; plays a role in resistance to weak organic acids; required for yeast cell adherence to silicone substrate; Spider biofilm induced
orf19.6967	USO6	23.61	29.18	31	32.93	0.151982436	0.998825	Putative vesicular transport protein; transcript induced by filamentous growth; rat catheter biofilm repressed
orf19.3676	ABP140	35.03	29.18	25.5	25.29	-0.2150377	0.998825	Ortholog of S. cerevisiae actin-binding protein Abp140; Hap43-induced; F-12/CO2 early biofilm induced
orf19.6709	orf19.6709	31.63	29.17	33.78	34.17	0.089602475	0.998825	Predicted alpha/beta hydrolase; Spider biofilm induced
orf19.789.1	orf19.789.1	28.73	29.17	30	38.04	0.133332569	0.998825	Ortholog(s) have signal sequence binding activity, role in protein targeting to ER and signal recognition particle, endoplasmic reticulum targeting localization
orf19.7539	INO2	29.81	29.14	35.31	25.44	0.004223463	0.998825	Transcriptional activator that forms a heterodimer with Ino4p; likely regulates genes involved in phosphatidylcholine and phosphatidylinositol biosynthesis, fatty acid beta-oxidation, and peroxisome biogenesis
orf19.4596	orf19.4596	28.4	29.09	32.81	25.23	0.004531917	0.998825	Protein of unknown function; Spider biofilm induced

orf19.6558	orf19.6558	29.85	29.08	34.96	35.09	0.14439516	0.998825	Has domain(s) with predicted zinc ion binding activity, role in ER to Golgi vesicle-mediated transport, intracellular protein transport and COPII vesicle coat localization
orf19.1600	orf19.1600	25.61	29.07	32.9	31.33	0.121368607	0.998825	Ortholog of C. dubliniensis CD36 : Cd36_23530, C. parapsilosis CDC317 : CPAR2_406140, Candida tenuis NRRL Y-1498 : CANTEDRAFT_116829 and Debaryomyces hansenii CBS767 : DEHA2B01826g
orf19.4107	orf19.4107	32.32	29.05	34.09	31.91	0.062188639	0.998825	Exopolyphosphatase, hydrolyzes inorganic polyphosphate (poly P) into Pi residues; Spider biofilm repressed
orf19.2938	orf19.2938	33.6	29.01	30.95	28.65	-0.049579836	0.998825	Putative mitochondrial inner membrane protein with a predicted role in the assembly of respiratory complex III; Hap43p-repressed gene; S. cerevisiae ortholog FMP25 localizes to mitochondrion
orf19.1707	orf19.1707	36.1	29	41.79	34.7	0.147318322	0.998825	Ortholog of Candida albicans WO-1 : CAWG_02476
orf19.5502	orf19.5502	26.67	29	27.38	20.14	-0.152267343	0.998825	Ortholog of C. dubliniensis CD36 : Cd36_73420, Debaryomyces hansenii CBS767 : DEHA2A05456g, Pichia stipitis Pignal : PICST_30999 and Candida guilliermondii ATCC 6260 : PGUG_00155
orf19.4415	MED3	35.84	28.99	25.35	20.46	-0.312652581	0.880321	RNA polymerase II mediator complex subunit
orf19.3894	orf19.3894	29.1	28.91	23.7	18.73	-0.250064396	NA	Putative inositol oxygenase; mutation confers hypersensitivity to toxic ergosterol analog; Spider biofilm induced
orf19.780	DUR1,2	38.34	28.91	24.75	19.88	-0.338697642	0.857977	Urea amidolyase; hydrolyzes urea to CO2; use of urea as N source and for hyphal switch in macrophage; regulated by Nrg1/Hap43; required for virulence; promotes mouse kidney and brain colonization; rat catheter and flow model biofilm induced

orf19.1324	RAD2	31.01	28.9	20.93	18.2	-0.351387236	NA	Ortholog of S. cerevisiae Rad2, a nucleotide excision repair nuclease; null mutant is extremely sensitive to UV irradiation; transcript induced by interaction with macrophages
orf19.1531	orf19.1531	31.23	28.89	25.73	26.41	-0.127794982	0.998825	Ortholog(s) have RNA binding, protein heterodimerization activity, role in mRNA cleavage, mRNA polyadenylation and cytosol, mRNA cleavage factor complex, mitochondrion localization
orf19.2391	orf19.2391	33.8	28.89	35.28	26.01	-0.036958746	0.998825	Putative protein of unknown function; Hap43p-repressed gene; S. cerevisiae ortholog YKR023W localizes to mitochondria
orf19.6053	CIS2	33.16	28.88	29.98	26.68	-0.080061773	0.998825	Putative role in regulation of biogenesis of the cell wall; upregulated in biofilm; Gcn4p-regulated
orf19.5040	orf19.5040	36.82	28.88	30.06	26.31	-0.149301003	0.998825	Ortholog(s) have nucleocytoplasmic transporter activity, phospholipid binding, single-stranded DNA binding activity
orf19.2901	NUP60	29.8	28.88	29.51	27.28	-0.037776246	0.998825	Ortholog of S. cerevisiae Nup60p; a subunit of the nuclear pore complex; mutants are viable
orf19.801	TBF1	37.26	28.87	29.08	23.82	-0.195301042	0.998825	Essential transcription factor; induces ribosomal protein genes and the rDNA locus; acts with Cbf1 at subset of promoters; recruits Fhl1 and Ifh1 to promoters; role is analogous to that of S. cerevisiae Rap1; Spider biofilm induced
orf19.2846	orf19.2846	28.38	28.85	37.41	37.4	0.224647558	0.987362	Protein of unknown function; Hap43-repressed; induced in core caspofungin response; regulated by yeast-hypha switch; Spider biofilm repressed
orf19.3332	orf19.3332	30.27	28.84	37.89	27.16	0.073846861	0.998825	Ortholog of Bre4 in S. cerevisiae has a role in brefeldin A resistence; a drug that affect intracellular transport; Spider biofilm induced

orf19.3227	FTH2	36.36	28.84	33.12	31.65	-0.006206034	0.998825	Putative iron transporter; similar to S. cerevisiae Fth1p
orf19.6326	orf19.6326	37.33	28.84	39.65	24.16	-0.036639383	0.998825	Ortholog of C. dubliniensis CD36 : Cd36_60130, C. parapsilosis CDC317 : CPAR2_603380, Candida tenuis NRRL Y-1498 : CANTEDRAFT_94402 and Debaryomyces hansenii CBS767 : DEHA2G06556g
orf19.7127	TLO16	47.45	28.82	62.16	45.12	0.323198001	0.854173	Member of a family of telomere-proximal genes of unknown function; may be spliced in vivo
orf19.2743	orf19.2743	24.29	28.81	24.03	21.71	-0.12267028	NA	Ortholog(s) have RNA polymerase II core promoter proximal region sequence-specific DNA binding transcription factor activity involved in negative regulation of transcription, more
orf19.795	VPS36	31.88	28.81	31.24	29.65	-0.00719993	0.998825	ESCRT II protein sorting complex subunit; involved in Rim8 processing and proteolytic activation of Rim101, which regulates pH response; role in uptake of Histatin-5 into vacuole
orf19.7243	orf19.7243	30.67	28.8	31.15	34.47	0.063850217	0.998825	Deoxycytidine monophosphate (dCMP) deaminase; role in dUMP and dTMP biosynthesis; Spider biofilm repressed
orf19.742	ALD6	35.22	28.8	39.34	32.48	0.08545582	0.998825	Putative aldehyde dehydrogenase; stationary phase enriched protein; expression regulated upon white-opaque switch; rat catheter biofilm induced; rat catheter and Spider biofilm induced
orf19.4502	orf19.4502	24.25	28.79	31.14	35.47	0.179487419	0.998825	Ortholog(s) have ATPase activity, DNA binding, TBP-class protein binding activity
orf19.3691	TIM21	36.55	28.76	38.8	29.62	0.03186137	0.998825	Component of the Translocase of the Inner Mitochondrial membrane (TIM23 complex); for protein import into mitochondria; Hap43, ketoconazole-repressed; Spider biofilm repressed

orf19.6377	orf19.6377	28.77	28.76	26.99	31.39	-0.006528926	0.998825	Ortholog(s) have protein C-terminal leucine carboxyl O-methyltransferase activity, role in C-terminal protein methylation, cellular protein complex assembly and mitochondrion localization
orf19.2166	VMA5	37.45	28.76	44.25	37.5	0.181907624	0.998825	Putative vacuolar H(+)-ATPase; plasma membrane localized; rat catheter biofilm repressed
orf19.6155	orf19.6155	33.53	28.73	26.84	23	-0.202556393	0.998825	Ortholog(s) have DNA ligase (ATP) activity and role in DNA ligation, DNA recombination, DNA replication, Okazaki fragment processing, base-excision repair, mitotic cell cycle, nucleotide-excision repair, telomere maintenance
orf19.5765	NUP82	30.34	28.71	39.4	33.77	0.18177574	0.998825	Linker nucleoporin of the nuclear pore complex; role in mRNA andexport from nucleus, protein import into nucleus, ribosomal large subunit export from nucleus, ribosomal small subunit export from nucleus; rat catheter biofilm repressed
orf19.879	orf19.879	31.73	28.7	30.38	25.86	-0.081366952	0.998825	Ortholog(s) have myosin binding activity, role in intracellular mRNA localization, mating type switching and cytoplasm localization
orf19.2790	SWD2	35.54	28.66	42.46	35.25	0.147318322	0.998825	Ortholog(s) have histone methyltransferase activity (H3-K4 specific) activity
orf19.1525	orf19.1525	30.22	28.65	32.48	30.13	0.035100728	0.998825	Ortholog(s) have role in nuclear-transcribed mRNA catabolic process, nonsense-mediated decay and cytoplasm, nucleus localization
orf19.5611	orf19.5611	32.13	28.65	11.83	8.01	-0.782291066	NA	Predicted 3-methylbutanol:NAD(P) oxidoreductase and methylglyoxal reductase (NADPH-dependent); role in ergosterol metabolic process; early stage flow model biofilm induced; Spider biofilm induced
orf19.4910	FGR41	35.5	28.64	34.64	27.04	-0.036169809	0.998825	Putative GPI-anchored adhesin-like protein; transposon mutation affects filamentous growth; Spider biofilm repressed

orf19.861	CAP4	33.06	28.64	36.26	22.33	-0.067222868	0.998825	Predicted bZip transcription factor; possibly an essential gene, disruptants not obtained by UAU1 method
orf19.6049	orf19.6049	29.8	28.63	31.88	26	-0.023758529	0.998825	Ortholog(s) have protein kinase activator activity, role in protein phosphorylation, regulation of cytokinesis and chromosome passenger complex, kinetochore microtubule, spindle midzone localization
orf19.4240	orf19.4240	30.85	28.63	38.19	34.7	0.168126815	0.998825	Ortholog(s) have role in GPI anchor biosynthetic process, cellular manganese ion homeostasis and endoplasmic reticulum, fungal-type vacuole membrane localization
orf19.3753	SEF1	31.04	28.62	25.08	21.2	-0.226985754	0.991735	Zn2-Cys6 transcription factor; regulates iron uptake; negatively regulated by Sfu1p, positively regulated by Tbf1; promotes virulence in mice; mutants display decreased colonization of mouse kidneys; Spider biofilm induced
orf19.6795	orf19.6795	35.35	28.61	46.45	44.19	0.313566291	0.868721	Ortholog(s) have enzyme activator activity
orf19.1881	LPT1	30.15	28.6	47.14	40.68	0.360874556	0.795912	Lysophospholipid acyltransferase, involved in phospholipid remodeling; role in glycerophospholipid biosynthesis; rat catheter biofilm repressed
orf19.2425	HGT18	31.49	28.6	40.38	35.12	0.192762926	0.998825	Putative glucose transporter of the major facilitator superfamily; the C. albicans glucose transporter family comprises 20 members; 12 probable membrane-spanning segments; expressed in rich medium with 2% glucose
orf19.5561	STE23	35.02	28.58	30.28	24.76	-0.137963324	0.998825	Ortholog of S. cerevisiae Ste23 metalloprotease; role in N-terminal processing of pro-a-factor to the mature form; Tn mutation affects filamentous growth; Spider biofilm induced
orf19.4997	KIS2	32.12	28.56	35.49	28.64	0.033599757	0.998825	Scaffold protein of Snf1p complex; similar to S. cerevisiae Gal83p and Sip2p; interacts with Snf4p; interaction with Snf1p complex is regulated by carbon source, decreased on ethanol; N-terminal myristoylation; Hog1p-downregulated

orf19.1301	orf19.1301	33.27	28.51	43.45	42.25	0.285434548	0.901691	Ortholog(s) have cytoplasm, nucleus, ribosome localization
orf19.7512	orf19.7512	27.48	28.51	28.9	31.41	0.051155576	0.998825	Has domain(s) with predicted electron carrier activity, heme binding, iron ion binding, oxidoreductase activity, acting on paired donors and with incorporation or reduction of molecular oxygen, more
orf19.1119	MTR10	30.75	28.5	33.28	26.83	-0.008837719	0.998825	Putative importin; member of a family of fungal-specific nuclear importins; Mig1-regulated
orf19.2587	HNM3	29.62	28.49	24.57	26.67	-0.097762561	0.998825	Putative transporter; Hap43, flucytosine repressed; possibly essential, disruptants not obtained by UAU1 method; Spider biofilm induced
orf19.2584	OPT9	19.83	28.48	17.87	20.31	-0.182413032	NA	Probable pseudogene similar to fragments of OPT1 oligopeptide transporter gene; decreased expression in hyphae compared to yeast-form cells; transcriptionally induced upon phagocytosis by macrophage
orf19.31	orf19.31	22.88	28.48	44.67	47.18	0.525767828	0.444301	Ortholog of C. parapsilosis CDC317 : CPAR2_201960, C. dubliniensis CD36 : Cd36_20900, Candida tropicalis MYA-3404 : CTRG_04756 and Candida albicans WO-1 : CAWG_04394
orf19.5628	orf19.5628	20.34	28.46	26.97	28.79	0.115030609	0.998825	Mitochondrial dicarboxylate transporter; possibly an essential gene, disruptants not obtained by UAU1 method
orf19.3643	orf19.3643	21.91	28.45	15.31	18.01	-0.310987827	NA	Protein of unknown function; Hap43-repressed gene
orf19.5132	orf19.5132	21.61	28.45	23.12	7.35	-0.355806962	NA	Protein of unknown function; transcript detected on high-resolution tiling arrays; Spider biofilm induced

orf19.5510	orf19.5510	30.11	28.44	29.89	25.46	-0.053412554	0.998825	Ortholog(s) have role in chromatin silencing at telomere, negative regulation of transcription from RNA polymerase II promoter by pheromones and CHRAC localization
orf19.4096	TAZ1	30.38	28.39	27.27	29.52	-0.020980843	0.998825	Putative lyso-phosphatidylcholine acyltransferase, required for normal phospholipid content of mitochondrial membranes; rat catheter biofilm induced
orf19.4337	orf19.4337	26.63	28.36	18.59	14.1	-0.393441528	NA	Predicted membrane transporter; monocarboxylate porter (MCP) family, major facilitator superfamily (MFS); Spider biofilm induced; rat catheter biofilm repressed
orf19.5772	orf19.5772	34.47	28.34	38.81	34.73	0.152725936	0.998825	Ortholog(s) have role in histone exchange and Swr1 complex, cytosol localization
orf19.4630	CPA1	27.91	28.34	28.85	31.57	0.066409666	0.998825	Putative carbamoyl-phosphate synthase subunit; alkaline repressed; rat catheter, Spider and flow model biofilm induced
orf19.7103	orf19.7103	35.37	28.32	34.24	25.84	-0.050352694	0.998825	Predicted exonuclease; Spider biofilm induced
orf19.3665	SET6	41.5	28.31	22.5	22.49	-0.361591407	0.823496	Ortholog of S. cerevisiae Set6, a SET domain protein of unknown function; Hap43-induced; rat catheter and Spider biofilm induced
orf19.1734	orf19.1734	26.65	28.31	25.96	22.35	-0.119911791	0.998825	Putative ATPase and nucleosome spacing factor; heterozygous null mutant displays sensitivity to virgineone
orf19.3460	orf19.3460	45.2	28.3	36.66	23.27	-0.177346184	0.998825	Protein of unknown function; mRNA binds She3; transcript regulated upon yeast-hypha switch; induced in oralpharyngeal candidasis

orf19.641	orf19.641	31.87	28.3	33.04	31.35	0.049437693	0.998825	Ortholog(s) have role in protein folding and endoplasmic reticulum localization
orf19.4893	orf19.4893	29.24	28.26	31.49	26.36	-0.008765155	0.998825	Ortholog of C. dubliniensis CD36 : Cd36_09630, C. parapsilosis CDC317 : CPAR2_805080, Candida tenuis NRRL Y-1498 : CANTEDRAFT_136811 and Debaryomyces hansenii CBS767 : DEHA2B12364g
orf19.430	YPT53	31.47	28.25	28.48	26.43	-0.082339893	0.998825	Rab-family GTPase involved in vacuolar trafficking, colocolizes with Vps1p and Ypt52p in late endosome; Hap43-repressed gene
orf19.5808	orf19.5808	36.05	28.22	27.24	20.95	-0.24772969	0.956789	Ortholog(s) have protein-lysine N-methyltransferase activity, role in negative regulation of G2/M transition of mitotic cell cycle, peptidyl-lysine methylation and cytosol, nucleus localization
orf19.6336	PGA25	28.8	28.22	28.08	29.63	0.008045788	0.998825	Putative GPI-anchored adhesin-like protein; fluconazole-downregulated; induced in oralpharyngeal candidasis; Spider biofilm induced
orf19.4665	orf19.4665	25.65	28.21	26.86	26.21	-0.023905612	0.998825	Protein of unknown function; Spider biofilm induced
orf19.5572	orf19.5572	22.17	28.2	23.97	26.69	0.007524914	NA	Protein of unknown function; Spider biofilm repressed
orf19.4171	orf19.4171	30.11	28.19	27.84	17.25	-0.213846657	0.998825	Has domain(s) with predicted ATP binding activity
orf19.3833	orf19.3833	24.62	28.19	30.35	23.98	0.005219741	0.998825	Putative DNA-binding transcription factor; decreased transcription is observed upon fluphenazine treatment; possibly an essential gene, disruptants not obtained by UAU1 method; Hap43p-induced gene

orf19.348	SKN2	31.54	28.19	30.18	24.95	-0.08200164	0.998825	Protein with a potential role in beta-1,6 glucan biosynthesis; similarity to Kre6 and Skn1; possibly essential, disruptants not obtained by UAU1 method; Hap43-induced; flow model biofilm induced; rat catheter biofilm repressed
orf19.4969	KEM1	32.62	28.18	33.66	28.26	0.006228191	0.998825	5'->3' exoribonuclease of cytoplasmic stress granules; role in filamentous growth; complements slow growth/mating of S. cerevisiae kem1 mutant; required for SD or Spider medium biofilm formation
orf19.1034	orf19.1034	19.73	28.18	24.25	24.59	0.006632416	NA	Protein with a predicted cytochrome b5-like Heme/Steroid binding domain; Hap43, caspofungin repressed; flow model biofilm induced
orf19.6061	orf19.6061	35.94	28.18	29.52	23.32	-0.167974348	0.998825	Ortholog(s) have role in N-acylethanolamine metabolic process, N-acylphosphatidylethanolamine metabolic process and integral to mitochondrial inner membrane localization
orf19.7199	orf19.7199	40.92	28.17	44.27	39.25	0.166015267	0.998825	Ortholog(s) have TRC complex, nucleus localization
orf19.4221	ORC4	31.23	28.15	32.13	28.29	0.006569528	0.998825	Phosphorylated protein similar to S. cerevisiae Orc4, subunit of the origin recognition complex (ORC); induced by alpha pheromone in SpiderM medium
orf19.6984	orf19.6984	31.71	28.15	15.85	14.56	-0.509295062	NA	Ortholog of C. dubliniensis CD36 : Cd36_85460, C. parapsilosis CDC317 : CPAR2_405760, Candida tenuis NRRL Y-1498 : CANTEDRAFT_116052 and Debaryomyces hansenii CBS767 : DEHA2D16940g
orf19.6830	orf19.6830	28.15	28.14	51.63	44.95	0.503132222	0.480175	Putative enoyl-CoA hydratase; Spider biofilm induced
orf19.684	orf19.684	30.9	28.13	30.94	29.25	0.00713284	0.998825	Putative transcription factor with zinc finger DNA-binding motif; heterozygous null mutant exhibits hypersensitivity to parnafungin and cordycepin in the C. albicans fitness test

orf19.35.1	orf19.35.1	37.77	28.09	48.18	44.65	0.318195475	0.859429	Protein of unknown function
orf19.6778	orf19.6778	29.21	28.05	38.23	36.78	0.237823363	0.957711	Aminophospholipid translocase (flippase); maintains membrane lipid asymmetry in post-Golgi secretory vesicles; contributes to clathrin-coated vesicle formation and endocytosis; flow model biofilm repressed
orf19.7276.1	TLO4	37.64	28.03	17.44	23.86	-0.386788807	0.780641	Member of a family of telomere-proximal genes of unknown function; transcript induced in an RHE model of oral candidiasis; Hap43-repressed
orf19.384	orf19.384	32.35	28.02	36.68	36.55	0.18201962	0.998825	Ortholog(s) have Golgi apparatus, cytosol, nucleus localization
orf19.6506	orf19.6506	30.43	28.01	27.59	26.14	-0.067941422	0.998825	Ortholog(s) have role in histone deacetylation, negative regulation of antisense RNA transcription, regulation of DNA-dependent DNA replication initiation, transcription elongation from RNA polymerase II promoter
orf19.3970	orf19.3970	25.16	27.98	24.59	24.32	-0.072318121	NA	Putative ribosome biogenesis factor; possibly essential, disruptants not obtained by UAU1 method; rat catheter and Spider biofilm induced
orf19.3762	orf19.3762	33.75	27.93	39.81	30.39	0.099350769	0.998825	Ortholog of C. dubliniensis CD36 : Cd36_11720, C. parapsilosis CDC317 : CPAR2_201890, Candida tenuis NRRL Y-1498 : CANTEDRAFT_91948 and Debaryomyces hansenii CBS767 : DEHA2E16786g
orf19.3099	TRP4	26.92	27.92	36.47	28.69	0.13430697	0.998825	Predicted enzyme of amino acid biosynthesis; upregulated in biofilm; regulated by Gcn2p and Gcn4p; S. cerevisiae ortholog is Gcn4p regulated
orf19.1736	orf19.1736	24.51	27.91	29.88	29.19	0.081492031	0.998825	Protein of unknown function; transcript detected on high-resolution tiling arrays

orf19.2898	orf19.2898	35.04	27.87	23.74	18.45	-0.336943024	0.858769	Ortholog(s) have borate efflux transmembrane transporter activity, role in borate transport, protein targeting to vacuole and fungal-type vacuole, plasma membrane localization
orf19.396	EAF6	26.97	27.87	34.15	27.79	0.092929344	0.998825	Subunit of the NuA4 histone acetyltransferase complex; Hap43-repressed; Spider biofilm repressed
orf19.3594	orf19.3594	32.72	27.87	22.36	29.6	-0.137092222	0.998825	Putative adhesin-like protein; predicted ORF from Assembly 19; removed from Assembly 20; subsequently reinstated in Assembly 21 based on comparative genome
orf19.194	orf19.194	33.89	27.86	27.57	22.02	-0.188187811	0.998825	Ortholog of C. dubliniensis CD36 : Cd36_19300, C. parapsilosis CDC317 : CPAR2_209720, Candida tenuis NRRL Y-1498 : CANTEDRAFT_114035 and Debaryomyces hansenii CBS767 : DEHA2E10494g
orf19.3275	orf19.3275	35.35	27.77	29.21	30.71	-0.047716337	0.998825	Ortholog of C. dubliniensis CD36 : Cd36_25870, C. parapsilosis CDC317 : CPAR2_804000, Candida tenuis NRRL Y-1498 : CANTEDRAFT_115661 and Debaryomyces hansenii CBS767 : DEHA2B05852g
orf19.1413	YFH1	37.6	27.74	30.22	19.29	-0.256488596	0.946319	Frataxin; may be required for iron storage or delivery; role in oxidative stress resistance; transcript regulated by Nrg1, repressed if iron absent induced by macrophage interaction; Hap43-repressed gene
orf19.2244	orf19.2244	34.63	27.73	51.02	46.29	0.40204611	0.692027	Similar to oxidoreductases and to S. cerevisiae Yjr096wp; Sfu1 repressed; induced by benomyl treatment, Ssr1; Hap43-repressed; flow model biofilm repressed
orf19.3856	CDC28	30.1	27.72	40.69	36.52	0.259519303	0.938642	Cyclin-dependent protein kinase; interacts with regulatory subunit Cyb1; determination of cell morphology during the cell cycle; phosphorylated mostly by Swe1 and phosphorylation is regulated by Hsl1; 5'-UTR intron; Spider biofilm repressed
orf19.7204	orf19.7204	33.49	27.69	16.26	9.84	-0.630783008	NA	Ortholog(s) have glyoxysome localization

orf19.425	orf19.425	25.74	27.68	22.42	21.11	-0.189111854	NA	Has domain(s) with predicted translation release factor activity and role in translational termination
orf19.192	orf19.192	31.28	27.67	32.72	27.05	0.005999782	0.998825	Ortholog of C. dubliniensis CD36 : Cd36_19320, C. parapsilosis CDC317 : CPAR2_209670, Candida tenuis NRRL Y-1498 : CANTEDRAFT_113478 and Debaryomyces hansenii CBS767 : DEHA2C15136g
orf19.164	orf19.164	28.44	27.65	18.91	19.98	-0.290899327	NA	Ortholog(s) have lipase activity and peroxisomal matrix localization
orf19.1586	FGR22	28.82	27.58	28.13	22.01	-0.117330689	0.998825	Putative phosphatidylinositol-specific phospholipase C (PI-PLC); predicted type 2 membrane protein; no S. cerevisiae ortholog; role in, and regulated by, filamentation, Hap43p; almost identical to orf19.5797
orf19.1057	orf19.1057	26.22	27.57	24.11	25.11	-0.086889144	0.998825	Ortholog of C. dubliniensis CD36 : Cd36_03980, C. parapsilosis CDC317 : CPAR2_107050, Candida tenuis NRRL Y-1498 : CANTEDRAFT_113999 and Debaryomyces hansenii CBS767 : DEHA2D12518g
orf19.3111	PRA1	27.14	27.56	17	16.15	-0.393600804	NA	Cell surface protein that sequesters zinc from host tissue; enriched at hyphal tips; released extracellularly; binds to host complement regulators; mediates leukocyte adhesion and migration; immunogenic in mouse; produced at ambient pH
orf19.5474	orf19.5474	25.38	27.52	40.29	49.18	0.461307099	0.579712	Protein of unknown function; induced by Mnl1 under weak acid stress; transcript detected on high-resolution tiling arrays; Spider biofilm repressed
orf19.6591	PCD1	30.38	27.51	27.6	22.36	-0.131864922	0.998825	Ortholog(s) have 8-oxo-7,8-dihydroguanosine triphosphate pyrophosphatase activity, role in DNA repair and peroxisome localization
orf19.2054	FGR15	30.48	27.48	21.58	20.02	-0.251570482	NA	Putative transcription factor with zinc finger DNA-binding motif; lacks an ortholog in S. cerevisiae; transposon mutation affects filamentous growth; caspofungin induced; mutation causes marginal increase in caspofungin sensitivity

orf19.6182	ZCF34	32.51	27.47	27.84	25.15	-0.111551652	0.998825	Zn(II)2Cys6 transcription factor involved in regulation of multidrug resistance via control of CDR1 expression; mutants display decreased colonization of mouse kidneys; required for yeast cell adherence to silicone substrate
orf19.5426	orf19.5426	35.86	27.45	36.79	28.47	0.019862709	0.998825	Putative patatin-like phospholipase; macrophage-regulated gene; fungal-specific (no human or murine homolog)
orf19.7574	orf19.7574	30.43	27.45	27.34	21.49	-0.149236107	0.998825	Ortholog(s) have ubiquitin ligase complex localization
orf19.1933	orf19.1933	27.55	27.44	38.06	31.85	0.202525231	0.998825	Ortholog(s) have role in peroxisome organization and peroxisomal membrane localization
orf19.1177	orf19.1177	28.48	27.44	32.91	35.81	0.191589602	0.998825	Ortholog of S. cerevisiae Rtt106; histone chaperone that regulates chromatin structure in transcribed and silenced chromosomal regions; affects transcriptional elongation; Hap43-repressed; Spider biofilm repressed
orf19.2826	orf19.2826	29.04	27.43	36.26	33.86	0.188945016	0.998825	Ortholog of C. dubliniensis CD36 : Cd36_27680, C. parapsilosis CDC317 : CPAR2_801140, Candida tenuis NRRL Y-1498 : CANTEDRAFT_129982 and Debaryomyces hansenii CBS767 : DEHA2E09526g
orf19.4873	orf19.4873	24.39	27.43	8.36	13.68	-0.563199139	NA	Protein of unknown function; transcript regulated by white-opaque switch; flow model biofilm induced; Spider biofilm induced
orf19.964	SMC4	36.53	27.41	28.77	24.17	-0.166697077	0.998825	Ortholog(s) have chromatin binding activity and role in cellular protein localization, mitotic chromosome condensation, tRNA gene clustering
orf19.6305	orf19.6305	38.43	27.41	34.95	34.09	0.048396844	0.998825	Hydroxytrimethyllysine aldolase, the second enzyme in the carnitine biosynthesis pathway; rat catheter biofilm repressed

orf19.5718	orf19.5718	27.98	27.41	25.98	24.05	-0.086194384	0.998825	Has domain(s) with predicted zinc ion binding activity and role in intracellular protein transport, vesicle-mediated transport
orf19.1592	orf19.1592	29.67	27.4	26.83	27.57	-0.036509131	0.998825	Protein of unknown function; Spider biofilm induced
orf19.6400	orf19.6400	24.24	27.4	32.23	27.33	0.111722428	0.998825	Ortholog(s) have role in inositol metabolic process and cytosol, nuclear envelope localization
orf19.527	orf19.527	35.63	27.38	25.38	27.06	-0.166817446	0.998825	Protein of unknown function; flow model biofilm induced
orf19.4381	VTC3	32.61	27.35	41.36	36.57	0.230916831	0.974208	Putative polyphosphate synthetase; downregulated in core caspofungin response; Hog1p-induced; transcription is opaque-specific; fungal-specific (no human or murine homolog)
orf19.5395	orf19.5395	28.27	27.32	28.29	25.61	-0.023141328	0.998825	Ortholog(s) have role in early endosome to late endosome transport, regulation of protein complex assembly, vacuolar acidification and RAVE complex, extrinsic to membrane localization
orf19.2848	orf19.2848	32.4	27.3	27.8	25.45	-0.097387168	0.998825	Predicted regulatory subunit of the Atg1 signaling complex; required for vesicle formation during autophagy and the cytoplasm-to-vacuole targeting (Cvt) pathway; Spider biofilm induced
orf19.3188	TAC1	30.61	27.29	28.29	22.23	-0.131156388	0.998825	Zn(2)-Cys(6) transcriptional activator of drug-responsive genes (CDR1 and CDR2); binds DRE element; gene in zinc cluster region near MTL locus; resequencing indicates that TAC1 spans orf19.3188 and orf19.3189; Spider biofilm induced
orf19.1260	LEA1	35.49	27.29	34.58	28.3	0.006646245	0.998825	Predicted component of U2 snRNP; induced by alpha pheromone in SpiderM medium

orf19.783	orf19.783	31.14	27.29	29.69	24.28	-0.068282094	0.998825	Ortholog(s) have role in endocytosis, retrograde vesicle-mediated transport, Golgi to ER, vacuole organization and COPI-coated vesicle, Golgi membrane, endosome localization
orf19.1968.1	orf19.1968.1	18.18	27.26	16.62	30.96	0.042350987	NA	Predicted non-catalytic subunit of N-terminal acetyltransferase; Spider biofilm induced
orf19.1835	PEA2	30.15	27.23	25.9	21.04	-0.165710548	0.998825	Putative coiled-coil polarisome; predicted role in polarized morphogenesis, cell fusion, and low affinity Ca2+ influx; rat catheter biofilm induced
orf19.2436	orf19.2436	26.86	27.19	21.03	21.64	-0.187887175	NA	Predicted protein serine/threonine kinase; Spider biofilm induced
orf19.292	orf19.292	19.09	27.17	33.02	27.37	0.203768741	0.998825	Ortholog of C. parapsilosis CDC317 : CPAR2_102390, C. dubliniensis CD36 : Cd36_82960, Candida tenuis NRRL Y-1498 : CANTEDRAFT_133198 and Lodderomyces elongisporus NRLL YB-4239 : LELG_02116
orf19.6722	orf19.6722	34.64	27.17	32.04	29.51	-0.005993549	0.998825	Protein similar to S. cerevisiae Rad4p; down-regulation associated with azole resistance
orf19.2758	PGA38	30.18	27.16	29.14	24.33	-0.069014386	0.998825	Putative adhesin-like GPI-anchored protein; repressed during cell wall regeneration; possibly an essential gene, disruptants not obtained by UAU1 method; rat catheter and Spider biofilm repressed
orf19.3409	SEC12	29.35	27.15	39.9	36.46	0.264915484	0.937109	Putative guanyl-nucleotide exchange factor; induced in high iron; Hap43-repressed
orf19.5505	HIS7	28.82	27.14	26.69	24.52	-0.069189729	0.998825	Putative imidazole glycerol phosphate synthase; histidine biosynthesis; no human/murine homolog; transcription induced by histidine starvation; regulated by Gcn2p and Gcn4p; higher protein level in stationary phase

orf19.318	orf19.318	25.93	27.12	30.22	27.57	0.067352187	0.998825	Ortholog(s) have role in iron-sulfur cluster assembly and mitochondrial matrix localization
orf19.5691	CDC11	29.6	27.11	58.58	45.73	0.567410828	0.340941	Septin; cell and hyphal morphology, agar-invasive growth, full virulence and kidney tissue invasion in mouse, but not kidney colonization, immunogenicity; hyphal and cell-cycle-regulated phosphorylation; rat catheter biofilm repressed
orf19.1323	orf19.1323	35.48	27.1	33.4	36.4	0.090377662	0.998825	Phosphorylated protein of unknown function; Hap43p-repressed gene
orf19.5855	MBP1	29.04	27.08	30.11	29.09	0.037220246	0.998825	Putative component of the MBF transcription complex involved in G1/S cell-cycle progression; non-periodic mRNA expression; predicted, conserved MBF binding sites upstream of G1/S-regulated genes
orf19.3939	orf19.3939	28.53	27.06	30.74	22.63	-0.039596372	0.998825	Ortholog(s) have role in protein folding in endoplasmic reticulum and ER membrane protein complex, cell division site, cytosol, mitotic spindle pole body, nucleus localization
orf19.2499	orf19.2499	27.88	27.04	24.14	24.3	-0.118201246	0.998825	Putative peptidyl-prolyl cis-trans isomerase
orf19.4848	SKI3	31.11	27.04	30.33	25.44	-0.052760011	0.998825	Ortholog(s) have role in negative regulation of G2/M transition of mitotic cell cycle, nuclear-transcribed mRNA catabolic process, 3'-5' exonucleolytic nonsense-mediated decay, nuclear-transcribed mRNA catabolic process, non-stop decay
orf19.5217	TES1	31.25	27.02	14.95	13.05	-0.544827749	NA	Putative acyl-CoA thioesterase
orf19.7442	orf19.7442	33.57	27.02	30.75	26.1	-0.06522582	0.998825	Ortholog of C. dubliniensis CD36 : Cd36_86500, C. parapsilosis CDC317 : CPAR2_206120, Candida tenuis NRRL Y-1498 : CANTEDRAFT_100862 and Debaryomyces hansenii CBS767 : DEHA2F17270g

orf19.3098	orf19.3098	26.87	27.01	25.57	25.16	-0.054791311	0.998825	Predicted RNA-dependent ATPase RNA helicase; Hap43-induced gene
orf19.6605	orf19.6605	45.23	27	40.29	40.27	0.093781961	0.998825	Ortholog(s) have fungal-type vacuole, mitochondrion localization
orf19.5468	orf19.5468	21.74	26.96	22.49	22.14	-0.092460749	NA	Predicted ORF from Assembly 19; removed from Assembly 20; restored based on transcription data; almost identical to orf19.6113, orf19.4055, orf19.5370
orf19.2351	NIT3	34.06	26.95	44.6	42.29	0.322310597	0.8593	Putative nitrilase; regulated by Gcn2p and Gcn4p; protein present in exponential and stationary growth phase yeast cultures
orf19.5205	orf19.5205	32.88	26.94	30.22	23.91	-0.096743487	0.998825	Protein of unknown function; Hap43-repressed gene
orf19.1621	GPA2	25.18	26.9	36.44	30.76	0.208402139	0.998825	G-protein alpha subunit; regulates filamentous growth, copper resistance; involved in cAMP-mediated glucose signaling; reports differ on role in cAMP-PKA pathway, MAP kinase cascade; Gpr1 C terminus binds Gpa2; regulates HWP1 and ECE1
orf19.4678	orf19.4678	28.8	26.9	25.53	23.08	-0.117590714	0.998825	Predicted triglyceride lipase; Spider biofilm induced
orf19.5300	orf19.5300	27.64	26.89	32.34	28.72	0.080416614	0.998825	Calnexin; integral membrane ER chaperone involved in folding and quality control of glycoproteins; caspofungin induced
orf19.4005	orf19.4005	35.31	26.87	42.04	39.13	0.238649346	0.957711	Putative dephospho-CoA kinase; protein likely to be essential for growth, based on an insertional mutagenesis strategy

orf19.109	orf19.109	30.67	26.83	29.23	25.4	-0.067812708	0.998825	Probable mitochondrial tyrosyl-tRNA synthetase, based on conservation in other fungi
orf19.6239	orf19.6239	37.24	26.82	30.77	21.11	-0.182272608	0.998825	Putative serine/threonine protein kinase; possibly an essential gene, disruptants not obtained by UAU1 method
orf19.1075	orf19.1075	25.17	26.81	4.62	5.28	-0.941209211	NA	Protein of unknown function; Spider biofilm induced
orf19.2036	orf19.2036	22.46	26.81	36.3	36.45	0.321703907	0.87024	Predicted dihydrodiol dehydrogenase; ortholog of S. pombe SPAC513.06c; flow model and rat catheter biofilm repressed
orf19.5377	HOS2	30.37	26.8	22.24	23.32	-0.19789964	NA	Putative histone deacetylase; mutations affect filamentous growth; genetic evidence suggests that Set3p and Hos2p function together as a complex involved in regulation of white-opaque switching, morphogenesis, and virulence
orf19.5630	APA2	28.51	26.79	29.45	28.25	0.007727333	0.998825	Putative ATP adenylyltransferase II; regulated by Gcn4; repressed by amino acid starvation (3-AT); induced by prostaglandins; Hap43-repressed; Spider biofilm repressed
orf19.5029	MODF	28.37	26.77	29.31	32.69	0.096270111	0.998825	Ortholog(s) have mitochondrion localization
orf19.2789	orf19.2789	28.53	26.75	23.94	22.95	-0.150080358	0.998825	Ortholog of C. dubliniensis CD36 : Cd36_07040, C. parapsilosis CDC317 : CPAR2_208700, Candida tenuis NRRL Y-1498 : CANTEDRAFT_108836 and Debaryomyces hansenii CBS767 : DEHA2F25674g
orf19.1573	orf19.1573	31.59	26.74	31.64	29.64	0.036291813	0.998825	Ortholog(s) have endoplasmic reticulum, fungal-type vacuole membrane localization

orf19.4519	SUV3	34.53	26.74	24.04	21.22	-0.269766151	0.938404	RNA helicase; mitochondrial RNA catabolism; required for chlamydospore formation, embedded hyphal growth, wild-type respiratory growth, alkaline-induced morphogenesis and SD or Spider biofilm formation; rat catheter biofilm induced
orf19.1499	CTF1	32.72	26.73	31.4	26.66	-0.036171446	0.998825	Putative zinc-finger transcription factor, similar to A. nidulans FarA and FarB; activates genes required for fatty acid degradation; induced by oleate; null mutant displays carbon source utilization defects and slightly reduced virulence
orf19.5247	orf19.5247	34.02	26.71	33.35	29.69	0.021688112	0.998825	Ortholog(s) have cytoplasm localization
orf19.1900	orf19.1900	30.94	26.7	29.84	27	-0.02211734	0.998825	Ortholog(s) have RNA methyltransferase activity, role in 7-methylguanosine cap hypermethylation, meiosis, nucleologenesis, regulation of telomere maintenance via telomerase and nucleolus localization
orf19.748	orf19.748	31.9	26.67	31.55	28.91	0.021809752	0.998825	Ortholog(s) have RNA binding, nucleocytoplasmic transporter activity, structural constituent of nuclear pore activity
orf19.3591	APE3	30.58	26.64	36.11	36.42	0.185886111	0.998825	Putative vacuolar aminopeptidase Y,; regulated by Gcn2 and Gcn4; rat catheter and Spider biofilm repressed
orf19.3271	orf19.3271	31.53	26.61	21.31	24.79	-0.207958371	0.998825	Ortholog of Candida albicans WO-1 : CAWG_01442
orf19.2839	CIRT4B	27.85	26.61	24.44	22.88	-0.135173605	NA	Cirt family transposase; transcript repressed in an azole-resistant strain that overexpresses CDR1 and CDR2; Hap43-repressed; flow model biofilm induced
orf19.1195	orf19.1195	35.64	26.6	31.94	33.02	0.0226184	0.998825	Ortholog(s) have metalloendopeptidase activity, role in cellular iron ion homeostasis, protein processing involved in protein targeting to mitochondrion and mitochondrion localization

orf19.2847	orf19.2847	30.72	26.56	20.49	18	-0.339657331	NA	Ortholog(s) have RNA polymerase III activity, role in tRNA transcription from RNA polymerase III promoter and DNA-directed RNA polymerase III complex, mitotic spindle pole body localization
orf19.1897	orf19.1897	27.48	26.56	24.87	27.21	-0.037980264	0.998825	Ortholog of C. dubliniensis CD36 : Cd36_21640, C. parapsilosis CDC317 : CPAR2_105040, Candida tenuis NRRL Y-1498 : CANTEDRAFT_112352 and Debaryomyces hansenii CBS767 : DEHA2C08008g
orf19.1842	BUD5	28.78	26.54	26.2	22.61	-0.117590714	0.998825	Predicted GTP/GDP exchange factor for Rsr1; rat catheter biofilm induced
orf19.2367	orf19.2367	27.43	26.53	16.32	13.67	-0.440896018	NA	Putative protein of unknown function; Hap43-induced; repressed by Rim101; Spider biofilm induced
orf19.515	orf19.515	18.14	26.53	16.32	14.11	-0.29070852	NA	Has domain(s) with predicted nucleic acid binding, zinc ion binding activity
orf19.4894	orf19.4894	24.19	26.52	40.72	35.26	0.33971717	0.850763	Protein with similarity to S. cerevisiae Yer010cp, a protein of unknown function belonging to the prokaryotic RraA family; repressed by benomyl; Hap43-induced; Spider biofilm induced
orf19.4288	CTA7	33.06	26.5	31.87	28.33	-0.007052307	0.998825	Zn(II)2Cys6 transcription factor; activates transcription in 1-hybrid assay in S. cerevisiae; has similarity to S. cerevisiae Stb4
orf19.2026	UBP13	22.69	26.47	28.16	26.74	0.085752903	0.998825	Ortholog of S. cerevisiae Ubp13; putative ubiquitin carboxyl-terminal hydrolase; flow model biofilm induced; rat catheter biofilm repressed
orf19.3674	GAL102	29.42	26.47	18.58	20.14	-0.274654892	NA	UDP-glucose 4,6-dehydratase; role in mannosylation of cell wall proteins; mutation confers hypersensitivity to toxic ergosterol analog; overlaps orf19.3673; Spider biofilm induced

orf19.5165	orf19.5165	28.8	26.46	29.22	24.73	-0.023068637	0.998825	Ortholog(s) have cytoplasm localization
orf19.5501	YAF9	30.18	26.43	28.11	23.87	-0.069049315	0.998825	Subunit of the NuA4 histone acetyltransferase complex; transcript regulated by Mig1; possibly an essential gene, disruptants not obtained by UAU1 method
orf19.7593	orf19.7593	31.77	26.38	25.94	20.82	-0.179288061	0.998825	Putative asparaginase; predicted role in asparagine catabolism; Spider biofilm induced
orf19.3936	GDE1	30.75	26.37	24.31	19.73	-0.215804906	NA	Glycerophosphocholine phosphodiesterase; mutation confers hypersensitivity to 5-fluorouracil (5-FU); F-12/CO2 early biofilm induced
orf19.4610	orf19.4610	28.13	26.36	33.62	37.42	0.2334534	0.974215	Predicted metallocarboxypeptidase; role in proteolysis; rat catheter biofilm repressed
orf19.2753	ZCF15	29.49	26.36	28.63	22.21	-0.070721356	0.998825	Predicted Zn(II)2Cys6 transcription factor of unknown function; rat catheter biofilm induced
orf19.925	SAM51	33.17	26.33	32.96	30.87	0.064761548	0.998825	Component of the SAM complex involved in mitochondrial protein import, involved in beta-barrel protein assembly; member of the Omp85 protein family
orf19.190	orf19.190	24.01	26.32	29.54	21.23	0.005198516	NA	Ortholog(s) have role in endoplasmic reticulum organization, histone exchange and Swr1 complex, mitochondrion localization
orf19.2104	orf19.2104	30.61	26.31	29	26.24	-0.037027141	0.998825	Ortholog(s) have chaperone binding activity, role in aerobic respiration, iron-sulfur cluster assembly and mitochondrion localization

orf19.7521	REP1	27.28	26.29	16.31	15.68	-0.382069578	NA	Protein involved in negative regulation of MDR1 transcription; mutants show increased resistance to azole drugs
orf19.3093	MSH2	28.82	26.28	34.08	29.43	0.108692947	0.998825	Putative DNA mismatch repair factor; transcript regulated by Nrg1; flucytosine repressed; transcript regulated by tyrosol and cell density
orf19.5804	HYU1	30.82	26.26	37.56	32.54	0.186697382	0.998825	Putative hydantoin utilization protein A; induced upon adherence to polystyrene; regulated by Gcn2p and Gcn4p
orf19.4432	KSP1	20.78	26.26	21.05	23.29	-0.058799726	NA	Putative serine/threonine protein kinase; mRNA binds She3 and is localized to hyphal tips; mutation confers hypersensitivity to amphotericin B
orf19.811	orf19.811	26.09	26.25	25.69	27.65	0.024299738	0.998825	Protein of unknown function; mutant is viable; Hap43-repressed
orf19.426	orf19.426	25.84	26.19	30.08	27.38	0.068296127	0.998825	Ortholog of C. dubliniensis CD36 : Cd36_05120, C. parapsilosis CDC317 : CPAR2_107740, Candida tenuis NRRL Y-1498 : CANTEDRAFT_136277 and Debaryomyces hansenii CBS767 : DEHA2A02266g
orf19.6893	orf19.6893	33.87	26.18	39.79	40.86	0.268046728	0.931333	Ortholog(s) have role in ER to Golgi vesicle-mediated transport, cellular response to cadmium ion, detoxification of cadmium ion
orf19.6118	orf19.6118	28.34	26.15	18.26	23.03	-0.220197277	NA	Putative guanine nucleotide dissociation stimulator; transcription is positively regulated by Tbf1p
orf19.183	HIS3	45.09	26.13	36.66	22.04	-0.164508292	0.998825	Imidazoleglycerol-phosphate dehydratase, enzyme of histidine biosynthesis; functionally complements S. cerevisiae his3-1 mutation; hyphal-induced expression; regulated by Gcn2p and Gcn4p; fungal-specific (no human or murine homolog)

orf19.4286	orf19.4286	25.73	26.13	20.47	18.64	-0.229694769	NA	Protein of unknown function; flow model biofilm induced; Spider biofilm induced
orf19.3448	orf19.3448	35.65	26.13	11.85	14.21	-0.640947176	NA	Protein of unknown function; ketoconazole-repressed
orf19.7370	orf19.7370	30.11	26.12	20.96	19.44	-0.272234135	NA	Possible G-protein coupled receptor; vacuolar membrane transporter for cationic amino acids; PQ-loop motif; rat catheter and Spider biofilm induced
orf19.2002	orf19.2002	33.46	26.11	32.42	30.36	0.036840725	0.998825	Ortholog(s) have structural constituent of nuclear pore activity and role in nuclear pore organization, poly(A)+ mRNA export from nucleus, protein import into nucleus, ribosomal large subunit export from nucleus
orf19.4439.1	orf19.4439.1	27.17	26.11	26.05	26.04	-0.022782521	0.998825	Ortholog(s) have role in nucleotide-excision repair, phosphorylation of RNA polymerase II C-terminal domain, transcription from RNA polymerase II promoter and core TFIIH complex, cytosol, holo TFIIH complex localization
orf19.2165	orf19.2165	32.91	26.09	25.35	23.53	-0.159639016	0.998825	Predicted hydrolase; induced by nitric oxide
orf19.2307	orf19.2307	27.34	26.08	31.13	26	0.052287167	0.998825	Ortholog(s) have role in mRNA cleavage, mRNA polyadenylation, mitotic sister chromatid segregation, regulation of G1/S transition of mitotic cell cycle and cytosol, mRNA cleavage and polyadenylation specificity factor complex localization
orf19.5335	SGS1	28.04	26.07	27.09	18.92	-0.137541859	NA	RecQ-related DNA helicase; Bloom's syndrome-related gene; haploinsufficient for wild-type lifespan; upregulated in farnesol treated biofilm
orf19.7183	orf19.7183	35.99	26.07	30.04	24.16	-0.123483934	0.998825	Ortholog(s) have role in protein folding in endoplasmic reticulum and ER membrane protein complex localization

orf19.7361	orf19.7361	33.78	26.02	30.55	21.75	-0.111660888	0.998825	Ortholog(s) have tRNA-intron endonuclease activity, role in tRNA-type intron splice site recognition and cleavage and mitochondrial outer membrane, tRNA-intron endonuclease complex localization
orf19.3043	orf19.3043	23.88	26.01	29.69	28.25	0.114583676	0.998825	Ortholog(s) have triglyceride lipase activity, role in triglyceride catabolic process and mitochondrion localization
orf19.4731	orf19.4731	29.66	25.99	41.67	37.68	0.314220045	0.870631	Ortholog(s) have role in CVT pathway, intra-Golgi vesicle-mediated transport and Golgi transport complex localization
orf19.3365	DAO2	25.28	25.92	20.68	18.74	-0.19583719	NA	Putative D-amino acid oxidase; rat catheter biofilm induced
orf19.6942	ORC3	32.78	25.91	32.07	31.96	0.065283484	0.998825	Protein similar to S. cerevisiae Orc3p, which is a component of the origin recognition complex involved in DNA replication; mutation confers hypersensitivity to toxic ergosterol analog; induced under hydroxyurea treatment
orf19.1656	orf19.1656	31.69	25.9	37.17	37.67	0.224350375	0.987362	Protein with a predicted FYVE/PHD zinc finger domain; Hap43-repressed; Spider biofilm induced
orf19.267	orf19.267	28.59	25.85	29.57	26.54	0.022663402	0.998825	Protein required for normal filamentous growth; mRNA binds She3
orf19.3421.1	MED19	30.11	25.85	20.4	20.39	-0.271530741	NA	RNA polymerase II mediator complex subunit
orf19.6626	orf19.6626	30.45	25.83	38.35	36.8	0.253702676	0.946319	Predicted homeodomain-containing protein; possible transcription factor; possibly transcriptionally regulated upon hyphal formation

orf19.3629	DSE1	26.18	25.83	17.55	19.91	-0.246882034	NA	Essential cell wall protein involved in cell wall integrity and rigidity; periodic mRNA expression peaks at M/G1 phase; Ace2p-induced; required for virulence in a mouse model of infection
orf19.1112	BUD7	27.85	25.82	32.43	32.33	0.139173323	0.998825	Ortholog(s) have role in Golgi to plasma membrane transport, fungal-type cell wall chitin biosynthetic process and cell division site, clathrin-coated vesicle, cytosol, exomer complex, nucleus, ribosome localization
orf19.5692	orf19.5692	28.61	25.81	44.78	39.54	0.387885264	0.743771	Ortholog(s) have ubiquitin-protein ligase activity, role in anaphase-promoting complex-dependent proteasomal ubiquitin-dependent protein catabolic process, protein ubiquitination and anaphase-promoting complex localization
orf19.2191	orf19.2191	29.03	25.76	26.06	22.43	-0.118687528	0.998825	Ortholog(s) have role in endocytic recycling, peptidyl-diphthamide biosynthetic process from peptidyl-histidine and cytosol, endosome, nucleus localization
orf19.2891	orf19.2891	36.91	25.76	32.72	21.46	-0.137163011	0.998825	Ortholog(s) have role in cellular response to oxidative stress, misfolded or incompletely synthesized protein catabolic process, protein import into peroxisome matrix and mitochondrial inner membrane localization
orf19.440	SDH1	22.42	25.7	27.18	24.09	0.038959775	NA	Putative mitochondrial succinate dehydrogenase; macrophage/pseudohyphal-induced; repressed by nitric oxide
orf19.1981	IMP2	29.3	25.68	26.82	36.41	0.11137124	0.998825	Predicted subunit of the mitochondrial inner membrane peptidase complex involved in protein targeting to mitochondria
orf19.4203	orf19.4203	35.93	25.68	32.49	30.44	-0.005368718	0.998825	Ortholog(s) have role in nuclear pore organization, spindle pole body duplication and nuclear pore transmembrane ring, spindle pole body localization
orf19.2423	ZCF11	28.43	25.66	30.66	29.77	0.096685802	0.998825	Zn(II)2Cys6 transcription factor; required for wild-type filamentous growth; mRNA binds She3

orf19.7460	orf19.7460	30.98	25.66	19.84	18.93	-0.305045744	NA	Putative 2'-O-methyltransferase with a predicted role in tRNA modification; transcription is activated in the presence of elevated CO2
orf19.1585	ZRT2	21.61	25.65	36.03	39.04	0.377279322	0.788835	Zinc transporter; ciclopirox olamine, fluconazole, alkaline repressed; transcript induced by amphotericin B, interaction with macrophages; possibly essential (UAU1 method); induced in oralpharyngeal candidiasis; Spider biofilm induced
orf19.1289	SCT1	25.31	25.62	33.44	36.69	0.266486452	0.938404	Putative glycerol-3-phosphate O-acyltransferase; fungal-specific (no human or murine homolog)
orf19.2677	orf19.2677	19.47	25.62	33.29	26.74	0.221536662	0.998825	Putative GPI transamidase component; possibly an essential gene, disruptants not obtained by UAU1 method
orf19.362	TLO9	36.22	25.61	58.5	86.8	0.836824238	0.03959	Member of a family of telomere-proximal genes of unknown function; Hap43p-repressed gene
orf19.4723	FAD1	37.43	25.61	32.22	32.2	0.009224533	0.998825	Ortholog(s) have ATP binding, FMN adenylyltransferase activity, magnesium ion binding activity, role in FAD biosynthetic process and cytosol, nucleus localization
orf19.3804	orf19.3804	22.35	25.57	25.02	25.5	0.023934468	NA	Protein of unknown function; filament induced
orf19.1527	orf19.1527	26.97	25.56	22.71	33.52	0.055996528	0.998825	Protein of unknown function; Spider biofilm induced
orf19.3180	orf19.3180	28.26	25.54	28.32	24.52	-0.023234667	0.998825	Ortholog(s) have phosphatidylinositol-4,5-bisphosphate 5-phosphatase activity, role in phosphatidylinositol dephosphorylation and endoplasmic reticulum localization

orf19.1871	orf19.1871	30.11	25.53	30.4	26.91	0.007780327	0.998825	Ortholog(s) have structural molecule activity, role in histone exchange and Swr1 complex, cytosol localization
orf19.4435	orf19.4435	22.33	25.51	24	23.57	-0.008536737	NA	Ortholog(s) have structural constituent of cytoskeleton activity, role in microtubule nucleation and spindle pole body localization
orf19.2284	orf19.2284	22.91	25.49	26.72	24.13	0.039763172	NA	Protein with an FMN-binding domain; Hap43-repressed; flow model biofilm induced
orf19.731	EMP46	30.17	25.49	35.41	36.99	0.231519086	0.976613	Protein similar to S. cerevisiae Emp46, an integral membrane component of ER-derived COPII-coated vesicles; functions in ER to Golgi transport; induced by alpha pheromone in SpiderM medium
orf19.2410	SYS3	23.24	25.49	30.49	28.43	0.147282096	0.998825	Protein similar to S. cerevisiae Sys3p; putative role in endosome-Golgi vesicle docking; upregulated in biofilm; induced upon adherence to polystyrene
orf19.28	orf19.28	30.51	25.49	24.59	20.41	-0.183128973	NA	Putative thiamine transmembrane transporter; Spider biofilm induced
orf19.4863	PDC2	27.84	25.48	27.11	24.16	-0.038868147	0.998825	Homeodomain-like transcription factor; regulator of pyruvate decarboxylase; contains a putative C-terminal activation domain, Glu- and Pro-rich; complements glucose utilization defect of S. cerevisiae pdc2 mutant
orf19.3087.1	orf19.3087.1	24.59	25.46	35.04	36.77	0.309033977	0.880321	Ortholog of C. dubliniensis CD36 : Cd36_54140 and Candida albicans WO-1 : CAWG_04820
orf19.4426	PEX3	33.79	25.46	33.29	33.55	0.106663609	0.998825	Putative peroxisomal protein involved in targeting proteins into peroxisomes; possibly an essential gene, disruptants not obtained by UAU1 method

orf19.2675	orf19.2675	28.43	25.45	26.12	20.19	-0.121534478	NA	Ortholog(s) have mRNA binding activity, role in spliceosomal complex assembly and U2 snRNP, U2-type prespliceosome localization
orf19.6197	DHH1	22.42	25.45	24.71	24.93	0.040916392	NA	Putative RNA helicase
orf19.5516	orf19.5516	25.93	25.44	28.09	26.04	0.039381273	0.998825	Ortholog(s) have signal sequence binding activity, role in protein targeting to ER and cytosol, nucleus, signal recognition particle, endoplasmic reticulum targeting localization
orf19.3211	RCF3	26.51	25.42	27.16	26.43	0.008622929	0.998825	Putative heteropentameric replication factor C subunit; transcription is induced upon filamentous growth
orf19.3785	orf19.3785	30.41	25.41	28.52	28.28	0.023860486	0.998825	DnaJ chaperone domain protein; role in pre-mRNA splicing; Spider biofilm induced
orf19.2137	orf19.2137	28.63	25.41	23.94	25.34	-0.085005883	0.998825	Ortholog(s) have mitochondrion localization
orf19.2261	orf19.2261	27.86	25.4	29	25.09	0.007818999	0.998825	Ortholog(s) have RNA binding activity, role in mRNA splicing, via spliceosome and U2 snRNP, U2-type prespliceosome, cytosol localization
orf19.6477	orf19.6477	41.91	25.4	30	24.45	-0.187425328	0.998825	Ortholog(s) have tRNA (guanine-N7-)-methyltransferase activity, role in tRNA methylation and cytosol, nucleus, tRNA methyltransferase complex localization
orf19.4522	orf19.4522	25.4	25.39	22.22	19.99	-0.143376102	NA	Ortholog(s) have role in response to drug and cytosol, nucleus localization

orf19.775	orf19.775	24.91	25.38	26.46	24.57	0.008421704	NA	Ortholog of C. dubliniensis CD36 : Cd36_04450, C. parapsilosis CDC317 : CPAR2_105460, Debaryomyces hansenii CBS767 : DEHA2D07128g and Pichia stipitis Pignal : PICST_80203
orf19.2335	orf19.2335	21.49	25.36	26.29	26.28	0.089000879	NA	Putative aspartyl aminopeptidase; stationary phase enriched protein; mutation confers hypersensitivity to 5-fluorouracil (5-FU); Hog1-induced; planktonic growth-induced; rat catheter and Spider biofilm repressed
orf19.7343	orf19.7343	28.74	25.34	25.96	21.62	-0.102938166	NA	Ortholog(s) have role in mRNA splicing, via spliceosome and U4/U6 x U5 tri-snRNP complex localization
orf19.5251	ZCF30	30.22	25.29	19.86	18.32	-0.293613066	NA	Predicted Zn(II)2Cys6 transcription factor; mutants are viable; repressed by fluphenazine; Spider biofilm induced
orf19.7646	orf19.7646	28.29	25.27	22.61	19.4	-0.190578613	NA	Ortholog(s) have role in box C/D snoRNP assembly, protein folding, rRNA processing, regulation of cell size and R2TP complex, cytoplasm, nucleus, small nucleolar ribonucleoprotein complex localization
orf19.2304	orf19.2304	33.87	25.26	58.61	53.11	0.608073845	0.261384	Protein similar to S. cerevisiae Gvp36p; transposon mutation affects filamentous growth
orf19.135	EXO84	30.38	25.24	33.47	26.02	0.051441241	0.998825	Predicted subunit of the exocyst complex, involved in exocytosis; localizes to a crescent on the surface of the hyphal tip
orf19.113	CIP1	29.46	25.24	28.54	20.35	-0.087441555	0.998825	Possible oxidoreductase; transcript induced by cadmium but not other heavy metals, heat shock, yeast-hypha switch, oxidative stress (via Cap1), or macrophage interaction; stationary phase enriched protein; Spider biofilm induced
orf19.5601	orf19.5601	25.56	25.24	29.61	31.73	0.158414645	0.998825	Ortholog(s) have extracellular region localization

orf19.1263	CFL1	28.94	25.23	24.8	23.84	-0.085696965	0.998825	Protein similar to ferric reductase Fre10p; possible functional homolog of S. cerevisiae Fre1p (reports differ); transcription is negatively regulated by Sfu1p, copper, amphotericin B, caspofungin; induced by ciclopirox olamine
orf19.4909	CBK1	27.65	25.21	31.52	29.66	0.126490602	0.998825	Ser/Thr kinase of cell wall integrity pathway; mutants show abnormal morphology and aggregation; Mob2p associated; required for wild-type hyphal growth and transcriptional regulation of cell-wall-associated genes
orf19.5213	orf19.5213	26.72	25.2	25.52	21.29	-0.089250133	NA	Putative protein of unknown function; transcript is upregulated in clinical isolates from HIV+ patients with oral candidiasis
orf19.3686	orf19.3686	25.51	25.19	28.52	27.43	0.069860627	0.998825	Ortholog(s) have protein domain specific binding activity, role in mitochondrial proton-transporting ATP synthase complex assembly and mitochondrion localization
orf19.757	orf19.757	25.6	25.16	31.61	24.31	0.067742699	0.998825	Ortholog of S. cerevisiae : YPR089W, C. glabrata CBS138 : CAGL0K08008g, C. dubliniensis CD36 : Cd36_04600, C. parapsilosis CDC317 : CPAR2_105630 and Candida tenuis NRRL Y-1498 : CANTEDRAFT_129373
orf19.7254	orf19.7254	25.56	25.14	24.26	21.57	-0.089726334	NA	Ortholog(s) have role in mRNA metabolic process, mitochondrial translational initiation and extrinsic to membrane, mitochondrial inner membrane localization
orf19.588	orf19.588	32.88	25.13	16.72	10.93	-0.538565066	NA	Ortholog(s) have role in aerobic respiration and mitochondrial intermembrane space, nucleus localization
orf19.5875	VAM3	31.07	25.11	28.57	29	0.024052507	0.998825	Predicted target membrane receptor (t-SNARE) involved in vacuolar transport, endocytosis, and secretion; required for biofilm formation in RPMI-1640 and for virulence; rat catheter biofilm repressed
orf19.1574	orf19.1574	29.62	25.09	28.28	27.33	-0.006230265	0.998825	Putative TFIID subunit involved in RNA polymerase II transcription initiation; possibly an essential gene, disruptants not obtained by UAU1 method

orf19.1456	orf19.1456	25.06	25.05	27.72	23.62	0.023327151	NA	Ortholog of Candida albicans WO-1 : CAWG_03922
orf19.212	VPS28	28.38	25.05	23.46	21.41	-0.154968506	NA	ESCRT I protein sorting complex subunit; involved in Rim8 processing and proteolytic activation of Rim101, which regulates pH response; role in echinocandin, azole sensitivity
orf19.6800	POS5	29.29	25.01	33.94	34.64	0.207552847	0.998825	Protein similar to S. cerevisiae Pos5p, a mitochondrial NADH kinase involved in the oxidative stress response; planktonic growth-induced gene; likely to be essential for growth, based on an insertional mutagenesis strategy
orf19.4315	orf19.4315	25.41	24.99	20.42	20.08	-0.178783752	NA	Ortholog(s) have Rab GTPase activator activity, role in vesicle-mediated transport and intracellular localization
orf19.4398	orf19.4398	34.27	24.97	22.59	14.28	-0.367399917	NA	Protein of unknown function; rat catheter biofilm induced
orf19.5035	HPC2	30.92	24.97	23.68	25.01	-0.114745152	0.998825	Ortholog(s) have DNA binding, nucleosome binding activity
orf19.5067	orf19.5067	35.92	24.94	22.43	22.96	-0.251843597	0.951606	Predicted nuclear exosome-associated nucleic acid binding protein; rat catheter and Spider biofilm induced
orf19.4516	orf19.4516	28.92	24.93	33.4	29.18	0.110589814	0.998825	Ortholog(s) have tetrahydrofolylpolyglutamate synthase activity, role in one-carbon metabolic process, regulation of DNA methylation and cytosol, mitochondrion, nucleus localization
orf19.2911	SEC3	28.32	24.88	35.53	24.97	0.108690972	0.998825	Predicted subunit of the exocyst complex, involved in exocytosis; required for hyphal growth after the first septin ring formation; interacts with septins Cdc3p, Cdc10p, and Cdc11p; localizes to a crescent on the surface of the hyphal tip

orf19.4420	orf19.4420	28.19	24.88	23.21	21.21	-0.154968506	NA	Predicted component of the core factor rDNA transcription factor complex; required for transcription of 35S rRNA genes by RNA polymerase I in S. cerevisiae; possibly an essential gene, disruptants not obtained by UAU1 method
orf19.6875	VPS35	27.86	24.87	25	23.76	-0.070514064	NA	Putative role in vacuolar sorting; downregulated in biofilm; induced upon adherence to polystyrene
orf19.3544	orf19.3544	35.25	24.84	35.83	31.79	0.104350489	0.998825	Putative protein of unknown function; Hap43p-repressed gene
orf19.5584	PEP3	26.18	24.83	24.64	22.03	-0.073197823	NA	Peptidase; activity useful for strain identification by multilocus enzyme electrophoresis (MLEE); clade-associated gene expression
orf19.2961	orf19.2961	28.89	24.8	25.37	24.57	-0.069158886	0.998825	Putative transcription factor with zinc finger DNA-binding motif
orf19.6829	orf19.6829	33.93	24.78	30.64	27.15	-0.020618796	0.998825	Protein with a predicted mitochondrial ATPase expression domain; possibly an essential gene, disruptants not obtained by UAU1 method
orf19.854	UGA11	28.51	24.78	50.44	45.7	0.525724771	0.432569	Putative gamma-aminobutyrate (GABA) transaminase; macrophage-induced; overlaps orf19.854.1, which is a region annotated as a blocked reading frame; Spider biofilm induced
orf19.2888	orf19.2888	34.71	24.74	30.93	27.79	-0.019914362	0.998825	Ortholog(s) have role in early endosome to Golgi transport, protein complex assembly and Golgi apparatus, TRAPP complex, clathrin-coated vesicle localization
orf19.5009	orf19.5009	30.54	24.73	25.16	19.46	-0.200352844	NA	Ortholog(s) have cytoplasm localization

orf19.3007	orf19.3007	32.98	24.73	26.31	30.82	-0.018367547	0.998825	Ortholog(s) have role in endosome organization, regulation of protein localization and BLOC-1 complex localization
orf19.2890	orf19.2890	28.93	24.66	25.82	26.11	-0.037316356	0.998825	Has domain(s) with predicted sugar:hydrogen symporter activity, role in carbohydrate transport and Golgi membrane, integral to membrane localization
orf19.6152	orf19.6152	33.59	24.65	32.21	24.94	-0.036110858	0.998825	Ortholog(s) have mitochondrion localization
orf19.5475	orf19.5475	27.42	24.65	45.04	43.64	0.481373191	0.543746	Sef1p-, Sfu1p-, and Hap43p-regulated gene
orf19.6550	orf19.6550	29.77	24.62	26.27	22.84	-0.101262289	0.998825	Putative mitochondrial outer membrane protein; regulated by Sef1p-, Sfu1p-, and Hap43p
orf19.2404	orf19.2404	28.72	24.61	24.71	21	-0.136334898	NA	Ortholog(s) have RNA binding, ribonuclease MRP activity, ribonuclease P activity and role in intronic box C/D snoRNA processing, mRNA cleavage, rRNA processing, tRNA processing
orf19.5623	ARP4	33.34	24.6	35.89	40.49	0.237084045	0.960406	Subunit of the NuA4 histone acetyltransferase complex
orf19.1604	orf19.1604	25.1	24.59	22.68	20.63	-0.108218898	NA	Predicted Gal4-like DNA-binding transcription factor; Spider biofilm induced
orf19.7016	PHM5	28.95	24.57	30.27	28.21	0.053451272	0.998825	Ortholog(s) have endopolyphosphatase activity, exopolyphosphatase activity, role in polyphosphate catabolic process and fungal-type vacuole membrane, nucleus localization

orf19.4306	orf19.4306	31.59	24.56	35	24.49	0.020635042	0.998825	Ortholog(s) have methylthioribulose 1-phosphate dehydratase activity, role in L-methionine salvage from methylthioadenosine and cytoplasm localization
orf19.1244	GYP2	29.03	24.53	37.2	30.37	0.178445764	0.998825	Ortholog(s) have Rab GTPase activator activity, role in intracellular protein transport and cell division site, cytosol localization
orf19.5243	TRP3	22.74	24.52	25.41	21.36	-0.042463949	NA	Putative bifunctional enzyme with predicted indole-3-glycerol-phosphate synthase and anthranilate synthase activities; regulated by Gcn2p and Gcn4p
orf19.4767	ZCF28	33.78	24.49	21.86	23.91	-0.19149847	0.998825	Zn(II)2Cys6 transcription factor; required for yeast cell adherence to silicone substrate; Spider biofilm induced
orf19.206	orf19.206	25.07	24.48	28.5	24.99	0.071087458	0.998825	Putative adhesin-like protein
orf19.6985	TEA1	20.72	24.46	20.67	20.73	-0.060226217	NA	Putative transcription factor with zinc cluster DNA-binding motif; has similarity to S. cerevisiae Tea1p; Hap43p-repressed gene
orf19.1849	orf19.1849	30.13	24.45	25.25	22.42	-0.118429734	NA	Ortholog(s) have U3 snoRNA binding, rRNA binding activity, role in maturation of SSU-rRNA from tricistronic rRNA transcript (SSU-rRNA, 5.8S rRNA, LSU-rRNA) and nucleolus, small-subunit processome localization
orf19.5796	SHE9	33.31	24.44	32.99	30.85	0.094633218	0.998825	Protein with similarity to S. cerevisiae She9p, which inhibits growth when overproduced; gene has possible growth-regulation element; fungal-specific (no human or murine homolog)
orf19.3359	ARP8	28.26	24.4	28.13	29.95	0.085734194	0.998825	Putative chromatin-remodeling enzyme complex protein; mutation confers hypersensitivity to toxic ergosterol analog, and to amphotericin B; rat catheter biofilm induced

orf19.6347	orf19.6347	23.93	24.4	16.15	16.26	-0.301065333	NA	Pre-mRNA splicing factor; important for catalytic step II of pre-mRNA splicing; possible role in cell cycle progression; Spider biofilm induced
orf19.2995	orf19.2995	28.16	24.38	27.34	22.31	-0.055564721	NA	Protein of unknown function; induced by nitric oxide; rat catheter and Spider biofilm induced
orf19.5442	orf19.5442	25.01	24.38	32.27	26.3	0.130623973	0.998825	Ortholog of C. dubliniensis CD36 : Cd36_80240, C. parapsilosis CDC317 : CPAR2_504040, Candida tenuis NRRL Y-1498 : CANTEDRAFT_130872 and Debaryomyces hansenii CBS767 : DEHA2C06006g
orf19.5619	orf19.5619	26.63	24.35	42.24	40.04	0.416813804	0.684155	Protein of unknown function; induced by alpha pheromone in SpiderM medium; Spider biofilm induced
orf19.6235	orf19.6235	29.01	24.34	29.44	29.95	0.084708718	0.998825	Protein of unknown function; Spider biofilm induced
orf19.4951	orf19.4951	24.33	24.32	19.12	22.19	-0.126429614	NA	Protein of unknown function; Spider biofilm induced
orf19.3635	orf19.3635	25.97	24.32	31.5	26.27	0.100270142	0.998825	Ortholog of C. dubliniensis CD36 : Cd36_22580, C. parapsilosis CDC317 : CPAR2_406960, Candida tenuis NRRL Y-1498 : CANTEDRAFT_113677 and Debaryomyces hansenii CBS767 : DEHA2D03608g
orf19.6673	HEX1	21.45	24.3	26.87	27.08	0.137594475	NA	Beta-N-acetylhexosaminidase/chitobiase, highly glycosylated enzyme that is secreted to the periplasm and culture medium; required for full virulence; may have role in carbon or nitrogen scavenging; possibly an essential gene (UAU1 method)
orf19.4808	NUP188	24.82	24.29	28.13	24.04	0.039928627	NA	Putative nuclear pore complex subunit; transcript regulated by Nrg1 and Mig1

orf19.6411	orf19.6411	24.82	24.26	35	31.13	0.244930838	0.957711	Protein similar to S. cerevisiae Vac14p; a protein involved in regulated synthesis of PtdIns(3,5)P(2); transposon mutation affects filamentous growth
orf19.4033	PRP22	24.86	24.23	25.16	21.22	-0.057939954	NA	Putative RNA-dependent ATPase; induced upon adherence to polystyrene; induced by Mnl1p under weak acid stress
orf19.1446	CLB2	25.2	24.18	22.87	20.47	-0.109366406	NA	B-type mitotic cyclin (cyclin-dependent protein kinase regulatory subunit); essential; required for wild-type mitotic exit; role in cell polarization; interacts with catalytic subunit Cdk1; Spider biofilm repressed
orf19.3714	orf19.3714	28.49	24.17	29.32	26.7	0.054907875	0.998825	Ortholog of C. dubliniensis CD36 : Cd36_33530, C. parapsilosis CDC317 : CPAR2_201980, Candida tenuis NRRL Y-1498 : CANTEDRAFT_134293 and Debaryomyces hansenii CBS767 : DEHA2A10164g
orf19.7096	orf19.7096	23.24	24.17	23.17	23.29	-0.024254627	NA	Ortholog(s) have fungal-type vacuole membrane, mitochondrion localization
orf19.3872	orf19.3872	18.83	24.14	47.44	36.34	0.553647377	0.405369	Protein of unknown function; oral infection induced; mutants have reduced capacity to damage oral epithelial cells; rat catheter biofilm repressed
orf19.6630	orf19.6630	25.13	24.13	35.87	24.07	0.156535554	0.998825	Ortholog(s) have role in ER-associated ubiquitin-dependent protein catabolic process
orf19.5694	orf19.5694	22.76	24.12	30.9	25.42	0.133076261	0.998825	Predicted ORF from Assembly 19; removed from Assembly 20; restored based on transcription data
orf19.7245	orf19.7245	26.33	24.11	20.98	18.18	-0.197510637	NA	Ortholog(s) have tRNA (5-methylaminomethyl-2-thiouridylate)-methyltransferase activity, role in mitochondrial tRNA thio-modification and mitochondrion localization

orf19.7072	PEL1	31.41	24.11	25.53	21.94	-0.116983648	0.998825	Predicted enzyme of mitochondrial phospholipid biosynthesis; rat catheter and flow model biofilm induced
orf19.4927	BNI1	29.75	24.11	36	31.8	0.193341012	0.998825	Formin; role in cytoskeletal organization, cell polarity; role in systemic virulence in mouse; cell-cycle regulated localization to site of polarized growth, bud neck; localizes to Spitzenkorper of hyphae, minor localization at septum
orf19.2046	POT1-2	34.27	24.1	37.18	28.46	0.091357127	0.998825	Putative peroxisomal 3-ketoacyl CoA thiolase; Hap43-repressed
orf19.2209	YVC1	23.82	24.09	36.68	31.21	0.287232914	0.914829	Putative vacuolar cation channel shock; repressed by alpha pheromone in SpiderM medium; flow model biofilm repressed
orf19.4361	IFF3	24.06	24.08	30.12	28.6	0.163159422	0.998825	Putative GPI-anchored protein
orf19.6432	orf19.6432	29.62	24.07	24.5	20.16	-0.152574244	NA	Ortholog(s) have ATPase activity, role in response to drug, ribosomal large subunit biogenesis and cytosol, preribosome, large subunit precursor localization
orf19.2808	ZCF16	28.28	24.07	23.96	20.49	-0.139291867	NA	Predicted Zn(II)2Cys6 transcription factor; mutants are viable; rat catheter biofilm induced
orf19.3568	RXT3	24.26	24.02	24.08	23.12	-0.024099761	NA	Putative transcriptional repressor
orf19.6023	orf19.6023	29.89	24.01	23.33	22.19	-0.151636199	NA	Protein with a predicted multidrug transporter domain; Hap43-repressed gene

orf19.2309	PET127	29.95	24	24.3	20.46	-0.169614357	NA	Protein with a predicted role in 5'-end processing of mitochondrial RNAs; ortholog of S. cerevisiae Pet127; Hap43-induced; rat catheter and Spider biofilm induced
orf19.988	orf19.988	25.67	23.99	23.02	23.62	-0.055477945	NA	Protein of unknown function; oral infection upregulated gene; upregulated in strains from HIV+ patients with oral candidiasis; mutants have reduced ability to damage oral epithelial cells; transcript possibly regulated upon hyphal formation
orf19.7058	orf19.7058	29.21	23.92	30.48	25.51	0.038879341	0.998825	Ortholog(s) have role in cellular response to cadmium ion, detoxification of cadmium ion and cytoplasm, nucleus localization
orf19.516	orf19.516	22.81	23.9	30.99	26.69	0.163739723	0.998825	S. cerevisiae ortholog Rft1p has role in glycolipid translocation, protein N-linked glycosylation and localizes to endoplasmic reticulum membrane; regulated by Sef1p-, Sfu1p-, and Hap43p
orf19.7434	GLG2	21.3	23.89	50.25	39.35	0.590494298	0.325518	Putative self-glucosylating initiator of glycogen synthesis; expression regulated upon white-opaque switch; hypha-induced; Spider biofilm induced
orf19.1196	PKH3	29.02	23.88	34.06	28.5	0.14007153	0.998825	Ortholog(s) have protein kinase activity and role in MAPK cascade involved in cell wall biogenesis, protein phosphorylation
orf19.4747	HEM14	27.5	23.77	30.34	30.9	0.144646442	0.998825	Putative protoporphyrinogen oxidase; involved in heme biosynthesis; predicted Kex2p substrate; iron regulated transcript; Yfh1-induced; Hap43-repressed; rat catheter biofilm repressed
orf19.7479	NTH1	26.58	23.73	26.98	25.15	0.008888592	0.998825	Neutral trehalase; hyphal induction in mutant delayed but not reduced overall; not required for virulence in mice; possible regulatory cAMP-dependent phosphorylation at S10,S213; Hap43-repressed gene; Spider biofilm induced
orf19.5702	orf19.5702	25.78	23.68	26.4	25.17	0.009101934	NA	Ortholog(s) have cell division site, spindle localization

orf19.2318.1	orf19.2318.1	31.4	23.68	26.37	19.77	-0.150565384	NA	Ortholog(s) have inositol phosphoceramide synthase regulator activity, role in inositolphosphoceramide metabolic process and inositol phosphoceramide synthase complex, integral to Golgi membrane localization
orf19.4122	orf19.4122	33.48	23.65	16.69	16.11	-0.418517277	NA	Ortholog(s) have acyl-CoA hydrolase activity, role in fatty acid beta-oxidation and mitochondrion, peroxisome localization
orf19.5731	PAD1	30.02	23.62	31.33	30.48	0.097741734	0.998825	Putative phenylacrylic acid decarboxylase; repressed by Rgt1p
orf19.7074	orf19.7074	24.1	23.61	24.27	23.31	-0.024099761	NA	Ortholog(s) have RNA polymerase II transcription factor recruiting transcription factor activity, methylated histone residue binding activity
orf19.3061	IMP1	35.59	23.6	32.68	28.8	0.023105657	0.998825	Predicted subunit of the mitochondrial inner membrane peptidase complex involved in protein targeting to mitochondria
orf19.2980	orf19.2980	26.74	23.59	25.82	22.25	-0.05617663	NA	Ortholog(s) have ATP-dependent RNA helicase activity and role in generation of catalytic spliceosome for first transesterification step, spliceosomal complex disassembly
orf19.6522	orf19.6522	24.46	23.59	22.36	19.89	-0.110610806	NA	Putative allantoate permease; Gcn4-regulated; Spider biofilm induced
orf19.529	orf19.529	29.22	23.57	26.29	25.04	-0.037560362	0.998825	Ortholog of C. dubliniensis CD36 : Cd36_29750, C. parapsilosis CDC317 : CPAR2_205260, Candida tenuis NRRL Y-1498 : CANTEDRAFT_91782 and Debaryomyces hansenii CBS767 : DEHA2F19294g
orf19.352	orf19.352	26.09	23.57	24.77	21.23	-0.073856067	NA	Ortholog of C. dubliniensis CD36 : Cd36_83550, C. parapsilosis CDC317 : CPAR2_404510, Debaryomyces hansenii CBS767 : DEHA2B13794g and Pichia stipitis Pignal : PICST_30078

orf19.642	orf19.642	28.01	23.55	29.86	24.48	0.023204125	0.998825	Ortholog of C. dubliniensis CD36 : Cd36_30470, C. parapsilosis CDC317 : CPAR2_203380, Candida tenuis NRRL Y-1498 : CANTEDRAFT_117493 and Debaryomyces hansenii CBS767 : DEHA2G19228g
orf19.3213	orf19.3213	28.59	23.52	31.04	25.16	0.0382296	0.998825	Protein of unknown function; Spider biofilm induced
orf19.136	QDR3	24.74	23.49	15.24	17.26	-0.299068136	NA	Predicted membrane transporter, member of the drug:proton antiporter (12 spanner) (DHA1) family, major facilitator superfamily (MFS); Hap43p-repressed gene
orf19.6699	orf19.6699	27.83	23.48	37.6	35.03	0.306041826	0.882407	Ortholog(s) have histidinol-phosphatase activity, role in histidine biosynthetic process and cytosol, nucleus localization
orf19.4820	orf19.4820	22.8	23.48	26.85	24.1	0.074028376	NA	Ortholog(s) have mitochondrion localization
orf19.3259	orf19.3259	21.89	23.45	26.13	20.27	0.007654088	NA	Ortholog(s) have peptidase activity, role in protein targeting to ER, signal peptide processing and signal peptidase complex localization
orf19.7538	orf19.7538	29.15	23.45	28.58	22.29	-0.023231058	0.998825	DNA helicase involved in rDNA replication; Spider biofilm repressed
orf19.1259	orf19.1259	25.86	23.44	25.2	26.53	0.04240191	NA	Ortholog(s) have DNA binding, ubiquitin-protein ligase activity and role in histone catabolic process, histone ubiquitination, regulation of transcription from RNA polymerase II promoter in response to oxidative stress
orf19.3666	orf19.3666	25.29	23.41	22.6	15.48	-0.183378354	NA	Predicted debranching enzyme-associated ribonuclease; rat catheter biofilm induced

orf19.2133	LIP4	21.51	23.39	24.01	22.92	0.026112738	NA	Secreted lipase, member of a differentially expressed lipase gene family with possible roles in nutrition and/or in creating an acidic microenvironment; expressed more strongly during mucosal infections than during systemic infections
orf19.1291	ABZ1	26.45	23.38	25.71	22.87	-0.007144773	NA	Ortholog(s) have 4-amino-4-deoxychorismate synthase activity, role in para-aminobenzoic acid biosynthetic process and cytosol, nucleus localization
orf19.2129	orf19.2129	24.99	23.36	25.73	21.46	-0.024640803	NA	Ortholog of S. cerevisiae Spo71; a meiosis-specific protein required for spore wall formation during sporulation in S. cerevisiae; possibly an essential gene, disruptants not obtained by UAU1 method
orf19.4269	orf19.4269	29.93	23.36	31.96	24.04	0.038107534	0.998825	Has domain(s) with predicted ATP binding, protein kinase activity and role in protein phosphorylation
orf19.2041	orf19.2041	24.73	23.36	25.14	24.94	0.02612431	NA	Ortholog(s) have DNA translocase activity, histone acetyl-lysine binding activity
orf19.6679	orf19.6679	29.97	23.35	17.8	14.28	-0.379105971	NA	Ortholog(s) have cytosol, nucleus localization
orf19.6681	orf19.6681	27.37	23.3	26.55	25.93	0.041252722	0.998825	Protein of unknown function; Spider biofilm induced
orf19.2606	HDA1	22.85	23.29	21.1	18.37	-0.131794238	NA	Histone deacetylase; inducer of filamentation; conserved deacetylation motif; regulates white-to-opaque switch frequency but not opaque-to-white switch; greater expression in white cells than opaque cells; inhibited by trichostatin-A;
orf19.6597	orf19.6597	28.71	23.28	30.22	30.68	0.129423714	0.998825	Ortholog(s) have cytoplasm localization

orf19.1992	SIR2	29.35	23.25	30.1	21.4	-0.023759201	NA	Required for wild-type lifespan, asymmetric inheritance of oxidatively damaged proteins, rDNA silencing; regulates phenotypic switch in strain 3153A, not WO-1; partially complements S. cerevisiae sir2 mating defect; Spider biofilm induced
orf19.7356	orf19.7356	22.04	23.25	20.76	19.53	-0.077882263	NA	Ortholog of C. parapsilosis CDC317 : CPAR2_807080, C. dubliniensis CD36 : Cd36_85690, Candida orthopsilosis Co 90-125 : CORT_0C02090 and Candida albicans WO-1 : CAWG_02885
orf19.2000	orf19.2000	23.77	23.25	16.15	19.63	-0.201629735	NA	Ortholog(s) have DNA helicase activity, role in DNA replication initiation, donor selection, interstrand cross-link repair, mitotic sister chromatid cohesion, negative regulation of DNA recombination and cytosol, nucleus localization
orf19.2114	orf19.2114	26.36	23.24	32.7	32.69	0.246645854	0.956789	Predicted uricase; ortholog of S. pombe SPCC1223.09; Spider biofilm induced
orf19.4991	MPT5	22.62	23.2	30.9	27.76	0.196149245	0.998825	Putative RNA-binding protein; Hap43p-repressed gene; protein induced during the mating process
orf19.2210	orf19.2210	25.83	23.2	27.2	19.63	-0.041238814	NA	Protein of unknown function; Spider biofilm induced
orf19.1523	FMO1	16.47	23.2	13.18	10.39	-0.340001787	NA	Putative oxidoreductase; mutation confers hypersensitivity to toxic ergosterol analog, and to amphotericin B
orf19.3604	orf19.3604	28.91	23.19	27.53	21.66	-0.038904839	NA	Ortholog(s) have nucleus localization
orf19.4160	orf19.4160	26.37	23.18	16.93	18.03	-0.25565172	NA	Ortholog(s) have serine-type endopeptidase activity, role in glycoprotein metabolic process, mitochondrial tRNA threonylcarbamoyladenosine modification and mitochondrion localization

orf19.4030	orf19.4030	22.6	23.18	24.15	21.36	-0.025041485	NA	Ortholog(s) have DNA primase activity, single-stranded DNA binding activity, role in DNA replication, synthesis of RNA primer, telomere maintenance and alpha DNA polymerase:primase complex localization
orf19.3617	GTR1	30.51	23.16	27.26	27.03	-0.004862149	0.998825	Putative GTP-binding protein; transcript is upregulated in clinical isolates from HIV+ patients with oral candidiasis; (see Locus History Note for Assembly 19 correction)
orf19.2389	orf19.2389	27.92	23.14	22.91	20.43	-0.140147223	NA	Ortholog(s) have DNA replication origin binding, single-stranded DNA binding activity
orf19.1878	MED4	22.14	23.11	23.05	23.82	0.026734479	NA	RNA polymerase II core mediator complex subunit
orf19.5597	POL5	27.11	23.09	20.96	24.19	-0.087665939	NA	Putative DNA Polymerase phi; F-12/CO2 early biofilm induced
orf19.4411	HOS1	23.38	23.09	23.61	21.75	-0.007710117	NA	Histone deacetylase; similar to S. cerevisiae Hos1; has conserved deacetylation motif; slightly greater expression in white cells than in opaque cells
orf19.6512	EXO70	26.79	23.09	27.18	17.86	-0.090934554	NA	Predicted subunit of the exocyst complex, involved in exocytosis; localizes to a crescent on the surface of the hyphal tip
orf19.3552	orf19.3552	26.89	23.08	24.21	21.83	-0.072545652	NA	Ortholog(s) have structural constituent of nuclear pore activity
orf19.176	OPT4	23.48	23.07	34.61	27.96	0.251715114	0.951606	Oligopeptide transporter; detected at germ tube plasma membrane; transcript inducede during phagocytosis by macrophages; fungal-specific; Hap43-repressed; merged with orf19.2292 in Assembly 20; rat catheter and Spider biofilm induced

orf19.333	FCY2	27.65	23.06	29.1	24.52	0.040025444	0.998825	Purine-cytosine permease of pyrimidine salvage; mutation associated with resistance to flucytosine in clinical isolates; transposon mutation affects filamentation; farnesol-upregulated in biofilm
orf19.3063	HFL1	17.16	23.05	22.2	18.98	0.00800837	NA	HAP5-like; ortholog of S. cerevisiae Dpb3; third-largest subunit of DNA polymerase II (DNA polymerase epsilon); phosphorylated protein; mutants have a growth defect
orf19.6392	orf19.6392	25.76	23.03	22.44	22.07	-0.090227586	NA	Ortholog of C. dubliniensis CD36 : Cd36_33870, C. parapsilosis CDC317 : CPAR2_204450, Candida tenuis NRRL Y-1498 : CANTEDRAFT_113827 and Debaryomyces hansenii CBS767 : DEHA2E04158g
orf19.5409	IST1	24.54	23.03	26.72	25.46	0.057388255	NA	Protein with a positive role in the multivesicular body sorting pathway; rat catheter biofilm repressed
orf19.1417	orf19.1417	23.63	23.02	18.74	15.1	-0.244437941	NA	Ortholog of C. dubliniensis CD36 : Cd36_43970, C. parapsilosis CDC317 : CPAR2_401720, Candida tenuis NRRL Y-1498 : CANTEDRAFT_102588 and Debaryomyces hansenii CBS767 : DEHA2G16698g
orf19.2361	SPT10	26.99	23.01	26.92	22.55	-0.007163894	NA	Ortholog(s) have sequence-specific DNA binding activity
orf19.7059	orf19.7059	28.37	22.99	32.69	26.22	0.113712949	0.998825	Ortholog(s) have endoribonuclease activity, role in nuclear mRNA surveillance of mRNP export, transcription, DNA-templated and cytoplasm, nucleus localization
orf19.5015	MYO2	21.54	22.94	34.39	35.93	0.366313661	0.811201	Class V myosin; nonessential; sole class V myosin in C. albicans; required for WT actin cytoskeletal polarity, nuclear organization, migration, hyphal growth; conserved myosin ATPase/tail domains; Hap43-induced; flow model biofilm repressed
orf19.4072	IFF6	20.29	22.94	16.87	18.09	-0.155207908	NA	Putative GPI-anchored adhesin-like protein; opaque-specific transcript; macrophage-induced gene; Hap43-repressed gene; Spider biofilm induced

orf19.5295	orf19.5295	28.58	22.89	45.5	33.81	0.360649763	0.813407	Protein with a predicted endonuclease/exonuclease/phosphatase family domain and a carbon catabolite repressor protein 4 domain; induced by alpha pheromone in SpiderM medium
orf19.1442	PLB4.5	17.1	22.88	15.83	17.4	-0.141186321	NA	Phospholipase B; Hog1-induced; regulated by Ssn6; putative GPI-anchor; repressed during cell wall regeneration; clade-associated gene expression; Hap43-induced; rat catheter and Spider biofilm repressed
orf19.5574	orf19.5574	25.05	22.85	21.86	23.1	-0.056537944	NA	Ortholog(s) have mitochondrial outer membrane localization
orf19.4353	ULP2	32.32	22.84	19.89	20.89	-0.234403808	NA	SUMO deconjugation enzyme; removes the small ubiquitin-like modifier (SUMO) from proteins; Spider biofilm induced
orf19.1317	OSH3	26.26	22.84	36.79	26.9	0.214944099	0.998825	Protein required for wild-type filamentation; has oxysterol binding protein domain and pleckstrin homology motif; overproducton suppresses cph1 homozygous null filamnetation defect; similar to S. cerevisiae Osh3p
orf19.7165	MED9	25.76	22.83	19.27	14.59	-0.276904052	NA	RNA polymerase II mediator complex subunit; Hap43p-repressed gene
orf19.5704	orf19.5704	25.46	22.81	22.04	21.07	-0.091929705	NA	Ortholog(s) have rRNA binding activity, role in mitochondrial RNA processing, mitochondrial genome maintenance, rRNA metabolic process and mitochondrion localization
orf19.3519	SUA72	26.41	22.79	28.32	22.54	0.02443619	NA	Predicted transcription factor; induced during planktonic growth, whereas related SUA71 is downregulated
orf19.2219	ORF298	21.42	22.77	19.99	23.77	-0.006293017	NA	Predicted ORF in retrotransposon Tca3; similar to Gag-Pol; a-specific transcript, alpha-factor induced; rat catheter biofilm repressed; mRNA binds She3

orf19.1396	orf19.1396	23.69	22.72	30.7	19.44	0.038013363	NA	Ortholog(s) have ARF GTPase activator activity, role in ER to Golgi vesicle-mediated transport, intra-Golgi vesicle-mediated transport and Golgi apparatus, clathrin-coated vesicle localization
orf19.5837	orf19.5837	29.24	22.66	30.22	30.2	0.11487162	0.998825	Ortholog(s) have 4-amino-4-deoxychorismate lyase activity, role in folic acid biosynthetic process and cytoplasm localization
orf19.1772	orf19.1772	30.71	22.66	20.37	21.79	-0.201366419	NA	Ortholog(s) have mitochondrion localization
orf19.4865	orf19.4865	25.46	22.65	33.65	28.76	0.219845827	0.998825	Ortholog(s) have phosphatidylinositol-3,5-bisphosphate 5-phosphatase activity, phosphatidylinositol-3-phosphatase activity, phosphatidylinositol-4-phosphate phosphatase activity and role in phosphatidylinositol dephosphorylation
orf19.4370	orf19.4370	23.28	22.64	11.5	11.08	-0.492088972	NA	Protein of unknown function; induced by nitric oxide; oxidative stress-induced via Cap1; fungal-specific (no human or murine homolog)
orf19.700	SEO1	22.06	22.62	22.83	25.4	0.043590178	NA	Protein with similarity to permeases; Sfu1-repressed; flucytosine induced; induced by Mnl1 under weak acid stress; flow model biofilm repressed
orf19.2258	orf19.2258	29.43	22.62	18.46	19.08	-0.263761543	NA	Ortholog of C. dubliniensis CD36 : Cd36_21310, C. parapsilosis CDC317 : CPAR2_406650, Candida tenuis NRRL Y-1498 : CANTEDRAFT_130046 and Debaryomyces hansenii CBS767 : DEHA2F24134g
orf19.4400	orf19.4400	27.74	22.6	32.31	26.02	0.099482772	0.998825	Has domain(s) with predicted role in anaphase-promoting complex-dependent proteasomal ubiquitin-dependent protein catabolic process, regulation of mitotic metaphase/anaphase transition and anaphase-promoting complex localization
orf19.4247	orf19.4247	26.6	22.59	27.53	30.3	0.117677782	0.998825	Protein of unknown function; rat catheter and Spider biofilm repressed

orf19.5887	NUP85	28.71	22.58	29.27	26.4	0.040050025	0.998825	Ortholog of S. cerevisiae Nup85; a structural constituent of the nuclear pore; required for alkaline-induced hyphal morphogenesis and for SD or Spider media biofilm formation
orf19.284	orf19.284	29.51	22.58	23.33	23.41	-0.118240242	NA	Ortholog of C. dubliniensis CD36 : Cd36_82890, Debaryomyces hansenii CBS767 : DEHA2G01034g, Pichia stipitis Pignal : PICST_32242 and Candida guilliermondii ATCC 6260 : PGUG_04638
orf19.5274	orf19.5274	23.46	22.55	28.07	22.37	0.056898054	NA	Ortholog(s) have cellular bud neck, cellular bud tip, cytoplasm localization
orf19.5921	orf19.5921	26.69	22.55	29.53	20.42	-0.008957162	NA	Ortholog(s) have glyoxysome localization
orf19.7627	orf19.7627	28.07	22.53	27.78	23.32	-0.007178889	NA	Ortholog(s) have role in establishment of cell polarity, mycelium development, spore germination and endoplasmic reticulum, hyphal tip, mitochondrion, plasma membrane localization
orf19.2313	orf19.2313	23.77	22.5	31.71	25.26	0.148414106	0.998825	Putative ortholog of S. cerevisiae Pex32 a peroxisomal integral membrane protein with a role in negative regulation of peroxisome size; Hap43-repressed gene
orf19.2514	orf19.2514	28	22.5	22.62	16.07	-0.212278596	NA	Predicted methyltransferase; rat catheter biofilm induced
orf19.2583.2	orf19.2583.2	9.39	22.47	6.73	9.41	-0.344328043	NA	Pseudogene; formerly an ORF Predicted by Annotation Working Group
orf19.3726	orf19.3726	30.45	22.45	24.36	20.3	-0.137258743	NA	Ortholog of C. dubliniensis CD36 : Cd36_65640 and Candida albicans WO-1 : CAWG_01583

orf19.5736	ALS5	17.36	22.42	22.16	19.17	0.02668131	NA	ALS family adhesin; highly variable; expression in S. cerevisiae causes adhesion to human epithelium, endothelium or ECM, endothelial invasiveness by endocytosis and, at high abundance, ECM-induced aggregation; can form amyloid fibrils
orf19.4800	RIM20	22.24	22.38	23.89	24.12	0.06068179	NA	Protein involved in the pH response pathway; binds to the transcription factor Rim101 and may serve as a scaffold to facilitate the C-terminal proteolytic cleavage that activates Rim101; required for alkaline pH-induced hyphal growth
orf19.6684	PNC1	20.29	22.36	27.5	23.85	0.142676438	NA	Putative nicotinamidase, involved in NAD salvage pathway; decreased transcription is observed in an azole-resistant strain that overexpresses MDR1
orf19.1582	orf19.1582	25.83	22.35	24.98	24.85	0.026792307	NA	Predicted membrane transporter; member of the drug:proton antiporter (12 spanner) (DHA1) family, major facilitator superfamily (MFS); mRNA binds She3
orf19.7661	HMI1	24.49	22.35	22.79	21.13	-0.041247845	NA	ATP-dependent 3' - 5' helicase involved in maintenance of mitochondrial DNA; ortholog of S. cerevisiae Hmi1; rat catheter biofilm repressed
orf19.2660	EAF3	26.24	22.34	18.74	16.03	-0.240024027	NA	Subunit of the NuA4 histone acetyltransferase complex; rat catheter and Spider biofilm induced
orf19.4464	SEN15	17.04	22.34	17	17.75	-0.083509599	NA	Similar to delta subunit of tRNA splicing endonuclease; increased transcription is observed upon fluphenazine treatment
orf19.834	orf19.834	27.2	22.31	20.29	15.05	-0.255977081	NA	Ortholog(s) have carbohydrate binding, mannosyl-oligosaccharide 1,2-alpha-mannosidase activity, role in ER-associated ubiquitin-dependent protein catabolic process and endoplasmic reticulum lumen localization
orf19.2077	ZCF9	23.56	22.3	21.22	19.74	-0.093955145	NA	Putative Zn(II)2Cys6 transcription factor; hypersensitive to toxic ergosterol analog ECC69 and/or ECC1384

orf19.4846	orf19.4846	28.49	22.3	27.51	25.7	0.056993931	0.998825	GlcNAc-induced protein
orf19.1461	orf19.1461	21.32	22.28	33.9	21.25	0.18172024	NA	S. pombe ortholog SPCC576.01c is a predicted sulfonate dioxygenase; possibly transcriptionally regulated upon hyphal formation; Spider biofilm induced
orf19.6525	orf19.6525	24.13	22.23	26.13	20.38	-0.008275169	NA	S. cerevisiae ortholog Inp1 is a peripheral membrane protein of peroxisomes involved in peroxisomal inheritance; induced by Mnl1 under weak acid stress
orf19.6181	orf19.6181	24.24	22.21	25.52	22.15	0.025751777	NA	Ortholog of Candida albicans WO-1 : CAWG_03093
orf19.6551	orf19.6551	18.78	22.18	27.24	20.99	0.110130605	NA	Ortholog(s) have SNAP receptor activity, role in Golgi vesicle transport, vesicle fusion and Golgi medial cisterna, SNARE complex localization
orf19.1925	TLO5	25.37	22.17	35.33	30.42	0.264657788	0.938642	Member of a family of telomere-proximal genes of unknown function; may be spliced in vivo
orf19.2461	PRN4	30.01	22.16	32.22	23.82	0.053938151	0.998825	Protein with similarity to pirins; induced by benomyl treatment; flow model biofilm repressed
orf19.2949	IML1	21.46	22.16	22.7	22.64	0.044389579	NA	Putative protein with a role in autophagy; rat catheter biofilm induced
orf19.2671	orf19.2671	18.78	22.15	17.83	23.46	-0.006079659	NA	Protein with a predicted FAD-dependent pyridine nucleotide reductase domains; putative oxidoreductase; Plc1-regulated; possibly an essential gene, disruptants not obtained by UAU1 method

orf19.6862	orf19.6862	26.54	22.14	23.04	20.8	-0.090067701	NA	Hap43-induced gene; mutation confers hypersensitivity to 5-fluorocytosine (5-FC), 5-fluorouracil (5-FU), and tubercidin (7-deazaadenosine)
orf19.4346	orf19.4346	23.83	22.13	28.85	25.73	0.137130147	NA	Ortholog(s) have protein anchor activity, role in COPII vesicle coating, protein localization to endoplasmic reticulum exit site and ER to Golgi transport vesicle membrane, endoplasmic reticulum exit site localization
orf19.3395	orf19.3395	19.67	22.1	16.71	15.25	-0.19850758	NA	Predicted MFS membrane transporter, member of the drug:proton antiporter (12 spanner) (DHA1) family; induced by nitric oxide, oxidative stress, alpha pheromone; fungal-specific; Hap43-repressed; Spider biofilm induced
orf19.7104	orf19.7104	20.14	22.06	27.61	25.48	0.174202151	NA	Adhesin-like cell wall protein; similar to mucins and to a Litomosoides microfilarial sheath protein; N-terminal secretion signal; rat catheter and Spider biofilm repressed
orf19.5381	orf19.5381	24.43	22.02	25.61	22.82	0.042497621	NA	Ortholog(s) have mRNA binding activity
orf19.4801	orf19.4801	29.1	22.02	21.83	22.09	-0.120769619	NA	Ortholog(s) have tRNA dimethylallyltransferase activity, role in tRNA modification and cytosol, mitochondrion, nucleolus localization
orf19.4119	SPO72	26.54	22	25.35	24.26	-0.005822591	NA	Protein described as similar to S. cerevisiae sporulation protein; ortholog of S. cerevisiae Atg2, an autophagic vesicle formation protein; up-regulation associated with azole resistance; Spider biofilm induced
orf19.6653	MTG2	24.71	22	20.26	19.01	-0.147002196	NA	Putative Obg family GTPase member; peripheral protein of the mitochondrial inner membrane; associates with the large ribosomal subunit; required for mitochondrial translation; rat catheter biofilm repressed
orf19.1390	PMI1	23.25	21.98	24.46	24.3	0.043769209	NA	Phosphomannose isomerase; cell wall biosynthesis enzyme; drug target; functional homolog of S. cerevisiae, E. coli phosphomannose isomerase; Gcn4-regulated; induced on adherence to polystyrene, phagocytosis; 3-AT, Spider biofilm repressed

orf19.3057	orf19.3057	29.32	21.98	43.87	37.02	0.402887826	0.720423	Ortholog(s) have role in mitochondrial genome maintenance and endoplasmic reticulum localization
orf19.5087	BUD6	25.83	21.97	29.26	22.26	0.040467182	NA	Protein required for Spitzenkorper formation in hyphal cells (wild-type localization of Mlc1p to the Spitzenkorper); localizes to polarisome
orf19.5488	orf19.5488	21.59	21.96	26.06	15.48	-0.062576166	NA	Ortholog(s) have mitochondrion localization
orf19.2580	HST2	21.59	21.96	22.29	17.75	-0.079324366	NA	Putative histone deacetylase; role in regulation of white-opaque switch; Spider biofilm induced
orf19.7041	orf19.7041	26.96	21.96	25.68	19.82	-0.056962699	NA	Putative pre-tRNA processing protein; heterozygous null mutant exhibits hypersensitivity to parnafungin and cordycepin in the C. albicans fitness test
orf19.5610	ARG3	23.41	21.95	40.17	38.89	0.481149232	0.55586	Putative ornithine carbamoyltransferase; Gcn4-regulated; Hap43-induced; repressed in alkalinizing medium; rat catheter and Spider biofilm induced
orf19.4312	orf19.4312	21.96	21.95	28.27	24.91	0.139936205	NA	Ortholog(s) have TBP-class protein binding, transcription cofactor activity
orf19.915	orf19.915	12.68	21.94	8.81	14.47	-0.255904475	NA	Protein of unknown function; Spider biofilm induced
orf19.473	TPO4	19.65	21.92	15.92	16.96	-0.176965099	NA	Putative sperimidine transporter; fungal-specific (no human or murine homolog); Spider biofilm induced; promoter bound by Tec1 and Ndt80; Bcr1-repressed in RPMI a/a biofilms

orf19.5318	RAD1	23.25	21.92	25.21	19.28	-0.025762541	NA	Putative single-stranded DNA endonuclease; transcript regulated by Nrg1; macrophage-induced gene
orf19.1576	orf19.1576	26.48	21.91	18.99	15.23	-0.259828754	NA	Ortholog(s) have nuclear localization sequence binding, ubiquitin binding activity, role in response to ethanol and cytosol localization
orf19.5662	PEP7	26.95	21.91	34.95	29.37	0.217504643	0.998825	Vesicle transport protein; acts in endosomal transport; roles in metal ion resistance, virulence, adhesion, hyphal growth, chlamydospore formation; similar to S. cerevisiae Pep7p
orf19.6184	orf19.6184	24.5	21.91	29.58	28.7	0.180874367	0.998825	Protein of unknown function; Spider biofilm repressed
orf19.4085	orf19.4085	21	21.89	24.53	17.64	-0.009550948	NA	Protein of unknown function; transcript detected on high-resolution tiling arrays
orf19.4837.1	orf19.4837.1	13.91	21.85	22.79	19.81	0.115783316	NA	Ortholog of C. parapsilosis CDC317 : CPAR2_804880, Candida tenuis NRRL Y-1498 : CANTEDRAFT_111283, Debaryomyces hansenii CBS767 : DEHA2B10604g and Pichia stipitis Pignal : PICST_40454
orf19.6742	orf19.6742	26.74	21.85	31.54	29.53	0.19096662	0.998825	Ortholog(s) have CTD phosphatase activity
orf19.5074	UBA2	19.97	21.84	26.92	23.95	0.142676438	NA	Ortholog(s) have SUMO activating enzyme activity, role in protein sumoylation and SUMO activating enzyme complex localization
orf19.6907	orf19.6907	20.82	21.83	21.78	16.66	-0.08075717	NA	Ortholog(s) have DNA binding activity and cytoplasm, nuclear chromatin localization

orf19.3419	MAE1	22.71	21.82	25.39	27.62	0.124562963	NA	Malic enzyme, mitochondrial; transcription regulated by Mig1, Tup1; colony morphology-related gene regulation by Ssn6; Hap43-repressed; Spider biofilm repressed
orf19.6929	orf19.6929	22.91	21.79	28.38	22.47	0.074295001	NA	Ortholog(s) have acid phosphatase activity and cytosol, nucleus localization
orf19.6790	orf19.6790	21.31	21.78	33.81	31.79	0.347118564	0.846842	Ortholog(s) have mRNA binding activity, role in 3'-UTR-mediated mRNA destabilization, mitochondrion organization and cytoplasmic mRNA processing body, cytoplasmic stress granule, cytosol, perinuclear region of cytoplasm localization
orf19.2269	orf19.2269	21.75	21.75	37.97	39.76	0.486148591	0.547054	Putative 3-phosphoserine phosphatase; induced by benomyl or in azole-resistant strain that overexpresses MDR1; early-stage flow model biofilm induced; Spider biofilm repressed
orf19.3113	orf19.3113	24.98	21.73	18.45	20.75	-0.145107678	NA	Protein of unknown function; Spider biofilm induced
orf19.1893	orf19.1893	23.06	21.72	33.48	26.01	0.211861567	0.998825	Protein of unknown function; Spider biofilm repressed
orf19.4912	orf19.4912	25.37	21.72	26.35	22.04	0.009247428	NA	Ortholog(s) have ubiquitin protein ligase binding activity, role in protein ubiquitination, ubiquitin-dependent endocytosis and Golgi apparatus, clathrin-coated vesicle, cytosol, nucleus, plasma membrane localization
orf19.2001	orf19.2001	20.96	21.71	18.13	15.1	-0.195995865	NA	Has domain(s) with predicted L-ascorbic acid binding, iron ion binding, oxidoreductase activity, acting on paired donors, with incorporation or reduction of molecular oxygen and 2-oxoglutarate as one donor, more
orf19.2881	MNN4	21.1	21.71	17.67	21.04	-0.077932779	NA	Regulator of mannosylphosphorylation of N-linked mannans to cell wall proteins; no role in virulence or normal interaction with macrophages; mutant induces inflammatory cytokines in dendritic cells; rat catheter and Spider biofilm induced

orf19.510	orf19.510	16.71	21.69	15.54	21.01	-0.043533927	NA	Protein of unknown function; Spider biofilm induced
orf19.3000	ORC1	19.69	21.67	25.12	19.4	0.025385287	NA	Putative origin recognition complex large subunit; essential for viability; similar to S. cerevisiae Orc1p origin recognition complex subunit
orf19.3783	orf19.3783	11.06	21.67	18.97	17.64	0.0644434	NA	Protein of unknown function; rat catheter biofilm induced
orf19.5930	orf19.5930	23.22	21.67	26.05	18.38	-0.026329013	NA	Ortholog(s) have nucleocytoplasmic transporter activity, structural constituent of nuclear pore activity
orf19.5518	orf19.5518	20.63	21.65	35.75	29.56	0.345429033	0.849053	Protein of unknown function; Spider biofilm induced
orf19.3323	orf19.3323	22.1	21.65	20.95	15.92	-0.135305845	NA	Ortholog of C. dubliniensis CD36 : Cd36_01240, C. parapsilosis CDC317 : CPAR2_109140, Candida tenuis NRRL Y-1498 : CANTEDRAFT_114227 and Debaryomyces hansenii CBS767 : DEHA2D15026g
orf19.2415	orf19.2415	19.96	21.65	23.06	20.99	0.026832408	NA	Ortholog(s) have role in mitochondrial translation and mitochondrion localization
orf19.5932	orf19.5932	25.05	21.64	23.57	19.95	-0.058337675	NA	Protein of unknown function; Spider biofilm induced
orf19.6264	orf19.6264	18.15	21.62	22.9	19.69	0.044207177	NA	Ortholog of C. dubliniensis CD36 : Cd36_06080, C. parapsilosis CDC317 : CPAR2_803360, Candida tenuis NRRL Y-1498 : CANTEDRAFT_112428 and Debaryomyces hansenii CBS767 : DEHA2G15532g

orf19.6909	orf19.6909	27.41	21.61	22.71	17.31	-0.161811845	NA	Ortholog(s) have TFIIH-class transcription factor binding, TFIIH-class transcription factor recruiting transcription factor activity
orf19.793	CAK1	34.55	21.61	31.32	26.34	-0.004862909	0.998825	Monomeric CDK-activating kinase; functional homolog of S. cerevisiae Cak1p; phosphorylates cyclin-free human CDK2; lacks glycine loop motif; conserved lysine (K36) not required for activity; possibly essential gene (by UAU1 method)
orf19.5599	MDL2	27.55	21.58	26.46	23.32	-0.02233066	NA	Putative mitochondrial, half-size MDR-subfamily ABC transporter
orf19.5034	YBP1	18.6	21.57	19.32	18.07	-0.08197979	NA	Protein involved in response to oxidative stress, binds and stabilizes Cap1p transcription factor in response to H2O2; essential for macrophage killing
orf19.5038	orf19.5038	24.38	21.54	22.12	22.83	-0.023016819	NA	Predicted tRNA (guanine) methyltransferase activity; Spider biofilm induced
orf19.2650	orf19.2650	23.79	21.53	22.68	18.27	-0.095051001	NA	Protein of unknown function; rat catheter biofilm induced
orf19.2939	orf19.2939	37.84	21.53	34.36	23.3	-0.048358077	0.998825	Ortholog(s) have mitochondrion localization
orf19.3066	ENG1	21.16	21.53	20.79	21.2	-0.02488212	NA	Endo-1,3-beta-glucanase; ortholog of S. cerevisiae Dse4 needed for cell separation; caspofungin, fluconazole repressed; repressed by alpha pheromone in SpiderM medium; flow model biofilm induced; rat catheter biofilm repressed
orf19.3774.1	orf19.3774.1	18.57	21.5	26.32	23.38	0.146542268	NA	Ortholog(s) have protein tag activity and role in cell morphogenesis involved in conjugation with cellular fusion, cellular protein localization, cellular protein modification process, mRNA cis splicing, via spliceosome

orf19.7579	FGR34	24.95	21.5	27.85	21.91	0.058262929	NA	Protein lacking an ortholog in S. cerevisiae; transposon mutation affects filamentous growth
orf19.1395	orf19.1395	27.61	21.49	37.52	38.79	0.389225556	0.752654	Ortholog(s) have inorganic phosphate transmembrane transporter activity, role in phosphate ion transport, transmembrane transport and mitochondrion localization
orf19.6832	orf19.6832	25.19	21.46	27.53	24.92	0.106875469	NA	Protein of unknown function; regulated by Sef1p-, Sfu1p-, and Hap43p
orf19.3087.2	orf19.3087.2	26.51	21.46	27.7	31.46	0.165511591	0.998825	Ortholog of C. dubliniensis CD36 : Cd36_26300, Candida tenuis NRRL Y-1498 : cten_CGOB_00015, Debaryomyces hansenii CBS767 : DEHA2G16104g and Pichia stipitis Pignal : psti_CGOB_00212
orf19.6901	orf19.6901	21.03	21.44	22.85	23.67	0.079593871	NA	Ortholog(s) have exoribonuclease activator activity, m7G(5')pppN diphosphatase activity
orf19.4733	YMC2	18.12	21.44	37.8	30.72	0.456559453	0.612468	Putative mitochondrial carrier protein; Gcn4-regulated; F-12/ CO2 early biofilm induced; Spider biofilm induced
orf19.1990	SNX4	26.73	21.43	35.35	25.67	0.190229796	0.998825	Putative sorting nexin; induced during the mating process
orf19.4575	orf19.4575	29.24	21.4	28.9	20.11	-0.023261902	NA	Ortholog(s) have mitochondrion localization
orf19.6414	orf19.6414	26.4	21.38	26.14	22.09	0.009933404	NA	Ortholog of C. dubliniensis CD36 : Cd36_34040, C. parapsilosis CDC317 : CPAR2_205850, Candida tenuis NRRL Y-1498 : CANTEDRAFT_132607 and Debaryomyces hansenii CBS767 : DEHA2A11638g

orf19.4847	orf19.4847	29.21	21.36	35.22	29.55	0.215573427	0.998825	Ortholog of Candida albicans WO-1 : CAWG_00450
orf19.5495	orf19.5495	20.88	21.32	22.33	20.81	0.010517177	NA	Putative RNA-binding protein; induced by alpha pheromone in SpiderM medium
orf19.4769	IPT1	23.01	21.32	21.63	18.74	-0.060204046	NA	Inositol phosphoryl transferase; catalyzes the synthesis of the most abundant sphingolipid, mannose-(inositol-P)2-ceramide, M(IP)2C, from MIPC; required for wild-type membrane localization of Cdr1; Spider biofilm induced
orf19.470	orf19.470	22.96	21.31	19.16	16.68	-0.153140633	NA	Ortholog(s) have RNA polymerase II activating transcription factor binding, TBP-class protein binding, chromatin binding, protein complex scaffold activity
orf19.6581	orf19.6581	27.17	21.31	27.93	24.72	0.07388831	NA	Ortholog(s) have palmitoyltransferase activity, role in protein palmitoylation and endoplasmic reticulum localization
orf19.6297	orf19.6297	26.62	21.29	22.76	16.81	-0.144855166	NA	Ortholog(s) have pseudouridine synthase activity, role in tRNA pseudouridine synthesis and cytosol, nucleus localization
orf19.852	SAP98	23.35	21.28	16.09	20.02	-0.150129537	NA	Glycosyl-phosphatidylinositol-anchored aspartic endopeptidase; regulated by Gcn2p and Gcn4p; expressed only in opaque MTLa/MTLa cells
orf19.580	orf19.580	24.85	21.27	24.6	21.92	0.010182037	NA	Protein of unknown function; mutants are viable; Spider biofilm induced
orf19.7023	orf19.7023	18.24	21.25	24.52	27.99	0.22941874	NA	Ortholog of S. cerevisiae Ioc4, a protein that influences gene expression through chromatin remodeling; oxidative stress-induced via Cap1

orf19.7339	BGL22	21.57	21.23	23.57	22.69	0.061660623	NA	Putative glucanase; induced during cell wall regeneration
orf19.875	orf19.875	27.92	21.23	26.66	23.25	0.010083261	NA	Protein required for respiratory growth
orf19.4326	orf19.4326	20.23	21.23	17.53	18.71	-0.080628244	NA	Ortholog(s) have role in mRNA cis splicing, via spliceosome, maturation of 5S rRNA and U4/U6 x U5 tri-snRNP complex localization
orf19.5320	NCE4	19.3	21.22	21.17	24.24	0.081527471	NA	Putative RecQ-mediated genome instability protein; Hap43-repressed gene; periodic mRNA expression, peak at cell-cycle G2/M phase; flow model biofilm induced
orf19.3578	orf19.3578	20.35	21.22	18.53	22.49	-0.006056697	NA	Ortholog of C. dubliniensis CD36 : Cd36_19950, C. parapsilosis CDC317 : CPAR2_104135, Candida tenuis NRRL Y-1498 : cten_CGOB_00075 and Debaryomyces hansenii CBS767 : DEHA2F07964g
orf19.4878	orf19.4878	20.78	21.21	38.99	30.1	0.402412109	0.735159	Protein of unknown function; Hap43-repressed; rat catheter biofilm repressed
orf19.1359	orf19.1359	23.52	21.18	26.52	22.39	0.059179495	NA	Ortholog(s) have protein complex binding activity, role in ER-associated ubiquitin-dependent protein catabolic process, sporulation resulting in formation of a cellular spore and cytoplasm, extrinsic to membrane, nucleus localization
orf19.4861.1	orf19.4861.1	23.36	21.17	38.59	26.93	0.324737345	0.87024	Protein of unknown function
orf19.6005	HGT5	25.55	21.16	29.35	23.24	0.07352721	NA	Putative glucose transporter of the major facilitator superfamily; the C. albicans glucose transporter family comprises 20 members; 12 probable membrane-spanning segments, extended N terminus; expressed in rich medium; Hap43p-repressed

orf19.3225	CWH43	22.78	21.15	38.14	33.1	0.396600957	0.743771	Putative sensor/transporter protein with a predicted role in cell wall biogenesis; possibly an essential gene, disruptants not obtained by UAU1 method; flow model and rat catheter biofilm repressed
orf19.1618.1	orf19.1618.1	33.67	21.15	38.37	23.97	0.092449196	0.998825	Ortholog(s) have cytosol localization
orf19.1240	orf19.1240	18.78	21.13	18.17	19.16	-0.062771306	NA	Ortholog of S. cerevisiae : YPR117W, C. glabrata CBS138 : CAGL0D04510g, C. dubliniensis CD36 : Cd36_45200, C. parapsilosis CDC317 : CPAR2_500480 and Candida tenuis NRRL Y-1498 : CANTEDRAFT_120679
orf19.4890	CLA4	20.85	21.09	24.43	21.16	0.044500439	NA	Ste20p family Ser/Thr kinase required for wild-type filamentous growth, organ colonization and virulence in mouse systemic infection; role in chlamydospore formation; functional homolog of S. cerevisiae Cla4p; mutant caspofungin sensitive
orf19.6559	orf19.6559	27.1	21.09	26.9	22.56	0.026166277	NA	RNA polymerase III transcription initiation factor complex (TFIIIC) subunit; growth phase regulated protein; downregulaated in stationary phase yeast cultures; Hap43-repressed; flow model biofilm induced; Spider biofilm repressed
orf19.867	orf19.867	19.7	21.08	30.91	20.9	0.173101414	NA	Ortholog(s) have superoxide-generating NADPH oxidase activity, role in apoptotic process, regulation of actin cytoskeleton organization and perinuclear endoplasmic reticulum, ribosome localization
orf19.3521	ARH2	21.32	21.07	31.29	33.67	0.352403401	0.841979	Putative adrenodoxin-NADPH oxidoreductase; role in heme biosynthesis
orf19.6817	FCR1	22.04	21.07	12.06	7.52	-0.488072802	NA	Transcription factor; repressor of fluconazole/ketoconazole/brefeldin A resistance; Tn mutation enhances filamentation; partially rescues S. cerevisiae pdr1 pdr3 fluconazole sensitivity; rat catheter biofilm induced/Spider biofilm repressed
orf19.2933	orf19.2933	19.76	21.07	20.08	18.76	-0.043936463	NA	Ortholog(s) have ubiquitin-specific protease activity, role in protein deubiquitination, regulation of transcription, DNA-dependent and cytosol, nucleus localization

orf19.2654	RMS1	29.24	21.05	23.57	19.09	-0.122887064	NA	Putative lysine methyltransferase; Hap43-induced; protein induced during mating; possibly essential, disruptants not obtained by UAU1 method; rat catheter and Spider biofilm induced
orf19.353	ULP1	20.14	21.02	19.55	19.9	-0.025220858	NA	SUMO deconjugation enzyme that cleaves the small ubiquitin-like modifier (SUMO) off proteins
orf19.1500	orf19.1500	25.05	21.01	20.84	23.26	-0.03909689	NA	Ortholog of C. dubliniensis CD36 : Cd36_16720, C. parapsilosis CDC317 : CPAR2_210210, Candida tenuis NRRL Y-1498 : CANTEDRAFT_128446 and Debaryomyces hansenii CBS767 : DEHA2B04092g
orf19.5267	orf19.5267	16.56	21	9.27	9.26	-0.447404531	NA	Putative cell wall adhesin-like protein; repressed in core caspofungin response and by alpha pheromone in SpiderM medium; transcript reduced in ace2 mutant; flow model, rat catheter and Spider biofilm repressed
orf19.4078	orf19.4078	30.11	21	20.76	15.19	-0.265760976	NA	Ortholog(s) have role in U1 snRNA 3'-end processing, U4 snRNA 3'-end processing and U5 snRNA 3'-end processing, more
orf19.275	orf19.275	22.34	20.98	18.23	16.87	-0.155100753	NA	Ortholog(s) have RNA binding, ribonuclease MRP activity, ribonuclease P activity, role in intronic box C/D snoRNA processing, mRNA cleavage and endoplasmic reticulum, nucleolar ribonuclease P complex, ribonuclease MRP complex localization
orf19.1978	GIT2	21.78	20.97	50.15	44.47	0.688242915	0.181528	Putative glycerophosphoinositol permease; fungal-specific; repressed by alpha pheromone in SpiderM medium; Hap43-repressed; Spider biofilm induced
orf19.5163	SFI1	22.02	20.92	20.22	14.76	-0.155963182	NA	Putative centrin-binding protein; predicted role in spindle pole body duplication; induced by alpha pheromone in SpiderM medium; essential for growth
orf19.2509.1	orf19.2509.1	32.39	20.89	23.97	16.66	-0.203373378	NA	Ortholog of C. dubliniensis CD36 : Cd36_29340, Spathaspora passalidarum NRRL Y-27907 : spas_CGOB_00040 and Candida albicans WO-1 : CAWG_00248

orf19.1994	orf19.1994	26.05	20.89	23.18	19.11	-0.092551891	NA	Ortholog(s) have phosphatidylinositol-3-phosphate binding activity, role in retrograde transport, endosome to Golgi and endosome localization
orf19.831	orf19.831	26.99	20.85	24.59	20.18	-0.056830925	NA	Ortholog of C. dubliniensis CD36 : Cd36_18570, C. parapsilosis CDC317 : CPAR2_211360, Candida tenuis NRRL Y-1498 : CANTEDRAFT_115359 and Debaryomyces hansenii CBS767 : DEHA2D06886g
orf19.7489	orf19.7489	18.34	20.83	24.93	23.38	0.148320704	NA	Ortholog(s) have Rho GTPase activator activity
orf19.5846	orf19.5846	27.23	20.82	23.4	18.89	-0.108707426	NA	Putative TFIIH and nucleotide excision repair factor 3 complex subunit; possibly an essential gene, disruptants not obtained by UAU1 method
orf19.5483	orf19.5483	25.16	20.82	23.4	21.89	-0.022954557	NA	Ortholog(s) have GTPase activity, ribosome binding activity, role in positive regulation of translation and mitochondrial matrix, mitochondrial ribosome localization
orf19.1968	orf19.1968	13.26	20.82	19.52	18.88	0.084075278	NA	Ortholog of C. dubliniensis CD36 : Cd36_51280, C. parapsilosis CDC317 : CPAR2_501840, Candida tenuis NRRL Y-1498 : cten_CGOB_00020 and Debaryomyces hansenii CBS767 : DEHA2F24310g
orf19.3884	FGR50	29.91	20.8	25.94	21	-0.070436925	NA	Protein lacking an ortholog in S. cerevisiae; transposon mutation affects filamentous growth; Spider biofilm repressed
orf19.319	orf19.319	17.19	20.8	18.04	16.23	-0.085479232	NA	Ortholog of S. cerevisiae : YDR286C, C. glabrata CBS138 : CAGL0H01111g, C. dubliniensis CD36 : Cd36_83160, C. parapsilosis CDC317 : CPAR2_101820 and Candida tenuis NRRL Y-1498 : CANTEDRAFT_113333
orf19.6980	orf19.6980	19.22	20.79	18.64	16.84	-0.083008511	NA	Has domain(s) with predicted ATP binding, protein tyrosine kinase activity and role in protein phosphorylation

orf19.7164	orf19.7164	30.04	20.77	22.94	16.6	-0.191517867	NA	Ortholog(s) have role in ascospore formation, conidiophore development, conidium formation, response to oxidative stress, sporocarp development involved in sexual reproduction, vacuolar protein processing
orf19.6934	orf19.6934	22.65	20.77	18.23	19.15	-0.132798538	NA	Ortholog(s) have role in protein insertion into mitochondrial membrane from inner side and integral to mitochondrial inner membrane localization
orf19.4627	orf19.4627	19.89	20.76	20.05	19.28	-0.043936463	NA	Ortholog(s) have structural constituent of nuclear pore activity
orf19.1124	orf19.1124	17.13	20.76	22	17.29	0.008648829	NA	Protein of unknown function; flow model biofilm induced; Spider biofilm induced
orf19.1305	orf19.1305	22.55	20.75	24.56	26.62	0.126374798	NA	Ortholog(s) have tRNA (guanine) methyltransferase activity, role in tRNA methylation and mitochondrial matrix, nucleus localization
orf19.7620	orf19.7620	20.76	20.75	22.37	18.69	-0.025712783	NA	Ortholog(s) have role in mRNA splicing, via spliceosome and spliceosomal complex localization
orf19.604	orf19.604	19.79	20.74	17.32	14.71	-0.180326328	NA	Ortholog(s) have cytosol localization
orf19.21	orf19.21	16.8	20.74	9.85	10.83	-0.36990142	NA	Ortholog(s) have role in ethanol metabolic process and mitochondrial inner membrane localization
orf19.5388	SWD1	23.49	20.7	21.21	21.06	-0.041308166	NA	Ortholog(s) have histone methyltransferase activity (H3-K4 specific) activity, role in chromatin silencing at telomere, histone H3-K4 methylation, telomere maintenance and Set1C/COMPASS complex, cytosol localization

orf19.427	orf19.427	20.52	20.69	17.47	16.95	-0.15698869	NA	Ortholog(s) have telomeric DNA binding activity and role in chromatin silencing at silent mating-type cassette, chromatin silencing at telomere, single-species biofilm formation on inanimate substrate, telomere capping
orf19.1421	orf19.1421	18.6	20.69	20.64	19.14	-0.008119386	NA	Ortholog(s) have ureidoglycolate hydrolase activity and cytosol, nucleus localization
orf19.6141	HGT16	25.71	20.67	17.13	14.5	-0.282670087	NA	Putative glucose transporter of the major facilitator superfamily; the C. albicans glucose transporter family comprises 20 members; 12 probable membrane-spanning segments; gene has intron; expressed in rich medium with 2% glucose
orf19.4187	orf19.4187	23.36	20.66	24.29	24.7	0.078097872	NA	Ortholog(s) have role in hyphal growth, mitochondrial genome maintenance, phospholipid transport, protein import into mitochondrial outer membrane, sporocarp development involved in sexual reproduction
orf19.6266	orf19.6266	20.01	20.64	20.38	16.37	-0.1014441	NA	Protein of unknown function; Spider biofilm induced
orf19.6900	orf19.6900	21.07	20.63	25.36	21.87	0.078327753	NA	Ortholog(s) have role in establishment or maintenance of cell polarity, mitochondrial genome maintenance, mitochondrion inheritance, phospholipid transport, protein import into mitochondrial outer membrane
orf19.7098	orf19.7098	18.21	20.59	16.08	14.28	-0.185464604	NA	Putative protein of unknown function; Hap43-repressed; repressed by nitric oxide; Spider biofilm induced
orf19.6988	OST1	24.03	20.59	34.63	31.98	0.326916794	0.86673	Alpha subunit of the oligosaccharyltransferase complex of the ER lumen; catalyzes asparagine-linked glycosylation of newly synthesized proteins; Spider biofilm repressed
orf19.2127	CST5	19.55	20.52	16.25	21.11	-0.078613424	NA	Scaffold protein for the mitogen-activated protein (MAP) kinase cascade that regulates mating; required for opaque mating or white biofilm formation in response to mating pheromone; induced in response to pheromone; Hap43p-repressed

orf19.4521	orf19.4521	23.87	20.5	25.48	34.2	0.215060688	0.998825	S. cerevisiae ortholog Env9 has similarity to oxidoreductases and is proposed to have vacuolar functions, found in lipid particles; hyphal-induced expression
orf19.2882	XUT1	24.14	20.47	15.53	13.12	-0.294848411	NA	Putative high-affinity, high-capacity xanthine-uric acid/H+ symporter; similar to A. nidulans UapA; member of the Nucleobase-Ascorbate Transporter/Nucleobase-Cation Symporter (NAT/NCS2) family; rat catheter biofilm induced
orf19.2984	MST1	27.84	20.47	22.46	19.73	-0.10709871	NA	Ortholog(s) have role in mitochondrial threonyl-tRNA aminoacylation and mitochondrion localization
orf19.4316	orf19.4316	18.42	20.46	29.98	24.01	0.261551943	NA	Trimethyllysine dioxygenase, the first enzyme in the carnitine biosynthesis pathway; hypha-induced expression, regulated by Cyr1, Ras1, Efg1; rat catheter biofilm repressed
orf19.5210.1	orf19.5210.1	22.04	20.46	31.4	25.1	0.220713717	NA	Protein conserved in C. dublinensis
orf19.3019	orf19.3019	23.08	20.44	22.47	21.28	-0.006103013	NA	Putative DNA-dependent ATPase; transcription may be increased in an azole-resistant strain that overexpresses MDR1
orf19.5938	SEN1	19.78	20.43	19.06	19.17	-0.043643979	NA	Putative helicase; repressed by prostaglandins
orf19.729	orf19.729	23.41	20.42	25.87	24.71	0.127631508	NA	Adaptor protein required for specific mRNA transport; protein similar but not orthologous to S. cerevisiae She3; transposon mutation affects filamentous growth
orf19.4922	orf19.4922	25.42	20.42	31.69	26.25	0.198660443	NA	Ortholog(s) have Rab GTPase binding activity and Golgi apparatus, endoplasmic reticulum localization

orf19.672	orf19.672	25.41	20.41	21.31	20.14	-0.075604142	NA	Ortholog(s) have U5 snRNP localization
orf19.6822	orf19.6822	30.19	20.41	24.81	22.68	-0.036720495	NA	Ortholog(s) have NEDD8 ligase activity, ubiquitin binding activity and role in positive regulation of ubiquitin-protein ligase activity, protein neddylation
orf19.5346	orf19.5346	23.5	20.4	26.67	23.12	0.110542277	NA	Ortholog(s) have role in transcription from RNA polymerase II promoter and transcription factor TFIID complex localization
orf19.1906	orf19.1906	17.93	20.4	21.59	17.26	0.00947378	NA	Protein of unknown function; flow model biofilm induced
orf19.4614	orf19.4614	21.9	20.37	21.16	21.2	-0.006061158	NA	Adapter protein for pexophagy and the cytoplasm-to-vacuole targeting (Cvt) pathway; Spider biofilm induced
orf19.3508	orf19.3508	19.21	20.36	41.12	34.99	0.55004377	0.41868	Putative protein of unknown function; stationary phase enriched protein
orf19.1182	orf19.1182	27.97	20.33	34.45	25.35	0.161587128	0.998825	Ortholog(s) have SNAP receptor activity, phosphatidylinositol-3-phosphate binding activity and role in piecemeal microautophagy of nucleus, vacuole fusion, non-autophagic, vesicle fusion
orf19.6119	orf19.6119	19.56	20.28	18.59	17.67	-0.062890045	NA	Protein of unknown function; Spider biofilm repressed
orf19.3105	orf19.3105	23.86	20.27	22.96	21.88	0.011396951	NA	Putative cytochrome P450 protein; possibly an essential gene, disruptants not obtained by UAU1 method

orf19.1557	orf19.1557	16.22	20.27	14.15	19.76	-0.044009764	NA	Ortholog(s) have S-adenosylmethionine-dependent methyltransferase activity, role in protein methylation and cytosol, nucleus localization
orf19.321	orf19.321	17.46	20.27	19.77	14.6	-0.047701264	NA	Ortholog(s) have L-methionine transmembrane transporter activity and role in methionine import
orf19.5755	orf19.5755	16.2	20.24	11.7	10.72	-0.284269847	NA	Ortholog(s) have cyclin-dependent protein serine/threonine kinase regulator activity
orf19.6719	orf19.6719	19.98	20.21	20.77	18.51	-0.007366761	NA	Putative nucleosome assembly protein; homozygous transposon insertion causes decreased colony wrinkling under filamentous growth-inducing conditions, but does not block true hyphal formation in liquid media
orf19.6703	orf19.6703	21.63	20.2	20.92	17.56	-0.061466453	NA	Ortholog of C. dubliniensis CD36 : Cd36_73260 and Candida albicans WO-1 : CAWG_05699
orf19.2664	orf19.2664	22.38	20.19	23.14	23.27	0.0632637	NA	Ortholog(s) have role in cellular response to DNA damage stimulus, interspecies interaction between organisms and cytosol, nucleus, polysome localization
orf19.5763	orf19.5763	19.71	20.19	23.3	27.41	0.165383804	NA	Has domain(s) with predicted oxidoreductase activity and role in metabolic process
orf19.2537	orf19.2537	14.85	20.19	11.85	7.7	-0.336721872	NA	Protein of unknown function; transcript detected on high-resolution tiling arrays
orf19.178	orf19.178	25.41	20.13	23.79	18.51	-0.041342327	NA	Ortholog of C. dubliniensis CD36 : Cd36_32460, C. parapsilosis CDC317 : CPAR2_205480, Candida tenuis NRRL Y-1498 : CANTEDRAFT_115156 and Debaryomyces hansenii CBS767 : DEHA2E22154g

orf19.5870	CTP1	28.05	20.12	32.25	32.66	0.249843787	0.951606	Putative citrate transport protein; flucytosine induced; amphotericin B repressed, caspofungin repressed; Hap43p-induced gene
orf19.5859	DAL8	15.18	20.11	15.26	15.95	-0.087609791	NA	Putative allantoate permease; fungal-specific (no human or murine homolog)
orf19.2229	orf19.2229	25.05	20.08	17.56	21.04	-0.110272369	NA	Ortholog(s) have ubiquitin-protein ligase activity, role in protein ubiquitination, response to ethanol and cytoplasm, nucleus localization
orf19.1755	SET2	23.64	20.07	22.42	18.78	-0.059400976	NA	Ortholog(s) have histone methyltransferase activity (H3-K36 specific) activity
orf19.1428	DUO1	17.04	20.07	15.49	14.72	-0.147100795	NA	Subunit of the Dam1 (DASH) complex, which acts in chromosome segregation by coupling kinetochores to spindle microtubules
orf19.6080	orf19.6080	28.05	20.05	24.49	22.45	-0.038180391	NA	Ortholog(s) have role in mitotic spindle orientation checkpoint and spindle pole body localization
orf19.4177	HIS5	22.77	20.03	28.94	28.29	0.220248313	NA	Putative histidinol-phosphate aminotransferase; Gcn4p-regulated; protein present in exponential and stationary growth phase yeast cultures
orf19.68.2	orf19.68.2	19.56	20.03	25.22	15.22	-0.01005579	NA	Ortholog of C. dubliniensis CD36 : Cd36_11670, C. parapsilosis CDC317 : CPAR2_201750, Candida tenuis NRRL Y-1498 : CANTEDRAFT_115679 and Debaryomyces hansenii CBS767 : DEHA2E16940g
orf19.7010	orf19.7010	27.21	20.02	22.7	19.46	-0.091568077	NA	Ortholog(s) have cytosol, ubiquitin ligase complex localization

orf19.1567	orf19.1567	25.06	20.02	21.98	18.25	-0.094585828	NA	Ortholog(s) have Rab guanyl-nucleotide exchange factor activity, phosphatidylinositol binding activity
orf19.3461	orf19.3461	22.24	20.01	33.27	43.23	0.494946357	0.541043	Protein of unknown function; oxidative stress-induced via Cap1; induced by alpha pheromone in SpiderM medium
orf19.1814	STT4	21.7	20.01	30.72	26.29	0.236278009	NA	Putative phosphatidylinositol-4-kinase
orf19.3352	orf19.3352	21.33	20	21.28	15.81	-0.081890461	NA	Has domain(s) with predicted oxidoreductase activity and role in metabolic process
orf19.3767	PEP1	19.35	19.98	31.54	30.07	0.364193875	NA	Type I transmembrane sorting receptor for multiple vacuolar hydrolases; cycles between late-Golgi and prevacuolar endosome-like compartments; rat catheter biofilm repressed
orf19.707	APG7	24.8	19.97	24.64	26.65	0.109924688	NA	Ortholog(s) have APG12 activating enzyme activity, APG8 activating enzyme activity
orf19.3661	orf19.3661	23.11	19.97	23.12	21.4	0.010998266	NA	Putative deubiquitinating enzyme; induced by Mnl1 under weak acid stress
orf19.3919	orf19.3919	23.12	19.96	28.39	23.22	0.126155654	NA	RNI-like superfamily domain-containing protein; early-stage flow model biofilm induced; Spider biofilm induced
orf19.1420	orf19.1420	20.43	19.96	24.5	18.08	0.044349909	NA	Ortholog(s) have cytosol, nucleus localization

orf19.5114	orf19.5114	31.96	19.92	34.36	28.96	0.156042153	0.998825	Sorting nexin; role in maintaining late-Golgi resident enzymes in their proper location by recycling molecules from the prevacuolar compartment; Spider biofilm induced
orf19.1938	orf19.1938	19.3	19.89	16.24	12.62	-0.206613123	NA	Ortholog of S.pombe SPCC825.05c; a predicted splicing coactivator; transcription repressed in azole-resistant strain overexpressing CDR1 and CDR2; induced by benomyl treatment
orf19.3422	FMP27	19.17	19.88	24.91	24.38	0.166233888	NA	Putative mitochondrial protein; mRNA binds She3
orf19.4209	orf19.4209	27.84	19.88	25.12	21.52	-0.021795624	NA	Ortholog(s) have NEDD8 activating enzyme activity, role in protein neddylation and cytosol, nucleus localization
orf19.3272	orf19.3272	28.22	19.88	22.29	21.9	-0.071514368	NA	Ortholog(s) have chaperone binding activity, role in box C/D snoRNP assembly, protein folding and R2TP complex, cytoplasm, nucleus localization
orf19.2968	orf19.2968	20.52	19.87	15.35	11.19	-0.30870395	NA	Protein of unknown function; Spider biofilm induced
orf19.6693	orf19.6693	19.75	19.85	21.09	22.36	0.04730766	NA	Ortholog(s) have cytosol, nucleus localization
orf19.3954	PSD2	22.32	19.85	32.13	27.21	0.265916058	NA	Ortholog(s) have phosphatidylserine decarboxylase activity, role in phosphatidylcholine biosynthetic process, phosphatidylethanolamine biosynthetic process and cell division site, cytosol, endosome localization
orf19.6134	orf19.6134	22.78	19.84	35.62	29.83	0.346999204	0.846842	Ortholog(s) have role in ER-dependent peroxisome organization, retrograde vesicle-mediated transport, Golgi to ER and Dsl1p complex, endoplasmic reticulum membrane, nuclear envelope, peroxisome localization

orf19.479.2	SEC22	17.74	19.8	32.73	22.98	0.289040696	NA	Ortholog(s) have SNAP receptor activity and role in ER to Golgi vesicle-mediated transport, retrograde vesicle-mediated transport, Golgi to ER, vesicle fusion with Golgi apparatus
orf19.166	ASG1	21.97	19.8	16.3	15.67	-0.196616677	NA	Gal4p family zinc-finger transcription factor with similarity to S. cerevisiae Asg1p
orf19.5382	orf19.5382	5.87	19.79	12.69	14.19	0.009439845	NA	Ortholog of Candida albicans WO-1 : CAWG_02410
orf19.1393	orf19.1393	25.09	19.79	22.81	16.51	-0.094982348	NA	Ortholog(s) have NAD transporter activity, pyruvate secondary active transmembrane transporter activity, role in NAD transmembrane transport, mitochondrial pyruvate transport and endoplasmic reticulum, mitochondrion localization
orf19.7202	orf19.7202	26.06	19.77	19.73	25.98	-0.002899261	NA	Ortholog(s) have role in ER to Golgi vesicle-mediated transport, protein retention in ER lumen, retrograde vesicle-mediated transport, Golgi to ER and COPI-coated vesicle, ER to Golgi transport vesicle, Golgi cisterna localization
orf19.1724	orf19.1724	28.2	19.77	16.79	13.84	-0.317665759	NA	Protein of unknown function; induced by nitric oxide independent of Yhb1p
orf19.4010	PAN3	26.02	19.75	20.59	20.58	-0.073991451	NA	Ortholog(s) have poly(A)-specific ribonuclease activity, role in mRNA 3'-end processing, postreplication repair and PAN complex, cytosol, nucleus localization
orf19.5575	orf19.5575	26.74	19.73	19.57	17.62	-0.163779724	NA	Putative peripheral peroxisomal membrane peroxin; required for regulating peroxisome size and maintenance; Spider biofilm induced
orf19.6479	SEC1	22.73	19.72	27.23	23.13	0.110542277	NA	Ortholog(s) have SNARE binding activity and role in positive regulation of vesicle fusion, vesicle docking involved in exocytosis

orf19.2697	orf19.2697	19.62	19.68	19.57	18.54	-0.025389788	NA	Ortholog(s) have role in protein monoubiquitination, regulation of dipeptide transmembrane transport by regulation of transcription from RNA polymerase II promoter and cytoplasm, nucleus localization
orf19.6809	orf19.6809	19.49	19.67	20.79	25.06	0.117478678	NA	Putative phosphomutase-like protein; protein present in exponential and stationary growth phase yeast; Hap43-repressed; Spider biofilm repressed
orf19.3817	orf19.3817	23.39	19.66	23.59	17.22	-0.043430546	NA	Ortholog(s) have cytoplasm, nucleolus localization
orf19.6671	LAP4	23.94	19.66	28.57	22.16	0.108035697	NA	Protein similar to aminopeptidase I; mutation confers hypersensitivity to amphotericin B; transcript regulated in macrophage response; flow model biofilm induced
orf19.5039	RRP42	23.95	19.65	19.4	14.08	-0.211671178	NA	Putative exosome non-catalytic core component; involved in 3'-5' RNA processing; rat catheter biofilm induced
orf19.1999	orf19.1999	19.31	19.64	16.27	21.57	-0.023173584	NA	Protein of unknown function; transcript detected on high-resolution tiling arrays
orf19.6255	orf19.6255	25.89	19.62	21.28	23.4	-0.038282311	NA	Putative protein of unknown function; Hap43p-repressed gene
orf19.50	orf19.50	24	19.6	21.33	19.55	-0.058755552	NA	Ortholog(s) have role in SCF-dependent proteasomal ubiquitin-dependent protein catabolic process, cellular response to DNA damage stimulus, cellular response to methylmercury and SCF ubiquitin ligase complex, cytosol, nucleus localization
orf19.7441	orf19.7441	29.18	19.58	23.81	20.34	-0.088619293	NA	Ortholog(s) have role in cellular lipid metabolic process, mRNA export from nucleus, protein export from nucleus and nuclear envelope localization

orf19.1877	orf19.1877	33.56	19.57	23.94	25.09	-0.080053957	NA	Ortholog(s) have cytoplasm, nucleus localization
orf19.7063	THG1	31.36	19.56	15.52	21.39	-0.240389078	NA	tRNA guanylyltransferase, with role in tRNA modification; catalyzes reverse (3'-5') nucleotide polymerization
orf19.1217	orf19.1217	22.86	19.56	21.54	16.23	-0.098039301	NA	Ortholog(s) have ribosome binding, ubiquitin-protein ligase activity and role in chromatin modification, chromatin silencing at telomere, protein ubiquitination, ribosome-associated ubiquitin-dependent protein catabolic process
orf19.5608	orf19.5608	31.54	19.56	22.92	21.4	-0.134116343	NA	RNA polymerase III subunit; Spider biofilm induced
orf19.6694	orf19.6694	24.41	19.54	22.01	16.41	-0.11502182	NA	Ortholog of C. dubliniensis CD36 : Cd36_73320, C. parapsilosis CDC317 : CPAR2_703860, Candida tenuis NRRL Y-1498 : CANTEDRAFT_114480 and Debaryomyces hansenii CBS767 : DEHA2C13794g
orf19.2097	orf19.2097	22.99	19.53	22.59	21.56	0.028550593	NA	Predicted DNA-dependent ATPase/helicase; Spider biofilm induced
orf19.1795	PUF3	20.33	19.53	23.5	22.24	0.081349044	NA	Ortholog os S. cerevisiae Puf3; mRNA-binding protein involved in RNA catabolism; mutant is viable
orf19.6526	orf19.6526	18.87	19.53	19.92	22.57	0.065833425	NA	Ortholog(s) have COPI-coated vesicle, Golgi apparatus localization
orf19.2063	orf19.2063	24.35	19.52	19.11	14.98	-0.191720516	NA	Ortholog of C. parapsilosis CDC317 : CPAR2_213140, C. dubliniensis CD36 : Cd36_15600, Lodderomyces elongisporus NRLL YB-4239 : LELG_01226 and Debaryomyces hansenii CBS767 : DEHA2B02442g

orf19.6996	MNN14	18.88	19.5	23.49	21.42	0.100918371	NA	Predicted alpha-1,3-mannosyltransferase activity with a role in protein glycosylation; Hap43-repressed; Spider biofilm induced
orf19.6956	DAL9	20.65	19.5	19.79	17.64	-0.043597905	NA	Putative allantoate permease; fungal-specific (no human or murine homolog)
orf19.1219	orf19.1219	20.88	19.47	19.58	17.86	-0.043597905	NA	Ortholog of Spathaspora passalidarum NRRL Y-27907 : SPAPADRAFT_149713
orf19.5053	orf19.5053	17.04	19.46	18.45	22.56	0.086899842	NA	Ortholog(s) have acetyltransferase activity, chromatin binding activity
orf19.1235	HOM3	28.2	19.44	25.85	23.69	0.044243394	NA	Putative L-aspartate 4-P-transferase; fungal-specific (no human or murine homolog); regulated by Gcn2 and Gcn4; early-stage flow model biofilm induced
orf19.4003	TIP20	19.6	19.44	29.09	25.24	0.245561236	NA	Protein interacting with Sec20p, possibly involved in retrograde transport between the Golgi and the endoplasmic reticulum; similar to S. cerevisiae Tip20p
orf19.2068	orf19.2068	22.59	19.41	25.11	22.87	0.096668736	NA	Ortholog of C. dubliniensis CD36 : Cd36_15550, C. parapsilosis CDC317 : CPAR2_213190, Candida tenuis NRRL Y-1498 : CANTEDRAFT_119026 and Debaryomyces hansenii CBS767 : DEHA2G08514g
orf19.7449	orf19.7449	21.46	19.4	13.55	16.02	-0.200685608	NA	Ortholog(s) have role in mitochondrial genome maintenance, plasmid maintenance and cytosol, mitochondrion localization
orf19.1311	SPO75	22.32	19.39	16.57	16.63	-0.137796294	NA	Ortholog(s) have role in ascospore wall assembly

orf19.1712	GEA2	21.56	19.38	27.57	24.95	0.19428014	NA	Putative ARF GTP/GDP exchange factor; induced in low iron; flow model biofilm repressed
orf19.3315	CTA9	23.45	19.37	22.37	19.75	-0.005988158	NA	Protein required for normal filamentous growth; induced during the mating process; mRNA binds She3; activates transcription in 1-hybrid assay in S. cerevisiae
orf19.4273	orf19.4273	25.7	19.35	20.77	17.23	-0.130325725	NA	Putative mitochondrial membrane protein; ortholog of S. cerevisiae Sls1; coordinates expression of mitochondrially-encoded genes; Hap43-induced
orf19.4252	orf19.4252	17.22	19.34	16.33	12.51	-0.149930638	NA	Ortholog(s) have protein serine/threonine kinase activity
orf19.1290	XKS1	21.54	19.33	27.77	25.06	0.19428014	NA	Putative xylulokinase; Hap43-repressed; induced by prostaglandins; rat catheter biofilm repressed
orf19.2770	orf19.2770	26.65	19.33	28.29	21.42	0.042869594	NA	Ortholog of C. dubliniensis CD36 : Cd36_42220, C. parapsilosis CDC317 : CPAR2_500340, Debaryomyces hansenii CBS767 : DEHA2G17754g and Pichia stipitis Pignal : PICST_31424
orf19.4305.1	orf19.4305.1	17.06	19.3	17.46	15.66	-0.066488182	NA	Ortholog(s) have RNA binding activity, role in mRNA splicing, via spliceosome and U4/U6 x U5 tri-snRNP complex, U6 snRNP, nucleolus localization
orf19.1773	RAP1	24.94	19.28	22.27	17.05	-0.095345387	NA	Transcription factor; binds telomeres and regulatory sequences in DNA; involved in telomere maintenance; represses hyphal growth under yeast-favoring conditions; similar to (but shorter than) S. cerevisiae Rap1
orf19.5485	MEC3	28.14	19.28	19.82	27.07	-0.001573601	NA	Putative DNA damage and meiotic pachytene checkpoint protein; increased transcription is observed upon benomyl treatment

orf19.3946	orf19.3946	21.63	19.27	17.3	20.28	-0.078197338	NA	Ortholog(s) have membrane insertase activity, role in protein insertion into mitochondrial membrane from inner side, respiratory chain complex IV assembly and integral to mitochondrial inner membrane localization
orf19.3174	CDC24	23.57	19.2	28.66	22.41	0.125684281	NA	GDP-GTP exchange factor for Cdc42p; phosphorylated; required for maintenance of hyphal growth; misexpression blocks hyphal growth and causes avirulence in a mouse model of systemic infection; antigenic during human oral infection
orf19.3405	ZCF18	22.47	19.18	19.02	19.13	-0.060680508	NA	Putative Zn(II)2Cys6 transcription factor; heterozygous null mutant displays sensitivity to virgineone and decreased colonization of mouse kidneys
orf19.4392	DEM1	17.57	19.18	21.56	21.55	0.120060822	NA	Putative mitochondrial exonuclease; alpha-factor induced
orf19.123	RCN1	19.79	19.18	21.26	21.25	0.048172971	NA	Protein involved in calcineurin-dependent signaling that controls stress response and virulence; inhibits calcineurin function
orf19.2563	orf19.2563	27.82	19.17	24.54	21.18	-0.021810215	NA	Ortholog(s) have U6 snRNA binding activity, role in spliceosomal complex assembly, spliceosomal tri-snRNP complex assembly and U6 snRNP localization
orf19.6805	orf19.6805	18.4	19.17	15.24	14.78	-0.146702897	NA	Protein of unknown function; Spider biofilm induced
orf19.6788	orf19.6788	30.56	19.16	23.25	25.56	-0.018043053	NA	Protein with a predicted role in cotranslational protein targeting to membrane; induced during chlamydospore formation in both C. albicans and C. dubliniensis
orf19.7369	TRM12	20.35	19.15	11.26	14.36	-0.291303493	NA	Ortholog(s) have S-adenosylmethionine-dependent methyltransferase activity, transferase activity, transferring alkyl or aryl (other than methyl) groups activity and role in tRNA methylation, wybutosine biosynthetic process

orf19.91	orf19.91	22.73	19.14	23.08	27.06	0.130642939	NA	Protein of unknown function; flow model biofilm induced; Hap43-repressed
orf19.4903	orf19.4903	28.53	19.13	26.15	24.91	0.044152541	NA	Ortholog(s) have endoplasmic reticulum localization
orf19.7205	DUR7	18	19.13	19.42	17.65	-0.007205115	NA	Putative urea transporter; fungal-specific (no human or murine homolog)
orf19.2792	IST2	14.49	19.12	30.38	28.12	0.418395274	NA	Ortholog(s) have lipid binding activity, role in endoplasmic reticulum membrane organization, regulation of phosphatidylinositol dephosphorylation and cellular bud membrane, cortical endoplasmic reticulum localization
orf19.301	PGA18	14.21	19.1	23.38	21.25	0.194719923	NA	Putative GPI-anchored protein; regulated by Nrg1, Tup1; rat catheter biofilm repressed
orf19.474	orf19.474	27.72	19.09	21.2	21.47	-0.089228715	NA	Ortholog(s) have role in mRNA export from nucleus, nuclear envelope organization, protein export from nucleus and endoplasmic reticulum, nuclear envelope localization
orf19.3923	PGA37	8.61	19.07	4.3	3.68	-0.522523305	NA	Putative GPI-anchored protein; Hap43-repressed; Spider biofilm induced
orf19.1054	orf19.1054	24.66	19.02	37.95	37.46	0.449200246	0.617445	Ortholog(s) have structural constituent of nuclear pore activity
orf19.5219	IRA2	19.09	19.01	20	20.91	0.048888776	NA	GTPase-activating protein; negatively regulates RAS by converting it from the GTP- to the GDP-bound inactive form; Spider biofilm induced; flow model biofilm repressed

orf19.3114	orf19.3114	23.37	19	17.93	19.97	-0.077359871	NA	Predicted pseudouridine synthase; Spider biofilm induced
orf19.1491	orf19.1491	14.97	18.99	14.43	12.62	-0.153753536	NA	Ortholog(s) have RNA binding activity, role in mRNA splicing, via spliceosome and U1 snRNP, U2-type prespliceosome localization
orf19.4332	orf19.4332	16.21	18.99	15.75	27.59	0.156741225	NA	Ortholog of C. dubliniensis CD36 : Cd36_52800, C. parapsilosis CDC317 : CPAR2_303110, Candida tenuis NRRL Y-1498 : CANTEDRAFT_92321 and Debaryomyces hansenii CBS767 : DEHA2G07964g
orf19.5513	PRP45	25.78	18.94	17.58	14.5	-0.226863474	NA	Protein required for pre-mRNA splicing; Spider biofilm induced
orf19.7181	orf19.7181	21.17	18.92	24.24	20.99	0.081413219	NA	Ortholog of C. dubliniensis CD36 : Cd36_73670, C. parapsilosis CDC317 : CPAR2_702370, Candida tenuis NRRL Y-1498 : CANTEDRAFT_119320 and Debaryomyces hansenii CBS767 : DEHA2G16632g
orf19.6072	CDC13	23.49	18.9	18.3	17.18	-0.135994706	NA	Essential protein with similarity to S. cerevisiae Cdc13p, involved in telomere maintenance
orf19.1867	orf19.1867	21.09	18.89	9.5	7.47	-0.54033832	NA	Putative malate permease; induced during macrophage infection; regulated by Gcn2 and Gcn4; putative peroxisome targeting signal; Hap43-repressed; Spider biofilm induced
orf19.718	RRN11	21.83	18.89	15.64	16.26	-0.178104468	NA	Putative RNA polymerase I subunit; rat catheter biofilm induced; Spider biofilm induced
orf19.5711	orf19.5711	25.13	18.87	30.71	27.73	0.231963522	0.986773	Putative phosphatidylinositol transfer protein; possibly an essential gene, disruptants not obtained by UAU1 method

orf19.1169	orf19.1169	33.78	18.87	17.64	20.8	-0.232335086	NA	Putative DnaJ-like molecular chaperone; Spider biofilm induced
orf19.177	orf19.177	21.64	18.86	28.41	19.57	0.111640546	NA	Has domain(s) with predicted phosphatidylinositol binding activity and role in cell communication
orf19.5965	orf19.5965	24.95	18.85	29.99	27.08	0.202468132	NA	Ortholog(s) have ubiquitin-ubiquitin ligase activity, role in ER-associated ubiquitin-dependent protein catabolic process, protein ubiquitination, response to stress and cytoplasm, nucleus localization
orf19.3878	orf19.3878	20.97	18.85	28.82	19.59	0.14515463	NA	Protein of unknown function; flow model biofilm induced
orf19.7425	orf19.7425	18.15	18.84	27.76	25.81	0.28320749	NA	Ortholog(s) have uracil DNA N-glycosylase activity, role in DNA repair and mitochondrion, nucleus localization
orf19.4006	orf19.4006	26.84	18.83	21.45	22.14	-0.055084521	NA	Ortholog(s) have cytosol, nucleus localization
orf19.3963	orf19.3963	22.08	18.82	22.23	16.83	-0.043705811	NA	Ortholog(s) have mitochondrion localization
orf19.2907.1	orf19.2907.1	32.04	18.8	21.54	19.44	-0.169949911	NA	Ortholog(s) have chromatin DNA binding activity, role in positive regulation of transcription from RNA polymerase II promoter by pheromones, threonylcarbamoyladenosine metabolic process and EKC/KEOPS complex, nuclear chromatin localization
orf19.1519	orf19.1519	20.2	18.8	18.48	18.69	-0.043307743	NA	Protein of unknown function; Hap43-repressed gene

orf19.6191	TLO8	0.01	18.79	17.1	24.64	0.118907726	NA	Member of a family of telomere-proximal genes of unknown function; may be spliced in vivo
orf19.6163	CSE4	16.06	18.78	16.62	16.91	-0.02709558	NA	Centromeric histone H3 variant; role in structural changes of centromeric nucleosomes during cell cycle; 4 nucleosomes bind 1 centromere, each has 1 Cse4 molecule during most of cell cycle, 2 molecules in anaphase; Spider biofilm repressed
orf19.994	orf19.994	20.91	18.78	20.15	16.96	-0.062796606	NA	Protein of unknown function; induced by Mnl1 under weak acid stress; flow model biofilm repressed
orf19.428	orf19.428	22.6	18.78	20.51	16.45	-0.098537204	NA	Putative serine/threonine kinase; induced during planktonic growth; rat catheter biofilm repressed
orf19.7317	UGA33	20.59	18.78	17.07	17.7	-0.099870702	NA	Zinc-finger transcription factor; similar to S. cerevisiae Uga3 which regulates gamma-aminobutyrate metabolism gene expression; required for yeast cell adherence to silicone substrate; rat catheter biofilm induced
orf19.2040	orf19.2040	21.07	18.76	16.1	20.9	-0.05946431	NA	Ortholog(s) have oxidoreductase activity, acting on NAD(P)H activity, role in iron-sulfur cluster assembly, nitric oxide biosynthetic process, oxidation-reduction process and mitochondrion localization
orf19.520	orf19.520	19.21	18.74	21.18	15.57	-0.027115079	NA	Ortholog of C. dubliniensis CD36 : Cd36_29660, C. parapsilosis CDC317 : CPAR2_205200, Candida tenuis NRRL Y-1498 : CANTEDRAFT_96118 and Debaryomyces hansenii CBS767 : DEHA2F21428g
orf19.6079	orf19.6079	21.04	18.72	26.86	20.98	0.130530872	NA	Predicted ORF in retrotransposon Tca8 with similarity to the Gag region encoding nucleocapsid-like protein; repressed by ciclopirox olamine; filament induced; regulated by Rfg1, Tup1; overlaps orf19.6078.1
orf19.5860	orf19.5860	13.8	18.72	3.44	4.42	-0.677471094	NA	Predicted ORF from Assembly 19; removed from Assembly 20; subsequently reinstated in Assembly 21 based on comparative genome analysis

orf19.6976	orf19.6976	23.25	18.72	15.96	14.35	-0.237916172	NA	Predicted MFS membrane transporter; member of the proton coupled folate transporter/heme carrier protein family; virulence-group-correlated expression; Spider biofilm induced
orf19.4110	orf19.4110	20.1	18.69	19.7	17.87	-0.025541586	NA	Ortholog of C. dubliniensis CD36 : Cd36_20490, C. parapsilosis CDC317 : CPAR2_104180, Candida tenuis NRRL Y-1498 : CANTEDRAFT_116074 and Debaryomyces hansenii CBS767 : DEHA2F05148g
orf19.3308	STB5	19.65	18.68	21.33	22.09	0.066137922	NA	Putative transcription factor with zinc cluster DNA-binding motif; has similarity to S. cerevisiae Stb5p; Hap43p-repressed gene
orf19.5992	WOR2	25.91	18.66	50.71	41.8	0.638757821	0.24345	Zn(II)2Cys6 transcription factor; regulator of white-opaque switching; required for maintenance of opaque state; Hap43-induced
orf19.1026.1	orf19.1026.1	29.52	18.64	15.5	29.43	-0.078103063	NA	Ortholog of C. dubliniensis CD36 : Cd36_03580, C. parapsilosis CDC317 : CPAR2_105090, Candida tenuis NRRL Y-1498 : CANTEDRAFT_106351 and Debaryomyces hansenii CBS767 : DEHA2A09548g
orf19.5190	orf19.5190	19.11	18.64	32.54	38.57	0.519558726	0.488168	Ortholog of Candida albicans WO-1 : CAWG_05610
orf19.27	orf19.27	20.45	18.64	24.75	21.29	0.116111499	NA	Ortholog of C. dubliniensis CD36 : Cd36_20860, C. parapsilosis CDC317 : CPAR2_104250, Candida tenuis NRRL Y-1498 : CANTEDRAFT_115100 and Debaryomyces hansenii CBS767 : DEHA2E10450g
orf19.4412	orf19.4412	20.79	18.64	20.29	17.98	-0.043597905	NA	Ortholog(s) have DNA-directed DNA polymerase activity, deoxycytidyl transferase activity and role in error-free translesion synthesis, error-prone translesion synthesis
orf19.1120	FAV2	19.11	18.64	18.6	22.3	0.049464125	NA	Adhesin-like protein; induced by mating factor in MTLa/a opaque cells, in cyr1 null, in filaments; regulated by Nrg1, Rfg1, Tup1, Tec1, Efg1, Ntd80, Rob1, Brg1; induced in oralpharyngeal candidasis; Spider biofilm induced

orf19.5604	MDR1	17.42	18.62	8.52	4.15	-0.542439385	NA	Plasma membrane MDR/MFS multidrug efflux pump; methotrexate is preferred substrate; overexpression in drug-resistant clinical isolates confers fluconazole resistance; repressed in young biofilms; rat catheter biofilm induced
orf19.1510	SRD1	22.27	18.62	27.22	16.01	0.025707905	NA	Sur7 family protein with PalI-like domains; not required for stress responses; mutant is viable
orf19.539	LAP3	22.15	18.61	24.39	24.87	0.131632143	NA	Putative aminopeptidase; positively regulated by Sfu1; clade-associated gene expression; virulence-group-correlated expression; induced by alpha pheromone in SpiderM medium; Hap43-induced; Spider and flow model biofilm induced
orf19.7061	orf19.7061	22.83	18.6	17.71	18.26	-0.116191938	NA	Ortholog(s) have ATPase activity and cytosol, nucleus localization
orf19.2008	orf19.2008	22.89	18.55	18.21	19.09	-0.096640518	NA	Ortholog(s) have S-methyl-5-thioribose-1-phosphate isomerase activity, role in L-methionine salvage from methylthioadenosine and cytosol, nucleus localization
orf19.1698	orf19.1698	21.37	18.53	28.22	25.29	0.212262846	NA	Ortholog(s) have phosphatidate phosphatase activity, role in actin cytoskeleton organization, cellular lipid metabolic process and actin cortical patch localization
orf19.5496	AVT1	23.82	18.5	24.91	22.25	0.062297917	NA	Putative vacuolar transporter; promoter bound by a1p and alpha2p by ChIP-chip analysis
orf19.3523	CRK1	22.13	18.49	21.24	20.49	0.012621417	NA	Protein kinase of the Cdc2 subfamily involved in hyphal development, virulence; promotes hyphal development independently of Cph1 and Efg1; functionally complements pheromone hypersensitivity of S. cerevisiae sgv1 mutant; Hap43p-repressed
orf19.6275	orf19.6275	22.97	18.48	17.35	19.85	-0.077261065	NA	Ortholog(s) have cytosol localization

orf19.1383	orf19.1383	23.25	18.48	26.04	20.14	0.079903294	NA	Protein of unknown function; induced by alpha pheromone in SpiderM medium
orf19.5871	SNF5	23.62	18.48	30.7	22.75	0.189697522	NA	SWI/SNF chromatin remodeling complex subunit involved in transcriptional regulation; mutants have defects in silicone adherence, biofilm formation, hyphal morphogenesis, cell wall defects; increased cell aggregation during yeast form growth
orf19.5539	orf19.5539	18.7	18.47	15.28	19.99	-0.042358775	NA	Ortholog(s) have role in retrograde vesicle-mediated transport, Golgi to ER and SNARE complex, integral to cytosolic side of endoplasmic reticulum membrane localization
orf19.5924	ZCF31	17.93	18.46	19.12	11.66	-0.107848197	NA	Zn(II)2Cys6 transcription factor of unknown function; mutant is sensitive to copper and SDS, and resistant to Calcofluor White; required for yeast cell adherence to silicone substrate
orf19.2059	orf19.2059	24.49	18.46	15.94	16.76	-0.173513376	NA	Protein with homology to magnesium-dependent endonucleases and phosphatases; regulated by Sef1, Sfu1, and Hap43; Spider biofilm induced
orf19.2565	orf19.2565	19.05	18.45	22.55	18.81	0.084343947	NA	Predicted intermediate filament protein; required for nuclear and mitochondrial transmission to daughter buds in S. cerevisiae; Spider biofilm induced
orf19.2507	ARP9	24.6	18.44	18.66	20.43	-0.075467066	NA	Protein similar to S. cerevisiae Arp3p, a component of the Arp2/3 complex involved in actin-dependent processes; likely to be essential for growth, based on an insertional mutagenesis strategy
orf19.3720	orf19.3720	20.23	18.43	16.26	14.89	-0.144105279	NA	Ortholog(s) have endo-1,4-beta-xylanase activity and SCF ubiquitin ligase complex, cytoplasm localization
orf19.259	orf19.259	26.51	18.43	17.63	13.34	-0.265501758	NA	Protein with a predicted role in 60S ribosomal subunit assembly; flow model biofilm induced

orf19.6010	CDC5	20.58	18.4	24.1	17.88	0.046653894	NA	Polo-like kinase; member of conserved Mcm1 regulon; depletion causes defects in spindle elongation and Cdc35-dependent filamentation; virulence-group-correlated expression; likely essential (UAU1 method); Spider biofilm repressed
orf19.5892	orf19.5892	19.56	18.39	37.24	35.85	0.534290701	0.458126	Ortholog of S. cerevisiae Hul4; similar to hect domain E3 ubiquitin-protein ligases; Hap43-repressed gene
orf19.6533	MSK1	24.75	18.38	23.64	19.48	-0.005920085	NA	Putative mitochondrial lysine-tRNA synthetase; flucytosine repressed
orf19.3245	orf19.3245	20.85	18.38	22.96	14.94	-0.026634963	NA	Ortholog of C. dubliniensis CD36 : Cd36_26090, C. parapsilosis CDC317 : CPAR2_801510, Candida tenuis NRRL Y-1498 : CANTEDRAFT_137093 and Debaryomyces hansenii CBS767 : DEHA2F20548g
orf19.4441	orf19.4441	20.03	18.37	21.72	20.48	0.066570617	NA	Ortholog(s) have DNA replication origin binding, double-stranded DNA binding, single-stranded DNA binding activity
orf19.2443	RGD1	19.84	18.37	20.6	16.87	-0.007161782	NA	GTPase activator protein; transcript induced in low iron; alkaline upregulated; localized to the bud emergence area in G1 phase and in the bud tip during S and G2 phases
orf19.349	orf19.349	25.47	18.35	26.01	24.25	0.112145056	NA	Ortholog(s) have role in aerobic respiration, mRNA metabolic process and mitochondrial outer membrane localization
orf19.2365	POL2	17.74	18.34	17.47	17.66	-0.025377662	NA	DNA polymerase epsilon; transcript induced by interaction with macrophage; transcript is regulated by Tup1; periodic mRNA expression, peak at cell-cycle G1/S phase
orf19.3219	orf19.3219	21.47	18.28	27.86	24.68	0.231034655	NA	Ortholog of S. cerevisiae Sia1; involved in activation of the Pma1 plasma membrane H+-ATPase by glucose in S. cerevisiae; Spider biofilm induced

orf19.1458	orf19.1458	18.48	18.24	15.4	14.46	-0.148823416	NA	Ortholog(s) have tRNA-specific adenosine deaminase activity, role in tRNA modification and cytoplasm, nucleus localization
orf19.4523	orf19.4523	19.17	18.24	24.36	16.34	0.046522962	NA	Ortholog(s) have 5-formyltetrahydrofolate cyclo-ligase activity, role in folic acid-containing compound biosynthetic process and mitochondrion localization
orf19.3289	orf19.3289	20.83	18.24	24.55	18.88	0.081665348	NA	Phosphorylated protein of unknown function
orf19.2078	orf19.2078	19.06	18.23	19.29	17.54	-0.006416845	NA	Ortholog(s) have role in Golgi to vacuole transport and AP-1 adaptor complex localization
orf19.7660	VPS52	19.77	18.22	25.03	20.06	0.117675532	NA	Protein required for hyphal growth; has similarity to S. cerevisiae Vps52p
orf19.7497	orf19.7497	20.65	18.22	18.39	17.45	-0.081797332	NA	Ortholog(s) have ubiquitin-protein ligase activity, role in protein ubiquitination involved in ubiquitin-dependent protein catabolic process and Cul3-RING ubiquitin ligase complex, cytoplasm, nucleus localization
orf19.4983	orf19.4983	28.2	18.17	26.73	27.88	0.139423557	NA	Ortholog of C. dubliniensis CD36 : Cd36_12520, C. parapsilosis CDC317 : CPAR2_201720, Candida tenuis NRRL Y-1498 : CANTEDRAFT_108003 and Debaryomyces hansenii CBS767 : DEHA2D05214g
orf19.3487	orf19.3487	20	18.17	21.15	12.69	-0.084783918	NA	Ortholog of C. dubliniensis CD36 : Cd36_33330, C. parapsilosis CDC317 : CPAR2_701980, Debaryomyces hansenii CBS767 : DEHA2C15840g and Pichia stipitis Pignal : PICST_31066
orf19.1082	orf19.1082	17.04	18.17	12.09	13.21	-0.21705809	NA	Protein with an Alba DNA/RNA-binding protein domain; Spider biofilm induced

orf19.5412	orf19.5412	19.91	18.17	23.14	18.64	0.06579098	NA	Ortholog of S. cerevisiae : ECM9, C. glabrata CBS138 : CAGL0L02255g, C. dubliniensis CD36 : Cd36_80470, C. parapsilosis CDC317 : CPAR2_503390 and Candida tenuis NRRL Y-1498 : CANTEDRAFT_133107
orf19.6296	VPS22	21.33	18.17	20.31	16.67	-0.043945434	NA	ESCRT-II complex protein with a role in multivesicular body (MVB) trafficking; required for processing of Rim8p; Hap43p-repressed gene
orf19.1418	SEC15	19.19	18.15	26.07	21.39	0.170116315	NA	Predicted exocyst subunit; cellular bud tip-associated protein; ortholog of S. cerevisiae SEC15
orf19.6337	TLO13	33.57	18.13	21.04	29.32	-0.0298656	NA	Member of a family of telomere-proximal genes of unknown function; may be spliced in vivo; overlaps orf19.6337.1, which is a region annotated as blocked reading frame
orf19.1326	orf19.1326	19.82	18.13	18.27	15.83	-0.083568966	NA	Ortholog of Candida albicans WO-1 : CAWG_03455
orf19.7443	orf19.7443	16.64	18.12	12.14	20.3	-0.062055221	NA	Ortholog of C. dubliniensis CD36 : Cd36_86510, C. parapsilosis CDC317 : CPAR2_206110, Candida tenuis NRRL Y-1498 : CANTEDRAFT_112184 and Debaryomyces hansenii CBS767 : DEHA2F17292g
orf19.826	MED1	21.07	18.06	25.19	24.98	0.184318832	NA	RNA polymerase II mediator complex subunit; RNA polymerase II transcription cofactor
orf19.6744	orf19.6744	14.76	18.06	25.96	24.4	0.297071509	NA	Ortholog(s) have role in regulation of mitotic metaphase/anaphase transition and cytosol, nucleus localization
orf19.6563	KCH1	15.24	18.04	15.76	15.48	-0.048416853	NA	Ortholog of Kch1 a potassium transporter; mediates K+ influx and activates high-affinity Ca2+ influx system during mating pheromone response in S. cerevisiae; induced by alpha pheromone in SpiderM medium

orf19.5856	orf19.5856	20.59	18.04	18.19	19.55	-0.023274401	NA	Membrane-localized protein of unknown function
orf19.1204	orf19.1204	23.35	18.02	22.56	16.04	-0.0433822	NA	Phosphorylated protein of unknown function; transcript is upregulated clinical isolates from HIV positive patients with oral candidiasis
orf19.3241	orf19.3241	18.96	18.01	23	21.57	0.13790983	NA	Ortholog(s) have Seh1-associated complex, extrinsic to fungal-type vacuolar membrane localization
orf19.1883	YCS4	19.42	17.95	20.72	17.33	0.011359932	NA	Putative condensin complex subunit; cell-cycle regulated periodic mRNA expression
orf19.7194	orf19.7194	26.53	17.95	20.55	15.66	-0.147798283	NA	Protein required for virulence in reconstituted human epithelium (RHE) model of ex vivo infection; decreased transcription is observed upon fluphenazine treatment; induced upon adherence to polystyrene
orf19.4774	AOX1	21.74	17.95	17.41	17.9	-0.098949212	NA	Alternative oxidase; low abundance; constitutively expressed; one of two isoforms (Aox1p and Aox2p); involved in a cyanide-resistant respiratory pathway present in plants, protists, and some fungi, absent in S. cerevisiae; Hap43p-repressed
orf19.4900	MNN12	16.32	17.94	13.02	7.99	-0.292465992	NA	Predicted alpha-1,3-mannosyltransferase activity with a role in protein glycosylation
orf19.2445	orf19.2445	14.56	17.94	15.76	12.6	-0.09102379	NA	Putative dicarboxylic amino acid permease; fungal-specific (no human or murine homolog); induced by alpha pheromone in SpiderM medium
orf19.6009	SET1	20.97	17.9	20.97	18.96	0.012221011	NA	Lysine histone methyltransferase; methylates histone H3 K4; regulates of white-opaque switch, epithelial cell adhesion, agar-embedded filamentation, virulence in mice; unique N-terminus immunogenic in human; rat catheter biofilm repressed

orf19.5138	IFA21	17.87	17.87	24.14	20.41	0.138263593	NA	IPF family A protein; mutants are viable; Spider biofilm induced
orf19.4369	orf19.4369	19.42	17.86	19.22	17.14	-0.025916963	NA	Ortholog of S. cerevisiae Spp41; protein involved in negative regulation of expression of spliceosome components PRP4 and PRP3 in S. cerevisiae; mutants are viable
orf19.6366	orf19.6366	16.6	17.85	17.81	20.05	0.051286511	NA	Has domain(s) with predicted RNA binding activity
orf19.1762	OCA1	19.26	17.85	23.89	18.5	0.101552565	NA	Putative protein phosphatase of the PTP family (tyrosine-specific); ortholog of S. cerevisiae Oca1; mutant is viable; mutant shows virulence defect
orf19.2669	orf19.2669	15.99	17.84	18.77	16.45	0.011479328	NA	ORF in retrotransposon Tca4; similar to Pol region of retrotransposons encoding reverse transcriptase, protease, integrase; downstream of RHD2, similar to the Gag region encoding nucleocapsid-like protein; rat catheter biofilm induced
orf19.3407	RAD18	23.61	17.83	16.96	16.79	-0.154083909	NA	Putative transcription factor with zinc finger DNA-binding motif; Hap43p-repressed gene
orf19.4063	GPT1	16.76	17.83	15.65	16.71	-0.045887192	NA	GABA/polyamine transporter; 9 to 11 membrane spanning segments; complements GABA uptake defect of an S. cerevisiae uga4 put4 gap1 triple mutant; complements growth of an S. cerevisiae spe1 mutant under polyamine limitation
orf19.5367	RDH54	17.52	17.83	20.56	15.47	-0.008541335	NA	Putative DNA-dependent ATPase with a predicted role in DNA recombination and repair; transcriptionally induced by interaction with macrophages
orf19.305	orf19.305	21.7	17.8	28.5	27.45	0.259179284	NA	Protein of unknown function; upregulated in a cyr1 null mutant

orf19.7251	WSC4	16.29	17.8	28.96	21.39	0.27413433	NA	Putative cell wall integrity and stress response subunit 4 precursor; transcription is specific to white cell type
orf19.7548	MED21	25.04	17.77	24.97	23.87	0.096329529	NA	Ortholog(s) have RNA polymerase II repressing transcription factor binding, RNA polymerase II transcription coactivator activity, RNA polymerase II transcription corepressor activity
orf19.5618	orf19.5618	22.97	17.77	29.7	28.18	0.270876515	NA	Ortholog(s) have SNARE binding, unfolded protein binding activity
orf19.4418	FMT1	16.2	17.76	18.41	12.5	-0.06932168	NA	Putative methionyl-tRNA transformylase; induced upon adherence to polystyrene
orf19.3865	RFX1	18.04	17.74	16.76	14.53	-0.086434836	NA	Transcription factor; involved in DNA damage responses, morphogenesis, and virulence; Spider biofilm induced
orf19.326	orf19.326	22.51	17.74	34.97	24.6	0.295510746	NA	Protein of unknown function; Spider biofilm repressed
orf19.4697	MDN1	15.46	17.73	16.39	19.23	0.033125287	NA	Putative midasin, a very large dynein-related AAA-type ATPase; induced during the mating process
orf19.2676	orf19.2676	23.65	17.73	18.32	15.79	-0.154817665	NA	Predicted hexameric RecA-like ATPase Elp456 Elongator subcomplex subunit; required for modification of wobble nucleosides in tRNA; Spider biofilm induced
orf19.2673	orf19.2673	23.46	17.73	27.09	18.05	0.061564142	NA	Ortholog(s) have role in DNA repair and Smc5-Smc6 complex, cytoplasm, nucleus localization

orf19.5214	VPS33	17.02	17.72	20.85	18.03	0.068151392	NA	Ortholog(s) have ATP binding, phosphatidylinositol binding activity
orf19.567	TFB3	17.89	17.71	20.61	15.75	0.010894358	NA	Putative C3HC4 zinc finger transcription factor; transcript positively regulated by Tbf1; Spider biofilm induced
orf19.7212	orf19.7212	22.36	17.71	20.71	21.33	0.030824445	NA	Phosphorylated protein; homozygous transposon insertion causes decreased colony wrinkling in filamentous growth-inducing conditions, but does not block hyphal formation in liquid media; Hap43-repressed
orf19.2579	orf19.2579	19.74	17.67	18.39	18.23	-0.043736009	NA	Msh4 ortholog of S. cerevisiae; involved in meiotic recombination and is required for normal levels of crossing over; Spider biofilm induced
orf19.7615	orf19.7615	18.67	17.64	16.32	14.78	-0.125534391	NA	Protein involved in endoplasmic reticulum (ER) to Golgi vesicle-mediated transport; putative subunit of the transport protein particle (TRAPP) complex of the cis-Golgi; Spider biofilm induced
orf19.281	orf19.281	23.34	17.58	17.74	16.5	-0.117464711	NA	Ortholog of C. dubliniensis CD36 : Cd36_82880, C. parapsilosis CDC317 : CPAR2_102370, Candida tenuis NRRL Y-1498 : CANTEDRAFT_118463 and Pichia stipitis Pignal : PICST_32241
orf19.5269	orf19.5269	21.59	17.56	19.39	17.78	-0.061269267	NA	Ortholog of C. dubliniensis CD36 : Cd36_11210, C. parapsilosis CDC317 : CPAR2_805180, Candida tenuis NRRL Y-1498 : CANTEDRAFT_116279 and Debaryomyces hansenii CBS767 : DEHA2B10714g
orf19.4867	SWE1	16.25	17.55	18.49	14.6	-0.028065964	NA	Putative protein kinase with a role in control of growth and morphogenesis, required for full virulence; mutant cells are small, rounded, and sometimes binucleate; not required for filamentous growth; mutant is hypersensitive to caspofungin
orf19.5137	orf19.5137	14.35	17.54	23.86	14.84	0.122483338	NA	Protein of unknown function; regulated by Sef1, Sfu1, Hap43; Spider biofilm repressed

orf19.4756	orf19.4756	25.63	17.53	19.96	19.39	-0.093125748	NA	Ortholog of S. cerevisiae : YTP1, C. dubliniensis CD36 : Cd36_08490, C. parapsilosis CDC317 : CPAR2_801590, Candida tenuis NRRL Y-1498 : CANTEDRAFT_109732 and Debaryomyces hansenii CBS767 : DEHA2C10384g
orf19.3821	orf19.3821	20.99	17.51	25.03	19.51	0.099335951	NA	Ortholog of C. dubliniensis CD36 : Cd36_44290, C. parapsilosis CDC317 : CPAR2_500740, Candida tenuis NRRL Y-1498 : CANTEDRAFT_137302 and Debaryomyces hansenii CBS767 : DEHA2F07326g
orf19.268	orf19.268	20.02	17.51	20.24	19.81	0.031234134	NA	Ortholog(s) have role in transcription-coupled nucleotide-excision repair and Ddb1-Ckn1 complex, cytosol localization
orf19.4570	orf19.4570	13.02	17.5	17.46	11.19	-0.052012234	NA	Ortholog of C. dubliniensis CD36 : Cd36_42090, Debaryomyces hansenii CBS767 : DEHA2C14872g, Pichia stipitis Pignal : PICST_74821 and Spathaspora passalidarum NRRL Y-27907 : SPAPADRAFT_58607
orf19.2698	orf19.2698	26.24	17.49	30.53	28.65	0.263691466	NA	Ortholog(s) have role in protein folding in endoplasmic reticulum and ER membrane protein complex localization
orf19.496	orf19.496	15.75	17.47	19.88	21.26	0.145822123	NA	Ortholog(s) have DNA-dependent ATPase activity, dinucleotide insertion or deletion binding, guanine/thymine mispair binding activity, role in mismatch repair, mitochondrial DNA repair and mitochondrion localization
orf19.2266	orf19.2266	20.64	17.46	26.79	18.22	0.115988832	NA	Ortholog(s) have ATPase activity, DNA binding, nucleosome binding activity, role in chromatin remodeling and Isw1 complex localization
orf19.6729	TIP120	18.73	17.45	25.22	23.21	0.20776535	NA	Protein similar to human CAND1 (Cullin-Associated Nedd8-Dissociated) protein involved in regulation of SCF complexes; binds unnedddylated cullin Cdc53; mutants are viable
orf19.3449	orf19.3449	25.72	17.43	20.26	18.77	-0.075106824	NA	Ortholog(s) have ubiquitin-protein ligase activity, role in histone catabolic process, histone ubiquitination and cytoplasm, ribosome localization

orf19.1771	CWC22	23.69	17.4	19.36	16.5	-0.097290894	NA	Predicted spliceosome-associated protein; role in pre-mRNA splicing; Spider biofilm induced
orf19.3751	orf19.3751	22.93	17.38	25.39	19.54	0.081746793	NA	Putative serine/threonine protein kinase; possibly an essential gene, disruptants not obtained by UAU1 method
orf19.6309	MSS11	21	17.37	22.88	23.84	0.154227187	NA	Transcription factor; activator that binds to Flo8 via a LisH motif to cooperatively activate transcription of hypha-specific genes; required for hyphal growth
orf19.4457	BNI4	18.32	17.36	21.51	19.14	0.105562394	NA	Protein required for wild-type cell wall chitin distribution, morphology, hyphal growth; not essential; similar to S. cerevisiae Bni4p (targeting subunit for Glc7p phosphatase, involved in bud-neck localization of chitin synthase III)
orf19.2615	MDL1	17.8	17.35	24.48	18.64	0.140204066	NA	Putative mitochondrial, half-size MDR-subfamily ABC transporter
orf19.3877	orf19.3877	17.05	17.32	17.04	21.71	0.090601517	NA	Ortholog of C. dubliniensis CD36 : Cd36_73360 and Candida albicans WO-1 : CAWG_01936
orf19.1360.1	orf19.1360.1	18.55	17.31	35.77	33.29	0.519995452	0.491076	Putative adhesin-like protein
orf19.2462	PRN3	21.74	17.31	21.87	14.19	-0.063297326	NA	Protein similar to pirin; induced by Mnl1 under weak acid stress; Hap43-repressed; Spider biofilm induced
orf19.3286	orf19.3286	25.17	17.29	24.4	22.71	0.081321988	NA	Ortholog(s) have alpha-glucosidase activity, role in polysaccharide biosynthetic process, protein N-linked glycosylation and endoplasmic reticulum lumen, glucosidase II complex localization

orf19.7237	orf19.7237	19.41	17.27	22.9	18.17	0.085559938	NA	Ortholog(s) have arylformamidase activity and role in NAD biosynthetic process
orf19.3818	GOA1	24.06	17.24	24.45	19.68	0.047196565	NA	Protein required for respiratory growth, resistance to oxidants, chlamydospore formation, hyphal growth under some conditions, and virulence; relocalizes from the cytoplasm to the mitochondrion during oxidative or osmotic stress
orf19.7311	orf19.7311	18.54	17.21	28.73	29.05	0.36526769	NA	Protein of unknown function; Spider biofilm induced
orf19.53	orf19.53	16.89	17.21	17.67	18.49	0.032860249	NA	Ortholog(s) have role in generation of catalytic spliceosome for first transesterification step, mRNA cis splicing, via spliceosome and commitment complex, cytosol localization
orf19.4646	UEC1	14.92	17.18	8.57	11.27	-0.275242079	NA	Protein required for damage to oral epithelial cells and for normal hyphal growth and stress resistance; transcription induced on contact with vascular endothelial cells; not highly conserved
orf19.6858	EDC3	21.41	17.17	21.71	19.65	0.067242541	NA	Ortholog(s) have role in cytoplasmic mRNA processing body assembly, deadenylation-independent decapping of nuclear-transcribed mRNA and cytoplasmic mRNA processing body localization
orf19.1371	orf19.1371	17.3	17.17	14.19	13.33	-0.153003416	NA	Ortholog of C. dubliniensis CD36 : Cd36_23880, C. parapsilosis CDC317 : CPAR2_806590, Candida tenuis NRRL Y-1498 : CANTEDRAFT_92769 and Debaryomyces hansenii CBS767 : DEHA2B13376g
orf19.923	THR1	22.59	17.16	20.79	15.36	-0.080712721	NA	Putative homoserine kinase; regulated by Tup1; amphotericin B repressed; regulated by Gcn2 and Gcn4; Spider biofilm repressed
orf19.1473	orf19.1473	19.23	17.16	13.87	11.81	-0.214209309	NA	2-hydroxyacid dehydrogenase domain-containing protein; Hap43-repressed gene; induced by alpha pheromone in SpiderM medium

orf19.154	orf19.154	21.99	17.14	13.64	14.5	-0.202301191	NA	Putative ortholog of S. cerevisiae Utp30; a U3-containing 90S preribosome complex protein; Hap43-induced; Spider biofilm induced
orf19.6436	orf19.6436	24.74	17.13	23.61	22.01	0.064319524	NA	Ortholog of C. dubliniensis CD36 : Cd36_34210, C. parapsilosis CDC317 : CPAR2_205590, Candida tenuis NRRL Y-1498 : cten_CGOB_00133 and Debaryomyces hansenii CBS767 : DEHA2E02178g
orf19.2326	orf19.2326	18.79	17.13	18.11	18.1	-0.005565834	NA	Ortholog(s) have role in chromatin silencing at silent mating-type cassette, double-strand break repair via homologous recombination, intra-S DNA damage checkpoint, mitotic sister chromatid cohesion and nucleus localization
orf19.5277	orf19.5277	18.82	17.13	13.28	11.9	-0.236656842	NA	Ortholog(s) have role in nuclear-transcribed mRNA catabolic process, 3'-5' exonucleolytic nonsense-mediated decay, protein ubiquitination and cytoplasm, nucleolus, polysome localization
orf19.5809	orf19.5809	26.59	17.12	24.92	32.14	0.203293739	NA	Putative arylformamidase, enzyme of the NAD biosynthesis pathway; Gcn4p-regulated
orf19.7405	orf19.7405	16.85	17.11	13.28	15.98	-0.107412232	NA	Ortholog of Rad33; involved in nucleotide excision repair in S. cerevisiae; induced by Mnl1 under weak acid stress
orf19.6276	orf19.6276	18.28	17.08	12.69	12.96	-0.194337361	NA	Protein of unknown function; rat catheter biofilm repressed
orf19.872	orf19.872	16.94	17.08	16.6	16.16	-0.026555995	NA	Ortholog of C. dubliniensis CD36 : Cd36_18230, Debaryomyces hansenii CBS767 : DEHA2E20636g, Pichia stipitis Pignal : PICST_66811 and Spathaspora passalidarum NRRL Y-27907 : SPAPADRAFT_60843
orf19.6055	orf19.6055	18.51	17.07	17.94	18.15	-0.005565834	NA	Ortholog of C. dubliniensis CD36 : Cd36_00460, C. parapsilosis CDC317 : CPAR2_800010, Candida tenuis NRRL Y-1498 : CANTEDRAFT_125085 and Debaryomyces hansenii CBS767 : DEHA2D09086g

orf19.4740	orf19.4740	17.36	17.05	14.88	13.66	-0.109505595	NA	Ortholog(s) have aminoacyl-tRNA hydrolase activity, role in mitochondrial translation and mitochondrion localization
orf19.7454	TAF60	23.38	17.05	20.7	18.79	-0.005043749	NA	Putative TFIID and SAGA complex subunit; mutation confers hypersensitivity to amphotericin B
orf19.4294	orf19.4294	21.17	17.04	23.66	22.43	0.136550012	NA	Ortholog(s) have oxidoreductase activity, role in cytochrome c-heme linkage, mitochondrial membrane organization and extrinsic to mitochondrial inner membrane localization
orf19.398	orf19.398	17.85	17.01	27.32	21.93	0.242029497	NA	Ortholog of C. dubliniensis CD36 : Cd36_08040, C. parapsilosis CDC317 : CPAR2_207180, Candida tenuis NRRL Y-1498 : CANTEDRAFT_114140 and Debaryomyces hansenii CBS767 : DEHA2G13992g
orf19.3305	ZCF17	20.26	17	19.54	14.87	-0.045440288	NA	Putative Zn(II)2Cys6 transcription factor
orf19.940	BUD2	19.08	17	20.06	18.33	0.031707189	NA	GTPase activating protein (GAP) for Rsr1; negative regulator of filament branching, acts in hyphal growth guidance; required for wild-type budding pattern; induced in low iron; regulated by tyrosol and cell density
orf19.5555	orf19.5555	17.15	16.98	16.78	16.13	-0.026555995	NA	Ortholog of C. dubliniensis CD36 : Cd36_63300, Candida tropicalis MYA-3404 : CTRG_05769 and Candida albicans WO-1 : CAWG_05077
orf19.695	RGS2	24.04	16.98	16.08	18.22	-0.134424609	NA	Protein of RGS superfamily; mutants are viable; rat catheter and Spider biofilm induced
orf19.1397	orf19.1397	19.39	16.96	17.19	12.62	-0.127567153	NA	Has domain(s) with predicted heme binding activity

orf19.7642	orf19.7642	16.11	16.95	18.06	17.97	0.052448069	NA	Ortholog of S. cerevisiae Vps3; CORVET tethering complex component involved in vacuolar protein sorting; Hap43-repressed gene
orf19.1934	HST3	23.04	16.93	23.74	14.98	-0.025479023	NA	Histone H3K56 deacetylase; reduced copy number increases opaque cell formation; repressed by MMS, hydroxyurea and high-levels of hydrogen peroxide; Hap43p-induced; ectopic expression blocks genotoxin-induced switching; nicotinamide target
orf19.7027	orf19.7027	12.36	16.9	12.66	10.99	-0.118783906	NA	Protein of unknown function; Spider biofilm induced
orf19.5469	orf19.5469	15.42	16.86	25.31	26.91	0.351321248	NA	Protein with a predicted DEAD-like DNA/RNA helicase domain; shows colony morphology-related gene regulation by Ssn6; overlaps orf19.5472; Spider biofilm repressed
orf19.2412	orf19.2412	25.06	16.85	26.81	27.25	0.193061116	NA	Ortholog(s) have role in late endosome to vacuole transport via multivesicular body sorting pathway and Golgi apparatus, Vps55/Vps68 complex, fungal-type vacuole membrane, late endosome localization
orf19.6995	ATO6	13.47	16.83	24.62	19.69	0.272950072	NA	Putative fungal-specific transmembrane protein
orf19.3251	ARC19	19.83	16.83	25	22.37	0.171718354	NA	Putative ARP2/3 complex subunit; Hap43-induced gene; mutation confers hypersensitivity to cytochalasin D; rat catheter biofilm repressed
orf19.1729	orf19.1729	22.44	16.83	19.03	17.53	-0.042378792	NA	Putative transcription factor with zinc finger DNA-binding motif
orf19.4744	orf19.4744	21.01	16.82	20.12	18.02	-0.005661221	NA	Putative phosphatidylinositol 3-phosphate (PI3P) phosphatase; repressed by alpha pheromone in SpiderM medium

orf19.5220	orf19.5220	22.56	16.8	11.91	11.68	-0.332326784	NA	Putative RNA exonuclease; induced in a ssr1 null mutant
orf19.1676	orf19.1676	18.67	16.8	25.56	24.37	0.24053461	NA	Predicted potassium ion transporter; Spider biofilm induced
orf19.6043	orf19.6043	14.14	16.79	24.28	21.2	0.254329121	NA	Ortholog(s) have mitochondrial membrane localization
orf19.856	IFK2	18.87	16.76	15.53	18.38	-0.04379754	NA	Putative thiol-specific monooxygenase; mutant is viable; flow model biofilm induced
orf19.2521	orf19.2521	20.33	16.74	18.71	16.57	-0.02510044	NA	Ortholog of C. dubliniensis CD36 : Cd36_26390, Debaryomyces hansenii CBS767 : DEHA2C05302g, Pichia stipitis Pignal : PICST_68531 and Spathaspora passalidarum NRRL Y-27907 : SPAPADRAFT_59916
orf19.7068	MAC1	21.5	16.73	18.99	14.67	-0.083216344	NA	Copper fist transcription factor; regulator of CTR1 copper transporter; induces CTR1 in low-copper conditions; ectopic expression causes copper-sensitive stimulation of filamentation in S. cerevisiae; Spider biofilm induced
orf19.4795	orf19.4795	20.62	16.73	26.11	22.02	0.169154634	NA	Protein of unknown function; Sef1-, Sfu1-, and Hap43 regulated; Spider biofilm induced
orf19.4194	orf19.4194	15.15	16.69	15.97	11.33	-0.114092844	NA	Putative TFIIH complex subunit; possibly an essential gene, disruptants not obtained by UAU1 method
orf19.5665	orf19.5665	21.86	16.68	22.57	19.31	0.048135986	NA	Ortholog(s) have D-arabinose 1-dehydrogenase [NAD(P)+] activity and role in dehydro-D-arabinono-1,4-lactone biosynthetic process

orf19.2469	RAD10	18.16	16.67	13.12	17.18	-0.104957932	NA	Ortholog of S. cerevisiae Rad10, an endonuclease involved in nucleotide excision repair; mutant is extremely sensitive to UV irradiation; transcript repressed in alkaline conditions
orf19.5742	ALS9	13.13	16.67	12.29	14.02	-0.092731552	NA	ALS family cell-surface glycoprotein; expressed during infection of human epithelial cells; confers laminin adhesion to S. cerevisiae; highly variable; putative GPI-anchor; Hap43-repressed
orf19.4409	orf19.4409	17.82	16.66	16.71	12.88	-0.108540321	NA	Ortholog(s) have nucleoside-triphosphatase activity, nucleoside-triphosphate diphosphatase activity and role in cellular response to phosphate starvation, nucleoside triphosphate metabolic process
orf19.1836	APN2	22.86	16.66	19.37	17.57	-0.060306661	NA	Putative class II abasic (AP) endonuclease; flucytosine induced
orf19.794	SSN3	17.16	16.64	13.87	10.83	-0.1984559	NA	Putative cyclin-dependent protein kinase; mutants are sensitive to growth on H2O2 medium
orf19.6131	KSR1	15.81	16.61	24.86	18.39	0.177676431	NA	3-ketosphinganine reductase, catalyzes the second step in phytosphingosine synthesis
orf19.5865	orf19.5865	20.79	16.6	16.56	15.07	-0.122840469	NA	Ortholog(s) have RNA-dependent ATPase activity, role in generation of catalytic spliceosome for first transesterification step and U2-type catalytic step 1 spliceosome localization
orf19.805	orf19.805	23.67	16.58	22.06	19.4	-0.005084272	NA	Putative protein of unknown function; possibly mitochondrial; Hap43-repressed; Spider biofilm repressed
orf19.2187	ALG7	22.33	16.56	23.9	18.51	0.065324185	NA	Protein involved in cell wall mannan biosynthesis; mutation confers hypersensitivity to tunicamycin; transcript elevated in chk1, nik1, and sln1 homozygous null mutants; possibly essential gene, disruptants not obtained by UAU1 method

orf19.5533	orf19.5533	19.69	16.55	22.52	19.15	0.085063178	NA	Ortholog(s) have guanyl-nucleotide exchange factor activity, ubiquitin binding activity
orf19.7648	orf19.7648	15.25	16.55	21.19	17.41	0.108560131	NA	Has domain(s) with predicted antiporter activity, drug transmembrane transporter activity, role in drug transmembrane transport and membrane localization
orf19.1332	SNG4	18.67	16.54	16.5	12.37	-0.148790746	NA	Putative membrane transporter; Hap43-induced; inducd by Mnl1p under weak acid stress; shows Mob2-dependent hyphal regulation
orf19.1310	orf19.1310	17.77	16.51	12.84	13.51	-0.171458431	NA	Ortholog(s) have role in nucleotide-excision repair and nucleus localization
orf19.1860.1	orf19.1860.1	16.12	16.51	24.71	22.57	0.265958923	NA	Ortholog(s) have extracellular region localization
orf19.4629	orf19.4629	19.18	16.51	13.37	10.45	-0.283628058	NA	Ortholog(s) have ubiquitin-ubiquitin ligase activity, role in mitochondrion inheritance, protein monoubiquitination, protein polyubiquitination and plasma membrane, ubiquitin ligase complex localization
orf19.7032	orf19.7032	19.37	16.49	16.57	18.39	-0.004685555	NA	Ortholog of C. dubliniensis CD36 : Cd36_70790, C. parapsilosis CDC317 : CPAR2_301530, Candida tenuis NRRL Y-1498 : CANTEDRAFT_94947 and Debaryomyces hansenii CBS767 : DEHA2A05258g
orf19.5977	CEM1	22.27	16.48	19.96	21.49	0.050993931	NA	Protein similar to S. cerevisiae Cem1p, an acyl carrier protein involved in fatty acid biosynthesis; likely to be essential for growth, based on an insertional mutagenesis strategy
orf19.4911	orf19.4911	21.77	16.47	22.1	19.75	0.067799777	NA	BED zinc finger protein; predicted DNA binding protein; Spider biofilm repressed

orf19.2751	orf19.2751	20.92	16.47	17.27	19.42	-0.02272262	NA	Predicted membrane transporter; member of the monocarboxylate porter (MCP) family, major facilitator superfamily; Hap43-induced gene; alkaline upregulated by Rim101; possibly an essential gene, disruptants not obtained by UAU1 method
orf19.3545	orf19.3545	18.91	16.46	19.57	17.66	0.05156339	NA	Ortholog(s) have protein serine/threonine kinase activity, role in meiotic anaphase II, protein phosphorylation, septation initiation signaling cascade and cellular bud neck, cytoplasm, new mitotic spindle pole body, nucleus localization
orf19.4428	orf19.4428	20.85	16.44	19.24	17.68	-0.004805075	NA	Ortholog of C. dubliniensis CD36 : Cd36_06910, C. parapsilosis CDC317 : CPAR2_208560, Candida tenuis NRRL Y-1498 : CANTEDRAFT_95854 and Debaryomyces hansenii CBS767 : DEHA2F25982g
orf19.6437	CDC23	17.3	16.44	23.82	21.05	0.21589818	NA	Similar to anaphase-promoting complex component; possibly transcriptionally regulated by Tac1p
orf19.7402	DOT1	18.06	16.42	18.74	15.83	0.01315798	NA	Putative modulator of white-opaque switching
orf19.6566	orf19.6566	19.64	16.41	19.17	14.12	-0.065301136	NA	Ortholog of C. dubliniensis CD36 : Cd36_71420, C. parapsilosis CDC317 : CPAR2_300980, Candida tenuis NRRL Y-1498 : CANTEDRAFT_94952 and Debaryomyces hansenii CBS767 : DEHA2A05346g
orf19.1181	orf19.1181	12.6	16.4	14.29	12.22	-0.073212068	NA	Has domain(s) with predicted catalytic activity and membrane localization
orf19.6170	orf19.6170	18.68	16.39	30.22	22.76	0.305355495	NA	Ortholog(s) have microtubule binding, protein heterodimerization activity, protein homodimerization activity
orf19.144	SNU114	22.49	16.36	17.81	14.22	-0.122106005	NA	Protein similar to S. cerevisiae Snu114p, which is an RNA helicase involved in pre-mRNA splicing; likely to be essential for growth, based on an insertional mutagenesis strategy

orf19.2711.1	MED20	13.14	16.35	18.99	13.36	0.052524322	NA	Subunit of the RNA polymerase II mediator complex; involved in control of filamentous growth and Spider biofilm formation; rat catheter biofilm repressed
orf19.931	orf19.931	22.98	16.32	26.9	21.76	0.166664506	NA	Ortholog of Candida albicans WO-1 : CAWG_04452
orf19.5155	CHS6	19.23	16.32	20.95	19.98	0.107043592	NA	Protein with tetratricopeptide repeats (TPRs); ortholog of S. cerevisiae Chs6, which has role in localizing chitin synthase; Cyr1-regulated in hyphae; possibly essential, disruptants not obtained by UAU1 method; flow model biofilm repressed
orf19.5487	CDC46	21.47	16.31	37.17	31.59	0.499116543	0.541043	Putative hexameric MCM complex subunit; predicted role in control of cell division; periodic mRNA expression. peak at cell-cycle M/G1 phase; regulated by tyrosol, cell density, Plc1; repressed by alpha pheromone in SpiderM medium
orf19.3802	PMT6	18.9	16.31	28.27	23.91	0.291528509	NA	Protein mannosyltransferase; required for virulence in mice, adhesion to endothelium; role in hyphal growth signaling, hygromycin B sensitivity; no major role in cellular PMT activity; Hap4-repressed; Bcr1-repressed in RPMI a/a biofilms
orf19.547	orf19.547	22.83	16.28	25.11	18.57	0.082676258	NA	Ortholog(s) have 5'-flap endonuclease activity, single-stranded DNA 5'-3' exodeoxyribonuclease activity
orf19.3765	RAX2	15.08	16.26	19.85	20.02	0.168454211	NA	Plasma membrane protein involved in establishment of bud sites and linear direction of hyphal growth; filament induced, repressed by Rim101, Tup1; Hap43-induced; Spider biofilm induced
orf19.2757	orf19.2757	13.78	16.23	12.8	15.06	-0.048406625	NA	Has domain(s) with predicted role in cell redox homeostasis
orf19.524	orf19.524	17.89	16.22	17.84	15.62	-0.006136946	NA	Ortholog(s) have role in mitotic sister chromatid segregation and cytosol, nucleus localization

orf19.3234	OYE22	21.63	16.22	12.13	13.86	-0.247546022	NA	Putative NADPH dehydrogenase; rat catheter biofilm induced
orf19.4163	orf19.4163	18.62	16.22	22.9	16.63	0.086716212	NA	Ortholog(s) have role in protein targeting to vacuole, receptor-mediated endocytosis, response to pheromone and actin cortical patch, cytosol, endosome, plasma membrane, ribosome localization
orf19.3871	DAD3	14.32	16.22	21.89	21.4	0.242284873	NA	Subunit of the Dam1 (DASH) complex, which acts in chromosome segregation by coupling kinetochores to spindle microtubules
orf19.5050	MTO1	18.87	16.21	17.05	15.72	-0.045056187	NA	Putative mitochondrial protein; rat catheter biofilm induced
orf19.2996	orf19.2996	17.89	16.19	20.17	17.31	0.051682319	NA	Ortholog(s) have cytoplasm localization
orf19.1534	orf19.1534	16.82	16.19	13.34	16.98	-0.065360933	NA	Ortholog of S. cerevisiae Zrt3, vacuolar membrane zinc transporter; predicted Kex2 substrate; induced in oralpharyngeal candidasis; flow model biofilm induced; Spider biofilm induced
orf19.679	FGR39	16.18	16.18	17.51	15.45	0.013047287	NA	Protein lacking an ortholog in S. cerevisiae; transposon mutation affects filamentous growth
orf19.6845	orf19.6845	23.57	16.18	16.53	13.42	-0.198666627	NA	Putative transcription factor with bZIP DNA-binding motif; rat catheter biofilm induced
orf19.7337	orf19.7337	16.18	16.17	20.93	14.3	0.050749545	NA	Protein with a nischarin related domain and leucine rich repeats; Spider biofilm induced

orf19.5404.1	FLO9	14.98	16.16	16.25	16.36	0.014162244	NA	Putative adhesin-like cell wall mannoprotein; repressed during the mating process; mutation confers hypersensitivity to toxic ergosterol analog; decreased transcription is observed upon fluphenazine treatment
orf19.248	APL5	18.77	16.15	18.11	18.1	0.014317625	NA	Ortholog of S. cerevisiae and S. pombe Apl5; subunit of the AP-3 adaptor complex involved in Golgi-to-vacuole transport; phosphorylated protein; mutant is viable;
orf19.6481	YPS7	17.04	16.15	16.99	14.45	-0.047644323	NA	Putative aspartic-type endopeptidase with limited ability to degrade alpha pheromone; mutants show increased sensitivity to alpha pheromone
orf19.2196	orf19.2196	18.06	16.14	17.46	13.09	-0.08836961	NA	Ortholog(s) have ubiquitin binding activity and cytoplasm localization
orf19.4919	orf19.4919	13.69	16.11	15.79	20.47	0.114619815	NA	Has domain(s) with predicted DNA binding, nucleic acid binding activity
orf19.1955	orf19.1955	18.74	16.1	15.77	11.38	-0.172025946	NA	Ortholog(s) have palmitoyltransferase activity, role in protein palmitoylation, protein targeting to membrane and endoplasmic reticulum palmitoyltransferase complex, extrinsic to endoplasmic reticulum membrane localization
orf19.5177	orf19.5177	19.18	16.1	13.59	11.02	-0.216870613	NA	Ortholog of Candida albicans WO-1 : CAWG_05627
orf19.6013	orf19.6013	16.34	16.09	19.06	16.54	0.072508883	NA	Ortholog(s) have cytosol, nucleolus localization
orf19.7372	MRR1	19.94	16.07	17.63	13.33	-0.105883651	NA	Putative Zn(II)2Cys6 transcription factor; regulator of MDR1 transcription; gain-of-function mutations cause upregulation of MDR1 (a plasma membrane multidrug efflux pump) and multidrug resistance; Hap43-induced

orf19.5275	orf19.5275	11.78	16.06	21.06	18.31	0.210154508	NA	Ortholog(s) have ATPase activator activity, role in late endosome to vacuole transport via multivesicular body sorting pathway, positive regulation of protein oligomerization and endosome membrane, multivesicular body localization
orf19.508	QDR1	6.69	16.06	5.83	12.13	-0.121739994	NA	Putative antibiotic resistance transporter; regulated by white-opaque switch, Nrg1, Tup1; Hap43, caspofungin repressed; repressed during chlamydospore formation; flow model biofilm induced; Spider biofilm repressed
orf19.3848	orf19.3848	22.56	16.05	21.93	23.07	0.102386604	NA	Ortholog of C. dubliniensis CD36 : Cd36_31420, C. parapsilosis CDC317 : CPAR2_205100, Debaryomyces hansenii CBS767 : DEHA2G10648g and Pichia stipitis Pignal : PICST_32997
orf19.4628	orf19.4628	14.93	16.05	16.51	13.02	-0.028744204	NA	Putative cleavage and polyadenylation factor; heterozygous null mutant exhibits hypersensitivity to parnafungin and cordycepin in the C. albicans fitness test; possibly an essential gene, disruptants not obtained by UAU1 method
orf19.2472	orf19.2472	24.63	16.04	22.08	16	-0.059535014	NA	Ortholog of S. pombe replication termination factor Rtf2; Spider biofilm induced
orf19.4765	PGA6	18.36	16.02	21.21	18.01	0.089181987	NA	GPI-anchored cell wall adhesin-like protein; induced by high iron; upregulated upon Als2 depletion; mRNA binds She3 and is localized to hyphal tips; Spider biofilm repressed
orf19.4403	VPS11	16.97	16.02	19.92	15.25	0.03193348	NA	Protein involved in protein trafficking; putative role in vesicle-target membrane fusion; mutant lacks vacuole; role in hyphal growth, possibly via vacuole expansion into hypha; role in killing of and survival within macrophage
orf19.7406	orf19.7406	20.2	15.98	18.36	17.81	-0.004748847	NA	Ortholog of C. dubliniensis CD36 : Cd36_86220, C. parapsilosis CDC317 : CPAR2_404490, Candida tenuis NRRL Y-1498 : CANTEDRAFT_94473 and Debaryomyces hansenii CBS767 : DEHA2D01804g
orf19.4354	MCM2	17.94	15.96	30.49	24.04	0.34142693	NA	Phosphorylated protein of unknown function; transcription is periodic with a peak at M/G1 phase of the cell cycle

orf19.4148	orf19.4148	16.98	15.95	23.61	18.73	0.180018749	NA	Ortholog of C. dubliniensis CD36 : Cd36_50820, C. parapsilosis CDC317 : CPAR2_501670, Candida tenuis NRRL Y-1498 : CANTEDRAFT_115294 and Debaryomyces hansenii CBS767 : DEHA2E13464g
orf19.3701	orf19.3701	17.01	15.94	13.78	11.18	-0.177974635	NA	Protein of unknown function; mutants are viable; ortholog of S. cervisiae Art10, a protein of unknown function
orf19.121	ARC18	17.01	15.94	14.14	12.01	-0.155132774	NA	Putative ARP2/3 complex subunit; mutation confers hypersensitivity to cytochalasin D
orf19.2986	orf19.2986	18.07	15.94	20.15	14.84	0.01237047	NA	Ortholog of C. dubliniensis CD36 : Cd36_02750, C. parapsilosis CDC317 : CPAR2_106015, Candida tenuis NRRL Y-1498 : CANTEDRAFT_107500 and Debaryomyces hansenii CBS767 : DEHA2D08250g
orf19.2896	SOU1	23.34	15.93	17.91	11.41	-0.20160227	NA	Enzyme involved in utilization of L-sorbose; has sorbitol dehydrogenase, fructose reductase, and sorbose reductase activities; NAD-binding site motif; transcriptional regulation affected by chromosome 5 copy number; Hap43p-induced gene
orf19.666	orf19.666	15.34	15.93	15.55	16.56	0.034758377	NA	Essential protein required for the DNA integrity checkpoint pathway; Spider biofilm induced
orf19.3469	orf19.3469	17.2	15.93	47.53	44.83	0.874782764	0.042877	S. cerevisiae ortholog Stb1 has a role in regulation of MBF-specific transcription at Start; induced in a cyr1 null mutant; Spider biofilm induced
orf19.2629	USO1	14.3	15.92	17.41	17.6	0.094703205	NA	Ortholog(s) have role in ER to Golgi vesicle-mediated transport, Golgi vesicle docking, SNARE complex assembly and ER to Golgi transport vesicle membrane, Golgi membrane localization
orf19.1887	orf19.1887	18.42	15.91	19.13	14.67	-0.006879764	NA	Ortholog(s) have sterol esterase activity, role in sterol metabolic process and integral to membrane, lipid particle localization

orf19.6689	ARG4	13.13	15.91	15.61	19.96	0.135035493	NA	Argininosuccinate lyase, catalyzes the final step in the arginine biosynthesis pathway; alkaline downregulated; flow model biofilm induced; Spider biofilm induced
orf19.458.1	orf19.458.1	15.15	15.9	21.15	15.86	0.110317434	NA	Ortholog of C. parapsilosis CDC317 : CPAR2_205150, Debaryomyces hansenii CBS767 : DEHA2G10538g, Pichia stipitis Pignal : psti_CGOB_00130 and Candida guilliermondii ATCC 6260 : PGUG_02975
orf19.1369	orf19.1369	15.62	15.9	17	14.28	-0.027741785	NA	Protein with predicted peptidase domains; Hap43-repressed gene
orf19.4742	orf19.4742	18.02	15.9	11.98	11.8	-0.21980357	NA	Putative SH3-domain-containing protein
orf19.2062	SOD4	13.71	15.9	18.87	25.15	0.259810383	NA	Cu and Zn-containing superoxide dismutase; role in response to host innate immune ROS; regulated on white-opaque switch; ciclopirox olamine induced; caspofungin repressed; SOD1,4,5,6 gene family; yeast-associated; Spider biofilm induced
orf19.4131	orf19.4131	17.9	15.89	21.69	16.35	0.069149764	NA	Ortholog(s) have role in protein folding in endoplasmic reticulum and ER membrane protein complex localization
orf19.4264	orf19.4264	16.93	15.88	25.63	26.04	0.332627717	NA	Protein of unknown function; induced during chlamydospore formation in both C. albicans and C. dubliniensis; flow model biofilm induced
orf19.7091	orf19.7091	19.97	15.88	13.36	13.35	-0.212243333	NA	Protein of unknown function; induced by nitric oxide; Spider biofilm repressed
orf19.4805	orf19.4805	16.44	15.87	15.69	15.54	-0.006038487	NA	Putative membrane protein; induced by alpha pheromone in SpiderM medium; Hap4-induced gene; Spider biofilm induced

orf19.2771	BEM3	16.46	15.87	16.79	14.97	-0.006814623	NA	Putative GTPase-activating protein (GAP) for Rho-type GTPase Cdc42p; involved in cell signaling pathways that control cell polarity; similar to S. cerevisiae Bem3p
orf19.5880	orf19.5880	21.37	15.85	21.72	17.96	0.049735244	NA	Putative voltage-gated chloride channel; predicted role in copper ion and iron ion homeostasis; flow model biofilm induced
orf19.122	CDC20	14.4	15.85	17.56	15.22	0.053612013	NA	Activator of anaphase-promoting complex/cyclosome; induced under Cdc5 depletion; member of conserved Mcm1 regulon; mRNA expression peaks at cell-cycle G2/M phase; mRNA binds She3 and is localized to buds of yeast cells and to hyphal tips
orf19.1163	orf19.1163	23.76	15.83	22.49	14.42	-0.079834663	NA	Has domain(s) with predicted GTP binding activity
orf19.3673	orf19.3673	16.98	15.82	15.78	18.85	0.034934812	NA	Ortholog(s) have Rab guanyl-nucleotide exchange factor activity, role in ER to Golgi vesicle-mediated transport, chromosome organization and TRAPP complex, cytosol, nucleus localization
orf19.3178	orf19.3178	24.14	15.81	18.3	14.2	-0.157617361	NA	Ortholog(s) have RNA binding activity, role in mRNA splicing, via spliceosome and U2-type prespliceosome localization
orf19.1509	ROD1	17.08	15.81	11.8	10.53	-0.224085421	NA	Protein similar to S. cerevisiae Rod1;a membrane protein with a role in drug tolerance; repressed by Rgt1; mutant is viable
orf19.4494	KTR2	20.98	15.79	15.37	15.49	-0.143913528	NA	Mannosyltransferase; transcription elevated in chk1, nik1, and sln1 homozygous null mutants; fungal-specific (no human or murine homolog); possibly an essential gene, disruptants not obtained by UAU1 method
orf19.5523	orf19.5523	18.6	15.78	13.49	18.54	-0.061756181	NA	Ortholog of C. dubliniensis CD36 : Cd36_62780, C. parapsilosis CDC317 : CPAR2_601690, Candida tenuis NRRL Y-1498 : CANTEDRAFT_113271 and Debaryomyces hansenii CBS767 : DEHA2A06644g

orf19.5971	orf19.5971	14.67	15.77	15.23	12.92	-0.070711526	NA	Ortholog of S. cerevisiae : MTC6, C. glabrata CBS138 : CAGL0I01892g, C. dubliniensis CD36 : Cd36_84990, C. parapsilosis CDC317 : CPAR2_405620 and Candida tenuis NRRL Y-1498 : CANTEDRAFT_99433
orf19.1221	ALG2	18.85	15.77	19.22	17.68	0.033072883	NA	Putative mannosyltransferase involved in cell wall mannan biosynthesis; transcription is elevated in chk1, nik1, and sln1 homozygous null mutants
orf19.5558	RBF1	17.61	15.75	20.34	17.58	0.070962885	NA	Transcription factor; glutamine-rich activation domain; binds RPG-box DNA sequences; predominantly nuclear; mutation causes accelerated induction of filamentous growth; antigenic during human oral infection; Sko1p-repressed
orf19.7022	orf19.7022	16.11	15.74	23.19	23	0.254054503	NA	Ortholog of C. dubliniensis CD36 : Cd36_70850, Candida tenuis NRRL Y-1498 : cten_CGOB_00183, Debaryomyces hansenii CBS767 : DEHA2F22462g and Pichia stipitis Pignal : PICST_60416
orf19.6952	orf19.6952	16.12	15.74	14.24	10.74	-0.15798416	NA	Ortholog(s) have DNA binding, chromatin binding, histone deacetylase activity
orf19.4145	ZCF20	19.86	15.74	18.06	12.09	-0.12666994	NA	Zn(II)2Cys6 transcription factor orthologous to S. cerevisiae Hap1; regulated by Sef1, Sfu1; Hap43-induced; Spider biofilm induced
orf19.7564	DPB2	14.61	15.73	12.23	12.57	-0.136079888	NA	Probable subunit of DNA polymerase II (DNA polymerase epsilon), similar to S. cerevisiae Dpb2p; essential for viability; rat catheter biofilm induced
orf19.422	SPT20	16.38	15.72	18.29	13.14	-0.028431969	NA	Putative transcription factor; downregulated upon adherence to polystyrene; flucytosine repressed
orf19.2728	orf19.2728	15.31	15.72	17.88	14.84	0.033211101	NA	Ortholog(s) have DNA clamp loader activity, role in DNA damage checkpoint, nucleotide-excision repair, reciprocal meiotic recombination and Rad17 RFC-like complex, nucleus localization

orf19.6641	orf19.6641	17.03	15.7	28.44	23.23	0.314548241	NA	Ortholog of Candida albicans WO-1 : CAWG_01892
orf19.540	orf19.540	16.69	15.69	18.43	15.84	0.013496431	NA	Has domain(s) with predicted palmitoyl-(protein) hydrolase activity and role in cellular protein modification process
orf19.2788	orf19.2788	11.31	15.65	12.98	14.68	0.014616292	NA	Has domain(s) with predicted RNA binding, pseudouridine synthase activity and role in pseudouridine synthesis
orf19.5097	CAT8	18.91	15.65	22.38	21.68	0.160393351	NA	Zn(II)2Cys6 transcription factor; similar to S. cerevisiae Cat8 but mutant phenotype suggests different target genes; mutant displays increased filamentous/invasive growth; flucytosine repressed; rat catheter biofilm induced
orf19.5673	OPT7	16.63	15.64	15.03	10.59	-0.155453398	NA	Putative oligopeptide transporter; possibly transports GSH or related compounds; Hog1-induced; expression of OPT6, -7, or -8 does not suppress defect of mutant lacking OPT1-3; Hap43-repressed; F-12/CO2 early biofilm induced
orf19.1670	BRO1	18.36	15.63	18.38	15.85	-0.006136946	NA	Class E vacuolar protein sorting factor; role in transport from multivesicular body to vacuole; not involved in Rim101 pathway; macrophage and pseudohyphal-repressed; flow model biofilm induced
orf19.2455	orf19.2455	18.76	15.63	21.26	15.97	0.031498608	NA	Ortholog(s) have gamma-glutamyltransferase activity, omega peptidase activity, role in glutathione catabolic process and cytoplasm, nucleus localization
orf19.2382	orf19.2382	16.38	15.62	17.47	17.9	0.054185748	NA	Protein similar to isoleucyl-tRNA synthetase; isoleucyl-tRNA synthetase is the target of drugs including the cyclic beta-amino acid icofungipen/PLD-118/BAY-10-8888 and mupirocin
orf19.5342	orf19.5342	18.81	15.61	27.2	23.24	0.259463266	NA	Ortholog(s) have cytosol, extracellular region, fungal-type vacuole, nucleus localization

orf19.1029	RPP1	24.32	15.6	18.19	15.76	-0.11730109	NA	Putative ortholog of S. cerevisiae Rpp1; subunit of both RNase MRP and nuclear RNase P; rat catheter and Spider biofilm induced
orf19.7101	orf19.7101	16.61	15.59	17.73	15.78	0.013496431	NA	Ortholog(s) have telomeric DNA binding activity, role in protein localization to chromosome, telomere maintenance via telomerase and ASTRA complex, nuclear telomere cap complex localization
orf19.5454	DAL1	16.76	15.59	17.13	14.08	-0.047644323	NA	Putative allantoinase; transcript regulated by Nrg1 and Mig1; macrophage/pseudohyphal-repressed
orf19.4440	SEC34	16.69	15.58	24.82	22.25	0.249904794	NA	Ortholog(s) have role in CVT pathway, ER to Golgi vesicle-mediated transport, macroautophagy, peroxisome degradation, retrograde transport, vesicle recycling within Golgi and Golgi transport complex, cell division site localization
orf19.5103	orf19.5103	16.72	15.57	14.02	11.93	-0.155132774	NA	Protein with a predicted phosphoglycerate mutase family domain; Hap43-repressed; clade-associated gene expression; induced by hypoxia
orf19.335	orf19.335	14.73	15.56	11.15	8.13	-0.28195502	NA	Sef1p-, Sfu1p-, and Hap43p-regulated gene
orf19.5410	orf19.5410	16.59	15.56	16.54	9.41	-0.154714354	NA	Ortholog(s) have microtubule plus-end binding activity and role in conidium formation, nuclear migration along microtubule, regulation of microtubule motor activity, sexual sporulation resulting in formation of a cellular spore
orf19.7206	OCA6	14.91	15.54	16.14	14.87	-0.00677119	NA	Putative protein phosphatase of the PTP family (tyrosine-specific), similar to S. cerevisiae Oca6p; mutant shows virulence defect
orf19.2184	orf19.2184	21.78	15.52	28.18	22.14	0.200867277	NA	Putative fumarylacetoacetate hydrolase; clade-associated gene expression; rat catheter and Spider biofilm repressed

orf19.6850	orf19.6850	17.13	15.52	16.55	13.98	-0.047644323	NA	Putative transcription factor with C3HC4 zinc finger DNA-binding motif; mutants are viable
orf19.4191.1	orf19.4191.1	16.27	15.51	16.16	13.33	-0.069777537	NA	Ortholog(s) have ubiquitin-ubiquitin ligase activity, role in ER-associated ubiquitin-dependent protein catabolic process, protein polyubiquitination and cytosol, nuclear envelope, proteasome complex localization
orf19.1303	orf19.1303	17.41	15.5	14.2	13.88	-0.089333144	NA	Ortholog(s) have translation release factor activity, role in mitochondrial translational termination and mitochondrion localization
orf19.2866	orf19.2866	24.89	15.49	51.67	35.9	0.66101324	0.229994	Has domain(s) with predicted DNA binding activity
orf19.3531	orf19.3531	18	15.49	19.77	17.26	0.072082533	NA	Ortholog(s) have role in regulation of DNA-dependent DNA replication initiation and DNA replication preinitiation complex, endoplasmic reticulum, nuclear envelope, spindle pole body localization
orf19.5817	orf19.5817	21.21	15.48	29.88	26.18	0.333645839	NA	Ortholog(s) have Rab guanyl-nucleotide exchange factor activity, role in ER to Golgi vesicle-mediated transport, intra-Golgi vesicle-mediated transport and TRAPP complex, cis-Golgi network membrane, cytosol, nucleus localization
orf19.231	APL2	17.08	15.48	21.86	21.3	0.202672099	NA	Phosphorylated protein of unknown function; mutation confers hypersensitivity to toxic ergosterol analog
orf19.3163	orf19.3163	18.54	15.48	16.59	13.91	-0.066282249	NA	Ortholog of C. dubliniensis CD36 : Cd36_51910, C. parapsilosis CDC317 : CPAR2_101650, Candida tenuis NRRL Y-1498 : CANTEDRAFT_94797 and Debaryomyces hansenii CBS767 : DEHA2D06578g
orf19.894	orf19.894	18.49	15.48	18.71	19.84	0.110987731	NA	Ortholog(s) have guanyl-nucleotide exchange factor activity, role in retrograde transport, endosome to Golgi and Golgi membrane, Ric1p-Rgp1p complex, guanyl-nucleotide exchange factor complex localization

orf19.5397	orf19.5397	13.38	15.47	15.18	10.98	-0.052033642	NA	Putative conserved oligomeric Golgi complex subunit; decreased transcription is observed upon fluphenazine treatment
orf19.5910	orf19.5910	15.15	15.47	14.87	12.76	-0.04937342	NA	Putative histone acetyltransferase complex subunit; induced upon low-level peroxide stress; Spider biofilm induced
orf19.6908	orf19.6908	13.74	15.47	16.01	11.53	-0.029257671	NA	Dihydrofolate synthetase involved in folic acid biosynthesis
orf19.6758	orf19.6758	18.09	15.47	28.26	34.55	0.496584627	NA	Predcted glucose 1-dehydrogenase (NADP+); rat catheter biofilm repressed
orf19.3793	orf19.3793	15.47	15.46	13.2	11.29	-0.139096637	NA	Protein of unknown function; mRNA binds She3; regulated by Nrg1; upregulated in a cyr1 or ras1 mutant
orf19.1135	CAS1	16.67	15.45	20.17	18.95	0.1304198	NA	Putative transcription factor with Ku70/Ku80 beta-barrel DNA-binding motif; involved in telomerase regulation and telomere protection; mutation causes marginal increase in caspofungin sensitivity
orf19.3796	DCR1	18.69	15.45	17.23	14.5	-0.045433431	NA	Putative Dicer RNAse involved in RNA interference, similar to S. cerevisiae Rnt1p but orthologous to S. castellii Dcr1p, which is not conserved in S. cerevisiae
orf19.2733	orf19.2733	13.89	15.44	17.2	12.65	0.01332018	NA	Putative subunit of phosphatidylinositol 3-kinase complexes I and II; transcription is activated in the presence of elevated CO2
orf19.2194	APM4	20.6	15.44	20.8	15	-0.006090805	NA	Cargo-binding subunit of the clathrin associated protein complex (AP-2), involved in endocytosis

orf19.5124	RBR3	13.25	15.43	14.92	11.96	-0.029571531	NA	Cell wall adhesin-like protein; repressed by Rim101; possibly an essential gene, disruptants not obtained by UAU1 method
orf19.3769	orf19.3769	18.74	15.41	26.55	19.57	0.228027437	NA	Ortholog(s) have zinc ion transmembrane transporter activity, role in zinc ion transport and endoplasmic reticulum localization
orf19.7419	HNT2	21.87	15.41	22.53	22.52	0.15640635	NA	Putative dinucleoside triphosphate hydrolase; induced upon low-level peroxide stress
orf19.5713	YMX6	19.34	15.37	10.68	8.8	-0.315826477	NA	Putative NADH dehydrogenase; macrophage-downregulated gene; induced by nitric oxide; rat catheter biofilm induced
orf19.7473	orf19.7473	17.3	15.35	14.02	12.96	-0.111653601	NA	Ortholog(s) have role in endocytosis and actin cortical patch, cellular bud neck, cytoplasm localization
orf19.4365	orf19.4365	15.75	15.33	14.14	11.41	-0.136913021	NA	Has domain(s) with predicted RNA methyltransferase activity and role in RNA processing
orf19.5873	POL1	15.66	15.32	16.9	13.33	-0.027908525	NA	Putative DNA directed DNA polymerase alpha; RNA abundance regulated by cell cycle, tyrosol and cell density; rat catheter biofilm induced
orf19.4471	orf19.4471	18.29	15.32	17.05	16.62	0.0150772	NA	Ortholog(s) have role in protein targeting to vacuole, re-entry into mitotic cell cycle after pheromone arrest and endoplasmic reticulum membrane localization
orf19.2975	YPT7	21.66	15.31	24.44	21.37	0.138458167	NA	Protein of YPT/RAB family; induced during the mating process

orf19.3273	orf19.3273	17.22	15.3	19.72	15.47	0.052609857	NA	Ortholog(s) have actin monomer binding, protein kinase inhibitor activity, ribosome binding activity, role in negative regulation of protein phosphorylation and cytoplasm, nucleus, polysome, ribosome localization
orf19.4612	orf19.4612	19.5	15.28	6.57	6.83	-0.476785026	NA	Protein with a dienelactone hydrolase domain; Hap43-repressed gene
orf19.2476	orf19.2476	15.72	15.28	20.99	18.02	0.149899394	NA	Ortholog(s) have histone demethylase activity (H3-trimethyl-K4 specific) activity
orf19.4913	orf19.4913	19.07	15.27	19.42	19.46	0.072362197	NA	Ortholog(s) have ubiquitin binding activity, role in cellular protein localization, positive regulation of deubiquitinase activity and cell cortex of cell tip, cell division site, cytosol localization
orf19.4895	orf19.4895	21.97	15.26	24.45	20.29	0.120902223	NA	Ortholog of C. dubliniensis CD36 : Cd36_09650, C. parapsilosis CDC317 : CPAR2_805060, Candida tenuis NRRL Y-1498 : CANTEDRAFT_95780 and Debaryomyces hansenii CBS767 : DEHA2B12320g
orf19.3916	orf19.3916	19.14	15.26	20.03	13.93	-0.006769832	NA	Ortholog of C. dubliniensis CD36 : Cd36_53980, C. parapsilosis CDC317 : CPAR2_100980, Candida tenuis NRRL Y-1498 : CANTEDRAFT_116966 and Debaryomyces hansenii CBS767 : DEHA2G19822g
orf19.1043	orf19.1043	18.76	15.25	20.76	24.25	0.198077204	NA	Ortholog(s) have lysophosphatidic acid acyltransferase activity and role in cellular response to cadmium ion, cellular triglyceride homeostasis, detoxification of cadmium ion, lipid particle organization, protein targeting to vacuole
orf19.2411	SYN8	20.68	15.2	19.67	19.43	0.052020847	NA	Putative endosomal SNARE; role in protein transport; rat catheter and Spider biofilm induced
orf19.7280	orf19.7280	18.94	15.14	0.63	1.26	-0.898320575	NA	Ortholog of Candida albicans WO-1 : CAWG_02163

orf19.2670	orf19.2670	16.23	15.14	17.7	23.52	0.205786107	NA	Ortholog(s) have 3-hydroxyacyl-[acyl-carrier-protein] dehydratase activity, role in fatty acid biosynthetic process and mitochondrion localization
orf19.3730	orf19.3730	17.11	15.14	19.01	12.18	-0.028204071	NA	Protein similar to S. cerevisiae Kre27p, which has a role in resistance to killer toxin; predicted Kex2p substrate
orf19.6530	orf19.6530	19.1	15.14	14.67	13.13	-0.129468708	NA	Similar to bacterial DnaJ; transcript upregulated in low iron; flow model biofilm induced; Spider biofilm induced
orf19.714	orf19.714	16.9	15.14	15.69	15.68	-0.005186987	NA	Ortholog(s) have GTPase activity and role in intracellular protein transport, retrograde transport, endosome to Golgi
orf19.2630	RAD59	22.28	15.14	24.29	11.25	-0.045088223	NA	Protein involved in homologous recombination and DNA breaks repair; similar to S. cerevisiae Rad59p; N-terminal similarity with Rad52p
orf19.6124	ACE2	14.44	15.14	17.85	14.24	0.054651555	NA	Transcription factor; similar to S. cerevisiae Ace2 and Swi5; regulates morphogenesis, cell separation, adherence, virulence in a mice; mutant is hyperfilamentous; rat catheter and Spider biofilm induced
orf19.1459	PPE1	17.19	15.1	20.09	14.71	0.052609857	NA	Protein similar to S. cerevisiae Ppe1p; shows genetic interaction with some genes involved in diploid filamentous growth and haploid invasive growth in S. cerevisiae
orf19.3151	orf19.3151	29.26	15.1	24.53	23.55	0.079700917	NA	Has domain(s) with predicted catalytic activity, coenzyme binding activity and role in cellular metabolic process
orf19.7549	PMT5	16.3	15.1	16.17	13.71	-0.027181442	NA	Protein mannosyltransferase (PMT), expressed at extremely low levels; not required for wild-type hyphal growth, drug resistance, or virulence in mouse systemic infection; one of five PMT family members

orf19.5238	orf19.5238	16.73	15.09	18.42	17.29	0.054327935	NA	Ortholog(s) have cytosol, nucleus localization
orf19.4905	orf19.4905	12.34	15.08	17.08	15.28	0.097823065	NA	Putative MFS transporter; Hap43p-induced gene; also regulated by regulated by Sef1p and Sfu1p; repressed in a ssr1 null mutant
orf19.5517	orf19.5517	18.79	15.06	22.45	19.07	0.126285551	NA	Similar to alcohol dehydrogenases; induced by benomyl treatment, nitric oxide; induced in core stress response; oxidative stress-induced via Cap1; Spider biofilm repressed
orf19.4376	orf19.4376	7.77	15.02	9.04	8.78	-0.128622733	NA	Protein of unknown function; Spider biofilm induced
orf19.5239	orf19.5239	20.95	15.01	20.37	19.17	0.052020847	NA	Predicted alanine-tRNA ligase; oxidative stress-induced via Cap1
orf19.7295	orf19.7295	19.05	15	20.3	14.36	-0.006769832	NA	Ortholog(s) have protein phosphatase 1 binding activity, role in actin filament organization, cortical actin cytoskeleton organization, endocytosis, protein secretion and actin cortical patch, nucleus localization
orf19.6635	orf19.6635	19.67	14.99	13.61	8.21	-0.283893619	NA	Ortholog of C. dubliniensis CD36 : Cd36_31170, C. parapsilosis CDC317 : CPAR2_205340, Candida tenuis NRRL Y-1498 : CANTEDRAFT_133521 and Debaryomyces hansenii CBS767 : DEHA2E21868g
orf19.5292	AXL2	16.3	14.98	19.47	21.95	0.18814513	NA	Ortholog of S. cerevisiae Axl2; a plasma membrane protein involved in determination of budding pattern; O-glycosylated by Pmt4; mutant is viable
orf19.2799	GPI8	20.04	14.98	23.07	16.73	0.087274288	NA	Protein similar to S. cerevisiae Gpi8p, which is a subunit of the GPI transamidase complex that adds GPI anchors to proteins; likely to be essential for growth, based on an insertional mutagenesis strategy

orf19.1077	ATM1	16.06	14.97	13.74	14.01	-0.069118947	NA	Member of MDR subfamily of ABC family; ortholog of S. cerevisiae ABC transporter, Atm1; induced in low iron; induced by nitric oxide independent of Yhb1
orf19.5940	ZCF32	18.4	14.95	19.03	18.25	0.072879788	NA	Predicted Zn(II)2Cys6 transcription factor; possibly an essential gene, disruptants not obtained by UAU1 method; Hap43-repressed
orf19.3199	PIKA	19.05	14.94	21.68	19.24	0.126285551	NA	Putative phosphatidylinositol kinase; non-sex gene located within the MTLa mating-type-like locus
orf19.4764	orf19.4764	16.65	14.9	16.56	14.71	-0.005963126	NA	Ortholog(s) have poly(A)-specific ribonuclease activity, role in mRNA 3'-end processing, postreplication repair and PAN complex localization
orf19.2544	orf19.2544	15.56	14.9	18.1	16.8	0.074587171	NA	Ortholog(s) have aminoacyl-tRNA hydrolase activity, role in negative regulation of proteasomal ubiquitin-dependent protein catabolic process and mitochondrial outer membrane localization
orf19.4208	RAD52	20.93	14.9	16.39	15.62	-0.082767699	NA	Required for homologous DNA recombination, repair of UV- or MMS-damaged DNA, telomere length, UV-induced LOH; constitutive expression, MMS-induced; weakly complements S. cerevisiae rad52 mutant; slow growth, increased white-to-opaque switch
orf19.2926	orf19.2926	18.5	14.87	16.83	14.83	-0.045433431	NA	Putative nuclease required for DNA single- and double-strand break repair; rat catheter biofilm induced
orf19.6978	orf19.6978	9.89	14.84	10.48	8.01	-0.176073183	NA	Planktonic growth-induced gene
orf19.2076	orf19.2076	18.57	14.82	25.63	22.44	0.246618521	NA	Protein of unknown function; S. pombe ortholog SPAC7D4.05 encodes a predicted hydrolase; Hap43-repressed; Spider biofilm induced

orf19.4213	orf19.4213	17.1	14.82	16.76	15.57	0.014680346	NA	Putative iron transport multicopper oxidase precursor; flucytosine induced; caspofungin repressed
orf19.314	orf19.314	18.34	14.78	19.45	17.43	0.053371819	NA	Ortholog of S. cerevisiae : STB6, C. dubliniensis CD36 : Cd36_83110, C. parapsilosis CDC317 : CPAR2_103210, Candida tenuis NRRL Y-1498 : CANTEDRAFT_133418 and Debaryomyces hansenii CBS767 : DEHA2G08382g
orf19.3166	orf19.3166	14.99	14.76	23.07	20.18	0.242838515	NA	Ortholog(s) have role in Golgi to endosome transport, cell morphogenesis, cleistothecium development, conidium formation and endoplasmic reticulum organization, more
orf19.7355	SSN8	14.76	14.75	16.57	13	-0.007439902	NA	Ortholog of S. cerevisiae Ssn8; a component of RNA polymerase II holoenzyme; mutants are viable and are sensitive to hydrogen peroxide medium
orf19.5134	orf19.5134	17.18	14.74	10.99	11.63	-0.202016664	NA	Ortholog of C. parapsilosis CDC317 : CPAR2_704140, C. dubliniensis CD36 : Cd36_72870, Lodderomyces elongisporus NRLL YB-4239 : LELG_05208 and Candida orthopsilosis Co 90-125 : CORT_0G04320
orf19.2408	orf19.2408	13.31	14.69	12.13	9.61	-0.143897575	NA	Ortholog(s) have tRNA methyltransferase activity, role in tRNA methylation, wybutosine biosynthetic process and cytosol, nucleus localization
orf19.6458	orf19.6458	24.29	14.66	19.43	14.62	-0.098766008	NA	Ortholog of C. dubliniensis CD36 : Cd36_72260, C. parapsilosis CDC317 : CPAR2_702840, Candida tenuis NRRL Y-1498 : CANTEDRAFT_115664 and Debaryomyces hansenii CBS767 : DEHA2B05940g
orf19.2074	orf19.2074	21.98	14.65	15.28	11.95	-0.207658885	NA	Ortholog of Candida albicans WO-1 : CAWG_03833
orf19.6915	MRE11	17.61	14.63	17.65	11.61	-0.068333196	NA	Putative DNA double-strand break repair factor; involved in response to oxidative stress and drug resistance

orf19.6662	orf19.6662	16.04	14.61	9.24	7.11	-0.360748067	NA	Putative coenzyme Q (ubiquinone) binding protein; transcript is upregulated in clinical isolates from HIV+ patients with oral candidiasis
orf19.5179	LIP5	16.95	14.61	21.48	21.97	0.203188624	NA	Cold-activated secreted lipase, differentially expressed lipase gene family member with possible roles in nutrition and acidic microenvironment; LIP5 and LIP8 expressed at all stages of mucosal and systemic infection; affects filamentation
orf19.1555	SAC3	17.85	14.6	17.25	16.9	0.0150772	NA	Putative nuclear pore-associated protein, required for small ribosomal subunit biogenesis; possibly an essential gene, disruptants not obtained by UAU1 method
orf19.5391	orf19.5391	15.65	14.59	15.86	14.4	-0.027181442	NA	Predicted RNA splicing and ER to Golgi transport protein; Hap43-induced gene
orf19.56	ARG2	20.24	14.59	18.48	20.81	0.072340112	NA	Putative enzyme of arginine biosynthesis; transcription of genes of arginine biosynthesis pathway, except for ARG2, is induced upon phagocytosis by macrophage
orf19.4621	orf19.4621	15.28	14.58	19.42	15.23	0.074093573	NA	Ortholog(s) have cytoplasmic mRNA processing body localization
orf19.7629	orf19.7629	16.21	14.57	17.56	14.19	0.013444291	NA	Ortholog(s) have Seh1-associated complex, cytosol, extrinsic to fungal-type vacuolar membrane, ribosome localization
orf19.6912	orf19.6912	16.81	14.56	23.06	17.06	0.146316743	NA	Putative ethanolamine kinase
orf19.7426	orf19.7426	14.92	14.55	19.47	15.79	0.094266279	NA	Ortholog(s) have dolichyl-diphosphooligosaccharide-protein glycotransferase activity, role in protein N-linked glycosylation via asparagine, protein complex assembly and oligosaccharyltransferase complex localization

orf19.5052	orf19.5052	18.42	14.54	19.5	15.63	0.033582153	NA	Ortholog of S. cerevisiae : YFR045W, C. glabrata CBS138 : CAGL0C02013g, C. dubliniensis CD36 : Cd36_07340, C. parapsilosis CDC317 : CPAR2_208340 and Candida tenuis NRRL Y-1498 : CANTEDRAFT_116470
orf19.2081	POM152	16.14	14.54	18.62	16.58	0.093615056	NA	Putative nuclear pore membrane glycoprotein
orf19.5162	BCK1	16.25	14.53	15.38	13.66	-0.047997794	NA	Ortholog of S. cerevisiae Bck1; MAP kinase kinase kinase of cell integrity pathway; mutant is hypersensitive to caspofungin
orf19.7447	JEN1	18.64	14.52	20.3	13.79	-0.006769832	NA	Lactate transporter; induced by lactic acid, activated by Cat8; alkaline repressed by Rim101; localizes to plasma membrane; Spider biofilm induced
orf19.280	orf19.280	30	14.52	0.12	21.89	-0.100474477	NA	Ortholog of C. dubliniensis CD36 : Cd36_82875 and Candida albicans WO-1 : CAWG_02609
orf19.2042	orf19.2042	13.78	14.52	18.08	13.99	0.054651555	NA	Ortholog(s) have role in mitochondrial tRNA wobble uridine modification and mitochondrial inner membrane localization
orf19.345	orf19.345	16.79	14.51	9.57	6.67	-0.353756393	NA	Succinate semialdehyde dehydrogenase; for utilization of gamma-aminobutyrate (GABA) as a nitrogen source; part of 4-aminobutyrate and glutamate degradation pathways; rat catheter biofilm induced
orf19.4738	orf19.4738	15.51	14.51	20.22	20.76	0.187896809	NA	Ortholog(s) have role in protein folding in endoplasmic reticulum and integral to endoplasmic reticulum membrane localization
orf19.4791	orf19.4791	16.96	14.5	17.66	16.03	0.034255661	NA	Protein of unknown function; Spider biofilm induced

orf19.3660	orf19.3660	15.24	14.5	12.16	12.88	-0.114765341	NA	Ortholog of C. dubliniensis CD36 : Cd36_60600, C. parapsilosis CDC317 : CPAR2_602890, Candida tenuis NRRL Y-1498 : CANTEDRAFT_135512 and Debaryomyces hansenii CBS767 : DEHA2F15312g
orf19.1333	SNG3	17.23	14.49	12.23	9.19	-0.23019465	NA	Putative membrane transporter; Hap43p-induced gene; mutation confers hypersensitivity to toxic ergosterol analog; shows colony morphology-related gene regulation by Ssn6p
orf19.2494	orf19.2494	14.61	14.48	17.49	15.45	0.056513337	NA	Ortholog(s) have glutaminyl-tRNA synthase (glutamine-hydrolyzing) activity, role in glutaminyl-tRNAGln biosynthesis via transamidation, mitochondrial translation and glutamyl-tRNA(Gln) amidotransferase complex, mitochondrion localization
orf19.1136	orf19.1136	18.09	14.47	15.34	15.33	-0.045641511	NA	Ortholog(s) have AP-2 adaptor complex, cytosol, nucleus localization
orf19.5722	orf19.5722	14.46	14.46	15.34	11.21	-0.05124752	NA	Ortholog(s) have DNA binding activity
orf19.6205	orf19.6205	10.86	14.4	12.8	9.64	-0.054034707	NA	Ortholog of C. dubliniensis CD36 : Cd36_06550, C. parapsilosis CDC317 : CPAR2_209030, Candida tenuis NRRL Y-1498 : CANTEDRAFT_111035 and Debaryomyces hansenii CBS767 : DEHA2F25300g
orf19.4330	orf19.4330	15.37	14.39	18.22	17.61	0.136684796	NA	Ortholog of C. dubliniensis CD36 : Cd36_52790, Candida tropicalis MYA-3404 : CTRG_05968 and Candida albicans WO-1 : CAWG_04684
orf19.5441	ULP3	21.14	14.39	20.17	17.5	0.033692246	NA	SUMO deconjugation enzyme that removes the small ubiquitin-like modifier (SUMO) from proteins
orf19.1424	orf19.1424	14.39	14.39	20.94	16.5	0.194207721	NA	Predicted membrane transporter, member of the oxalate:formate antiporter (OFA) family, major facilitator superfamily (MFS)

orf19.2506	orf19.2506	19.13	14.37	27.64	17.94	0.228478792	NA	Protein of unknown function; opaque-specific transcript; induced during chlamydospore formation in both C. albicans and C. dubliniensis; Hog1-repressed; Spider biofilm induced
orf19.2928	orf19.2928	15.94	14.36	22.36	24.27	0.297038322	NA	Ortholog(s) have COPI-coated vesicle, Golgi apparatus localization
orf19.4964	orf19.4964	17.51	14.36	13.21	12.75	-0.132306437	NA	Ortholog(s) have role in cellular bud site selection, mRNA export from nucleus, mRNA splicing, via spliceosome and RES complex localization
orf19.6268	orf19.6268	14.45	14.36	18.22	13.81	0.076709774	NA	Putative cohesin complex subunit; expression downregulated in an ssr1 null mutant
orf19.6628	orf19.6628	17.01	14.35	14.05	19.08	0.037718737	NA	Protein of unknown function; rat catheter biofilm repressed
orf19.4768	orf19.4768	15.73	14.33	11.16	14.64	-0.089369517	NA	Protein of unknown function; Spider biofilm induced
orf19.5504	orf19.5504	16.24	14.31	15.81	14.97	0.01548811	NA	Ortholog of C. dubliniensis CD36 : Cd36_73400, C. parapsilosis CDC317 : CPAR2_703180, Candida tenuis NRRL Y-1498 : CANTEDRAFT_104803 and Debaryomyces hansenii CBS767 : DEHA2A04466g
orf19.4918	orf19.4918	21.23	14.31	14.04	15.7	-0.102819377	NA	Has domain(s) with predicted DNA binding activity
orf19.5863	orf19.5863	17.69	14.3	18.39	16.51	0.055051896	NA	Ortholog of C. dubliniensis CD36 : Cd36_84160, Candida tenuis NRRL Y-1498 : cten_CGOB_00085, Debaryomyces hansenii CBS767 : DEHA2B08404g and Pichia stipitis Pignal : PICST_67031

orf19.1538	TLG2	17.84	14.3	17.79	16.46	0.035025704	NA	Putative syntaxin-like t-SNARE; macrophage/pseudohyphal-repressed
orf19.295	orf19.295	13.13	14.25	12.99	9.27	-0.122040849	NA	Ortholog of C. dubliniensis CD36 : Cd36_82970, Lodderomyces elongisporus NRLL YB-4239 : LELG_02117, Debaryomyces hansenii CBS767 : DEHA2G01012g and Candida lusitaniae ATCC 42720 : CLUG_03529
orf19.2006	orf19.2006	14.46	14.24	14.84	13.14	-0.00648674	NA	Ortholog(s) have fungal-type vacuole membrane localization
orf19.6797	orf19.6797	17.36	14.22	18.91	16.28	0.074521472	NA	Ortholog of C. dubliniensis CD36 : Cd36_86960, C. parapsilosis CDC317 : CPAR2_808800, Candida tenuis NRRL Y-1498 : CANTEDRAFT_113621 and Debaryomyces hansenii CBS767 : DEHA2F02486g
orf19.3218	orf19.3218	16.01	14.22	16.85	13.42	-0.006563188	NA	Putative multidrug resistance protein; upregulated by Efg1p
orf19.4299	MSW1	16.56	14.21	14.18	13.39	-0.090445103	NA	Protein similar to S. cerevisiae Msw1p, which is mitochondrial tryptophanyl-tRNA synthetase; Hap43p-repressed gene; likely to be essential for growth, based on an insertional mutagenesis strategy
orf19.910	PRP3	17.27	14.2	15.56	14.86	-0.005043217	NA	Predicted splicing factor, component of the U4/U6-U5 snRNP complex; Hap43-induced gene; rat catheter biofilm induced
orf19.7604	orf19.7604	15.64	14.2	18.23	14.88	0.055470421	NA	Ortholog(s) have Rab guanyl-nucleotide exchange factor activity
orf19.2878	PGA15	15.35	14.16	11.77	15.3	-0.047237016	NA	Putative GPI-anchored protein

orf19.4734	orf19.4734	16.32	14.16	18.6	12.69	0.033834841	NA	Protein of unknown function; Hap43-repressed
orf19.4819	orf19.4819	11.33	14.15	12.99	9.6	-0.054034707	NA	Protein of unknown function; transcript detected on high-resolution tiling arrays; rat catheter biofilm induced
orf19.6469	orf19.6469	9.98	14.15	11.05	12.07	-0.0297914	NA	Predicted ORF in retrotransposon Tca11 with similarity to the Gag-Pol region of retrotransposons, which encodes nucleocapsid-like protein, reverse transcriptase, protease, and integrase
orf19.1451	SRB9	15.79	14.14	19.44	15.27	0.074951749	NA	Subunit of the RNA polymerase II mediator complex; transposon mutation affects filamentous growth; suppresses S. cerevisiae diploid filamentous (flo8, ste7, ste12, tec1) or haploid invasive (flo8) mutant growth defects
orf19.4781	GRP1	19.7	14.12	15.76	16.12	-0.043645789	NA	Protein similar to dihydroflavonol-4-reductases
orf19.3456	orf19.3456	16.23	14.12	15.41	14.41	-0.02657293	NA	Protein with a predicted serine/threonine kinase and tyrosine kinase domain; possibly an essential gene, disruptants not obtained by UAU1 method
orf19.4837	DAM1	15.04	14.12	14.77	16.12	0.037110042	NA	Essential subunit of the Dam1 (DASH) complex, which acts in chromosome segregation by coupling kinetochores to spindle microtubules
orf19.3964	ASH2	22.38	14.11	23.64	22.44	0.174728758	NA	Ortholog(s) have histone methyltransferase activity (H3-K4 specific) activity
orf19.5646	orf19.5646	11.07	14.11	15.45	16.83	0.143906706	NA	Ortholog of S. cerevisiae : YPL034W, C. glabrata CBS138 : CAGL0L06996g, C. dubliniensis CD36 : Cd36_40280, C. parapsilosis CDC317 : CPAR2_402170 and Candida tenuis NRRL Y-1498 : CANTEDRAFT_137348

orf19.277	THI6	14.7	14.09	15.02	12.73	-0.027655734	NA	Putative thiamin-phosphate pyrophosphorylase, hydroxyethylthiazole kinase; fungal-specific; Spider biofilm induced
orf19.6016	orf19.6016	18.05	14.09	13.2	13.28	-0.132306437	NA	Ortholog(s) have GTPase activity, calcium ion binding activity, role in mitochondrion inheritance, phospholipid homeostasis, regulation of mitochondrion organization and ERMES complex, cytosol, nuclear envelope localization
orf19.2873	TOP2	16.93	14.08	17.09	16.46	0.035734046	NA	DNA topoisomerase II; catalyzes ATP-dependent DNA relaxation and decatenation in vitro; Y842 predicted to be catalytic; functional homolog of S. cerevisiae Top2p; sensitive to amsacrine or doxorubicin; farnesol-upregulated in biofilm
orf19.4904	orf19.4904	19.09	14.07	15.01	12.64	-0.10876793	NA	Ortholog(s) have nucleosome binding activity, role in chromatin silencing at telomere, histone exchange and Swr1 complex, cytosol, nuclear chromosome, telomeric region localization
orf19.1092	orf19.1092	18.73	14.07	24.98	24.38	0.284380881	NA	Dolichol-P-Man dependent alpha(1-3) mannosyltransferase; role in the synthesis of dolichol-linked oligosaccharide donor for N-linked glycosylation of proteins; rat catheter biofilm repressed
orf19.1053	orf19.1053	16.63	14.07	20.89	20.56	0.206218726	NA	Ortholog(s) have phosphatidylinositol deacylase activity
orf19.6222.1	orf19.6222.1	10.82	14.06	30.22	12.94	0.329630853	NA	Ortholog of C. parapsilosis CDC317 : CPAR2_208910, Candida tenuis NRRL Y-1498 : CANTEDRAFT_114047, Debaryomyces hansenii CBS767 : DEHA2D14388g and Pichia stipitis Pignal : PICST_37629
orf19.4438	RME1	10.26	14.05	0.49	1.83	-0.651218403	NA	Zinc finger protein; controls meiosis in S. cerevisae; white-specific transcript; upregulation correlates with clinical development of fluconazole resistance; Upc2-regulated in hypoxia; flow model biofilm induced; Spider biofilm repressed
orf19.5621	orf19.5621	21.38	14.05	20.19	15.67	0.014346493	NA	Putative protein of unknown function; mutation confers hypersensitivity to amphotericin B; overlaps orf19.5621

orf19.673	orf19.673	19.97	14.04	15.53	13.33	-0.106958774	NA	Has domain(s) with predicted FMN binding, oxidoreductase activity and role in oxidation-reduction process
orf19.3633	orf19.3633	16.45	14.04	30.42	18.8	0.33861035	NA	Ortholog(s) have role in purine nucleobase catabolic process and cytosol, nucleus localization
orf19.7556	orf19.7556	14.33	14.03	22.63	19.64	0.286055666	NA	Ortholog of C. dubliniensis CD36 : Cd36_34965, C. parapsilosis CDC317 : CPAR2_200910, Candida tenuis NRRL Y-1498 : CANTEDRAFT_95984 and Debaryomyces hansenii CBS767 : DEHA2E02926g
orf19.6431	orf19.6431	15.45	14.02	16.35	13.98	0.015066074	NA	Protein of unknown function; Spider biofilm induced
orf19.4106	orf19.4106	15.64	14.02	12.91	13.98	-0.069073334	NA	Ortholog of S. cerevisiae : YJR141W, C. glabrata CBS138 : CAGL0M00264g, C. dubliniensis CD36 : Cd36_20520, C. parapsilosis CDC317 : CPAR2_104880 and Candida tenuis NRRL Y-1498 : CANTEDRAFT_126860
orf19.4410	ALG1	19.61	14.02	19.97	15.07	0.013940603	NA	Protein similar to S. cerevisiae Alg1p, a mannosyltransferase involved in N-linked protein glycosylation; likely to be essential for growth, based on an insertional mutagenesis strategy
orf19.2779	orf19.2779	13.77	14	11.09	11.8	-0.118161457	NA	Protein of unknown function; Hap43-repressed gene
orf19.1547	orf19.1547	17.55	14	21.26	16.46	0.091716399	NA	Ortholog(s) have double-stranded DNA binding, polynucleotide 3'-phosphatase activity and role in double-strand break repair
orf19.1142	AVT4	17.76	13.99	18.96	17.51	0.093519045	NA	Putative vacuolar transporter of large neutral amino acids; induced by alpha pheromone in SpiderM medium

orf19.5514	orf19.5514	11.13	13.96	11.57	6.37	-0.175369601	NA	Ortholog of S. pombe SPCC550.08, an N-acetyltransferase; transcript induced during growth in the mouse cecum
orf19.6968	orf19.6968	16.93	13.95	14.29	15.45	-0.046214448	NA	Protein of unknown function; Hog1-repressed; Spider biofilm induced
orf19.3983	orf19.3983	17.72	13.95	20.01	16.83	0.092701621	NA	Protein with a predicted transcription factor BTF3 domain; flow model biofilm induced
orf19.4550	orf19.4550	15.3	13.93	11.76	11.07	-0.140419528	NA	Predicted MFS membrane transporter, member of the drug:proton antiporter (12 spanner) (DHA1) family; flow model biofilm induced
orf19.6780	MET8	18.14	13.93	19.41	17.64	0.093519045	NA	Putative bifunctional dehydrogenase and ferrochelatase with a predicted role in siroheme biosynthesis; regulated by Gcn2p and Gcn4p
orf19.6359	orf19.6359	16.39	13.9	9.91	13.86	-0.134486828	NA	Ortholog(s) have endopeptidase activity, role in mitochondrial respiratory chain complex assembly, proteolysis and mitochondrial inner membrane peptidase complex localization
orf19.3563	orf19.3563	13.63	13.88	12.73	10.87	-0.096137865	NA	Ortholog of C. dubliniensis CD36 : Cd36_19810, C. parapsilosis CDC317 : CPAR2_206450, Candida tenuis NRRL Y-1498 : CANTEDRAFT_113905 and Debaryomyces hansenii CBS767 : DEHA2C05566g
orf19.1409.2	orf19.1409.2	10.22	13.87	20.39	19.65	0.31944004	NA	Protein of unknown function; Spider biofilm induced
orf19.5528	MOB1	14.11	13.87	17.53	13.6	0.076709774	NA	Putative mitotic exit network component; periodic mRNA expression, peak at cell-cycle G2/M phase

orf19.1144	orf19.1144	13.98	13.86	20.31	18.23	0.195282311	NA	Protein with SEL-1 like protein domain; early-stage flow model biofilm induced
orf19.2940	orf19.2940	13.06	13.84	10.68	10.68	-0.120870841	NA	Putative v-SNARE of the endoplasmic reticulum membrane; possibly an essential gene, disruptants not obtained by UAU1 method
orf19.119	orf19.119	9.3	13.83	11.18	15.22	0.061691948	NA	Ortholog(s) have protein C-terminal S-isoprenylcysteine carboxyl O-methyltransferase activity
orf19.4787	orf19.4787	14.33	13.82	15.89	12.27	-0.007293568	NA	Ortholog(s) have cytoplasm, nucleus localization
orf19.6624	orf19.6624	14.81	13.82	14.28	13.16	-0.049136541	NA	Ortholog(s) have GTPase activator activity and clathrin-coated vesicle, endosome localization
orf19.3886	orf19.3886	15.81	13.82	15.77	14.31	-0.00577873	NA	Ortholog of C. dubliniensis CD36 : Cd36_31820, C. parapsilosis CDC317 : CPAR2_205050, Debaryomyces hansenii CBS767 : DEHA2G10736g and Pichia stipitis Pignal : PICST_32993
orf19.2936	orf19.2936	15.88	13.79	13.75	11.25	-0.115450822	NA	Ortholog of C. parapsilosis CDC317 : CPAR2_106450, C. dubliniensis CD36 : Cd36_02330, Lodderomyces elongisporus NRLL YB-4239 : LELG_01618 and Candida orthopsilosis Co 90-125 : CORT_0B07690
orf19.2272	AFT2	17.86	13.78	22.27	18.5	0.166644123	NA	Putative Aft domain transcription factor; role in regulation of iron metabolism, oxidative stress, adhesion, hyphal growth, colony morphology, virulence; complements S. cerevisiae aft1 mutation; Spider biofilm induced
orf19.3688	orf19.3688	20.94	13.77	24.72	23.07	0.227471001	NA	Ortholog(s) have chromatin DNA binding activity and role in cell wall mannoprotein biosynthetic process, positive regulation of transcription from RNA polymerase II promoter, telomere maintenance

orf19.2838	orf19.2838	11.59	13.77	11.26	12.01	-0.074601716	NA	Protein of unknown function; mutation confers hypersensitivity to amphotericin B; flow model biofilm induced
orf19.1155	DPP2	13.1	13.76	19.48	22.57	0.29090135	NA	Protein similar to S. cerevisiae pyrophosphate phosphatase Lpp1p; possible role in farnesol biosynthesis
orf19.4921	orf19.4921	13.38	13.73	16.48	14.03	0.057682591	NA	Ortholog of C. dubliniensis CD36 : Cd36_12030 and Candida albicans WO-1 : CAWG_00152
orf19.3501	orf19.3501	11.48	13.7	16.18	13.95	0.101284345	NA	S. cerevisiae ortholog Pxl1 localizes to sites of polarized growth and is required for selection and/or maintenance of polarized growth sites; Hog1p-repressed
orf19.3433	OYE23	16.98	13.68	15	11.69	-0.091113393	NA	Putative NAPDH dehydrogenase; induced by nitric oxide, benomyl; oxidative stress-induced via Cap1; Hap43p-repressed; rat catheter biofilm induced
orf19.1170	ARO7	22.17	13.67	24.93	18.8	0.138880116	NA	Putative chorismate mutase; fungal-specific (no human or murine homolog); alkaline upregulated
orf19.860	BMT8	18.81	13.66	24.87	22.82	0.267582894	NA	Putative beta-mannosyltransferase, member of a 9-gene family including characterized BMT genes with roles in beta-1,2-mannosylation of cell wall phosphopeptidomannan; transposon insertion in promoter region causes decreased colony wrinkling
orf19.7313	SSU1	10.57	13.66	6.82	6.94	-0.283436948	NA	Protein similar to S. cerevisiae Ssu1 sulfite transport protein; Tn mutation affects filamentous growth; regulated by Gcn2 and Gcn4; induced by nitric oxide; Hap43-repressed; Spider and flow model biofilm induced
orf19.4858	VPS41	17.03	13.65	20.29	17.36	0.113046421	NA	Protein similar to S. cerevisiae Vps41p, which is involved in vacuole organization and biogenesis; transposon mutation affects filamentous growth; regulated by Gcn4p; induced in response to amino acid starvation (3-aminotriazole treatment)

orf19.1229	orf19.1229	18.7	13.65	13.21	13.03	-0.151977408	NA	Ortholog(s) have importin-alpha export receptor activity, role in cell division, protein export from nucleus and nuclear membrane localization
orf19.4901	orf19.4901	15.47	13.64	9.26	12.52	-0.160486586	NA	Predicted methyltransferase; Spider biofilm induced
orf19.2879	IFF5	14.2	13.64	18.97	17.61	0.176744142	NA	Putative GPI-anchored protein; adhesin-like protein; possibly an essential gene, disruptants not obtained by UAU1 method
orf19.5384	CHS8	13.35	13.62	20.61	21.54	0.3088433	NA	Chitin synthase required for synthesis of long-chitin fibrils; nonessential; 8 or 9 membrane spanning regions; mRNA present in yeast and hyphae; induced during cell wall regeneration; flow model biofilm repressed
orf19.6169	ATO1	15.27	13.59	66.86	32.82	0.982036066	0.016306	Putative fungal-specific transmembrane protein; induced by Rgt1; Spider biofilm induced
orf19.29	orf19.29	16.32	13.59	11.24	9.49	-0.234494383	NA	Ortholog of S. cerevisiae Tah11, a DNA replication licensing factor required for pre-replication complex assembly; rat catheter, flow model and Spider biofilm induced
orf19.7038	orf19.7038	19.18	13.55	19.71	16.15	0.053196133	NA	Ortholog(s) have phosphatidylinositol-3-phosphate binding activity, role in protein targeting to vacuole and cytoplasm, nucleus localization
orf19.340	orf19.340	24.5	13.53	21.86	17.35	-0.003955179	NA	Ortholog(s) have role in protein targeting to membrane, protein targeting to vacuole and ESCRT I complex localization
orf19.341	orf19.341	16.3	13.52	15.4	9.85	-0.115711716	NA	Putative spermidine export pump; fungal-specific (no human or murine homolog)

orf19.4924	orf19.4924	15.5	13.5	11.85	10.18	-0.142368643	NA	Protein of unknown function; repressed in ssr1 mutant; Spider biofilm induced
orf19.4916	orf19.4916	14.01	13.5	17.85	13.47	0.077979421	NA	Protein of unknown function; induced by alpha pheromone in SpiderM medium
orf19.1276	orf19.1276	15.05	13.5	11.16	13.85	-0.069766622	NA	Protein with a selenoprotein domain and a thioredoxin-like fold domain; similar to S. cerevisiae Vhs3p, which is a putative phosphopantothenoylcysteine decarboxylase
orf19.6723	orf19.6723	14.88	13.49	14.05	11.69	-0.049651	NA	Protein of unknown function; Spider biofilm induced
orf19.2381	orf19.2381	11.07	13.48	14.26	15.25	0.105024608	NA	Protein of unknown function; possibly an essential gene, disruptants not obtained by UAU1 method
orf19.6348	orf19.6348	16.75	13.48	19.04	14.03	0.055242278	NA	Predicted cysteine proteinase domain; mutants are viable
orf19.6910	PSF1	13.47	13.46	18.27	15.57	0.162339125	NA	Putative GINS complex subunit which is localized to DNA replication origins; implicated in assembly of the DNA replication machinery; flow model biofilm induced
orf19.4607	orf19.4607	14.76	13.46	21	18.14	0.214333061	NA	Possible Golgi membrane protein; Hap43-repressed; hypha induced; flow model biofilm induced; Spider biofilm induced
orf19.529.1	orf19.529.1	12.51	13.46	23.02	22.05	0.366813336	NA	Protein of unknown function

orf19.1060	orf19.1060	16.11	13.42	15.3	14.91	0.01673616	NA	Ortholog of Candida albicans WO-1 : CAWG_00968
orf19.5308	orf19.5308	22.67	13.41	27.22	19.37	0.170976294	NA	Protein of unknown function; induced by Rgt1
orf19.3317	MED22	13.98	13.4	11.62	11.04	-0.096720969	NA	RNA polymerase II mediator complex subunit
orf19.1367	MTW1	10.8	13.4	17.15	13.56	0.145351008	NA	Kinetochore component; amount of Nuf2p and Mtw1p protein detected at each centromere is consistent with a single kinetochore microtubule attachment site
orf19.5839	PDR17	13.05	13.39	9.93	10.78	-0.1217949	NA	Fungal-specific protein (no human or murine homolog); role in sensitivity to fluconazole, specifically
orf19.3115	orf19.3115	24.92	13.34	16.51	18.43	-0.057137652	NA	Protein of unknown function; rat catheter and Spider biofilm induced
orf19.7389	REV3	14	13.32	18.03	16.16	0.140440056	NA	Ortholog(s) have DNA-directed DNA polymerase activity, role in error-free translesion synthesis, error-prone translesion synthesis and cytosol, mitochondrion, nuclear chromatin, nucleus, zeta DNA polymerase complex localization
orf19.799	STE4	16.71	13.31	12.47	10.84	-0.15996202	NA	Beta subunit of heterotrimeric G protein of mating signal transduction pathway; required for mating; transcript is specific to cells homozygous at MTL; induced by alpha pheromone; ortholog of S. cerevisiae Ste4
orf19.102	orf19.102	20.35	13.31	14.48	17.15	-0.042182236	NA	Ortholog(s) have translation regulator activity, role in regulation of translation and mitochondrion localization

orf19.6884	GWT1	16.24	13.29	15.43	19.88	0.118865221	NA	Inositol acyltransferase with role in early steps of GPI anchor biosynthetic process; antifungal drug target
orf19.4348	MED14	18.23	13.28	13.88	12.32	-0.111743869	NA	RNA polymerase II mediator complex subunit
orf19.1037	orf19.1037	14.52	13.27	17.82	18.82	0.17817315	NA	Protein of unknown function; rat catheter biofilm repressed
orf19.7397	orf19.7397	14.45	13.26	15.53	14.23	0.058657444	NA	Ortholog(s) have sequence-specific DNA binding activity and cytoplasm, nucleus, spindle localization
orf19.5974	ATG9	20.22	13.26	13.23	12	-0.173176458	NA	Protein similar to S. cerevisiae Atg9; required for early step in autophagy; required for cytoplasm to vacuole trafficking of Lap41; Spider biofilm induced
orf19.4257	INT1	11.59	13.26	15.25	12.22	0.037610316	NA	Protein structurally similar to alpha-subunit of human leukocyte integrin; role in morphogenesis, adhesion, and mouse cecal colonization and systemic virulence; similar to S. cerevisiae Bud4; Hap43-induced gene
orf19.5467	TLO7	0	13.25	9.6	28.88	0.166197286	NA	Member of a family of telomere-proximal genes of unknown function; may be spliced in vivo; rat catheter biofilm repressed
orf19.7035	RFC2	16.01	13.25	14.59	16.82	0.058770431	NA	Putative heteropentameric replication factor C subunit; periodic mRNA expression, peak at cell-cycle G1/S phase
orf19.7082	orf19.7082	19.04	13.23	17.83	25	0.203315658	NA	S-adenosylmethionine transporter of the mitochondrial inner membrane; mitochondrial carrier family; predicted role in biotin biosynthesis and respiratory growth; Spider biofilm repressed

orf19.3819	orf19.3819	12.67	13.22	18.13	14.28	0.120924207	NA	Protein of unknown function; transcript detected on high-resolution tiling arrays
orf19.6199	orf19.6199	16.43	13.18	17.07	14.25	0.036344651	NA	Ortholog(s) have ATP-dependent 5'-3' DNA helicase activity and DNA helicase A complex, alpha DNA polymerase:primase complex, cytosol localization
orf19.315	orf19.315	15.07	13.18	17.54	18.78	0.17817315	NA	Ortholog of C. dubliniensis CD36 : Cd36_83120, C. parapsilosis CDC317 : CPAR2_103220, Candida tenuis NRRL Y-1498 : CANTEDRAFT_112860 and Debaryomyces hansenii CBS767 : DEHA2G08360g
orf19.6916	orf19.6916	20.66	13.18	18.84	17.65	0.054294134	NA	Ortholog(s) have unfolded protein binding activity, role in mitochondrial proton-transporting ATP synthase complex assembly and mitochondrion localization
orf19.3708	SAP2	13.78	13.15	18.12	19.67	0.218931291	NA	Major secreted aspartyl proteinase; utilization of protein as nitrogen source; role in virulence complicated by URA3 effects; immunoprotective; regulated by growth, albumin, drugs, white cell-type; flow model biofilm induced
orf19.6872	orf19.6872	14.38	13.14	19.05	24.37	0.309577952	NA	Putative protein of unknown function; Hap43p-repressed gene
orf19.1162.1	orf19.1162.1	24.82	13.14	14.2	12.01	-0.24200935	NA	Ortholog(s) have Golgi apparatus localization
orf19.6732	orf19.6732	10.72	13.12	10.91	14.39	0.017916746	NA	Ortholog(s) have cytosol, nucleus localization
orf19.6349	RVS162	18.52	13.09	16.23	19.39	0.056951036	NA	Protein containing a BAR domain, which is found in proteins involved in membrane curvature; null mutant does not display the endocytic, hyphal growth, virulence, or cell wall defects exhibited by mutants in related genes RVS161 and RVS167

orf19.7158	orf19.7158	13.86	13.08	12.71	14.38	-0.004683877	NA	Protein of allantoate permease family; fungal-specific (no human or murine homolog); Hap43p-repressed gene
orf19.4917	orf19.4917	15.23	13.08	9.97	11.27	-0.165214753	NA	Ortholog of Candida albicans WO-1 : CAWG_00155
orf19.3468	ALG11	14.09	13.07	21.73	16.47	0.215233162	NA	Alpha-1,2-mannosyltransferase; catalyzes sequential addition of 2 terminal alpha 1,2-mannose residues to the Man5GlcNAc2-PP-dolichol intermediate during asparagine-linked glycosylation in the ER; Spider biofilm induced
orf19.7401	ISW2	13.86	13.04	12.61	11.21	-0.073718312	NA	Ortholog of S. cerevisiae Isw2; an ATPase involved in chromatin remodeling; required for chlamydospore formation; Hap43-induced gene; repressed by high-level peroxide stress
orf19.5297	orf19.5297	14.78	13.02	15.43	12.3	-0.027822566	NA	Ortholog(s) have phosphatidylinositol-3-phosphate binding, phosphatidylinositol-5-phosphate binding activity
orf19.4497	MED8	17.54	13.02	15.9	12.72	-0.0467641	NA	Ortholog(s) have RNA polymerase II core promoter proximal region sequence-specific DNA binding, RNA polymerase II transcription corepressor activity, TBP-class protein binding, structural molecule activity
orf19.5784	AMO1	18.67	13.02	14.55	14.19	-0.066031593	NA	Putative peroxisomal copper amine oxidase
orf19.4091	MED18	12.83	13.01	18.49	14.39	0.120924207	NA	RNA polymerase II mediator complex subunit; rat catheter biofilm repressed
orf19.1998	orf19.1998	17.38	13.01	22.03	20.46	0.226662557	NA	Ortholog of C. dubliniensis CD36 : Cd36_16140, C. parapsilosis CDC317 : CPAR2_213870, Candida tenuis NRRL Y-1498 : CANTEDRAFT_92202 and Debaryomyces hansenii CBS767 : DEHA2A01980g

orf19.3601	orf19.3601	15.49	13	18.04	13.41	0.056530313	NA	Has domain(s) with predicted ATP binding, nucleoside-triphosphatase activity
orf19.209	DFG10	12.75	12.97	15.83	19.17	0.183354102	NA	Ortholog(s) have role in dolichol biosynthetic process, dolichol-linked oligosaccharide biosynthetic process, polyprenol catabolic process, pseudohyphal growth and endoplasmic reticulum localization
orf19.1624	orf19.1624	15.19	12.96	16.3	15.14	0.058410646	NA	Non-catalytic subunit of N-terminal acetyltransferase of the NatC type; flow model biofilm repressed
orf19.5472	orf19.5472	12.96	12.96	22.21	27.84	0.449891507	NA	Protein of unknown function
orf19.4817	RAM2	12.33	12.94	12.5	10.85	-0.029616305	NA	Alpha subunit of heterodimeric protein geranylgeranyltransferase type I and farnesyltransferase; a-specific transcript; lovastatin, fluconazole regulated; GGTase I binds zinc, is Mg-dependent; Cdc42 substrate; rat catheter biofilm repressed
orf19.438	orf19.438	16.13	12.94	14.29	13.86	-0.025911432	NA	Ortholog of S. cerevisiae : YHR078W, C. glabrata CBS138 : CAGL0J10626g, C. dubliniensis CD36 : Cd36_05020, Candida tenuis NRRL Y-1498 : CANTEDRAFT_95811 and Debaryomyces hansenii CBS767 : DEHA2E07524g
orf19.4378	PPH3	12.12	12.94	16.6	12.29	0.079792033	NA	Putative catalytic subunit of protein phosphatase complex; functions with regulatory subunit Psy2p in dephosphorylation of Rad53p in response to DNA damage; dephosphorylates Rfa2p in G1 phase; ortholog of S. cerevisiae Pph3p
orf19.1400	orf19.1400	15.97	12.92	16.1	14.28	0.015930961	NA	Ortholog of C. dubliniensis CD36 : Cd36_23640, C. parapsilosis CDC317 : CPAR2_406130, Candida tenuis NRRL Y-1498 : CANTEDRAFT_116273 and Debaryomyces hansenii CBS767 : DEHA2B11176g
orf19.4105	orf19.4105	15.5	12.91	14.47	10.3	-0.095704112	NA	Ortholog(s) have role in DNA repair, DNA replication checkpoint, establishment of mitotic sister chromatid cohesion, maintenance of DNA repeat elements, meiotic chromosome segregation, replication fork arrest, replication fork protection

orf19.17	SCP1	19.87	12.91	14.86	11.22	-0.151384982	NA	Putative cortical actin cytoskeleton protein; constitutive expression independent of MTL or white-opaque status
orf19.7130	orf19.7130	11.42	12.91	18.83	21.79	0.340594647	NA	Protein of unknown function; Hap43-repressed gene
orf19.1725	orf19.1725	10.35	12.9	14.22	13.62	0.105840365	NA	Putative adhesin-like protein
orf19.6137	orf19.6137	16.91	12.9	13.16	12.08	-0.113645138	NA	Ortholog of C. dubliniensis CD36 : Cd36_32940, Candida tenuis NRRL Y-1498 : CANTEDRAFT_134955, Debaryomyces hansenii CBS767 : DEHA2E04422g and Pichia stipitis Pignal : PICST_33357
orf19.4295	orf19.4295	13.49	12.89	19.16	14.47	0.140541638	NA	Ortholog(s) have DNA binding, nucleosome binding, transcription corepressor activity
orf19.5065	ERD1	13.42	12.89	13.92	12.06	-0.006306915	NA	Putative membrane protein required for the retention of lumenal endoplasmic reticulum proteins; rat catheter biofilm induced
orf19.5566	orf19.5566	15.22	12.88	12.58	10.25	-0.118822243	NA	Ortholog(s) have role in protein lipoylation and mitochondrion localization
orf19.4929	orf19.4929	15.37	12.87	14.76	14.39	0.016346704	NA	Ortholog(s) have translation regulator activity and role in RNA metabolic process, aerobic respiration, mitochondrial respiratory chain complex IV biogenesis, positive regulation of mitochondrial translational initiation
orf19.7375	orf19.7375	11.62	12.87	13.15	10.64	-0.029616305	NA	Putative U1A component of the U1 snRNP, involved in splicing; contains two RNA recognition motifs (RRMs); ortholog of S. cerevisiae MUD1

orf19.6981	orf19.6981	9.55	12.86	11.38	9.72	-0.054696295	NA	Ortholog of S. cerevisiae : YKR051W, C. glabrata CBS138 : CAGL0K08228g, C. dubliniensis CD36 : Cd36_85420, C. parapsilosis CDC317 : CPAR2_405720 and Candida tenuis NRRL Y-1498 : CANTEDRAFT_124861
orf19.6848	VPS16	12.77	12.83	15.58	11.11	0.014554122	NA	Protein similar to S. cerevisiae Vps16p, which is involved in protein-vacuolar targeting; likely to be essential for growth, based on insertional mutagenesis; downregulated in biofilm or in azole-resistant strain that overexpresses MDR1
orf19.4416	VPS13	11.64	12.8	16.47	17.13	0.165516963	NA	Putative vacuolar protein sorting-associated protein; gene used for multilocus sequence typing
orf19.139	TRA1	11.26	12.8	13.96	16.57	0.147333844	NA	Subunit of the NuA4 histone acetyltransferase complex
orf19.6868	orf19.6868	14.46	12.78	13.31	12.56	-0.027325661	NA	Putative NADPH-dependent methylglyoxal reductase; homozygous transposon insertion causes decreased colony wrinkling under filamentous growth-inducing conditions, but does not block true hyphal formation in liquid media
orf19.4680	orf19.4680	12.28	12.77	14.86	14.53	0.103489417	NA	Possile protease; mutation confers hypersensitivity to toxic ergosterol analog
orf19.4019	orf19.4019	8.08	12.74	20.15	17.97	0.350907835	NA	Ortholog(s) have protein phosphatase regulator activity, role in negative regulation of DNA damage checkpoint, protein dephosphorylation and cytoplasm, nucleus localization
orf19.2828	orf19.2828	9.17	12.74	18.73	16.05	0.270691606	NA	Ortholog(s) have alpha-tubulin binding, microtubule binding activity, role in establishment or maintenance of cell polarity, post-chaperonin tubulin folding pathway and cytosol, nucleus localization
orf19.3498	orf19.3498	14.98	12.74	10.47	8.41	-0.242210447	NA	Ortholog of C. dubliniensis CD36 : Cd36_62270, C. parapsilosis CDC317 : CPAR2_602240, Candida tenuis NRRL Y-1498 : CANTEDRAFT_114419 and Debaryomyces hansenii CBS767 : DEHA2E04884g

orf19.1659	ALG8	13.05	12.73	13.22	11.54	-0.028474942	NA	Putative glucosyltransferase involved in cell wall mannan biosynthesis; transcription is elevated in nik1 and sln1 homozygous null mutants, but not in the chk1 null mutant; possibly an essential gene, disruptants not obtained by UAU1 method
orf19.6826	SLF1	15.86	12.71	16.91	13.3	0.015088053	NA	Putative polysome-associated RNA binding protein; macrophage-induced gene
orf19.5246	orf19.5246	13.66	12.69	19.4	16.34	0.159676384	NA	Protein of unknown function; Hap43-repressed gene
orf19.1684	orf19.1684	13.91	12.69	16.73	14.74	0.100132948	NA	Ortholog of C. dubliniensis CD36 : Cd36_81500, C. parapsilosis CDC317 : CPAR2_503620, Candida tenuis NRRL Y-1498 : CANTEDRAFT_93693 and Debaryomyces hansenii CBS767 : DEHA2D15268g
orf19.3506	DBR1	12.95	12.68	16.51	10.91	0.035785123	NA	Debranchase; homozygous mutant accumulates lariat intermediates of mRNA splicing; rat catheter biofilm repressed
orf19.3265	TRM1	16.01	12.67	18.85	16.96	0.136615358	NA	Putative N2,N2-dimethylguanine tRNA methyltransferase; induced upon adherence to polystyrene
orf19.2158	NAG3	12.78	12.66	8.9	8.35	-0.224622196	NA	Putative MFS transporter; similar to Nag4; required for wild-type mouse virulence and cycloheximide resistance; in gene cluster that includes genes encoding enzymes of GlcNAc catabolism; Spider biofilm repressed
orf19.2064	orf19.2064	14.38	12.66	14.17	14.17	0.016757531	NA	Putative transcription factor with zinc finger DNA-binding motif
orf19.4593.1	orf19.4593.1	11.76	12.66	7.22	9.92	-0.198643795	NA	Protein with a predicted role in mitochondrial respiratory chain complex II assembly; rat catheter biofilm induced

orf19.5451	orf19.5451	21.08	12.64	18.92	13.66	-0.024333108	NA	Ortholog of Candida guilliermondii ATCC 6260 : PGUG_05321, Candida lusitaniae ATCC 42720 : CLUG_00887 and Candida albicans WO-1 : CAWG_02354
orf19.5012	orf19.5012	15.56	12.63	15.84	14.21	0.015930961	NA	Ortholog of C. dubliniensis CD36 : Cd36_12740, C. parapsilosis CDC317 : CPAR2_201620, Candida tenuis NRRL Y-1498 : CANTEDRAFT_107586 and Debaryomyces hansenii CBS767 : DEHA2D04994g
orf19.2685	PGA54	5.49	12.63	9.68	7.85	-0.0094546	NA	GPI-anchored protein; Hog1-repressed; induced in cyr1 or efg1 mutant or in hyphae; colony morphology-related gene regulation by Ssn6; induced in RHE model; mRNA binds She3; regulated in Spider biofilms by Tec1, Egf1, Ntd80, Rob1, Brg1
orf19.6371	orf19.6371	12.77	12.61	15.22	10.97	-0.007137677	NA	Ortholog of C. dubliniensis CD36 : Cd36_33750, C. parapsilosis CDC317 : CPAR2_205650, Candida tenuis NRRL Y-1498 : cten_CGOB_00253 and Debaryomyces hansenii CBS767 : DEHA2A11088g
orf19.4064	GPI7	12.34	12.61	16.04	13.55	0.102654004	NA	Protein involved in attachment of GPI-linked proteins to cell wall; member of major facilitator superfamily; phosphodiesterase/nucleotide pyrophosphatase domain; similar to S. cerevisiae Gpi7p
orf19.4210	orf19.4210	13.94	12.6	14.98	12.96	0.015926097	NA	Ortholog(s) have phospholipase A2 activity, role in cardiolipin acyl-chain remodeling and mitochondrion localization
orf19.3012	ARO80	13.4	12.59	16.46	9.86	-0.007950517	NA	Zn(II)2Cys6 transcription factor; transcriptional activator of aromatic amino acid catabolism; regulator of aromatic alcohol biosynthesis via the Ehrlich pathway; mutant is viable
orf19.668	TOS4	10.08	12.59	9.3	7.41	-0.182363292	NA	Putative fork-head transcription factor; rat catheter and Spider biofilm repressed
orf19.6606	orf19.6606	16.28	12.58	18.42	19.36	0.156778323	NA	Ortholog of C. parapsilosis CDC317 : CPAR2_201040, Candida tenuis NRRL Y-1498 : CANTEDRAFT_112677, Debaryomyces hansenii CBS767 : DEHA2E02420g and Pichia stipitis Pignal : PICST_37559

orf19.3252	DAL81	11.39	12.58	14.36	9.06	-0.031459286	NA	Zn(II)2Cys6 transcription factor; ortholog of S. cerevisiae Dal81, involved in the regulation of nitrogen-degradation genes; required for yeast cell adherence to silicone substrate; Spider biofilm induced
orf19.1251	BRN1	14.78	12.55	16.55	15.14	0.078558077	NA	Putative condensin complex subunit; cell-cycle regulated periodic mRNA expression
orf19.3639	orf19.3639	9.72	12.54	10.57	10.04	-0.054696295	NA	Ortholog(s) have alkylbase DNA N-glycosylase activity and role in DNA dealkylation involved in DNA repair, base-excision repair, AP site formation
orf19.3280	orf19.3280	14.63	12.53	24.49	8.85	0.088556821	NA	Ortholog of Candida albicans WO-1 : CAWG_01435
orf19.4719	CWH41	16.94	12.5	22.14	18.51	0.208257847	NA	Processing alpha glucosidase I, involved in N-linked protein glycosylation and assembly of cell wall beta 1,6 glucan; rat catheter biofilm repressed
orf19.2619	PHO113	11.99	12.5	23.95	22.02	0.405963307	NA	Putative constitutive acid phosphatase; Rim101-repressed; DTT-extractable; N-glycosylated; possibly an essential gene, disruptants not obtained by UAU1 method
orf19.3156	orf19.3156	10.36	12.5	16.23	21.17	0.287950462	NA	Protein of unknown function; induced by Mnl1 under weak acid stress
orf19.7304	orf19.7304	15.5	12.47	14.83	12.56	0.016812881	NA	Protein of unknown function; Hap43-induced; transcript induced early in infection of reconstituted human epithelium, while expression of the C. dubliniensis ortholog is not upregulated; mutants are viable; rat catheter biofilm repressed
orf19.3362	orf19.3362	10.13	12.45	9.62	8.96	-0.08011673	NA	Has domain(s) with predicted flavin adenine dinucleotide binding, oxidoreductase activity and role in oxidation-reduction process

orf19.2540	SAS3	12.98	12.44	11.27	13.62	-0.003560802	NA	Similar to silencing proteins; increased transcription is observed upon fluphenazine treatment; rat catheter biofilm repressed
orf19.81	orf19.81	15.97	12.44	13.35	13.35	-0.047775163	NA	Ortholog(s) have role in exonucleolytic trimming to generate mature 3'-end of 5.8S rRNA from tricistronic rRNA transcript (SSU-rRNA, 5.8S rRNA and LSU-rRNA), more
orf19.3852	orf19.3852	12.44	12.44	17.5	15.33	0.167880959	NA	Has domain(s) with predicted carbohydrate binding, catalytic activity and role in carbohydrate metabolic process
orf19.3899	SDC1	7.25	12.43	11.36	10.84	0.065475632	NA	Putative COMPASS/SET1C histone methyltransferase complex subunit
orf19.926	EXO1	14.27	12.42	14.85	10.54	-0.006205253	NA	Putative exodeoxyribonuclease; cell-cycle regulated periodic mRNA expression
orf19.949	orf19.949	14.17	12.42	14.35	12.46	-0.005374085	NA	Ortholog of C. dubliniensis CD36 : Cd36_50420, C. parapsilosis CDC317 : CPAR2_304100, Candida tenuis NRRL Y-1498 : CANTEDRAFT_98299 and Debaryomyces hansenii CBS767 : DEHA2B07634g
orf19.3592	JEM1	15.03	12.41	16.92	12.37	0.036882865	NA	Functional homolog of S. cerevisiae Jem1p, which acts with Scj1p and Kar2p (BiP) in protein folding and ER-associated degradation of misfolded proteins, and also has a role in karyogamy; has J domain and 4 tetratricopeptide repeats
orf19.6413	orf19.6413	14.3	12.4	11.27	9.67	-0.121848166	NA	Protein of unknown function; rat catheter biofilm induced
orf19.5142	DFR1	12.72	12.38	17.03	17.36	0.18640073	NA	Trimethoprim resistant dihydrofolate reductase (DHFR), reduces 7,8-dihydrofolate to 5,4,7,8-tetrahydrofolate; binds NADPH; target of drugs that selectively inhibit the fungal enzyme rather than the human enzyme

orf19.3992	orf19.3992	7.17	12.38	7.15	11.04	-0.029978703	NA	BLOC-1 complex subunit involved in endosomal maturation; Predicted ORF from Assembly 19; removed from Assembly 20; subsequently reinstated in Assembly 21 based on comparative genome analysis
orf19.2369	orf19.2369	14.74	12.37	12.71	10.59	-0.07266896	NA	Ortholog(s) have ATP binding, DNA replication origin binding activity
orf19.1720	orf19.1720	18.61	12.36	13.39	7.93	-0.225596764	NA	Ortholog(s) have role in mitotic recombination
orf19.487	SPT14	14.15	12.36	20.69	15.75	0.219782965	NA	Putative DNA-binding transcription factor; predicted role in regulation of biogenesis of the cell wall; upregulated in biofilm; protein newly produced during adaptation to the serum
orf19.3543	orf19.3543	16.47	12.35	18.78	16.42	0.138545787	NA	Dubious open reading frame
orf19.6103	orf19.6103	16.46	12.34	11.94	11.19	-0.116958977	NA	Ortholog(s) have role in ascospore wall assembly and cytosol localization
orf19.3898	orf19.3898	12.91	12.34	10.91	10.35	-0.099642671	NA	Ortholog(s) have SNAP receptor activity, role in endocytosis, vesicle fusion and Golgi apparatus, SNARE complex, endoplasmic reticulum, endosome, trans-Golgi network localization
orf19.5380	LYS144	15.33	12.33	14.65	11.66	-0.005426669	NA	Zn(II)2Cys6 transcription factor; has similarity to S. cerevisiae Lys14, involved in the regulation of lysine biosynthesis genes
orf19.3279	HYR4	13.68	12.33	12.35	11.64	-0.050050229	NA	Putative GPI-anchored adhesin-like protein; Rim101-repressed; constitutive expression independent of MTL or white-opaque status

orf19.5508	orf19.5508	10.22	12.32	8.07	6.75	-0.185328381	NA	Ortholog of Candida albicans WO-1 : CAWG_05700
orf19.2760	orf19.2760	11.53	12.3	12.74	10.55	-0.006115087	NA	Putative mRNA cleavage and polyadenylation factor; heterozygous null mutant exhibits hypersensitivity to parnafungin and cordycepin in the C. albicans fitness test
orf19.5237	VPH2	9.6	12.3	15.26	15.25	0.173161221	NA	Ortholog(s) have role in vacuolar acidification, vacuolar proton-transporting V-type ATPase complex assembly and endoplasmic reticulum membrane localization
orf19.6028	HGC1	9.4	12.29	15.15	16	0.217486703	NA	Hypha-specific G1 cyclin-related protein involved in regulation of morphogenesis, biofilm formation; Cdc28-Hgc1 maintains Cdc11 S394 phosphorylation during hyphal growth; required for virulence in mice; regulated by Nrg1, Tup1, farnesol
orf19.276	orf19.276	16.11	12.27	14.35	15.79	0.039444726	NA	Plasma membrane-associated protein; upregulated in an azole-resistant strain that overexpresses MDR1; Hap43-repressed; Spider biofilm induced
orf19.6921	orf19.6921	13.38	12.26	13.26	12.38	-0.0053125	NA	Ortholog(s) have RNA polymerase I upstream control element sequence-specific DNA binding transcription factor activity and role in chromatin organization, transcription of nuclear large rRNA transcript from RNA polymerase I promoter
orf19.1490.1	orf19.1490.1	16.59	12.26	26.62	29.49	0.479038268	NA	Predicted ORF in retrotransposon Tca16 with similarity to the Gag-Pol region of retrotransposons; added to Assembly 21 based on comparative genome analysis; overlaps orf19.1490.2, which is annotated as blocked reading frame
orf19.5760	IHD1	11.77	12.24	100.1	55.34	1.591145048	1.75E-06	GPI-anchored protein; alkaline, hypha-induced; regulated by Nrg1, Rfg1, Tup1 and Tsa1, Tsa1B in minimal media at 37; oralpharyngeal candidasis induced ; Spider biofilm induced; regulated in Spider biofilms by Tec1, Efg1, Ndt80, Rob1, Brg1
orf19.3314	TRS20	10.03	12.2	17.39	18.69	0.294601322	NA	Ortholog(s) have role in ER to Golgi vesicle-mediated transport and TRAPP complex, cytosol, nucleus localization

orf19.3982	orf19.3982	11.99	12.2	15.77	16.72	0.189288726	NA	Maltase; induced during growth on sucrose; induced by alpha pheromone in SpiderM medium; early-stage flow model biofilm indced
orf19.302	orf19.302	20.74	12.2	21.09	11.76	-0.005591737	NA	Ortholog(s) have Rab guanyl-nucleotide exchange factor activity, role in ER to Golgi vesicle-mediated transport and TRAPP complex, cytosol, nucleus localization
orf19.1595	orf19.1595	14.25	12.18	15.63	11.39	0.015476145	NA	Ortholog(s) have Dsc E3 ubiquitin ligase complex, endoplasmic reticulum localization
orf19.69	orf19.69	7.51	12.17	11.62	9.81	0.040572445	NA	Protein of unknown function; induced by Mnl1 under weak acid stress
orf19.2426	orf19.2426	8.03	12.16	15.05	9.46	0.084453278	NA	Predicted HD domain metal dependent phosphohydrolase; Spider biofilm repressed
orf19.3791	FGR10	10.17	12.16	11.33	9.94	-0.030550471	NA	Putative asparaginase; lacks ortholog in S. cerevisiae; transposon mutation affects filamentous growth; Spider biofilm induced
orf19.354	orf19.354	16.07	12.14	19.05	16.77	0.158425318	NA	Ortholog(s) have aminopeptidase activity, role in protein processing, protein stabilization and extrinsic to mitochondrial inner membrane, nucleus localization
orf19.1532	SAM37	8.26	12.12	10.16	14.53	0.111711708	NA	Protein of the mitochondrial outer membrane, component of the Sorting and Assembly Machinery (SAM) involved in insertion of proteins into the outer membrane; mutants show loss of mtDNA
orf19.2597	MRS2	13	12.06	11.9	11.37	-0.051648342	NA	Putative magnesium ion transporter, mitochondrial; fungal-specific (no human or murine homolog)

orf19.6261	BPH1	15.97	12.05	16.78	13.89	0.058267448	NA	Ortholog of S. cerevisiae Bph1; a putative ortholog of human Chediak-Higashi syndrome protein and murine beige gene implicated in disease syndromes involving defective lysosomal trafficking; mutant is viable
orf19.6698	orf19.6698	15.09	12.04	15.17	11.63	-0.005426669	NA	Ortholog(s) have asparagine-tRNA ligase activity, role in mitochondrial asparaginyl-tRNA aminoacylation and mitochondrion localization
orf19.4703	orf19.4703	11.86	12.03	13.73	14.55	0.106266413	NA	Protein with similarity to mutator-like element (MULE) transposase
orf19.6357	orf19.6357	17.07	12.03	15.79	13.58	0.016755965	NA	Ortholog(s) have role in mitotic DNA integrity checkpoint, negative regulation of protein import into nucleus during spindle assembly checkpoint, nucleocytoplasmic transport
orf19.6308	orf19.6308	17.67	12.02	20.31	15.16	0.095100662	NA	Ortholog of Candida albicans WO-1 : CAWG_01818
orf19.328	NPR2	15.5	11.95	16.2	11.26	-0.006237655	NA	Putative urea transporter; induced during infection of murine kidney, compared to growth in vitro; has murine homolog
orf19.2971	orf19.2971	11.43	11.95	13.38	15.15	0.107882944	NA	Ortholog of C. dubliniensis CD36 : Cd36_02630, C. parapsilosis CDC317 : CPAR2_108390, Candida tenuis NRRL Y-1498 : CANTEDRAFT_115724 and Debaryomyces hansenii CBS767 : DEHA2D07986g
orf19.3307	orf19.3307	13.94	11.93	14.84	11.49	-0.006205253	NA	Flavin-containing monooxygenase; catalyzes oxidation of biological thiols to maintain the ER redox buffer ratio for correct folding of disulfide-bonded proteins; Spider biofilm repressed
orf19.1028	ELA1	10.5	11.93	7.3	6.82	-0.236579892	NA	Elongin A; F-box protein; ubiquitin-protein ligase; role in protein ubiquitination and ubiquitin-dependent protein catabolism; rat catheter biofilm induced

orf19.5816	EBP7	13.21	11.92	17.53	14.52	0.16502599	NA	Putative NADPH oxidoreductase; mutation confers hypersensitivity to toxic ergosterol analog; oxidative stress-induced via Cap1p
orf19.3417	ACF2	10.9	11.9	13.29	9.94	-0.006934208	NA	Putative endo-1,3-beta-glucanase; fungal-specific (no human or murine homolog)
orf19.1745	orf19.1745	19.41	11.88	17.38	14.61	0.016684285	NA	Protein of unknown function; Hap43-repressed gene
orf19.6306	orf19.6306	17.07	11.87	26.03	20.22	0.314932886	NA	Trimethylaminobutyraldehyde dehydrogenase, the third enzyme of the carnitine biosynthesis pathway
orf19.4524	ZCF24	14.05	11.85	14.37	15.15	0.061405681	NA	Predicted Zn(II)2Cys6 transcription factor; caspofungin induced; Hap43-repressed
orf19.2463	PRN2	12.23	11.85	13.89	12.19	0.039425745	NA	Protein similar to pirin; Hap43p-repressed gene
orf19.313	DAL4	11.66	11.85	15.35	14.01	0.105548146	NA	Putative allantoin permease; fungal-specific (no human or murine homolog)
orf19.4985	GUP1	15.34	11.84	24.68	18.03	0.305984179	NA	Putative O-acyltransferase with a role in glycerol uptake; functionally complements growth of S. cerevisiae gup1 mutant under salt stress; required for normal ergosterol distribution, hyphal growth, biofilm formation
orf19.6350	orf19.6350	5.74	11.83	10.73	11.08	0.089396738	NA	Ortholog of C. dubliniensis CD36 : Cd36_11890 and Candida albicans WO-1 : CAWG_00170

orf19.2195	orf19.2195	20.8	11.83	14.66	17.52	-0.001082034	NA	Ortholog of Candida albicans WO-1 : CAWG_05881
orf19.1343	MED16	11.53	11.81	16.95	13.81	0.146703262	NA	Putative RNA polymerase II mediator complex subunit; induced by nitric oxide
orf19.1313	CDR3	13.96	11.81	17.14	14.38	0.101798027	NA	Transporter of the Pdr/Cdr family of the ATP-binding cassette superfamily; transports phospholipids out-to-in; expressed in opaque-phase cells; induced by macrophage interaction; fluconazole-repressed; Spider biofilm induced
orf19.6966	orf19.6966	15.08	11.8	15.93	13.15	0.037853825	NA	Ortholog(s) have choline kinase activity, ethanolamine kinase activity, role in phosphatidylcholine biosynthetic process, phosphatidylethanolamine biosynthetic process and cytosol, nucleus localization
orf19.3216	orf19.3216	10.6	11.78	11.97	10.79	-0.006055556	NA	Similar to Rab GTPase activators; Hap43p-induced gene
orf19.6316	orf19.6316	14.2	11.77	12.98	13.01	-0.004526865	NA	Predicted membrane transporter, member of the L-amino acid transporter-3 (LAT3) family, major facilitator superfamily (MFS)
orf19.6476	orf19.6476	11.29	11.75	10.72	10.49	-0.053960321	NA	Putative protein with a predicted role in exocytic transport from the Golgi; filament induced
orf19.444	orf19.444	11.76	11.75	8.12	13.98	-0.048626767	NA	Has domain(s) with predicted nucleic acid binding, nucleotide binding activity
orf19.1926	SEF2	13.71	11.74	10.04	8.09	-0.197603704	NA	Zn(II)2Cys6 transcription factor, required for normal resistance to copper; repressed by Sfu1 in high-iron conditions; regulated by Sef1, Sfu1, and Hap43; rat catheter and Spider biofilm induced

orf19.6804	orf19.6804	12.94	11.71	14.12	13.1	0.039507092	NA	Ortholog(s) have signal sequence binding activity, role in protein targeting to ER and cytosol, nuclear periphery, signal recognition particle, endoplasmic reticulum targeting localization
orf19.2761	orf19.2761	13.6	11.69	17.61	15.93	0.163200055	NA	Putative glycosylphosphatidylinositol (GPI) anchor assembly protein; transposon insertion causes decreased colony wrinkling but does not block true hyphal growth; induced by nitric oxide independent of Yhb1p
orf19.2778	orf19.2778	12.85	11.68	9.01	9.26	-0.174644837	NA	Protein of unknown function; transcript is upregulated in clinical isolates from HIV+ patients with oral candidiasis
orf19.5070	orf19.5070	9.96	11.68	5.61	6.33	-0.270135293	NA	Similar to cell-wall mannoproteins; induced in low iron; induced in cyr1 homozygous null; regulated by osmotic and oxidative stress via Hog1; Spider biofilm induced
orf19.5245	BUL4	10.48	11.67	9.27	7.82	-0.131633447	NA	Protein with a predicted BUL1 N-terminal and C-terminal domains; Bul1 binds the ubiquitin ligase Rsp5 in S. cerevisiae; Hap43-repressed gene; rat catheter biofilm induced
orf19.4284	BUR2	12.83	11.65	16.2	11.5	0.037572633	NA	Protein with similarity to S. cerevisiae Bur2p, contains a cyclin domain; not required for wild-type hyphal growth, adherence to buccal epithelial cells, or virulence in mouse systemic infection
orf19.4776	LYS143	11.91	11.63	10.2	9.22	-0.126776264	NA	Zn(II)2Cys6 transcription factor; ortholog of S. cerevisiae Lys14 involved in the regulation of lysine biosynthesis genes
orf19.3782	orf19.3782	12.08	11.62	15.83	14.27	0.126321887	NA	Predicted membrane transporter; member of the peptide-acetyl-coA transporter (PAT) family, major facilitator superfamily (MFS); induced by alpha pheromone in Spider medium
orf19.4673	BMT9	9.34	11.61	5.56	7.9	-0.1862863	NA	Beta-mannosyltransferase, 9-gene family that includes characterized genes BMT1, BMT2, BMT3, and BMT4 with roles in beta-1,2-mannosylation of cell wall phosphopeptidomannan; regulated by Sef1, Sfu1, Hap43; rat catheter biofilm repressed

orf19.4649	ZCF27	11	11.55	15.71	12.03	0.105266127	NA	Putative Zn(II)2Cys6 transcription factor
orf19.3965	orf19.3965	10.64	11.52	13.27	12.38	0.040225961	NA	Ortholog(s) have role in double-strand break repair via homologous recombination, meiotic gene conversion, reciprocal meiotic recombination, replication fork protection and Smc5-Smc6 complex, cytosol, nucleus localization
orf19.2174	RAD57	11.39	11.51	8.94	7.97	-0.155317273	NA	Putative DNA recombination and repair protein; induced by interaction with macrophage; transcript is regulated by Nrg1, Mig1, and Tup1; essential protein; S. cerevisiae ortholog is essential
orf19.4741	orf19.4741	12.83	11.5	11.91	11.91	-0.004277406	NA	Protein of unknown function; C. albicans- and C. dubliniensis specific gene; rat catheter biofilm repressed
orf19.2334	BIG1	12.67	11.49	13.02	12.04	0.018235754	NA	Endoplasmic reticulum (ER) protein; ortholog of S. cerevisiae Big1; required for beta-1,6-glucan synthesis, filamentation, adhesion, and virulence; rat catheter biofilm repressed
orf19.5840	orf19.5840	15.45	11.48	17.78	15.4	0.143120956	NA	Ortholog(s) have cytoplasm, mating projection tip localization
orf19.4761	HST1	16.14	11.44	12.54	12.34	-0.048194931	NA	Putative histone deacetylase, involved in regulation of white-opaque switching
orf19.3995	RIM13	13.48	11.41	16.69	10.7	0.082044195	NA	Protease of the pH response pathway; likely to mediate activation of Rim101 via C-terminal cleavage; required for alkaline pH-induced hyphal growth and for normal chlamydospore formation; Hap43-repressed; flow model biofilm induced
orf19.4270	MNN13	14.87	11.41	12.83	10.42	-0.073375016	NA	Predicted alpha-1,3-mannosyltransferase activity with a role in protein glycosylation; regulated by Sef1p-, Sfu1p-, and Hap43p

orf19.1296	orf19.1296	14.44	11.4	10.07	11.15	-0.097466508	NA	Ortholog(s) have role in spliceosomal snRNP assembly and U4/U6 x U5 tri-snRNP complex, mitochondrion localization
orf19.3901	orf19.3901	12.39	11.4	16.64	15.04	0.191951895	NA	Putative benzene desulfurase; induced by nitric oxide independent of Yhb1p
orf19.7319	SUC1	16.45	11.37	13.69	11.71	-0.026151146	NA	Zinc-finger transcription factor; regulates alpha-glucosidase exression; complements S. cerevisiae suc2 for sucrose utilization and mal13 maltase defect; required for yeast cell adherence to silicone substrate; rat catheter biofilm induced
orf19.478	orf19.478	14.88	11.37	13.42	9.6	-0.073375016	NA	Protein required for fusion of cvt-vesicles and autophagosomes with the vacuole; plays a role in autophagy, protein targeting to vacuole and vesicle docking; flow model biofilm induced
orf19.2087	SAS2	11.91	11.37	9.75	7.09	-0.154863349	NA	Predicted histone acetyltransferase involved in histone H4 acetylation; member of MYST family
orf19.2805	PEX8	15.08	11.36	16.89	12.81	0.081040065	NA	Putative peroxisomal biogenesis factor; expression regulated during planktonic growth
orf19.4498	orf19.4498	17.04	11.36	9.98	9.17	-0.212547329	NA	Ortholog of C. dubliniensis CD36 : Cd36_19120, C. parapsilosis CDC317 : CPAR2_103880, Candida tenuis NRRL Y-1498 : CANTEDRAFT_115581 and Debaryomyces hansenii CBS767 : DEHA2F08426g
orf19.1133	MSB1	12.06	11.35	14.45	11.41	0.040291532	NA	Putative regulator of transcription; expression in S. cerevisiae flo8 and flo11 mutants suggests a role in regulation of adhesion factors
orf19.114	orf19.114	14.16	11.32	13.93	10.92	-0.005208474	NA	Ortholog of C. dubliniensis CD36 : Cd36_61190, C. parapsilosis CDC317 : CPAR2_603230, Candida tenuis NRRL Y-1498 : cten_CGOB_00054 and Debaryomyces hansenii CBS767 : DEHA2E21142g

orf19.3743	orf19.3743	14.41	11.27	21.37	18.05	0.28182636	NA	Ortholog of C. dubliniensis CD36 : Cd36_27230, C. parapsilosis CDC317 : CPAR2_801320, Candida tenuis NRRL Y-1498 : CANTEDRAFT_113198 and Debaryomyces hansenii CBS767 : DEHA2E23782g
orf19.1392	orf19.1392	12.85	11.26	18.81	15.64	0.228370491	NA	Ortholog(s) have chaperone binding, protein disulfide isomerase activity, unfolded protein binding activity and role in ER-associated ubiquitin-dependent protein catabolic process, protein retention in ER lumen
orf19.3937	orf19.3937	14.28	11.26	15.19	12.18	0.039458815	NA	Ortholog(s) have protein phosphatase type 2A regulator activity, role in TOR signaling cascade, cellular response to nitrogen starvation and cytosol, nucleus localization
orf19.2312	orf19.2312	8.44	11.25	11.49	10.49	0.042718767	NA	Protein similar to ferric reductase Fre10p; possibly an essential gene, disruptants not obtained by UAU1 method
orf19.6329	orf19.6329	12.64	11.25	12.78	17.26	0.128811163	NA	Protein of unknown function; opaque-specific transcript; fluconazole-repressed; induced in cyr1 mutant and in oralpharyngeal candidasis; Spider biofilm induced
orf19.4349	orf19.4349	9.89	11.24	13.62	10.61	0.087299043	NA	Protein of unknown function; transcript repressed by elevated CO2; Spider biofilm induced
orf19.7163	orf19.7163	14.17	11.24	15.11	9.74	-0.006043562	NA	Ortholog of C. dubliniensis CD36 : Cd36_73790, C. parapsilosis CDC317 : CPAR2_702880, Candida tenuis NRRL Y-1498 : CANTEDRAFT_92070 and Debaryomyces hansenii CBS767 : DEHA2D10318g
orf19.2740	orf19.2740	13.31	11.24	11.61	10.03	-0.05222038	NA	Ortholog(s) have 5'-flap endonuclease activity
orf19.7365	orf19.7365	12.21	11.23	11.85	10.88	-0.005084712	NA	Ortholog(s) have ubiquitin-protein ligase activity and role in negative regulation of apoptotic process, negative regulation of gluconeogenesis, proteasome-mediated ubiquitin-dependent protein catabolic process

orf19.1513	FAB1	13.31	11.22	13.55	12.9	0.063031715	NA	Phosphatidylinositol 3-phosphate 5-kinase; required for hyphal growth on solid media, and for wild-type vacuolar morphology and acidification; not required for wild-type virulence in mouse systemic infection or for adherence to HeLa cells
orf19.3459	orf19.3459	11.51	11.2	21.42	17.28	0.308944665	NA	Putative serine/threonine/tyrosine (dual-specificity) kinase; disruptants not obtained by UAU1 method
orf19.1297	orf19.1297	18.01	11.19	17.71	13.09	0.036807084	NA	Ortholog(s) have phosphatidylinositol-3-phosphate binding activity and role in CVT pathway, early endosome to Golgi transport, macroautophagy, mitochondrion degradation
orf19.1617	orf19.1617	8.95	11.19	10.21	11.16	0.019202472	NA	Protein similar to S. cerevisiae Ydr282cp; transposon mutation affects filamentous growth; Hap43p-repressed gene
orf19.5934	orf19.5934	14.81	11.17	14.38	14.08	0.04032886	NA	Ortholog(s) have DNA topoisomerase type I activity
orf19.1590	orf19.1590	9.79	11.14	8.31	9.24	-0.105952754	NA	Ortholog of C. dubliniensis CD36 : Cd36_18970, C. parapsilosis CDC317 : CPAR2_212800, Debaryomyces hansenii CBS767 : DEHA2F08690g and Candida guilliermondii ATCC 6260 : PGUG_01488
orf19.4068	orf19.4068	12.99	11.13	14.57	13.84	0.106447205	NA	Protein of unknown function; flow model biofilm induced
orf19.1087	orf19.1087	14.1	11.13	9.96	9.42	-0.148199742	NA	Ortholog of C. parapsilosis CDC317 : CPAR2_601130, C. dubliniensis CD36 : Cd36_64620, Lodderomyces elongisporus NRLL YB-4239 : LELG_04885 and Candida orthopsilosis Co 90-125 : CORT_0F02130
orf19.5935	orf19.5935	13.96	11.12	14.33	12.4	0.017751895	NA	Ortholog of C. dubliniensis CD36 : Cd36_84710, C. parapsilosis CDC317 : CPAR2_807470, Candida tenuis NRRL Y-1498 : CANTEDRAFT_136475 and Debaryomyces hansenii CBS767 : DEHA2B08844g

orf19.7223	orf19.7223	15.76	11.1	13.92	11.7	-0.026151146	NA	Ortholog(s) have protein-lysine N-methyltransferase activity, role in peptidyl-lysine monomethylation and cytosol, nucleus localization
orf19.7321	orf19.7321	13.85	11.1	18.59	15.84	0.205381487	NA	Ortholog(s) have role in re-entry into mitotic cell cycle after pheromone arrest and endoplasmic reticulum localization
orf19.1795.1	orf19.1795.1	2.56	11.09	6.81	11.91	0.117384868	NA	Ortholog(s) have role in protein targeting to ER, signal peptide processing and signal peptidase complex localization
orf19.5254	orf19.5254	17.39	11.06	9.99	13.13	-0.112967934	NA	Protein of unknown function; repressed by nitric oxide
orf19.4355	orf19.4355	12.58	11.05	8.36	9.12	-0.176296514	NA	Ortholog of C. dubliniensis CD36 : Cd36_29080, C. parapsilosis CDC317 : CPAR2_202490, Candida tenuis NRRL Y-1498 : CANTEDRAFT_112323 and Debaryomyces hansenii CBS767 : DEHA2F19184g
orf19.928	orf19.928	15.92	11.04	17.76	13.69	0.100304874	NA	Ortholog of C. dubliniensis CD36 : Cd36_50610, C. parapsilosis CDC317 : CPAR2_303690, Candida tenuis NRRL Y-1498 : CANTEDRAFT_93989 and Debaryomyces hansenii CBS767 : DEHA2E15400g
orf19.1409	VAC7	11.68	11.04	13.3	11.41	0.017821435	NA	Ortholog of S. cerevisiae Vac7; integral vacuolar membrane protein involved in vacuole inheritance and morphology; mutants are viable
orf19.4275	RAD9	13.24	11.04	11.48	11.27	-0.051358235	NA	DNA damage-dependent checkpoint protein; involved in regulation of DNA-damage-induced filamentous growth; induced by alpha pheromone in SpiderM medium
orf19.4222	SST2	12.32	11.04	8.19	6.73	-0.208421378	NA	Predicted regulator of G-protein signaling in mating pathway; null mutation causes alpha-factor hypersensitivity and mating defect (in opaque MTLa/MTLa background); transcript induced by alpha factor

orf19.101	RIM9	11.22	11.03	14.07	11.9	0.086761677	NA	Protein required for alkaline pH response via the Rim101 signaling pathway; ortholog of S. cerevisiae Rim9 and A. nidulans palI; Spider biofilm induced
orf19.4784	CRP1	11.6	11.03	7.24	8.3	-0.207167236	NA	Copper transporter; CPx P1-type ATPase; mediates Cu resistance; similar to Menkes and Wilson disease proteins; copper-induced; Tbf1-activated; suppresses Cu sensitivity of S. cerevisiae cup1 mutant; flow model biofilm induced
orf19.5532	orf19.5532	7.16	11.01	2.75	1.65	-0.393034509	NA	Protein of unknown function; Spider biofilm induced
orf19.3647	SEC8	12.5	11	20.57	16.43	0.265695821	NA	Predicted subunit of the exocyst complex, involved in exocytosis; localizes to a crescent on the surface of the hyphal tip
orf19.5798	LIG4	8.86	10.97	11.93	9.55	0.041915017	NA	DNA ligase; mRNA detected in yeast-form and pseudohyphal cells, induced upon hyphal induction; suppresses S. cerevisiae ime1-1 mutant IME2 transcription defect; required for wild-type filamentous growth and wild-type pathogenesis
orf19.1763	IFR1	12.66	10.97	25.25	24.57	0.499042158	NA	Predicted oxidoreductase/dehydrogenase; induced by nitric oxide; rat catheter and Spider biofilm induced
orf19.2637	orf19.2637	10.97	10.96	12.05	12.79	0.065439356	NA	Ortholog(s) have Golgi apparatus, fungal-type vacuole membrane localization
orf19.4766	ARG81	11.02	10.96	8.6	7.77	-0.131013213	NA	Zn(II)2Cys6 transcription factor; required for utilization of ornithine as a nitrogen source and for wild-type resistance to caffeine; required for yeast cell adherence to silicone substrate
orf19.3784	orf19.3784	8.81	10.96	14.57	13.5	0.178524117	NA	Ortholog of C. dubliniensis CD36 : Cd36_44580

orf19.4518	orf19.4518	11.92	10.96	10.12	12.32	-0.027408259	NA	Protein kinase of unknown function; mutants are viable
orf19.7218	RBE1	14.45	10.95	10.46	9.76	-0.122636226	NA	Pry family cell wall protein; Rim101, Efg1, Ssn6, alkaline repressed; O-glycosylation; no GPI anchor predicted; ketoconazol induced; regulated by Sef1, Sfu1, Hap4; flow model biofilm induced; rat catheter and Spider biofilm repressed
orf19.5970	orf19.5970	14.17	10.89	13.35	12.07	-0.004356241	NA	Putative DNA repair helicase; transcriptionally induced by interaction with macrophage; fungal-specific (no human or murine homolog)
orf19.5780	orf19.5780	14.56	10.89	13.88	14.42	0.04032886	NA	Putative protein of unknown function; Hap43p-repressed gene
orf19.3677	orf19.3677	13.85	10.89	9.51	10.18	-0.122636226	NA	Ortholog(s) have Atg8 ligase activity and role in C-terminal protein lipidation, CVT pathway, ascospore formation, cellular response to nitrogen starvation, macroautophagy, mitochondrion degradation, piecemeal microautophagy of nucleus
orf19.3318	orf19.3318	14.3	10.88	17.26	14.74	0.145883936	NA	Ortholog(s) have cytoplasm localization
orf19.5897	orf19.5897	13.28	10.87	19.74	18.93	0.306010036	NA	Ortholog(s) have Seh1-associated complex, cytosol, extrinsic to fungal-type vacuolar membrane localization
orf19.2756	orf19.2756	14.47	10.85	18.64	11.72	0.121969694	NA	Ortholog(s) have HDEL sequence binding activity, role in ER to Golgi vesicle-mediated transport, protein retention in ER lumen and Golgi apparatus, integral to endoplasmic reticulum membrane localization
orf19.6194	orf19.6194	13.65	10.83	14.81	14.8	0.105273763	NA	Ortholog of C. dubliniensis CD36 : Cd36_06640, C. parapsilosis CDC317 : CPAR2_208220, Candida tenuis NRRL Y-1498 : CANTEDRAFT_136902 and Debaryomyces hansenii CBS767 : DEHA2F26730g

orf19.4549	FGR38	12.38	10.82	17.61	16.91	0.252710992	NA	Protein lacking an ortholog in S. cerevisiae; member of a family of related proteins; transposon mutation affects filamentous growth; macrophage-induced gene; Hap43p-repressed gene
orf19.10	ALK8	12.26	10.82	4.32	4.08	-0.419007171	NA	Alkane-inducible cytochrome P450; catalyzes hydroxylation of lauric acid to hydroxylauric acid; overproduction causes fluconazole resistance in WT and causes multidrug resistance in a cdr1 cdr2 double mutant; rat catheter biofilm repressed
orf19.342	BMT7	9.85	10.79	10.39	7.17	-0.10761169	NA	Beta-mannosyltransferase, member of a 9-gene family that includes characterized genes BMT1, BMT2, BMT3, and BMT4 with roles in beta-1,2-mannosylation of cell wall phosphopeptidomannan; downregulated in azole-resistant strain; Hap43p-induced
orf19.5249	orf19.5249	13.96	10.78	16.77	16.76	0.186972053	NA	Ortholog(s) have role in ascospore formation, meiosis and cytosol, nucleus localization
orf19.5428	orf19.5428	16.38	10.76	16.8	14.23	0.080396649	NA	Putative Golgi membrane protein with a predicted role in manganese homeostasis; Hap43p-repressed gene; clade-associated gene expression
orf19.3429	FGR47	13.84	10.76	13.56	11.98	0.017751895	NA	Protein lacking an ortholog in S. cerevisiae; transposon mutation affects filamentous growth
orf19.3203	RCY1	11.76	10.75	11.08	9.71	-0.053038603	NA	Putative F-box protein involved in endocytic membrane traffic and/or recycling; fungal-specific (no human or murine homolog)
orf19.2552	orf19.2552	7.92	10.74	9.78	10.8	0.043596971	NA	Protein similar to S. cerevisiae Pmr1p; amphotericin B induced; previously merged with orf19.2553; unmerged from orf19.2553 in a revision of Assembly 21
orf19.2542	orf19.2542	10.48	10.74	9.95	8.67	-0.055484949	NA	Ortholog(s) have nucleus localization

orf19.3062	orf19.3062	11.88	10.74	12.26	10.09	-0.029277054	NA	Ortholog(s) have role in chromatin remodeling and ASTRA complex, mitochondrion localization
orf19.2921	orf19.2921	9.87	10.73	11.45	8.86	-0.030874128	NA	Ortholog(s) have alpha-tubulin binding activity, role in protein folding, tubulin complex assembly and cytosol, nucleus localization
orf19.5595	SHE3	10.98	10.73	18.68	13.44	0.212325142	NA	mRNA-binding protein that localizes specific mRNAs to daughter yeast cells and to hyphal tips; required for normal filamentation and host epithelial cell damage; ortholog of S. cerevisiae She3 but target mRNAs differs
orf19.7279	NIT2	17.04	10.73	21.62	16.78	0.213069144	NA	Putative carbon-nitrogen hydrolase; rat catheter biofilm repressed
orf19.3239	CTF18	11.37	10.72	11.92	11.05	0.018300265	NA	Protein with a predicted role in sister chromatid cohesion; cell-cycle regulated periodic mRNA expression
orf19.764	orf19.764	10.33	10.67	15.14	12.58	0.154623631	NA	Ortholog(s) have clathrin-coated vesicle, cytoplasm localization
orf19.2914	orf19.2914	13.06	10.64	13.59	10.47	-0.006008138	NA	Ortholog of C. dubliniensis CD36 : Cd36_45760, C. parapsilosis CDC317 : CPAR2_401920, Candida tenuis NRRL Y-1498 : CANTEDRAFT_126814 and Debaryomyces hansenii CBS767 : DEHA2D16742g
orf19.1492	PRP39	11.19	10.63	8.77	10.41	-0.078316322	NA	Putative component of the U1 snRNP; involved in splicing; Hap43-induced gene; Spider biofilm induced
orf19.2674	orf19.2674	10.62	10.62	7.12	4.93	-0.270263406	NA	Putative transcription factor with zinc finger DNA-binding motif; Hap43p-repressed gene

orf19.257	orf19.257	4.37	10.62	8.1	11.21	0.093898607	NA	Transcription is negatively regulated by Sfu1p; repressed by nitric oxide
orf19.2835	orf19.2835	12.86	10.61	13.63	11.7	0.040362167	NA	Ortholog(s) have SUMO activating enzyme activity, role in cellular response to DNA damage stimulus, protein sumoylation, regulation of mitotic cell cycle and SUMO activating enzyme complex, cytosol localization
orf19.4444	PHO15	16.73	10.61	25.23	26.85	0.437697066	NA	4-nitrophenyl phosphatase, possible histone H2A phosphatase; involved in regulation of white-opaque switch; hyphal repressed; induced in core stress response; induced by cadmium stress via Hog1; rat catheter and Spider biofilm repressed
orf19.6499	orf19.6499	11.2	10.59	9.31	10.71	-0.052694612	NA	Predicted DNA-directed RNA polymerase; role in transcription; Spider biofilm induced
orf19.7400	ALS7	11.71	10.59	11.1	10.22	-0.053038603	NA	ALS family protein; hypermutable contingency gene; growth-regulated, downregulated in biofilm; two variable repeat regions; expression in S. cerevisiae does not confer adhesiveness; ALS family includes adhesins, cell-surface glycoproteins
orf19.5625	orf19.5625	17.16	10.58	14.56	5.09	-0.182778127	NA	Ortholog of C. dubliniensis CD36 : Cd36_63640, Pichia stipitis Pignal : PICST_32878, Candida guilliermondii ATCC 6260 : PGUG_03423 and Candida tropicalis MYA-3404 : CTRG_02106
orf19.1793	orf19.1793	16.91	10.58	18.52	13.55	0.098213175	NA	Ortholog(s) have phosphatidylinositol binding activity, role in cellular response to nitrogen starvation, piecemeal microautophagy of nucleus and Golgi apparatus, endosome, extrinsic to membrane, fungal-type vacuole localization
orf19.3624	MSU1	15.11	10.57	11.34	10.75	-0.095906218	NA	Ortholog(s) have exoribonuclease II activity, role in mitochondrial RNA catabolic process and mitochondrial degradosome localization
orf19.5559	RAV2	14.27	10.57	15.11	15.1	0.105273763	NA	Protein similar to S. cerevisiae Rav2; a regulator of (H+)-ATPase in vacuolar membrane; transposon mutation affects filamentous growth

orf19.3574	MDJ2	10.56	10.56	10.99	10.98	-0.005007389	NA	Predicted component of the mitochondrial import motor; involved in protein import into mitochondrial matrix; early-stage flow model biofilm induced
orf19.3152	AMO2	12.67	10.56	17.12	17.3	0.209936139	NA	Protein similar to A. niger predicted peroxisomal copper amino oxidase; mutation confers hypersensitivity to toxic ergosterol analog; F-12/CO2 early biofilm induced
orf19.4404	PGA49	15.76	10.56	15.63	13.12	0.038595925	NA	Putative GPI-anchored protein
orf19.323	orf19.323	13.46	10.56	13.67	11.44	0.017333416	NA	Putative haloacid dehalogenase; localized to plasma membrane
orf19.1850	SIW14	13.69	10.55	11.64	8.28	-0.123736547	NA	Putative protein phosphatase of the PTP family (tyrosine-specific), similar to S. cerevisiae Siw14p; mutant shows virulence defect; Hap43p-repressed gene
orf19.1535	orf19.1535	9.34	10.54	7.88	10.03	-0.055049076	NA	Protein of unknown function; Hap43-induced gene
orf19.3214	orf19.3214	12.42	10.54	11.39	9.38	-0.078061347	NA	Alpha/beta-HydrolasE superfamily protein; membrane-localized
orf19.1070	orf19.1070	9.54	10.53	18.23	13.67	0.237791688	NA	Ortholog(s) have cytoplasm localization
orf19.351	orf19.351	13.47	10.52	18.89	16.36	0.228370491	NA	Ortholog of S. cerevisiae : YPR153W, C. glabrata CBS138 : CAGL0L08382g, C. dubliniensis CD36 : Cd36_83540, C. parapsilosis CDC317 : CPAR2_404520 and Candida tenuis NRRL Y-1498 : CANTEDRAFT_115795

orf19.1318	orf19.1318	16.64	10.51	9.96	8.9	-0.212547329	NA	Ortholog of C. dubliniensis CD36 : Cd36_43350, C. parapsilosis CDC317 : CPAR2_403560, Debaryomyces hansenii CBS767 : DEHA2A08250g and Pichia stipitis Pignal : PICST_29838
orf19.6021	IHD2	13.04	10.48	14.54	14.78	0.151816352	NA	Protein of unknown function; induced during hyphae development; induced in low iron; Spider biofilm induced
orf19.3687	orf19.3687	6.06	10.46	18.68	4.94	0.169890568	NA	Ortholog(s) have unfolded protein binding activity, role in cytoskeleton organization, protein folding and prefoldin complex localization
orf19.3263	ATO10	11.34	10.46	23.91	11.73	0.307510136	NA	Putative fungal-specific transmembrane protein; rat catheter and Spider biofilm induced
orf19.5976	orf19.5976	12.82	10.44	11.67	9.72	-0.028230401	NA	Protein of unknown function; ortholog of S. cerevisiae Urn1; downregulated by fluphenazine treatment, in azole-resistant strains that overexpress CDR1 and CDR2 or MDR1
orf19.3394	orf19.3394	13.77	10.4	18.93	12.51	0.166295009	NA	Predicted membrane protein; induced by alpha pheromone in SpiderM medium
orf19.1156	FUS1	11.05	10.39	16.85	13.6	0.219107711	NA	Membrane protein required for mating; ortholog of S. cerevisiae Fus1; transcript induced by Cph1 in cells homozygous for the MTLa locus; alpha factor induced
orf19.4715	orf19.4715	8.59	10.39	13.5	15.22	0.205986569	NA	Ortholog(s) have tubulin binding activity, role in microtubule cytoskeleton organization, mitochondrial fission, mitochondrion inheritance, nuclear migration along microtubule and cell cortex, cellular bud tip, mitochondrion localization
orf19.1275	GAT1	13.79	10.38	7.59	7.05	-0.229567787	NA	GATA-type transcription factor; regulator of nitrogen utilization; required for nitrogen catabolite repression and utilization of isoleucine, tyrosine and tryptophan N sources; required for virulence in a mouse systemic infection model

orf19.2749	orf19.2749	10.75	10.37	6.06	6.31	-0.244772179	NA	BTB/POZ domain protein; induced by Mnl1 under weak acid stress; flow model biofilm induced; Spider biofilm induced
orf19.4713	orf19.4713	12.31	10.37	15.98	11.41	0.108004572	NA	Ortholog of C. dubliniensis CD36 : Cd36_07300, C. parapsilosis CDC317 : CPAR2_208300, Candida tenuis NRRL Y-1498 : CANTEDRAFT_95879 and Debaryomyces hansenii CBS767 : DEHA2F26246g
orf19.5362	PSO2	11.87	10.37	8.62	8.96	-0.103367914	NA	Putative DNA cross-link repair protein; expressed in opaque or white MTLa/MTLa or MTLalpha/MTLalpha, but not MTLa/MTLalpha cells; telomere-proximal gene; mutation does not affect white-to-opaque phenotypic switching
orf19.4280	orf19.4280	10.64	10.34	8.84	10.61	-0.02796186	NA	Protein of unknown function; Spider biofilm induced; rat catheter biofilm repressed
orf19.4007	orf19.4007	9.91	10.34	8.59	12.31	0.021115039	NA	Ortholog(s) have protein-lysine N-methyltransferase activity, role in peptidyl-lysine monomethylation, peptidyl-lysine trimethylation and cytosol, nucleolus localization
orf19.6269	orf19.6269	11.34	10.33	12.51	8.1	-0.006635796	NA	Ortholog of C. dubliniensis CD36 : Cd36_05990, Candida tenuis NRRL Y-1498 : CANTEDRAFT_91852, Debaryomyces hansenii CBS767 : DEHA2G15356g and Pichia stipitis Pignal : PICST_87044
orf19.647.3	orf19.647.3	9.64	10.33	9.62	4.81	-0.135497067	NA	Protein of unknown function; Spider biofilm induced
orf19.130	VPS15	12.95	10.32	13.51	9.58	0.017803523	NA	Protein involved in retrograde endosome-to-Golgi protein transport; required for normal virulence
orf19.1648	RAD50	9.68	10.32	16.15	14.72	0.244642266	NA	Putative DNA double-strand break repair factor; involved in response to oxidative stress and drug resistance; flow model biofilm repressed

orf19.4097	orf19.4097	14.4	10.31	12.07	12.21	-0.003297411	NA	Ortholog of C. dubliniensis CD36 : Cd36_23480, C. parapsilosis CDC317 : CPAR2_407050, Candida tenuis NRRL Y-1498 : CANTEDRAFT_116822 and Debaryomyces hansenii CBS767 : DEHA2B02046g
orf19.2844	APL4	12.02	10.31	18.94	16.27	0.276034805	NA	Predicted gamma-adaptin, large subunit of the clathrin-associated protein (AP-1) complex; that binds clathrin and is involved in vesicle mediated transport; induced in core caspofungin response; rat catheter biofilm repressed
orf19.1632	orf19.1632	10.77	10.3	16.87	11.5	0.152752037	NA	Ortholog(s) have cytosol, nucleus localization
orf19.389	orf19.389	10.28	10.28	16.19	18.88	0.326299896	NA	Protein of unknown function; Hap43-induced gene
orf19.2216	PDS5	11.21	10.27	13.71	12.02	0.111590238	NA	Putative protein with a predicted role in establishment and maintenance of sister chromatid condensation and cohesion; cell-cycle regulated periodic mRNA expression
orf19.7209	orf19.7209	7.82	10.26	6.34	4.38	-0.229751763	NA	Putative Rho GDP dissociation inhibitor; induced by nitric oxide independent of Yhb1p
orf19.6168	orf19.6168	14.38	10.25	14.96	13.37	0.085081358	NA	Ortholog of C. dubliniensis CD36 : Cd36_80830, Debaryomyces hansenii CBS767 : DEHA2F17930g, Candida tropicalis MYA-3404 : CTRG_04030 and Candida lusitaniae ATCC 42720 : CLUG_04224
orf19.6972	SMI1B	10.79	10.23	13.46	10.09	0.042105307	NA	Putative cell wall assembly regulatory protein; Rim101-repressed; possibly an essential gene, disruptants not obtained by UAU1 method
orf19.3447	orf19.3447	15.99	10.18	14.4	9.38	-0.072422885	NA	Ortholog(s) have mannosyltransferase activity, role in ER-associated ubiquitin-dependent protein catabolic process, GPI anchor biosynthetic process, protein processing and endoplasmic reticulum localization

orf19.5253	orf19.5253	10.84	10.17	10.39	8.8	-0.054532264	NA	Has domain(s) with predicted ATP binding, protein tyrosine kinase activity and role in protein phosphorylation
orf19.7376	orf19.7376	10.57	10.16	8.05	6.76	-0.159923313	NA	Ortholog of C. dubliniensis CD36 : Cd36_85880, C. parapsilosis CDC317 : CPAR2_806870, Candida tenuis NRRL Y-1498 : CANTEDRAFT_96983 and Debaryomyces hansenii CBS767 : DEHA2D16148g
orf19.3942	orf19.3942	14.74	10.15	9.47	9.14	-0.171458707	NA	Ortholog(s) have structural molecule activity
orf19.1622	YCG1	10.34	10.15	10.07	8.42	-0.056250852	NA	Putative condensin G
orf19.4589	orf19.4589	10.66	10.14	17.9	13.48	0.217477689	NA	Ortholog(s) have polyamine oxidase activity, role in pantothenate biosynthetic process, polyamine catabolic process and cytoplasm localization
orf19.5557	MNN4-4	9.71	10.13	13.13	10.73	0.090217185	NA	Mannosyltransferase; transcript upregulated in Ssk1 response regulator mutant or in nik1 null mutant, but not in chk1 or sln1 null mutants; pheromone induced; Spider biofilm induced
orf19.6238	orf19.6238	11.84	10.12	18.02	12.42	0.171654118	NA	Ortholog(s) have mitochondrion localization
orf19.6342	orf19.6342	14.54	10.1	8.06	12.48	-0.117909812	NA	Ortholog of C. dubliniensis CD36 : Cd36_11980, C. parapsilosis CDC317 : CPAR2_201330, Candida tenuis NRRL Y-1498 : CANTEDRAFT_114815 and Debaryomyces hansenii CBS767 : DEHA2G10032g
orf19.4511	orf19.4511	13	10.1	13.69	11.91	0.064201336	NA	Ortholog of Candida albicans WO-1 : CAWG_04197

orf19.2303	FGR16	9.83	10.1	11.98	10.88	0.067345771	NA	Protein lacking an ortholog in S. cerevisiae; transposon mutation affects filamentous growth; fluconazole-downregulated
orf19.2532	PRS	12.14	10.1	17.9	17.89	0.297005652	NA	Putative prolyl-tRNA synthetase; monofunctional Class II synthetase; gene is constitutively expressed
orf19.1596	FGR28	12.97	10.09	12.32	11.28	-0.004096034	NA	Protein lacking an ortholog in S. cerevisiae; transposon mutation affects filamentous growth; possibly an essential gene, disruptants not obtained by UAU1 method
orf19.2684	orf19.2684	11.97	10.07	14.88	13.82	0.153882892	NA	Protein of unknown function; flow model biofilm repressed
orf19.3295	orf19.3295	11.13	10.07	11.75	6.65	-0.055819513	NA	Has domain(s) with predicted role in cell division, chromosome segregation and MIS12/MIND type complex localization
orf19.5841	orf19.5841	9.05	10.05	14.29	11.28	0.137140484	NA	Ortholog of C. dubliniensis CD36 : Cd36_17700, C. parapsilosis CDC317 : CPAR2_212340, Candida tenuis NRRL Y-1498 : CANTEDRAFT_101113 and Debaryomyces hansenii CBS767 : DEHA2G03784g
orf19.4655	OPT6	11.16	10.05	14.95	11.97	0.133268965	NA	Putative oligopeptide transporter; fungal-specific (no human or murine homolog); expression of OPT6, OPT7, or OPT8 does not suppress defect of mutant lacking Opt1p, Opt2p, and Opt3p; alleles are nonidentical
orf19.4192	CDC14	11.53	10.04	12.29	9.56	-0.004913336	NA	Protein involved in exit from mitosis and morphogenesis; ortholog of S. cerevisiae Cdc14p, which is a dual-specificity phosphatase and cell-cycle regulator; suppresses S. cerevisiae cdc15-lyt1, dbf2-2, and (partially) tem1 mutant phenotypes
orf19.2281	orf19.2281	9.05	10.03	14.34	14.21	0.205617918	NA	Ortholog(s) have mitochondrion localization

orf19.4592	HSX11	11.37	10	12.7	11.79	0.089464111	NA	UDP-glucose:ceramide glucosyltransferase (glucosylceramide synthase [GCS], EC 2.4.1.80); involved in glucosylceramide biosynthesis, which is important for virulence
orf19.6692	MNN26	15.62	10	13.95	12.08	-0.003462049	NA	Putative alpha-1,2-mannosyltransferase; mutant sensitive to calcofluor, SDS and growth at 42 deg; present in detergent-resistant membrane fraction (possible lipid raft component); predicted N-terminal acetylation and transmembrane helix
orf19.2620	orf19.2620	8.09	9.98	18.68	18.46	0.414616066	NA	Ortholog of C. dubliniensis CD36 : Cd36_33120 and Candida albicans WO-1 : CAWG_02046
orf19.309	DAL5	7.18	9.97	5.61	4.33	-0.203272584	NA	Allantoate permease; nitrogen catabolite repressed, induced in absence of preferred N sources; nitrogen source regulation requires Gat1; possibly essential gene (by UAU1 method); Hap43-repressed
orf19.4583	orf19.4583	19.32	9.96	8.92	9.53	-0.228663447	NA	Protein with a mitochondrial carrier protein domain; possibly an essential gene, disruptants not obtained by UAU1 method; Spider biofilm repressed
orf19.7232	IRR1	9.78	9.95	11.49	9.58	0.019388509	NA	Putative cohesin complex subunit; cell-cycle regulated periodic mRNA expression
orf19.6465	orf19.6465	7.97	9.95	9.32	11.52	0.070382713	NA	Ortholog of C. dubliniensis CD36 : Cd36_83910, Candida tropicalis MYA-3404 : CTRG_05087 and Candida albicans WO-1 : CAWG_00654
orf19.3238	orf19.3238	10.93	9.93	9.91	13.37	0.044657268	NA	Ortholog of Candida albicans WO-1 : CAWG_01469
orf19.247	orf19.247	13.32	9.92	10.42	8.85	-0.10154831	NA	Ortholog(s) have role in endosome organization, regulation of protein localization and BLOC-1 complex, endosome localization

orf19.1440.1	orf19.1440.1	4.41	9.91	10.99	6.59	0.093877834	NA	Protein of unknown function
orf19.736	SRB8	9.46	9.91	7.28	7.17	-0.137903319	NA	Putative RNA polymerase II mediator complex subunit; early-stage flow model biofilm induced
orf19.74	SEC5	11.06	9.9	12.18	10.13	0.018869492	NA	Predicted exocyst component; ortholog of S. cerevisiae Sec5p; merged with orf19.75 in Assembly 21
orf19.413	orf19.413	8.11	9.89	10.22	8.97	0.019423233	NA	Protein of unknown function; induced by Sfu1; Spider biofilm induced
orf19.6008.3	orf19.6008.3	13.75	9.88	17.15	10.71	0.082682577	NA	Ortholog of C. dubliniensis CD36 : Cd36_00920, Debaryomyces hansenii CBS767 : DEHA2F20724g, Pichia stipitis Pignal : PICST_32745 and Candida albicans WO-1 : CAWG_01277
orf19.4239	orf19.4239	10.84	9.87	11.09	10.42	-0.004840911	NA	Planktonic growth-induced gene
orf19.5188	CHS1	11.11	9.85	14.05	11.25	0.088404054	NA	Chitin synthase; essential; for primary septum synthesis in yeast and hyphae; 1 of several chitin synthases; enzymatically activated by proteolytic processing; complements defects of S. cerevisiae chs1 or chs2; Spider biofilm repressed
orf19.4253	orf19.4253	13.93	9.83	14.44	11.69	0.041166124	NA	Ortholog(s) have role in negative regulation of gluconeogenesis, proteasome-mediated ubiquitin-dependent protein catabolic process, regulation of nitrogen utilization, vacuolar protein catabolic process
orf19.5071	NRP1	5.96	9.83	10.37	9.88	0.095459594	NA	Ortholog(s) have cytoplasmic stress granule, nucleolus localization

orf19.5820	UGA6	7.48	9.83	7.37	9.68	-0.004424983	NA	Putative GABA-specific permease; decreased transcription is observed upon benomyl treatment or in an azole-resistant strain that overexpresses MDR1
orf19.6827	SLU7	19.13	9.83	14.26	12.12	-0.066656738	NA	Protein similar to S. cerevisiae RNA splicing factor, Slu7; induced by alpha pheromone in SpiderM medium
orf19.5854.1	orf19.5854.1	5.73	9.82	9.39	10.61	0.096100311	NA	Has domain(s) with predicted ATP binding, ATPase activity
orf19.751	orf19.751	9.01	9.82	13.88	12.79	0.183256056	NA	Ortholog of C. dubliniensis CD36 : Cd36_04660, Candida tropicalis MYA-3404 : CTRG_04484 and Candida albicans WO-1 : CAWG_00904
orf19.360	FUR4	12.41	9.82	19.36	16.36	0.276034805	NA	Putative uracil permease
orf19.1223	DBF2	12.57	9.79	12.02	11.23	-0.004096034	NA	Essential serine/threonine protein kinase involved in mitotic spindle formation and cytokinesis; required for septum formation, exit from mitosis, and normal hyphal morphogenesis; virulence-group-correlated expression
orf19.4334	PGA58	7.15	9.79	6.86	7.65	-0.058555716	NA	Putative GPI-anchored protein; transcription is positively regulated by Tbf1p
orf19.3988	orf19.3988	6.34	9.77	6.86	7.18	-0.06030444	NA	Putative adhesin-like protein; induced by Mnl1 under weak acid stress; rat catheter and Spider biofilm induced
orf19.7463	orf19.7463	10.49	9.75	10.61	12.38	0.068173956	NA	Ortholog(s) have role in ascospore wall assembly and ascospore wall, nuclear envelope localization

orf19.6879	orf19.6879	16.16	9.74	14.69	10.19	-0.026918211	NA	Ortholog(s) have tRNA-intron endonuclease activity, role in tRNA-type intron splice site recognition and cleavage and mitochondrial outer membrane, tRNA-intron endonuclease complex localization
orf19.3781	orf19.3781	10.92	9.72	10.76	10.62	0.019793526	NA	Protein of unknown function; Hap43-repressed; Spider biofilm induced
orf19.7545	orf19.7545	11.16	9.69	13.79	13.98	0.156697903	NA	Protein similarity to mutator-like element (MULE) transposase
orf19.3625	orf19.3625	12.37	9.68	12.87	11.26	0.042149411	NA	Has domain(s) with predicted zinc ion binding activity
orf19.3561	CDC7	11.27	9.67	11.81	12.64	0.09024542	NA	Putative kinase; cell-cycle regulated periodic mRNA expression; S. cerevisiae ortholog is not cell-cycle regulated; possibly an essential gene, disruptants not obtained by UAU1 method
orf19.769	IFE1	9.97	9.65	6.47	6.31	-0.220269025	NA	Putative medium-chain alcohol dehydrogenase; rat catheter and Spider biofilm repressed
orf19.3910	orf19.3910	8.29	9.64	13.98	10.37	0.138147121	NA	Has domain(s) with predicted RNA binding, ribonuclease T2 activity
orf19.5265	KIP4	10.99	9.63	10.49	9.13	-0.054532264	NA	S. cerevisiae Smy1 ortholog; Tn mutation affects filamentous growth; filament induced; has Mob2-dependent hyphal regulation; regulated by Nrg1, Tup1; oralpharyngeal candidasis induced; Spider biofilm induced; flow model biofilm repressed
orf19.2600	SPC98	10.96	9.63	13.59	10.22	0.06484209	NA	Putative component of the microtubule-nucleating Tub4p (gamma-tubulin) complex; periodic mRNA expression, peak at cell-cycle S/G2 phase

orf19.3658	orf19.3658	9.53	9.62	12.73	11.02	0.090217185	NA	Ortholog(s) have role in protein folding in endoplasmic reticulum and Golgi apparatus, integral to endoplasmic reticulum membrane, mitochondrion localization
orf19.4981	orf19.4981	9.62	9.62	10.73	8.37	-0.031144759	NA	Ortholog(s) have endoplasmic reticulum localization
orf19.5090	orf19.5090	14.24	9.62	7.48	8.05	-0.228289552	NA	Ortholog(s) have tRNA-specific adenosine deaminase activity, tRNA-specific adenosine-34 deaminase activity and role in adenosine to inosine editing, tRNA modification
orf19.5089	TERT	9.91	9.62	11.23	10.44	0.019388509	NA	Telomerase reverse transcriptase; catalytic protein subunit of telomere synthesis; essential for telomerase activity; has telomerase-specific motif T and other conserved reverse transcriptase motifs
orf19.4725	SWI6	12.66	9.62	18.35	14.64	0.212227389	NA	Putative component of the MBF and SBF transcription complexes involved in G1/S cell-cycle progression; periodic mRNA expression, peak at cell-cycle G1/S phase
orf19.1143	orf19.1143	13.79	9.61	14.77	10.3	0.017267967	NA	Ortholog of Pichia stipitis Pignal : PICST_30878, Spathaspora passalidarum NRRL Y-27907 : SPAPADRAFT_60017, Candida guilliermondii ATCC 6260 : PGUG_05223 and Candida tropicalis MYA-3404 : CTRG_01052
orf19.5235	orf19.5235	15.08	9.59	14.22	10.66	-0.00423643	NA	Putative mitochondrial ribosomal protein of the large subunit; Hap43-induced; mutants are viable; protein level decreases in stationary phase
orf19.889	THI20	12.87	9.59	10.02	9.79	-0.076039087	NA	Putative trifunctional enzyme of thiamine biosynthesis, degradation and salvage; Spider biofilm induced
orf19.4482	IFI3	10.84	9.57	13.23	11.58	0.089464111	NA	Protein of unknown function; constitutive expression independent of MTL or white-opaque status

orf19.3567	BIO32	9.82	9.57	13.61	10.81	0.112615223	NA	Putative class III aminotransferase with a predicted role in biotin biosynthesis; Spider biofilm induced
orf19.7344	orf19.7344	12.21	9.56	8.58	6.82	-0.156788415	NA	Ortholog(s) have DNA binding, chromatin binding, histone deacetylase activity
orf19.3981	MAL31	10.08	9.55	42.53	28.79	0.912938498	NA	Putative high-affinity maltose transporter; transcript is upregulated in clinical isolates from HIV+ patients with oral candidiasis; alkaline induced; Spider biofilm induced
orf19.234	PHA2	14.24	9.55	9.33	7.91	-0.17580928	NA	Putative prephenate dehydratase; Hap43p-repressed gene; expression downregulated in an ssr1 null mutant
orf19.537	orf19.537	17.66	9.54	14.51	9.51	-0.06910643	NA	Ortholog of C. dubliniensis CD36 : Cd36_29810, C. parapsilosis CDC317 : CPAR2_206030, Candida tenuis NRRL Y-1498 : CANTEDRAFT_102086 and Debaryomyces hansenii CBS767 : DEHA2F14014g
orf19.2945	PUT4	10.63	9.53	16.07	13.44	0.176806153	NA	Putative proline permease; fungal-specific (no human or murine homolog); repressed during the mating process
orf19.1941	NUF2	7.94	9.52	6.33	9.1	-0.082678875	NA	Kinetochore component; amount of Nuf2p and Mtw1p protein detected at each centromere is consistent with a single kinetochore microtubule attachment site
orf19.3537	orf19.3537	11.52	9.51	13.99	7.99	-0.006659898	NA	Putative sulfiredoxin; regulated by Tsa1, Tsa1B in minimal media at 37 degrees C; flow model biofilm induced; Spider biofilm induced
orf19.971	SKN7	16.37	9.51	12.13	11.8	-0.047690411	NA	Putative homolog of S. cerevisiae Skn7p, which is predicted to be a response regulator protein in a phosphorelay signal transduction pathway; required for normal hydrogen peroxide resistance

orf19.5051	orf19.5051	12.36	9.51	11.36	8.19	-0.078979611	NA	Has domain(s) with predicted protein heterodimerization activity
orf19.1668	orf19.1668	10.69	9.5	9.58	8.59	-0.029234983	NA	Protein of unknown function; expression downregulated in an ssr1 null mutant
orf19.290	KRE5	10.42	9.49	12.88	12.58	0.162593404	NA	UDP-glucose:glycoprotein glucosyltransferase; 1,6-beta-D-glucan biosynthesis, hyphal growth, virulence in mouse IV model; partially complements S. cerevisiae kre5 mutant defects; flow biofilm repressed, Bcr1-repressed in RPMI a/a biofilms
orf19.5705	NAM2	13.05	9.48	14.79	12.15	0.109869683	NA	Mitochondrial leucyl-tRNA synthetase
orf19.3473	orf19.3473	14.31	9.46	12.81	9.54	-0.003970086	NA	Ortholog(s) have enzyme activator activity, histone acetyltransferase activity, structural molecule activity
orf19.6166	orf19.6166	13.24	9.45	17.49	12.51	0.173602663	NA	Ortholog of C. dubliniensis CD36 : Cd36_80810, C. parapsilosis CDC317 : CPAR2_101800, Candida tenuis NRRL Y-1498 : CANTEDRAFT_103482 and Debaryomyces hansenii CBS767 : DEHA2F17864g
orf19.6379	orf19.6379	11.17	9.45	16.29	15	0.245443767	NA	Ortholog of C. dubliniensis CD36 : Cd36_33820, C. parapsilosis CDC317 : CPAR2_206210, Candida tenuis NRRL Y-1498 : CANTEDRAFT_92567 and Debaryomyces hansenii CBS767 : DEHA2E06138g
orf19.5842	orf19.5842	9.14	9.43	16.39	19.23	0.377929308	NA	Protein with predicted siroheme synthase middle domains NAD(P)-binding; Hap43-repressed gene
orf19.579	FOL1	12.56	9.43	16.8	11.74	0.15167543	NA	Putative dihydroneopterin aldolase (dihydro-6-hydroxymethylpterin pyrophosphokinase); fungal-specific (no human or murine homolog)

orf19.3368	orf19.3368	2.69	9.42	7.39	1.34	-0.121024953	NA	Ortholog of Candida albicans WO-1 : CAWG_03466
orf19.2663	orf19.2663	11.1	9.41	14.77	11.49	0.135420484	NA	Ortholog of Pichia stipitis Pignal : PICST_29216 and Candida albicans WO-1 : CAWG_00788
orf19.3680	SEP7	11.4	9.38	18.34	16.23	0.306309741	NA	Septin, required for wild-type invasive growth in vitro but not required for virulence in a mouse model of systemic infection; localizes to hyphal septum or bud neck; Asn-rich; aberrant gel mobility; phosphorylated in vitro by Gin4p
orf19.1237	ARO9	13.61	9.35	18.65	20.88	0.347769443	NA	Aromatic transaminase; Ehrlich fusel oil pathway of aromatic alcohol biosynthesis; Rim101-dependent pH-regulation (alkaline induced); Hap43-induced gene
orf19.5879	orf19.5879	8.05	9.32	8.88	10.14	0.047028318	NA	Has domain(s) with predicted oxidoreductase activity and role in metabolic process
orf19.2238	LTE1	8.09	9.31	11.39	10.53	0.120442561	NA	Protein similar to GDP/GTP exchange factors; repressed by alpha pheromone in SpiderM medium; flow model biofilm repressed
orf19.6861	orf19.6861	9.18	9.31	9.3	8.98	-0.004488102	NA	Ortholog(s) have ubiquitin-protein ligase activity and role in anaphase-promoting complex-dependent proteasomal ubiquitin-dependent protein catabolic process, chromatin assembly, protein ubiquitination
orf19.1365	orf19.1365	7.1	9.3	7.57	10.37	0.047870203	NA	Putative monooxygenase; mutation confers hypersensitivity to toxic ergosterol analog; constitutive expression independent of MTL or white-opaque status
orf19.1495	orf19.1495	14.08	9.3	12.37	10.94	-0.003091206	NA	Ortholog(s) have role in L-methionine salvage from methylthioadenosine and cytosol, nucleus localization

orf19.6244	orf19.6244	10.18	9.3	10.55	9.83	0.045104479	NA	Ortholog of C. dubliniensis CD36 : Cd36_06250, C. parapsilosis CDC317 : CPAR2_206820, Candida tenuis NRRL Y-1498 : CANTEDRAFT_125137 and Debaryomyces hansenii CBS767 : DEHA2G13266g
orf19.7358	SAM50	11	9.28	11.94	11.2	0.068306436	NA	Predicted component of the SAM complex involved in mitochondrial protein import
orf19.3430	orf19.3430	5.46	9.28	11.43	8.71	0.148360461	NA	Plasma membrane-associated protein; physically interacts with TAP-tagged Nop1p
orf19.4948	REX3	13.11	9.25	8.15	6.3	-0.209686139	NA	Ortholog(s) have 3'-5'-exoribonuclease activity and role in U5 snRNA 3'-end processing, exonucleolytic trimming to generate mature 3'-end of 5.8S rRNA from tricistronic rRNA transcript (SSU-rRNA, 5.8S rRNA, LSU-rRNA)
orf19.2653	orf19.2653	11.54	9.24	11.51	11.05	0.043994017	NA	Protein with a transient receptor potential (TRP) ion channel domain; mutants are viable; rat catheter and Spider biofilm induced; flow model biofilm repressed
orf19.721	orf19.721	8.44	9.24	10.21	8.14	0.020097717	NA	Ortholog(s) have polynucleotide 5'-hydroxyl-kinase activity
orf19.2383	YKU80	10.44	9.22	14.76	13.94	0.228180276	NA	Yku70p-Yku80p Ku complex subunit involved in nonhomologous end joining during double-strand break repair repair; Hap43-repressed gene; flow model biofilm induced
orf19.3411	orf19.3411	9.39	9.17	13.19	10	0.11708045	NA	Ortholog(s) have role in cellular bud site selection and cytosol, nucleus localization
orf19.4321	orf19.4321	9.59	9.15	6.96	11.3	-0.02734624	NA	Ortholog of Candida albicans WO-1 : CAWG_04677

orf19.2889	orf19.2889	12.51	9.13	7.2	11.51	-0.07282342	NA	Ortholog(s) have role in chromatin remodeling and Ino80 complex, cytoplasm localization
orf19.6821	orf19.6821	11.95	9.13	9.34	10.09	-0.052357542	NA	Ortholog(s) have ubiquitin-protein ligase activity
orf19.5980	orf19.5980	7.4	9.11	13.8	8.87	0.166278604	NA	Protein of unknown function; Hap43-repressed gene
orf19.3879	orf19.3879	12.68	9.11	20.56	14.86	0.292723592	NA	Predicted protein of unknown funtion; overlaps orf19.3879.1, which is a region annotated as blocked reading frame
orf19.1719	SGA1	13.37	9.09	17.27	17.48	0.25748134	NA	Putative glucoamylase; induced in oralpharyngeal candidasis; rat catheter and Spider biofilm induced
orf19.4748	orf19.4748	13.13	9.09	10.07	10.57	-0.026554367	NA	Putative U2B" component of the U2 snRNP, involved in splicing; contains an RNA recognition motif (RRM); ortholog of S. cerevisiae MSL1; Hap43p-induced gene
orf19.3557	GPI19	8.66	9.08	10.32	9.05	0.020559386	NA	Subunit of GPI-GlcNAc transferase; role in synthesis of N-acetylglucosaminyl phosphatidylinositol (GlcNAc-PI), the first intermediate in GPI anchor synthesis; complements S. cerevisiae gpi19 mutant; flow model biofilm induced
orf19.1988	CDC45	13.02	9.07	10	12.12	-0.002078857	NA	Putative DNA replication initiation factor; transcriptionally regulated by interaction with macrophage
orf19.5287	orf19.5287	8.59	9.06	11.3	10.7	0.094920433	NA	Ortholog of C. dubliniensis CD36 : Cd36_30690, C. parapsilosis CDC317 : CPAR2_204140, Debaryomyces hansenii CBS767 : DEHA2F16940g and Candida tropicalis MYA-3404 : CTRG_00570

orf19.5785	orf19.5785	12.08	9.05	6.45	6.88	-0.213168419	NA	Protein of unknown function; upregulated in a cyr1 or ras1 null mutant; induced by nitric oxide
orf19.5851	STE13	8.18	9.03	12.43	11.83	0.167652653	NA	Putative pheromone-processing dipeptidyl aminopeptidase; possible Kex2 substrate; transposon mutation affects filamentous growth; induced by low nitrogen, germ tube formation; flow model biofilm repressed
orf19.7052	orf19.7052	9.83	9.02	15.19	13.03	0.205973651	NA	Putative polyphosphatidylinositol phosphatase; possibly an essential gene, disruptants not obtained by UAU1 method
orf19.7396	orf19.7396	8.39	9.01	8.68	11.77	0.097069904	NA	Protein of unknown function; Spider biofilm induced; Hap43-repressed
orf19.7456	orf19.7456	7.07	9.01	13.92	11.98	0.238086606	NA	Protein of unknown function; flow model biofilm repressed
orf19.5209	orf19.5209	13.32	9.01	11.85	12.96	0.067084296	NA	Ortholog(s) have role in CVT pathway, intra-Golgi vesicle-mediated transport and Golgi transport complex, cell division site, cytosol, nucleus localization
orf19.3950	MSM1	11.35	8.99	10.04	10.35	-0.003724843	NA	Mitochondrial methionyl-tRNA synthetase (MetRS); functionally complements methionine auxotrophy of an E. coli MetRS mutant; transcript regulated by Nrg1; flow model biofilm induced
orf19.2325	orf19.2325	9.94	8.98	8.36	8.36	-0.082201613	NA	Ortholog(s) have RNA polymerase III type 1 promoter sequence-specific DNA binding and RNA polymerase III type 1 promoter sequence-specific DNA binding TFIIIB recruiting transcription factor activity, more
orf19.1641	orf19.1641	11.91	8.97	9.1	10.26	-0.052357542	NA	Ortholog(s) have cytosol, extracellular region, nucleus localization

orf19.1356	orf19.1356	11.78	8.97	15.86	14.54	0.220630207	NA	Ortholog(s) have thiosulfate sulfurtransferase activity, role in tRNA wobble position uridine thiolation and mitochondrion localization
orf19.4601	orf19.4601	12.51	8.95	15.78	10.76	0.108515258	NA	Putative RNA polymerase III transcription initiation factor complex (TFIIIC) subunit; possibly an essential gene, disruptants not obtained by UAU1 method
orf19.6219	orf19.6219	8.81	8.95	16.9	10.85	0.227562541	NA	Ortholog of C. dubliniensis CD36 : Cd36_06470, C. parapsilosis CDC317 : CPAR2_208960, Candida tenuis NRRL Y-1498 : CANTEDRAFT_114040 and Pichia stipitis Pignal : PICST_51125
orf19.2786	orf19.2786	12.47	8.94	14.9	13.78	0.178436327	NA	Ortholog(s) have AP-2 adaptor complex, cellular bud neck localization
orf19.6046	APC1	9.58	8.94	10.55	8.28	-0.005518303	NA	Putative Anaphase-Promoting Complex/Cyclosome subunit; essential for growth; periodic mRNA expression, peak at cell-cycle S/G2 phase
orf19.7157	orf19.7157	11.92	8.94	12.63	8.42	-0.005613418	NA	Protein with a tubulin binding cofactor C domain; flow model biofilm induced
orf19.7348	orf19.7348	10.21	8.93	19.72	10.17	0.239343365	NA	Ortholog of Candida albicans WO-1 : CAWG_02878
orf19.7033	orf19.7033	13.77	8.93	10.56	7.77	-0.100568258	NA	Putative dual specificity protein phosphatase, similar to S. cerevisiae Pps1p
orf19.4161	orf19.4161	11.62	8.92	8.23	7.89	-0.130875237	NA	Ortholog(s) have role in DNA repair and Smc5-Smc6 complex, nucleus localization

orf19.5014	orf19.5014	11.63	8.92	18.35	14.83	0.260558123	NA	Ortholog(s) have mitochondrion localization
orf19.7668	MAL2	10.73	8.89	24.33	16.97	0.435887039	NA	Alpha-glucosidase; hydrolyzes sucrose for sucrose utilization; transcript regulated by Suc1, induced by maltose, repressed by glucose; Tn mutation affects filamentous growth; upregulated in RHE model; rat catheter and Spider biofilm induced
orf19.5392	NGT1	8.52	8.88	12.26	14.19	0.188269701	NA	N-acetylglucosamine (GlcNAc)-specific transporter; role in GlcNAc (but not serum) induced hyphal growth; localizes to plasma membrane; induced by GlcNAc, macrophage engulfment; 12 transmembrane, major facilitator superfamily
orf19.1373	INP51	11.37	8.88	10.91	9.29	-0.004658132	NA	Putative phosphatidylinositol-4,5-bisphosphate phosphatase; involved in maintenance of phosphoinositide levels; affects hyphal growth, virulence, cell integrity; interacts with Irs4p
orf19.2440	orf19.2440	12.28	8.88	10.73	9.14	-0.028801952	NA	S. cerevisiae ortholog Rtt101 is a cullin subunit of E3 ubiquitin ligase complex with a role in anaphase progression; induced by alpha pheromone in SpiderM medium
orf19.2500	orf19.2500	11.82	8.87	11.3	9.33	-0.028801952	NA	Has domain(s) with predicted transferase activity and role in biosynthetic process
orf19.3608	MSH3	10.81	8.86	10.6	10.54	0.045008956	NA	Mismatch repair protein; predicted role in repair of insertion or deletion mutations and removal of nonhomologous DNA ends; rat catheter biofilm repressed
orf19.3107	orf19.3107	6.19	8.84	3.09	1.76	-0.31205369	NA	Ortholog of C. dubliniensis CD36 : Cd36_46490, Pichia stipitis Pignal : PICST_33598, Spathaspora passalidarum NRRL Y-27907 : spas_CGOB_00037 and Candida tropicalis MYA-3404 : CTRG_03758
orf19.11	orf19.11	7.71	8.83	12.17	16.08	0.257629023	NA	Protein of unknown function; transcript regulated by Mig1 and Tup1; rat catheter biofilm induced

orf19.4535	PTR3	9.92	8.8	12.5	9.09	0.067557408	NA	Protein similar to S. cerevisiae Ptr3p, which is a sensor of external amino acids; expression upregulated in an ssr1 null mutant
orf19.210	orf19.210	13.26	8.79	8.36	8.76	-0.127205475	NA	Ortholog(s) have role in protein localization to bud neck and cellular bud neck, cytosol localization
orf19.6038	UGA32	9.43	8.79	19.34	15.52	0.376597476	NA	Predicted Zn(II)2Cys6 transcription factor; has similarity to S. cerevisiae Uga3, a transcription factor involved in the regulation of gamma-aminobutyrate metabolism genes; rat catheter biofilm induced
orf19.7331	FCY24	9.26	8.79	7.36	9.11	-0.055812888	NA	Putative transporter; more similar to S. cerevisiae Tpn1, which is a vitamin B6 transporter, than to purine-cytosine permeases; transcription is regulated by Nrg1; Spider biofilm induced
orf19.1497	ZCF6	12.15	8.77	9.6	10.1	-0.027895641	NA	Putative transcription factor with zinc cluster DNA-binding motif
orf19.5259	orf19.5259	7.97	8.77	13.52	9.8	0.165097054	NA	Ortholog of C. dubliniensis CD36 : Cd36_11290, C. parapsilosis CDC317 : CPAR2_805090, Pichia stipitis Pignal : PICST_28850 and Candida guilliermondii ATCC 6260 : PGUG_02709
orf19.3549	CDC21	11.16	8.77	18.09	20.07	0.385339111	NA	Putative thymidylate synthase; flucytosine induced; rat catheter biofilm repressed; Spider biofilm repressed
orf19.6670	CAC2	9.99	8.77	8.75	8.07	-0.056141986	NA	Component of the chromatin assembly factor I (CAF-1); involved in regulation of white-opaque switching frequency; macrophage-induced
orf19.6557	orf19.6557	8.11	8.75	10.85	11.81	0.144554341	NA	Protein with a predicted fatty acid amide hydrolase I domain; induced by Mnl1 under weak acid stress

orf19.6547	orf19.6547	9.91	8.74	9.3	10.46	-0.003619998	NA	Ortholog of Candida albicans WO-1 : CAWG_05528
orf19.7305	orf19.7305	9.32	8.74	12.2	9.15	0.069127393	NA	Ortholog of C. dubliniensis CD36 : Cd36_34510, Candida tropicalis MYA-3404 : CTRG_05938 and Candida albicans WO-1 : CAWG_02183
orf19.3788	SPC34	7.7	8.73	7.68	3.58	-0.143374037	NA	Subunit of the Dam1 (DASH) complex, which acts in chromosome segregation by coupling kinetochores to spindle microtubules
orf19.3715	ASF1	12.07	8.73	11.01	10.75	0.020748016	NA	Protein similar to S. cerevisiae Asf1p, a chromatin assembly complex component; likely to be essential for growth, based on an insertional mutagenesis strategy
orf19.5221	orf19.5221	9.35	8.73	9.89	9.26	0.020559386	NA	Ortholog(s) have phosphatidylinositol-4,5-bisphosphate binding activity
orf19.2253	orf19.2253	8.01	8.72	9.42	5.36	-0.087186	NA	Protein of unknown function; expression regulated by white-opaque switch; repressed by alpha pheromone in SpiderM medium; Spider biofilm induced
orf19.1685	ZCF7	10.24	8.71	16.14	12.55	0.226982163	NA	Predicted Zn(II)2Cys6 transcription factor; mutant shows reduced colony wrinkling on Spider medium and is unable to utilize mannitol as a carbon source; mutants have increased colonization of mouse kidneys
orf19.663	GIN4	8.97	8.69	18.97	12.05	0.290311296	NA	Autophosphorylated kinase; role in pseudohyphal-hyphal switch and cytokinesis; phosphorylates Cdc11p on S395; necessary for septin ring within germ tube but not for septin band at mother cell junction; physically associates with septins
orf19.5526	SEC20	12.27	8.68	16.95	12.42	0.175761104	NA	Essential protein; similar to S. cerevisiae Sec20p; depletion causes membrane accumulation and drug sensitivity; expression regulated by growth phase; O-mannosylation regulates proteolysis; does not complement S. cerevisiae sec20-1 mutant

orf19.232	orf19.232	12.2	8.68	8.46	9.48	-0.103737112	NA	RNA polymerase II mediator complex subunit; possibly an essential gene, disruptants not obtained by UAU1 method
orf19.4982	orf19.4982	8.45	8.67	7.06	8.65	-0.030257115	NA	Has domain(s) with predicted role in lipid metabolic process
orf19.3761	CDC54	9.89	8.67	21.2	17.63	0.428480703	NA	Putative pre-replication complex helicase subunit; transcript regulated by Nrg1 and Mig1; periodic mRNA expression, peak at cell-cycle M/G1 phase; Hap43-induced
orf19.4470	orf19.4470	13.35	8.65	11.87	10.43	-0.003898884	NA	Protein of unknown function; Hap43-repressed gene
orf19.6242	CYK3	10.43	8.65	8.05	8.43	-0.082201613	NA	Essential protein involved in cytokinesis; contains an SH3 domain
orf19.810	orf19.810	9.07	8.63	10.07	8.8	0.020559386	NA	Ortholog(s) have DNA helicase activity, role in DNA unwinding involved in DNA replication, reciprocal meiotic recombination, synapsis and nucleus localization
orf19.4231	PTH2	8.32	8.63	3.29	6.26	-0.229154791	NA	Putative cAMP-independent regulatory protein; constitutive expression independent of MTL or white-opaque status; Spider biofilm induced
orf19.6356	orf19.6356	12.73	8.62	9.4	6.44	-0.182022845	NA	Ortholog(s) have role in mRNA splicing, via spliceosome and U4/U6 x U5 tri-snRNP complex localization
orf19.6690	orf19.6690	9.69	8.61	9.09	10.3	-0.003619998	NA	Protein of unknown function; Hap43-repressed gene

orf19.1434	orf19.1434	9.81	8.61	9.87	10.47	0.020955566	NA	Ortholog(s) have protein kinase activator activity
orf19.1828	BUD16	11.12	8.6	13.77	12.33	0.136754527	NA	Putative pyridoxal kinase; a key enzyme in pyridoxal 5'-phosphate synthesis, the active form of vitamin B6; involved in bud-site selection and genome integrity in S. cerevisiae; induced by alpha pheromone in SpiderM medium
orf19.6536	IQG1	7.67	8.59	11.27	7.72	0.045001984	NA	Actomyosin ring component at bud neck; cell-cycle regulated ser phosphorylation at CDK sites regulate association with Bni1/Bnr1, Iqg1 degradation, and ring disassembly; mutation causes cytokinetic defects; rat catheter biofilm repressed
orf19.5429	orf19.5429	10.86	8.58	10.38	6.64	-0.081426169	NA	Ortholog(s) have DNA replication origin binding, ubiquitin-protein ligase activity
orf19.1718	ZCF8	10.19	8.58	9.03	8.02	-0.056141986	NA	Predicted Zn(II)2Cys6 transcription factor; repressed by fluphenazine treatment; required for yeast cell adherence to silicone substrate
orf19.1664	orf19.1664	7.58	8.57	15.48	10.88	0.210815746	NA	Protein of unknown function; expression downregulated in an ssr1 null mutant
orf19.652	orf19.652	10.27	8.55	10.06	6.55	-0.056902486	NA	Ortholog(s) have crossover junction endodeoxyribonuclease activity, role in DNA repair and cytoplasm, nucleus localization
orf19.7225	orf19.7225	9.59	8.52	11.1	5.07	-0.083404347	NA	Ortholog of C. dubliniensis CD36 : Cd36_13150, C. parapsilosis CDC317 : CPAR2_700610, Candida tenuis NRRL Y-1498 : CANTEDRAFT_135418 and Debaryomyces hansenii CBS767 : DEHA2B01298g
orf19.6629	ISC1	10.36	8.51	9.91	10.04	0.020955566	NA	Mitochondrial membrane localized inositol phosphosphingolipid phospholipase C; hydrolyzes complex sphingolipids to produce ceramide; mutant is viable

orf19.7324	THI13	9.79	8.5	15.48	14.73	0.249920093	NA	Thiamin pyrimidine synthase; synthesis of the thiamine precursor hydroxymethylpyrimidine phosphate; single-turnover enzyme that provides histidine for HMP-P formation; induced by nitric oxide independent of Yhb1; Spider biofilm induced
orf19.7190	OGG1	9.39	8.5	6.72	6.36	-0.140545034	NA	Mitochondrial glycosylase/lyase; repairs oxidative damage to mitochondrial DNA, contributes to UVA resistance, role in base-excision repair; Spider biofilm induced
orf19.538	GPI2	7.24	8.48	5.16	8.45	-0.058166228	NA	Subunit of the GPI-N-acetylglucosaminyl transferase (GPI-GnT) that catalyzes the first step in GPI anchor biosynthesis
orf19.1508	orf19.1508	5.45	8.48	9.07	7.25	0.075486545	NA	Protein of unknown function; transcript detected on high-resolution tiling arrays
orf19.3457	SWD3	11.89	8.47	13.32	10.72	0.091714154	NA	Ortholog(s) have histone methyltransferase activity (H3-K4 specific) activity, role in chromatin silencing at telomere, histone H3-K4 methylation, telomere maintenance and Set1C/COMPASS complex localization
orf19.2818	orf19.2818	10.36	8.47	7.88	6.27	-0.11137062	NA	Ortholog(s) have RNA-dependent ATPase activity, second spliceosomal transesterification activity, role in generation of catalytic spliceosome for second transesterification step and U2-type catalytic step 2 spliceosome localization
orf19.2982	orf19.2982	10.3	8.47	16.64	12.21	0.251191084	NA	Ortholog(s) have protein complex scaffold, protein kinase activator activity
orf19.6449	orf19.6449	8.87	8.47	7.26	7.55	-0.056794548	NA	Ortholog of C. dubliniensis CD36 : Cd36_34310
orf19.2051	orf19.2051	9.43	8.47	12.33	10.35	0.120646936	NA	Ortholog of C. dubliniensis CD36 : Cd36_15670, C. parapsilosis CDC317 : CPAR2_213100, Candida tenuis NRRL Y-1498 : CANTEDRAFT_135034 and Debaryomyces hansenii CBS767 : DEHA2E14960g

orf19.4702	orf19.4702	10.07	8.47	12.23	9.99	0.094874034	NA	Possible similarity to mutator-like element (MULE) transposase; flow model biofilm induced; expression regulated during planktonic growth
orf19.1803	CDC43	8.94	8.45	7.62	4.86	-0.11415898	NA	Beta subunit of heterodimeric protein geranylgeranyltransferase type I; GGTase I enzyme binds zinc, is Mg-dependent; Cdc42p is GGTase I substrate
orf19.3255	TEN1	8.43	8.43	6.83	4.73	-0.11672112	NA	Protein involved in telomere maintenance; forms a complex with Stn1; transcription is regulated upon yeast-hypha switch; Spider biofilm induced
orf19.4975	HYR1	8.34	8.41	129.86	75.38	2.162124141	2.33E-12	GPI-anchored hyphal cell wall protein; macrophage-induced; repressed by neutrophils; resistance to killing by neutrophils, azoles; regulated by Rfg1, Efg1, Nrg1, Tup1, Cyr1, Bcr1, Hap43; Spider and flow model biofilm induced
orf19.356	GTT13	7.7	8.41	12.71	10.07	0.169969377	NA	Putative glutathione S-transferase; opaque-specific transcript; repressed by alpha pheromone in SpiderM medium; Spider biofilm induced
orf19.5169	orf19.5169	9.6	8.41	10.97	6.77	-0.005327622	NA	Ortholog(s) have cytosol, nucleus localization
orf19.1638	orf19.1638	10.41	8.41	14.43	10.52	0.164386797	NA	Has domain(s) with predicted zinc ion binding activity, role in ER to Golgi vesicle-mediated transport, intracellular protein transport and COPII vesicle coat localization
orf19.433	orf19.433	6.18	8.41	12.22	12.63	0.271795338	NA	Ortholog(s) have Rab guanyl-nucleotide exchange factor activity, role in intra-Golgi vesicle-mediated transport, protein complex assembly and TRAPP complex, trans-Golgi network localization
orf19.6917	orf19.6917	11.56	8.4	6.29	9.78	-0.101831799	NA	Putative heat shock protein with a zinc finger motif; required for protein import into mitochondria in S. cerevisiae; Spider biofilm induced

orf19.687	orf19.687	9.48	8.37	8.68	9.23	0.022207704	NA	Ortholog of C. dubliniensis CD36 : Cd36_62090, C. parapsilosis CDC317 : CPAR2_602150, Candida tenuis NRRL Y-1498 : CANTEDRAFT_112751 and Debaryomyces hansenii CBS767 : DEHA2F11814g
orf19.3565	orf19.3565	12.78	8.37	11.65	8.35	-0.028378264	NA	Ortholog of C. dubliniensis CD36 : Cd36_19830, C. parapsilosis CDC317 : CPAR2_206470, Candida tenuis NRRL Y-1498 : CANTEDRAFT_113899 and Debaryomyces hansenii CBS767 : DEHA2C05676g
orf19.612	orf19.612	10.45	8.36	12.32	8.52	0.070102908	NA	Ortholog of S. cerevisiae : AIM32, C. glabrata CBS138 : CAGL0L01529g, C. dubliniensis CD36 : Cd36_33550, C. parapsilosis CDC317 : CPAR2_702070 and Candida tenuis NRRL Y-1498 : CANTEDRAFT_114174
orf19.7413	MMS21	11.38	8.36	11.81	7.87	0.019925659	NA	Putative MMS21-SMC5-SMC6 complex component
orf19.274	TFC4	9.39	8.34	11.79	10.11	0.120646936	NA	Putative RNA polymerase III transcription initiation factor complex (TFIIIC) subunit; induced by Mnl1 under weak acid stress; possibly essential gene, disruptants not obtained by UAU1 method
orf19.2738	SUL2	12.49	8.33	23.8	13.56	0.373922342	NA	Putative sulfate transporter; transcript negatively regulated by Sfu1; amphotericin B induced; F-12/CO2 and Spider biofilm induced
orf19.6537	orf19.6537	6.36	8.3	8.28	5.24	-0.034472398	NA	Ortholog(s) have role in chromosome segregation and nuclear MIS12/MIND complex, spindle pole localization
orf19.651	LYP1	8.31	8.3	7.23	8.05	-0.030702853	NA	Putative permease; amphotericin B induced; flucytosine repressed; possibly an essential gene, disruptants not obtained by UAU1 method
orf19.779	HAT1	6.78	8.29	9.92	7.96	0.073820834	NA	Hat1-Hat2 histone acetyltransferase complex subunit; involved in DNA damage repair and morphogenesis; mutations cause constitutive pseudohyphal growth, white to opaque switch, caspofungin sensitivity; rat catheter, Spider biofilm repressed

orf19.264	orf19.264	9.94	8.28	8.95	7.57	-0.029890524	NA	Has domain(s) with predicted protein kinase binding activity and role in regulation of cyclin-dependent protein serine/threonine kinase activity
orf19.3999	orf19.3999	9.86	8.25	11.51	11.51	0.143020374	NA	Ortholog(s) have fungal-type vacuole membrane localization
orf19.727	orf19.727	10.26	8.25	10.32	8.64	0.02159714	NA	Predicted ORF overlapping the Major Repeat Sequence on chromosome R; member of a family encoded by FGR6-related genes in the RB2 repeat sequence
orf19.5644	orf19.5644	7.63	8.24	8.22	11.3	0.074837607	NA	Ortholog(s) have role in ascospore wall assembly, spore membrane bending pathway and cytoplasm, prospore membrane localization
orf19.1855	orf19.1855	11.15	8.22	11.3	9.31	0.020971273	NA	Predicted membrane transporter, member of the anion:cation symporter (ACS) family, major facilitator superfamily (MFS); Gcn4p-regulated; flucytosine induced; ketoconazole-repressed; oxidative stress-induced via Cap1p
orf19.3648	orf19.3648	8.94	8.2	10.07	9.23	0.047256115	NA	Ortholog(s) have crossover junction endodeoxyribonuclease activity, transcription coactivator activity
orf19.57	PSF2	7.58	8.2	10.07	10.7	0.12397918	NA	Ortholog(s) have role in DNA-dependent DNA replication, cellular protein localization, double-strand break repair via break-induced replication, meiotic chromosome segregation
orf19.1465	orf19.1465	11.41	8.2	12.44	6.04	-0.030836867	NA	Has domain(s) with predicted N-acetyltransferase activity
orf19.2593	BIO2	8.85	8.18	13.65	10.32	0.16637135	NA	Putative biotin synthase; induced by high iron; repressed by ciclopirox olamine; upregulated in clinical isolates from HIV+ patients with oral candidiasis; Hap43-repressed; Spider biofilm induced

orf19.7371	ZCF35	10.89	8.16	9.36	5.43	-0.136097547	NA	Zn(II)2Cys6 transcription factor; Hap43-induced; Spider biofilm induced
orf19.1488	orf19.1488	12.53	8.15	7.26	11.62	-0.048269187	NA	Ortholog of C. dubliniensis CD36 : Cd36_16630, C. parapsilosis CDC317 : CPAR2_210300, Candida tenuis NRRL Y-1498 : CANTEDRAFT_116870 and Debaryomyces hansenii CBS767 : DEHA2B04334g
orf19.4142	orf19.4142	5.55	8.14	9.23	12.18	0.177269848	NA	Putative transporter; decreased transcription is observed upon fluphenazine treatment
orf19.1041	orf19.1041	9.02	8.14	8.87	7.11	-0.031278944	NA	Ortholog(s) have cyclin-dependent protein serine/threonine kinase activity
orf19.5568	VPS51	11.13	8.14	12.48	9.9	0.069596532	NA	Protein with a role in vacuolar function; null mutant has defect in damaging oral epithelial and vascular endothelial cells; required for normal hyphal growth and stress resistance; induced in presence of host oral or vascular cells
orf19.4778	LYS142	9.92	8.13	10.79	8.11	0.020569298	NA	Zn(II)2Cys6 transcription factor; has similarity to S. cerevisiae Lys14, involved in the regulation of lysine biosynthesis genes; fungal-specific
orf19.2352	orf19.2352	10.65	8.13	14.87	15.25	0.250162757	NA	Ortholog(s) have mitochondrion localization
orf19.5017	DUR32	10.2	8.13	9.61	7.72	-0.004383899	NA	Putative urea transporter; possibly an essential gene, disruptants not obtained by UAU1 method
orf19.6037	ASM3	8.88	8.09	11.9	10.33	0.120646936	NA	Putative secreted acid sphingomyelin phosphodiesterase; possible Kex2 substrate; transcript increased in an azole-resistant strain that overexpresses MDR1; flow model biofilm induced; F-12/CO2 early biofilm induced

orf19.1255	ZCF5	9.79	8.08	12.69	8.82	0.093531527	NA	Zn(II)2Cys6 transcription factor; colony morphology-related gene regulation by Ssn6
orf19.2547	orf19.2547	7.77	8.08	7.25	6.13	-0.087598999	NA	Has domain(s) with predicted RNA binding, ribonuclease activity
orf19.3042	orf19.3042	5.76	8.06	8.04	6.89	0.021948723	NA	Protein of unknown function; Hap43-induced; rat catheter biofilm induced
orf19.1040	MAD2	8.66	8.06	6.25	9.82	-0.027919585	NA	Protein required for the spindle assembly checkpoint of the cell cycle; necessary for survival in host cell macrophages and for virulence in a mouse model of systemic candidiasis
orf19.5586	orf19.5586	9.3	8.05	10.27	9.83	0.072808317	NA	Ortholog(s) have phosphatidylinositol-3,5-bisphosphate 5-phosphatase activity, role in phosphatidylinositol dephosphorylation and PAS complex, cytosol, extrinsic to membrane, fungal-type vacuole membrane localization
orf19.1307	orf19.1307	7.93	8.05	7.66	5.19	-0.08814305	NA	Predicted membrane protein; rat catheter biofilm induced
orf19.4823	LIP6	4.92	8.04	8.28	8.35	0.076629804	NA	Secreted lipase, member of family of lipase genes expressed differentially in response to carbon source and during infection; may have a role in nutrition and/or in creating an acidic microenvironment; induced on adherence to polystyrene
orf19.2495	GSL1	7.92	8.04	9.14	8.95	0.048340142	NA	Beta-1,3-glucan synthase subunit; 10 predicted transmembrane regions; caspofungin induced; repressed by yeast-to-hypha transition; young biofilm repressed, induced by biofilm drug exposure; Bcr1-repressed in RPMI a/a biofilms
orf19.3623	SMC2	7.99	8.04	7.97	7.6	-0.004198508	NA	Protein similar to S. cerevisiae Smc2p, which is a component of the condensin complex involved in mitotic chromosome condensation; induced under hydroxyurea treatment

orf19.6012	orf19.6012	9.09	8.03	12.93	11.78	0.191585955	NA	Ortholog(s) have cell division site, cell tip, cytosol localization
orf19.5664	HOF1	9.05	8.03	10.04	8.11	0.021205223	NA	Ortholog of S. cerevisiae Hof1; a protein localized to the bud neck and required for cytokinesis in S. cerevisiae; periodic mRNA expression, peak at cell-cycle G2/M phase; mutant is viable
orf19.286	orf19.286	11.68	8.03	9.47	11.28	-0.00172543	NA	Has a predicted autophagy-related protein domain; transcription repressed by fluphenazine treatment
orf19.246	orf19.246	10.85	8.03	11.81	11.22	0.093821971	NA	Predicted metalloendopeptidase; Spider biofilm induced
orf19.1360	orf19.1360	6.42	8.02	5.6	9.2	0.0238134	NA	Ortholog(s) have role in mitochondrial genome maintenance and mitochondrion localization
orf19.1789	orf19.1789	12.35	8.02	9.4	9.14	-0.052955114	NA	Ortholog(s) have mitochondrion localization
orf19.7632	orf19.7632	7.39	8.01	11.86	7.99	0.122825609	NA	Ortholog of C. dubliniensis CD36 : Cd36_35360, C. parapsilosis CDC317 : CPAR2_200320, Candida tenuis NRRL Y-1498 : CANTEDRAFT_136418 and Debaryomyces hansenii CBS767 : DEHA2A14080g
orf19.2851	orf19.2851	9.58	8.01	9.38	7.81	-0.029890524	NA	Putative ornithine transport protein; localized to the mitochondrial membrane
orf19.1496	orf19.1496	8.81	7.99	7.83	8.51	-0.003303719	NA	Putative transcription factor with zinc finger DNA-binding motif; Hap43p-repressed gene

orf19.4988	orf19.4988	9.57	7.97	10.64	6.97	-0.005327622	NA	S. cerevisiae ortholog Sae2 is an endonuclease that processes hairpin DNA structures with the MRX complex, involved in meiotic and mitotic double-strand break repair; upregulated in a cyr1 null mutant
orf19.131	orf19.131	4.27	7.96	11.35	6.52	0.152507694	NA	Ortholog of C. dubliniensis CD36 : Cd36_61330, C. parapsilosis CDC317 : CPAR2_602800, Candida tenuis NRRL Y-1498 : CANTEDRAFT_94450 and Debaryomyces hansenii CBS767 : DEHA2F15554g
orf19.6453	orf19.6453	9.12	7.96	9.28	6.87	-0.031278944	NA	Ortholog of C. dubliniensis CD36 : Cd36_72300, C. parapsilosis CDC317 : CPAR2_703060, Candida tenuis NRRL Y-1498 : CANTEDRAFT_92002 and Debaryomyces hansenii CBS767 : DEHA2B06028g
orf19.3721	orf19.3721	12.85	7.95	7.94	5.49	-0.211850339	NA	Protein of unknown function; Spider biofilm induced
orf19.1286	orf19.1286	7.36	7.93	1.69	0.56	-0.385894004	NA	Ortholog of Candida albicans WO-1 : CAWG_04776
orf19.1667	orf19.1667	10.32	7.92	8.83	7.65	-0.029890524	NA	Protein with a predicted SWI/SNF-related matrix-associated actin-dependent regulator of chromatin subfamily domain; Hap43-repressed gene
orf19.1728	orf19.1728	7.61	7.91	7.29	10.32	0.023647135	NA	Ortholog of C. dubliniensis CD36 : Cd36_81190, C. parapsilosis CDC317 : CPAR2_503840, Debaryomyces hansenii CBS767 : DEHA2A09042g and Pichia stipitis Pignal : PICST_42519
orf19.1693	CAS4	8.06	7.91	11.34	10.69	0.147886636	NA	RAM cell wall integrity signaling network protein; cell separation, azole sensitivity; needed for hyphal growth; insertion mutation near 3' end of gene increases caspofungin sensitivity; pheromone/hyphal induced; flow biofilm repressed
orf19.5578	orf19.5578	4.94	7.9	6.57	3.83	-0.064263462	NA	Ortholog of C. dubliniensis CD36 : Cd36_63050 and Candida albicans WO-1 : CAWG_05094

orf19.4763	orf19.4763	17.19	7.89	17.43	20.23	0.242534949	NA	Protein of unknown function; possibly membrane bound; mutants are viable; rat catheter biofilm repressed
orf19.5157	orf19.5157	9.15	7.89	14.48	8.81	0.142012348	NA	Protein with a protein tyrosine phosphatase-like protein domain; putative membrane-spanning regions; rat catheter biofilm induced
orf19.1270	FRE3	6.86	7.87	7.6	6.33	-0.033154288	NA	Protein with similarity to ferric reductase Fre10p; possibly an essential gene, disruptants not obtained by UAU1 method
orf19.3222	orf19.3222	10.13	7.86	15.25	12.73	0.232088932	NA	Predicted vacuolar protein; rat catheter biofilm repressed; flow model biofilm repressed
orf19.2519	orf19.2519	12.12	7.86	11.11	5.88	-0.080216887	NA	Ortholog(s) have role in chromosome segregation and nuclear MIS12/MIND complex localization
orf19.6592	orf19.6592	10.17	7.86	9.2	10.97	0.04790279	NA	Predicted membrane transporter, member of the aromatic acid:proton symporter (AAHS) family, major facilitator superfamily (MFS)
orf19.175	orf19.175	8.28	7.8	13.76	10.89	0.216574398	NA	Ortholog of C. dubliniensis CD36 : Cd36_27430, C. parapsilosis CDC317 : CPAR2_802450, Candida tenuis NRRL Y-1498 : CANTEDRAFT_113969 and Debaryomyces hansenii CBS767 : DEHA2A13002g
orf19.1484	orf19.1484	4.27	7.79	8.28	6.27	0.048928996	NA	Ortholog of S, cerevisiae Spc24; a component of the conserved kinetochore-associated Ndc80 complex involved in chromosome segregation, spindle checkpoint activity and kinetochore clustering; Hap43-induced gene
orf19.4574	orf19.4574	11.23	7.78	8.87	5.99	-0.108869437	NA	Ortholog(s) have lipid particle localization

orf19.6898	orf19.6898	11.57	7.78	9.86	9.02	-0.028053788	NA	Protein similar to S. pombe SPBC1709.16c a predicted aromatic ring-opening dioxygenase; induced by benomyl treatment or in azole-resistant strain that overexpresses MDR1; Spider biofilm induced
orf19.6286	orf19.6286	8.43	7.77	11.96	10.66	0.171301626	NA	Ortholog(s) have role in vacuolar transport and endosome localization
orf19.3261	ATO9	7.39	7.76	9.21	11.79	0.150685464	NA	Putative fungal-specific transmembrane protein
orf19.5311	orf19.5311	9.84	7.73	13.6	10.83	0.164386797	NA	Ortholog of C. dubliniensis CD36 : Cd36_43850, C. parapsilosis CDC317 : CPAR2_402060, Pichia stipitis Pignal : PICST_68242, Candida tropicalis MYA-3404 : CTRG_00303 and Candida albicans WO-1 : CAWG_03390
orf19.3589	SPO11	7.12	7.72	4.26	4.87	-0.178811768	NA	DNA endonuclease; required for genetic recombination between homologous chromosomes during the parasexual cycle; produced in mitotically dividing cells; similar to S. cerevisiae Spo11 which functions in recombination during meiosis
orf19.1515	CHT4	7.07	7.71	13.15	7.69	0.146410527	NA	Chitinase; similar to S. cerevisiae sporulation-specific Cts2p; functionally complements A. gossypii cts2 mutant sporulation defect; homozygous null mutation causes no obvious defects; transcription decreases upon yeast-to-hyphal switch
orf19.3490	FGR6-4	14.8	7.7	18.9	14.68	0.230053527	NA	Protein lacking an ortholog in S. cerevisiae; member of a family encoded by FGR6-related genes in the RB2 repeat sequence; transposon mutation affects filamentous growth
orf19.6986	orf19.6986	8.96	7.7	7.26	8.26	-0.056794548	NA	Has domain(s) with predicted Rab GTPase activator activity, role in regulation of Rab GTPase activity and intracellular localization
orf19.2065	orf19.2065	7.14	7.69	11.5	10.95	0.174428998	NA	Ortholog(s) have role in allantoin catabolic process and cytosol, nucleus localization

orf19.639	orf19.639	6.42	7.67	11.56	9.52	0.200044742	NA	S. cerevisiae ortholog YDR370C/DXO1 has decapping and 5′-3′ exoRNase activity; decreased transcription is observed upon fluphenazine treatment or in an azole-resistant strain that overexpresses CDR1 and CDR2
orf19.3538	FRE9	9.2	7.63	6.96	7.69	-0.056794548	NA	Ferric reductase; alkaline induced; ciclopirox olamine; Hap43-induced
orf19.4069	orf19.4069	2.97	7.63	6.76	5.07	0.021090169	NA	Protein of unknown function; repressed by alpha pheromone in SpiderM medium
orf19.5203	orf19.5203	10.59	7.63	9.17	10.79	0.022804632	NA	Protein of unknown function; Hap43-induced gene
orf19.3685	PSY2	8.51	7.63	7.24	6.98	-0.085030758	NA	Putative protein phosphatase PP4 complex subunit; macrophage-induced gene
orf19.1681	orf19.1681	6.93	7.62	10.02	9.32	0.099943323	NA	Ortholog of C. dubliniensis CD36 : Cd36_81530, C. parapsilosis CDC317 : CPAR2_503590, Candida tenuis NRRL Y-1498 : CANTEDRAFT_115755 and Debaryomyces hansenii CBS767 : DEHA2D15334g
orf19.6243	VPS34	9.55	7.62	11.68	7.06	0.019449174	NA	Autophosphorylated class III phosphatidylinositol 3-kinase; required for normal vesicle transport, hyphal growth, fibroblast adherence, virulence in mouse systemic infection; growth-regulated; caspofungin and hydrogen peroxide sensitivity
orf19.2442	orf19.2442	12.77	7.61	9.76	5.69	-0.129810011	NA	Ortholog of S. cerevisiae Shg1; a COMPASS (Set1C) complex subunit that methylates histone H3 on lysine 4 and is required in transcriptional silencing near telomeres; repressed in an azole-resistant strain overexpressing MDR1
orf19.2072	orf19.2072	9.01	7.6	10.83	8.23	0.046137247	NA	Ortholog(s) have (R)-carnitine transmembrane transporter activity, choline transmembrane transporter activity, ethanolamine transmembrane transporter activity

orf19.996	orf19.996	7.4	7.6	8.18	4.98	-0.061556102	NA	Protein with a predicted leucine-rich repeat domain; possibly an essential gene, disruptants not obtained by UAU1 method
orf19.3247	orf19.3247	7.98	7.59	8.94	10.28	0.074284199	NA	Putative ortholog of S. cerevisiae Laa1p; likely to be essential for growth, based on an insertional mutagenesis strategy
orf19.4080	orf19.4080	7.95	7.59	8.63	9.16	0.048340142	NA	Putative ribonuclease H2 subunit; required for RNase H2 activity; repressed in Spider biofilms by Bcr1, Tec1, Brg1, Rob1
orf19.6918	orf19.6918	7.72	7.59	5.9	6.16	-0.11595246	NA	Protein of unknown function; Hap43-induced gene; Spider biofilm induced
orf19.5008.1	DAD1	15.84	7.57	12.36	12.36	-0.001295803	NA	Subunit of the Dam1 (DASH) complex, which acts in chromosome segregation by coupling kinetochores to spindle microtubules
orf19.4439	orf19.4439	7.58	7.57	6.67	5.77	-0.087598999	NA	Ortholog of Candida albicans WO-1 : CAWG_00687
orf19.2678	BUB1	9.2	7.56	11.22	8.09	0.046137247	NA	Putative cell cycle checkpoint kinase; mutation confers increased sensitivity to nocodazole
orf19.5919	MEA1	10.88	7.55	9.88	8.6	-0.003518493	NA	Protein similar to A. nidulans MesA, which is involved in localization of actin cables; Hap43p-induced gene
orf19.4243	orf19.4243	8.63	7.55	12.91	8.17	0.094180439	NA	Ortholog(s) have ubiquitin binding activity

orf19.115	orf19.115	9.55	7.54	11.73	9.51	0.094874034	NA	Ortholog of Candida tropicalis MYA-3404 : CTRG_02973 and Candida albicans WO-1 : CAWG_05252
orf19.4414	orf19.4414	11.54	7.53	7.83	5.11	-0.188411801	NA	Ortholog of C. dubliniensis CD36 : Cd36_45590, C. parapsilosis CDC317 : CPAR2_501450, Candida tenuis NRRL Y-1498 : CANTEDRAFT_105024 and Debaryomyces hansenii CBS767 : DEHA2D12364g
orf19.2541	orf19.2541	10.08	7.52	10.2	9.74	0.047106424	NA	Ortholog(s) have 3'-5'-exodeoxyribonuclease activity, endonuclease activity, role in apoptotic DNA fragmentation, cellular response to oxidative stress and cytosol, nucleus localization
orf19.3431	orf19.3431	8.17	7.52	8.14	7.05	-0.03176315	NA	Ortholog(s) have 3'-5' exonuclease activity, DNA-directed DNA polymerase activity, role in mitochondrial DNA replication and mitochondrion localization
orf19.7186	CLB4	8.93	7.52	9.16	8.27	-0.004297987	NA	B-type mitotic cyclin; nonessential; negative regulator of pseudohyphal growth; dispensible for mitotic exit, cytokinesis; Fkh2-represed; flow model biofilm repressed; farnesol-upregulated in biofilm; reduced total RNA in clb4 mutant
orf19.2908	orf19.2908	10.65	7.51	11.5	9.23	0.020971273	NA	Predicted oxidoreductase; rat catheter biofilm induced
orf19.1382	YEA4	8.27	7.5	15.73	13.23	0.333928034	NA	Putative uridine diphosphate-N-acetylglucosamine (UDP-GlcNAc) transporter; required for cell wall chitin synthesis; induced by alpha pheromone in SpiderM medium
orf19.7594	orf19.7594	7.5	7.5	9.57	7.78	0.102244333	NA	Protein of unknown function; Spider biofilm induced
orf19.5580	TEL1	8.41	7.49	11.97	11.71	0.223180405	NA	Ortholog(s) have protein serine/threonine kinase activity, telomeric DNA binding activity and role in DNA damage induced protein phosphorylation, double-strand break repair, histone phosphorylation, telomere maintenance

orf19.4002	DUN1	10.74	7.47	11.65	8.96	0.07074086	NA	Protein similar to S. cerevisiae Dun1p, which is a serine-threonine protein kinase involved in DNA damage cell-cycle checkpoint; induced under Cdc5p depletion
orf19.3697	orf19.3697	7.28	7.46	9.54	8.37	0.102244333	NA	Ortholog(s) have role in ascospore formation, re-entry into mitotic cell cycle after pheromone arrest, regulation of meiosis I, regulation of mitosis and sporocarp development involved in sexual reproduction, more
orf19.3956	orf19.3956	14.77	7.45	13.91	9.36	0.019842883	NA	Ortholog(s) have glutaminyl-tRNA synthase (glutamine-hydrolyzing) activity and role in endoplasmic reticulum organization, glutaminyl-tRNAGln biosynthesis via transamidation
orf19.7621	orf19.7621	8.34	7.45	7.37	5.45	-0.088935416	NA	Putative subunit of an alternative replication factor C complex; role in DNA replication, genome integrity, homologous recombination-mediated repair and telomere homeostasis;
orf19.7102	orf19.7102	8.98	7.4	8.26	8.25	-0.003086868	NA	Has domain(s) with predicted exonuclease activity, nucleic acid binding activity and intracellular localization
orf19.3522	orf19.3522	9.43	7.4	8.28	11.41	0.075821951	NA	Protein of unknown function; flow model biofilm induced; transcript repressed by ciclopirox olamine
orf19.3455	orf19.3455	11.38	7.4	9.02	6.42	-0.082497872	NA	Putative mitochondrial inner membrane magnesium transporter; possibly an essential gene, disruptants not obtained by UAU1 method
orf19.3269	GSL2	6.55	7.4	11.09	11.31	0.203177738	NA	Protein similar to beta-1,3-glucan synthase; 16 predicted membrane-spanning regions; transcript regulated by Nrg1; very low gene expression in yeast-form and hyphal cells
orf19.587	orf19.587	8.79	7.38	10.33	6.42	-0.005066188	NA	Ortholog(s) have transcription export complex localization

orf19.3001	TEM1	14.75	7.37	15.67	9.27	0.063348228	NA	Protein similar to S. cerevisiae Tem1p, which is a GTPase involved in termination of the M phase of the cell cycle; induced under Cdc5p depletion
orf19.1105.3	orf19.1105.3	4.91	7.37	4.9	7.35	-0.003617304	NA	Protein of unknown function
orf19.6518	orf19.6518	10.07	7.36	7.16	6.51	-0.083601178	NA	Predicted aldehyde dehydrogenase [NAD(P)+]; Spider biofilm induced
orf19.4584	PHO114	9.67	7.35	12.22	10.86	0.145557773	NA	Acid phosphatase; induced by Mnl1 under weak acid stress; Spider biofilm induced
orf19.450	orf19.450	11.02	7.34	10.07	6.41	-0.056008262	NA	Protein of unknown function
orf19.3996	orf19.3996	10.42	7.33	11.16	7.18	0.021097678	NA	Putative mannosyltransferase; similar to S. cerevisiae Gpi10p; has HKEXRF motif
orf19.2007	orf19.2007	10.47	7.33	12.88	9.06	0.119791045	NA	Ortholog(s) have role in Golgi to vacuole transport, ascospore wall assembly, retrograde transport, endosome to Golgi and GARP complex, mitochondrion localization
orf19.2681	RBT7	4.63	7.33	5.39	7.12	-0.003617304	NA	Protein with similarity to RNase T2 enzymes; has putative secretion signal; expression is Tup1-repressed
orf19.816	DCK2	7.38	7.31	7.19	6.36	-0.032592915	NA	Protein similar to S. cerevisiae Ylr422wp; transposon mutation affects filamentous growth; induced by Mnl1p under weak acid stress

orf19.1706	MET18	9.56	7.31	7.18	6.67	-0.083601178	NA	Putative protein with a predicted role in nucleotide excision repair (NER) and RNA polymerase II (RNAP II) transcription; Plc1p-regulated
orf19.6148	orf19.6148	6.42	7.3	8.05	6.75	0.050883482	NA	Homolog of nuclear distribution factor NudE, NUDEL; regulates dynein targeting to microtubule plus ends; flow model biofilm repressed
orf19.3794	CSR1	9.09	7.29	7.47	6.97	-0.057672227	NA	Transcription factor; role in zinc homeostasis and regulation of Spider biofilm matrix; mutation affects filamentous growth; can suppress S. cerevisiae rok1 mutant inviability; Spider biofilm induced; mutants for abnormal Spider biofilms
orf19.1391	orf19.1391	9.59	7.28	9.18	3.82	-0.112175538	NA	Protein with similarity to trimeric LpxA-like enzymes; Hap43-repressed gene
orf19.7043	orf19.7043	10.74	7.28	6.12	6.88	-0.136188291	NA	Ortholog(s) have endoplasmic reticulum localization
orf19.5013	AGM1	8.53	7.27	15.08	10.54	0.239150094	NA	Phosphoacetylglucosamine mutase (N-acetylglucosamine-phosphate mutase); enzyme of UDP-N-acetylglucosamine (UDP-GlcNAc) biosynthesis
orf19.750	orf19.750	5.44	7.26	6.15	5.43	-0.034532074	NA	Protein of unknown function; exogenously expressed protein is a substrate for Kex2p processing in vitro
orf19.151	TPO5	7.36	7.26	9.08	6.14	0.02205381	NA	Putative polyamine transporter; mutation confers hypersensitivity to toxic ergosterol analog; hyphal induced; macrophage induced
orf19.4367	SMC1	5.77	7.25	8.21	5.9	0.022736542	NA	Protein similar to chromosomal ATPases; RNA abundance regulated by tyrosol and cell density; cell-cycle regulated periodic mRNA expression

orf19.5257	LCB4	10.51	7.24	13.16	7.8	0.069335184	NA	Putative sphingosine kinase; Tac1p-regulated expression; rat catheter biofilm induced
orf19.3210	orf19.3210	3.92	7.23	8.72	9.32	0.186692976	NA	Predicted protein of unknown function; Plc1-regulated
orf19.3954.1	orf19.3954.1	9.95	7.23	6.31	9.02	-0.054068692	NA	Ortholog of C. parapsilosis CDC317 : CPAR2_101170, Candida tenuis NRRL Y-1498 : CANTEDRAFT_113855, Debaryomyces hansenii CBS767 : DEHA2G20240g and Pichia stipitis Pignal : psti_CGOB_00174
orf19.6294	MYO1	6.79	7.22	8.71	7.73	0.076818911	NA	Component of actomyosin ring at neck of newly-emerged bud
orf19.2251	AAH1	8.3	7.21	3.96	5.03	-0.17786147	NA	Adenine deaminase; purine salvage and nitrogen catabolism; colony morphology-related regulation by Ssn6; Hog1, CO2-induced; chlamydospore formation repressed in C. albicans and C. dubliniensis; rat catheter and F-12/CO2 biofilm induced
orf19.4341	orf19.4341	9.65	7.2	14.24	10.13	0.166798772	NA	Ortholog(s) have role in attachment of GPI anchor to protein, conidium formation, hyphal growth, sporocarp development involved in sexual reproduction and GPI-anchor transamidase complex localization
orf19.1502	orf19.1502	8.94	7.2	9.16	6.68	-0.004089362	NA	Has domain(s) with predicted aminoacyl-tRNA hydrolase activity
orf19.4581	orf19.4581	3.45	7.2	6.56	7.18	0.108432154	NA	Putative glycosylphosphatidylinositol-alpha 1,4 mannosyltransferase I; regulated by Tsa1p, Tsa1Bp under H2O2 stress conditions
orf19.2417	SMC5	7.76	7.19	9.33	7.85	0.049580634	NA	Protein similar to S. cerevisiae Smc5p, which is involved in DNA repair; transposon mutation affects filamentous growth

orf19.1782.1	orf19.1782.1	7.18	7.18	2.8	4.36	-0.214742522	NA	Ortholog of C. parapsilosis CDC317 : CPAR2_407200.1, C. dubliniensis CD36 : Cd36_24070, Pichia stipitis Pignal : PICST_31669 and Spathaspora passalidarum NRRL Y-27907 : spas_CGOB_00103
orf19.5748	orf19.5748	7.18	7.18	7.79	3.11	-0.090869983	NA	Ortholog of C. dubliniensis CD36 : Cd36_64250, Candida tenuis NRRL Y-1498 : cten_CGOB_00218, Debaryomyces hansenii CBS767 : DEHA2E19888g and Spathaspora passalidarum NRRL Y-27907 : spas_CGOB_00025
orf19.3763	orf19.3763	7.3	7.17	8.5	6.2	0.02205381	NA	Has domain(s) with predicted serine-type endopeptidase activity and integral to membrane localization
orf19.4577	orf19.4577	6.77	7.16	8.8	7.03	0.049680792	NA	Ortholog(s) have GTPase activating protein binding, cAMP-dependent protein kinase inhibitor activity, signal transducer activity
orf19.7614	orf19.7614	9.37	7.16	7.51	6.41	-0.05859266	NA	Protein of unknown function; Hap43-induced gene; mutant is viable
orf19.5862	AFP99	5.21	7.14	7.13	5.19	-0.005802648	NA	Protein related to arginases; downregulated upon adherence to polystyrene; regulated by Gcn2p and Gcn4p
orf19.2082	SAP30	6	7.14	5.56	5.98	-0.033044633	NA	Aspartic-type endopeptidase; involved in degradation of alpha pheromone; functional equivalent of S. cerevisiae Bar1; a-cell specific; induced by alpha pheromone
orf19.998	orf19.998	10.72	7.14	9.62	6.88	-0.029507978	NA	Putative adapter protein; links synaptojanins Inp52 and Inp53 to the cortical actin cytoskeleton in S. cerevisiae; mutants are viable
orf19.3281	orf19.3281	3.89	7.13	6.21	5.82	0.023922301	NA	Ortholog(s) have histone demethylase activity (H3-K36 specific), methylated histone residue binding activity and role in histone demethylation

orf19.7578	orf19.7578	9.81	7.11	12.23	7.09	0.04571623	NA	Ortholog(s) have oligosaccharide binding activity, role in ER-associated ubiquitin-dependent protein catabolic process and Hrd1p ubiquitin ligase ERAD-L complex, luminal surveillance complex localization
orf19.4308	HSL1	7.43	7.1	10.95	7.79	0.127062615	NA	Probable protein kinase involved in determination of morphology during the cell cycle of both yeast-form and hyphal cells via regulation of Swe1p and Cdc28p; required for full virulence and kidney colonization in mouse systemic infection
orf19.6224	orf19.6224	12.84	7.1	6.84	4.35	-0.242850317	NA	RTA domain protein; predicted role in response to stress; Spider biofilm induced
orf19.6982	orf19.6982	7.68	7.1	6.94	5.35	-0.088935416	NA	Ortholog(s) have ribosome binding activity and mitochondrion localization
orf19.5548	LYS14	12	7.09	9.26	5.61	-0.106990119	NA	Zn(II)2Cys6 transcription factor; has similarity to S. cerevisiae Lys14, which is a transcription factor involved in the regulation of lysine biosynthesis genes
orf19.6755	DLD2	9.4	7.08	12.27	10.99	0.172137842	NA	Ortholog(s) have D-lactate dehydrogenase (cytochrome) activity, role in aerobic respiration, cellular carbohydrate metabolic process and mitochondrial inner membrane localization
orf19.3009	orf19.3009	6.96	7.06	8.73	6.47	0.02205381	NA	Ortholog of S. cerevisiae : YDR179W-A, C. glabrata CBS138 : CAGL0F05819g, C. dubliniensis CD36 : Cd36_02970, C. parapsilosis CDC317 : CPAR2_108540 and Candida tenuis NRRL Y-1498 : CANTEDRAFT_132957
orf19.4250	orf19.4250	8.18	7.06	14.83	14.45	0.335313238	NA	Ortholog of C. dubliniensis CD36 : Cd36_52200, C. parapsilosis CDC317 : CPAR2_502480, Candida tenuis NRRL Y-1498 : CANTEDRAFT_115972 and Debaryomyces hansenii CBS767 : DEHA2G19976g
orf19.972	orf19.972	6.83	7.05	9.01	8.35	0.076818911	NA	Ortholog(s) have 5'-flap endonuclease activity, role in DNA-dependent DNA replication and Slx1-Slx4 complex localization

orf19.7472	IFF4	7.56	7.04	7.74	6.38	-0.032063392	NA	Adhesin-like cell surface protein; putative GPI-anchor; null mutant germ tubes show decreased adhesion to plastic substrate; mutants are viable; Hap43-repressed gene
orf19.3976	orf19.3976	8.37	7.03	8.18	8.51	0.050521753	NA	Ortholog(s) have structural constituent of cytoskeleton activity, role in establishment of mitotic spindle orientation, nuclear migration and astral microtubule, cell cortex, dynactin complex, spindle pole body localization
orf19.1948	orf19.1948	10.34	7.03	13.03	10.74	0.168352552	NA	Ortholog of C. dubliniensis CD36 : Cd36_51110, C. parapsilosis CDC317 : CPAR2_302560, Candida tenuis NRRL Y-1498 : CANTEDRAFT_135271 and Debaryomyces hansenii CBS767 : DEHA2E12914g
orf19.6510	GRX1	5.95	7.03	11.33	8.63	0.180613063	NA	Putative glutaredoxin; induced in low iron
orf19.5506	PLC1	9.07	7.01	8.82	7.1	-0.004089362	NA	Phosphoinositide-specific phospholipase C (PI-PLC), delta-form; essential; roles in stress resistance, hyphal growth; upregulated in biofilm; predicted N-linked glycosylation; lacks conserved EF-hand domain
orf19.5270	orf19.5270	8.84	7	18.19	9.18	0.254060321	NA	Protein of unknown function; rat catheter biofilm induced
orf19.832	GPI13	7.67	7	6.86	7.88	-0.002963425	NA	Major facilitator superfamily protein; has phosphodiesterase/nucleotide pyrophosphatase domain; similar to S. cerevisiae Gpi13p, which acts in GPI anchor biosynthesis; Hap43p-induced gene
orf19.2581	orf19.2581	8.14	7	6.04	8.12	-0.029903158	NA	Protein with a predicted epimerase/dehydratase domain; Hap43-repressed gene
orf19.5041	orf19.5041	9.46	7	10.31	9.25	0.074386227	NA	Ortholog of C. dubliniensis CD36 : Cd36_43600, C. parapsilosis CDC317 : CPAR2_403780, Candida tenuis NRRL Y-1498 : CANTEDRAFT_93767 and Debaryomyces hansenii CBS767 : DEHA2G16984g

orf19.5821	orf19.5821	8.59	6.99	10.11	8.1	0.048326625	NA	Ortholog of S. cerevisiae : YML002W, C. glabrata CBS138 : CAGL0L07634g, C. dubliniensis CD36 : Cd36_17530, C. parapsilosis CDC317 : CPAR2_212430 and Candida tenuis NRRL Y-1498 : CANTEDRAFT_128807
orf19.2280	ZCF10	9.32	6.99	8.3	5.64	-0.05859266	NA	Putative transcription factor with zinc cluster DNA-binding motif
orf19.5852	orf19.5852	6.85	6.99	10.81	8.92	0.152995041	NA	Protein of unknown function; rat catheter biofilm repressed
orf19.6913	GCN2	8.57	6.98	9.79	7.75	0.048326625	NA	Translation initiation factor 2-alpha (eIF2alpha) kinase; has nonessential role in amino acid starvation response, in contrast to S. cerevisiae homolog; similar to S. cerevisiae Gcn2p
orf19.7503	CDA2	5.11	6.98	4.92	6.28	-0.033384618	NA	Putative chitin deacetylase; transcription is positively regulated by Tbf1p
orf19.6307	orf19.6307	10.62	6.98	9.92	8.71	0.022532146	NA	Ortholog of C. dubliniensis CD36 : Cd36_30250, Candida tenuis NRRL Y-1498 : CANTEDRAFT_98619, Debaryomyces hansenii CBS767 : DEHA2G22594g and Pichia stipitis Pignal : PICST_50168
orf19.5094	BUL1	7.89	6.97	10.27	10.59	0.151793746	NA	Protein similar but not orthologous to S. cerevisiae Bul1; a protein involved in selection of substrates for ubiquitination; mutants are viable; macrophage/pseudohyphal-induced; rat catheter biofilm induced
orf19.1258	orf19.1258	8.58	6.97	5.62	2.67	-0.204996612	NA	Adhesin-like protein; regulated by Tsa1, Tsa1B in minimal media at 37 deg; clade-associated gene expression; induced by alpha pheromone in SpiderM medium; Hap43-induced; Spider biofilm repressed
orf19.2203.1	orf19.2203.1	2.79	6.96	4.86	3.47	-0.067422971	NA	Ortholog of C. parapsilosis CDC317 : CPAR2_103590, Debaryomyces hansenii CBS767 : DEHA2C08118g, Pichia stipitis Pignal : psti_CGOB_00124 and Spathaspora passalidarum NRRL Y-27907 : SPAPADRAFT_49916

orf19.9	orf19.9	6.14	6.95	6.29	6.44	-0.033044633	NA	Ortholog of C. dubliniensis CD36 : Cd36_32700, C. parapsilosis CDC317 : CPAR2_204280, Candida tenuis NRRL Y-1498 : CANTEDRAFT_113831 and Debaryomyces hansenii CBS767 : DEHA2E03916g
orf19.6569	orf19.6569	10.9	6.93	8.4	7.41	-0.081878719	NA	Predicted transmembrane transporter; induced during chlamydospore formation in both C. albicans and C. dubliniensis
orf19.7148	TPO2	8.34	6.91	9.09	7.77	0.049580634	NA	Putative polyamine transport protein; fungal-specific (no human or murine homolog)
orf19.359	GTT12	4.01	6.9	3.19	5.55	-0.062729615	NA	Ortholog(s) have glutathione peroxidase activity, glutathione transferase activity, role in glutathione metabolic process and endoplasmic reticulum, mitochondrial outer membrane, plasma membrane localization
orf19.853	SAP99	4.65	6.88	4.81	5.32	-0.063941467	NA	Putative secreted aspartyl protease; possible Kex2 substrate; induced by low iron; repressed by alpha pheromone in SpiderM medium; possibly essential (UAU1 method); regulated by Sef1, Sfu1, and Hap43; Spider biofilm induced
orf19.3607	orf19.3607	14.16	6.88	12.8	9.89	0.044250385	NA	Ortholog of C. dubliniensis CD36 : Cd36_22860, C. parapsilosis CDC317 : CPAR2_806540, Candida tenuis NRRL Y-1498 : CANTEDRAFT_108823 and Debaryomyces hansenii CBS767 : DEHA2B13596g
orf19.3764	GSG1	8.43	6.88	9.78	7.35	0.048397775	NA	Putative subunit of the TRAPP complex; involved in targeting of ER-to-Golgi transport vesicles; flow model biofilm induced
orf19.4433	CPH1	6.39	6.86	8.62	9.46	0.131035575	NA	Transcription factor; for mating, filamentation on solid media, pheromone-stimulated biofilms; in pathway with Ess1, Czf1; required with Efg1 for host cytokine response; regulates galactose metabolism genes; rat cathether biofilm repressed
orf19.1939	orf19.1939	6.7	6.86	13.53	15.4	0.363671322	NA	Ortholog of S. cerevisiae : YDR374C, C. glabrata CBS138 : CAGL0A02970g, C. dubliniensis CD36 : Cd36_51030, C. parapsilosis CDC317 : CPAR2_303320 and Candida tenuis NRRL Y-1498 : CANTEDRAFT_106457

orf19.5268	MED10	9.53	6.86	7.6	7.6	-0.029115391	NA	RNA polymerase II mediator complex subunit; possibly an essential gene, disruptants not obtained by UAU1 method
orf19.6977	GPI1	6.94	6.85	7.4	5.97	-0.032592915	NA	Putative protein of GPI synthesis; expression is reduced in a fluconazole-resistant isolate
orf19.4374	PRP42	10.31	6.82	14.71	9.49	0.164689646	NA	Putative component of the U1 snRNP, involved in splicing; ortholog of S. cerevisiae PRP42
orf19.6315	orf19.6315	8.17	6.81	11.2	9.16	0.125703476	NA	Ortholog of C. dubliniensis CD36 : Cd36_30140, C. parapsilosis CDC317 : CPAR2_204040, Candida tenuis NRRL Y-1498 : CANTEDRAFT_114703 and Debaryomyces hansenii CBS767 : DEHA2G22880g
orf19.5236	orf19.5236	8.2	6.8	12.77	8.17	0.147707253	NA	Ortholog(s) have dehydrodolichyl diphosphate synthase activity, role in protein glycosylation and lipid particle localization
orf19.4151	SPO1	5.54	6.78	7.34	8.49	0.05185997	NA	Protein similar to phospholipase B; fungal-specific (no human or murine homolog)
orf19.4749	orf19.4749	8.86	6.76	13.04	10.71	0.195004091	NA	Protein of unknown function; hyphal-induced expression, regulated by Cyr1, Ras1, Efg1; Hap43-induced gene; Spider biofilm induced
orf19.1264	CFL2	5.78	6.75	3.88	3.86	-0.154998519	NA	Oxidoreductase; iron utilization; Sfu1/Sef1/Hap43/Nrg1/Tup1/Rim101 regulated; alkaline/low iron/fluphenazine/ciclopirox olamine, flucytosine, fluconazole, Spider/flow model/rat catheter biofilm induced; caspofungin/amphotericin B repressed
orf19.1798	TSC2	7.49	6.75	7.6	5.96	-0.004926416	NA	Putative GTPase-activating protein; similar to mammalian tuberin; involved in control of filamentous growth; mutants are viable

orf19.4675	ASK1	6.24	6.75	8.09	8.5	0.105358624	NA	Essential subunit of the Dam1 (DASH) complex, which acts in chromosome segregation by coupling kinetochores to spindle microtubules
orf19.3432.1	orf19.3432.1	17.96	6.73	2.24	6.71	-0.39371417	NA	Ortholog(s) have enzyme regulator activity, role in cation transport and plasma membrane localization
orf19.5926	ARG11	6.34	6.73	9.28	9.28	0.131035575	NA	Putative ornithine transporter of the mitochondrial inner membrane; induced during the mating process
orf19.3828	orf19.3828	4.28	6.73	5.49	4.27	-0.065872209	NA	Ortholog of Candida albicans WO-1 : CAWG_03357
orf19.3254	orf19.3254	9.88	6.72	10.06	9.95	0.073603775	NA	Ortholog of C. dubliniensis CD36 : Cd36_26020, Candida tropicalis MYA-3404 : CTRG_00984 and Candida albicans WO-1 : CAWG_01455
orf19.7342	AXL1	7.5	6.71	5.55	5.12	-0.090837501	NA	Putative endoprotease; induced by alpha factor; transcript is upregulated in an RHE model of oral candidiasis and in clinical isolates from HIV+ patients with oral candidiasis
orf19.728	TSC11	9.36	6.69	8.29	5.59	-0.05859266	NA	Protein similar to S. cerevisiae Tsc11p which is involved in sphingolipid biosynthesis; transposon mutation affects filamentous growth
orf19.87	GPX1	12.51	6.67	9.43	10.25	-0.025770452	NA	Putative thiol peroxidase; rat catheter and Spider biofilm induced
orf19.5004	RAD54	7.82	6.66	11.41	12.2	0.199926925	NA	Putative DNA-dependent ATPase involved in DNA repair; induced under hydroxyurea treatment; plays an essential role during mitotic growth; mutants display aberrant cell and nuclear morphology

orf19.4266	SPR28	6.66	6.66	6.17	5.85	-0.060868785	NA	Septin; similar to S. cerevisiae meiotic/sporulation septin; mutant has no obvious phenotype; two introns with noncanonical branch site and 5' splice site, respectively; splicing inhibited upon exposure to alpha-factor
orf19.5498	EFH1	6.5	6.66	6.31	7.67	-0.002823317	NA	APSES transcription factor; homodimer; minor role in transcriptional regulation vs Efg1; regulates filamentous growth, phenotypic switch; EFG1 and EFH1 genetically interact; expression interferes with mouse intestinal tract colonization
orf19.4548	MAK32	10.13	6.64	7.29	6.79	-0.083601178	NA	Putative protein involved in the structural stability of L-A double-stranded RNA-containing particles; downregulated upon adherence to polystyrene
orf19.6610	orf19.6610	6.44	6.64	7.15	6.95	0.023838184	NA	Ortholog(s) have microtubule plus-end binding, structural constituent of cytoskeleton activity
orf19.927	orf19.927	5.37	6.63	5.04	4.09	-0.095128535	NA	Ortholog(s) have role in positive regulation of transcription from RNA polymerase II promoter, telomere maintenance and EKC/KEOPS complex, cytosol, nucleus localization
orf19.4478	orf19.4478	10.85	6.61	12.76	9.06	0.094027432	NA	Ortholog(s) have aspartate-tRNA ligase activity, role in mitochondrial aspartyl-tRNA aminoacylation and mitochondrion localization
orf19.6115	orf19.6115	7.8	6.6	7.18	5.98	-0.059788163	NA	Dubious open reading frame
orf19.3277	orf19.3277	4.2	6.6	6.58	4.19	-0.006784916	NA	Ortholog of Candida albicans WO-1 : CAWG_01437
orf19.4786	FGR43	4.02	6.58	6.34	5.45	-0.005707629	NA	Protein lacking an S. cerevisiae ortholog; transposon mutation affects filamentous growth; induced by by Mnl1 under weak acid stress

orf19.2810	AAP1	8.81	6.58	15.06	16.31	0.350919708	NA	Putative amino acid permease; fungal-specific; possibly essential, disruptants not obtained by UAU1 method; Spider biofilm induced
orf19.2963	orf19.2963	10.89	6.57	12.29	13.31	0.166409326	NA	Has domain(s) with predicted NAD+ binding activity
orf19.2343	VPS23	7.08	6.56	9.37	9.36	0.103362972	NA	ESCRT I protein sorting complex protein; involved in proteolytic activation of Rim101, which regulates pH response; ortholog of S. cerevisiae Stp22/Vps23
orf19.1283	MEC1	6.99	6.52	9.38	8.46	0.076818911	NA	Cell cycle checkpoint protein with a role in genome integrity; RNA abundance regulated by tyrosol and cell density
orf19.7100	orf19.7100	6.09	6.51	8.59	7.43	0.077300315	NA	Putative vacuolar transporter of large neutral amino acids; possibly transcriptionally regulated upon hyphal formation; not the true Avt1 ortholog of S. cerevisiae
orf19.2446	orf19.2446	9.31	6.5	13.66	10.51	0.2173437	NA	Ortholog(s) have cell division site, cell tip, cytoplasm, nucleus localization
orf19.1063	orf19.1063	5.06	6.48	4.42	6.15	-0.03266929	NA	Ortholog(s) have dolichyl-phosphate-mannose-glycolipid alpha-mannosyltransferase activity, role in GPI anchor biosynthetic process and integral to endoplasmic reticulum membrane, mannosyltransferase complex, mitochondrion localization
orf19.7414	ALS6	5.72	6.46	10.01	8.61	0.186056387	NA	ALS family protein; expression in S. cerevisiae confers adhesion to gelatin; macrophage-induced gene; N-terminal adhesion domain; ALS family includes cell-surface glycoproteins, some with adhesin function
orf19.4958	ECM25	6.45	6.44	4.95	5.54	-0.032353114	NA	Non-essential protein involved in cell morphogenesis

orf19.6782	BMT1	6.39	6.39	2.96	2.6	-0.19141836	NA	Beta-mannosyltransferase, required for addition of the 1st beta-mannose residue to acid-stable fraction of cell wall phosphopeptidomannan; 9-gene family member; mutants induce higher levels of inflammatory cytokines in mouse dendritic cells
orf19.952	orf19.952	8.11	6.39	8.63	6.92	0.050723065	NA	Has domain(s) with predicted DNA binding activity
orf19.2168	orf19.2168	10.42	6.39	6.93	7.76	-0.028222226	NA	Putative sterol deacetylase; flow model biofilm induced; rat catheter biofilm repressed
orf19.4520	orf19.4520	7.74	6.39	6.38	8.39	-0.001668905	NA	Putative gluconokinase; rat catheter biofilm induced
orf19.4691	MRV1	9.57	6.38	2.86	7.63	-0.134903936	NA	Ortholog of Candida albicans WO-1 : CAWG_03682
orf19.4557	orf19.4557	7.39	6.38	9.99	9.26	0.157468255	NA	Ortholog(s) have microtubule binding, structural constituent of cytoskeleton activity and role in microtubule nucleation, mitotic spindle assembly checkpoint, protein localization to kinetochore, sister chromatid biorientation
orf19.1476	orf19.1476	6.15	6.38	7.84	4.88	0.023468695	NA	Ortholog(s) have mRNA (N6-adenosine)-methyltransferase activity, role in mRNA methylation, meiosis, negative regulation of pseudohyphal growth and MIS complex localization
orf19.1808	MED5	9.1	6.38	8.9	7.66	0.050477784	NA	RNA polymerase II mediator complex subunit; transcription positively regulated by Tbf1p
orf19.1009	orf19.1009	10.84	6.38	4.45	8.9	-0.104156265	NA	Ortholog of Candida albicans WO-1 : CAWG_01857

orf19.3444	orf19.3444	8.02	6.38	4.09	3.45	-0.214592329	NA	Predicted membrane transporter, member of the drug:proton antiporter (14 spanner) (DHA2) family, major facilitator superfamily (MFS); Hap43p-repressed gene
orf19.5931	ARV1	8.29	6.38	5.94	7.84	-0.001668905	NA	Lipid transporter involved in sterol trafficking and transport of glycosylphosphatidylinositol and sphingolipid precursors
orf19.3231	CDC27	7.58	6.38	6.79	5.17	-0.060510313	NA	Putative ubiquitin-protein ligase; periodic mRNA expression, peak at cell-cycle S/G2 phase
orf19.5579	orf19.5579	6.37	6.36	6.82	7.76	0.081468149	NA	Protein with a predicted double-strand break repair domain; Hap43-repressed gene
orf19.5527	orf19.5527	7.61	6.36	11.81	9.2	0.177710328	NA	Protein with a predicted role in 5.8S rRNA processing; flow model biofilm induced
orf19.4227	orf19.4227	4.29	6.35	7.02	6.85	0.111452726	NA	Ortholog of C. parapsilosis CDC317 : CPAR2_100400, C. dubliniensis CD36 : Cd36_52020, Debaryomyces hansenii CBS767 : DEHA2F14278g and Candida orthopsilosis Co 90-125 : CORT_0F00500
orf19.2553	orf19.2553	11.27	6.34	12.65	9.66	0.144062223	NA	Protein similar to S. cerevisiae Pmr1p; amphotericin B induced; previously merged with orf19.2552 in Assembly 20; unmerged from orf19.2552 in a revision of Assembly 21
orf19.2370	DSL1	9.15	6.34	10.48	7.68	0.075794277	NA	Protein similar to S. cerevisiae Dsl1p, which is a member of the t-SNARE complex of the endoplasmic reticulum
orf19.5792	SMP3	5.72	6.34	7.07	9.06	0.108769145	NA	Mannosyltransferase of glycosylphosphatidylinositol (GPI) biosynthesis; catalyzes mannosylation of Man3-GPI precursor; essential for viability; 8-9 transmembrane regions predicted; has HQEXRF motif; functional homolog of S. cerevisiae Smp3p

orf19.6936	RAD53	9.34	6.34	9.66	7.46	0.049285101	NA	Protein involved in regulation of DNA-damage-induced filamentous growth; putative component of cell cycle checkpoint; ortholog of S. cerevisiae Rad53p, protein kinase required for cell-cycle arrest in response to DNA damage
orf19.5046	RAM1	8.34	6.33	7.69	5.47	-0.032251642	NA	Protein that acts in prenylation; transcription is alpha-factor induced; regulated in response to lovastatin and fluconazole; Hap43p-repressed gene
orf19.6353	orf19.6353	8.45	6.32	11	10.02	0.179165662	NA	Ortholog of Candida albicans WO-1 : CAWG_00172
orf19.1447	orf19.1447	6.44	6.32	9.4	9.17	0.160905873	NA	Ortholog of C. dubliniensis CD36 : Cd36_16290, C. parapsilosis CDC317 : CPAR2_214050, Candida tenuis NRRL Y-1498 : CANTEDRAFT_92180 and Debaryomyces hansenii CBS767 : DEHA2A01738g
orf19.6456	orf19.6456	8.86	6.31	12.04	8.17	0.124621145	NA	Ortholog of C. dubliniensis CD36 : Cd36_72280, C. parapsilosis CDC317 : CPAR2_703040, Candida tenuis NRRL Y-1498 : CANTEDRAFT_125842 and Debaryomyces hansenii CBS767 : DEHA2B05984g
orf19.3284	orf19.3284	6.31	6.31	2.52	6.29	-0.090848613	NA	Ortholog of Candida albicans WO-1 : CAWG_01431
orf19.2558	orf19.2558	7.59	6.3	5.52	5.64	-0.059589727	NA	Has domain(s) with predicted zinc ion binding activity and nucleus localization
orf19.5258	orf19.5258	8.38	6.3	9.83	8.64	0.129352263	NA	Protein of unknown function; induced by nitric oxide
orf19.1608	orf19.1608	5.57	6.3	9.53	7.75	0.159703059	NA	Ortholog(s) have cytosol, nucleus localization

orf19.184	MDM10	11.41	6.29	9.16	9.94	0.049702065	NA	Predicted component of the mitochondrial sorting and assembly machinery (SAM complex) and ER-mitochondria encounter structure (ERMES) complex with a role in protein import into mitochondria
orf19.6704	orf19.6704	3.39	6.28	4.98	7.43	0.084871497	NA	Protein of unknown function; Hap43-repressed gene
orf19.4664	NAT4	7.22	6.28	6.26	5.63	-0.031908479	NA	Putative histone acetyltransferase; involved in regulation of white-opaque switch; early-stage flow model biofilm induced; Spider biofilm induced
orf19.1429	SOH1	8.53	6.28	10.3	10.29	0.127306672	NA	Subunit of the RNA polymerase II mediator complex; involved in control of cytokinesis, morphogenesis, biofilm formation, virulence; cell-cycle regulated periodic mRNA expression
orf19.1096	orf19.1096	9.01	6.28	7.31	7.66	-0.001820252	NA	Has domain(s) with predicted voltage-gated chloride channel activity, role in chloride transport, transmembrane transport and membrane localization
orf19.2399	orf19.2399	7.76	6.27	6.26	6.15	-0.059589727	NA	Putative transcription factor with zinc finger DNA-binding motif; similar to bacterial DnaJ; induced in low iron
orf19.4743	AFG1	7.97	6.26	9.46	10.08	0.130185669	NA	Putative mitochondrial ATPase of the AAA family; similar but not orthologous to S. cerevisiae Afg1; mutant is viable; regulated by Sef1, Sfu1, and Hap43
orf19.725	orf19.725	5.05	6.25	7.91	5.51	0.081063342	NA	Ortholog of C. dubliniensis CD36 : Cd36_31880, C. parapsilosis CDC317 : CPAR2_702220, Candida tenuis NRRL Y-1498 : CANTEDRAFT_115848 and Debaryomyces hansenii CBS767 : DEHA2E06028g
orf19.1309	orf19.1309	9.14	6.25	6.72	4.79	-0.08737864	NA	Ortholog of C. dubliniensis CD36 : Cd36_43290, C. parapsilosis CDC317 : CPAR2_403630, Candida tenuis NRRL Y-1498 : CANTEDRAFT_114295 and Debaryomyces hansenii CBS767 : DEHA2G17336g

orf19.3432	orf19.3432	9.06	6.23	6.55	3.27	-0.145693549	NA	Predicted membrane transporter, member of the drug:proton antiporter (12 spanner) (DHA1) family, major facilitator superfamily (MFS); induced by nitric oxide
orf19.4821	LIP1	4.91	6.23	4.5	3.3	-0.096970593	NA	Secreted lipase, member of a lipase gene family whose members are expressed differentially in response to carbon source and during infection; may have a role in nutrition and/or in creating an acidic microenvironment
orf19.4515	orf19.4515	5.49	6.21	6.56	7.82	0.110293231	NA	Predicted protein with similarity to cell wall proteins; possibly an essential gene, disruptants not obtained by UAU1 method
orf19.5503	orf19.5503	1.86	6.18	3.08	4.31	-0.036022963	NA	Putative protein of unknown function; transcript is upregulated in clinical isolates from HIV+ patients with oral candidiasis; Spider biofilm induced
orf19.4467	orf19.4467	8.39	6.17	9.72	8	0.103179653	NA	Ortholog of C. dubliniensis CD36 : Cd36_03660, C. parapsilosis CDC317 : CPAR2_106860, Candida tenuis NRRL Y-1498 : CANTEDRAFT_96694 and Debaryomyces hansenii CBS767 : DEHA2G15950g
orf19.1234	FGR6-10	7.65	6.16	9.64	10.15	0.154945178	NA	Protein lacking an ortholog in S. cerevisiae; member of a family encoded by FGR6-related genes in the RB2 repeat sequence; transposon mutation affects filamentous growth
orf19.2853	orf19.2853	9.66	6.15	7.88	7.44	-0.029279158	NA	Protein of unknown function; rat catheter biofilm induced
orf19.1116	orf19.1116	4.61	6.15	9.51	10.12	0.240713313	NA	Protein of unknown function; planktonic growth-induced gene
orf19.3440	FRP5	2.51	6.15	1.82	3.18	-0.133235925	NA	Protein with a GPR1/FUN34/yaaH family domain; flucytosine repressed

orf19.2903	AGO1	6.56	6.15	13.94	12.34	0.325137561	NA	Putative Argonaute protein involved in RNA silencing; hyphal-induced expression; regulated by Cyr1, Ras1, Efg1, Nrg1, Tup1; rat catheter biofilm repressed
orf19.6880	COG4	6.07	6.14	9.09	7.09	0.106958301	NA	Ortholog(s) have role in CVT pathway, macroautophagy, peroxisome degradation, retrograde transport, vesicle recycling within Golgi and Golgi transport complex, cell division site, cell tip, nucleus localization
orf19.1207	orf19.1207	7.35	6.12	4.88	4.88	-0.091443114	NA	Ortholog of C. dubliniensis CD36 : Cd36_33200 and Candida albicans WO-1 : CAWG_03416
orf19.4701	orf19.4701	5.51	6.12	9.56	6.51	0.132704137	NA	Ortholog(s) have ATPase activity, tRNA binding activity, role in protein urmylation, regulation of transcription from RNA polymerase II promoter, tRNA wobble uridine modification and Elongator holoenzyme complex localization
orf19.5316	FGR29	5.47	6.1	9.56	8.05	0.18853009	NA	Protein lacking an ortholog in S. cerevisiae; transposon mutation affects filamentous growth; rat catheter biofilm repressed
orf19.6240	orf19.6240	7.75	6.1	10.31	8.31	0.103179653	NA	SH3-domain protein; role in barrier septum assembly involved in cell cycle cytokinesis; activates the Chs2 chitin synthase activity during cytokinesis; Spider biofilm repressed
orf19.2893	orf19.2893	7.63	6.1	7.46	5.58	-0.031376047	NA	Protein of unknown function; regulation correlates with clinical development of fluconazole resistance; transcript is upregulated in an RHE model of oral candidiasis
orf19.1238	TUB4	5.85	6.1	7.32	5.33	-0.004628914	NA	Putative gamma-tubulin; induced upon adherence to polystyrene; transcript regulated by Nrg1 and Mig1; periodic mRNA expression, peak at cell-cycle S/G2 phase
orf19.1002	orf19.1002	8.32	6.07	8.79	8.49	0.077806793	NA	Protein of unknown function; Hap43-repressed gene

orf19.5725	orf19.5725	7.55	6.04	8.06	3.37	-0.089539362	NA	Ortholog(s) have guanyl-nucleotide exchange factor activity, role in autophagic vacuole fusion, protein targeting to vacuole, vesicle docking and Mon1-Ccz1 complex localization
orf19.2919	MPH1	5.67	6.03	5.55	4.78	-0.033501931	NA	Protein similar to S. cerevisiae Mph1p, which is a DNA helicase involved in DNA repair; induced under hydroxyurea treatment
orf19.2927	MNN11	10.64	6.02	8.1	8.65	-0.001088303	NA	Ortholog(s) have alpha-1,2-galactosyltransferase activity, alpha-1,6-mannosyltransferase activity
orf19.5585	SAP5	2.73	6.02	35.74	19.08	0.965655712	NA	Secreted aspartyl proteinase; sap4,5,6 triple null defective in utilization of protein as N source; virulence role effected by URA3; expressed during infection; mRNA localized to hyphal tip via She3; rat catheter and Spider biofilm induced
orf19.3581	orf19.3581	8.29	6	7.84	4.56	-0.032251642	NA	Ortholog(s) have histone binding activity, role in DNA replication-dependent nucleosome assembly and CAF-1 complex, cytoplasm, nucleus localization
orf19.4690	orf19.4690	5.25	5.99	2.78	4.16	-0.127721685	NA	NRAMP metal ion transporter domain-containing protein; induced by nitric oxide independent of Yhb1; flow model biofilm induced; rat catheter biofilm repressed
orf19.5210	orf19.5210	5.25	5.99	9.5	8.65	0.189776218	NA	Putative Xbp1 transcriptional repressor; binds to cyclin gene promoters in S. cerevisiae; Hap43-repressed; possibly essential, disruptants not obtained by UAU1 method
orf19.2401	orf19.2401	9.33	5.99	9.45	9.03	0.076871503	NA	Ortholog(s) have cysteine-type endopeptidase activity
orf19.486	NIP100	5.35	5.96	9.64	9.7	0.240713313	NA	p150 subunit of dynactin; required for normal spindle formation and position

orf19.4136	YBL053	7.7	5.95	8.45	6.75	0.02429666	NA	Ortholog(s) have protein heterodimerization activity
orf19.2397	orf19.2397	7.79	5.93	6.54	7.15	-0.00273026	NA	Predicted membrane transporter, member of the anion:cation symporter (ACS) family, major facilitator superfamily (MFS); amphotericin B, caspofungin repressed; Hap43p-repressed
orf19.6304	LYS5	8	5.91	4.37	2.28	-0.247325509	NA	Phosphopantetheinyl transferase; enzyme of lysine biosynthesis; modifies, and thereby activates, Lys2p alpha-aminoadipate reductase; functional homolog of S. cerevisiae Lys5p
orf19.3744	orf19.3744	9.08	5.86	10.62	10.12	0.151417669	NA	Ortholog(s) have tRNA binding activity, role in tRNA export from nucleus and cytoplasm, nuclear pore localization
orf19.3551	DAD2	3.72	5.85	9.01	3.18	0.049688585	NA	Essential subunit of the Dam1 (DASH) complex, which acts in chromosome segregation by coupling kinetochores to spindle microtubules
orf19.7112	FRP2	5.95	5.84	8.01	9.15	0.135115228	NA	Putative ferric reductase; alkaline induced by Rim101; fluconazole-downregulated; upregulated in the presence of human neutrophils; possibly adherence-induced; regulated by Sef1, Sfu1, and Hap43
orf19.3248	orf19.3248	4.33	5.83	4.5	4.69	-0.004406231	NA	Ortholog of C. dubliniensis CD36 : Cd36_26070, C. parapsilosis CDC317 : CPAR2_803220, Candida tenuis NRRL Y-1498 : CANTEDRAFT_131795 and Debaryomyces hansenii CBS767 : DEHA2E12694g
orf19.3851	orf19.3851	10.49	5.82	12.29	8.96	0.123017419	NA	Protein of unknown function; induced by alpha pheromone in SpiderM medium
orf19.656	DPP1	9.21	5.82	6.77	5.56	-0.058371215	NA	Putative diacylglycerol pyrophosphate phosphatase of diacylglycerol production for phospholipid biosynthesis; downregulation correlates with clinical development of fluconazole resistance

orf19.644	HGT9	5.18	5.82	2.37	1.07	-0.268157752	NA	Putative glucose transporter of the major facilitator superfamily; the C. albicans glucose transporter family comprises 20 members; 12 probable membrane-spanning segments; induced at low (0.2%, compared to 2%) glucose in rich media; intron
orf19.6310	orf19.6310	6.63	5.79	7.53	6	0.02393455	NA	Has domain(s) with predicted myosin binding activity
orf19.2748	ARG83	7.11	5.79	8.16	6.71	0.052114387	NA	GAL4-like Zn(II)2Cys6 transcription factor; clade-associated expression; null shows abnormal regulation of invasive colony growth, is unable to utilize proline as a nitrogen source; flow model biofilm induced
orf19.3550	SGO1	8.13	5.78	8.24	4.81	-0.032251642	NA	Component of the spindle checkpoint; cell-cycle regulated periodic mRNA expression
orf19.3142	orf19.3142	6.25	5.78	8.1	8.41	0.108159406	NA	Ortholog of C. dubliniensis CD36 : Cd36_46140, C. parapsilosis CDC317 : CPAR2_501210, Candida tenuis NRRL Y-1498 : CANTEDRAFT_97195 and Debaryomyces hansenii CBS767 : DEHA2E08800g
orf19.1039	BET4	6.62	5.77	5.42	9.48	0.026755241	NA	Ortholog(s) have Rab geranylgeranyltransferase activity and role in ER to Golgi vesicle-mediated transport, protein geranylgeranylation, protein targeting to membrane
orf19.7596	orf19.7596	4.03	5.75	2.3	8.22	-0.000889412	NA	Protein with a phosphoglycerate mutase family domain; Hap43-repressed gene
orf19.5043	orf19.5043	5.97	5.72	7.35	6.21	0.024687213	NA	Ortholog of C. dubliniensis CD36 : Cd36_43610, C. parapsilosis CDC317 : CPAR2_403770, Debaryomyces hansenii CBS767 : DEHA2G16962g and Pichia stipitis Pignal : PICST_30926
orf19.5145	SSP96	6	5.7	10.32	7.03	0.132704137	NA	Putative flavin-containing monooxygenase; F-12/CO2 early biofilm induced

orf19.5729	FGR17	7.35	5.65	6.3	5.64	-0.031908479	NA	Putative DNA-binding transcription factor; has zinc cluster DNA-binding motif; lacks an ortholog in S. cerevisiae; transposon mutation affects filamentous growth; Hap43p-repressed gene
orf19.1334	orf19.1334	5.09	5.65	7.99	6.11	0.081063342	NA	Protein of unknown function; repressed by alpha pheromone in SpiderM medium
orf19.2910	PGA43	7.27	5.65	11.01	6.71	0.129417829	NA	Putative GPI-anchored protein
orf19.5986	THI4	5.48	5.65	8.46	4.76	0.052180709	NA	Thiamine biosynthetic enzyme precursor; repressed during the mating process; stationary phase enriched protein; Spider biofilm induced
orf19.69.2	orf19.69.2	1.41	5.64	0.7	5.62	-0.002602911	NA	Dubious open reading frame
orf19.6802	orf19.6802	6.95	5.63	5.94	5.45	-0.06185335	NA	Ortholog(s) have palmitoyltransferase activity, role in protein palmitoylation, vacuole fusion, non-autophagic and Golgi apparatus, endoplasmic reticulum, fungal-type vacuole membrane localization
orf19.3610	orf19.3610	4.96	5.63	5.62	3.9	-0.035012404	NA	Protein of unknown function; upregulation correlates with clinical development of fluconazole resistance; regulated by Sef1, Sfu1, and Hap43
orf19.576	CTF8	8.83	5.62	10.01	10.4	0.127306672	NA	Putative kinetochore protein with a predicted role in sister chromatid cohesion; repressed during the mating process; flow model biofilm induced
orf19.7490	orf19.7490	7.6	5.61	8.98	4.9	-0.004782149	NA	Predicted membrane transporter; fucose:proton symporter family member, MFS superfamily; flow model biofilm induced

orf19.5011	KAR9	8.19	5.6	11.08	10.19	0.179165662	NA	Ortholog of S. cerevisiae Kar9; role in mitotic spindle positioning; nonessential; localizes to bud tip, bud neck, hyphal tip; wild-type localization requires actin and microtubule cytoskeleton; induced by alpha factor
orf19.6017	orf19.6017	4.8	5.6	6.16	7.86	0.082997691	NA	Protein of unknown function; Spider biofilm induced
orf19.4116	orf19.4116	8.38	5.59	7.92	7.33	0.02429666	NA	Ortholog(s) have N-acetyltransferase activity, role in response to drug and nuclear envelope, plasma membrane localization
orf19.1192	DNA2	5.1	5.57	3.84	4.95	-0.064042219	NA	Protein similar to S. cerevisiae Dna2p, which is a DNA replication factor involved in DNA repair; induced under hydroxyurea treatment
orf19.5753	HGT10	6.34	5.55	7.57	4.97	0.023468695	NA	Glycerol permease involved in glycerol uptake; member of the major facilitator superfamily; induced by osmotic stress, at low glucose in rich media, during cell wall regeneration; 12 membrane spans; Hap43p-induced gene
orf19.2479	UGA4	5.88	5.55	5.98	4.31	-0.063960692	NA	Putative gamma-aminobutyric acid/polyamine permease; nitrogen catabolite repressed gene, induced in absence of preferred N sources; transcriptionally induced upon phagocytosis by macrophage; gene regulation by nitrogen source requires Gat1p
orf19.1932	CFL4	5.12	5.55	1.47	0.78	-0.306340469	NA	C-terminus similar to ferric reductases; induced in low iron; Sfu1-repressed; ciclopirox olamine induced; colony morphology-related gene regulation by Ssn6; Hap43-repressed; Sef1-regulated
orf19.3266	orf19.3266	7.27	5.54	7.08	4.66	-0.032908044	NA	Ortholog of C. dubliniensis CD36 : Cd36_25930, C. parapsilosis CDC317 : CPAR2_800910, Candida tenuis NRRL Y-1498 : CANTEDRAFT_132053 and Debaryomyces hansenii CBS767 : DEHA2E17160g
orf19.3100	orf19.3100	6.43	5.54	9.36	7.71	0.134251387	NA	Protein with t-SNARE domains and a microtubule associated domain; Hap43-induced gene; repressed by alpha pheromone in SpiderM medium

orf19.3971	orf19.3971	9.56	5.53	5.52	7.27	-0.083458648	NA	Protein of unknown function; early-stage flow model biofilm induced
orf19.7094	HGT12	6.35	5.53	26.87	19.71	0.770003311	NA	Glucose, fructose, mannose transporter; major facilitator superfamily; role in macrophage-induced hyphal growth; detected at germ tube plasma membrane by mass spectrometry; Snf3p-induced; 12 probable transmembrane segments
orf19.2739	orf19.2739	7.02	5.51	10.34	8.4	0.13120301	NA	Ortholog(s) have histone binding activity, role in DNA replication-dependent nucleosome assembly and CAF-1 complex, chromosome, centromeric region, nucleus localization
orf19.2044	PGA27	4.35	5.5	3.83	3.19	-0.067837654	NA	Putative GPI-anchored protein
orf19.4962	RMP1	7.66	5.49	4.98	4.98	-0.089722617	NA	Ortholog of Rmp1; subunit of RNase MRP subunit that processes pre-rRNA and has a role in cell cycle-regulated degradation of daughter cell-specific mRNAs; rat catheter and Spider biofilm induced
orf19.7093	HGT13	6.5	5.49	10.72	10.38	0.236764497	NA	Predicted sugar transporter, involved in glycerol utilization; member of the major facilitator superfamily; 12 transmembrane; gene has intron; oxidative stress-induced via Cap1p; expressed in rich medium, 2% glucose
orf19.4737	TPO3	4.86	5.49	4.63	3.37	-0.066337297	NA	Putative polyamine transporter; MFS-MDR family; induced by Sfu1, regulated upon white-opaque; decreased expression in hyphae vs yeast-form cells; regulated by Nrg1; Spider biofilm repressed
orf19.3699	TEP1	5.01	5.49	4.67	4.35	-0.034505271	NA	Putative protein phosphatase of the PTP family (tyrosine-specific), similar to S. cerevisiae Tep1p
orf19.3931.1	orf19.3931.1	2.72	5.45	2.72	1.36	-0.137545945	NA	Pseudogene; added to Assembly 21 based on comparative genome analysis

orf19.3940	orf19.3940	7.55	5.45	8.57	6.48	0.051691533	NA	Ortholog of C. dubliniensis CD36 : Cd36_54170, C. parapsilosis CDC317 : CPAR2_100960, Candida tenuis NRRL Y-1498 : CANTEDRAFT_96367 and Debaryomyces hansenii CBS767 : DEHA2G24618g
orf19.7537	BNR1	6.82	5.43	9.39	5.61	0.080066186	NA	Formin; probable role in hyphal cytoskeletal polarity; synthetic lethality if Bnr1p and Bni1p are absent
orf19.4206	orf19.4206	6.03	5.43	8.02	6.81	0.110723211	NA	Ortholog(s) have crossover junction endodeoxyribonuclease activity
orf19.5244	MCD4	6.69	5.43	10.14	9.12	0.186761002	NA	Protein of major facilitator superfamily; has phosphodiesterase/nucleotide pyrophosphatase domain; similar to S. cerevisiae Mcd4p, which acts in GPI anchor biosynthesis; possibly an essential gene, disruptants not obtained by UAU1 method
orf19.1767	orf19.1767	7.76	5.43	6.33	5.72	-0.030544927	NA	Ortholog(s) have protein complex scaffold, ubiquitin-specific protease activity, role in histone H3-K4 methylation, histone H3-K79 methylation, histone deubiquitination and DUBm complex, SAGA complex, SLIK (SAGA-like) complex localization
orf19.2745	UME7	4.34	5.42	4.06	3.11	-0.067837654	NA	Putative transcription factor with zinc cluster DNA-binding motif; similar to S. cerevisiae Ume6p, which is a transcription factor involved in the regulation of meiotic genes
orf19.7353	orf19.7353	7.13	5.42	8.73	5.47	0.051792799	NA	Ortholog(s) have plus-end-directed microtubule motor activity, tubulin-dependent ATPase activity
orf19.36	orf19.36	10.39	5.41	11.39	11.24	0.175280793	NA	Component of the conserved oligomeric Golgi complex; predicted to mediate fusion of transport vesicles to Golgi compartments; clade-associated gene expression
orf19.3777	IMG2	13.53	5.41	4.86	7.01	-0.180242415	NA	Mitochondrial ribosomal protein of the large subunit; rat catheter biofilm induced

orf19.7056	orf19.7056	4.17	5.4	6.07	5.61	0.086512548	NA	Putative protein of unknown function; transcript is upregulated in clinical isolates from HIV+ patients with oral candidiasis; regulated by Sef1, Sfu1, and Hap43
orf19.2227	orf19.2227	5.84	5.38	8.51	4.92	0.080609638	NA	Protein of unknown function; flow model biofilm induced; nitric oxide-repressed
orf19.4510	IFA4	5.86	5.38	5.35	7.27	0.027603108	NA	Protein of unknown function; oxidative stress-induced via Cap1
orf19.4459	orf19.4459	10.1	5.38	22.55	15.21	0.506318604	NA	Predicted heme-binding stress-related protein; Tn mutation affects filamentous growth; induced during chlamydospore formation in C. albicans and C. dubliniensis; Spider biofilm induced
orf19.7609	PGA11	3.22	5.36	2.67	3.74	-0.035425626	NA	Putative GPI-anchored protein
orf19.6784	PGA32	4.92	5.35	4.33	4.61	-0.033306392	NA	Putative GPI-anchored adhesin-like protein; induced in high iron; Spider biofilm induced
orf19.7380	orf19.7380	5.94	5.35	9.87	8.55	0.216473967	NA	Has domain(s) with predicted nucleic acid binding, nucleotide binding activity
orf19.1208	orf19.1208	7.19	5.33	8.07	5.62	0.053304671	NA	Predicted ORF in the Major Repeat Sequence on chromosome 4; member of a family encoded by FGR6-related genes in the RB2 repeat sequence
orf19.2962	orf19.2962	4.27	5.33	3.62	2.34	-0.100963454	NA	Protein of unknown function; Spider biofilm induced

orf19.1202	orf19.1202	5.88	5.32	8.24	8.37	0.138402171	NA	Ortholog of C. dubliniensis CD36 : Cd36_60350, C. parapsilosis CDC317 : CPAR2_603550, Candida tenuis NRRL Y-1498 : CANTEDRAFT_114325 and Debaryomyces hansenii CBS767 : DEHA2G04950g
orf19.7495	orf19.7495	6.62	5.32	2.73	1.87	-0.224276534	NA	Protein with NADPH oxidoreductase containing flavin mononucleotide (FMN) domain; induced by nitric oxide
orf19.4170	CSP2	5.99	5.3	9.19	8.27	0.164526226	NA	Putative cell wall associated protein; C. albicans and C. dubliniensis specific gene highly induced during chlamydospore development in both species; localized to chlamydospore cell wall; Hap43-repressed; Spider biofilm induced
orf19.4590	RFX2	4.63	5.29	10.66	6.37	0.189891656	NA	Transcriptional repressor; regulator of filamentation, response to DNA damage, adhesion, virulence in murine mucosal, systemic infections; RFX domain; regulated by Nrg1, UV-induced; partially complements S. cerevisiae rfx1 mutant defects
orf19.6958	ECM18	4.55	5.28	6.82	5.37	0.055062066	NA	Ortholog(s) have mitochondrion localization
orf19.1405	PHO13	5.28	5.28	12.87	14.03	0.439949256	NA	Putative 4-nitrophenylphosphatase; Hap43p-repressed gene; transcription is regulated upon yeast-hyphal switch
orf19.4569	orf19.4569	6.23	5.27	3.82	3.58	-0.095139096	NA	Ortholog of C. dubliniensis CD36 : Cd36_42100, Debaryomyces hansenii CBS767 : DEHA2C14850g, Pichia stipitis Pignal : PICST_52615 and Spathaspora passalidarum NRRL Y-27907 : SPAPADRAFT_58608
orf19.1090	orf19.1090	5.24	5.24	11.62	0.58	0.070051162	NA	Ortholog of C. parapsilosis CDC317 : CPAR2_601110, C. dubliniensis CD36 : Cd36_64660, Candida tenuis NRRL Y-1498 : CANTEDRAFT_119969 and Lodderomyces elongisporus NRLL YB-4239 : LELG_04881
orf19.2512	orf19.2512	6.7	5.24	8.14	4.36	-0.004473593	NA	Ortholog of C. dubliniensis CD36 : Cd36_81000, C. parapsilosis CDC317 : CPAR2_103050, Candida tenuis NRRL Y-1498 : CANTEDRAFT_116326 and Debaryomyces hansenii CBS767 : DEHA2G01958g

orf19.3727	PHO112	4.68	5.24	5.55	7.6	0.113953999	NA	Putative constitutive acid phosphatase; Rim101-repressed; DTT-extractable; N-glycosylated; Hap43-induced; possibly an essential gene, disruptants not obtained by UAU1 method
orf19.6501	orf19.6501	2.82	5.21	6.72	4.76	0.115511913	NA	Ortholog of C. dubliniensis CD36 : Cd36_71960, Pichia stipitis Pignal : psti_CGOB_00136, Spathaspora passalidarum NRRL Y-27907 : SPAPADRAFT_63786 and Candida tropicalis MYA-3404 : CTRG_05049
orf19.2228	orf19.2228	5.2	5.2	8.09	8.39	0.168287736	NA	Ortholog(s) have actin filament binding, actin monomer binding activity and role in actin cortical patch organization, actin filament depolymerization, actin filament severing, sequestering of actin monomers
orf19.5827	BUB2	4.8	5.2	3.59	3.59	-0.06547878	NA	Ortholog(s) have GTPase activator activity, role in mitotic spindle orientation checkpoint, negative regulation of septation initiation signaling cascade and cytosol, mitotic spindle pole body, nucleus localization
orf19.7475	PHO81	5.51	5.19	6.87	5.27	0.025475273	NA	Protein involved in regulation of hyphal development; required for response to farnesoic acid; possibly adherence-induced
orf19.5587	orf19.5587	10.12	5.19	16.07	12.61	0.331137907	NA	Protein of unknown function; transcript is upregulated in clinical isolates from HIV+ patients with oral candidiasis
orf19.3427	orf19.3427	3.46	5.19	5.18	4.03	0.026610012	NA	Ortholog of Candida albicans WO-1 : CAWG_05207
orf19.2905	orf19.2905	2.01	5.17	3.44	2.29	-0.070992552	NA	Ortholog of Candida albicans WO-1 : CAWG_03194
orf19.7550	IFA14	4.17	5.17	4.74	5.89	0.057588288	NA	Putative LPF family protein; Plc1-regulated; induced by alpha pheromone in SpiderM medium

orf19.2510	orf19.2510	4.61	5.17	5.83	5.4	0.026317457	NA	Ortholog(s) have role in protein folding, tubulin complex assembly
orf19.3147	orf19.3147	5.67	5.16	6.94	4.11	-0.004397796	NA	Ortholog of C. dubliniensis CD36 : Cd36_21050, C. parapsilosis CDC317 : CPAR2_104310, Candida tenuis NRRL Y-1498 : CANTEDRAFT_104515 and Debaryomyces hansenii CBS767 : DEHA2F01650g
orf19.2991	HOL1	6.01	5.16	6.62	5.04	0.025475273	NA	Putative MFS transporter; regulated by Nrg1; macrophage/pseudohyphal-repressed; induced by alpha pheromone in SpiderM medium; possibly an essential gene, disruptants not obtained by UAU1 method
orf19.1624.1	orf19.1624.1	3.87	5.16	5.14	7.71	0.115838609	NA	Ortholog of C. parapsilosis CDC317 : CPAR2_405060 and Candida orthopsilosis Co 90-125 : CORT_0C04470
orf19.857	FMO2	7.34	5.16	16.54	13.78	0.464009909	NA	Protein with a monooxygenase domain; Spider biofilm induced
orf19.3374	ECE1	5.36	5.13	336.83	167.52	3.401436212	1.35E-33	Hypha-specific protein; regulated by Rfg1, Nrg1, Tup1, Cph1, Efg1, Hog1, farnesol, phagocytosis; fluconazole-induced; rat catheter and Spider biofilm induced; flow model biofilm repressed; Bcr1-repressed in RPMI a/a biofilms
orf19.5596	orf19.5596	8.11	5.13	7.27	7.43	0.026060903	NA	Ortholog(s) have ASTRA complex localization
orf19.2350	orf19.2350	5.6	5.12	2.73	3.8	-0.12662558	NA	Protein similar to S. cerevisiae Yor378w; MFS family transporter; transposon mutation affects filamentous growth; null mutants are viable; fungal-specific (no human or murine homolog)
orf19.4153	orf19.4153	4.88	5.11	8.8	5.44	0.110037994	NA	Ortholog(s) have NEDD8 activating enzyme activity, role in protein neddylation and cytosol, nucleus localization

orf19.4643	orf19.4643	5.54	5.11	6.06	5.7	0.026638751	NA	Ortholog of C. dubliniensis CD36 : Cd36_41430, Candida tropicalis MYA-3404 : CTRG_00187 and Candida albicans WO-1 : CAWG_03642
orf19.5999	DYN1	4.29	5.11	4.78	5.05	0.027093978	NA	Dynein heavy chain; motor protein that moves to microtubule minus end; required for yeast cell separation, spindle positioning, nuclear migration, hyphal growth; regulated by Mig1, Hap43; flow model and rat catheter biofilm repressed
orf19.1522	orf19.1522	4.53	5.11	5.51	3.98	-0.004305464	NA	Ortholog of C. dubliniensis CD36 : Cd36_16850, C. parapsilosis CDC317 : CPAR2_211990, Candida tenuis NRRL Y-1498 : cten_CGOB_00169 and Debaryomyces hansenii CBS767 : DEHA2G03036g
orf19.4939	MON2	5	5.08	8.86	6.85	0.166976164	NA	Peripheral membrane protein; role in endocytosis and vacuole integrity; flow model and rat catheter biofilm repressed
orf19.4936	orf19.4936	7.07	5.05	5.04	4.53	-0.061685267	NA	Dubious open reading frame
orf19.2812	orf19.2812	5.91	5.05	7.62	7.61	0.138402171	NA	Protein of unknown function; Spider biofilm induced
orf19.4383	orf19.4383	3.67	5.04	5.82	7.02	0.115690794	NA	Ortholog(s) have DNA 5'-adenosine monophosphate hydrolase activity, mismatched DNA binding, zinc ion binding activity, role in cellular response to DNA damage stimulus and cytosol, nucleus localization
orf19.258	orf19.258	8.05	5.03	6.02	6.52	-0.001404281	NA	Protein of unknown function; Spider biofilm induced
orf19.460	CEK2	2.85	5.03	2.85	3.01	-0.069019652	NA	MAP kinase required for wild-type efficiency of mating; component of the signal transduction pathway that regulates mating; ortholog of S. cerevisiae Fus3; induced by Cph1, pheromone; transposon mutation affects filamentous growth

orf19.3330.3	POX18	1.5	5.01	3	3.99	0.027485941	NA	Similar to Pox18, a peroxisomal protein; induced during chlamydospore formation in C. albicans and C. dubliniensis; A21 sequence updated based on new sequence and analysis, the allelic orf19.10841 was reinstated; Spider biofilm induced
orf19.193	orf19.193	8.17	4.99	5.43	8.82	0.027772227	NA	Ortholog of C. dubliniensis CD36 : Cd36_19310, C. parapsilosis CDC317 : CPAR2_209710, Candida tenuis NRRL Y-1498 : CANTEDRAFT_113479 and Debaryomyces hansenii CBS767 : DEHA2C15114g
orf19.4945	MSH6	4.33	4.99	5.93	4.17	0.025850654	NA	Protein similar to S. cerevisiae Msh6p, which is involved in mismatch repair; repressed under Cdc5p depletion; Hap43p-induced gene
orf19.1427	orf19.1427	6.6	4.98	8.69	7.44	0.107863642	NA	Putative transporter; fungal-specific; Spider biofilm induced
orf19.4712	FGR6-3	3.26	4.98	6.71	6.63	0.173797456	NA	Protein lacking an ortholog in S. cerevisiae; member of a family encoded by FGR6-related genes in the RB2 repeat sequence; transposon mutation affects filamentous growth
orf19.7444	orf19.7444	6.61	4.96	5.31	3.11	-0.124519142	NA	Ortholog(s) have cytoplasm, holo TFIIH complex localization
orf19.2897	SOU2	4.06	4.95	4.94	5.17	0.027093978	NA	Protein similar to Sou1; not required for utilization of L-sorbose; Spider biofilm induced
orf19.3466	orf19.3466	5.6	4.94	7.72	4.1	0.024109625	NA	Predicted methyltransferase; downregulated by fluphenazine treatment or in an azole-resistant strain that overexpresses CDR1 and CDR2
orf19.2952	EXG2	3.63	4.92	3.49	2.58	-0.10043774	NA	GPI-anchored cell wall protein, similar to S. cerevisiae exo-1,3-beta-glucosidase Exg2p; predicted Kex2p substrate; induced during cell wall regeneration; possibly an essential gene, disruptants not obtained by UAU1 method; Hap43p-repressed

orf19.3148	orf19.3148	4.99	4.92	4.98	3.77	-0.034505271	NA	Protein of unknown function; possibly an essential gene, disruptants not obtained by UAU1 method
orf19.4861	orf19.4861	5.62	4.91	3.27	4.43	-0.12662558	NA	Protein of unknown function; rat catheter biofilm repressed
orf19.1146	orf19.1146	6.01	4.91	10.89	15.24	0.384980525	NA	Ortholog of Candida albicans WO-1 : CAWG_00265
orf19.5542	SAP6	3.44	4.9	3.93	1.85	-0.069770342	NA	Secreted aspartyl protease; expressed during hyphal growth, oral carriage, infection; sap4,5,6 triple mutant defective in protein utilization as N source; virulence role affecte by URA3; N-glycosylated; rat catheter biofilm induced
orf19.956	orf19.956	4.67	4.9	6.83	6.54	0.113376168	NA	Ortholog(s) have role in fermentation and mitochondrion localization
orf19.6562	RNH35	4.04	4.9	7.48	5.9	0.114730316	NA	Putative ribonuclease H2 catalytic subunit; flucytosine induced; Spider biofilm repressed
orf19.7340	orf19.7340	1.22	4.9	0.61	1.22	-0.142506621	NA	Protein of unknown function; possibly C. albicans specific; Spider biofilm induced
orf19.3780	orf19.3780	5.18	4.89	6.6	5.17	0.055062066	NA	Protein likely to be essential for growth, based on an insertional mutagenesis strategy
orf19.6803	HUT1	4.56	4.89	5.72	6.39	0.056314884	NA	Ortholog(s) have UDP-galactose transmembrane transporter activity, role in UDP-galactose transmembrane transport, UDP-glucose transport, regulation of protein folding in endoplasmic reticulum and endoplasmic reticulum localization

orf19.1776	orf19.1776	5.85	4.87	6.22	8.55	0.111911441	NA	Putative pantetheine-phosphate adenylyltransferase (PPAT); which catalyzes 4th step in coenzyme A biosynthesis from pantothenate; rat catheter biofilm repressed
orf19.3998	orf19.3998	4.85	4.85	5.64	3.62	-0.004305464	NA	Ortholog(s) have Atg8 ligase activity, protein binding, bridging activity and role in C-terminal protein lipidation, CVT pathway, macroautophagy, mitochondrion degradation, piecemeal microautophagy of nucleus
orf19.1167	orf19.1167	4.52	4.85	4.19	5.32	-0.033306392	NA	Ortholog(s) have sulfonate dioxygenase activity and role in sulfur compound catabolic process
orf19.4608	PDC12	5.57	4.84	9.15	9.35	0.191232576	NA	Putative pyruvate decarboxylase; fungal-specific (no human or murine homolog)
orf19.245	DDC1	3.3	4.83	5.32	4.43	0.026610012	NA	Putative DNA damage checkpoint protein; transcript induced during filamentous growth; regulated by Nrg1, Tup1
orf19.2943.5	orf19.2943.5	4.13	4.82	14.42	8.24	0.344991365	NA	Ortholog of Candida albicans WO-1 : CAWG_05543
orf19.3698	orf19.3698	9.02	4.79	6.75	7.31	-0.001553514	NA	Protein of unknown function that may function in RNA processing; filament induced
orf19.6027	orf19.6027	9.39	4.78	8.17	6.47	-0.00259154	NA	Ortholog of C. dubliniensis CD36 : Cd36_00740, C. parapsilosis CDC317 : CPAR2_110170, Candida tenuis NRRL Y-1498 : CANTEDRAFT_133175 and Debaryomyces hansenii CBS767 : DEHA2D09724g
orf19.7073	orf19.7073	6.62	4.76	5.01	4.22	-0.092932235	NA	Ortholog of S. cerevisiae : YCL002C, C. glabrata CBS138 : CAGL0B04719g, C. dubliniensis CD36 : Cd36_70180, C. parapsilosis CDC317 : CPAR2_805360 and Candida tenuis NRRL Y-1498 : cten_CGOB_00114

orf19.2431	orf19.2431	6.54	4.75	5.93	3.55	-0.062590417	NA	Dubious open reading frame, only conserved in Candida dubliniensis; induced by alpha pheromone in SpiderM medium; mRNA binds She3; Spider biofilm induced; flow model biofilm repressed
orf19.4320	orf19.4320	2.11	4.74	3.15	7.88	0.11732587	NA	Ortholog of Candida albicans WO-1 : CAWG_04676
orf19.1080	orf19.1080	3.47	4.74	2.77	3.8	-0.035425626	NA	Ortholog of C. dubliniensis CD36 : Cd36_64900, C. parapsilosis CDC317 : CPAR2_600390, Pichia stipitis Pignal : PICST_30726 and Candida guilliermondii ATCC 6260 : PGUG_04023
orf19.7293	MPS1	4.64	4.73	6.68	5.34	0.055062066	NA	Monopolar spindle protein, a putative kinase; essential for growth; periodic mRNA expression, peak at cell-cycle S/G2 phase
orf19.3283	orf19.3283	4.45	4.72	3.05	5.26	-0.033628047	NA	Ortholog(s) have 3-oxoacyl-[acyl-carrier-protein] reductase (NADPH) activity, role in aerobic respiration, fatty acid metabolic process and mitochondrion localization
orf19.1330	orf19.1330	5.72	4.71	3.62	3.96	-0.095139096	NA	Ortholog of Candida albicans WO-1 : CAWG_05678
orf19.929	orf19.929	2.57	4.71	5.27	6.84	0.117470339	NA	Protein of unknown function; rat catheter biofilm repressed
orf19.4505	ADH3	7.35	4.7	10.71	6.5	0.158509998	NA	Putative NAD-dependent (R,R)-butanediol dehydrogenase; regulated by white-opaque switch; induced by nitric oxide independent of Yhb1; Spider biofilm induced
orf19.7517	CHT1	6.99	4.7	7.37	5.63	0.025815557	NA	Chitinase; putative N-terminal catalytic domain; has secretory signal sequence; lacks S/T region and N-glycosylation motifs of Chs2p and Chs3p; alkaline downregulated; expression not detected in yeast-form or hyphal cells

orf19.5376	orf19.5376	7.04	4.69	8.16	7.93	0.109063947	NA	Ortholog of S. cerevisiae Elm1; a serine/threonine protein kinase that regulates cellular morphogenesis, septin behavior, and cytokinesis in S. cerevisiae; possibly an essential gene, disruptants not obtained by UAU1 method
orf19.3204	orf19.3204	7.42	4.67	6.82	4.4	-0.033079303	NA	Ortholog of C. dubliniensis CD36 : Cd36_51610, C. parapsilosis CDC317 : CPAR2_303630, Candida tenuis NRRL Y-1498 : CANTEDRAFT_135125 and Debaryomyces hansenii CBS767 : DEHA2E19030g
orf19.4507	orf19.4507	6.29	4.66	7.9	9.53	0.191584088	NA	Protein of unknown function; Hap43-repressed; rat catheter biofilm repressed
orf19.104	orf19.104	3.77	4.66	4.21	4.43	-0.035061523	NA	Protein of unknown function; induced by alpha pheromone in SpiderM medium
orf19.2837	ALG5	6.02	4.66	7.55	3.87	0.024109625	NA	Putative glucosyltransferase involved in cell wall mannan biosynthesis; possibly an essential gene, disruptants not obtained by UAU1 method
orf19.712	KIP1	4.65	4.64	4.03	2.75	-0.098320671	NA	Non-essential kinesin from the bipolar (Kinesis-5) family; involved in formation of the mitotic spindle
orf19.6482	orf19.6482	8.1	4.58	5.27	3.16	-0.152310352	NA	Protein of unknown function; induced by nitric oxide independent of Yhb1p; regulated by Sef1, Sfu1, and Hap43; rat catheter biofilm induced
orf19.4245	orf19.4245	6.24	4.57	11.2	10.09	0.266673186	NA	Protein with a predicted pleckstrin domain; Hap43-repressed gene
orf19.6480	orf19.6480	7.56	4.56	7.06	4.71	-0.031544237	NA	Ortholog(s) have role in UDP-glucose transport and COPI-coated vesicle, Golgi apparatus localization

orf19.660	orf19.660	7.71	4.56	7.97	4.93	-0.003521134	NA	Protein of unknown function; mRNA binds She3
orf19.4070	orf19.4070	5.47	4.55	9.09	6.81	0.166976164	NA	Ortholog of C. dubliniensis CD36 : Cd36_23270
orf19.3626	orf19.3626	5.77	4.55	6.37	5.58	0.026638751	NA	Ortholog(s) have cytosol, nucleus localization
orf19.5290	orf19.5290	5.71	4.55	5.86	3.56	-0.03382913	NA	Protein of unknown function; repressed by Sfu1; Hap43-induced gene
orf19.7029	orf19.7029	5.87	4.55	4.14	3.53	-0.095139096	NA	Putative guanine deaminase; mutation confers hypersensitivity to toxic ergosterol analog; Spider biofilm induced
orf19.5536	orf19.5536	4.21	4.54	4.96	5.92	0.057588288	NA	Has domain(s) with predicted carbon-nitrogen ligase activity, with glutamine as amido-N-donor, hydrolase activity, acting on carbon-nitrogen (but not peptide) bonds, in linear amides activity
orf19.416	orf19.416	2.68	4.52	5.34	5.51	0.088084754	NA	Ortholog of C. dubliniensis CD36 : Cd36_05200, C. parapsilosis CDC317 : CPAR2_107660, Candida tenuis NRRL Y-1498 : cten_CGOB_00159 and Debaryomyces hansenii CBS767 : DEHA2E07854g
orf19.554	DIT2	5.48	4.51	6.75	5.14	0.055062066	NA	Monooxygenase of the cytochrome P450 family; produces N,N'-bisformyl dityrosine from N-formyltyrosine; regulated by Nrg1, Mig1, and Tup1; role in chlamydospore formation; Spider biofilm induced
orf19.5223	orf19.5223	6.01	4.51	8.99	7.99	0.164526226	NA	Ortholog of Candida albicans WO-1 : CAWG_00199

orf19.4195	orf19.4195	5.51	4.51	8.99	5.49	0.080609638	NA	Ortholog of C. dubliniensis CD36 : Cd36_60540, C. parapsilosis CDC317 : CPAR2_602830, Candida tenuis NRRL Y-1498 : CANTEDRAFT_108530 and Debaryomyces hansenii CBS767 : DEHA2F15532g
orf19.873	orf19.873	3.37	4.49	5.04	5.59	0.121633167	NA	Ortholog of Candida albicans WO-1 : CAWG_04115
orf19.4750	orf19.4750	5.54	4.48	3.16	3.86	-0.095388604	NA	Ortholog of C. dubliniensis CD36 : Cd36_08410, C. parapsilosis CDC317 : CPAR2_301700, Candida tenuis NRRL Y-1498 : cten_CGOB_00267 and Debaryomyces hansenii CBS767 : DEHA2C10560g
orf19.7411	OAC1	7.33	4.47	4.1	4.63	-0.061192788	NA	Putative mitochondrial inner membrane transporter; rat catheter biofilm induced
orf19.2645	orf19.2645	6.38	4.46	4.45	5.72	-0.000770533	NA	Ortholog of Candida albicans WO-1 : CAWG_04710
orf19.5387	VPS8	6.58	4.45	6.56	5.02	0.026500007	NA	Putative vacuolar sorting protein; downregulated upon adherence to polystyrene
orf19.5191	FGR6-1	4.16	4.45	3.95	1.98	-0.068945914	NA	Protein lacking an ortholog in S. cerevisiae; member of a family encoded by FGR6-related genes in the RB2 repeat sequence; transposon mutation affects filamentous growth
orf19.2958	orf19.2958	5.32	4.45	6.91	6.83	0.145005909	NA	Planktonic growth-induced gene
orf19.5655	orf19.5655	5.59	4.44	5.86	7.43	0.086489331	NA	Has domain(s) with predicted 2 iron, 2 sulfur cluster binding, iron ion binding, oxidoreductase activity, acting on paired donors, with incorporation or reduction of molecular oxygen and NAD(P)H as one donor, more

orf19.2310	orf19.2310	3.8	4.43	2.11	2.32	-0.137382694	NA	Predicted single-stranded nucleic acid binding protein; flow model biofilm induced
orf19.1943	orf19.1943	6.38	4.43	5.28	4.34	-0.033133295	NA	Ortholog of C. parapsilosis CDC317 : CPAR2_302610, C. dubliniensis CD36 : Cd36_51070, Candida orthopsilosis Co 90-125 : CORT_0E04770 and Spathaspora passalidarum NRRL Y-27907 : SPAPADRAFT_50634
orf19.5589	orf19.5589	6.94	4.42	3.78	3.15	-0.125810602	NA	Protein of unknown function; transcript detected on high-resolution tiling arrays
orf19.2517	orf19.2517	3.5	4.4	5.29	5.74	0.121633167	NA	Predicted membrane transporter, member of the drug:proton antiporter (12 spanner) (DHA1) family, major facilitator superfamily (MFS)
orf19.1265	orf19.1265	4.69	4.4	8.78	7.14	0.19864455	NA	Ortholog(s) have Rab guanyl-nucleotide exchange factor activity and role in CVT pathway, early endosome to Golgi transport, intra-Golgi vesicle-mediated transport, macroautophagy, regulation of Rab GTPase activity
orf19.3010	orf19.3010	9.66	4.39	7.01	5.47	-0.056767442	NA	Putative lipoyl ligase; role in modification of mitochondrial enzymes by attachment of lipoic acid groups; rat catheter biofilm induced
orf19.3908	MRV8	4.4	4.39	7.2	3.13	0.056212038	NA	Protein of unknown function; Spider biofilm induced
orf19.7588	orf19.7588	7.4	4.39	4.84	5.07	-0.031346975	NA	Ortholog(s) have mitochondrion localization
orf19.6949	orf19.6949	3.75	4.37	5.61	7.47	0.148369812	NA	Ortholog of Candida albicans WO-1 : CAWG_02687

orf19.4573	ZCF26	6.96	4.36	4.35	3.39	-0.125810602	NA	Zn2-Cys6 transcription factor of unknown function; induced by alpha pheromone in SpiderM medium
orf19.7491	RTT109	5.05	4.35	5.38	6.25	0.058930167	NA	Histone acetyltransferase, mutants are sensitive to DNA damage, show decreased virulence in mice, decreased white-to-opaque switching and increased susceptibility to killing by macrophages
orf19.768	SYG1	4.28	4.34	2.72	3.09	-0.068192845	NA	Ortholog(s) have role in signal transduction and Golgi apparatus, endoplasmic reticulum, mitochondrion, plasma membrane localization
orf19.5573	orf19.5573	3.65	4.34	2.77	6.06	0.030071034	NA	Protein of unknown function; expression downregulated in an ssr1 null mutant
orf19.3925	orf19.3925	7.36	4.33	8.63	3.02	0.023567557	NA	Putative protein of unknown function, transcription is activated in the presence of elevated CO2
orf19.2468	orf19.2468	2.88	4.31	5.12	6.14	0.121633167	NA	Ortholog(s) have trans-aconitate 3-methyltransferase activity and cytosol, nucleus localization
orf19.6024	orf19.6024	3.78	4.31	4.84	5.2	0.059504424	NA	Ortholog of C. dubliniensis CD36 : Cd36_00770, Candida tenuis NRRL Y-1498 : CANTEDRAFT_112621, Debaryomyces hansenii CBS767 : DEHA2D09812g and Pichia stipitis Pignal : PICST_61958
orf19.1834	orf19.1834	4.99	4.31	4.64	5.98	0.058930167	NA	Ortholog of C. dubliniensis CD36 : Cd36_09960, C. parapsilosis CDC317 : CPAR2_212580, Debaryomyces hansenii CBS767 : DEHA2G11770g and Pichia stipitis Pignal : PICST_51041
orf19.4162	MLH1	6.09	4.29	3.94	3.59	-0.06398998	NA	Putative mismatch repair protein; cell-cycle regulated periodic mRNA expression

orf19.1580	orf19.1580	3.07	4.29	1.75	2.79	-0.069358291	NA	Protein of unknown function; Spider biofilm induced
orf19.1581	orf19.1581	3.06	4.28	3.05	3.05	-0.036256323	NA	Planktonic growth-induced gene
orf19.6997	ATO5	5.63	4.28	2.92	3.14	-0.129346362	NA	Putative fungal-specific transmembrane protein
orf19.2530	orf19.2530	1.22	4.28	3.05	1.83	-0.004761912	NA	Dubious open reading frame
orf19.6579	orf19.6579	6.13	4.28	6.83	6.25	0.085502856	NA	Ortholog of C. dubliniensis CD36 : Cd36_71340, C. parapsilosis CDC317 : CPAR2_301350, Candida tenuis NRRL Y-1498 : CANTEDRAFT_114098 and Debaryomyces hansenii CBS767 : DEHA2D03432g
orf19.3406	orf19.3406	3.99	4.25	4.24	4.5	0.029252068	NA	Predicted chloride transporter; member of conserved Mcm1 regulon; Spider biofilm repressed
orf19.690	PLB2	4.15	4.25	6.97	7.07	0.176397039	NA	Putative phospholipase B; conserved catalytic region; 6 putative N-glycosylation motifs; predicted secretion signal; no GPI anchor predicted; fungal-specific (no human or murine homolog)
orf19.1158	orf19.1158	4.45	4.24	8.88	6.97	0.230175685	NA	Ortholog of S. cerevisiae Yft2 required for normal ER membrane biosynthesis; Hap43-repressed gene
orf19.6247	orf19.6247	2.47	4.23	2.81	3.44	-0.003679212	NA	Ortholog(s) have chromatin binding activity

orf19.1411	orf19.1411	5.97	4.22	3.69	4.51	-0.031931078	NA	Putative cytochrome P450; Hap43-repressed gene
orf19.1110	orf19.1110	6.9	4.22	9.95	8.22	0.189808144	NA	Thiamine pyrophosphokinase, phosphorylates thiamine to produce the coenzyme thiamine pyrophosphate (thiamine diphosphate); active as a homodimer
orf19.6185	orf19.6185	9.62	4.22	10.79	0	-0.077874866	NA	Putative pseudouridine-5'-phosphate glycosidase; overlaps orf19.6186 and orf19.6187; Spider biofilm repressed
orf19.7173	orf19.7173	4.64	4.2	4.73	4.08	-0.00292247	NA	Ortholog of C. parapsilosis CDC317 : CPAR2_702310, C. dubliniensis CD36 : Cd36_73730, Lodderomyces elongisporus NRLL YB-4239 : LELG_04029 and Candida orthopsilosis Co 90-125 : CORT_0G02490
orf19.4017	orf19.4017	4.2	4.2	5.19	3.39	-0.003952471	NA	Ortholog of C. dubliniensis CD36 : Cd36_54840, C. parapsilosis CDC317 : CPAR2_100270, Candida tenuis NRRL Y-1498 : cten_CGOB_00242 and Debaryomyces hansenii CBS767 : DEHA2D14058g
orf19.2946	HNM4	5.3	4.19	6.88	4.42	0.056321753	NA	Putative choline permease; fungal-specific (no human or murine homolog)
orf19.4880	orf19.4880	4.52	4.17	10.2	7.73	0.251786259	NA	Protein of unknown function; flow model biofilm induced
orf19.2925	orf19.2925	4.81	4.17	8.31	3.84	0.084529071	NA	Ortholog(s) have role in protein folding, tubulin complex assembly and cytoplasm localization
orf19.1717	orf19.1717	5.52	4.17	3.63	4.16	-0.06398998	NA	Ortholog of C. dubliniensis CD36 : Cd36_81270, C. parapsilosis CDC317 : CPAR2_502880, Candida tenuis NRRL Y-1498 : CANTEDRAFT_101483 and Debaryomyces hansenii CBS767 : DEHA2A11990g

orf19.4244	orf19.4244	7.42	4.12	10.69	6.16	0.160370008	NA	Ortholog of Candida albicans WO-1 : CAWG_04619
orf19.6777	orf19.6777	6.03	4.11	3.83	7.65	0.058538659	NA	Ortholog of Candida albicans WO-1 : CAWG_03032
orf19.22.1	orf19.22.1	2.87	4.1	0.82	0.2	-0.216578555	NA	Pseudogene; added to Assembly 21 based on comparative genome analysis
orf19.4923	orf19.4923	2.63	4.06	2.62	3.69	-0.00260987	NA	Predicted membrane transporter; sugar porter (SP) family member, major facilitator superfamily; caspofungin induced; Spider biofilm induced
orf19.7362	SKN1	4.86	4.05	8.16	5.53	0.143630422	NA	Protein with a role in beta-1,6-glucan synthesis; probable N-glycosylated type II membrane protein; transcript and mRNA length change induced by yeast-hypha transition; induced by Rim101, caspofungin; rat catheter and Spider biofilm induced
orf19.6741	orf19.6741	3.9	4.05	5.98	6.28	0.119115923	NA	Putative plasma membrane protein; predicted role in cell wall integrity; regulated by Nrg1, Tup1; induced during chlamydospore formation in both C. albicans and C. dubliniensis
orf19.836	orf19.836	4.39	4.05	5.72	4.88	0.089010482	NA	Protein likely to be essential for growth, based on an insertional mutagenesis strategy
orf19.1349	orf19.1349	3.59	4.04	5.82	4.47	0.058110041	NA	Ortholog of C. dubliniensis CD36 : Cd36_22470, Candida tropicalis MYA-3404 : CTRG_01829 and Candida albicans WO-1 : CAWG_05920
orf19.3572	orf19.3572	2.89	4.04	2.73	2.01	-0.070707085	NA	Ortholog of C. dubliniensis CD36 : Cd36_19880, Candida tenuis NRRL Y-1498 : CANTEDRAFT_117672, Debaryomyces hansenii CBS767 : DEHA2D11154g and Pichia stipitis Pignal : PICST_59570

orf19.4668	orf19.4668	4.03	4.03	3.77	3.59	-0.002777707	NA	Protein with a glycoside hydrolase domain; mutants are viable
orf19.2038	orf19.2038	4.17	4.02	1.34	2.82	-0.134242096	NA	Protein of unknown function; Spider biofilm induced
orf19.1106	orf19.1106	2.67	4	4.07	3.91	0.02897777	NA	Protein with Mob2p-dependent hyphal regulation; fluconazole-induced
orf19.7455	orf19.7455	1.92	4	8.34	9.55	0.347764328	NA	Ortholog of C. dubliniensis CD36 : Cd36_86630 and Candida tropicalis MYA-3404 : CTRG_05698
orf19.2282	orf19.2282	4.56	3.99	5.68	5.3	0.057872331	NA	Has domain(s) with predicted catechol 1,2-dioxygenase activity, ferric iron binding activity and role in catechol-containing compound metabolic process, oxidation-reduction process
orf19.1113	orf19.1113	2.73	3.97	8.67	6.93	0.261844845	NA	Ortholog of C. dubliniensis CD36 : Cd36_53540, Debaryomyces hansenii CBS767 : DEHA2G07854g, Pichia stipitis Pignal : PICST_32162 and Spathaspora passalidarum NRRL Y-27907 : SPAPADRAFT_50795
orf19.4568	ZCF25	4.81	3.97	4.64	4.41	-0.00292247	NA	Putative Zn(II)2Cys6 transcription factor
orf19.1610	orf19.1610	3.31	3.97	5.61	3.96	0.089680925	NA	Putative protein of unknown function, transcription is activated in the presence of elevated CO2
orf19.1049	orf19.1049	3.97	3.97	4.62	4.95	0.059504424	NA	Predicted NUDIX hydrolase domain; Hap43-induced

orf19.1843	ALG6	7.58	3.95	7.34	5.58	0.026736891	NA	Putative glucosyltransferase involved in cell wall mannan biosynthesis; transcription is elevated in chk1, nik1, and sln1 homozygous null mutants; repressed by nitric oxide; possibly essential gene, disruptants not obtained by UAU1 method
orf19.1587	HGT20	4.43	3.94	6.63	7.74	0.204522928	NA	Putative glucose transporter of the major facilitator superfamily; the C. albicans glucose transporter family comprises 20 members; 12 probable membrane-spanning segments; regulated by Nrg1
orf19.7595	orf19.7595	7.17	3.94	6.03	5.61	0.027663688	NA	Has domain(s) with predicted dynactin complex localization
orf19.1430	orf19.1430	3.32	3.93	1.21	2.26	-0.139970367	NA	Ortholog of C. parapsilosis CDC317 : CPAR2_402120, C. dubliniensis CD36 : Cd36_43870, Lodderomyces elongisporus NRLL YB-4239 : LELG_04437 and Candida orthopsilosis Co 90-125 : CORT_0E02170
orf19.2907	PGA42	3.93	3.93	4.2	3.36	-0.035624302	NA	Putative GPI-anchored adhesin-like protein; decreased transcription is observed in azole-resistant strains that overexpress CDR1 and CDR2 or MDR1
orf19.1327	RBT1	2.05	3.92	3.32	3.74	0.029654079	NA	Cell wall protein with similarity to Hwp1; required for virulence; predicted glycosylation; fluconazole, Tup1 repressed; farnesol, alpha factor, serum, hyphal and alkaline induced; Rfg1, Rim101-regulated
orf19.5284	orf19.5284	0.65	3.91	2.6	4.55	0.093406722	NA	Dubious open reading frame
orf19.2060	SOD5	6.14	3.91	10.03	7.52	0.220457685	NA	Cu and Zn-containing superoxide dismutase; protects against oxidative stress; induced by neutrophils, hyphal growth, caspofungin, osmotic/oxidative stress; oralpharyngeal candidiasis induced; rat catheter and Spider biofilm induced
orf19.7051	orf19.7051	4.2	3.9	11.67	7.18	0.305004959	NA	Maf-like protein; rat catheter biofilm induced

orf19.3267	orf19.3267	6.91	3.9	2.3	4.24	-0.15567865	NA	Ortholog(s) have palmitoyltransferase activity and role in ascospore wall assembly, cortical actin cytoskeleton organization, establishment of cell polarity, protein palmitoylation, regulation of exocytosis, vacuole fusion, non-autophagic
orf19.7079	orf19.7079	3.36	3.89	5.48	4.2	0.059787237	NA	Ortholog(s) have role in axial cellular bud site selection and cellular bud neck contractile ring localization
orf19.570	IFF8	3.62	3.88	3.1	3.78	-0.034370878	NA	Putative GPI-anchored adhesin-like protein; decreased transcription is observed in an azole-resistant strain that overexpresses MDR1
orf19.3655	orf19.3655	3.38	3.86	4.72	4.34	0.059787237	NA	Ortholog of C. dubliniensis CD36 : Cd36_60640, C. parapsilosis CDC317 : CPAR2_602930, Candida tenuis NRRL Y-1498 : CANTEDRAFT_115038 and Pichia stipitis Pignal : PICST_31932
orf19.4466	orf19.4466	5.18	3.85	5.53	5.52	0.087891391	NA	Ortholog(s) have palmitoyltransferase activity and role in ascospore formation, cellular response to cadmium ion, detoxification of cadmium ion, protein palmitoylation, protein targeting to membrane
orf19.3474	IPL1	7.01	3.85	7.22	6.17	0.055671682	NA	Putative Aurora kinase; Hap43-induced; induced during planktonic growth; possibly an essential gene, disruptants not obtained by UAU1 method
orf19.1792	orf19.1792	4.99	3.84	6.29	3.88	0.027157924	NA	Ortholog(s) have ubiquitin-protein ligase activity
orf19.95	orf19.95	1.49	3.83	4.03	4.45	0.092869732	NA	Ortholog of S. cerevisiae : PRM5, C. dubliniensis CD36 : Cd36_60980, C. parapsilosis CDC317 : CPAR2_603060, Candida tenuis NRRL Y-1498 : CANTEDRAFT_113703 and Debaryomyces hansenii CBS767 : DEHA2F10032g
orf19.1178	orf19.1178	5.42	3.83	7.32	3.5	0.025652038	NA	Putative transcription factor with bZIP DNA-binding motif; Hap43p-repressed gene

orf19.3207	CCN1	3.91	3.82	5.67	4.34	0.058110041	NA	G1 cyclin; required for hyphal growth maintenance (not initiation); cell-cycle regulated transcription (G1/S); Cdc28p-Ccn1p initiates Cdc11p S394 phosphorylation on hyphal induction; expression in S. cerevisiae inhibits pheromone response
orf19.5925	orf19.5925	6.79	3.81	5.94	5.78	0.027663688	NA	Ortholog of S. cerevisiae : AIM6, C. glabrata CBS138 : CAGL0C05533g, C. dubliniensis CD36 : Cd36_84610, C. parapsilosis CDC317 : CPAR2_404550 and Candida tenuis NRRL Y-1498 : CANTEDRAFT_115337
orf19.6036	RIC1	7.14	3.81	8.81	5.18	0.081217636	NA	Ortholog of S. cerevisiae Ric1 guanyl-nucleotide exchange factor; mutant is viable; rat catheter biofilm repressed
orf19.2923	orf19.2923	7.71	3.79	8.08	7.03	0.081825108	NA	Predicted membrane transporter, member of the drug:proton antiporter (14 spanner) (DHA2) family, MFS superfamily; induced by alpha pheromone in SpiderM medium
orf19.2652	TEF4	1.46	3.79	4.94	3.78	0.12302315	NA	Putative translation elongation factor; genes encoding ribosomal subunits, translation factors, and tRNA synthetases are downregulated upon phagocytosis by murine macrophage
orf19.669	PRM1	2.67	3.76	3.75	4.05	0.02897777	NA	Putative membrane protein with a predicted role in membrane fusion during mating; Hap43p-repressed gene; protein induced during the mating process
orf19.2376	orf19.2376	5.13	3.75	10.5	14.91	0.447914657	NA	Has domain(s) with predicted DNA 5'-adenosine monophosphate hydrolase activity, DNA binding activity and role in DNA repair
orf19.7553	orf19.7553	4.83	3.75	4.55	3.47	-0.034837609	NA	Protein of unknown function; rat catheter biofilm repressed
orf19.2734	orf19.2734	6.55	3.74	4.85	6.53	0.02862228	NA	Protein with a glucose/ribitol dehydrogenase family domain; mutants are viable

orf19.4390	orf19.4390	4.47	3.72	12.38	8.67	0.359301847	NA	Protein of unknown function; repressed by alpha pheromone in SpiderM medium; transcript induced by Mnl1 under weak acid stress
orf19.103	KAR5	6.91	3.72	1.06	6.89	-0.083911589	NA	Ortholog of S. cerevisiae Kar5; involved in nuclear membrane fusion during karyogamy; induced by alpha factor
orf19.4965	orf19.4965	3.63	3.71	3.44	2.56	-0.068192845	NA	Protein of unknown function; rat catheter biofilm induced
orf19.4637	orf19.4637	7.27	3.7	7.38	5.19	0.026500007	NA	Ortholog(s) have role in SCF-dependent proteasomal ubiquitin-dependent protein catabolic process, cellular response to methylmercury and SCF ubiquitin ligase complex, cytosol localization
orf19.5929	orf19.5929	7.37	3.68	5.69	3.12	-0.062961054	NA	Putative protein of unknown function, transcription is positively regulated by Tbf1p
orf19.6789	orf19.6789	3.53	3.67	3.81	2.75	-0.035624302	NA	S. cerevisiae ortholog Nud1 is a spindle pole body outer plaque component; it acts through the mitotic exit network to specify asymmetric spindle pole body inheritance; Hap43-induced gene
orf19.2433	orf19.2433	4.9	3.67	4.27	3.66	-0.033822495	NA	Ortholog of C. dubliniensis CD36 : Cd36_05790, C. parapsilosis CDC317 : CPAR2_103730, Pichia stipitis Pignal : PICST_28344, Candida tropicalis MYA-3404 : CTRG_04275 and Candida albicans WO-1 : CAWG_00795
orf19.2827	orf19.2827	3.9	3.66	4.12	3.42	-0.035624302	NA	Ortholog(s) have structural constituent of cytoskeleton activity
orf19.530	orf19.530	5.78	3.66	6.89	5.48	0.056105758	NA	Protein with a UV radiation resistance protein/autophagy-related protein 14 domain; Hap43p-induced gene; mutants are viable

orf19.2647	ZCF14	4.22	3.63	4.33	5.37	0.029252068	NA	Putative Zn(II)2Cys6 transcription factor; caspofungin induced
orf19.2285	orf19.2285	2.41	3.62	2.62	2.84	-0.003679212	NA	Protein of unknown function; transcription induced by benomyl treatment
orf19.34	GIT1	4.53	3.58	5	4.88	0.028400503	NA	Glycerophosphoinositol permease; involved in utilization of glycerophosphoinositol as a phosphate source; Rim101-repressed; virulence-group-correlated expression
orf19.1686	orf19.1686	4.02	3.58	5.74	5.85	0.119115923	NA	Ortholog(s) have mitochondrion localization
orf19.3471	orf19.3471	8.91	3.56	1.78	2.96	-0.246092331	NA	Protein with a predicted anaphase-promoting complex APC subunit 1 CDC26 domain; Hap43-repressed gene
orf19.2213	orf19.2213	3.15	3.56	2.73	4.23	-0.00260987	NA	Ortholog(s) have cytoplasm, nucleus localization
orf19.230	orf19.230	6.08	3.56	5.3	4.63	-0.001921159	NA	Ortholog of S. cerevisiae Pet111; a mitochondrial translational activator specific for the COX2 mRNA; Hap43-induced gene
orf19.3453	orf19.3453	5.47	3.54	7.13	4.83	0.086548409	NA	Ortholog(s) have cellular bud tip, cytoplasm localization
orf19.3512	CSP1	1.77	3.53	1.76	3.17	-0.036615534	NA	Putative cell wall associated protein; gene only found in C. albicans and C. dubliniensis; highly upregulated during chlamydospore development in both species; localized to chlamydospore cell wall

orf19.3863	orf19.3863	4.41	3.52	4.1	2.83	-0.035624302	NA	Ortholog of C. dubliniensis CD36 : Cd36_31580, C. parapsilosis CDC317 : CPAR2_205000, Debaryomyces hansenii CBS767 : DEHA2G10846g and Pichia stipitis Pignal : PICST_32988
orf19.5714	SAP1	5.42	3.51	4.61	3.82	-0.00292247	NA	Secreted aspartyl proteinase; acts in utilization of protein as nitrogen source; assessment of virulence role complicated by URA3 effects; regulated by growth phase, alpha-pheromone; produced by opaque cells
orf19.2649	PCL1	6.03	3.51	8.01	6.34	0.112914816	NA	Cyclin homolog; transcript induced by filamentous growth; induced by alpha pheromone in SpiderM medium
orf19.2763	orf19.2763	3.29	3.51	4.95	2.89	0.027635861	NA	Protein not essential for viability; orf19.10279 possibly transcriptionally regulated upon hyphal formation
orf19.7083	DCC1	3.33	3.51	5.34	4.6	0.09116384	NA	Protein with a predicted role in sister chromatid cohesion and telomere length maintenance; cell-cycle regulated periodic mRNA expression
orf19.3450.1	orf19.3450.1	6.7	3.49	6.97	6.1	0.085598967	NA	Ortholog of C. dubliniensis CD36 : Cd36_61450, C. parapsilosis CDC317 : CPAR2_602710, Candida tenuis NRRL Y-1498 : cten_CGOB_00240 and Pichia stipitis Pignal : psti_CGOB_00191
orf19.6110	orf19.6110	8.16	3.49	12.79	12.78	0.379857708	NA	Ortholog of Candida albicans WO-1 : CAWG_01361
orf19.6583	orf19.6583	2.68	3.47	6.13	3.64	0.123875595	NA	Protein of unknown function; induced by alpha pheromone in SpiderM medium
orf19.3296	orf19.3296	4.33	3.46	5.18	2.71	0.029004697	NA	Ortholog of C. dubliniensis CD36 : Cd36_01020, C. parapsilosis CDC317 : CPAR2_110130, Candida tenuis NRRL Y-1498 : CANTEDRAFT_93515 and Debaryomyces hansenii CBS767 : DEHA2D14542g

orf19.3112	ZRT1	4.11	3.45	3.84	3.7	0.030345386	NA	Putative zinc transporter; acts with Pra1 in sequestration of zinc from host tissues during infection; hyphal, macrophage-induced; alkaline induced upon adherence to polystyrene; induced in oralpharyngeal candidasis; Spider biofilm induced
orf19.7377	ASE1	4.4	3.45	4.66	3.44	0.029004697	NA	Putative microtubule-associated protein; member of conserved Mcm1p regulon; periodic mRNA expression, peak at cell-cycle G2/M phase
orf19.708	orf19.708	4.72	3.45	4.71	2.88	-0.002635762	NA	Ortholog(s) have structural constituent of cytoskeleton activity
orf19.5762	PGA61	1.14	3.43	2.85	0.57	-0.004533854	NA	Putative GPI-anchored protein
orf19.2508	PRM9	2.1	3.42	2.97	1.98	-0.003429352	NA	Protein described a similar to S. cerevisiae Prm9; not the ortholog though; mutant is viable
orf19.2202	orf19.2202	6.59	3.42	6.57	10.51	0.219569453	NA	Protein of unknown function; induced by alpha pheromone in SpiderM medium
orf19.5271	orf19.5271	4.36	3.4	4.14	4.88	0.062297483	NA	Protein of unknown function; Hap43-induced gene
orf19.2906	PGA41	2.55	3.4	4.03	2.33	-0.00356555	NA	Putative GPI-anchored protein; adhesin-like protein
orf19.3264	CCE1	3.59	3.4	2.26	1.51	-0.103917916	NA	Putative Holliday junction resolving enzyme; similar to S. cerevisiae Cce1p

orf19.4405	PPS1	4.09	3.37	4.26	4.98	0.062297483	NA	Putative dual specificity protein phosphatase, similar to S. cerevisiae Pps1p; mutant shows virulence defect
orf19.1980	GIT4	3.37	3.37	1.97	1.62	-0.071294493	NA	Glycerophosphocholine transporter; fungal-specific (no human or murine homolog); possibly an essential gene, disruptants not obtained by UAU1 method; Hap43p-repressed gene
orf19.7320	LIP7	1.31	3.36	2.04	3.06	0.031754965	NA	Probable lipase, part of a gene family whose members are differentially expressed during infection; lacks a signal sequence for secretion, unlike other family members; may have a role in nutrition or in creating an acidic microenvironment
orf19.643	orf19.643	4.07	3.35	4.77	3.62	0.061191529	NA	Ortholog(s) have role in apoptotic process, mitotic spindle elongation, sister chromatid biorientation, spindle assembly checkpoint
orf19.7385	orf19.7385	4.45	3.34	5.18	4.07	0.061191529	NA	CCCH zinc finger protein; Spider biofilm induced
orf19.6313	MNT4	3.25	3.34	4.35	3.33	0.030114557	NA	Predicted alpha-1,3-mannosyltransferase with a role in protein glycosylation; induced in low iron
orf19.4325	orf19.4325	4.34	3.32	4.06	4.22	0.030345386	NA	Ortholog of C. dubliniensis CD36 : Cd36_52760, C. parapsilosis CDC317 : CPAR2_303150, Candida tenuis NRRL Y-1498 : CANTEDRAFT_133407 and Debaryomyces hansenii CBS767 : DEHA2G08030g
orf19.564	KAR3	5.11	3.32	3.93	3.22	-0.034144872	NA	Kinesin-like microtubule motor protein; required for nuclear fusion during mating; C-terminal motor domain; mutants are viable; null has high-frequency white-opaque switching phenotype
orf19.2590	orf19.2590	3.32	3.32	3.91	4.51	0.095030009	NA	Putative dethiobiotin synthetase; transcript upregulated in clinical isolates from HIV+ patients with oral candidiasis; Hap43-repressed; GlcNAc-induced protein; Spider biofilm induced

orf19.781	DUR3	4.16	3.31	4.4	4.57	0.062297483	NA	High affinity spermidine transporter; expression is induced by urea; fungal-specific (no human or murine homolog); not required for virulence in a mouse intravenous model
orf19.3356	ESP1	3.82	3.29	3.41	3.12	-0.03494742	NA	Putative caspase-like cysteine protease; mutation confers increased sensitivity to nocodazole; periodic mRNA expression, peak at cell-cycle S/G2 phase; mRNA binds She3
orf19.5775	orf19.5775	4.22	3.29	5.43	3.82	0.061191529	NA	Predicted ORF overlapping the Major Repeat Sequence on chromosome 6; member of a family encoded by FGR6-related genes in the RB2 repeat sequence; rat catheter biofilm repressed
orf19.7494	orf19.7494	3.19	3.29	3	2.43	-0.036892041	NA	Protein of unknown function; cell-cycle regulated periodic mRNA expression
orf19.4636	orf19.4636	5.64	3.29	3.75	1.4	-0.129937056	NA	Predicted protein of unknown function; overlaps orf19.4636
orf19.6001	SAP3	5.01	3.29	2.34	2.81	-0.099507836	NA	Secreted aspartyl proteinase, acts in utilization of protein as nitrogen source; assessment of virulence role complicated by URA3 effects; regulated by growth phase; produced by opaque phase cells; alpha-pheromone repressed
orf19.5023	DAL7	2.56	3.28	5.34	1.94	0.028499353	NA	Putative allantoate permease; mutant is viable
orf19.6737	orf19.6737	6.34	3.28	6.55	4.36	0.057038713	NA	Protein of unknown function; mutants are viable; induced in a cyr1, ras1, or efg1 homozygous null
orf19.812	orf19.812	1.22	3.26	1.22	2.03	-0.037932937	NA	Ortholog of C. dubliniensis CD36 : Cd36_18730, C. parapsilosis CDC317 : CPAR2_212510, Candida tenuis NRRL Y-1498 : CANTEDRAFT_135843 and Debaryomyces hansenii CBS767 : DEHA2D06732g

orf19.3667	orf19.3667	2.87	3.22	3.44	4.47	0.031304067	NA	Ortholog(s) have microtubule motor activity, protein homodimerization activity and kinesin complex localization
orf19.1321	HWP1	1.85	3.21	142.03	69.04	2.535442865	4.11E-16	Hyphal cell wall protein; host transglutaminase substrate; opaque-, a-specific, alpha-factor induced; at MTLa side of conjugation tube; virulence complicated by URA3 effects; Bcr1-repressed in RPMI a/a biofilms; Spider biofilm induced
orf19.1507	AMN1	3	3.18	2.8	2.05	-0.036892041	NA	Putative negative regulator of exit from mitosis; Plc1-regulated; rat catheter biofilm induced
orf19.5033	orf19.5033	0.79	3.17	5.93	3.16	0.157305631	NA	Ortholog(s) have Atg8 ligase activity, protein tag activity
orf19.5216	orf19.5216	8.45	3.17	3.16	1.89	-0.188421843	NA	Has domain(s) with predicted acyl-CoA hydrolase activity and role in acyl-CoA metabolic process
orf19.310	orf19.310	5.86	3.16	4.53	2.68	-0.033233699	NA	Putative mitochondrial protein with a predicted role in cell wall biogenesis; possibly an essential gene, disruptants not obtained by UAU1 method
orf19.2822	orf19.2822	4.26	3.14	7.39	4.7	0.150741266	NA	Essential component of the conserved oligomeric Golgi complex; role in fusion of transport vesicles to Golgi compartments; rat catheter biofilm repressed
orf19.5831	orf19.5831	1.56	3.12	1.27	2.41	-0.072556131	NA	Ortholog of C. parapsilosis CDC317 : CPAR2_212260, C. dubliniensis CD36 : Cd36_17620, Candida orthopsilosis Co 90-125 : CORT_0A11590 and Candida tropicalis MYA-3404 : CTRG_01348
orf19.6551.1	STE18	3.07	3.07	4.59	2.29	0.028499353	NA	Protein similar to S. cerevisiae Ste18p; expressed in opaque or white MTLa/MTLa or MTLalpha/MTLalpha, but not MTLa/MTLalpha cells; MTLa1p, MTLalpha2p bind promoter region

orf19.4115	orf19.4115	3.67	3.06	7.33	4.88	0.150741266	NA	Ortholog of Candida albicans WO-1 : CAWG_04349
orf19.1747	KIP2	3.15	3.06	4.81	2.4	0.028499353	NA	Putative kinesin-related motor protein involved in mitotic spindle positioning; repressed by alpha pheromone in SpiderM medium; periodic mRNA expression, peak at cell-cycle G2/M phase
orf19.4169	orf19.4169	2.53	3.03	6.04	4.53	0.155078672	NA	Ortholog of C. dubliniensis CD36 : Cd36_40780, Candida tenuis NRRL Y-1498 : CANTEDRAFT_116553, Lodderomyces elongisporus NRLL YB-4239 : LELG_04263 and Debaryomyces hansenii CBS767 : DEHA2G21736g
orf19.6636	orf19.6636	5.87	3.03	4.91	2.27	-0.065055646	NA	Protein of unknown function; rat catheter biofilm repressed
orf19.1409.3	orf19.1409.3	4.55	3.03	4.53	2.27	-0.034898187	NA	Protein of unknown function; Spider biofilm induced
orf19.7453	orf19.7453	5.09	3.01	6	5.76	0.119834555	NA	Ortholog of Candida albicans WO-1 : CAWG_02974
orf19.4834	orf19.4834	7.88	2.99	5.42	3.52	-0.060160064	NA	Ortholog of C. dubliniensis CD36 : Cd36_09140, C. parapsilosis CDC317 : CPAR2_804920, Debaryomyces hansenii CBS767 : DEHA2B10516g and Pichia stipitis Pignal : PICST_30058
orf19.403	PCL2	4.24	2.99	3.48	2.24	-0.069436537	NA	Cyclin homolog; reduced expression observed upon depletion of Cln3; farnesol regulated; periodic mRNA expression, peak at cell-cycle G1/S phase; Hap43-induced; rat catheter biofilm repressed
orf19.5056	orf19.5056	4.92	2.98	5.04	4.26	0.029277265	NA	Ortholog(s) have enzyme regulator activity, mitotic anaphase-promoting complex activity

orf19.262	SMC3	2.37	2.96	3.2	2.12	-0.003429352	NA	Protein similar to S. cerevisiae Smc3p, which is an ATPase involved in sister chromatid cohesion; likely to be essential for growth, based on an insertional mutagenesis strategy
orf19.1227	ZCF4	1.97	2.95	3.27	3.27	0.031084883	NA	Putative Zn(II)2Cys6 transcription factor
orf19.4356	HGT3	5.53	2.95	5.7	4.65	0.058583611	NA	Putative glucose transporter of the major facilitator superfamily; the C. albicans glucose transporter family comprises 20 members; 12 probable membrane-spanning segments, extended C terminus; expressed in rich medium with 2% glucose
orf19.1050	orf19.1050	3.56	2.93	8.16	3.14	0.116978218	NA	Ortholog of S. cerevisiae : YMR087W, C. glabrata CBS138 : CAGL0J01397g, C. dubliniensis CD36 : Cd36_03920, C. parapsilosis CDC317 : CPAR2_107150 and Pichia stipitis Pignal : PICST_61741
orf19.1825	orf19.1825	2.23	2.93	3.42	1.99	-0.003429352	NA	Protein of unknown function; mutants are viable; filament induced; regulated by Nrg1, Rfg1, Tup1
orf19.658	GIN1	3.27	2.92	4.1	4.4	0.063057156	NA	Protein involved in regulation of DNA-damage-induced filamentous growth; putative component of DNA replication checkpoint; ortholog of S. cerevisiae Mrc1p, an S-phase checkpoint protein; Hap43p-induced gene
orf19.2798	orf19.2798	3.67	2.92	3.66	2.16	-0.035944965	NA	Putative helicase; fungal-specific (no human or murine homolog)
orf19.3048	orf19.3048	4.7	2.91	4.88	3.52	0.029277265	NA	Protein of unknown function; repressed by alpha pheromone in SpiderM medium
orf19.5826	orf19.5826	3.32	2.9	3.62	4.26	0.063057156	NA	Predicted amino acid transmembrane transporter; rat catheter biofilm repressed

orf19.4856	LIP3	2.15	2.9	0.93	1.78	-0.072556131	NA	Secreted lipase; gene family member whose members are expressed differentially in response to carbon source and infection; possible role in nutrition and/or in creating an acidic microenvironment; flow model biofilm induced
orf19.3626.1	orf19.3626.1	0	2.88	0	0	-0.115069546	NA	Ortholog of C. parapsilosis CDC317 : CPAR2_406905, Candida orthopsilosis Co 90-125 : CORT_0C06265 and Candida tropicalis MYA-3404 : CTRG_05051
orf19.3571	orf19.3571	0.48	2.86	1.43	1.43	-0.039424231	NA	Ortholog of C. dubliniensis CD36 : Cd36_19870, C. parapsilosis CDC317 : CPAR2_209420, Candida tenuis NRRL Y-1498 : CANTEDRAFT_100922 and Debaryomyces hansenii CBS767 : DEHA2D11176g
orf19.1123	orf19.1123	2.29	2.86	0.57	2.85	-0.035841531	NA	Dubious open reading frame
orf19.4707	orf19.4707	2.33	2.85	1.38	2.15	-0.072556131	NA	Putative secreted acid sphingomyelin phosphodiesterase; possible Kex2p substrate
orf19.1737	orf19.1737	1.7	2.83	1.69	0	-0.109742075	NA	Ortholog of Candida albicans WO-1 : CAWG_01802
orf19.5616	orf19.5616	3.29	2.82	4.22	2.34	-0.00356555	NA	Ortholog of C. dubliniensis CD36 : Cd36_63740, C. parapsilosis CDC317 : CPAR2_503850, Pichia stipitis Pignal : PICST_32045 and Candida guilliermondii ATCC 6260 : PGUG_00070
orf19.3902	MRV2	4.86	2.81	1.79	1.53	-0.135387186	NA	Protein of unknown function; repressed by fluphenazine treatment or in an azole-resistant strain that overexpresses CDR1 and CDR2; Spider biofilm induced
orf19.3924	orf19.3924	3.46	2.8	7.23	5.09	0.183749082	NA	Ortholog of Candida albicans WO-1 : CAWG_04810

orf19.6786	orf19.6786	3.32	2.78	3.1	3.1	-0.002337356	NA	Protein of unknown function; flow model biofilm induced
orf19.7557	FGR46	2.78	2.78	1.66	1.66	-0.071294493	NA	Protein lacking an ortholog in S. cerevisiae; transposon mutation affects filamentous growth
orf19.5978	orf19.5978	2.47	2.77	1.54	2	-0.037632624	NA	Has domain(s) with predicted oxidoreductase activity, transferase activity, transferring acyl groups other than amino-acyl groups, zinc ion binding activity and role in oxidation-reduction process
orf19.242	SAP8	5.23	2.77	3.37	1.84	-0.100892677	NA	Secreted aspartyl protease; regulated by growth phase, temperature, white-opaque switch; highly expressed in opaque cells and upon deep epidermal invasion; greater expression in vaginal than oral infection; prominent role in biofilms
orf19.2394	orf19.2394	2.42	2.77	3.28	5.01	0.096873794	NA	Ortholog(s) have cytoplasm, nucleus localization
orf19.6896	orf19.6896	7.24	2.76	8.06	6.92	0.140633391	NA	Predicted ORF overlapping the Major Repeat Sequence on chromosome 7; member of a family encoded by FGR6-related genes in the RB2 repeat sequence
orf19.6398	orf19.6398	1.3	2.75	2.1	1.13	-0.039304518	NA	S. pombe ortholog SPBC460.04c is a predicted sulfonate/alpha-ketoglutare dioxygenase; induced by nitric oxide; Spider biofilm induced
orf19.5422	orf19.5422	1.53	2.75	5.19	3.36	0.094662809	NA	Ortholog of C. dubliniensis CD36 : Cd36_80400, Candida tropicalis MYA-3404 : CTRG_06188 and Candida albicans WO-1 : CAWG_02379
orf19.244	DCG1	3.56	2.74	2.73	4.65	0.031322968	NA	Protein of unknown function; ortholog of S. cerevisiae Dcg1; transcript regulated by Nrg1 and Mig1

orf19.7151	orf19.7151	2.73	2.73	1.82	0.91	-0.107679762	NA	Ortholog of Candida albicans WO-1 : CAWG_05751
orf19.4942	orf19.4942	1.64	2.73	1.09	0.54	-0.110638153	NA	Ortholog of Candida albicans WO-1 : CAWG_00134
orf19.5918	orf19.5918	9.43	2.69	6.04	6.04	0.000120109	NA	Ortholog of Candida albicans WO-1 : CAWG_02773
orf19.2796	orf19.2796	1.95	2.69	3.24	1.94	-0.003429352	NA	Ortholog(s) have DNA-directed DNA polymerase activity, role in DNA replication initiation, telomere capping and alpha DNA polymerase:primase complex, cytosol, nuclear envelope localization
orf19.7631	SLD5	8.62	2.69	5.64	3.22	-0.088079187	NA	Putative GINS complex subunit; cell-cycle regulated periodic mRNA expression
orf19.8	orf19.8	3.18	2.68	2.34	1.92	-0.071294493	NA	Ortholog of C. dubliniensis CD36 : Cd36_32690, C. parapsilosis CDC317 : CPAR2_204290, Candida tenuis NRRL Y-1498 : CANTEDRAFT_121459 and Debaryomyces hansenii CBS767 : DEHA2E03938g
orf19.5489	orf19.5489	1.72	2.66	2.65	2.34	-0.003429352	NA	Ortholog of C. dubliniensis CD36 : Cd36_20660, C. parapsilosis CDC317 : CPAR2_104730, Candida tenuis NRRL Y-1498 : CANTEDRAFT_133951 and Debaryomyces hansenii CBS767 : DEHA2F02068g
orf19.6354	orf19.6354	7.98	2.66	2.36	2.95	-0.186924948	NA	Ortholog of C. dubliniensis CD36 : Cd36_11870, C. parapsilosis CDC317 : CPAR2_201650, Candida tenuis NRRL Y-1498 : cten_CGOB_00224 and Debaryomyces hansenii CBS767 : DEHA2D04884g
orf19.5140	orf19.5140	2.21	2.66	1.77	1.59	-0.037632624	NA	Protein of unknown function; rat catheter and Spider biofilm induced

orf19.5172	LIP9	1.88	2.65	4.77	4.53	0.158728506	NA	Secreted lipase, member of a lipase gene family whose members are expressed differentially in response to carbon source and during infection; may have a role in nutrition and/or in creating an acidic microenvironment
orf19.6950	orf19.6950	4.41	2.64	3.96	3.08	-0.002497762	NA	Putative vacuolar membrane transporter for cationic amino acids; Spider biofilm induced
orf19.1340	orf19.1340	5.69	2.64	7.5	4.26	0.057038713	NA	Putative aldose reductase; protein level decreases in stationary phase cultures; Spider biofilm repressed
orf19.1605	PMS1	2.29	2.63	2.62	4.3	0.064575015	NA	Putative DNA mismatch repair factor; ortholog of S. cerevisiae PMS1 which is an ATP-binding protein involved in DNA mismatch repair
orf19.7017	YOX1	2.43	2.62	3.36	0.37	-0.072908489	NA	Putative homeodomain-containing transcription factor; transcriptional repressor; periodic mRNA expression, peak at cell-cycle G1/S phase
orf19.605	orf19.605	0.65	2.61	2.6	1.95	0.030509579	NA	Ortholog of Candida albicans WO-1 : CAWG_02087
orf19.3232	orf19.3232	1.43	2.61	7.23	6.75	0.308454446	NA	Putative transporter; mutation confers hypersensitivity to toxic ergosterol analog; fungal-specific (no human or murine homolog)
orf19.6008	orf19.6008	3.58	2.61	3.57	3.51	0.030345386	NA	S. cerevisiae ortholog YLL032C interacts with ribosomes; repressed by alpha pheromone in SpiderM medium
orf19.741	orf19.741	1.95	2.61	0	0	-0.188980285	NA	Dubious open reading frame

orf19.4391	orf19.4391	2.35	2.61	2.34	1.04	-0.073969359	NA	Ortholog of C. dubliniensis CD36 : Cd36_28730, Candida tropicalis MYA-3404 : CTRG_00749 and Candida albicans WO-1 : CAWG_01683
orf19.279	orf19.279	11.21	2.59	0	3.33	-0.32157489	NA	Protein with a predicted D-Tyr-tRNA(Tyr) deacylase domain; Hap43-repressed gene
orf19.557	orf19.557	2.57	2.57	2.56	2.75	-0.002337356	NA	Protein of unknown function; Hap43-repressed; Spider biofilm repressed
orf19.1639	orf19.1639	1.77	2.57	3.85	2.88	0.063413879	NA	Has domain(s) with predicted oxidoreductase activity and role in oxidation-reduction process
orf19.3285	orf19.3285	3.23	2.56	2.82	2.68	-0.002337356	NA	Ortholog of C. dubliniensis CD36 : Cd36_25800, Candida tenuis NRRL Y-1498 : CANTEDRAFT_116265, Debaryomyces hansenii CBS767 : DEHA2E17380g and Pichia stipitis Pignal : PICST_62836
orf19.2641	ARP1	3.16	2.56	3.3	2.85	-0.002337356	NA	Putative centractin; induced upon adherence to polystyrene
orf19.1748	orf19.1748	3.71	2.53	3.25	2.52	-0.03494742	NA	Protein of unknown function; shows colony morphology-related gene regulation by Ssn6
orf19.7252	orf19.7252	0	2.52	3.15	1.89	0.063687727	NA	Ortholog of Candida albicans WO-1 : CAWG_00017
orf19.3437	orf19.3437	3.31	2.51	2.39	1.71	-0.071294493	NA	Has domain(s) with predicted serine-type endopeptidase activity and role in proteolysis

orf19.306	USO5	4.09	2.49	4.37	2.19	-0.002184113	NA	Putative type II myosin heavy chain; transcription is induced upon filamentous growth and in response to alpha pheromone in SpiderM medium
orf19.3436	orf19.3436	3.92	2.49	4.98	2.49	0.029645675	NA	Protein with a Rho GDP-dissociation inhibitor domain; macrophage-induced gene
orf19.4567	orf19.4567	3.11	2.49	1.45	1.66	-0.071210193	NA	Catechol 1,2-dioxygenase (1,2-CTD), involved in degradation of aromatic compounds; Spider biofilm induced
orf19.5716	SAP4	2.17	2.49	12.19	7.44	0.443845018	NA	Secreted aspartyl proteinase; sap4,5,6 mutant defective in protein utilization for nitrogen; virulence role complicated by URA3 effects; expressed during mucosal and systemic infections; N-glycosylated; rat catheter, Spider biofilm induced
orf19.2781	orf19.2781	2.42	2.49	2.12	1.83	-0.001852491	NA	Protein with predicted serine/threonine kinase and tyrosine kinase domains; possibly an essential gene, disruptants not obtained by UAU1 method; Spider biofilm induced
orf19.6248	orf19.6248	1.09	2.46	2.18	1.9	0.033475805	NA	Ortholog of C. dubliniensis CD36 : Cd36_06210, Candida tropicalis MYA-3404 : CTRG_04227 and Candida albicans WO-1 : CAWG_00750
orf19.4104	orf19.4104	2.43	2.43	1.73	2.77	0.0336226	NA	Ortholog of C. dubliniensis CD36 : Cd36_20540, C. parapsilosis CDC317 : CPAR2_104860, Candida tenuis NRRL Y-1498 : CANTEDRAFT_116071 and Pichia stipitis Pignal : PICST_32327
orf19.1794	orf19.1794	2.43	2.43	5.2	3.46	0.130796819	NA	Ortholog(s) have RNA binding, translation regulator activity and role in Group I intron splicing, mitochondrial respiratory chain complex IV biogenesis, positive regulation of mitochondrial translation
orf19.3694	orf19.3694	1.92	2.43	3.98	3.38	0.099509557	NA	Ortholog of C. dubliniensis CD36 : Cd36_02210, C. parapsilosis CDC317 : CPAR2_106350, Candida tenuis NRRL Y-1498 : CANTEDRAFT_116785 and Debaryomyces hansenii CBS767 : DEHA2C01980g

orf19.5491	orf19.5491	1.58	2.37	1.73	1.1	-0.038337568	NA	Ortholog of C. dubliniensis CD36 : Cd36_20670, C. parapsilosis CDC317 : CPAR2_104720, Candida tenuis NRRL Y-1498 : CANTEDRAFT_103989 and Debaryomyces hansenii CBS767 : DEHA2F02046g
orf19.3711	orf19.3711	0.68	2.37	1.18	1.43	-0.039272862	NA	Protein with monooxygenase domains; flow model biofilm induced
orf19.1766	orf19.1766	2.8	2.33	3.56	2.01	0.031042625	NA	Secreted protein; fluconazole-induced
orf19.1351	orf19.1351	1.55	2.32	4.11	2.83	0.099509557	NA	Ortholog of C. parapsilosis CDC317 : CPAR2_407000, C. dubliniensis CD36 : Cd36_22440, Candida tenuis NRRL Y-1498 : cten_CGOB_00049 and Lodderomyces elongisporus NRLL YB-4239 : LELG_02401
orf19.6595	RTA4	3.13	2.31	3.67	2.58	0.06488572	NA	Protein similar to S. cerevisiae Rsb1p, involved in fatty acid transport; transposon mutation affects filamentous growth; alkaline downregulated; caspofungin induced; possibly an essential gene; Hap43p-repressed
orf19.5399	IFF11	2.18	2.3	1.57	1.93	-0.001852491	NA	Secreted protein required for normal cell wall structure and for virulence; member of the IFF family; Hap43p-repressed gene
orf19.3320	orf19.3320	1.72	2.29	4.56	3.99	0.163646795	NA	Dubious open reading frame
orf19.1768	orf19.1768	1.53	2.29	1.76	1.75	-0.001852491	NA	Ortholog of C. parapsilosis CDC317 : CPAR2_407480, C. dubliniensis CD36 : Cd36_24170, Candida orthopsilosis Co 90-125 : CORT_0C06830 and Spathaspora passalidarum NRRL Y-27907 : SPAPADRAFT_65786
orf19.1806	orf19.1806	1.05	2.27	2.79	1.74	0.067505647	NA	Ortholog of C. dubliniensis CD36 : Cd36_30340, C. parapsilosis CDC317 : CPAR2_203900, Candida tenuis NRRL Y-1498 : CANTEDRAFT_113094 and Debaryomyces hansenii CBS767 : DEHA2G22374g

orf19.4946	orf19.4946	6.01	2.25	2.5	5.49	0.001064733	NA	Ortholog of C. dubliniensis CD36 : Cd36_12240, C. parapsilosis CDC317 : CPAR2_801750, Candida tenuis NRRL Y-1498 : CANTEDRAFT_135403 and Pichia stipitis Pignal : PICST_29111
orf19.1714	PGA44	1.02	2.25	1.84	1.63	0.033475805	NA	Putative GPI-anchored protein
orf19.1122	orf19.1122	1.35	2.25	1.12	1.57	-0.001642595	NA	Protein of unknown function; induced by alpha pheromone in SpiderM medium; Spider biofilm induced
orf19.265	orf19.265	3.1	2.23	1.98	1.85	-0.036262309	NA	Protein with a ribonuclease III domain; flow model biofilm induced; Spider biofilm induced
orf19.6661	orf19.6661	2.71	2.23	3.02	3.18	0.032519212	NA	Predicted ORF from Assembly 19; removed from Assembly 20; subsequently reinstated in Assembly 21 based on comparative genome analysis
orf19.552	orf19.552	2.48	2.23	0.62	2.47	-0.036987956	NA	Ortholog of a S. cerevisiae Atg22; a vacuolar integral membrane protein required for efflux of amino acids during autophagic body breakdown in the vacuole; possibly an essential gene, disruptants not obtained by UAU1 method
orf19.6971	DIE2	2.67	2.22	3.55	3.1	0.06488572	NA	Ortholog(s) have dolichyl-phosphate-glucose-glycolipid alpha-glucosyltransferase activity, role in protein N-linked glycosylation and endoplasmic reticulum membrane localization
orf19.4473	SPC19	6.28	2.22	4.42	4.05	-0.000124495	NA	Essential subunit of the Dam1 (DASH) complex, which acts in chromosome segregation by coupling kinetochores to spindle microtubules
orf19.3986	PPR1	3.78	2.21	3.63	3.7	0.063972242	NA	Putative transcription factor with zinc cluster DNA-binding motif; has similarity to S. cerevisiae Ppr1p, which is a transcription factor involved in the regulation of uracil biosynthesis genes

orf19.7054	orf19.7054	1.65	2.2	0	1.65	-0.07189098	NA	Dubious open reading frame
orf19.5242	CDC6	2.58	2.19	3.99	2.83	0.06488572	NA	Putative ATP-binding protein with a predicted role in DNA replication; member of conserved Mcm1p regulon; periodic mRNA expression, peak at cell-cycle M/G1 phase
orf19.2035	orf19.2035	0.59	2.17	2.95	2.16	0.067505647	NA	Ortholog of C. parapsilosis CDC317 : CPAR2_212930, C. dubliniensis CD36 : Cd36_15830, Candida orthopsilosis Co 90-125 : CORT_0A12240 and Candida tropicalis MYA-3404 : CTRG_01155
orf19.2899	orf19.2899	2.17	2.17	0.96	1.44	-0.074946524	NA	Ortholog(s) have cytosol, nucleus localization
orf19.3306	orf19.3306	2.03	2.13	1.83	1.82	-0.001852491	NA	Ortholog(s) have four-way junction DNA binding activity, role in meiotic recombination checkpoint, synaptonemal complex assembly and lateral element localization
orf19.4647	HAP3	2.48	2.1	2.09	1.71	-0.001852491	NA	Predicted CCAAT-binding transcription factor that regulates respiration; Cap2-dependent induction in low iron; opaque specific, alkaline, ciclopirox olamine induced; regulated by Sef1, Sfu1, Hap43; Bcr1-regulated in RPMI a/a biofilms
orf19.5276	orf19.5276	2.4	2.1	1.5	1.64	-0.036987956	NA	Putative nuclear pore-associated protein; Hap43p-induced gene; induced upon low-level peroxide stress; possibly an essential gene, disruptants not obtained by UAU1 method
orf19.7452	orf19.7452	2.51	2.09	5.09	4.42	0.128729355	NA	Ortholog(s) have chromatin binding activity and role in DNA replication initiation, DNA unwinding involved in DNA replication, double-strand break repair via break-induced replication
orf19.1823	orf19.1823	2.35	2.08	3.42	2.21	0.032370773	NA	Predicted membrane protein; transcript repressed by ciclopirox olamine

orf19.4001	orf19.4001	3.99	2.07	2.95	2.06	-0.03526574	NA	Ortholog(s) have role in protein insertion into mitochondrial membrane from inner side and extrinsic to mitochondrial inner membrane, mitochondrial matrix localization
orf19.1741	DIT1	3.66	2.07	3.04	3.52	0.032446702	NA	Ortholog(s) have catalytic activity and role in ascospore wall assembly
orf19.6391	orf19.6391	2.47	2.05	2.25	2.05	-0.001852491	NA	Protein of unknown function; transcript upregulated in azole-resistant strain overexpressing MDR1; Spider biofilm induced
orf19.4923.1	orf19.4923.1	5.14	2.05	6.15	2.05	0.028247876	NA	Ortholog of C. parapsilosis CDC317 : CPAR2_801655, Candida tropicalis MYA-3404 : CTRG_06172 and Candida albicans WO-1 : CAWG_02233
orf19.2957	orf19.2957	1.22	2.05	2.2	1.67	0.033475805	NA	Protein required for transfer of mannosylphosphate; induced by alpha pheromone in SpiderM medium
orf19.3208	DAL52	2.04	2.04	2.49	1.35	-0.038337568	NA	Putative allantoate permease; mutant is viable; similar but not orthologous to S. cerevisiae Dal5
orf19.3202	orf19.3202	3.31	2.01	3.8	1.79	0.031042625	NA	Ortholog of C. dubliniensis CD36 : Cd36_51590, C. parapsilosis CDC317 : CPAR2_303610, Candida tenuis NRRL Y-1498 : CANTEDRAFT_135127 and Debaryomyces hansenii CBS767 : DEHA2E19074g
orf19.1774	orf19.1774	2.53	2.01	1.64	3.06	-0.000752279	NA	Predicted dehydrogenase; transcript upregulated in an RHE model of oral candidiasis; virulence-group-correlated expression; Spider biofilm repressed
orf19.6288	orf19.6288	3.17	1.98	1.98	4.34	0.033473704	NA	Dubious open reading frame

orf19.2513	orf19.2513	2.66	1.98	2.91	2.57	0.032519212	NA	Ortholog of C. dubliniensis CD36 : Cd36_81010, C. parapsilosis CDC317 : CPAR2_103060, Candida tenuis NRRL Y-1498 : CANTEDRAFT_127339 and Debaryomyces hansenii CBS767 : DEHA2G01936g
orf19.4656	orf19.4656	1.37	1.95	3.02	1.17	0.031789754	NA	Ortholog of C. dubliniensis CD36 : Cd36_41300, C. parapsilosis CDC317 : CPAR2_400390, Candida tenuis NRRL Y-1498 : CANTEDRAFT_94704 and Debaryomyces hansenii CBS767 : DEHA2B02992g
orf19.5289	orf19.5289	2.61	1.95	1.3	1.3	-0.109332336	NA	Dubious open reading frame
orf19.4251	ZCF22	2.38	1.94	3.07	4.21	0.100649915	NA	Predicted Zn(II)2Cys6 transcription factor
orf19.6025	orf19.6025	3.65	1.93	3.43	2.14	-0.03526574	NA	Ortholog(s) have N-acetylglucosaminyldiphosphodolichol N-acetylglucosaminyltransferase activity and role in dolichol-linked oligosaccharide biosynthetic process
orf19.6022	NRM1	2.36	1.93	2.67	1.6	0.032370773	NA	Transcriptional regulator of cell cycle gene expression; regulates expression of genes involved in DNA replication stress; interacts with Swi4 and Swi6; repressed during core stress response; Spider biofilm repressed
orf19.4509	orf19.4509	3.83	1.91	0	1.27	-0.17848234	NA	Protein of unknown function
orf19.3418	orf19.3418	1.73	1.9	2.42	1.38	-0.038337568	NA	Ortholog(s) have endoplasmic reticulum, fungal-type vacuole localization
orf19.3885	orf19.3885	2.07	1.9	1.55	2.58	0.0336226	NA	Has domain(s) with predicted N-acetyltransferase activity; highly induced during chlamydospore formation in both C. albicans and C. dubliniensis

orf19.2642	orf19.2642	1.52	1.89	2.64	2.64	0.067079011	NA	Ortholog(s) have myosin II binding activity, role in actomyosin contractile ring contraction and cellular bud neck contractile ring localization
orf19.6813	orf19.6813	1.18	1.89	2.36	4.25	0.102542976	NA	Protein of unknown function; Hap43-induced gene
orf19.4015	CAG1	2.03	1.88	2.6	1.45	-0.003139022	NA	heterotrimeric G protein alpha subunit; positive role in mating pheromone response; opaque-enriched transcript; transcript repressed by MTLa1-MTLalpha2; regulated by hemoglobin-responsive Hbr1 via MTL genes; rat catheter biofilm repressed
orf19.5777	orf19.5777	3.77	1.88	4.57	3.49	0.062465249	NA	Protein of unknown function; F-12/CO2 early biofilm induced
orf19.4394	orf19.4394	1.63	1.85	4.98	2.71	0.130796819	NA	Protein of unknown function; induced by alpha pheromone in SpiderM medium
orf19.4934	OP4	1.39	1.85	2.62	2	0.067505647	NA	Ala- Leu- and Ser-rich protein; secreted; N-terminal hydrophobic region; possible glycosylation; opaque-specific transcript; repressed by alpha pheromone in opaque MTLa homozygotes; fluconazole-induced; Spider biofilm induced
orf19.3140	orf19.3140	1.86	1.85	1.23	2.47	-0.036987956	NA	Ortholog of Candida albicans WO-1 : CAWG_03159
orf19.4599	PHO89	2.26	1.85	4.1	3.18	0.099509557	NA	Putative phosphate permease; transcript regulated upon white-opaque switch; alkaline induced by Rim101; possibly adherence-induced; F-12/CO2 model, rat catheter and Spider biofilm induced
orf19.762	orf19.762	1.14	1.84	1.31	2.18	-0.001642595	NA	Ortholog of C. parapsilosis CDC317 : CPAR2_105580, C. dubliniensis CD36 : Cd36_04550, Lodderomyces elongisporus NRLL YB-4239 : LELG_03202 and Candida orthopsilosis Co 90-125 : CORT_0B06800

orf19.4814	orf19.4814	0.61	1.82	0	0	-0.11730776	NA	Ortholog of C. dubliniensis CD36 : Cd36_08960, C. parapsilosis CDC317 : CPAR2_301680, Candida tenuis NRRL Y-1498 : cten_CGOB_00021 and Debaryomyces hansenii CBS767 : DEHA2F11088g
orf19.4908	orf19.4908	1.82	1.82	0.6	1.21	-0.074946524	NA	Dubious open reading frame
orf19.1822	UME6	2.19	1.82	7.05	4.72	0.253964931	NA	Zn(II)2Cys6 transcription factor; role in hyphal extension, virulence, adherence to plastic; rat catheter biofilm induced; has a long 5'-UTR that regulates translational efficiency and controls transition to filamentous growth
orf19.3529	ABP2	0.61	1.82	1.92	1.82	0.033475805	NA	Putative alpha-actinin-like protein; induced by alpha pheromone in SpiderM medium
orf19.4995	orf19.4995	1.22	1.82	4.24	1.97	0.100201172	NA	Ortholog of C. dubliniensis CD36 : Cd36_12580
orf19.4853	HCM1	2.03	1.81	4.15	3.51	0.132661126	NA	Protein with forkhead domain; similar to S. cerevisiae Hcm1p; Hap43p-induced gene
orf19.7303	orf19.7303	3.63	1.81	4.13	2.07	-0.002184113	NA	Ortholog of C. dubliniensis CD36 : Cd36_34495, Candida tropicalis MYA-3404 : CTRG_05947 and Candida albicans WO-1 : CAWG_02181
orf19.5042	orf19.5042	3.01	1.81	2.41	4.21	0.033473704	NA	Ortholog(s) have role in maintenance of rDNA and nuclear periphery localization
orf19.2481	orf19.2481	3.58	1.79	1.79	1.27	-0.105548929	NA	Protein of unknown function; Spider biofilm induced

orf19.4508	orf19.4508	0.89	1.78	3.85	2.67	0.134495045	NA	Ortholog of Candida albicans WO-1 : CAWG_04200
orf19.855	orf19.855	1.02	1.78	2.29	1.78	0.033475805	NA	Ortholog of C. dubliniensis CD36 : Cd36_18340, Candida tenuis NRRL Y-1498 : CANTEDRAFT_124631, Debaryomyces hansenii CBS767 : DEHA2D04356g and Pichia stipitis Pignal : PICST_34839
orf19.4872	orf19.4872	0.45	1.78	1.33	1.33	-0.002792964	NA	Protein of unknown function; induced by Mnl1 under weak acid stress; Hap43-induced; mutant is viable
orf19.5520	ASG7	1.77	1.77	0.88	0.44	-0.11328701	NA	a-cell specific protein of unknown function; two predicted transmembrane domains; member of conserved Mcm1 regulon; induced by alpha pheromone in SpiderM medium
orf19.2713	orf19.2713	1.69	1.76	1.53	1.45	-0.038337568	NA	Ortholog(s) have role in reciprocal meiotic recombination and nucleus localization
orf19.5717	orf19.5717	1.17	1.75	2.32	0.58	-0.002987928	NA	Ortholog of Candida albicans WO-1 : CAWG_05019
orf19.5915	DUR35	2.21	1.75	2.58	3.13	0.067079011	NA	Putative urea transporter
orf19.1541	orf19.1541	1.27	1.71	1.65	1.58	0.033475805	NA	Protein with a globin-like domain; repressed by alpha pheromone in SpiderM medium; Spider biofilm induced
orf19.6590	VMA22	2.4	1.71	5.12	2.73	0.130796819	NA	Ortholog(s) have unfolded protein binding activity, role in vacuolar acidification, vacuolar proton-transporting V-type ATPase complex assembly and extrinsic to endoplasmic reticulum membrane localization

orf19.1599	orf19.1599	5.66	1.7	3.39	0.56	-0.131536269	NA	Ortholog of Candida albicans WO-1 : CAWG_06018
orf19.4976	orf19.4976	2.27	1.7	2.26	1.69	-0.001852491	NA	Ortholog of Candida albicans WO-1 : CAWG_00105
orf19.2457	orf19.2457	1.69	1.69	3.29	2.93	0.067079011	NA	Protein of unknown function; induced during chlamydospore formation in both C. albicans and C. dubliniensis; Spider biofilm induced
orf19.4061	orf19.4061	2.37	1.66	3.07	1.89	0.032370773	NA	Protein of unknown function; clade-associated gene expression
orf19.1647	orf19.1647	2.02	1.65	2.56	2.93	0.067079011	NA	Ortholog(s) have role in ascospore wall assembly
orf19.935	AGA1	2.3	1.59	9.9	4.95	0.334373967	NA	Protein with some similarity to agglutinin subunit; expression regulated upon white-opaque switch; Spider biofilm induced
orf19.3420	orf19.3420	3.66	1.57	5.21	1.56	0.029645675	NA	Protein of unknown function; decreased expression in hyphae than in yeast
orf19.7568	orf19.7568	1.35	1.57	2.69	0.89	0.031789754	NA	Ortholog of S. cerevisiae : SPS4, C. glabrata CBS138 : CAGL0I09834g, C. dubliniensis CD36 : Cd36_35035, C. parapsilosis CDC317 : CPAR2_200810 and Candida tenuis NRRL Y-1498 : CANTEDRAFT_112327
orf19.1844	orf19.1844	1.15	1.56	1.39	0.98	-0.039272862	NA	Protein similar to ferric reductase Fre10p

orf19.4424	PHO100	1.94	1.55	0.97	0.97	-0.074946524	NA	Putative inducible acid phosphatase; DTT-extractable and observed in culture supernatant in low-phosphate conditions; slight effect on murine virulence; virulence-group-correlated expression; N-glycosylated; F-12/CO2 early biofilm induced
orf19.2395	IME2	1.07	1.55	1.31	1.66	-0.001642595	NA	Putative serine/threonine protein kinase; mutation confers hypersensitivity to amphotericin B
orf19.5212	orf19.5212	0.51	1.54	0.34	0.51	-0.076205238	NA	Ortholog(s) have SUMO ligase activity, role in protein sumoylation, reciprocal meiotic recombination, synaptonemal complex assembly and condensed nuclear chromosome localization
orf19.1780	orf19.1780	1.37	1.53	1.32	1.97	-0.001642595	NA	Has domain(s) with predicted catalytic activity
orf19.4011	orf19.4011	0.61	1.53	1.22	1.07	-0.039272862	NA	Similar to S. pombe mug180, a predicted esterase/lipase; highly induced during chlamydospore formation in both C. albicans and C. dubliniensis; flow model biofilm induced
orf19.5141	orf19.5141	1.28	1.53	1.36	1.36	-0.039272862	NA	Ortholog of Candida albicans WO-1 : CAWG_05647
orf19.5423	EST3	1.83	1.52	6.38	2.43	0.12731872	NA	Telomerase subunit; required for telomere maintenance in vivo; separable roles in telomerase activity and processivity; not required for catalytic activity in vitro; related to mammalian Tpp1p
orf19.6678	orf19.6678	0.84	1.51	4.84	3.34	0.16560705	NA	Protein of unknown function; Hap43-repressed gene
orf19.7364	orf19.7364	2.31	1.5	1.61	1.38	-0.001514458	NA	Has domain(s) with predicted N,N-dimethylaniline monooxygenase activity, NADP binding, flavin adenine dinucleotide binding activity and role in oxidation-reduction process

orf19.5266	orf19.5266	0.68	1.49	1.62	0.94	0.03497804	NA	Planktonic growth-induced gene
orf19.1616	FGR23	1.65	1.48	1.92	2.03	0.034982475	NA	Protein of unknown function; repressed by a1/alpha2 in white-phase cells, a-specific, alpha factor-induced; Hap43-repressed; flow model biofilm induced; Tn mutation affects filamentous growth;
orf19.6274	PBR1	0.37	1.48	1.84	0.74	0.072132625	NA	Protein of unknown function; required for cohesion, adhesion, and RPMI biofilm formation; induced by alpha pheromone in white cells; fluconazole-induced; Spider biofilm induced
orf19.7301	orf19.7301	0.95	1.47	4.52	2.73	0.204658347	NA	Has domain(s) with predicted DNA binding activity
orf19.2819	orf19.2819	1.77	1.47	0	2.64	0.001187295	NA	Ortholog of C. dubliniensis CD36 : Cd36_27570, C. parapsilosis CDC317 : CPAR2_801010, Candida tenuis NRRL Y-1498 : cten_CGOB_00198 and Debaryomyces hansenii CBS767 : DEHA2E09306g
CaalfMp01	COX2	0.48	1.45	0.72	0	-0.002497389	NA	Subunit II of cytochrome c oxidase, which is the terminal member of the mitochondrial inner membrane electron transport chain; one of three mitochondrially-encoded subunits
orf19.6475	orf19.6475	2.89	1.44	3.84	2.4	0.066040288	NA	Protein of unknown function; flow model biofilm induced
orf19.938	orf19.938	1.93	1.42	5.14	4.76	0.232191905	NA	Ortholog of C. dubliniensis CD36 : Cd36_50520, C. parapsilosis CDC317 : CPAR2_304000, Candida tenuis NRRL Y-1498 : cten_CGOB_00233 and Debaryomyces hansenii CBS767 : DEHA2E12408g
orf19.5144	PGA28	3.66	1.41	2.81	3.65	0.066183252	NA	Protein similar to S. cerevisiae Wsc2p, which has roles in stress- and cell-wall-related processes; predicted Kex2p substrate; putative GPI-anchored adhesin-like protein

orf19.5844	orf19.5844	0.35	1.4	0	0.7	-0.001075962	NA	Ortholog(s) have role in meiotic DNA recombinase assembly, reciprocal meiotic recombination and condensed nuclear chromosome localization
orf19.7482	orf19.7482	1.05	1.4	1.05	1.75	0.036305375	NA	Ortholog of C. parapsilosis CDC317 : CPAR2_803740, C. dubliniensis CD36 : Cd36_25640, Lodderomyces elongisporus NRLL YB-4239 : LELG_00467 and Candida orthopsilosis Co 90-125 : CORT_0A03760
orf19.2797	orf19.2797	1.29	1.39	1.38	1.28	-0.001314633	NA	Has domain(s) with predicted ATP binding, DNA binding, helicase activity
orf19.7127.1	orf19.7127.1	2.84	1.37	7.61	3.04	0.216763862	NA	Putative protein of unknown function, transcript is upregulated in an RHE model of oral candidiasis
orf19.3631	STN1	1.79	1.37	2.2	2.2	0.034982475	NA	Protein involved in telomere maintenance; forms a complex with Ten1p
orf19.6390	orf19.6390	0.68	1.36	0.68	0.68	-0.001314633	NA	Protein of unknown function; Spider biofilm induced
orf19.3621	orf19.3621	0.61	1.36	0.6	0.3	-0.040120758	NA	Possible pseudogene; similar to Ywp1p; ORF extended upstream from the initiating Met of orf19.3621 has a stop codon in the region corresponding to the Ywp1p signal peptide; disruption causes no apparent phenotype; no expression detected
orf19.1445	ESC4	0.88	1.36	1.63	0.88	0.03497804	NA	Protein similar to S. cerevisiae Esc4; a protein that represses transposition; transposon mutation affects filamentation; rat catheter biofilm repressed
orf19.4672	orf19.4672	0.68	1.36	2.04	2.04	0.071746301	NA	Ortholog of Candida albicans WO-1 : CAWG_03665

orf19.6267	orf19.6267	1.09	1.36	0.82	0.95	-0.001314633	NA	Ortholog of C. dubliniensis CD36 : Cd36_06020, C. parapsilosis CDC317 : CPAR2_803380, Candida tenuis NRRL Y-1498 : CANTEDRAFT_91858 and Pichia stipitis Pignal : PICST_30337
orf19.3196	orf19.3196	3.4	1.36	0.68	0.68	-0.072678399	NA	Ortholog of Candida albicans WO-1 : CAWG_04564
orf19.6864	orf19.6864	0.27	1.35	1.35	1.62	0.073449157	NA	Putative ubiquitin-protein ligase; role in protein ubiquitination; Spider biofilm induced
orf19.2247	orf19.2247	1.71	1.34	1.46	1.16	-0.037817236	NA	Ortholog of C. parapsilosis CDC317 : CPAR2_406700, C. dubliniensis CD36 : Cd36_21220, Candida tenuis NRRL Y-1498 : CANTEDRAFT_127772 and Candida orthopsilosis Co 90-125 : CORT_0C06120
orf19.7024	orf19.7024	2	1.33	2.66	1.33	0.033289175	NA	Ortholog of Candida albicans WO-1 : CAWG_05448
orf19.4652	orf19.4652	1.98	1.32	1.31	1.97	-0.000169167	NA	Protein of unknown function; rat catheter biofilm induced
orf19.5724	orf19.5724	0.26	1.32	0.26	0.79	-0.001075962	NA	Ortholog of C. parapsilosis CDC317 : CPAR2_500910, C. dubliniensis CD36 : Cd36_64110, Candida tenuis NRRL Y-1498 : CANTEDRAFT_115913 and Pichia stipitis Pignal : PICST_32870
orf19.4783	orf19.4783	0.79	1.31	1.04	0.78	-0.001314633	NA	Protein of unknown function; induced during chlamydospore formation in both C. albicans and C. dubliniensis
orf19.1778	orf19.1778	1.95	1.3	1.3	1.3	-0.037817236	NA	Dubious open reading frame

orf19.3209	FGR42	2.61	1.3	0	0.65	-0.10909907	NA	Protein lacking an ortholog in S. cerevisiae; transposon mutation affects filamentous growth
orf19.4472	orf19.4472	1.93	1.29	6.43	6.43	0.292704852	NA	Ortholog of Candida albicans WO-1 : CAWG_00996
orf19.2591	orf19.2591	1.58	1.29	2.86	2.43	0.069049269	NA	Ortholog(s) have adenosylmethionine-8-amino-7-oxononanoate transaminase activity and role in biotin biosynthetic process
orf19.4301	orf19.4301	1.43	1.28	2.63	1.66	0.105912539	NA	Ortholog(s) have role in chromatin silencing at rDNA, chromatin silencing at silent mating-type cassette, chromatin silencing at telomere, regulation of transcription from RNA polymerase II promoter and nucleus localization
orf19.2274	orf19.2274	1.53	1.28	1.28	1.02	-0.037817236	NA	Ortholog of C. dubliniensis CD36 : Cd36_21450, Candida tropicalis MYA-3404 : CTRG_01739 and Candida albicans WO-1 : CAWG_05814
orf19.2704	orf19.2704	0.64	1.28	0.64	0.64	-0.001314633	NA	Ortholog of Candida albicans WO-1 : CAWG_03507
orf19.3748	orf19.3748	0.64	1.28	1.27	0.64	-0.001314633	NA	Protein of unknown function; orf19.3747 and orf19.3748 are short, overlapping ORFs in intergenic region between 2 oligopeptide transporter-encoding genes; flow model biofilm induced
orf19.1746	orf19.1746	1.83	1.27	1.41	1.55	-0.000169167	NA	Protein similar to S. cerevisiae meiosis-specific, spore-wall-localized protein Ssp2p, which is required for wild-type outer spore wall formation in S. cerevisiae; possibly an essential gene (UAU1 method); Hap43p-repressed
orf19.2336	orf19.2336	3.49	1.27	1.58	3.16	0.034682201	NA	Pry family pathogenesis-related protein; predicted to be extracellular

orf19.6164	orf19.6164	0.62	1.25	0	1.87	8.81E-05	NA	Ortholog of Candida albicans WO-1 : CAWG_02424
orf19.6534	orf19.6534	0.84	1.25	2.5	3.75	0.17452792	NA	Ortholog of Candida albicans WO-1 : CAWG_05537
orf19.2885	PRI2	1.48	1.25	1.7	1.82	0.071746301	NA	Putative DNA primase; gene adjacent to and divergently transcribed with CDC68; Hap43-induced; Spider biofilm repressed
orf19.4571	orf19.4571	1.24	1.24	1.35	1.8	0.036305375	NA	CoA-transferase family protein; rat catheter biofilm repressed
orf19.3931.2	orf19.3931.2	1.24	1.24	0	0	-0.079610261	NA	Protein of unknown function
orf19.271	ADH4	0.74	1.23	1.22	0.73	-0.001314633	NA	Predicted 3-hydroxyacyl-CoA dehydrogenase; transcript is increased in azole-resistant strain overexpressing MDR1; transcript is increased in populations of cells exposed to fluconazole over multiple generations; mutant is viable
orf19.6570	NUP	3.07	1.23	4.9	3.21	0.130775036	NA	Nucleoside permease; adenosine and guanosine are substrates, whereas cytidine, adenine, guanine, uridine, uracil are not; similar to a nucleoside permease of S. pombe; possibly processed by Kex2p
orf19.1257	orf19.1257	1.22	1.22	1.83	1.22	0.03497804	NA	Ortholog of Candida albicans WO-1 : CAWG_03251
orf19.4310	orf19.4310	0.41	1.22	0.41	0	-0.041439977	NA	Dubious open reading frame

orf19.7606	orf19.7606	1.59	1.2	1.19	1.19	-0.037817236	NA	Ortholog of C. dubliniensis CD36 : Cd36_35262, Candida tropicalis MYA-3404 : CTRG_05848 and Candida albicans WO-1 : CAWG_02290
orf19.6687	orf19.6687	1.85	1.2	0.28	0.28	-0.115838505	NA	Protein of unknown function; rat catheter biofilm repressed
orf19.948	orf19.948	0	1.19	0.59	0.59	0.036277451	NA	Ortholog of Candida albicans WO-1 : CAWG_04435
orf19.5966	orf19.5966	0	1.19	0	0	-0.041439977	NA	Ortholog of Candida albicans WO-1 : CAWG_02810
orf19.2492	STE3	2.99	1.19	3.13	2.23	0.033436158	NA	Protein similar to S. cerevisiae Ste3p, the receptor for a-factor mating pheromone; alpha mating-type-specific transcription
orf19.138	FIG1	0.48	1.19	0.95	0.95	0.036277451	NA	S. cerevisiae Fig1 ortholog; an integral membrane protein required for mating; role in thigmotropism; transcript is opaque-specific and a-specific; activated by Cph1 or alpha pheromone
orf19.218	BUD20	0.59	1.19	1.19	0.59	-0.001314633	NA	Protein similar to S. cerevisiae Bud20p, which affects bud site selection; transposon mutation affects filamentous growth
orf19.2941	SCW4	0.53	1.18	2.03	1.49	0.03497804	NA	Putative cell wall protein; substrate for Kex2p processing in vitro; expression regulated by white-opaque switch; alkaline repressed; possibly essential (UAU1 method); flow model biofilm induced; Spider biofilm induced
orf19.6339	NRG2	0.59	1.18	1.17	1.76	0.036305375	NA	Transcription factor; transposon mutation affects filamentous growth

orf19.6919	orf19.6919	0.83	1.16	0.66	0.83	-0.001314633	NA	Ortholog of C. dubliniensis CD36 : Cd36_71210, C. parapsilosis CDC317 : CPAR2_702710, Candida tenuis NRRL Y-1498 : CANTEDRAFT_116256 and Debaryomyces hansenii CBS767 : DEHA2E17732g
orf19.6970	orf19.6970	1.94	1.16	3.68	5.61	0.232128946	NA	Ortholog of C. dubliniensis CD36 : Cd36_85310, C. parapsilosis CDC317 : CPAR2_807370, Candida tenuis NRRL Y-1498 : CANTEDRAFT_115544 and Debaryomyces hansenii CBS767 : DEHA2D11770g
orf19.3738	PGA22	1.53	1.15	2.3	0.76	-0.001514458	NA	Putative GPI-anchored protein; adhesin-like protein
orf19.217	orf19.217	1.65	1.15	10.35	9.36	0.488457079	NA	Ortholog(s) have sequence-specific DNA binding activity
orf19.2024	orf19.2024	0.56	1.12	0.56	5.59	0.16940256	NA	Ortholog of Candida albicans WO-1 : CAWG_03878
orf19.4200	orf19.4200	0.56	1.12	0.56	0	-0.040120758	NA	Ortholog of Candida albicans WO-1 : CAWG_05300
orf19.2033	PGA19	0.56	1.11	0.83	1.39	-0.001314633	NA	Putative GPI-anchored protein; macrophage-induced gene; mutants are viable
orf19.6574	ALK6	0.37	1.1	1.1	0.97	0.036277451	NA	Putative cytochrome P-450 of N-alkane-induced detoxification; macrophage-induced gene
orf19.7634	MCD1	0.99	1.1	2.08	1.31	0.03497804	NA	Alpha-kleisin cohesin complex subunit; for sister chromatid cohesion in mitosis and meiosis; repressed by alpha pheromone in SpiderM medium; periodic cell-cycle expression; Hap43-repressed; rat catheter and Spider biofilm repressed

orf19.6502	orf19.6502	0.91	1.09	0.9	1.09	-0.001314633	NA	Short-chain dehydrogenase/reductase; upregulation correlates with clinical development of fluconazole resistance
orf19.2317	orf19.2317	1.46	1.09	0.73	0.36	-0.040120758	NA	Protein of unknown function; induced by alpha pheromone in SpiderM medium
orf19.6851	CHL4	2.27	1.07	1.6	2	0.034982475	NA	Protein described as having role in chromosome segregation; RNA abundance regulated by tyrosol and cell density
CaalfMp11	COB	0	1.05	1.28	0	-0.002497389	NA	Cytochrome b, mitochondrially encoded subunit of the ubiquinol-cytochrome c reductase complex (complex III) that catalyzes a step in the respiratory chain of the mitochondrial inner membrane
orf19.756	SAP7	0.95	1.05	0.63	0.94	-0.001314633	NA	Pepstatin A-insensitive secreted aspartyl protease; self-processing; expressed in human oral infection; Ssn6p-regulated; role in murine intravenous infection; induced during, but not required for, murine vaginal infection; N-glycosylated
orf19.7105	FAR1	1.12	1.04	0.96	1.28	-0.001314633	NA	Protein involved in regulation of pheromone-mediated mating; repressed by A1p and Alpha2p in white-phase cells; null mutant shows no pheromone response in opaque cells; overexpression causes enhanced pheromone response and cell cycle arrest
orf19.7170	orf19.7170	2.05	1.03	0	1.02	-0.074759481	NA	Ortholog of C. dubliniensis CD36 : Cd36_73740, Candida tropicalis MYA-3404 : CTRG_05151 and Candida albicans WO-1 : CAWG_05736
orf19.355	orf19.355	0.29	1.03	0.59	0.44	-0.002497389	NA	Has domain(s) with predicted oxidoreductase activity and role in oxidation-reduction process
orf19.5894	orf19.5894	0.23	1.03	2.39	2.16	0.108762218	NA	Ortholog of S. cerevisiae : YEL023C, C. glabrata CBS138 : CAGL0I03168g, C. dubliniensis CD36 : Cd36_84380, Candida tenuis NRRL Y-1498 : CANTEDRAFT_101635 and Pichia stipitis Pignal : PICST_19384

orf19.6493	orf19.6493	0.51	1.02	3.05	1.02	0.069638866	NA	Ortholog of C. dubliniensis CD36 : Cd36_72020 and Candida albicans WO-1 : CAWG_05570
orf19.1611	orf19.1611	4.26	1.01	1.62	2.02	-0.034169675	NA	Protein of unknown function; Spider biofilm induced
orf19.2010	orf19.2010	2.02	1.01	1.01	2.52	0.035850263	NA	Ortholog of C. dubliniensis CD36 : Cd36_16030, C. parapsilosis CDC317 : CPAR2_213800, Debaryomyces hansenii CBS767 : DEHA2A01320g and Pichia stipitis Pignal : PICST_29434
orf19.4566	orf19.4566	2.66	1	0	1	-0.10909907	NA	Protein similar to S. cerevisiae Rot1p, which has roles in cytoskeletal dynamics and cell wall biosynthesis; predicted Kex2p substrate
orf19.5121	OPT5	1	1	1.4	0.74	-0.001314633	NA	Oligopeptide transporter; fungal-specific (no human or murine homolog); induced by BSA, but not by tetrapeptide or pentapeptide
orf19.3172	orf19.3172	0.5	0.99	1.98	1.49	0.03497804	NA	Ortholog of C. dubliniensis CD36 : Cd36_51840, Candida tropicalis MYA-3404 : CTRG_05315 and Candida albicans WO-1 : CAWG_04584
orf19.6532	orf19.6532	1.59	0.99	1.19	1.19	-0.037817236	NA	Ortholog(s) have FAD transmembrane transporter activity, role in FAD transport and mitochondrion localization
orf19.3906	MRV6	0.33	0.98	0	1.31	-0.001075962	NA	Ortholog of Candida albicans WO-1 : CAWG_04796
orf19.1215	orf19.1215	1.25	0.97	1.25	1.39	-0.001314633	NA	Ortholog of C. dubliniensis CD36 : Cd36_64550, Candida tropicalis MYA-3404 : CTRG_02747 and Candida albicans WO-1 : CAWG_04972

orf19.5954	orf19.5954	0.49	0.97	0.24	0.36	-0.041439977	NA	Ortholog(s) have enzyme activator activity, role in ascospore wall assembly, meiosis I, positive regulation of protein catabolic process and anaphase-promoting complex localization
orf19.1274	orf19.1274	1.95	0.96	1.12	1.83	-0.000169167	NA	Has domain(s) with predicted DNA binding, zinc ion binding activity, role in transcription, DNA-templated and nucleus localization
orf19.2429	orf19.2429	0.69	0.96	1.37	0.69	-0.001314633	NA	Protein of unknown function; induced by alpha pheromone in SpiderM medium
orf19.5264	orf19.5264	0.47	0.93	0.46	0.93	-0.001075962	NA	Dubious open reading frame
CaalfMp05	ATP9	0	0.93	3.72	0	0.100769043	NA	Subunit 9 of the F0 sector of mitochondrial F1F0 ATP synthase, which is a large, evolutionarily conserved enzyme complex required for ATP synthesis
orf19.1863	orf19.1863	0.21	0.93	0.1	0.21	-0.041439977	NA	Has domain(s) with predicted Rho guanyl-nucleotide exchange factor activity, role in regulation of Rho protein signal transduction and intracellular localization
orf19.1529	orf19.1529	1.1	0.92	0.55	0.55	-0.001314633	NA	Protein of unknown function; macrophage-induced gene
CaalfMp02	NAD6	0	0.9	0	0	-0.041439977	NA	Subunit 6 of NADH:ubiquinone oxidoreductase (NADH:ubiquinone dehydrogenase), a multisubunit enzyme complex (complex I) of the mitochondrial inner membrane that catalyzes the first step in mitochondrial respiration
orf19.7555	orf19.7555	0.9	0.9	0.45	1.35	-0.038676258	NA	Ortholog of Candida albicans WO-1 : CAWG_02247

orf19.3876	ZCF19	1.89	0.9	2.19	1.39	-0.001514458	NA	Predicted Zn(II)2Cys6 transcription factor
orf19.513	orf19.513	1.14	0.89	1.01	1.77	0.036305375	NA	Protein with a DNA-binding domain, similar to S. cerevisiae meiosis-specific transcription factor Ndt80p
orf19.2460	orf19.2460	0.71	0.89	1.6	0.36	-0.002662624	NA	Protein of unknown function; substrate for Kex2 processing in vitro; repressed by alpha pheromone in SpiderM medium; Spider biofilm induced; Bcr1-repressed in a/a RPMI biofilms
orf19.942	KRE62	0.2	0.89	1.09	0.49	-0.002497389	NA	Putative subunit of glucan synthase; macrophage-induced gene; Bcr1-regulated in a/a RPMI biofilms
orf19.6999	orf19.6999	0.88	0.88	0.88	0.29	-0.040120758	NA	Predicted oxidoreductase; rat catheter biofilm repressed
orf19.3693	PGA5	0.77	0.87	0.77	0.96	-0.001314633	NA	Putative GPI-anchored beta-1,3-glucanosyltransferase with similarity to the A. fumigatus GEL family; fungal-specific (no human or murine homolog)
orf19.4095	orf19.4095	0	0.87	0.43	0.43	-0.041439977	NA	Ortholog of C. parapsilosis CDC317 : CPAR2_407020, C. dubliniensis CD36 : Cd36_23460, Debaryomyces hansenii CBS767 : DEHA2B02002g and Pichia stipitis Pignal : PICST_31649
CaalfMp08	COX1	0.12	0.86	1.4	0	-0.002497389	NA	Subunit I of cytochrome c oxidase, which is the terminal member of the mitochondrial (mt) inner membrane electron transport chain; one of three mt-encoded subunits; alternatively spliced transcripts encode 4 putative splicing endonucleases
orf19.1577	orf19.1577	1.69	0.84	0.28	1.12	-0.074759481	NA	Has domain(s) with predicted sequence-specific DNA binding, sequence-specific DNA binding transcription factor activity, zinc ion binding activity and role in regulation of transcription, DNA-dependent

orf19.1841	orf19.1841	0.83	0.83	1.83	1	0.03497804	NA	Protein of unknown function; Hap43-induced gene
orf19.6282	orf19.6282	0.68	0.83	1.28	0.9	-0.001314633	NA	Has domain(s) with predicted nucleic acid binding, nucleotide binding activity
orf19.1105	orf19.1105	1.66	0.83	1.24	1.24	-0.037817236	NA	Ortholog of C. parapsilosis CDC317 : CPAR2_302980, C. dubliniensis CD36 : Cd36_53630, Pichia stipitis Pignal : PICST_32155 and Candida orthopsilosis Co 90-125 : CORT_0E05120
orf19.689	PLB1	1.73	0.82	1.53	1.42	-0.001514458	NA	Phospholipase B; host cell penetration and virulence in mouse systemic infection; Hog1-induced; signal sequence, N-glycosylation, and Tyr phosphorylation site; induced in fluconazole-resistant strains; rat catheter biofilm repressed
orf19.3600	orf19.3600	1.36	0.82	1.09	2.45	0.036305375	NA	Protein of unknown function; flow model biofilm induced
orf19.6824	TRY6	1.99	0.8	1.46	1.99	-0.000169167	NA	Helix-loop-helix transcription factor; regulator of yeast form adherence; required for yeast cell adherence to silicone substrate; Spider and F-12/CO2 biofilm induced; repressed by alpha pheromone in SpiderM medium
orf19.849	orf19.849	0.87	0.8	7.87	7.22	0.418281999	NA	Ortholog(s) have role in protein N-linked glycosylation, protein O-linked glycosylation, response to stress
orf19.2784	orf19.2784	2.6	0.8	1.4	2.19	-0.035127928	NA	Ortholog of C. dubliniensis CD36 : Cd36_07090, Candida tenuis NRRL Y-1498 : cten_CGOB_00237, Debaryomyces hansenii CBS767 : DEHA2F25542g and Pichia stipitis Pignal : psti_CGOB_00186
orf19.4305	orf19.4305	0.26	0.79	1.58	1.06	0.072132625	NA	Ortholog of C. dubliniensis CD36 : Cd36_52590, C. parapsilosis CDC317 : CPAR2_100565, Debaryomyces hansenii CBS767 : DEHA2G06908g and Candida guilliermondii ATCC 6260 : PGUG_02858

orf19.3926	RNY11	1.72	0.78	1.56	1.56	0.034982475	NA	Ortholog(s) have endoribonuclease activity, role in RNA catabolic process, apoptotic process, cell morphogenesis and cytosol, extracellular region, vacuole localization
orf19.3121	GST1	0	0.78	1.81	2.07	0.108762218	NA	Putative glutathione S-transferase; upregulated in the presence of human neutrophils; expression is regulated upon white-opaque switching; increased transcription is observed upon benomyl treatment
orf19.1539	orf19.1539	1.09	0.78	1.56	2.65	0.107102263	NA	Protein of unknown function; F-12/CO2 early biofilm induced
orf19.1930	CFL5	0.95	0.78	0.35	0.17	-0.079610261	NA	Ferric reductase; induced in low iron; ciclopirox olamine, flucytosine induced; amphotericin B, Sfu1 repressed; Tbf1, Hap43 induced
orf19.893	PGA17	1.22	0.78	1.55	0.77	0.03497804	NA	Putative GPI-anchored protein; exogenously expressed protein substrate for Kex2 processing in vitro; repressed by alpha pheromone in SpiderM medium; macrophage-induced; induced in oralpharyngeal candidiasis; Spider biofilm induced
orf19.4081	orf19.4081	0.39	0.78	2.34	0.78	0.072132625	NA	Dubious open reading frame
orf19.2869	orf19.2869	0	0.77	0	0.38	-0.041439977	NA	Ortholog of C. dubliniensis CD36 : Cd36_46110, Candida tropicalis MYA-3404 : CTRG_03792 and Candida albicans WO-1 : CAWG_03165
orf19.4366	orf19.4366	0.67	0.76	0	0.38	-0.079610261	NA	Ortholog of C. dubliniensis CD36 : Cd36_28870, Candida tropicalis MYA-3404 : CTRG_00731 and Candida albicans WO-1 : CAWG_01703
orf19.1239	orf19.1239	0	0.76	0.76	0	-0.002497389	NA	Secreted protein; exogenously expressed protein is a substrate for Kex2 processing in vitro; fluconazole-regulated; Spider biofilm induced

orf19.1066	GIG1	1.5	0.75	1.49	0.75	-0.001314633	NA	Protein induced by N-acetylglucosamine (GlcNAc); localized in cytoplasm; mutation causes increased resistance to nikkomycin Z
orf19.3404	orf19.3404	0.5	0.75	0.5	0.5	-0.001314633	NA	Protein of unknown function; transcription repressed by fluphenazine treatment
orf19.1651	orf19.1651	3.7	0.74	2.58	2.58	0.033294052	NA	Ortholog of C. dubliniensis CD36 : Cd36_81960, Pichia stipitis Pignal : psti_CGOB_00163, Candida tropicalis MYA-3404 : CTRG_02378 and Candida albicans WO-1 : CAWG_02525
orf19.2583.1	orf19.2583.1	1.9	0.74	0	0	-0.115838505	NA	Pseudogene; formerly an ORF Predicted by Annotation Working Group
orf19.984	PHO8	0.62	0.74	1.35	1.23	-0.001314633	NA	Putative repressible vacuolar alkaline phosphatase; Rim101-induced transcript; regulated by Tsa1, Tsa1B in minimal media at 37 deg; possibly adherence-induced
orf19.4955	orf19.4955	0.85	0.73	1.34	1.09	-0.001314633	NA	Ortholog(s) have role in ascospore wall assembly and endoplasmic reticulum, mitochondrion localization
orf19.2783	PIR32	0.15	0.73	0.59	1.17	0.036277451	NA	Cell wall protein, putative structural component of the cell wall; mutation leads to increased chitin content, increased virulence and resistance to stresses; macrophage-induced gene
orf19.3801	FAV1	0.87	0.72	0.87	0.65	-0.001314633	NA	Protein with weak similarity to S. cerevisiae Fus2p; induced by alpha pheromone mating factor in MTLa/MTLa opaque cells
orf19.3888.2	orf19.3888.2	0	0.72	0	0	-0.041439977	NA	Dubious open reading frame

orf19.2624	orf19.2624	0.61	0.71	0.51	0.51	-0.001314633	NA	Protein of unknown function; downregulated by fluphenazine treatment or in an azole-resistant strain that overexpresses CDR1 and CDR2
orf19.555	orf19.555	1.61	0.7	0.23	1.6	-0.035585767	NA	Protein similar to S. cerevisiae Cis3p, which has a role cell-wall-related processes; predicted Kex2p substrate; possibly an essential gene, disruptants not obtained by UAU1 method
orf19.2638	orf19.2638	0.92	0.69	2.52	2.52	0.140902086	NA	Protein of unknown function; Spider biofilm induced
orf19.1827	orf19.1827	0.55	0.69	0.69	1.23	-0.001314633	NA	Protein of unknown function; flow model biofilm induced; induced by alpha pheromone in SpiderM medium
orf19.3935	orf19.3935	0	0.68	0	0	-0.041439977	NA	Ortholog of Candida albicans WO-1 : CAWG_04819
orf19.5896	orf19.5896	1.13	0.68	0.68	0.9	-0.001314633	NA	Protein of unknown function; induced by alpha pheromone in SpiderM medium
orf19.2766	orf19.2766	0.67	0.67	0	0	-0.079610261	NA	Dubious open reading frame
orf19.2657	orf19.2657	1.32	0.67	1.45	1.12	-0.001314633	NA	Protein of unknown function
orf19.5701	orf19.5701	3.28	0.67	1.76	1.51	-0.000397909	NA	Ortholog(s) have role in DNA replication initiation, establishment of mitotic sister chromatid cohesion and condensed nuclear chromosome kinetochore localization

orf19.7208	CSK1	0	0.67	0	0.17	-0.041439977	NA	Putative mitogen-activated protein (MAP) kinase with an unknown role; null mutant produces wrinkled colonies; similar to S. cerevisiae Smk1p, which is a protein kinase required for sporulation
orf19.5556	orf19.5556	0.67	0.67	0.66	0.66	-0.001314633	NA	Ortholog of Candida albicans WO-1 : CAWG_05078
orf19.3968	orf19.3968	0	0.66	1.31	0	-0.002497389	NA	Protein of unknown function; flow model biofilm induced
orf19.1821	orf19.1821	0	0.66	0	0	-0.041439977	NA	Protein of unknown function; repressed by alpha pheromone in SpiderM medium
orf19.584	orf19.584	0.87	0.65	0.43	1.29	-0.038676258	NA	Has domain(s) with predicted heme binding, peroxidase activity and role in oxidation-reduction process, response to oxidative stress
orf19.1171	BTA1	0.57	0.65	0.24	0.57	-0.038676258	NA	Ortholog of C. dubliniensis CD36 : Cd36_10780, C. parapsilosis CDC317 : CPAR2_208080, Candida tenuis NRRL Y-1498 : CANTEDRAFT_120956 and Debaryomyces hansenii CBS767 : DEHA2F24508g
orf19.1117	orf19.1117	1.44	0.64	1.31	1.57	0.036305375	NA	Protein similar to Candida boidinii formate dehydrogenase; virulence-group-correlated expression; Hap43-repressed; Spider biofilm repressed
orf19.5606	orf19.5606	1.91	0.64	2.54	1.91	0.069049269	NA	Ortholog of Candida albicans WO-1 : CAWG_05051
orf19.1005	orf19.1005	1.85	0.62	1.74	1.43	-0.001514458	NA	Protein with predicted RNI-like domains; rat catheter biofilm induced

orf19.7192	orf19.7192	0	0.62	0	0	-0.041439977	NA	Ortholog of Candida albicans WO-1 : CAWG_05720
orf19.4653	orf19.4653	3.09	0.62	0	0	-0.150201915	NA	Protein similar to GPI-linked cell-wall proteins; induced in low iron; Spider biofilm induced; regulated in Spider biofilms by Bcr1, Tec1, Ndt80, Brg1
orf19.2922	orf19.2922	1.56	0.62	0.93	2.49	-0.000169167	NA	Ortholog(s) have double-stranded DNA binding activity, role in reciprocal meiotic recombination, synapsis and condensed nuclear chromosome localization
orf19.4654	orf19.4654	1.24	0.62	0	0.62	-0.038676258	NA	Protein of unknown function; Spider biofilm induced
orf19.6911	orf19.6911	0	0.62	0	0	-0.041439977	NA	Ortholog of Candida albicans WO-1 : CAWG_05482
orf19.7055	orf19.7055	1.87	0.62	1.25	1.25	-0.037817236	NA	Dubious open reading frame
orf19.5231	orf19.5231	0.76	0.61	0.15	0.45	-0.079610261	NA	Protein of unknown function; flow model biofilm induced
orf19.1062	orf19.1062	0.76	0.61	0.3	1.21	-0.038676258	NA	Ortholog of C. dubliniensis CD36 : Cd36_04020, C. parapsilosis CDC317 : CPAR2_107000, Candida tenuis NRRL Y-1498 : CANTEDRAFT_113990 and Pichia stipitis Pignal : PICST_32780
orf19.1679	orf19.1679	0.41	0.61	1.01	0.41	-0.002497389	NA	Ortholog of C. dubliniensis CD36 : Cd36_81550, C. parapsilosis CDC317 : CPAR2_503570, Candida tenuis NRRL Y-1498 : cten_CGOB_00261 and Debaryomyces hansenii CBS767 : DEHA2D15378g

orf19.5092	orf19.5092	0.6	0.6	1.2	2.4	0.036305375	NA	Ortholog of C. parapsilosis CDC317 : CPAR2_207660.1, C. dubliniensis CD36 : Cd36_07680, Candida tenuis NRRL Y-1498 : CANTEDRAFT_113324 and Debaryomyces hansenii CBS767 : DEHA2C11088g
orf19.6138	orf19.6138	0.67	0.59	0.92	1.18	-0.001314633	NA	Protein similar to ferric reductase Fre10p; possibly an essential gene, disruptants not obtained by UAU1 method
orf19.2025	orf19.2025	0	0.59	0	0	-0.041439977	NA	Dubious open reading frame
orf19.1366	orf19.1366	0.59	0.59	0.89	0.74	-0.001314633	NA	Has domain(s) with predicted oxidoreductase activity, acting on paired donors, with incorporation or reduction of molecular oxygen, 2-oxoglutarate as one donor, and incorporation of one atom each of oxygen into both donors activity
orf19.1302	orf19.1302	0	0.59	0.29	0	-0.041439977	NA	Ortholog of C. dubliniensis CD36 : Cd36_43500, Spathaspora passalidarum NRRL Y-27907 : SPAPADRAFT_64619, Candida tropicalis MYA-3404 : CTRG_00335 and Candida albicans WO-1 : CAWG_03437
orf19.6200	orf19.6200	0.79	0.59	2.57	1.97	0.105912539	NA	Pry family pathogenesis-related protein; oral infection upregulated gene; mutant has reduced capacity to damage oral epithelial cells
CaalfMp11.1	CaalfMp11.1	1.56	0.58	0.26	0.9	-0.074759481	NA	Putative endonuclease encoded by the first exon and part of the first intron of the mitochondrial COB gene; C-terminus contains a GIY-YIG domain found in intron-encoded endonucleases; conserved in fungal mitochondria
orf19.5549	orf19.5549	1.13	0.57	2.26	0.56	0.03497804	NA	Ortholog of C. dubliniensis CD36 : Cd36_63380 and Candida albicans WO-1 : CAWG_05071
orf19.3868	orf19.3868	0	0.57	0	0.57	-0.001075962	NA	Dubious open reading frame

orf19.599	orf19.599	0	0.56	0	0	-0.041439977	NA	Dubious open reading frame
orf19.5592	orf19.5592	0.55	0.55	0.55	0.37	-0.040120758	NA	Ortholog of C. dubliniensis CD36 : Cd36_63980, Candida tenuis NRRL Y-1498 : CANTEDRAFT_115187, Candida tropicalis MYA-3404 : CTRG_02719 and Candida albicans WO-1 : CAWG_05060
orf19.7440	HST6	0.74	0.55	1.11	0.6	-0.001314633	NA	ABC transporter related to mammalian P-glycoproteins; functional homolog of S. cerevisiae Ste6p (a-pheromone transporter); required for mating of MTLa cells; a-type specific, not regulated during white-opaque or yeast-hyphal switching
orf19.5524	orf19.5524	0.81	0.54	0.27	2.97	0.036426979	NA	Ortholog of C. dubliniensis CD36 : Cd36_62790, C. parapsilosis CDC317 : CPAR2_601680, Candida tenuis NRRL Y-1498 : cten_CGOB_00252 and Debaryomyces hansenii CBS767 : DEHA2A06622g
orf19.2430	orf19.2430	0	0.53	0	0	-0.041439977	NA	Ortholog of Candida albicans WO-1 : CAWG_00792
orf19.696	STE2	0.66	0.53	0.79	0.39	-0.040120758	NA	Receptor for alpha factor mating pheromone, MFalpha; required for a-type cells to respond to alpha factor, for opaque a-form mating and white a-form response; possible Kex2p substrate; a-specific, alpha-factor induced, A1p-Alpha2p repressed
orf19.3120	orf19.3120	0.85	0.53	0.21	0.53	-0.038676258	NA	PDR-subfamily ABC transporter (half-size); similar to WHITE subfamily proteins; repressed by fluphenazine treatment or in an azole-resistant strain that overexpresses CDR1 and CDR2; induced by nitric oxide; rat catheter biofilm induced
orf19.4822	LIP10	0.13	0.53	0.13	0	-0.041439977	NA	Secreted lipase, member of a lipase gene family whose members are expressed differentially in response to carbon source and during infection; may have a role in nutrition and/or in creating an acidic microenvironment
orf19.1148	orf19.1148	0.96	0.48	0	0.72	0.000483364	NA	Ortholog of C. dubliniensis CD36 : Cd36_11020 and Candida albicans WO-1 : CAWG_00266

orf19.6222	SPO22	0.46	0.46	0.65	1.36	0.077533458	NA	Ortholog(s) have role in regulation of synaptonemal complex assembly and condensed nuclear chromosome localization
orf19.322	orf19.322	0.46	0.46	1.36	0	0.038199476	NA	Ortholog of Candida albicans WO-1 : CAWG_02670
orf19.2503	orf19.2503	0.45	0.45	0.45	0.45	-0.000712397	NA	Ortholog of S. cerevisiae : YGL138C, C. glabrata CBS138 : CAGL0H08910g, C. dubliniensis CD36 : Cd36_80910, C. parapsilosis CDC317 : CPAR2_102230 and Candida tenuis NRRL Y-1498 : CANTEDRAFT_109984
orf19.3134	orf19.3134	0	0.45	0.45	0.89	0.039633123	NA	Protein of unknown function; flow model biofilm induced
orf19.654	orf19.654	0	0.45	0.23	0	-0.000712397	NA	Predicted protein only found in C. albicans and C. dubliniensis; highly induced during chlamydospore development
orf19.1935	orf19.1935	0	0.44	0	0	-0.000712397	NA	Dubious open reading frame
orf19.5874	orf19.5874	1.28	0.43	0.42	1.7	0.03842318	NA	Ortholog of C. dubliniensis CD36 : Cd36_84250, C. parapsilosis CDC317 : CPAR2_806160, Candida tenuis NRRL Y-1498 : CANTEDRAFT_137075 and Debaryomyces hansenii CBS767 : DEHA2F11462g
orf19.6405	orf19.6405	0	0.42	0.63	0.63	0.077533458	NA	Protein of unknown function; decreased transcription is observed in an azole-resistant strain that overexpresses MDR1
orf19.6859	orf19.6859	0.42	0.41	0	2.07	0.077311135	NA	Ortholog of C. dubliniensis CD36 : Cd36_44860, Candida tropicalis MYA-3404 : CTRG_04054 and Candida albicans WO-1 : CAWG_03295

orf19.4094	orf19.4094	0	0.4	0	0	-0.000712397	NA	Ortholog of C. dubliniensis CD36 : Cd36_23450, C. parapsilosis CDC317 : CPAR2_407010, Candida tenuis NRRL Y-1498 : cten_CGOB_00249 and Debaryomyces hansenii CBS767 : DEHA2B01980g
orf19.2583	PTR2	0.2	0.4	0.1	0.2	-0.000712397	NA	Oligopeptide transporter involved in uptake of di-/tripeptides; highly induced during chlamydospore formation in both C. albicans and C. dubliniensis
orf19.1620	orf19.1620	0	0.4	1.6	1.2	0.113513483	NA	Protein of unknown function; induced by alpha pheromone in SpiderM medium
orf19.4884	WOR1	0.23	0.39	14.91	15.92	0.915453081	NA	Transcription factor ("master switch") of white-opaque phenotypic switching; required to establish and maintain the opaque state; opaque-specific, nuclear; regulates its own expression; suggested role in regulation of adhesion factors
orf19.4067	FGR18	0.38	0.38	0.76	0.38	0.038199476	NA	Protein lacking an ortholog in S. cerevisiae; transposon mutation affects filamentous growth
orf19.6277	orf19.6277	0.49	0.38	0.33	0.33	-0.000712397	NA	Has domain(s) with predicted phosphatidylinositol binding activity and role in cell communication
orf19.1958	orf19.1958	0.77	0.38	1.15	2.29	0.075064834	NA	Dubious open reading frame
orf19.3376	orf19.3376	0.74	0.37	0.37	0.74	0.000483364	NA	Ortholog of Candida albicans WO-1 : CAWG_03459
orf19.3638	PGA46	0.37	0.37	0.92	0.37	0.038199476	NA	Putative GPI-anchored protein; secreted; fluconazole-induced

orf19.1920	orf19.1920	0	0.36	1.09	0.36	0.038199476	NA	Ortholog of C. dubliniensis CD36 : Cd36_15040
orf19.7009	orf19.7009	0.72	0.36	1.98	1.44	0.073747334	NA	Has domain(s) with predicted catalytic activity, coenzyme binding activity and role in cellular metabolic process
orf19.3378	orf19.3378	0.35	0.35	1.76	2.11	0.150577902	NA	Protein of unknown function; regulated by Tsa1, Tsa1B in minimal media at 37 degrees C
orf19.3494	CTF5	1.19	0.34	1.02	1.02	0.03785961	NA	Predicted component of the kinetochore sub-complex COMA; induced during the mating process; repressed by alpha pheromone in SpiderM medium
orf19.2392	orf19.2392	0.38	0.33	9.42	7.46	0.528065025	NA	Ortholog of C. dubliniensis CD36 : Cd36_28460, Debaryomyces hansenii CBS767 : DEHA2B00704g, Pichia stipitis Pignal : PICST_43964 and Spathaspora passalidarum NRRL Y-27907 : spas_CGOB_00057
orf19.2478	orf19.2478	0.82	0.33	1.47	1.47	0.03785961	NA	Ortholog of C. dubliniensis CD36 : Cd36_05410, C. parapsilosis CDC317 : CPAR2_107480, Candida tenuis NRRL Y-1498 : CANTEDRAFT_94695 and Debaryomyces hansenii CBS767 : DEHA2D02288g
orf19.2585	orf19.2585	0	0.33	0	0	-0.000712397	NA	Ortholog of Candida albicans WO-1 : CAWG_01548
orf19.701	CFL11	0.58	0.33	0.42	0.66	0.000483364	NA	Protein similar to ferric reductase Fre10p; flucytosine repressed; possibly adherence-induced; possibly an essential gene, disruptants not obtained by UAU1 method; rat catheter biofilm repressed
orf19.2160	NAG4	0.53	0.32	0.32	0	-0.03986449	NA	Putative transporter; fungal-specific; similar to Nag3p and to S. cerevisiae Ypr156Cp and Ygr138Cp; required for wild-type mouse virulence and wild-type cycloheximide resistance; gene cluster encodes enzymes of GlcNAc catabolism

orf19.7480	orf19.7480	0.32	0.32	1.61	0.64	0.113513483	NA	Putative protein of unknown function; transcript is upregulated in clinical isolates from HIV+ patients with oral candidiasis
orf19.3200	MTLA2	1.91	0.32	0.64	0.32	-0.037783556	NA	Master regulator (activator) of a-type mating; has HMG domain, which is predicted to bind DNA; gene has intron; not related to S. cerevisiae MATa2; a/alpha mating type may increase virulence, provides competitive advantage
orf19.6142	orf19.6142	0	0.32	0	0.64	0.039633123	NA	Ortholog(s) have double-stranded DNA binding activity, role in reciprocal meiotic recombination and condensed nuclear chromosome localization
orf19.2449	orf19.2449	0.21	0.31	0	0.1	-0.000712397	NA	Putative adhesin-like protein; macrophage-induced gene
orf19.5057	orf19.5057	1.64	0.3	0.74	1.19	0.000222293	NA	Ortholog of C. dubliniensis CD36 : Cd36_07380 and Candida albicans WO-1 : CAWG_00634
orf19.3897	orf19.3897	0	0.3	0	0	-0.000712397	NA	Protein of unknown function; decreased transcription is observed upon fluphenazine treatment or in an azole-resistant strain that overexpresses CDR1 and CDR2; transcription is repressed in response to alpha pheromone in SpiderM medium
orf19.2169	orf19.2169	0.28	0.28	0	0.55	0.039633123	NA	Ortholog of C. parapsilosis CDC317 : CPAR2_805720, C. dubliniensis CD36 : Cd36_22330, Lodderomyces elongisporus NRLL YB-4239 : LELG_02628 and Candida orthopsilosis Co 90-125 : CORT_0C03440
orf19.1874	orf19.1874	0.56	0.28	0.56	0.97	0.03785961	NA	Ortholog(s) have protein serine/threonine kinase activity, role in meiosis, meiotic recombination checkpoint, protein phosphorylation, regulation of linear element assembly and cytosol, linear element localization
orf19.5256	orf19.5256	0.69	0.28	1.24	0.82	0.03785961	NA	Protein of unknown function; Hap43-repressed gene; induced by alpha pheromone in SpiderM medium

orf19.7330	orf19.7330	0.27	0.27	0	0.53	0.039633123	NA	Protein with a predicted heme oxygenase domain; Spider biofilm induced
orf19.664	orf19.664	0.26	0.26	0.52	0.52	0.077533458	NA	Ortholog of C. dubliniensis CD36 : Cd36_10680, Debaryomyces hansenii CBS767 : DEHA2E14058g, Pichia stipitis Pignal : PICST_29620 and Spathaspora passalidarum NRRL Y-27907 : SPAPADRAFT_146037
orf19.2847.1	orf19.2847.1	2.73	0.25	1.7	0.49	-0.035709712	NA	Pseudogene; formerly an ORF Predicted by Annotation Working Group that was subsequently removed from Assembly 20
orf19.5588	PGA60	0.33	0.25	0.66	0.58	0.077533458	NA	Putative GPI-anchored adhesin-like protein; hyphal-induced expression
orf19.120	orf19.120	1.97	0.25	2.21	1.96	0.071770179	NA	Has domain(s) with predicted protein C-terminal S-isoprenylcysteine carboxyl O-methyltransferase activity, role in C-terminal protein methylation and integral to membrane localization
orf19.2332	orf19.2332	0	0.24	0	0	-0.000712397	NA	Ortholog of C. parapsilosis CDC317 : CPAR2_210560, C. dubliniensis CD36 : Cd36_10180, Candida orthopsilosis Co 90-125 : CORT_0A09890 and Candida tropicalis MYA-3404 : CTRG_04072
orf19.2237	SPR1	0.35	0.24	0	0	-0.000712397	NA	Putative GPI-anchored protein; similar but not orthologous to S. cerevisiae Spr1, a sporulation-specific exo-1,3-beta-glucanase; mutant is viable
orf19.7167	orf19.7167	0	0.24	0	0	-0.000712397	NA	Putative adhesin-like protein
orf19.6520	orf19.6520	0.11	0.22	0.33	0.22	-0.000712397	NA	Putative allantoate permease; fungal-specific (no human or murine homolog)

orf19.2804	orf19.2804	0	0.22	0	0	-0.000712397	NA	Ortholog of C. dubliniensis CD36 : Cd36_83980, C. parapsilosis CDC317 : CPAR2_102620, Candida tenuis NRRL Y-1498 : CANTEDRAFT_134550 and Debaryomyces hansenii CBS767 : DEHA2D12100g
orf19.634	orf19.634	0.22	0.22	0.22	0.22	-0.000712397	NA	Ortholog of C. dubliniensis CD36 : Cd36_30370, Candida tropicalis MYA-3404 : CTRG_00536 and Candida albicans WO-1 : CAWG_01831
orf19.6302	PGA39	0	0.22	0	0.22	-0.000712397	NA	Putative GPI-anchored protein
orf19.6484	orf19.6484	0.86	0.21	0	0.86	0.000483364	NA	Ortholog of C. parapsilosis CDC317 : CPAR2_808370, C. dubliniensis CD36 : Cd36_72070, Candida orthopsilosis Co 90-125 : CORT_0C00800 and Candida albicans WO-1 : CAWG_05577
orf19.6486	LDG3	0	0.21	0	0	-0.000712397	NA	Putative LDG family protein; F-12/CO2 early biofilm induced
orf19.4789	orf19.4789	1.27	0.21	0.85	0.21	-0.000937989	NA	Has domain(s) with predicted metal ion binding activity
orf19.4996	orf19.4996	0.42	0.21	1.9	2.32	0.150577902	NA	Ortholog(s) have cytoplasm, nucleus localization
CaalfMp08.2	CaalfMp08.2	0	0.2	0.21	0	-0.000712397	NA	Putative endonuclease encoded by the first two exons and part of the second intron of the mitochondrial COX1 gene; C-terminus contains a LAGLIDADG domain found in intron-encoded endonucleases; conserved in fungal mitochondria
orf19.1824	PGA50	0	0.2	0	0.2	-0.000712397	NA	Putative GPI-anchored protein; adhesin-like protein

CaalfMp08.3	CaalfMp08.3	0.11	0.19	0	0.33	-0.000712397	NA	Putative endonuclease encoded by the first three exons and part of the third intron of the mitochondrial COX1 gene; C-terminus contains a LAGLIDADG domain found in intron-encoded endonucleases; conserved in fungal mitochondria
orf19.776	orf19.776	0.19	0.19	0.29	0.48	-0.000712397	NA	Ortholog(s) have role in meiotic sister chromatid cohesion, protein localization to chromosome, centromeric region, reciprocal meiotic recombination, synaptonemal complex assembly
orf19.3760	DLH1	0.58	0.19	0.97	0.19	-0.000937989	NA	Functional homolog of S. cerevisiae Dmc1; a meiosis-specific protein required for repair of double-strand breaks and pairing between homologous chromosomes
orf19.4463	orf19.4463	0.35	0.18	0.18	0.88	0.039633123	NA	Ortholog of Candida albicans WO-1 : CAWG_01004
orf19.5479	FGR12	0	0.16	0	0.16	-0.000712397	NA	Protein lacking an ortholog in S. cerevisiae; transposon mutation affects filamentous growth; flow model biofilm induced
orf19.3735	orf19.3735	0.15	0.15	0.3	0.3	-0.000712397	NA	Protein of unknown function; induced by alpha pheromone in SpiderM medium
orf19.2787	PRY1	0.15	0.15	0.3	0	-0.000712397	NA	Pry family pathogenesis-related protein; extracellular; opaque specific transcript; repressed by alpha pheromone in SpiderM medium; possibly essential, disruptants not obtained by UAU1 method; Spider biofilm induced
orf19.7013	orf19.7013	0.3	0.15	0.3	0.15	-0.000712397	NA	Ortholog of C. dubliniensis CD36 : Cd36_70940, C. parapsilosis CDC317 : CPAR2_300280, Debaryomyces hansenii CBS767 : DEHA2F22220g and Pichia stipitis Pignal : PICST_32459
CaalfMp08.1	CaalfMp08.1	0.12	0.14	0	0	-0.000712397	NA	Putative endonuclease encoded by the first exon and part of the first intron of the mitochondrial COX1 gene; C-terminus contains a LAGLIDADG domain found in intron-encoded endonucleases; conserved in fungal mitochondria

CaalfMp14	NAD4	0	0.13	0.67	0	0.038199476	NA	Subunit 4 of NADH:ubiquinone oxidoreductase (NADH:ubiquinone dehydrogenase), a multisubunit enzyme complex (complex I) of the mitochondrial inner membrane that catalyzes the first step in mitochondrial respiration
orf19.2197	orf19.2197	0.12	0.12	0.37	0.49	-0.000712397	NA	Has domain(s) with predicted N,N-dimethylaniline monooxygenase activity, NADP binding, flavin adenine dinucleotide binding activity and role in oxidation-reduction process
CaalfMp15	COX3B	0	0.12	0.23	0	-0.000712397	NA	Subunit III of cytochrome c oxidase, which is the terminal member of the mitochondrial inner membrane electron transport chain; identical to Cox3Ap; one of three mitochondrially-encoded subunits
orf19.2252	orf19.2252	0.24	0.12	0	0.17	-0.000712397	NA	Protein of unknown function; rat catheter biofilm repressed
CaalfMp04	COX3A	0	0.12	0.23	0	-0.000712397	NA	Subunit III of cytochrome c oxidase, which is the terminal member of the mitochondrial inner membrane electron transport chain; identical to Cox3Bp; one of three mitochondrially-encoded subunits
CaalfMp13	NAD5	0	0.11	0.11	0	-0.000712397	NA	Subunit 5 of NADH:ubiquinone oxidoreductase (NADH:ubiquinone dehydrogenase), a multisubunit enzyme complex (complex I) of the mitochondrial inner membrane that catalyzes the first step in mitochondrial respiration
orf19.7336	orf19.7336	0	0.1	0	0.1	-0.000712397	NA	Predicted membrane transporter; member of the drug:proton antiporter (14 spanner) (DHA2) family, major facilitator superfamily (MFS); Spider biofilm induced
orf19.3380	HWP2	0	0.07	0	0	-0.000712397	NA	GPI-anchored, glycosylated cell wall protein; required for biofilm formation, adhesion, filamentous growth on some media; expressed in hyphae; mutant delayed in virulence; regulated by Efg1, Tup1; similar to Hwp1 and Rbt1 domains
orf19.2366	orf19.2366	0.21	0.07	0.07	0.07	-0.000712397	NA	Has domain(s) with predicted DNA binding activity and role in DNA integration

orf19.7278	orf19.7278	0	0.04	0	5.19	0.176897487	NA	Similar to a region of the Tca2 (pCal) retrotransposon, which is present in strain hOG1042 as 50 to 100 copies of a linear dsDNA; rat catheter biofilm repressed
orf19.3637	orf19.3637	0.64	0	0.64	0	-0.000937989	NA	Dubious open reading frame
orf19.5303	PGA30	0	0	0.23	0	-0.000712397	NA	GPI-anchored protein of cell wall
orf19.2505	orf19.2505	0	0	2.66	0	0.108629703	NA	Ortholog of Candida albicans WO-1 : CAWG_02437
orf19.7071	FGR2	0.12	0	0	0	-0.000712397	NA	Protein similar to phosphate transporters; transposon mutation affects filamentous growth; expression is regulated upon white-opaque switching
orf19.2833	PGA34	0	0	1.62	0.32	0.074561963	NA	Putative GPI-anchored protein; transcript repressed by alpha pheromone in SpiderM medium; induced in oralpharyngeal candidasis; flow model biofilm induced; Spider biofilm induced
orf19.1450	orf19.1450	0.17	0	0.17	0.17	-0.000712397	NA	Ortholog of C. dubliniensis CD36 : Cd36_16320, Candida tropicalis MYA-3404 : CTRG_01238 and Candida albicans WO-1 : CAWG_03918
orf19.3109	orf19.3109	0.62	0	0.61	0	-0.000937989	NA	Ortholog of C. dubliniensis CD36 : Cd36_46470, C. parapsilosis CDC317 : CPAR2_500900, Debaryomyces hansenii CBS767 : DEHA2B15730g and Candida tropicalis MYA-3404 : CTRG_03755
orf19.6491	orf19.6491	0.57	0	0.57	0	-0.000937989	NA	Ortholog of Candida albicans WO-1 : CAWG_05572

orf19.1348	orf19.1348	0	0	0.34	1.03	0.039633123	NA	Ortholog of C. dubliniensis CD36 : Cd36_22500, Candida tropicalis MYA-3404 : CTRG_01825 and Candida albicans WO-1 : CAWG_05921
orf19.7397.3	orf19.7397.3	0	0	0	0.3	-0.000712397	NA	Predicted ORF identical to orf19.7420
orf19.1524	SPR3	0.13	0	0.25	0.13	-0.000712397	NA	Septin; mutant has no obvious phenotype
orf19.1370	orf19.1370	0	0	0.66	0	0.038199476	NA	Protein of unknown function; expression regulated by white-opaque switch; repressed by alpha pheromone in SpiderM medium
orf19.3907	orf19.3907	0	0	0.66	0	0.038199476	NA	Ortholog of Candida albicans WO-1 : CAWG_04797
orf19.5262	orf19.5262	0.46	0	0	0	-0.000712397	NA	Ortholog of C. dubliniensis CD36 : Cd36_11270 and Candida albicans WO-1 : CAWG_00238
orf19.4196	orf19.4196	0.61	0	1.22	0	-0.000937989	NA	Dubious open reading frame
orf19.5302	PGA31	0	0	1.95	4.96	0.251701174	NA	Cell wall protein; putative GPI anchor; expression regulated upon white-opaque switch; induced by Congo Red and cell wall regeneration; Bcr1-repressed in RPMI a/a biofilms
orf19.7395	orf19.7395	0	0	0	0.64	0.039633123	NA	Ortholog of Candida albicans WO-1 : CAWG_02922

CaalfMp03	NAD1	0	0	0.4	0.2	-0.000712397	NA	Subunit 6 of NADH:ubiquinone oxidoreductase (NADH:ubiquinone dehydrogenase), a multisubunit enzyme complex (complex I) of the mitochondrial inner membrane that catalyzes the first step in mitochondrial respiration
orf19.4803	orf19.4803	0.53	0	0.53	0	-0.000937989	NA	Ortholog of Candida albicans WO-1 : CAWG_00488
orf19.6488	orf19.6488	0	0	0.11	0	-0.000712397	NA	Ortholog of C. dubliniensis CD36 : Cd36_72050, C. parapsilosis CDC317 : CPAR2_301140, Candida tenuis NRRL Y-1498 : CANTEDRAFT_135055 and Debaryomyces hansenii CBS767 : DEHA2E07678g
orf19.4461	orf19.4461	0	0	0	0.55	0.039633123	NA	Ortholog of Candida albicans WO-1 : CAWG_01005
CaalfMp10	NAD3	0	0	0.51	0	0.038199476	NA	Subunit 3 of NADH:ubiquinone oxidoreductase (NADH:ubiquinone dehydrogenase), a multisubunit enzyme complex (complex I) of the mitochondrial inner membrane that catalyzes the first step in mitochondrial respiration
orf19.4804	LIP2	0.67	0	0	0.13	-0.03986449	NA	Secreted lipase; member of a differentially expressed lipase gene family; expressed in alimentary tract, but not oral tissue, during mouse oral infection; may have a role in nutrition and/or in creating an acidic microenvironment
orf19.3905	MRV5	0.3	0	0	0	-0.000712397	NA	Planktonic growth-induced gene
orf19.4972	orf19.4972	0.38	0	0.76	0.95	0.077533458	NA	Putative transcription factor with zinc finger DNA-binding motif
orf19.936	orf19.936	0.4	0	0.32	0.4	-0.000712397	NA	Ortholog of C. dubliniensis CD36 : Cd36_50530, C. parapsilosis CDC317 : CPAR2_303990, Candida tenuis NRRL Y-1498 : CANTEDRAFT_107934 and Pichia stipitis Pignal : PICST_63283

orf19.77.1	orf19.77.1	0	0	0.84	0	0.038199476	NA	Protein of unknown function; Hap43-repressed gene
orf19.7002	orf19.7002	0.47	0	0.16	0.16	-0.000712397	NA	Ortholog of C. parapsilosis CDC317 : CPAR2_806990, Candida tenuis NRRL Y-1498 : CANTEDRAFT_122709, Debaryomyces hansenii CBS767 : DEHA2E00110g and Pichia stipitis Pignal : PICST_30109
orf19.7464	orf19.7464	0	0	0	0.33	-0.000712397	NA	Putative subtilisin-family protease; mutation confers hypersensitivity to toxic ergosterol analog
orf19.2203	orf19.2203	0	0	0.64	1.29	0.077533458	NA	Ortholog of Candida albicans WO-1 : CAWG_05873
orf19.1671.1	orf19.1671.1	0	0	0	0.52	0.039633123	NA	Predicted ORF added to Assembly 21 based on sequence re-analysis
orf19.5866	orf19.5866	0.63	0	0.63	0.63	0.03785961	NA	Ortholog of Candida albicans WO-1 : CAWG_02737
orf19.6450	orf19.6450	0.28	0	0	0	-0.000712397	NA	Protein of unknown function
orf19.553	orf19.553	0	0	0	0.12	-0.000712397	NA	Predicted membrane transporter, member of the drug:proton antiporter (12 spanner) (DHA1) family, major facilitator superfamily (MFS)
orf19.1571	ATO7	0.24	0	0	0.24	-0.000712397	NA	Putative fungal-specific transmembrane protein

orf19.2212	orf19.2212	5.16	0	3.86	8.35	0.208102603	NA	Ortholog of C. dubliniensis CD36 : Cd36_21980, Debaryomyces hansenii CBS767 : DEHA2F19734g, Pichia stipitis Pignal : psti_CGOB_00208 and Spathaspora passalidarum NRRL Y-27907 : spas_CGOB_00029
orf19.4214	orf19.4214	1.99	0	0.99	0.99	0.000222293	NA	Ortholog of Candida albicans WO-1 : CAWG_05313
orf19.4685	orf19.4685	0	0	1.29	1.29	0.077533458	NA	Dubious open reading frame
orf19.5499	orf19.5499	0.13	0	0.13	0.26	-0.000712397	NA	Ortholog(s) have 4-hydroxyphenylpyruvate dioxygenase activity and role in aromatic amino acid family biosynthetic process
orf19.2916	orf19.2916	0.28	0	0.28	0.56	0.039633123	NA	Ortholog of C. dubliniensis CD36 : Cd36_45740, C. parapsilosis CDC317 : CPAR2_401900, Candida tenuis NRRL Y-1498 : CANTEDRAFT_116046 and Pichia stipitis Pignal : PICST_28890
orf19.1008	orf19.1008	1.3	0	3.25	1.95	0.144353806	NA	Ortholog of Candida albicans WO-1 : CAWG_01856
orf19.4189	orf19.4189	0.17	0	0.52	0	0.038199476	NA	Has domain(s) with predicted hydrolase activity
orf19.7597	PGA12	0	0	0.6	0	0.038199476	NA	Putative GPI-anchored protein
orf19.1530	orf19.1530	0	0	0.37	0.75	0.039633123	NA	Protein of unknown function

orf19.3117	CSA2	0.45	0	0	0	-0.000712397	NA	Extracellular-associated protein; repressed by Rim101 at pH 8; regulated by Tsa1, Tsa1B in minimal media at 37 deg; induced by ketoconazole, nitric oxide, Hap43; required for normal RPMI biofilm formation; Bcr1 induced in RPMI
orf19.848	PGA16	0.63	0	0	0	-0.03986449	NA	Putative GPI-anchored protein
orf19.2211	orf19.2211	0	0	1.12	0.22	0.038199476	NA	Ortholog of C. dubliniensis CD36 : Cd36_21990, C. parapsilosis CDC317 : CPAR2_103660, Debaryomyces hansenii CBS767 : DEHA2F19756g and Pichia stipitis Pignal : PICST_61375
orf19.3671	orf19.3671	0	0	0	0.59	0.039633123	NA	Dubious open reading frame
orf19.2959	orf19.2959	0	0	0	0.68	0.039633123	NA	Predicted ORF from Assembly 19; removed from Assembly 20; subsequently reinstated in Assembly 21 based on comparative genome analysis
orf19.3375	orf19.3375	0.33	0	0	0	-0.000712397	NA	Ortholog of C. dubliniensis CD36 : Cd36_43270 and Candida albicans WO-1 : CAWG_03460
orf19.7608	orf19.7608	0	0	0	0.5	0.039633123	NA	Protein of unknown function; fluconazole-repressed; Spider biofilm induced
orf19.3336	orf19.3336	0	0	0.43	0.43	-0.000712397	NA	Ortholog of Candida albicans WO-1 : CAWG_01228
orf19.653	orf19.653	1.25	0	0.62	0.62	0.03785961	NA	Dubious open reading frame

orf19.5402	orf19.5402	4.2	0	1.84	3.78	0.067135232	NA	Ortholog of Candida albicans WO-1 : CAWG_02394
orf19.3049	orf19.3049	0.66	0	0.4	0.53	0.000483364	NA	Ortholog(s) have protein kinase activity, role in activation of bipolar cell growth, ascospore wall assembly, protein phosphorylation and cell division site, cytosol, nucleus, prospore membrane localization
orf19.4179	orf19.4179	0.73	0	0.73	1.1	0.03785961	NA	Putative adhesin-like protein
orf19.2725	orf19.2725	2.04	0	1.36	0.68	0.000222293	NA	Ortholog of Candida albicans WO-1 : CAWG_03525
orf19.4528.1	orf19.4528.1	0	0	0	1.49	0.039633123	NA	Ortholog of C. dubliniensis CD36 : Cd36_01830
orf19.3229	orf19.3229	0	0	0.67	0.67	0.077533458	NA	Protein of unknown function; transcript detected on high-resolution tiling arrays
orf19.2475	PGA26	0.51	0	1.01	0	-0.000937989	NA	GPI-anchored adhesin-like protein of the cell wall; role in cell wall integrity; required for normal virulence; induced in high iron and during cell wall regeneration; Hap43-repressed
orf19.5306	orf19.5306	0	0	0	0.48	-0.000712397	NA	Ortholog of C. dubliniensis CD36 : Cd36_43820 and Candida albicans WO-1 : CAWG_03393
orf19.2034	orf19.2034	0	0	0.55	0	0.038199476	NA	Ortholog of C. parapsilosis CDC317 : CPAR2_212925, C. dubliniensis CD36 : Cd36_15840, Candida orthopsilosis Co 90-125 : CORT_0A12230 and Candida tropicalis MYA-3404 : CTRG_01156

orf19.635	orf19.635	0	0	0.33	0	-0.000712397	NA	Protein of unknown function; induced by alpha pheromone in SpiderM medium
orf19.3413	FGR37	1.33	0	0.66	1.33	0.03785961	NA	Protein lacking an ortholog in S. cerevisiae; transposon mutation affects filamentous growth
orf19.2539	orf19.2539	1.21	0	0	0.6	0.000483364	NA	Protein of unknown function; transcript detected on high-resolution tiling arrays
orf19.1433.1	orf19.1433.1	0	0	2.16	0	0.074561963	NA	Ortholog of C. dubliniensis CD36 : Cd36_22550, Candida tropicalis MYA-3404 : CTRG_01836 and Candida albicans WO-1 : CAWG_01505
orf19.5933	orf19.5933	0.26	0	0.65	0.13	0.038199476	NA	Protein of unknown function; transcript induced by benomyl treatment
orf19.1268	orf19.1268	1.78	0	1.78	0	-0.001127412	NA	Protein of unknown function; transcript detected on high-resolution tiling arrays; rat catheter biofilm induced
orf19.6998	GTT1	0	0	0	0.24	-0.000712397	NA	Putative glutathione S-transferase; downregulated upon adherence to polystyrene; regulated upon white-opaque switching; induced in the presence of human neutrophils; oxidative stress-induced via Cap1p; regulated by Gcn2p, Gcn4p
orf19.937	orf19.937	0	0	0.41	0	-0.000712397	NA	Ortholog of Candida albicans WO-1 : CAWG_04446

Notes:

1.Gene names and descriptions were copied from the CGD database (http://www.candidagenome.org/).

2. log2(*rfg1* Δ/Δ/WT)=Log2 Ratio(FPKM value of *rfg1* Δ/Δ/FPKM value of WT).

3. FPKM, fragments per kilobase of transcript per million.

4. Our analysis was based on the genomic sequence of *C. albicans* SC5314 (Assembly 22)

5. FPKM values of two repeats of each condition are presented (WT-1, WT-2, *rfg1* Δ/Δ-1, *rfg1* Δ/Δ-2).
6.Adjusted p-values (FDR) were calculated using the Cufflinks software (version2.2.1).

Differentially expressed genes (1.5 fold cut-off).

Gene_ID	GENE	FPKM of WT-1	FPKM of WT-2	FPKM of *rfg1* Δ/Δ-1	FPKM of *rfg1* Δ/Δ-2	Log2(*rfg1* Δ/Δ /WT)	p value	Description
**pH response**								
orf19.3499	orf19.3499	44.67	53.98	124.75	172.71	1.223219901	2.88E-05	induced by Mnl1p under weak acid stress; Secreted potein; Hap43-repressed; fluconazole-induced; regulated by Tsa1, Tsa1B under H2O2 stress conditions; Spider biofilm induced
orf19.125	EBP1	73.12	78.03	25.66	25.42	-1.085695681	0.0019217	Cap1, Mnl1 induced; NADPH oxidoreductase; interacts with phenolic substrates (17beta-estradiol); possible role in estrogen response; induced by oxidative, weak acid stress, NO, benomyl, GlcNAc; Hap43-repressed; rat catheter biofilm induced
orf19.3369	MOH1	176.03	219.96	104.09	125.13	-0.674738204	0.031908	induced by alpha pheromone in SpiderM medium and by Mnl1 under weak acid stress;Ortholog of S. cerevisiae Moh1, essential for staionary phase growth; possibly essential (UAU1 method); flow model biofilm induced; Spider biofilm induced
orf19.1189	orf19.1189	143.98	172.74	82.8	89.67	-0.731307231	0.0212394	induced by Mnl1 in weak acid stress; Protein of unknown function; regulated by yeast-hypha switch; 5' UTR intron; repressed by chlamydospore formation in C. albicans and C. dubliniensis; rat catheter, Spider and flow model biofilm induced
orf19.1363	orf19.1363	256.88	294.63	144.2	175.04	-0.698354971	0.0098933	transcript induced by Mnl1 under weak acid stress;Putative protein of unknown function; Plc1-regulated; flow model, rat catheter, Spider biofilm induced
orf19.4779	orf19.4779	294.15	310.95	42.38	26.48	-2.574850689	2.68E-28	induced by Mnl1p under weak acid stress; Putative transporter; slightly similar to the Sit1p siderophore transporter; Gcn4p-regulated; fungal-specific;
orf19.7166	orf19.7166	206.54	171.32	42	35.47	-1.836960776	3.65E-13	induced by Mnl1 under weak acid stress; Predicted mitochondrial cardiolipin-specific phospholipase; upregulated in an azole-resistant strain that overexpresses MDR1; rat catheter and Spider biofilm induced

orf19.6070	ENA2	38.19	39.48	102.65	98.19	1.026333534	0.0016294	Putative sodium transporter; induced by ciclopirox olamine; alkaline induced by Rim101; repressed by high-level peroxide stress; induced in oral candidiasis clinical isolates; possibly essential gene; rat catheter and Spider biofilm induced
orf19.2770.1	SOD1	126.29	135.14	295.01	318.52	1.07153093	1.70E-06	Cytosolic copper- and zinc-containing superoxide dismutase; role in protection from oxidative stress; required for full virulence; alkaline induced by Rim101; induced by human blood; rat catheter, flow model and Spider biofilm repressed
orf19.7469	ARG1	227.74	282.25	133.26	171.83	-0.644746211	0.0336044	Argininosuccinate synthase; arginine synthesis; Gcn4, Rim101 regulated; induced by amino acid starvation (3-AT), benomyl treatment; stationary phase enriched protein; repressed in alkalinizing medium; rat catheter, Spider biofilm induced
orf19.2356	CRZ2	187.67	215.62	92.81	74.74	-1.074718925	3.29E-05	C2H2 transcription factor; crz1 not crz2 null mutation suppresses fluconazole resistance of homozygous cka2 null; Rim101-repressed at pH 8; required for yeast cell adherence to silicone substrate; Spider biofilm induced
orf19.4026	HIS1	64.19	65.86	22.53	24.21	-0.982805408	0.009188	ATP phosphoribosyl transferase; enzyme of histidine biosynthesis; acid upregulated/alkaline repressed by Rim101; regulated by Gcn2, Gcn4; strain CA9 is a his1 mutant; flow model biofilm induced; Spider biofilm repressed
orf19.3829	PHR1	48.3	57.76	189.87	183.25	1.462878326	9.37E-09	Cell surface glycosidase; may act on cell-wall beta-1,3-glucan prior to beta-1,6-glucan linkage; role in systemic, not vaginal virulence (neutral, not low pH); high pH or filamentation induced; Bcr1-repressed in RPMI a/a biofilm
**Transport associated**								
orf19.4384.1	orf19.4384.1	45.82	63.57	125.17	124.73	0.908940144	0.0049957	Putative MFS transporter; predicted ORF in Assembly 20; removed from Assembly 21; restored based on conservation among several Candida species
orf19.4384	HXT5	189.07	267.93	357.37	431.34	0.670210808	0.0150234	colony morphology-related gene regulation by Ssn6;Putative sugar transporter; induced by ciclopirox olamine; Snf3-induced; alkaline repressed; possibly essential gene
orf19.84	CAN3	102.04	116.4	43.96	46.18	-0.984672524	0.0022263	Predicted amino acid transmembrane transporter; transcript regulated by white-opaque switch; Hap43-repressed gene

orf19.6000	CDR1	953.28	1052.4	285.52	236.18	-1.801551654	3.60E-27	Multidrug transporter of ABC superfamily; transports phospholipids in an in-to-out direction; induced by beta-estradiol, progesterone, corticosteroid, or cholesterol; Spider biofilm induced
orf19.918	CDR11	231.21	229.9	92.85	87.23	-1.172145875	7.85E-07	Putative transporter of PDR subfamily of ABC family; Gcn4-regulated; induced by Rim101 at pH 8; Spider biofilm induced
orf19.5079	CDR4	316.73	338.33	103.18	87.77	-1.56112212	2.08E-13	Putative ABC transporter superfamily; fluconazole, Sfu1, Hog1, core stress response induced; caspofungin repressed; fluconazole resistance not affected by mutation or correlated with expression; rat catheter and flow model biofilm induced
orf19.6577	FLU1	81.24	99.01	32.49	34.09	-1.052587913	0.0019217	Multidrug efflux pump of the plasma membrane; MDR family member of the MFS (major facilitator superfamily) of transporters; involved in histatin 5 efflux; fungal-specific (no human/murine homolog)
orf19.3668	HGT2	951.58	1097.34	651.25	593.43	-0.684956267	0.0002654	Putative MFS glucose transporter; 20 member C. albicans glucose transporter family; 12 probable membrane-spanning segments; expressed in rich medium with 2% glucose; rat catheter and Spider biofilm induced
orf19.3749	OPT3	371.1	341.6	229.75	218.23	-0.616337188	0.0091955	Oligopeptide transporter; transcript induced by macrophage phagocytosis, BSA or peptides; fluconazole-induced; induced by Rim101 at pH 8; virulence-group-correlated expression; Hap43-repressed; Spider biofilm induced
orf19.6117	orf19.6117	401.74	420.93	274.26	250.61	-0.60265472	0.0091918	S. pombe ortholog SPAC5D6.04 is a predicted auxin family transmembrane transporter; ketoconazole and hypoxia induced
orf19.7554	orf19.7554	78.15	59.7	24.44	18.16	-1.131649334	0.0015537	Transporter; similar to the Sit1 siderophore transporter; induced by nitric oxide independent of Yhb1; repressed during chlamydospore formation in C. albicans and C. dubliniensis; rat catheter biofilm repressed
orf19.6937	PTR22	923.93	838.03	598.61	539.51	-0.600891121	0.0011281	Oligopeptide transporter involved in uptake of di-/tripeptides; regulated by Stp2 and Stp3; transcript induced upon phagocytosis by macrophage; repressed by Rim101 at pH 8; flow model biofilm induced
orf19.6992	QDR2	108.79	77.17	39.23	33.82	-0.989187313	0.0040016	Predicted MFS membrane transporter, member of the drug:proton antiporter (12 spanner) (DHA1) family; Spider biofilm induced

orf19.5759	SNQ2	244.98	233.91	13.68	13.51	-3.045335908	3.28E-33	Protein similar to S. cerevisiae Snq2p transporter; member of PDR subfamily of ABC family; transposon mutation affects filamentation; benomyl-induced transcription; detected at yeast-form cell plasma membrane by mass spec
orf19.1783	YOR1	117.84	88.47	34.09	19.02	-1.388561995	1.04E-05	Protein similar to S. cerevisiae Yor1; ABC-type plasma membrane transporter involved in resistance to aureobasidin A; white cell type-specific transcript; Spider biofilm induced
**Cell wall and GPI-anchored protein**								
orf19.4975	HYR1	8.34	8.41	129.86	75.38	2.162124141	2.33E-12	GPI-anchored hyphal cell wall protein; macrophage-induced; repressed by neutrophils; resistance to killing by neutrophils, azoles; regulated by Rfg1, Efg1, Nrg1, Tup1, Cyr1, Bcr1, Hap43; Spider and flow model biofilm induced
orf19.5760	IHD1	11.77	12.24	100.1	55.34	1.591145048	1.75E-06	GPI-anchored protein; alkaline, hypha-induced; regulated by Nrg1, Rfg1, Tup1 and Tsa1, Tsa1B in minimal media at 37; oralpharyngeal candidasis induced ; Spider biofilm induced; regulated in Spider biofilms by Tec1, Efg1, Ndt80, Rob1, Brg1
orf19.4651	PGA53	205	263.61	410.32	534.24	0.874428401	0.0002654	GPI-anchored cell surface protein of unknown function; greater mRNA abundance observed in a cyr1 homozygous null mutant than in wild type
orf19.1097	ALS2	98.25	138.97	31.08	34.91	-1.361233731	8.36E-06	ALS family protein; role in adhesion, biofilm formation, germ tube induction; expressed at infection of human buccal epithelial cells; putative GPI-anchor; induced by ketoconazole, low iron and at cell wall regeneration; regulated by Sfu1p
orf19.4555	ALS4	307.92	342.88	54.19	52.86	-2.212227314	7.61E-24	GPI-anchored adhesin; role in adhesion, germ tube induction; growth, temperature regulated; expressed during infection of human buccal epithelial cells; repressed by vaginal contact; biofilm induced; repressed during chlamydospore formation
orf19.5635	PGA7	162.57	177.08	89.79	101.07	-0.706203922	0.021711	GPI-linked hyphal surface antigen; induced by ciclopirox olamine, ketoconazole, Rim101 at pH 8; Hap43, fluconazole; flow model biofilm induced; Spider biofilm induced; required for RPMI biofilm; Bcr1-induced in a/a biofilm

orf19.6594	PLB3	315.21	337.72	157.37	165.57	-0.911889761	4.70E-05	GPI-anchored cell surface phospholipase B; possibly secreted; fungal-specific (no mammalian homolog); induced by Tbf1; fluconazole-induced; possible essential gene (UAU1 method); Spider and flow model biofilm induced
orf19.5636	RBT5	658.28	586.18	421.39	332.88	-0.677320652	0.0008769	GPI-linked cell wall protein; hemoglobin utilization; Rfg1, Rim101, Tbf1, Fe regulated; Sfu1, Hog1, Tup1, serum, alkaline pH, antifungal drugs, geldamycin repressed; Hap43 induced; required for RPMI biofilms; Spider biofilm induced
orf19.5305	RHD3	140.36	146.58	26.3	29.73	-1.787138231	6.13E-11	GPI-anchored yeast-associated cell wall protein; induced in high iron; clade-associated gene expression; not essential for cell wall integrity; fluconazole-repressed; flow model and Spider biofilm repressed
orf19.4246	orf19.4246	135.68	128.91	260.63	238.03	0.786853142	0.0016294	colony morphology-related gene regulation by Ssn6p;Protein with similarity to S. cerevisiae Ykr070w; Tn mutation affects filamentation; Hog1-repressed; induced during cell wall regeneration; possibly essential
orf19.3651	PGK1	1011.27	1180.7	1782.05	2007.03	0.722048963	8.48E-05	Phosphoglycerate kinase; localizes to cell wall and cytoplasm; antigenic in murine/human infection; flow model biofilm, Hog1-, Hap43-, GCN-induced; repressed upon phagocytosis; repressed in Spider biofilms by Bcr1, Ndt80, Rob1, Brg1
orf19.5602	BMT6	152.7	158.08	63.01	52.99	-1.162882572	2.19E-05	Beta-mannosyltransferase; beta-1,2-mannosylation of phospholipomannan; member of a 9-member family including Bmt1, Bmt2, Bmt3, and Bmt4 with roles in mannosylation of cell wall phosphopeptidomannan; flow model biofilm induced
orf19.3672	GAL10	134.35	202.17	68.36	93.8	-0.842341115	0.0103657	UDP-glucose 4-epimerase; galactose utilization; mutant has cell wall defects and increased filamentation; GlcNAc-, fluconazole- and ketoconazole-induced; stationary phase enriched protein; rat catheter and flow model biofilm induced
orf19.220	PIR1	543.42	640.51	258.35	280.95	-1.040925855	1.29E-07	1,3-beta-glucan-linked cell wall protein; N-mannosylated, O-glycosylated by Pmt1; cell wall defect in het mutant; Hog1/fluconazole/hypoxia induced; iron/Efg1/Plc1/temp regulated; flow model biofilm induced; hyphal, Spider biofilm repressed

orf19.54	RHD1	45.99	49.93	16.73	10.24	-1.049759526	0.0079754	Putative beta-mannosyltransferase required for the addition of beta-mannose to the acid-labile fraction of cell wall phosphopeptidomannan; 9-gene family memebr; regulated on yeast-hypha and white-opaque switches; Spider biofilm repressed
orf19.3575	CDC19	348.37	399.97	683.46	801.37	0.889674268	7.18E-06	Pyruvate kinase at yeast cell surface; Gcn4/Hog1/GlcNAc regulated; Hap43/polystyrene adherence induced; repressed by phagocytosis/farnesol; hyphal growth role; stationary phase enriched; flow model biofilm induced; Spider biofilm repressed
orf19.4477	CSH1	138.3	132.87	247.5	231.63	0.706650524	0.0072284	Aldo-keto reductase; role in fibronectin adhesion, cell surface hydrophobicity; regulated by temperature, growth phase, benomyl, macrophage interaction; azole resistance associated; Spider biofilm induced; rat catheter biofilm repressed
orf19.7021	GPH1	445.82	467.6	820.03	841.99	0.789680657	1.04E-05	Putative glycogen phosphorylase; role in glycogen metabolism; regulated by Ssk1, Mig1, Tup1, Hap43; fluconazole-induced; localizes to cell surface of hyphae, not yeast; stationary phase enriched protein; Spider biofilm induced
orf19.6814	TDH3	4212.17	5183.46	7763.38	9056.67	0.768059539	2.47E-05	NAD-linked glyceraldehyde-3-phosphate dehydrogenase; binds fibronectin, laminin; at cell surface; antigenic in infection; farnesol-repressed; stationary phase-enriched; GlcNAc-induced; flow model biofilm induced; Spider biofilm repressed
**Filamentous and biofilm formation**								
orf19.1321	HWP1	1.85	3.21	142.03	69.04	2.535442865	4.11E-16	Hyphal cell wall protein; host transglutaminase substrate; opaque-, a-specific, alpha-factor induced; at MTLa side of conjugation tube; virulence complicated by URA3 effects; Bcr1-repressed in RPMI a/a biofilms; Spider biofilm induced
orf19.3374	ECE1	5.36	5.13	336.83	167.52	3.401436212	1.35E-33	Hypha-specific protein; regulated by Rfg1, Nrg1, Tup1, Cph1, Efg1, Hog1, farnesol, phagocytosis; fluconazole-induced; rat catheter and Spider biofilm induced; flow model biofilm repressed; Bcr1-repressed in RPMI a/a biofilms
orf19.2241	PST1	303.55	364.1	533.1	536.21	0.60287284	0.0087505	Putative 1,4-benzoquinone reductase; hyphal-induced; regulated by Cyr1, Ras1, Efg1, Nrg1, Rfg1, Tup1; Hap43-induced; Spider biofilm induced

orf19.6202	RBT4	99.2	119.76	246.98	270.71	1.053514204	1.45E-05	Pry family protein; required for virulence in mouse systemic/rabbit corneal infections; not filamentation; mRNA binds She3, is localized to hyphal tips; Hap43-induced; in both yeast and hyphal culture supernatants; Spider biofilm induced
orf19.4551	CTN1	134.96	101.61	39.39	27.28	-1.376024394	4.27E-06	Carnitine acetyl transferase; required for growth on nonfermentable carbon sources, not for hyphal growth or virulence in mice; induced in macrophage; macrophage/pseudohyphal-repressed after 16 hr; rat catheter, Spider biofilm induced
orf19.638	FDH1	112.46	107.97	72.1	40.17	-0.75514795	0.0437278	Formate dehydrogenase; oxidizes formate to CO2; Mig1 regulated; induced by macrophages; fluconazole-repressed; repressed by Efg1 in yeast, not hyphal conditions; stationary phase enriched; rat catheter and Spider biofilm induced
orf19.3475	orf19.3475	129.51	145.74	67.35	54.54	-0.955537852	0.001518	Described as a Gag-related protein; hyphal induced; downregulation correlates with clinical development of fluconazole resistance; repressed by nitric oxide, 17-beta-estradiol, ethynyl estradiol
orf19.4899	GCA1	35.16	52.55	95.62	97.65	0.829357341	0.0237632	Extracellular/plasma membrane-associated glucoamylase; expressed in rat oral infection; regulated by carbohydrates, pH, galactose; promotes biofilm matrix formation; flow model biofilm induced; Bcr1 repressed in RPMI a/a biofilms
orf19.946	MET14	36.55	42.63	93.06	90.77	0.879470653	0.0125988	Putative adenylylsulfate kinase; predicted role in sulfur metabolism; possibly adherence-induced; protein present in exponential and stationary growth phase yeast; F-12/CO2 biofilm induced
orf19.2966	orf19.2966	68.9	66.36	128.4	135.86	0.771469551	0.0167692	Predicted dienelactone hydrolase domain; clade-associated gene expression; farnesol-downregulated; rat catheter biofilm repressed
orf19.5620	orf19.5620	49.62	46.55	96.13	115.14	0.849081713	0.0134423	Stationary phase enriched protein; Gcn4-regulated; induced by amino acid starvation (3-AT), benomyl or in azole-resistant strain that overexpresses MDR1; flow model biofilm induced; rat catheter biofilm repressed; overlaps orf19.5621
orf19.6816	orf19.6816	72.23	65.72	130.69	120.82	0.690345557	0.0472288	Putative xylose and arabinose reductase; flow model biofilm induced; Spider biofilm repressed
orf19.715	orf19.715	43.11	47.98	140.35	106.22	1.089626591	0.0003752	Protein of unknown function; Hap43-induced; rat catheter and Spider biofilm induced

orf19.716	orf19.716	57.08	43.38	117.37	97.82	0.839199355	0.0141678	Protein of unknown function; Hap43-induced; regulated by Nrg1, Tup1; repressed by alpha pheromone in SpiderM medium; Spider biofilm induced; Bcr1-repressed in RPMI a/a biofilms
orf19.6169	ATO1	15.27	13.59	66.86	32.82	0.982036066	0.0163056	Putative fungal-specific transmembrane protein; induced by Rgt1; Spider biofilm induced
orf19.542	HXK2	277.77	365.01	515.33	711.75	0.80836423	0.0010556	gene regulation by Ssn6;Hexokinase II; antigenic in humans; repressed by human neutrophils; Efg1-regulated; fluconazole-induced; present in exponential and stationary growth phase; flow model biofilm induced; Spider biofilm repressed
orf19.1709	orf19.1709	120.16	110.71	227.17	231.74	0.847163182	0.0008509	colony morphology-related gene regulation by Ssn6; Sterol carrier domain protein; alkaline downregulated; Spider biofilm induced
orf19.395	ENO1	2021.6	2407.78	3889.34	4272.27	0.810972276	1.87E-06	Enolase; glycolysis and gluconeogenesis; major cell-surface antigen; binds host plasmin/plasminogen; immunoprotective; Hap43, phagocytosis induced; farnesol-repressed; flow model biofilm induced; Spider biofilm repressed; may be essential
orf19.4618	FBA1	552.2	621.63	976.4	1018.97	0.697691184	0.0001875	Fructose-bisphosphate aldolase; glycolytic enzyme; antigenic in murine/human infection; regulated by yeast-hypha switch; induced by Efg1, Gcn4, Hog1, fluconazole; phagocytosis-repressed; flow model biofilm induced; Spider biofilm repressed
orf19.999	GCA2	89.61	110.26	269.47	273.97	1.225172434	1.31E-07	Predicted extracellular glucoamylase; induced by ketoconazole; possibly essential, disruptants not obtained by UAU1 method; promotes biofilm matrix formation; Spider biofilm induced; Bcr1-induced in RPMI a/a biofilms
orf19.5024	GND1	527.26	560.98	904.71	1032.19	0.760005083	4.62E-05	6-phosphogluconate dehydrogenase; soluble in hyphae; farnesol, macrophage-induced protein; antigenic in mice; dual localization to cytosol and peroxisomes depends on alternative splicing; rat catheter and Spider biofilm repressed
orf19.903	GPM1	1336.3	1579.15	2315.28	2692.15	0.712865209	0.0001363	Phosphoglycerate mutase; surface protein that binds host complement Factor H and FHL-1; antigenic; fluconazole, or amino acid starvation (3-AT) induced, farnesol-repressed; Hap43, flow model biofilm induced; Spider biofilm repressed

orf19.3590	IPP1	451.34	463.2	763.45	822.83	0.726369482	0.0001027	Putative inorganic pyrophosphatase; antigenic; soluble protein in hyphae; macrophage-induced protein; protein present in exponential and stationary phase yeast; possibly essential (UAU1 method); sumoylation target; Spider biofilm repressed
orf19.6561	LAT1	277.39	278.98	493.88	467.72	0.711190591	0.0005535	Putative dihydrolipoamide acetyltransferase component (E2) of pyruvate dehydrogenase complex; sumoylation target; Spider biofilm repressed
orf19.3358	LSC1	249.79	235.72	393.24	436.22	0.687658579	0.0021738	Putative succinate-CoA ligase subunit; induced by high iron; fluconazole-induced; protein present in exponential and stationary growth phase yeast cultures; Spider biofilm repressed
orf19.5645	MET15	120.5	117.77	243.02	241.21	0.873965931	0.0003796	O-acetylhomoserine O-acetylserine sulfhydrylase; sulfur amino acid synthesis; immunogenic; Hog1, adherence-induced; brown color of mutant in Pb(2+) medium a visual selection; chlamydospore formation induced, F-12/CO2 biofilm induced
orf19.1203.1	orf19.1203.1	175.25	139.17	275.24	244.08	0.626622551	0.0217184	Predicted dolichol-phosphate mannosyltransferase subunit; flow model and Spider biofilm repressed
orf19.1344	orf19.1344	236.09	185.18	434.81	348.42	0.788874357	0.0005258	Protein of unknown function; fluconazole-induced; Spider biofilm induced
orf19.1691	orf19.1691	70.15	93.83	248.65	318.46	1.482828973	8.07E-10	Plasma-membrane-localized protein; filament induced; Hog1, ketoconazole, fluconazole and hypoxia-induced; regulated by Nrg1, Tup1, Upc2; induced by prostaglandins; flow model biofilm induced; rat catheter and Spider biofilm repressed
orf19.1946	orf19.1946	86.9	76.57	159.28	142	0.713961766	0.0237632	Similar to an aldose 1-epimerase-related protein; antigenic during murine systemic infection; protein present in exponential and stationary phase yeast cultures; Hap43-induced; Spider biofilm repressed
orf19.2191.1	orf19.2191.1	159.88	148.23	234.04	282.77	0.643680765	0.0212642	Ortholog of C. dubliniensis CD36 : Cd36_11730, C. parapsilosis CDC317 : CPAR2_104195, Candida tenuis NRRL Y-1498 : CANTEDRAFT_109454 and Debaryomyces hansenii CBS767 : DEHA2C10076g
orf19.3469	orf19.3469	17.2	15.93	47.53	44.83	0.874782764	0.0428768	S. cerevisiae ortholog Stb1 has a role in regulation of MBF-specific transcription at Start; induced in a cyr1 null mutant; Spider biofilm induced
orf19.4530.1	orf19.4530.1	150.91	197.27	277.85	333.01	0.691252692	0.0121355	Protein of unknown function; regulated by Nrg1, Tup1; Spider and flow model biofilm induced

orf19.5614	orf19.5614	648.04	621.05	1149.88	1059.36	0.739464771	7.85E-06	Putative ribonuclease H1; possibly an essential gene, disruptants not obtained by UAU1 method; flow model biofilm induced; Spider biofilm induced
orf19.6435	orf19.6435	181.3	164.22	278.72	295.05	0.642200066	0.0121355	Highly conserved subunit of mitochondrial pyruvate carrier; Hap43-repressed; Spider biofilm repressed
orf19.676	orf19.676	98.19	101.95	190.37	187.12	0.764866379	0.0068998	Microtubule-binding protein of the cortical microtubule; delays exit from mitosis when the spindle is abnormally oriented; Spider biofilm repressed
orf19.7152	orf19.7152	199.09	193.37	394.9	379.69	0.875500411	2.75E-05	Protein similar to Aspergillus CYSK O-acetylserine sulfhydrylase, suggesting that C. albicans uses an O-acetyl-serine (OAS) pathway of sulfur assimilation; upregulated in biofilm; protein level decreases in stationary phase cultures
orf19.5021	PDX1	292.71	295.88	482.32	458.87	0.606361012	0.005707	Pyruvate dehydrogenase complex protein X; essential component of the mitochondrial pyruvate dehydrogenase complex; role in the respiratory pathway; protein present in exponential and stationary growth phase yeast; Spider biofilm repressed
orf19.3888	PGI1	377.55	432.93	641.39	667.19	0.620215996	0.0035068	Glucose-6-phosphate isomerase; enzyme of glycolysis; antigenic; Efg1-regulated; induced upon adherence to polystyrene; repressed by phagocytosis, human neutrophils; flow model biofilm induced; rat catheter and Spider biofilm repressed
orf19.2841	PGM2	188.65	156.25	301.75	273.22	0.64319793	0.0121355	Ortholog of S. cerevisiae Pgm2; induced in planktonic culture; Tye7p-regulated; flow model biofilm induced; rat catheter biofilm repressed
orf19.1655	PXP2	101.39	101.36	198.1	175.97	0.742352125	0.0093371	Putative acyl-CoA oxidase; enzyme of fatty acid beta-oxidation; induced during macrophage infection; opaque specific transcript; putative peroxisome targeting signal; Spider biofilm induced
orf19.2947	SNZ1	480.07	452.73	775.81	747.91	0.648783521	0.0004506	Stationary phase protein; vitamin B synthesis; induced byyeast-hypha switch, 3-AT or in azole-resistant strain overexpressing MDR1; soluble in hyphae; regulated by Gcn4, macrophage; Spider biofilm induced; rat catheter biofilm repressed
orf19.7544	TLO1	89.6	96.95	157.89	169.08	0.662654726	0.0398361	Member of a family of telomere-proximal genes of unknown function; hypha-induced expression; rat catheter biofilm repressed

orf19.6414.3	TPM2	444.41	430.52	682.55	716.85	0.618671427	0.0016601	Putative tropomyosin isoform 2; regulated by Gcn4; repressed by amino acid starvation; macrophage-induced; protein levels decrease in stationary cells; Hap43-induced; rat catheter and Spider biofilm repressed
orf19.6601.1	YKE2	239.69	269.89	385.88	448.42	0.625484534	0.0109473	Possible heterohexameric Gim/prefoldin protein complex subunit; role in folding alpha-tubulin, beta-tubulin, and actin; transcript induced by yeast-to-hypha switch; regulated by Nrg1, Tup1; Spider and flow model biofilm induced
orf19.111	CAN2	759.76	521.38	477.03	298.23	-0.649376975	0.0153362	Basic amino acid permease; arginine metabolism; regulated by Nrg1/Tup1; caspofungin, flucytosine induced; colony morphology-related regulation by Ssn6; Hap43-repressed; rat catheter and Spider biofilm induced; promoter bound by Efg1
orf19.97	CAN1	672.28	557.19	300.15	232.34	-1.110984169	3.78E-09	Basic amino acid permease; complements lysine transport mutation; 10 predicted transmembrane regions, 3 predicted N-glycosylation sites; phagocytosis by macrophages induces transcript; rat catheter, Spider and flow model biofilm induced
orf19.6586	orf19.6586	60	50.15	12.61	8.6	-1.32810869	0.0002089	Ssn6 colony morphology-related regulation; Protein of unknown function; transcript induced by benomyl or in azole-resistant strain overexpressing MDR1; induced by NO; Hap43-repressed; rat catheter and flow model biofilm induced
orf19.3618	YWP1	384.74	512.26	87.42	93.85	-2.001097495	7.54E-21	Ssk1/Ssn6/Efg1/Efh1/Hap43 regulated; Secreted yeast wall protein; possible role in dispersal in host; mutation increases adhesion and biofilm formation; propeptide; growth phase, phosphate,mRNA binds She3; flow and Spider biofilm repressed
orf19.4773	AOX2	1983.24	2459.62	221.26	284.98	-2.853126641	5.47E-60	Alternative oxidase; cyanide-resistant respiration; induced by antimycin A, oxidants; growth; Hap43, chlamydospore formation repressed; rat catheter, Spider biofilm induced; regulated in Spider biofilms by Bcr1, Tec1, Ndt80, Brg1
orf19.2849	AQY1	2051.43	2319.82	967.48	1224.32	-0.929531204	3.32E-07	Aquaporin water channel; osmotic shock resistance, WT freeze tolerance; virulent in mice; flucytosine repressed; flow model/RPMI/Spider/rat catheter biofilm induced; required for RPMI biofilm formation; Bcr1-induced in a/a RPMI biofilms

orf19.6447	ARF1	398.24	340.76	246.79	224.38	-0.597339833	0.0123647	ADP-ribosylation factor; probable GTPase involved in intracellular transport; one of several C. albicans ADP-ribosylation factors; N-myristoylprotein; substrate of Nmt1; Spider biofilm induced
orf19.1325	ECM38	90.53	69.53	35.63	25.84	-0.976269803	0.0068998	Putative gamma-glutamyltransferase; alkaline upregulated; Spider biofilm induced; possibly an essential gene, disruptants not obtained by UAU1 method
orf19.4631	ERG251	471.71	535.83	272.75	295.42	-0.76283888	0.0003451	C-4 sterol methyl oxidase; role in ergosterol biosynthesis; Hap43-induced; ketoconazole-induced; amphotericin B, caspofungin repressed; possibly essential gene, disruptants not obtained by UAU1 method; Spider biofilm repressed
orf19.6489	FMP45	394.57	389.12	144.48	128.31	-1.374355878	4.10E-12	Predicted membrane protein induced during mating; mutation confers hypersensitivity to toxic ergosterol analog, to amphotericin B; alkaline repressed; repressed by alpha pheromone in SpiderM medium; rat catheter, Spider biofilm induced
orf19.4526	HSP30	369.62	517.77	62.09	61.69	-2.404844238	1.70E-27	Putative heat shock protein; fluconazole repressed; amphotericin B induced; Spider biofilm induced; rat catheter biofilm induced
orf19.3664	HSP31	433.33	425.38	4.53	3.78	-4.407375541	5.93E-69	Putative 30 kda heat shock protein; repressed during the mating process; rat catheter biofilm induced
orf19.6844	ICL1	1279.15	1068.06	751.17	514.54	-0.830902103	2.11E-05	Isocitrate lyase; glyoxylate cycle enzyme; required for virulence in mice; induced upon phagocytosis by macrophage; farnesol regulated; Pex5-dependent peroxisomal localization; stationary phase enriched; rat catheter, Spider biofilm induced
orf19.5288	IFE2	2899.57	2897.38	1790.49	1652.55	-0.728924232	6.71E-07	Putative alcohol dehydrogenase; yeast-enriched transcript; Efg1-regulated; induced by prostaglandins, Hog1, fluconazole; rat catheter biofilm induced
orf19.7080	LEU2	62.44	68.61	3.67	5.01	-1.953884251	2.09E-09	Isopropyl malate dehydrogenase; leucine biosynthesis; induced by human whole blood or PMNs; protein level decreases in stationary phase; GlcNAc-induced protein; flow model biofilm repressed
orf19.1562	orf19.1562	131.6	159.51	64.01	76.49	-0.863187552	0.0056682	Protein of unknown function; flow model biofilm induced; Spider biofilm induced; repressed by alpha pheromone in SpiderM medium
orf19.2048	orf19.2048	1109	1124.02	565.38	588.71	-0.902992252	3.36E-08	Proten of unknown function; transcript positively regulated by Sfu1; Hap43 repressed; Spider biofilm induced

orf19.2049	orf19.2049	40.18	47.04	14.39	10.4	-1.023911762	0.0111556	Plasma membrane-associated protein; heterozygous null mutant displays sensitivity to virgineone; Spider biofilm induced
orf19.2175	orf19.2175	307.94	317.7	132.93	122.85	-1.149131655	1.08E-07	Putative mitochondrial cell death effector; induced by nitric oxide; Spider biofilm induced; rat catheter biofilm repressed
orf19.2220	orf19.2220	199.59	181.08	40.73	27.58	-1.954541045	1.55E-14	Protein of unknown function; Hap43-repressed gene; by Rgt1; repressed in Spider biofilms by Bcr1, Tec1, Brg1, Rob1 and induced by Efg1, Ndt80; Spider biofilm induced
orf19.2308	orf19.2308	166.74	191.08	84.44	97.34	-0.830301929	0.0040513	Putative 6-phosphofructo-2-kinase; catalyzes synthesis of fructose-2,6-bisphosphate; Hap43-repressed; flow model, rat catheter and Spider biofilm induced
orf19.2726	orf19.2726	275.83	217.16	120.27	82.28	-1.101266825	1.05E-05	Putative plasma membrane protein; Plc1-regulated; Spider biofilm induced
orf19.3337	orf19.3337	132.68	119.07	67.15	60.44	-0.80168147	0.0125988	Protein of unknown function; merged with orf19.3338; rat catheter, flow and Spider model biofilm induced; promoter bound by Bcr1, Efg1, Ndt80, and Rob1; orf19.3338 Bcr1-repressed in RPMI a/a biofilms
orf19.409	orf19.409	1151.13	1392.13	628.92	780.23	-0.789920817	9.93E-05	Ortholog of S. cerevisiae Aim38/Rcf2, cytochrome c oxidase subunit; plasma membrane localized; Hap43-repressed; induced in oralpharyngeal candidasis; flow model biofilm induced; Spider biofilm repressed
orf19.4886	orf19.4886	183.14	184.94	67.63	59.88	-1.270457061	6.39E-07	Putative adhesin-like protein; Hap43-repressed; rat catheter and Spider biofilm induced
orf19.4921.1	orf19.4921.1	732.85	853.75	215.05	264.11	-1.571424762	3.54E-17	Protein of unknown function; Spider biofilm repressed
orf19.5813	orf19.5813	676.02	797.63	417.17	461.4	-0.69695149	0.0008541	Putative adhesin-like protein; upregulated during growth in the mouse cecum; flow model, rat catheter and Spider biofilm induced
orf19.6688	orf19.6688	1115.45	1113.51	132.77	112.19	-2.924805451	1.99E-69	Protein of unknown function; expression decreases by benomyl treatment or in an azole-resistant strain overexpressing MDR1; Spider biofilm induced
orf19.670.2	orf19.670.2	5911.9	6002.02	3062.84	3116.17	-0.915151668	2.74E-11	Protein of unknown function; hypoxia, Hap43-repressed; ketoconazole induced; induced in oralpharyngeal candidasis; 16h flow model biofilm repressed, late-stage flow model biofilm induced; rat catheter and Spider biofilm induced
orf19.6720	orf19.6720	161.43	136.11	75.87	73.26	-0.824627691	0.0064394	P-Loop domain-containing protein of unknown function; transposon mutation affects filamentous growth; Spider biofilm induced

orf19.6770	orf19.6770	87.25	96.49	39.37	39.94	-0.907244194	0.0095131	protein with ENTH Epsin domain, N-terminal; Spider biofilm repressed
orf19.6840	orf19.6840	291.04	247.93	170.87	144.68	-0.689039842	0.0074459	Protein of unknown function; transcript detected in high-resolution tiling arrays; transcription induced by alpha pheromone in SpiderM medium; Spider and early-stage flow model biofilm induced
orf19.7279.1	orf19.7279.1	301.41	341.16	207.56	175.66	-0.673710332	0.0072477	Protein of unknown function; Spider biofilm induced
orf19.7283	orf19.7283	393.64	433.88	121.97	126.26	-1.55305502	9.80E-15	Protein of unknown function; rat catheter and Spider biofilm induced
orf19.7296	orf19.7296	562.57	582.38	198.61	140.24	-1.589411698	1.23E-16	Putative cation conductance protein; similar to stomatin mechanoreception protein; plasma-membrane localized; induced by Rgt1; rat catheter and Spider biofilm induced
orf19.732	orf19.732	249.78	192.02	126.68	92.06	-0.868470486	0.0013747	Possible dehydrogenase; flow model biofilm induced; rat catheter biofilm induced; Spider biofilm induced
orf19.5663	RCH1	120.6	86.48	39.89	22.8	-1.235203747	0.0001323	Plasma membrane protein; involved in regulation of cytosolic calcium homeostasis; null mutation confers sensitivity to calcium and resistance to azoles and terbinafine; rat catheter biofilm induced
orf19.23	RTA3	98.46	95.22	46.3	36.54	-0.923895181	0.0068998	Similar to S. cerevisiae Rta1 (role in 7-aminocholesterol resistance) and Rsb1 (flippase); putative drug-responsive regulatory site; induced by fluphenazine, estradiol, ketoconazole, caspofungin; rat catheter biofilm induced
orf19.802	UGA1	103.42	81.91	50.85	31.26	-0.867904892	0.0168409	Putative GABA transaminase; transcription regulated by Mig1 and Tup1; stationary phase enriched protein; rat catheter and Spider biofilm induced
orf19.677	CHO1	705.47	726.95	1597.01	1599.91	1.079176877	6.51E-13	Putative phosphatidylserine synthase; ortholog of S. cerevisiae CHO1; transposon mutation affects filamentous growth; regulated by Nrg1, Tup1
orf19.6445	ECI1	180.36	155.78	291.5	265.47	0.633181995	0.0142848	Protein similar to S. cerevisiae Eci1p, which is involved in fatty acid oxidation; transposon mutation affects filamentous growth; expression is regulated upon white-opaque switching
orf19.6116	GLK4	81.49	93.01	156.13	161.04	0.705252028	0.0260715	Putative glucokinase; decreased expression in hyphae compared to yeast-form cells

Transcriptional regulation

orf19.610	EFG1	65.08	68.95	136.63	121.64	0.752725541	0.0222747	bHLH transcription factor; required for white-phase cell type, RPMI and Spider biofilm formation, hyphal growth, cell-wall gene regulation; roles in adhesion, virulence; Cph1 and Efg1 have role in host cytokine response; binds E-box
orf19.467	WOR3	64.83	72.2	159.09	161.89	0.994568502	0.0003853	Transcription factor; modulator of white-opaque switch; induced in opaque cells; promoter bound by Wor1; overexpression at 25 degr shifts cells to opaque state; deletion stabilizes opaque cells at higher temperatures; Spider biofilm induced
orf19.5338	GAL4	218.41	263.88	439.51	540.78	0.900899583	5.35E-05	Zn(II)2Cys6 transcription factor; involved in control of glycolysis; ortholog of S. cerevisiae Gal4, but not involved in regulation of galactose utilization genes; caspofungin repressed; Spider biofilm repressed
orf19.1826	MDM34	117.83	134.25	71.12	60.41	-0.766542229	0.021711	Putative zinc finger transcription factor; macrophage-downregulated; flow model biofilm induced; flow model and Spider biofilm induced
orf19.909	STP4	367.92	374.26	178.17	151.6	-1.058318837	3.45E-07	C2H2 transcription factor; colony morphology-related gene regulation by Ssn6;induced in core caspofungin response; induced by 17-beta-estradiol, ethynyl estradiol; rat catheter and Spider biofilm induced
orf19.7570	UGA3	156.71	184.95	75.78	93.05	-0.846976524	0.0042801	Zn(II)2Cys6 transcription factor; required for utilization of gamma-aminobutyrate (GABA) as a nitrogen source; mutants display decreased CFU in mouse kidneys; Spider biofilm induced
orf19.1168	ZCF3	122.52	142.89	80.8	64.02	-0.718637242	0.0353415	Zn(II)2Cys6 domain transcription factor; required for filamentous growth, resistance to rapamycin and flucytosine; possibly an essential gene, disruptants not obtained by UAU1 method; Hap43-repressed; Spider and flow model biofilm induced
**Stress response**								
orf19.3803	MNN22	43.51	48.42	13.49	14.11	-1.011997918	0.0115639	Alpha-1,2-mannosyltransferase; required for normal cell wall mannan; regulated by Tsa1, Tsa1B at 37 deg; repressed in core stress response; NO, Hog1 induced; confers sensitivity to cell wall perturbing agents; Spider biofilm repressed

orf19.734	GLK1	246.9	278.4	455.99	469.9	0.729436846	0.0007637	colony morphology-related gene regulation by Ssn6;Putative glucokinase; transcript regulated upon yeast-hyphal switch; Efg1 regulated; fluconazole-induced; induced in core stress response; GlcNAc-induced protein
orf19.2613	ECM4	278.43	262.01	448.71	435.95	0.639762395	0.0032414	Cytoplasmic glutathione S-transferase; regulated by Nrg1, Tup1; induced in core stress response, in cyr1 or ras1 mutant (yeast or hyphal cells); Tn mutation affects filamentous growth; stationary phase enriched; Spider biofilm induced
orf19.5437	RHR2	519.48	680.33	264.14	354.75	-0.851920245	0.0003451	Glycerol 3-phosphatase; roles in osmotic tolerance, glycerol accumulation in response to salt; Spider/flow model biofilm induced; regulated by macrophage, stress, yeast-hyphal switch, pheromone, Gcn4, Hog1, Nrg1, Tup1
orf19.2762	AHP1	736.4	793.04	1212.8	1307.76	0.662534446	0.0002531	Ssk1/Nrg1/Tup1/Ssn6/Hog1 regulated; Alkyl hydroperoxide reductase; immunogenic; fluconazole-induced; amphotericin B, caspofungin, alkaline repressed; core stress response induced; flow model biofilm induced; rat catheter biofilm repressed
orf19.4082	DDR48	3729.24	4059.42	7418.01	8639.67	0.970999333	5.23E-10	Immunogenic stress-associated protein; filamentation regulated; induced by benomyl/caspofungin/ketoconazole or in azole-resistant strain; Hog1, farnesol, alkaline repressed; stationary phase enriched; Spider, flow model biofilm induced
orf19.4371	TAL1	273.48	288.92	482.93	559.36	0.797593128	0.000147	Transaldolase; protein present in exponential and stationary growth phase yeast cultures; oxidative stress-induced via Cap1; induced by nitric oxide independent of Yhb1p; sumoylation target; rat catheter biofilm repressed
orf19.4754	ZWF1	88.58	77.19	175.47	148.34	0.786090092	0.0086238	Glucose-6-phosphate dehydrogenase; antigenic in mice; activity induced by O2 or oxidizing agents H2O2, menadione, macrophage; caspofungin repressed; induced in core stress response; regulated by Gcn2, Gcn4; rat catheter biofilm repressed
orf19.822	HSP21	1186.15	1402.85	556.17	581.69	-1.112567849	6.13E-11	Small heat shock protein; role in stress response and virulence; fluconazole-downregulated; induced in cyr1 or ras1 mutant; stationary phase enriched protein; detected in some, not all, biofilm extracts; Spider biofilm induced

orf19.1149	MRF1	199.22	192.18	104.55	122.21	-0.677150127	0.021711	Putative mitochondrial respiratory protein; induced by farnesol, benomyl, nitric oxide, core stress response; oxidative stress-induced via Cap1; stationary-phase enriched protein; Spider biofilm induced
orf19.5818	SUR2	1145.93	1465.62	709.74	932.41	-0.616429702	0.0102999	Putative ceramide hydroxylase; predicted enzyme of sphingolipid biosynthesis; regulated by Tsa1, Tsa1B under H2O2 stress conditions; Spider and flow model biofilm induced
orf19.7306	orf19.7306	99.87	81.01	38.09	40.39	-0.90224791	0.0103664	Aldo-keto reductase; increased transcript associated with MDR1 overexpression, benomyl or long-term fluconazole treatment; overexpression does not affect drug or oxidative stress sensitivity; stationary phase enriched; flow biofim repressed
orf19.4907	orf19.4907	61.52	70.17	24.72	18.41	-1.067763905	0.0036171	Putative protein of unknown function; Hap43p-repressed gene; increased transcription is observed upon fluphenazine treatment; possibly transcriptionally regulated by Tac1p; induced by nitric oxide; fungal-specific (no human/murine homolog
**Others**								
orf19.5622	GLC3	210.08	202.44	449.83	454.28	1.017254046	1.93E-07	colony morphology-related gene regulation by Ssn6; Putative 1,4-glucan branching enzyme; fluconazole-induced; stationary phase enriched protein
orf19.338	orf19.338	238.19	240.1	444.42	431.49	0.78114864	0.0001627	shows colony morphology-related gene regulation by Ssn6p; Putative glycoside hydrolase; stationary phase enriched protein; Hog1p-downregulated;
orf19.7043.1	ACB1	1073.05	1020.96	1480.34	1869.45	0.621856735	0.0016899	Protein similar to a region of acyl-coenzyme-A-binding protein; amphotericin B repressed; regulated by Nrg1, Tup1; Hap43-induced
orf19.953.1	COF1	356.15	411.47	601.13	682.62	0.665971458	0.0021396	Putative cofilin; macrophage-induced protein; protein present in exponential and stationary-phase yeast cells, but higher amounts in stationary phase
orf19.787	orf19.787	116.49	117.79	205.49	223.51	0.741454132	0.0071413	Protein of unknown function
orf19.5112	TKL1	144.68	170	286.58	324.81	0.82762235	0.0007321	Putative transketolase; localizes to surface of yeast cells, not hyphae; soluble protein in hyphae; transcript regulated by Nrg1, Mig1, and Tup1; antigenic in human or murine infection; possibly essential (by UAU1 method)
orf19.2661	TLO34	297.53	288.94	476.98	520.26	0.688189949	0.00117	Non-telomeric member of a family of telomere-proximal genes of unknown function; predicted N-terminus is extended, relative to that of other family members; overlaps orf19.2662

orf19.362	TLO9	36.22	25.61	58.5	86.8	0.836824238	0.0395897	Member of a family of telomere-proximal genes of unknown function; Hap43p-repressed gene
orf19.7513	ALK2	191.41	199.52	46.25	45.04	-1.715827781	4.13E-12	N-Alkane inducible cytochrome P450
orf19.7219	FTR1	546.89	530.47	307.35	321.42	-0.725127066	0.0002784	High-affinity iron permease; required for mouse virulence, low-iron growth; iron, amphotericin B, caspofungin, ciclopirox, Hog1p, Sef1p, Sfu1p, and Hap43p regulated; complements S. cerevisiae ftr1 iron transport; Hap43p-repressed
orf19.1438	orf19.1438	89.68	72.36	3.36	1.53	-2.380806285	3.58E-14	Protein with homology to NADH dehydrogenase; regulated by Sef1p-, Sfu1p-, and Hap43p
orf19.2047	orf19.2047	101.21	100.56	46.02	44.16	-0.897892127	0.0078528	Putative protein of unknown function; Hap43p-repressed gene; mutation confers hypersensitivity to toxic ergosterol analog, and to amphotericin B
orf19.3364	orf19.3364	344.7	295.16	63.87	59.12	-2.040036608	3.74E-21	Ortholog of C. parapsilosis CDC317 : CPAR2_403360, Debaryomyces hansenii CBS767 : DEHA2D00814g, Pichia stipitis Pignal : PICST_32156 and Candida guilliermondii ATCC 6260 : PGUG_04611
orf19.344	orf19.344	50.81	71.06	8.14	5.81	-1.665379556	8.49E-07	Protein of unknown function; upregulated by fluphenazine treatment or in an azole-resistant strain that overexpresses CDR1 and CDR2; transcript possibly regulated by Tac1
orf19.3932.1	orf19.3932.1	128.2	114.95	57.34	50.34	-0.941678397	0.0024024	Has domain(s) with predicted nucleic acid binding, nucleotide binding activity
orf19.4534	orf19.4534	155.64	165.44	94.42	88.92	-0.689299123	0.0277144	Putative UBX-domain (ubiquitin-regulatory domain) protein; macrophage-downregulated gene
orf19.5114.1	orf19.5114.1	2122.21	2030.57	978.64	987.45	-1.033488346	5.20E-13	Ortholog of C. dubliniensis CD36 : Cd36_02630, C. parapsilosis CDC317 : CPAR2_206910, Candida tenuis NRRL Y-1498 : cten_CGOB_00051 and Pichia stipitis Pignal : psti_CGOB_00173
orf19.551	orf19.551	183.6	203.91	92.39	102.95	-0.84786573	0.0021288	Ortholog of C. dubliniensis CD36 : Cd36_29980, C. parapsilosis CDC317 : CPAR2_204170, Candida tenuis NRRL Y-1498 : CANTEDRAFT_110235 and Debaryomyces hansenii CBS767 : DEHA2F17072g
orf19.711	orf19.711	1487.11	1931.41	785.11	1000.89	-0.861524618	3.07E-05	Protein of unknown function; induced by nitric oxide; predicted ORF from Assembly 19; removed from Assembly 20; restored based on transcription data
orf19.733	orf19.733	150.63	133.89	68.92	65.6	-0.885761175	0.0029333	Ortholog(s) have endoplasmic reticulum localization
orf19.847	YIM1	162.64	153.95	94.36	90.01	-0.667109087	0.0354949	Protein similar to protease of mitochondrial inner membrane; increased transcription is observed upon benomyl treatment; macrophage-downregulated gene

Notes:

1.Gene names and descriptions were copied from the CGD database (http://www.candidagenome.org/).
2. log2(*rfg1* Δ/Δ/WT)=Log2 Ratio(FPKM value of *rfg1* Δ/Δ/FPKM value of WT).
3. FPKM, fragments per kilobase of transcript per million.
4. Our analysis was based on the genomic sequence of *C. albicans* SC5314 (Assembly 22)
5. FPKM values of two repeats of each condition are presented (WT-1, WT-2, *rfg1* Δ/Δ-1, *rfg1* Δ/Δ-2).
6.Adjusted p-values (FDR) were calculated using the Cufflinks software (version2.2.1).
7. Differential expression genes must satisfy three criteria "at least one FPKM>20", "fold change >= 1.5 x" and "FDR <= .05" .
